# Benzaldehyde, A New Absorption Promoter, Accelerating Absorption on Low Bioavailability Drugs Through Membrane Permeability

**DOI:** 10.3389/fphar.2021.663743

**Published:** 2021-05-28

**Authors:** Wen Wen, Jie Luo, Ping Li, Wenge Huang, Ping Wang, Shijun Xu

**Affiliations:** ^1^ School of Pharmacy, Chengdu University of Traditional Chinese Medicine, Chengdu, China; ^2^ Institute of Meterial Medica Integration and Transformation for Brain Disorders, Chengdu University of Traditional Chinese Medicine, Chengdu, China

**Keywords:** benzaldehyde, absorption-promoting, membrane permeability, drug interaction, transmembrane transport

## Abstract

Styrax, one of the most famous folk medicines, is a necessary medicine in formulas to help other drugs reach the focal zone and maximize the effectiveness, the mechanism that promotes absorption is not clear yet. This study was carried out to investigate the absorption-promoting effects and the mechanism of benzaldehyde, a key active compound of styrax, on the diffusion rates of drugs with different oral bioavailability. Caco-2 transport experiments were used to investigate the transport rate. Molecular Dynamics Simulation analysis and fluorescence-anisotropy measurements were used to explore the underlying mechanism of absorption-promoting. Validation test *in vivo* was carried out to reveal the absorption-promoting effects of benzaldehyde on high hydrophilicity drugs. Our data indicated that benzaldehyde(50 μM) elevated the cumulative quantity of passively diffusion drugs with high hydrophilicity such as acyclovir and hydrochlorothiazide. MD and membrane fluidity data explained that benzaldehyde can loosen the structure of the lipid bilayer. The validation tests showed that benzaldehyde (140 mg/kg) remarkably increased the C_max_ and AUC0-6 of acyclovir and hydrochlorothiazide *in vivo*. These present studies suggested that benzaldehyde can promote the absorption of drugs with a lower oral bioavailability through disturbing the integrity of lipid bilayer enhanced membrane permeability.

## Introduction

Transmembrane transport plays critical roles for bioavailability of drugs. It is known that the promotion of transmembrane transport could improve bioavailability and the efficacy of drugs. Despite the lipophilicity of a drug molecule as a rate-limiting factor for passive diffusion across biological membranes, how to improve the bioavailability of hydrophilic drugs had gained attention over the years. Some natural medicines had proved to promote the absorption of hydrophilic drugs such as “kaiqiao” medicine; however, the mechanism of absorption-promoting effects is less understood. “Kaiqiao” medicine, one of the most famous folk medicines, has been frequently used for the treatment of cardiovascular diseases and skin problems in Asia and Africa, which have been used for decades in East Asia, India, Africa, and Turkey. “Kaiqiao” means resuscitation and open orifice. According to the theories of traditional Chinese medicine (TCM), the function of “kaiqiao” medicine is to help other drugs reach the focal zone and enhance the effectiveness of other ingredients in a number of formulas.

**GRAPHICAL ABSTRACT:**
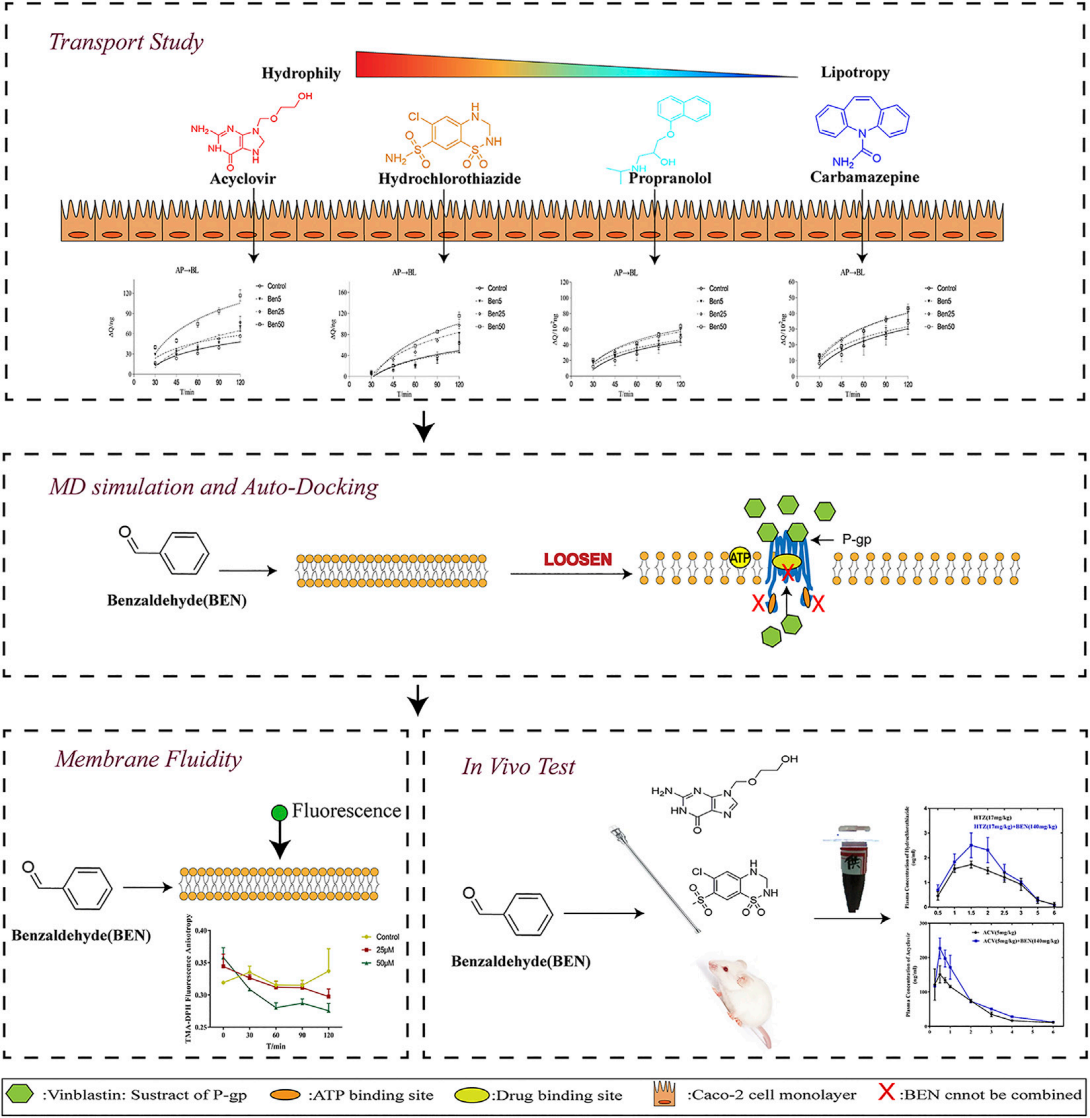
The changes in the fluorescence anisotropy of TMA-DPH in Caco-2 cells with different concentrations of benzaldehyde molecules. (●) HBSS, (■) 25 μM benzaldehyde, (▲) 100 μM benzaldehyde. Data are shown as the mean ± SD.

Styrax is a widely used “kaiqiao” medicine which is called SuHeXiang in China; it is the resin or balsam obtained from the trunk of *Styrax tonkinensis* (Pierre) Craib ex Hart(Ying et al., 2013; Pharmacopoeia, 2020). Styrax is usually used in combination with other drugs to treat acute fainting and chest pain in acute myocardial infarction and can help other drugs reach the target and maximize the effectiveness (Bensky et al., 1993). Modern pharmacological research shows that styrax can promote the absorption of sulpiride by increasing the permeability of the blood–brain barrier and styrax significantly increases the plasma exposure and half-life of warfarin in rats by inhibiting CYP3A (Liu et al., 2012; Dongna et al., 2020; Zhang et al., 2020). Styrax contains several volatile and nonvolatile components, such as cinnamic acid, benzoic acid, benzyl cinnamic acid, benzyl benzoate, benzaldehyde, and benzaldehyde is one of the main volatile components (≈2.21%) (Ye et al., 2005; Faye et al., 2008; Congwen et al., 2009). Benzaldehyde is the main active component of styrax, but the mechanisms of absorption have not been characterized in detail. In our laboratory, we studied vanillin, an active ingredient of another “kaiqiao” medicine benzoinum, (Banerjee and Chattopadhyay, 2019; Pharmacopoeia, 2020). We found that vanillin could enhance the passive transport rate and absorption of drugs with a moderate oral bioavailability by increasing the permeability of the cell membrane (W. Huang et al., 2020). Benzaldehyde and vanillin have similar log *p* values (benzaldehyde-1.48; vanillin-1.21) and both have the same benzaldehyde core. It suggests that benzaldehyde and vanillin may have similar mechanisms in promoting absorption. See from small to big, whether benzaldehyde and other “kaiqiao” medicine can promote drug absorption through a similar pathway is worth exploring.

Active absorption and passive diffusion are dominant mechanisms in drug absorption; the active transport depends on the function of carrier-mediated proteins, such as P-gp (Li et al., 2018). P-gp is a polyspecific energy-dependent active drug efflux pump, limiting the distribution of xenobiotics (e.g., a wide range of therapeutic compounds), which is a major cause of cancer MDR (Silva et al., 2015). While the permeability of cells and the drug lipophilicity (log P) almost determines the passive diffusion rate, high-lipophilicity drugs are more readily absorbed, while drugs with high hydrophilicity means low bioavailability. In our research, we performed a Caco-2 cell bidirectional transport experiment to investigate the effect of benzaldehyde on five different lipophilicity drugs: the transfer of passive (ACV, HTZ, PRO, and CBZ whose lipophilicity increased sequentially) and active (VIN, a substrate of P-gp, that can be excreted by Caco-2 cell) transport of marker drugs. A number of MD simulations were also conducted to explore the interaction between benzaldehyde molecules and membrane structures. A fluidity experiment was also carried out to investigate the effect of benzaldehyde on the membrane fluidity of Caco-2 cells. Finally, we studied the effect of benzaldehyde on the oral bioavailability of ACV and HTZ, the transport rates of which were obviously increased by benzaldehyde in the Caco-2 cell membrane model.

## Materials and Methods Section

### Materials

Benzaldehyde (BEN > 99%) was purchased from Chroma Biotechnology Co., Md (Chengdu, China). Parahydroxybenzoic acid (>98%), benzaldehyde (BEN > 99%), acyclovir (ACV > 99%), carbamazepine (CBZ > 99%), vinblastine (VIN > 99%), hydrochlorothiazide (HTZ >99%), and propranolol hydrochloride (PRO >99%) were purchased from Chengdu Alfa Biotechnology Co., Ltd. (Chengdu, China). TMA-DPH was purchased from MedChemExpress (MA, United States). Acetonitrile and formic acid (HPLC grade) were bought from Fisher Chemical (MA, United States). HBSS and MEM were bought from Hyclone (MA, United States), nonessential amino acids solution was purchased from Sigma-Aldrich (Shanghai, China), and sodium pyruvate was bought from Beijing Solarbio Science and Technology Co., Ltd. (Beijing, China). FBS was purchased from Every Green Biotechnology Co., Ltd. (Zhejiang, China). 0.25% Trypsin–EDTA and penicillin–streptomycin solution was provided by Gibco (NY, United States). DMSO was obtained from Meilunbio (DaLian, China). A 12-well Transwell^®^ plate was purchased from Corning (NY, United States). All other solvents were of analytical agents and aqueous solutions were prepared by double-distilled water.

### Animal Experiment

12 male SD rats (weight range 180–220 g) were purchased from the Chongqing Enswell Biotechnology Co., Ltd. China (China. Certificate: SCXK20180003). All the animals were housed in a suitable temperature (22–28°C) with free access to water and food, and a standard 12 h light/dark cycle. They were acclimatized to their housing environment for 2 days prior to experimentation. All animal care and use of animals were according to the guidelines of Institute of Materia Medica Integration and Transformation for Brain Disorders (No. IBD2018008) and all animals received humane care according to the National Institutes of Health guidelines. And this project has got an approval by the constituted research of Chengdu University of Traditional Chinese Medicine.

### Cell Culture

The Caco-2 cell line was obtained from the Institute of Basic Medical Science of the Chinese Academy of Medical Sciences (Beijing, China). The cell was maintained in MEM with 20% FBS, 1% nonessential amino acids, 1% sodium pyruvate, and 100 U/ml penicillin and streptomycin at 37°C in a humidified atmosphere of 5% CO_2_ medium replaced every 2 days. When the cells reached 75–95% confluence, cultures were passaged by 0.25% trypsin.

### Effect of Benzaldehyde on Transport Study

The transport experiments were carried out according to the recommendations previously described. Caco-2 cells were seeded onto corning ® polycarbonate inserts (1.2 cm^2^, 0.4 µm pore size; Corning, United States) at a density of 2 – 5 × 10^5^ cell/cm^2^, and the medium replaced thrice a week during the first week, then replaced every day until the TEER reached 200Ω/cm^2^ for the transport study, this process required round 21 days under the given cultured conditions.

Before the experiment, TEER was measured Equation 1 to assess the monolayer integrity.

$$\text{TEER}=\left(R_{monolater}-R_{blank}\right)\times A.$$ (1)

In Equation 1, R_*monolater*_ and R_*blank*_ signify the resistance of the cell monolayer and filter membrane, respectively, and *A* is the surface area of the membrane.

The transport of five marker drugs was detected in the absence or presence of benzaldehyde for 12 h, 0.25% DMSO in MEM was used as the control; before the subsequent experiment, the inserts were washed twice and then incubated at 37°C HBSS for 30 min. As described, HBSS was used as a transport buffer in the apical (AP) and basolateral (BL) compartment instead of the medium, and then marker drugs were dissolved in HBSS and added to AP/BL donor; 0.4/0.5 ml HBSS sample was collected from AP/BL donor at 30, 45, 60, 90, and 120 min; and fresh HBSS was replaced in time. All the above operations were carried out at 37°C.

Marker drugs such as ACV, HTZ, PRO, and CBZ were used to assess the permeability of passive diffusion, and the P-gp substrate VIN was used to assess the function of P-gp. All samples were analyzed by HPLC, and the apparent permeability coefficient (Papp) was calculated as follows:

$$P_{app}=\frac{\left(\Delta Q\right)}{\left(\Delta t\right)}\times \frac{1}{A\times C_{0}}.$$ (2)

The (∆Q)/(∆t) is the steady-state flux (μM/min), A is the surface of the filters (cm^2^), and *C*_0_ is the initial concentration of the marker drugs.

The efflux ratio (ER) was calculated as follows:

$$ER=\frac{P_{app\left(B\to A\right)}}{P_{app\left(A\to B\right)}}.$$ (3)

Quantitative determinations of all five marker drugs were performed on an Agilent 1260 HPLC instrument (United States). The separation was accomplished on an AQ-C18 column (5 μm, 4.6 × 250 mm; Welch, China), A gradient elution was provided with 0.1% formic acid aqueous solution as solvent A and acetonitrile as solvent B, at a flow rate of 1.0 ml/min, the elution started at 10:90 (A:B, v/v) and the gradient increased to 80:20 (A:B, v/v) for 22 min, then turned to the initial condition of 10:90 (A:B, v/v) for 5 min, followed by 5 min equilibration, 20 μL sample was injected and the effluent absorbance was measured at 275 nm.

### MD Simulation Analysis of Benzaldehyde on Membrane Structure

In order to investigate the interaction between benzaldehyde molecules and membrane structures, all-atoms simulations were performed in GROMACS 2021.1. (Páll et al., 2014; Abraham et al., 2015). The all-atoms force field was obtained from a standard script on the charmm36-feb2021 (Huang et al., 2017). Here, we used CHARM-GUI as a way to obtain the lipid bilayer containing 128 POPC molecules, with 64 POPC in each leaflet (Jo et al., 2008; Brooks et al., 2009; Lee et al., 2016). The TIP3P model was used to describe water molecules; the ratio of water molecules to POPC membrane molecules was 40:1. Then the benzaldehyde all-atom structure and topology files were obtained from CGenFF (Vanommeslaeghe et al., 2010). Finally, three simulation systems with an increased benzaldehyde concentration were obtained with a pure POPC system, POPC with 30 benzaldehyde molecules, and POPC with 60 benzaldehyde molecules as previous report (Wang and Meng, 2016). In order to clear the effect of benzaldehyde on membrane structure, benzaldehyde molecules are in the upper and lower region of POPC randomly by Packmol package and removed the influences of periodic boundary condition (Martínez et al., 2009).

POPC was firstly equilibrated using the steepest descent method for 10 ps to obtain the lowest energy structure, then 100 ps NVT and 100 ps NPT MDs were carried out. Final-production MDs were lasted 100 ns at NPT conditions. The temperature was set at 310 K by means of V-rescale coupling method with a time constant of 0.1 ps. The pressure was controlled semi-isotropically at 1 atm by using the Parrinello–Rahman method with a coupling time of 2 ps. The long-range Coulomb interaction was taken into account by means of the particle mesh Ewald (PME) technique and the cutoff distance for Coulomb interactions and van der Waals interactions were both set to be 1.4 nm (Wang and Meng, 2016). The total simulation time was 10 ns, and the time step was 2 ps. All topology files and MD running parameter files can be found in Supplement.

### Effect of Benzaldehyde on the Membrane Fluidity of Caco-2 Cells

The fluidity experiment was measured by the cell probe TMA-DPH, and the fluorescence detection was used on an F97Pro18045 fluorimeter with polarizer (Lengguang Technology, China). Caco-2 confluent monolayers were cultured in 25 cm^2^ cell culture flasks and washed three times with HBSS. Then incubated with HBSS (used as control) or benzaldehyde (25 and 50 μM) for 0, 30, 60, 90, and 120 min), after three washing with HBSS, the cells were trypsinized and resuspended in HBSS to 2 × 10^5^ cells/ml. Then the cells were labeled with TMA-DPH by adding 2 ml of 10 μM TMA-DPH stock solution to 2 ml of cell suspension and incubated at 37°C in dark for 20 min to complete labeling. After labeling, the supernatant was removed, and 4 ml HBSS was added to suspend the Caco-2 cells, the fluorescence anisotropy value was detected with a fluorimeter by a previously reported method. The steady-state fluorescent anisotropy value is calculated with the following formula:

$$r=\frac{I_{VV}-GI_{VH}}{I_{VV}+2GI_{VH}},$$

where *r* is the anisotropy value, *I*_*VV*_ and *I*_*VH*_ are the fluorescence intensity values detected by setting excitation polarizer to vertical orientation and the emission polarizer to vertical and horizontal orientation, respectively, and *G* is calculated as *I*_*HV*_ */I*_*HH*_.

### Effect of Benzaldehyde on Bioavailability of ACV and HTZ

After acclimatization, all rats were randomly divided into four groups of three animals each, control (ACV) group, control (HTZ) group, ACV group, and HTZ group. Food was withdrawn 12 h prior to experiments. ACV group and HTZ group were fed with freshly prepared benzaldehyde at the dose of 140 mg/kg, and the other two control groups were fed with physiological saline, and 4 h later, physical mixture was administered by the peroral route (2 ml, dose of 5 mg/kg of ACV, dose of 17 mg/kg of HTZ) by gavage to the corresponding rats, respectively. Details of group dosing can be seen in Table 1. The dosage of drugs was determined from previous researches (Shang et al., 2011; Nair et al., 2014; Al Asmari et al., 2016).

**TABLE 1:** Details of group dosing.

Group	Control (ACV)	ACV	Control (HTZ)	HTZ
i.g	NS	Benzaldehyde (140 mg/kg)	NS	Benzaldehyde (140 mg/kg)
		After 4 hours		
i.g	ACV(5 mg/kg)	ACV(5 mg/kg)	HTZ (17 mg/kg)	HTZ (17 mg/kg)
Time intervals of sample collecting	60, 120, 180, 240, 300, and 360 min		30, 60, 90, 120, 150, 180, 300, and 360 min	

The absorptivity of marker drugs was measured by detecting the plasma ACV and HTZ concentrations from previous research. The blood samples were collected at predetermined time intervals (60, 120, 180, 240, 300, and 360 min) or (30, 60, 90, 120, 150, 180, 300, and 360 min) from retro orbital plexus (∼200 µL), under anesthesia (ether inhalation anesthesia). The collection, preparing, and concentrations of ACV and HTZ were detected with reference to the methods(Shang et al., 2011; Djekic et al., 2018).

### PK Parameters Calculation and Statistical Analysis

We used Thermo Kinetica (Version 5.0, Thermo Electron Corporation., Waltham, MA) to perform the noncompartmental PK analysis to evaluate plasma concentrations vs. time data. The time that reached the maximum plasma concentration (T_max_) and the maximum plasma concentration (C_max_) were evaluated on the basis of observed values. The area under the plasma concentration–time curve, including from 0 h to infinity (AUC0-∞) and from 0 to 6 h, was figured using the linear trapezoidal rule. The mean residence time (MRT) was figured as well. Statistical analysis of PK parameters was by a one-way analysis of variance, followed by the Tukey tests for multiple comparisons. *p* values less than 0.05 were considered statistically significant.

### Statistical Analysis

Data for transport experiments are reported as the mean ± SD of three independent experiments. Linear and nonlinear regressions were analyzed in Microsoft Excel 2016^®^ (United States). *p* values less than 0.05 were considered statistically significant.

## Results

### Effect of Benzaldehyde on Transport Study

**FIGURE 1:**
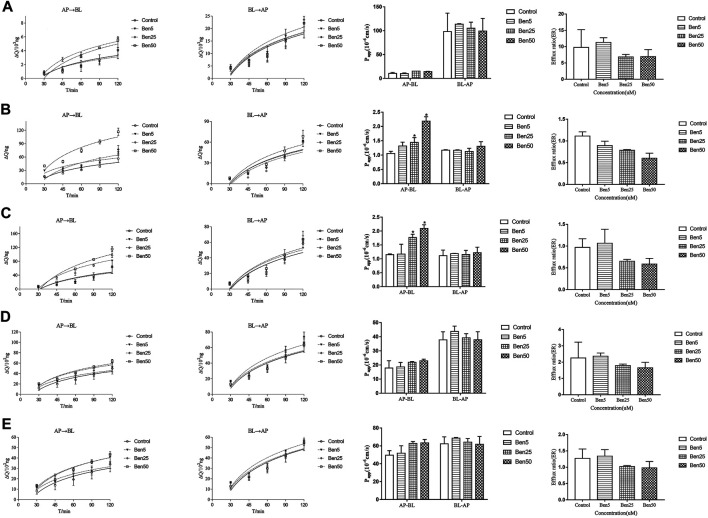
Effect of benzaldehyde on transmembrane transport study. Concentration of benzaldehyde molecules. From the left to right were the accumulation and Papp of marker drugs across AP-BL and BL-AP, the efflux of marker drugs. **(A)** VIN; **(B)** ACV; **(C)** HTZ; **(D)** PRO; and **(E)** CAR. *p < 0.05 meant compared to the control group (no benzaldehyde). Data are shown as the mean ± SD.

The effect of benzaldehyde(5–50 μM) on the transport of marker drugs across the Caco-2 cell monolayers was detected by the bidirectional experiments. The representative chromatogram of marker drugs is displayed in Supplementary File S1. As displayed in Figure 1. Compared to the control group, benzaldehyde (50 μM) significantly increased the Papp values of ACV and HTZ from AP-BL by 2.06- and 1.82-fold at 120 min, benzaldehyde (50 μM) increased the Papp values of PRO and CBZ from AP-BL little by 1.28- and 1.27-fold, respectively, and the cumulative amounts of VIN from AP-BL were increased slightly in the presence of benzaldehyde (25–30%). Nevertheless, benzaldehyde (50 μM) did not obviously affect the Papp values of the all four passive transport drugs from BL-AP. These results suggest that benzaldehyde could significantly enhance the passive diffusion of hydrophobic drugs (ACV and HTZ), which has the tendency to inhibit the active transport of P-gp.

### Influence of Benzaldehyde Concentration on the Membrane

**FIGURE 2:**
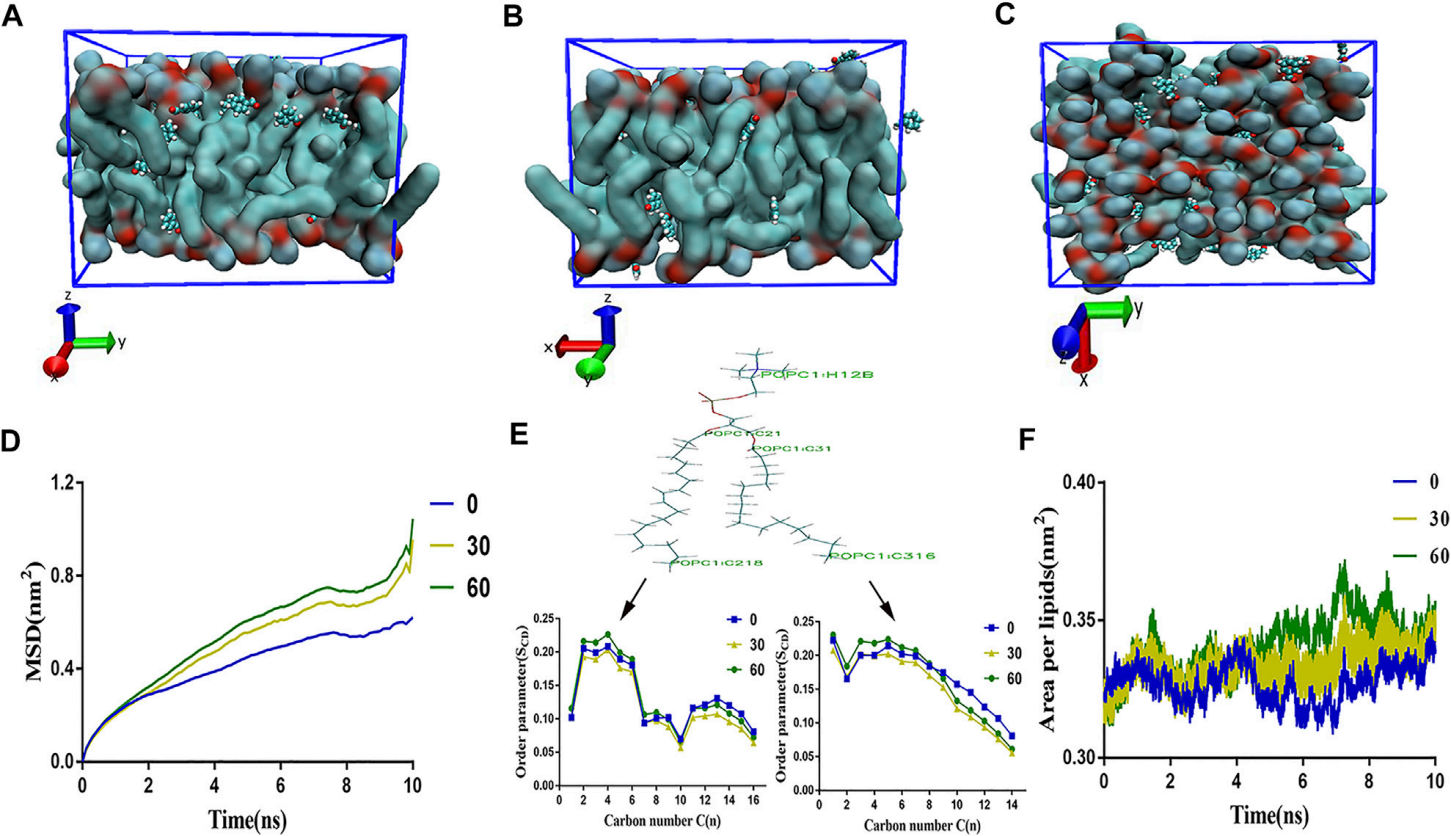
All atoms-MD simulation analysis of atom-bead mapping scheme of different concentration benzaldehyde on membrane structure. **(A–C)** The final simulation snapshots of the benzaldehyde molecules passing through the POPC membrane from the *X*, *Y*, and *Z* axis; **(D)** mean square displacement of the bilayer; **(E)** order parameter of alkyl chain C2_1_–C2_16_ and C3_1_–C3_18_ of POPC molecule; **(F)** area per lipid of the membrane.

In order to clarify the interaction between membrane bilayer with the different concentrations of benzaldehyde. Herein, we evaluated some parameters which could reflect the membrane flexibility, including the area per lipid (APL), lateral diffusion (MSD) bilayer thickness, and the order of alkyl chain C2_1_-C2_16_ and C3_1_-C3_18_. As showed in Figures 2D,E, as the concentration of benzaldehyde elevated, the APL rose and the bilayer thickness, as well as MSD and order parameter of lipids declined. As displayed in Figures 2A–C, the final simulation snapshots of the benzaldehyde molecules passing through the POPC membrane from the *X*, *Y*, *Z* axis (Humphrey et al., 1996). The results demonstrated that the membrane loosened and lipid molecules became flexible with the concentration of benzaldehyde rose.

### Effect of Benzaldehyde on the Membrane Fluidity of Caco-2 Cells

**FIGURE 3:**
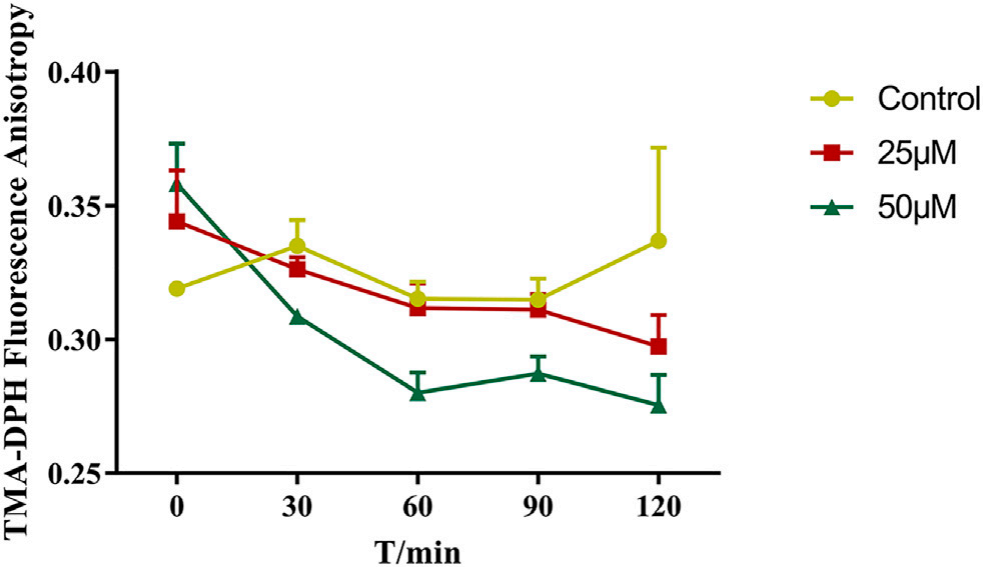
The changes in the fluorescence anisotropy of TMA-DPH in Caco-2 cells with different concentrations of benzaldehyde molecules. (●) HBSS, (■) 25 μM benzaldehyde, (▲) 100 μM benzaldehyde. Data are shown as the mean ± SD.

We further explored the effect of benzaldehyde on the membrane fluidity by the TMA-DPH fluorescence anisotropy. If there is a decrease in TMA-DPH fluorescence anisotropy upon benzaldehyde, it may be suggested that there is an increase in membrane fluidity of the cell bilayer. The R value for control group of TMA-DPH at 0 time point was about 0.319 ± 0.002, and benzaldehyde treatment significantly decreased the anisotropy at different concentration (25 and 50 μM), which were dose-dependent (Figure 3). The results indicated that benzaldehyde can increase the membrane fluidity of cell bilayer and make lipid molecules flexible which similar to the results in MD simulation.

### *In Vivo* Study

**FIGURE 4:**
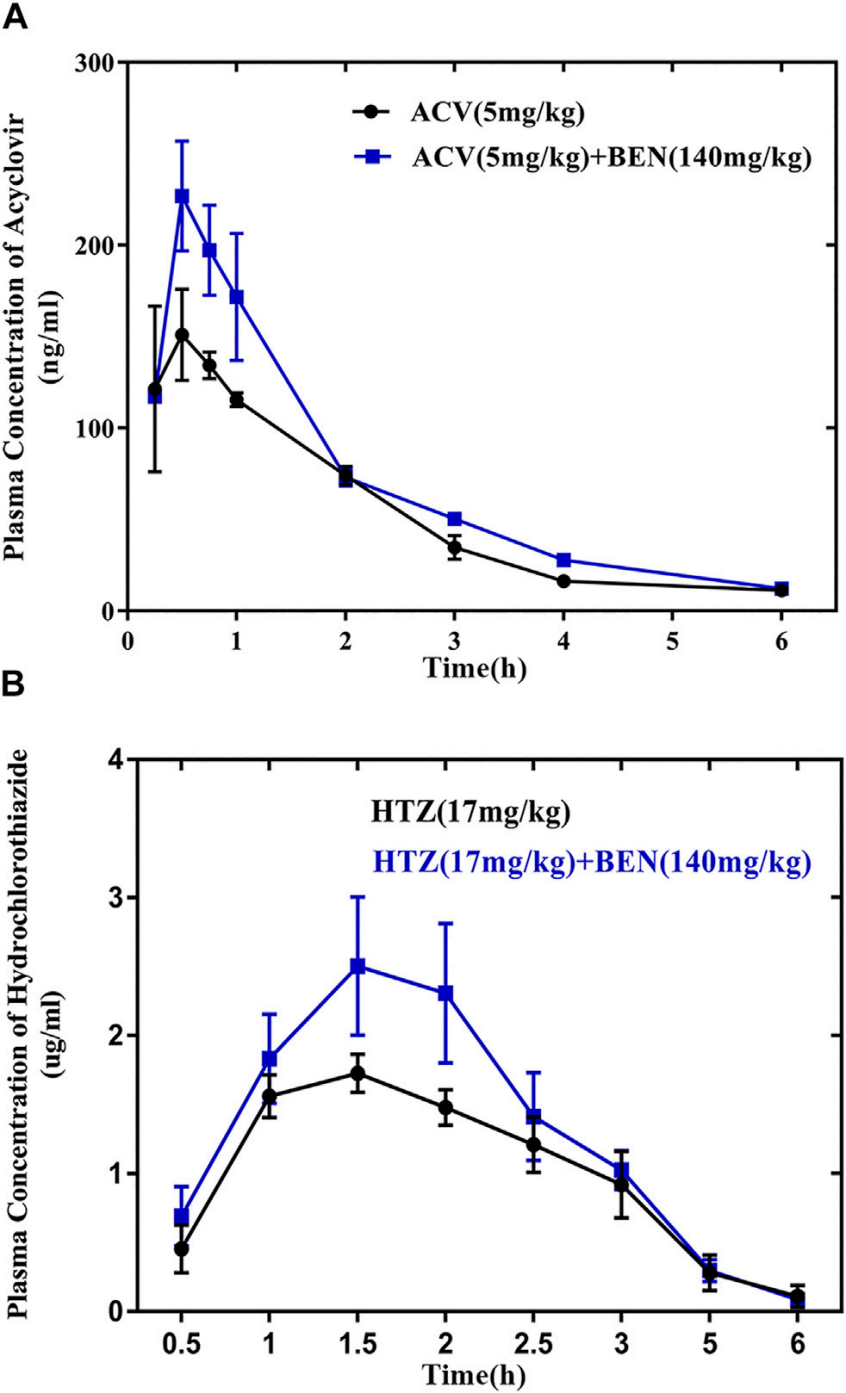
The absorption-promoting effects of benzaldehyde for acyclovir (ACV) and hydrochlorothiazide (HTZ) *in vivo*. **(A)** Mean plasma concentration–time profile of acyclovir with or without benzaldehyde. (●) Control group (acyclovir 5 mg/kg), (■) 140 mg/kg benzaldehyde (acyclovir 5 mg/kg). **(B)** Mean plasma concentration–time profile of hydrochlorothiazide with or without benzaldehyde. (●) Control group (hydrochlorothiazide 17 mg/kg), (■) 140 mg/kg benzaldehyde (hydrochlorothiazide 17 mg/kg).

The plasma concentration vs. time curve of ACV and HTZ after oral administration of ACV and HTZ alone or treat with benzaldehyde before was showed in Figure 4. PK parameters are displayed in Tables 2, 3. Benzaldehyde (140 mg/kg) remarkably enhanced the C_max_ and AUC0-6 of ACV and HTZ by 1.42-fold, 1.28-fold, and 1.42-fold, 1.28-fold, respectively. No obvious difference appeared in T_max_ and MRT between the control group and ACV BEN/HTZ BEN groups. These results show that benzaldehyde could increase oral absorption of ACV and HTZ.

**TABLE 2:** PK parameters of ACV in the absence or presence of benzaldehyde (140 mg kg^-1^).

Parameter	ACV (5 mg kg^-1^)	ACV (5 mg kg^-1^) + BEN (140 mg kg^-1^)
AUC0–6 (ng mL^−1^ h^−1^)	0.33 ± 0.03	0.42 ± 0.03^a^
AUC0–∞ (ng mL^−1^ h^−1^)	0.35 ± 0.02	0.48 ± 0.05^a^
C_max_ (μg ml^−1^)	0.15 ± 0.02	0.23 ± 0.03^a^
T_max_ (h)	0.48 ± 0.03	0.54 ± 0.02
MRT (h)	1.67 ± 0.09	1.71 ± 0.02

AUC0–6: area under the plasma concentration–time curve from 0 to 6 h; AUC0^–∞^: area under the plasma concentration–time curve from 0 h to infinity; C_max_: peak plasma concentration; T_max_: time to reach peak plasma concentration; MRT: mean residence time.

a *p* < 0.05 meant compared to the control group. Data are shown as the mean ± SD.

**TABLE 3:** PK parameters of HTZ in the absence or presence of benzaldehyde (140 mg kg^-1^).

Parameter	HTZ (17 mg kg^-1^)	HTZ (17 mg kg^-1^) + BEN (140 mg kg^-1^)
AUC0–6 (μg mL^−1^ h^−1^)	4.69 ± 0.63	6.11 ± 0.82^a^
AUC0–∞ (μg mL^−1^ h^−1^)	4.89 ± 0.81	6.41 ± 1.16^a^
C_max_ (μg ml^−1^)	1.75 ± 0.11	2.60 ± 0.45^a^
T_max_ (h)	1.42 ± 0.20	1.66 ± 0.23
MRT (h)	2.32 ± 0.18	2.15 ± 0.08

AUC0–6: area under the plasma concentration–time curve from 0 to 6 h; AUC0^–∞^: area under the plasma concentration–time curve from 0 h to infinity; C_max_: peak plasma concentration; T_max_: time to reach peak plasma concentration; MRT: mean residence time.

a *p* < 0.05 meant compared to the control group. Data are shown as the mean ± SD.

## Discussion

In this study, Caco-2 cell transport results showed that benzaldehyde (50 μM) markedly increased the Papp values of ACV and HTZ with dose-dependent, indicated the benzaldehyde can promote the absorption of drugs with high hydrophilicity. MD and membrane fluidity data shows that benzaldehyde can disturb the integrity of cell membrane, highly loosened membrane increased the fluidity of the lipid bilayer and further enhanced absorption of some drugs that not easy to diffuse (e.g., some drugs with high hydrophilicity). The oral bioavailability studies of ACV and HTZ also confirmed this conclusion.

Passive and active transports are two integral ways for drugs absorption (Patel and Patel, 2017). Passive transport mainly depends on lipid solubility of drugs and permeability of cell membrane as the carrier-mediated active transport depends on the function of carrier proteins, such as P-gp. The main barrier for passive diffusion is the phospholipid bilayer of cell membrane. The drug lipophilicity (log P) almost determines the diffusion rate. Actually, the higher the log P of drugs is, the greater diffusion rates are. Furthermore, the log P values of the four marker drugs are sorted as ACV (−1.56) <HTZ (−0.07) <CBZ (2.45) <PRO (3.48). According to the results, we concluded that benzaldehyde has a weak effect of promoting efflux by ER, energy needed for detecting efflux transport (ATP, ADP, and AMP in cells, displayed in Supplementary File S2) and the binding ability of efflux protein (docking affinity energy, displayed in Supplementary File S3). According to previous research, benzaldehyde is a compound showing high affinity (i.e., low K_d_) and effectiveness to cell membrane. This effect could be attributed to the chemical nature of aldehydes because it can interact with the polar head of POPC monolayer and then stabilize on the membrane with its benzene located on the hydrophobic acyl chains of the lipids. Because of the properties of the aldehydes, the cell membrane structure changed from dense to loose. The cell membrane changed to an undisciplined state from a tight state, and then the transport of some drugs becomes easy. P-gp–mediated drug efflux is a major cause of cancer multiple drug resistance because benzaldehyde has a weak effect of promoting efflux (125%). It is not recommended to combine with anticancer drugs and chemotherapeutic agents in clinical treatment, like doxorubicin, paclitaxel, and cyclosporine A, so as not to affect the function of cancer drugs and to prolong the life of patients.

Research on the promotion of drug absorption by “kaiqiao” medicine has attracted people’s attention. Studies have shown that borneol can increase the permeability of isolated rabbit cornea and promote the absorption of giniposide in rabbits (Song et al., 2013), and artificial musk can be rapidly absorbed through the skin by human body (Zhang et al., 2017). What is more, benzaldehyde, vanillin, muscone, and borneol could effectively reverse multidrug resistance via inhibiting the P-gp function and expression pathway (Leng et al., 2018).

Styrax is widely used in the TCM prescription, and benzaldehyde is often used as an artificial essential oil of almond and cherry flavors in foods (Loch et al., 2016). Our study indicated that benzaldehyde remarkably elevated the absorption of lower bioavailability drugs (ACV and HTZ) *in vivo* and *in vitro*. As drugs has similar log P value to ACV and HTZ, for instance, tripotolide and acetaminophen. TCM prescription containing benzaldehyde may lead to elevated drug plasma concentration, meanwhile RIFM safety assessment reported that benzaldehyde displayed no genotoxicity, and it is nontoxic for repeated use at a dose of 200 mg/kg. This suggests that these lower bioavailability drugs can be used in combination with benzaldehyde in the future to reduce the renal burden of drug metabolism. However, risks and benefits coexist: it may lead to increased hepatotoxicity or hallucination of patients and a series of serious side effects caused by excessive drug plasma concentration.

In clinical treatment of TCM, “kaiqiao” medicine is necessary in some first aid formulas to allow the active constituents to work quickly for the treatment of critical illness. Pharmacological research has found that some “kaiqiao” medicine can achieve the aim by inhibiting the efflux function of P-gp. We used cells, computer simulations, and animal experiments to clarify the mechanism of benzaldehyde promoting absorption which is to affect the membrane structure, so as vanillin. Our experiment showed that benzaldehyde is a novel absorbent of hydrophilic drugs, and it may provide the pharmacokinetic basis for the value of the “kaiqiao” medicine in clinical first aid. However, because of the remarkable effect of benzaldehyde in promoting absorption, food or herb containing benzaldehyde should be taken carefully when taking high hydrophilic drugs with significant side effects.

## Conclusion

Benzaldehyde can fortify the absorption of drugs with a lower oral bioavailability *in vitro* and *in vivo* through disturbing the integrity of lipid bilayer enhanced membrane permeability.

## Data Availability

The raw data supporting the conclusion of this article will be made available by the authors, without undue reservation.
