# Supplementary material for: Benzaldehyde, A New Absorption Promoter, Accelerating Absorption on Low Bioavailability Drugs Through Membrane Permeability
Source: Front Pharmacol. 2021 May 28;12:663743. doi: 10.3389/fphar.2021.663743 (PMC8194254; doi:10.3389/fphar.2021.663743)
Supplement: Supplementary file 1 [file DataSheet1.zip › Supplementary file 1.DOCX]

| 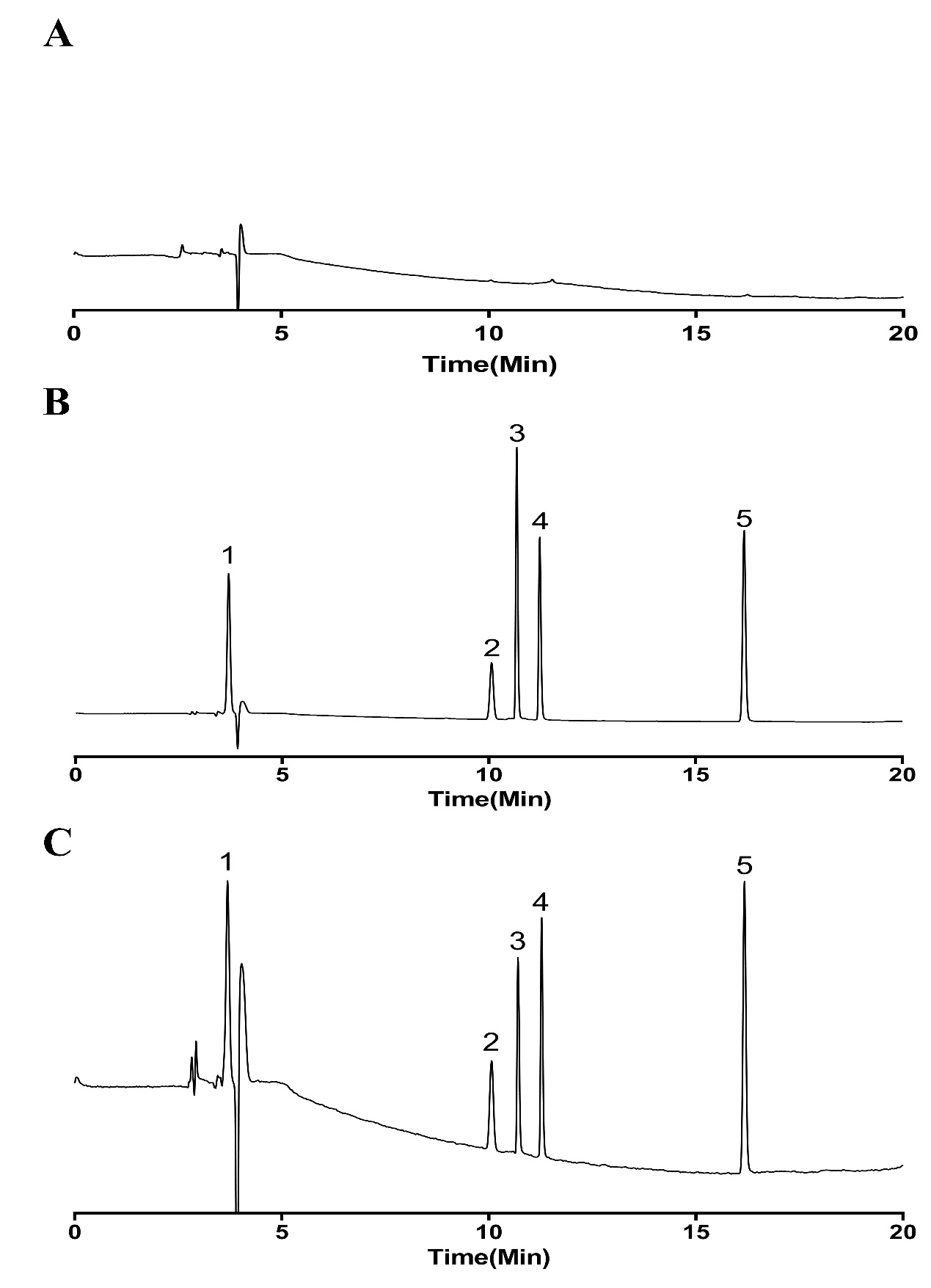 |
| --- |
| **Figure. S1 Typical chromatogram**  (A) HBSS buffer, (B) HBSS buffer spiked with ACV, HTZ, PRO, VIN, CBZ (50μM), (C) HBSS buffer obtained from Caco-2 cell bi-directional transport experiment.  (peak 1: ACV; peak 2: HTZ; peak 3: PRO; peak 4: VIN; peak5: CBZ) |
