# Supplementary material for: Benzaldehyde, A New Absorption Promoter, Accelerating Absorption on Low Bioavailability Drugs Through Membrane Permeability
Source: Front Pharmacol. 2021 May 28;12:663743. doi: 10.3389/fphar.2021.663743 (PMC8194254; doi:10.3389/fphar.2021.663743)
Supplement: Supplementary file 1 [file DataSheet1.zip › Supplementary file 10.DOCX]

define = -DPOSRES -DPOSRES_FC_BB=4000.0 -DPOSRES_FC_SC=2000.0 -DPOSRES_FC_LIPID=1000.0 -DDIHRES -DDIHRES_FC=1000.0

integrator = md

dt = 0.001

nsteps = 125000

nstxtcout = 5000

nstvout = 5000

nstfout = 5000

nstcalcenergy = 100

nstenergy = 1000

nstlog = 1000

;

cutoff-scheme = Verlet

nstlist = 20

rlist = 1.2

vdwtype = Cut-off

vdw-modifier = Force-switch

rvdw_switch = 1.0

rvdw = 1.2

coulombtype = pme

rcoulomb = 1.2

;

tcoupl = berendsen

tc_grps = POPC SOL benza

tau_t = 1.0 1.0 1.0

ref_t = 313.15 313.15 313.15

;

constraints = h-bonds

constraint_algorithm = LINCS

;

nstcomm = 100

comm_mode = linear

comm_grps = POPC SOL benza

;

gen-vel = yes

gen-temp = 313.15

gen-seed = -1

;

refcoord_scaling = com
