# Supplementary material for: Benzaldehyde, A New Absorption Promoter, Accelerating Absorption on Low Bioavailability Drugs Through Membrane Permeability
Source: Front Pharmacol. 2021 May 28;12:663743. doi: 10.3389/fphar.2021.663743 (PMC8194254; doi:10.3389/fphar.2021.663743)
Supplement: Supplementary file 1 [file DataSheet1.zip › Supplementary file 11.DOCX]

;;

;; Generated by CHARMM-GUI FF-Converter

;;

;; Correspondance:

;; jul316@lehigh.edu or wonpil@lehigh.edu

;;

;; GROMACS topology file for POPC

;;

[ moleculetype ]

; name nrexcl

POPC 3

[ atoms ]

; nr type resnr residu atom cgnr charge mass

1 NTL 1 POPC N 1 -0.600000 14.0070 ; qtot -0.600

2 CTL2 1 POPC C12 2 -0.100000 12.0110 ; qtot -0.700

3 HL 1 POPC H12A 3 0.250000 1.0080 ; qtot -0.450

4 HL 1 POPC H12B 4 0.250000 1.0080 ; qtot -0.200

5 CTL5 1 POPC C13 5 -0.350000 12.0110 ; qtot -0.550

6 HL 1 POPC H13A 6 0.250000 1.0080 ; qtot -0.300

7 HL 1 POPC H13B 7 0.250000 1.0080 ; qtot -0.050

8 HL 1 POPC H13C 8 0.250000 1.0080 ; qtot 0.200

9 CTL5 1 POPC C14 9 -0.350000 12.0110 ; qtot -0.150

10 HL 1 POPC H14A 10 0.250000 1.0080 ; qtot 0.100

11 HL 1 POPC H14B 11 0.250000 1.0080 ; qtot 0.350

12 HL 1 POPC H14C 12 0.250000 1.0080 ; qtot 0.600

13 CTL5 1 POPC C15 13 -0.350000 12.0110 ; qtot 0.250

14 HL 1 POPC H15A 14 0.250000 1.0080 ; qtot 0.500

15 HL 1 POPC H15B 15 0.250000 1.0080 ; qtot 0.750

16 HL 1 POPC H15C 16 0.250000 1.0080 ; qtot 1.000

17 CTL2 1 POPC C11 17 -0.080000 12.0110 ; qtot 0.920

18 HAL2 1 POPC H11A 18 0.090000 1.0080 ; qtot 1.010

19 HAL2 1 POPC H11B 19 0.090000 1.0080 ; qtot 1.100

20 PL 1 POPC P 20 1.500000 30.9740 ; qtot 2.600

21 O2L 1 POPC O13 21 -0.780000 15.9994 ; qtot 1.820

22 O2L 1 POPC O14 22 -0.780000 15.9994 ; qtot 1.040

23 OSLP 1 POPC O12 23 -0.570000 15.9994 ; qtot 0.470

24 OSLP 1 POPC O11 24 -0.570000 15.9994 ; qtot -0.100

25 CTL2 1 POPC C1 25 -0.080000 12.0110 ; qtot -0.180

26 HAL2 1 POPC HA 26 0.090000 1.0080 ; qtot -0.090

27 HAL2 1 POPC HB 27 0.090000 1.0080 ; qtot 0.000

28 CTL1 1 POPC C2 28 0.170000 12.0110 ; qtot 0.170

29 HAL1 1 POPC HS 29 0.090000 1.0080 ; qtot 0.260

30 OSL 1 POPC O21 30 -0.490000 15.9994 ; qtot -0.230

31 CL 1 POPC C21 31 0.900000 12.0110 ; qtot 0.670

32 OBL 1 POPC O22 32 -0.630000 15.9994 ; qtot 0.040

33 CTL2 1 POPC C22 33 -0.220000 12.0110 ; qtot -0.180

34 HAL2 1 POPC H2R 34 0.090000 1.0080 ; qtot -0.090

35 HAL2 1 POPC H2S 35 0.090000 1.0080 ; qtot 0.000

36 CTL2 1 POPC C3 36 0.080000 12.0110 ; qtot 0.080

37 HAL2 1 POPC HX 37 0.090000 1.0080 ; qtot 0.170

38 HAL2 1 POPC HY 38 0.090000 1.0080 ; qtot 0.260

39 OSL 1 POPC O31 39 -0.490000 15.9994 ; qtot -0.230

40 CL 1 POPC C31 40 0.900000 12.0110 ; qtot 0.670

41 OBL 1 POPC O32 41 -0.630000 15.9994 ; qtot 0.040

42 CTL2 1 POPC C32 42 -0.220000 12.0110 ; qtot -0.180

43 HAL2 1 POPC H2X 43 0.090000 1.0080 ; qtot -0.090

44 HAL2 1 POPC H2Y 44 0.090000 1.0080 ; qtot 0.000

45 CTL2 1 POPC C23 45 -0.180000 12.0110 ; qtot -0.180

46 HAL2 1 POPC H3R 46 0.090000 1.0080 ; qtot -0.090

47 HAL2 1 POPC H3S 47 0.090000 1.0080 ; qtot 0.000

48 CTL2 1 POPC C24 48 -0.180000 12.0110 ; qtot -0.180

49 HAL2 1 POPC H4R 49 0.090000 1.0080 ; qtot -0.090

50 HAL2 1 POPC H4S 50 0.090000 1.0080 ; qtot 0.000

51 CTL2 1 POPC C25 51 -0.180000 12.0110 ; qtot -0.180

52 HAL2 1 POPC H5R 52 0.090000 1.0080 ; qtot -0.090

53 HAL2 1 POPC H5S 53 0.090000 1.0080 ; qtot 0.000

54 CTL2 1 POPC C26 54 -0.180000 12.0110 ; qtot -0.180

55 HAL2 1 POPC H6R 55 0.090000 1.0080 ; qtot -0.090

56 HAL2 1 POPC H6S 56 0.090000 1.0080 ; qtot 0.000

57 CTL2 1 POPC C27 57 -0.180000 12.0110 ; qtot -0.180

58 HAL2 1 POPC H7R 58 0.090000 1.0080 ; qtot -0.090

59 HAL2 1 POPC H7S 59 0.090000 1.0080 ; qtot 0.000

60 CTL2 1 POPC C28 60 -0.180000 12.0110 ; qtot -0.180

61 HAL2 1 POPC H8R 61 0.090000 1.0080 ; qtot -0.090

62 HAL2 1 POPC H8S 62 0.090000 1.0080 ; qtot 0.000

63 CEL1 1 POPC C29 63 -0.150000 12.0110 ; qtot -0.150

64 HEL1 1 POPC H91 64 0.150000 1.0080 ; qtot 0.000

65 CEL1 1 POPC C210 65 -0.150000 12.0110 ; qtot -0.150

66 HEL1 1 POPC H101 66 0.150000 1.0080 ; qtot 0.000

67 CTL2 1 POPC C211 67 -0.180000 12.0110 ; qtot -0.180

68 HAL2 1 POPC H11R 68 0.090000 1.0080 ; qtot -0.090

69 HAL2 1 POPC H11S 69 0.090000 1.0080 ; qtot 0.000

70 CTL2 1 POPC C212 70 -0.180000 12.0110 ; qtot -0.180

71 HAL2 1 POPC H12R 71 0.090000 1.0080 ; qtot -0.090

72 HAL2 1 POPC H12S 72 0.090000 1.0080 ; qtot 0.000

73 CTL2 1 POPC C213 73 -0.180000 12.0110 ; qtot -0.180

74 HAL2 1 POPC H13R 74 0.090000 1.0080 ; qtot -0.090

75 HAL2 1 POPC H13S 75 0.090000 1.0080 ; qtot 0.000

76 CTL2 1 POPC C214 76 -0.180000 12.0110 ; qtot -0.180

77 HAL2 1 POPC H14R 77 0.090000 1.0080 ; qtot -0.090

78 HAL2 1 POPC H14S 78 0.090000 1.0080 ; qtot 0.000

79 CTL2 1 POPC C215 79 -0.180000 12.0110 ; qtot -0.180

80 HAL2 1 POPC H15R 80 0.090000 1.0080 ; qtot -0.090

81 HAL2 1 POPC H15S 81 0.090000 1.0080 ; qtot 0.000

82 CTL2 1 POPC C216 82 -0.180000 12.0110 ; qtot -0.180

83 HAL2 1 POPC H16R 83 0.090000 1.0080 ; qtot -0.090

84 HAL2 1 POPC H16S 84 0.090000 1.0080 ; qtot 0.000

85 CTL2 1 POPC C217 85 -0.180000 12.0110 ; qtot -0.180

86 HAL2 1 POPC H17R 86 0.090000 1.0080 ; qtot -0.090

87 HAL2 1 POPC H17S 87 0.090000 1.0080 ; qtot 0.000

88 CTL3 1 POPC C218 88 -0.270000 12.0110 ; qtot -0.270

89 HAL3 1 POPC H18R 89 0.090000 1.0080 ; qtot -0.180

90 HAL3 1 POPC H18S 90 0.090000 1.0080 ; qtot -0.090

91 HAL3 1 POPC H18T 91 0.090000 1.0080 ; qtot 0.000

92 CTL2 1 POPC C33 92 -0.180000 12.0110 ; qtot -0.180

93 HAL2 1 POPC H3X 93 0.090000 1.0080 ; qtot -0.090

94 HAL2 1 POPC H3Y 94 0.090000 1.0080 ; qtot 0.000

95 CTL2 1 POPC C34 95 -0.180000 12.0110 ; qtot -0.180

96 HAL2 1 POPC H4X 96 0.090000 1.0080 ; qtot -0.090

97 HAL2 1 POPC H4Y 97 0.090000 1.0080 ; qtot 0.000

98 CTL2 1 POPC C35 98 -0.180000 12.0110 ; qtot -0.180

99 HAL2 1 POPC H5X 99 0.090000 1.0080 ; qtot -0.090

100 HAL2 1 POPC H5Y 100 0.090000 1.0080 ; qtot 0.000

101 CTL2 1 POPC C36 101 -0.180000 12.0110 ; qtot -0.180

102 HAL2 1 POPC H6X 102 0.090000 1.0080 ; qtot -0.090

103 HAL2 1 POPC H6Y 103 0.090000 1.0080 ; qtot 0.000

104 CTL2 1 POPC C37 104 -0.180000 12.0110 ; qtot -0.180

105 HAL2 1 POPC H7X 105 0.090000 1.0080 ; qtot -0.090

106 HAL2 1 POPC H7Y 106 0.090000 1.0080 ; qtot 0.000

107 CTL2 1 POPC C38 107 -0.180000 12.0110 ; qtot -0.180

108 HAL2 1 POPC H8X 108 0.090000 1.0080 ; qtot -0.090

109 HAL2 1 POPC H8Y 109 0.090000 1.0080 ; qtot 0.000

110 CTL2 1 POPC C39 110 -0.180000 12.0110 ; qtot -0.180

111 HAL2 1 POPC H9X 111 0.090000 1.0080 ; qtot -0.090

112 HAL2 1 POPC H9Y 112 0.090000 1.0080 ; qtot 0.000

113 CTL2 1 POPC C310 113 -0.180000 12.0110 ; qtot -0.180

114 HAL2 1 POPC H10X 114 0.090000 1.0080 ; qtot -0.090

115 HAL2 1 POPC H10Y 115 0.090000 1.0080 ; qtot 0.000

116 CTL2 1 POPC C311 116 -0.180000 12.0110 ; qtot -0.180

117 HAL2 1 POPC H11X 117 0.090000 1.0080 ; qtot -0.090

118 HAL2 1 POPC H11Y 118 0.090000 1.0080 ; qtot 0.000

119 CTL2 1 POPC C312 119 -0.180000 12.0110 ; qtot -0.180

120 HAL2 1 POPC H12X 120 0.090000 1.0080 ; qtot -0.090

121 HAL2 1 POPC H12Y 121 0.090000 1.0080 ; qtot 0.000

122 CTL2 1 POPC C313 122 -0.180000 12.0110 ; qtot -0.180

123 HAL2 1 POPC H13X 123 0.090000 1.0080 ; qtot -0.090

124 HAL2 1 POPC H13Y 124 0.090000 1.0080 ; qtot 0.000

125 CTL2 1 POPC C314 125 -0.180000 12.0110 ; qtot -0.180

126 HAL2 1 POPC H14X 126 0.090000 1.0080 ; qtot -0.090

127 HAL2 1 POPC H14Y 127 0.090000 1.0080 ; qtot 0.000

128 CTL2 1 POPC C315 128 -0.180000 12.0110 ; qtot -0.180

129 HAL2 1 POPC H15X 129 0.090000 1.0080 ; qtot -0.090

130 HAL2 1 POPC H15Y 130 0.090000 1.0080 ; qtot 0.000

131 CTL3 1 POPC C316 131 -0.270000 12.0110 ; qtot -0.270

132 HAL3 1 POPC H16X 132 0.090000 1.0080 ; qtot -0.180

133 HAL3 1 POPC H16Y 133 0.090000 1.0080 ; qtot -0.090

134 HAL3 1 POPC H16Z 134 0.090000 1.0080 ; qtot 0.000

[ bonds ]

; ai aj funct b0 Kb

1 2 1

1 5 1

1 9 1

1 13 1

2 3 1

2 4 1

2 17 1

5 6 1

5 7 1

5 8 1

9 10 1

9 11 1

9 12 1

13 14 1

13 15 1

13 16 1

17 18 1

17 19 1

17 23 1

20 21 1

20 22 1

20 23 1

20 24 1

24 25 1

25 26 1

25 27 1

25 28 1

28 29 1

28 30 1

28 36 1

30 31 1

31 32 1

31 33 1

33 34 1

33 35 1

33 45 1

36 37 1

36 38 1

36 39 1

39 40 1

40 41 1

40 42 1

42 43 1

42 44 1

42 92 1

45 46 1

45 47 1

45 48 1

48 49 1

48 50 1

48 51 1

51 52 1

51 53 1

51 54 1

54 55 1

54 56 1

54 57 1

57 58 1

57 59 1

57 60 1

60 61 1

60 62 1

60 63 1

63 64 1

63 65 1

65 66 1

65 67 1

67 68 1

67 69 1

67 70 1

70 71 1

70 72 1

70 73 1

73 74 1

73 75 1

73 76 1

76 77 1

76 78 1

76 79 1

79 80 1

79 81 1

79 82 1

82 83 1

82 84 1

82 85 1

85 86 1

85 87 1

85 88 1

88 89 1

88 90 1

88 91 1

92 93 1

92 94 1

92 95 1

95 96 1

95 97 1

95 98 1

98 99 1

98 100 1

98 101 1

101 102 1

101 103 1

101 104 1

104 105 1

104 106 1

104 107 1

107 108 1

107 109 1

107 110 1

110 111 1

110 112 1

110 113 1

113 114 1

113 115 1

113 116 1

116 117 1

116 118 1

116 119 1

119 120 1

119 121 1

119 122 1

122 123 1

122 124 1

122 125 1

125 126 1

125 127 1

125 128 1

128 129 1

128 130 1

128 131 1

131 132 1

131 133 1

131 134 1

[ pairs ]

; ai aj funct c6 c12

1 18 1

1 19 1

1 23 1

2 6 1

2 7 1

2 8 1

2 10 1

2 11 1

2 12 1

2 14 1

2 15 1

2 16 1

2 20 1

3 5 1

3 9 1

3 13 1

3 18 1

3 19 1

3 23 1

4 5 1

4 9 1

4 13 1

4 18 1

4 19 1

4 23 1

5 10 1

5 11 1

5 12 1

5 14 1

5 15 1

5 16 1

5 17 1

6 9 1

6 13 1

7 9 1

7 13 1

8 9 1

8 13 1

9 14 1

9 15 1

9 16 1

9 17 1

10 13 1

11 13 1

12 13 1

13 17 1

17 21 1

17 22 1

17 24 1

18 20 1

19 20 1

20 26 1

20 27 1

20 28 1

21 25 1

22 25 1

23 25 1

24 29 1

24 30 1

24 36 1

25 31 1

25 37 1

25 38 1

25 39 1

26 29 1

26 30 1

26 36 1

27 29 1

27 30 1

27 36 1

28 32 1

28 33 1

28 40 1

29 31 1

29 37 1

29 38 1

29 39 1

30 34 1

30 35 1

30 37 1

30 38 1

30 39 1

30 45 1

31 36 1

31 46 1

31 47 1

31 48 1

32 34 1

32 35 1

32 45 1

33 49 1

33 50 1

33 51 1

34 46 1

34 47 1

34 48 1

35 46 1

35 47 1

35 48 1

36 41 1

36 42 1

37 40 1

38 40 1

39 43 1

39 44 1

39 92 1

40 93 1

40 94 1

40 95 1

41 43 1

41 44 1

41 92 1

42 96 1

42 97 1

42 98 1

43 93 1

43 94 1

43 95 1

44 93 1

44 94 1

44 95 1

45 52 1

45 53 1

45 54 1

46 49 1

46 50 1

46 51 1

47 49 1

47 50 1

47 51 1

48 55 1

48 56 1

48 57 1

49 52 1

49 53 1

49 54 1

50 52 1

50 53 1

50 54 1

51 58 1

51 59 1

51 60 1

52 55 1

52 56 1

52 57 1

53 55 1

53 56 1

53 57 1

54 61 1

54 62 1

54 63 1

55 58 1

55 59 1

55 60 1

56 58 1

56 59 1

56 60 1

57 64 1

57 65 1

58 61 1

58 62 1

58 63 1

59 61 1

59 62 1

59 63 1

60 66 1

60 67 1

61 64 1

61 65 1

62 64 1

62 65 1

63 68 1

63 69 1

63 70 1

64 66 1

64 67 1

65 71 1

65 72 1

65 73 1

66 68 1

66 69 1

66 70 1

67 74 1

67 75 1

67 76 1

68 71 1

68 72 1

68 73 1

69 71 1

69 72 1

69 73 1

70 77 1

70 78 1

70 79 1

71 74 1

71 75 1

71 76 1

72 74 1

72 75 1

72 76 1

73 80 1

73 81 1

73 82 1

74 77 1

74 78 1

74 79 1

75 77 1

75 78 1

75 79 1

76 83 1

76 84 1

76 85 1

77 80 1

77 81 1

77 82 1

78 80 1

78 81 1

78 82 1

79 86 1

79 87 1

79 88 1

80 83 1

80 84 1

80 85 1

81 83 1

81 84 1

81 85 1

82 89 1

82 90 1

82 91 1

83 86 1

83 87 1

83 88 1

84 86 1

84 87 1

84 88 1

86 89 1

86 90 1

86 91 1

87 89 1

87 90 1

87 91 1

92 99 1

92 100 1

92 101 1

93 96 1

93 97 1

93 98 1

94 96 1

94 97 1

94 98 1

95 102 1

95 103 1

95 104 1

96 99 1

96 100 1

96 101 1

97 99 1

97 100 1

97 101 1

98 105 1

98 106 1

98 107 1

99 102 1

99 103 1

99 104 1

100 102 1

100 103 1

100 104 1

101 108 1

101 109 1

101 110 1

102 105 1

102 106 1

102 107 1

103 105 1

103 106 1

103 107 1

104 111 1

104 112 1

104 113 1

105 108 1

105 109 1

105 110 1

106 108 1

106 109 1

106 110 1

107 114 1

107 115 1

107 116 1

108 111 1

108 112 1

108 113 1

109 111 1

109 112 1

109 113 1

110 117 1

110 118 1

110 119 1

111 114 1

111 115 1

111 116 1

112 114 1

112 115 1

112 116 1

113 120 1

113 121 1

113 122 1

114 117 1

114 118 1

114 119 1

115 117 1

115 118 1

115 119 1

116 123 1

116 124 1

116 125 1

117 120 1

117 121 1

117 122 1

118 120 1

118 121 1

118 122 1

119 126 1

119 127 1

119 128 1

120 123 1

120 124 1

120 125 1

121 123 1

121 124 1

121 125 1

122 129 1

122 130 1

122 131 1

123 126 1

123 127 1

123 128 1

124 126 1

124 127 1

124 128 1

125 132 1

125 133 1

125 134 1

126 129 1

126 130 1

126 131 1

127 129 1

127 130 1

127 131 1

129 132 1

129 133 1

129 134 1

130 132 1

130 133 1

130 134 1

[ angles ]

; ai aj ak funct th0 cth S0 Kub

2 1 5 5

2 1 9 5

2 1 13 5

5 1 9 5

5 1 13 5

9 1 13 5

1 2 3 5

1 2 4 5

1 2 17 5

3 2 4 5

3 2 17 5

4 2 17 5

1 5 6 5

1 5 7 5

1 5 8 5

6 5 7 5

6 5 8 5

7 5 8 5

1 9 10 5

1 9 11 5

1 9 12 5

10 9 11 5

10 9 12 5

11 9 12 5

1 13 14 5

1 13 15 5

1 13 16 5

14 13 15 5

14 13 16 5

15 13 16 5

2 17 18 5

2 17 19 5

2 17 23 5

18 17 19 5

18 17 23 5

19 17 23 5

21 20 22 5

21 20 23 5

21 20 24 5

22 20 23 5

22 20 24 5

23 20 24 5

17 23 20 5

20 24 25 5

24 25 26 5

24 25 27 5

24 25 28 5

26 25 27 5

26 25 28 5

27 25 28 5

25 28 29 5

25 28 30 5

25 28 36 5

29 28 30 5

29 28 36 5

30 28 36 5

28 30 31 5

30 31 32 5

30 31 33 5

32 31 33 5

31 33 34 5

31 33 35 5

31 33 45 5

34 33 35 5

34 33 45 5

35 33 45 5

28 36 37 5

28 36 38 5

28 36 39 5

37 36 38 5

37 36 39 5

38 36 39 5

36 39 40 5

39 40 41 5

39 40 42 5

41 40 42 5

40 42 43 5

40 42 44 5

40 42 92 5

43 42 44 5

43 42 92 5

44 42 92 5

33 45 46 5

33 45 47 5

33 45 48 5

46 45 47 5

46 45 48 5

47 45 48 5

45 48 49 5

45 48 50 5

45 48 51 5

49 48 50 5

49 48 51 5

50 48 51 5

48 51 52 5

48 51 53 5

48 51 54 5

52 51 53 5

52 51 54 5

53 51 54 5

51 54 55 5

51 54 56 5

51 54 57 5

55 54 56 5

55 54 57 5

56 54 57 5

54 57 58 5

54 57 59 5

54 57 60 5

58 57 59 5

58 57 60 5

59 57 60 5

57 60 61 5

57 60 62 5

57 60 63 5

61 60 62 5

61 60 63 5

62 60 63 5

60 63 64 5

60 63 65 5

64 63 65 5

63 65 66 5

63 65 67 5

66 65 67 5

65 67 68 5

65 67 69 5

65 67 70 5

68 67 69 5

68 67 70 5

69 67 70 5

67 70 71 5

67 70 72 5

67 70 73 5

71 70 72 5

71 70 73 5

72 70 73 5

70 73 74 5

70 73 75 5

70 73 76 5

74 73 75 5

74 73 76 5

75 73 76 5

73 76 77 5

73 76 78 5

73 76 79 5

77 76 78 5

77 76 79 5

78 76 79 5

76 79 80 5

76 79 81 5

76 79 82 5

80 79 81 5

80 79 82 5

81 79 82 5

79 82 83 5

79 82 84 5

79 82 85 5

83 82 84 5

83 82 85 5

84 82 85 5

82 85 86 5

82 85 87 5

82 85 88 5

86 85 87 5

86 85 88 5

87 85 88 5

85 88 89 5

85 88 90 5

85 88 91 5

89 88 90 5

89 88 91 5

90 88 91 5

42 92 93 5

42 92 94 5

42 92 95 5

93 92 94 5

93 92 95 5

94 92 95 5

92 95 96 5

92 95 97 5

92 95 98 5

96 95 97 5

96 95 98 5

97 95 98 5

95 98 99 5

95 98 100 5

95 98 101 5

99 98 100 5

99 98 101 5

100 98 101 5

98 101 102 5

98 101 103 5

98 101 104 5

102 101 103 5

102 101 104 5

103 101 104 5

101 104 105 5

101 104 106 5

101 104 107 5

105 104 106 5

105 104 107 5

106 104 107 5

104 107 108 5

104 107 109 5

104 107 110 5

108 107 109 5

108 107 110 5

109 107 110 5

107 110 111 5

107 110 112 5

107 110 113 5

111 110 112 5

111 110 113 5

112 110 113 5

110 113 114 5

110 113 115 5

110 113 116 5

114 113 115 5

114 113 116 5

115 113 116 5

113 116 117 5

113 116 118 5

113 116 119 5

117 116 118 5

117 116 119 5

118 116 119 5

116 119 120 5

116 119 121 5

116 119 122 5

120 119 121 5

120 119 122 5

121 119 122 5

119 122 123 5

119 122 124 5

119 122 125 5

123 122 124 5

123 122 125 5

124 122 125 5

122 125 126 5

122 125 127 5

122 125 128 5

126 125 127 5

126 125 128 5

127 125 128 5

125 128 129 5

125 128 130 5

125 128 131 5

129 128 130 5

129 128 131 5

130 128 131 5

128 131 132 5

128 131 133 5

128 131 134 5

132 131 133 5

132 131 134 5

133 131 134 5

[ dihedrals ]

; ai aj ak al funct phi0 cp mult

5 1 2 17 9

9 1 2 17 9

13 1 2 17 9

2 1 5 6 9

2 1 5 7 9

2 1 5 8 9

2 1 9 10 9

2 1 9 11 9

2 1 9 12 9

5 1 9 10 9

5 1 9 11 9

5 1 9 12 9

2 1 13 14 9

2 1 13 15 9

2 1 13 16 9

5 1 13 14 9

5 1 13 15 9

5 1 13 16 9

9 1 13 14 9

9 1 13 15 9

9 1 13 16 9

3 2 1 5 9

3 2 1 9 9

3 2 1 13 9

4 2 1 5 9

4 2 1 9 9

4 2 1 13 9

1 2 17 18 9

1 2 17 19 9

1 2 17 23 9

3 2 17 18 9

3 2 17 19 9

3 2 17 23 9

4 2 17 18 9

4 2 17 19 9

4 2 17 23 9

6 5 1 9 9

6 5 1 13 9

7 5 1 9 9

7 5 1 13 9

8 5 1 9 9

8 5 1 13 9

10 9 1 13 9

11 9 1 13 9

12 9 1 13 9

2 17 23 20 9

18 17 23 20 9

19 17 23 20 9

21 20 24 25 9

22 20 24 25 9

23 20 24 25 9

17 23 20 21 9

17 23 20 22 9

17 23 20 24 9

20 24 25 26 9

20 24 25 27 9

20 24 25 28 9

24 25 28 29 9

24 25 28 30 9

24 25 28 36 9

26 25 28 29 9

26 25 28 30 9

26 25 28 36 9

27 25 28 29 9

27 25 28 30 9

27 25 28 36 9

25 28 30 31 9

29 28 30 31 9

25 28 36 37 9

25 28 36 38 9

25 28 36 39 9

29 28 36 37 9

29 28 36 38 9

29 28 36 39 9

30 28 36 37 9

30 28 36 38 9

30 28 36 39 9

31 30 28 36 9

28 30 31 32 9

28 30 31 33 9

30 31 33 34 9

30 31 33 35 9

30 31 33 45 9

32 31 33 34 9

32 31 33 35 9

32 31 33 45 9

31 33 45 46 9

31 33 45 47 9

31 33 45 48 9

34 33 45 46 9

34 33 45 47 9

34 33 45 48 9

35 33 45 46 9

35 33 45 47 9

35 33 45 48 9

28 36 39 40 9

37 36 39 40 9

38 36 39 40 9

36 39 40 41 9

36 39 40 42 9

39 40 42 43 9

39 40 42 44 9

39 40 42 92 9

41 40 42 43 9

41 40 42 44 9

41 40 42 92 9

40 42 92 93 9

40 42 92 94 9

40 42 92 95 9

43 42 92 93 9

43 42 92 94 9

43 42 92 95 9

44 42 92 93 9

44 42 92 94 9

44 42 92 95 9

33 45 48 49 9

33 45 48 50 9

33 45 48 51 9

46 45 48 49 9

46 45 48 50 9

46 45 48 51 9

47 45 48 49 9

47 45 48 50 9

47 45 48 51 9

45 48 51 52 9

45 48 51 53 9

45 48 51 54 9

49 48 51 52 9

49 48 51 53 9

49 48 51 54 9

50 48 51 52 9

50 48 51 53 9

50 48 51 54 9

48 51 54 55 9

48 51 54 56 9

48 51 54 57 9

52 51 54 55 9

52 51 54 56 9

52 51 54 57 9

53 51 54 55 9

53 51 54 56 9

53 51 54 57 9

51 54 57 58 9

51 54 57 59 9

51 54 57 60 9

55 54 57 58 9

55 54 57 59 9

55 54 57 60 9

56 54 57 58 9

56 54 57 59 9

56 54 57 60 9

54 57 60 61 9

54 57 60 62 9

54 57 60 63 9

58 57 60 61 9

58 57 60 62 9

58 57 60 63 9

59 57 60 61 9

59 57 60 62 9

59 57 60 63 9

57 60 63 64 9

57 60 63 65 9

61 60 63 64 9

61 60 63 65 9

62 60 63 64 9

62 60 63 65 9

60 63 65 66 9

60 63 65 67 9

64 63 65 66 9

64 63 65 67 9

63 65 67 68 9

63 65 67 69 9

63 65 67 70 9

66 65 67 68 9

66 65 67 69 9

66 65 67 70 9

65 67 70 71 9

65 67 70 72 9

65 67 70 73 9

68 67 70 71 9

68 67 70 72 9

68 67 70 73 9

69 67 70 71 9

69 67 70 72 9

69 67 70 73 9

67 70 73 74 9

67 70 73 75 9

67 70 73 76 9

71 70 73 74 9

71 70 73 75 9

71 70 73 76 9

72 70 73 74 9

72 70 73 75 9

72 70 73 76 9

70 73 76 77 9

70 73 76 78 9

70 73 76 79 9

74 73 76 77 9

74 73 76 78 9

74 73 76 79 9

75 73 76 77 9

75 73 76 78 9

75 73 76 79 9

73 76 79 80 9

73 76 79 81 9

73 76 79 82 9

77 76 79 80 9

77 76 79 81 9

77 76 79 82 9

78 76 79 80 9

78 76 79 81 9

78 76 79 82 9

76 79 82 83 9

76 79 82 84 9

76 79 82 85 9

80 79 82 83 9

80 79 82 84 9

80 79 82 85 9

81 79 82 83 9

81 79 82 84 9

81 79 82 85 9

79 82 85 86 9

79 82 85 87 9

79 82 85 88 9

83 82 85 86 9

83 82 85 87 9

83 82 85 88 9

84 82 85 86 9

84 82 85 87 9

84 82 85 88 9

82 85 88 89 9

82 85 88 90 9

82 85 88 91 9

86 85 88 89 9

86 85 88 90 9

86 85 88 91 9

87 85 88 89 9

87 85 88 90 9

87 85 88 91 9

42 92 95 96 9

42 92 95 97 9

42 92 95 98 9

93 92 95 96 9

93 92 95 97 9

93 92 95 98 9

94 92 95 96 9

94 92 95 97 9

94 92 95 98 9

92 95 98 99 9

92 95 98 100 9

92 95 98 101 9

96 95 98 99 9

96 95 98 100 9

96 95 98 101 9

97 95 98 99 9

97 95 98 100 9

97 95 98 101 9

95 98 101 102 9

95 98 101 103 9

95 98 101 104 9

99 98 101 102 9

99 98 101 103 9

99 98 101 104 9

100 98 101 102 9

100 98 101 103 9

100 98 101 104 9

98 101 104 105 9

98 101 104 106 9

98 101 104 107 9

102 101 104 105 9

102 101 104 106 9

102 101 104 107 9

103 101 104 105 9

103 101 104 106 9

103 101 104 107 9

101 104 107 108 9

101 104 107 109 9

101 104 107 110 9

105 104 107 108 9

105 104 107 109 9

105 104 107 110 9

106 104 107 108 9

106 104 107 109 9

106 104 107 110 9

104 107 110 111 9

104 107 110 112 9

104 107 110 113 9

108 107 110 111 9

108 107 110 112 9

108 107 110 113 9

109 107 110 111 9

109 107 110 112 9

109 107 110 113 9

107 110 113 114 9

107 110 113 115 9

107 110 113 116 9

111 110 113 114 9

111 110 113 115 9

111 110 113 116 9

112 110 113 114 9

112 110 113 115 9

112 110 113 116 9

110 113 116 117 9

110 113 116 118 9

110 113 116 119 9

114 113 116 117 9

114 113 116 118 9

114 113 116 119 9

115 113 116 117 9

115 113 116 118 9

115 113 116 119 9

113 116 119 120 9

113 116 119 121 9

113 116 119 122 9

117 116 119 120 9

117 116 119 121 9

117 116 119 122 9

118 116 119 120 9

118 116 119 121 9

118 116 119 122 9

116 119 122 123 9

116 119 122 124 9

116 119 122 125 9

120 119 122 123 9

120 119 122 124 9

120 119 122 125 9

121 119 122 123 9

121 119 122 124 9

121 119 122 125 9

119 122 125 126 9

119 122 125 127 9

119 122 125 128 9

123 122 125 126 9

123 122 125 127 9

123 122 125 128 9

124 122 125 126 9

124 122 125 127 9

124 122 125 128 9

122 125 128 129 9

122 125 128 130 9

122 125 128 131 9

126 125 128 129 9

126 125 128 130 9

126 125 128 131 9

127 125 128 129 9

127 125 128 130 9

127 125 128 131 9

125 128 131 132 9

125 128 131 133 9

125 128 131 134 9

129 128 131 132 9

129 128 131 133 9

129 128 131 134 9

130 128 131 132 9

130 128 131 133 9

130 128 131 134 9

[ dihedrals ]

; ai aj ak al funct q0 cq

31 30 33 32 2

40 39 42 41 2

#ifdef POSRES

[ position_restraints ]

20 1 0.0 0.0 POSRES_FC_LIPID

#endif

#ifdef DIHRES

[ dihedral_restraints ]

25 36 28 30 1 -120.0 2.5 DIHRES_FC

60 63 65 67 1 0.0 0.0 DIHRES_FC

#endif
