# Supplementary material for: Benzaldehyde, A New Absorption Promoter, Accelerating Absorption on Low Bioavailability Drugs Through Membrane Permeability
Source: Front Pharmacol. 2021 May 28;12:663743. doi: 10.3389/fphar.2021.663743 (PMC8194254; doi:10.3389/fphar.2021.663743)
Supplement: Supplementary file 1 [file DataSheet1.zip › Supplementary file 12.DOCX]

HEADER

TITLE Built with Packmol

REMARK Packmol generated pdb file

REMARK Home-Page: http://m3g.iqm.unicamp.br/packmol

REMARK

ATOM 1 N POPC 1 1.189 -1.765 20.857 1.00 0.00 MEMB N

ATOM 2 C12 POPC 1 1.863 -2.049 19.513 1.00 0.00 MEMB C

ATOM 3 H12A POPC 1 2.029 -3.121 19.414 1.00 0.00 MEMB H

ATOM 4 H12B POPC 1 2.829 -1.561 19.478 1.00 0.00 MEMB H

ATOM 5 C13 POPC 1 -0.073 -2.573 20.960 1.00 0.00 MEMB C

ATOM 6 H13A POPC 1 -0.693 -2.235 21.780 1.00 0.00 MEMB H

ATOM 7 H13B POPC 1 -0.644 -2.468 20.026 1.00 0.00 MEMB H

ATOM 8 H13C POPC 1 0.139 -3.628 21.063 1.00 0.00 MEMB H

ATOM 9 C14 POPC 1 2.079 -2.141 21.994 1.00 0.00 MEMB C

ATOM 10 H14A POPC 1 1.504 -2.334 22.903 1.00 0.00 MEMB H

ATOM 11 H14B POPC 1 2.644 -3.046 21.781 1.00 0.00 MEMB H

ATOM 12 H14C POPC 1 2.778 -1.347 22.201 1.00 0.00 MEMB H

ATOM 13 C15 POPC 1 0.820 -0.322 20.951 1.00 0.00 MEMB C

ATOM 14 H15A POPC 1 0.114 -0.127 20.137 1.00 0.00 MEMB H

ATOM 15 H15B POPC 1 1.688 0.305 20.820 1.00 0.00 MEMB H

ATOM 16 H15C POPC 1 0.342 -0.093 21.898 1.00 0.00 MEMB H

ATOM 17 C11 POPC 1 1.117 -1.645 18.216 1.00 0.00 MEMB C

ATOM 18 H11A POPC 1 1.753 -1.940 17.346 1.00 0.00 MEMB H

ATOM 19 H11B POPC 1 0.986 -0.547 18.163 1.00 0.00 MEMB H

ATOM 20 P POPC 1 -0.562 -3.405 17.176 1.00 0.00 MEMB P

ATOM 21 O13 POPC 1 -0.603 -2.927 15.778 1.00 0.00 MEMB O

ATOM 22 O14 POPC 1 -1.750 -4.039 17.775 1.00 0.00 MEMB O

ATOM 23 O12 POPC 1 -0.167 -2.227 18.153 1.00 0.00 MEMB O

ATOM 24 O11 POPC 1 0.657 -4.408 17.319 1.00 0.00 MEMB O

ATOM 25 C1 POPC 1 0.685 -5.360 18.385 1.00 0.00 MEMB C

ATOM 26 HA POPC 1 -0.291 -5.896 18.482 1.00 0.00 MEMB H

ATOM 27 HB POPC 1 0.886 -4.818 19.331 1.00 0.00 MEMB H

ATOM 28 C2 POPC 1 1.797 -6.445 18.203 1.00 0.00 MEMB C

ATOM 29 HS POPC 1 2.002 -6.913 19.193 1.00 0.00 MEMB H

ATOM 30 O21 POPC 1 2.995 -5.897 17.656 1.00 0.00 MEMB O

ATOM 31 C21 POPC 1 3.740 -5.140 18.396 1.00 0.00 MEMB C

ATOM 32 O22 POPC 1 3.707 -5.062 19.622 1.00 0.00 MEMB O

ATOM 33 C22 POPC 1 4.697 -4.407 17.448 1.00 0.00 MEMB C

ATOM 34 H2R POPC 1 5.553 -5.076 17.221 1.00 0.00 MEMB H

ATOM 35 H2S POPC 1 5.088 -3.503 17.951 1.00 0.00 MEMB H

ATOM 36 C3 POPC 1 1.349 -7.602 17.265 1.00 0.00 MEMB C

ATOM 37 HX POPC 1 0.374 -7.979 17.640 1.00 0.00 MEMB H

ATOM 38 HY POPC 1 2.079 -8.438 17.335 1.00 0.00 MEMB H

ATOM 39 O31 POPC 1 1.127 -7.187 15.917 1.00 0.00 MEMB O

ATOM 40 C31 POPC 1 2.053 -7.342 14.998 1.00 0.00 MEMB C

ATOM 41 O32 POPC 1 3.179 -7.787 15.148 1.00 0.00 MEMB O

ATOM 42 C32 POPC 1 1.461 -6.884 13.662 1.00 0.00 MEMB C

ATOM 43 H2X POPC 1 1.301 -7.779 13.029 1.00 0.00 MEMB H

ATOM 44 H2Y POPC 1 2.205 -6.227 13.171 1.00 0.00 MEMB H

ATOM 45 C23 POPC 1 3.942 -4.039 16.141 1.00 0.00 MEMB C

ATOM 46 H3R POPC 1 2.902 -3.724 16.404 1.00 0.00 MEMB H

ATOM 47 H3S POPC 1 3.855 -4.930 15.479 1.00 0.00 MEMB H

ATOM 48 C24 POPC 1 4.530 -2.881 15.344 1.00 0.00 MEMB C

ATOM 49 H4R POPC 1 5.635 -2.977 15.286 1.00 0.00 MEMB H

ATOM 50 H4S POPC 1 4.284 -1.939 15.887 1.00 0.00 MEMB H

ATOM 51 C25 POPC 1 3.966 -2.756 13.917 1.00 0.00 MEMB C

ATOM 52 H5R POPC 1 4.416 -3.515 13.238 1.00 0.00 MEMB H

ATOM 53 H5S POPC 1 4.293 -1.754 13.568 1.00 0.00 MEMB H

ATOM 54 C26 POPC 1 2.433 -2.842 13.820 1.00 0.00 MEMB C

ATOM 55 H6R POPC 1 1.974 -2.177 14.585 1.00 0.00 MEMB H

ATOM 56 H6S POPC 1 2.091 -3.873 14.067 1.00 0.00 MEMB H

ATOM 57 C27 POPC 1 1.855 -2.470 12.439 1.00 0.00 MEMB C

ATOM 58 H7R POPC 1 0.774 -2.233 12.591 1.00 0.00 MEMB H

ATOM 59 H7S POPC 1 1.904 -3.357 11.772 1.00 0.00 MEMB H

ATOM 60 C28 POPC 1 2.560 -1.299 11.724 1.00 0.00 MEMB C

ATOM 61 H8R POPC 1 3.649 -1.483 11.623 1.00 0.00 MEMB H

ATOM 62 H8S POPC 1 2.473 -0.404 12.381 1.00 0.00 MEMB H

ATOM 63 C29 POPC 1 1.950 -1.038 10.367 1.00 0.00 MEMB C

ATOM 64 H91 POPC 1 1.166 -0.277 10.336 1.00 0.00 MEMB H

ATOM 65 C210 POPC 1 2.166 -1.629 9.196 1.00 0.00 MEMB C

ATOM 66 H101 POPC 1 1.608 -1.218 8.343 1.00 0.00 MEMB H

ATOM 67 C211 POPC 1 3.099 -2.758 8.871 1.00 0.00 MEMB C

ATOM 68 H11R POPC 1 2.499 -3.659 8.610 1.00 0.00 MEMB H

ATOM 69 H11S POPC 1 3.749 -3.040 9.728 1.00 0.00 MEMB H

ATOM 70 C212 POPC 1 3.940 -2.422 7.630 1.00 0.00 MEMB C

ATOM 71 H12R POPC 1 4.688 -3.241 7.533 1.00 0.00 MEMB H

ATOM 72 H12S POPC 1 4.496 -1.467 7.763 1.00 0.00 MEMB H

ATOM 73 C213 POPC 1 3.063 -2.382 6.361 1.00 0.00 MEMB C

ATOM 74 H13R POPC 1 2.353 -1.526 6.418 1.00 0.00 MEMB H

ATOM 75 H13S POPC 1 2.466 -3.315 6.342 1.00 0.00 MEMB H

ATOM 76 C214 POPC 1 3.818 -2.252 5.034 1.00 0.00 MEMB C

ATOM 77 H14R POPC 1 4.646 -1.538 5.204 1.00 0.00 MEMB H

ATOM 78 H14S POPC 1 3.143 -1.785 4.278 1.00 0.00 MEMB H

ATOM 79 C215 POPC 1 4.339 -3.595 4.479 1.00 0.00 MEMB C

ATOM 80 H15R POPC 1 3.504 -4.302 4.310 1.00 0.00 MEMB H

ATOM 81 H15S POPC 1 5.093 -4.034 5.169 1.00 0.00 MEMB H

ATOM 82 C216 POPC 1 4.997 -3.509 3.124 1.00 0.00 MEMB C

ATOM 83 H16R POPC 1 4.285 -3.061 2.381 1.00 0.00 MEMB H

ATOM 84 H16S POPC 1 5.426 -4.451 2.753 1.00 0.00 MEMB H

ATOM 85 C217 POPC 1 6.151 -2.607 3.151 1.00 0.00 MEMB C

ATOM 86 H17R POPC 1 5.760 -1.655 3.557 1.00 0.00 MEMB H

ATOM 87 H17S POPC 1 6.281 -2.481 2.040 1.00 0.00 MEMB H

ATOM 88 C218 POPC 1 7.550 -2.976 3.699 1.00 0.00 MEMB C

ATOM 89 H18R POPC 1 8.203 -2.094 3.925 1.00 0.00 MEMB H

ATOM 90 H18S POPC 1 8.091 -3.493 2.881 1.00 0.00 MEMB H

ATOM 91 H18T POPC 1 7.490 -3.567 4.623 1.00 0.00 MEMB H

ATOM 92 C33 POPC 1 0.120 -6.130 13.814 1.00 0.00 MEMB C

ATOM 93 H3X POPC 1 0.271 -5.176 14.362 1.00 0.00 MEMB H

ATOM 94 H3Y POPC 1 -0.627 -6.729 14.392 1.00 0.00 MEMB H

ATOM 95 C34 POPC 1 -0.488 -5.832 12.457 1.00 0.00 MEMB C

ATOM 96 H4X POPC 1 -0.850 -6.826 12.153 1.00 0.00 MEMB H

ATOM 97 H4Y POPC 1 0.272 -5.537 11.707 1.00 0.00 MEMB H

ATOM 98 C35 POPC 1 -1.623 -4.801 12.519 1.00 0.00 MEMB C

ATOM 99 H5X POPC 1 -1.359 -3.910 13.123 1.00 0.00 MEMB H

ATOM 100 H5Y POPC 1 -2.511 -5.311 12.975 1.00 0.00 MEMB H

ATOM 101 C36 POPC 1 -2.021 -4.310 11.135 1.00 0.00 MEMB C

ATOM 102 H6X POPC 1 -3.049 -3.881 11.192 1.00 0.00 MEMB H

ATOM 103 H6Y POPC 1 -1.982 -5.256 10.578 1.00 0.00 MEMB H

ATOM 104 C37 POPC 1 -1.082 -3.343 10.400 1.00 0.00 MEMB C

ATOM 105 H7X POPC 1 -0.141 -3.867 10.117 1.00 0.00 MEMB H

ATOM 106 H7Y POPC 1 -0.875 -2.575 11.168 1.00 0.00 MEMB H

ATOM 107 C38 POPC 1 -1.664 -2.493 9.237 1.00 0.00 MEMB C

ATOM 108 H8X POPC 1 -0.921 -1.759 8.879 1.00 0.00 MEMB H

ATOM 109 H8Y POPC 1 -2.619 -2.057 9.607 1.00 0.00 MEMB H

ATOM 110 C39 POPC 1 -2.077 -3.213 7.997 1.00 0.00 MEMB C

ATOM 111 H9X POPC 1 -3.031 -2.906 7.506 1.00 0.00 MEMB H

ATOM 112 H9Y POPC 1 -2.281 -4.089 8.565 1.00 0.00 MEMB H

ATOM 113 C310 POPC 1 -1.004 -3.621 7.015 1.00 0.00 MEMB C

ATOM 114 H10X POPC 1 -1.228 -4.627 6.769 1.00 0.00 MEMB H

ATOM 115 H10Y POPC 1 -0.019 -3.828 7.453 1.00 0.00 MEMB H

ATOM 116 C311 POPC 1 -1.073 -2.759 5.759 1.00 0.00 MEMB C

ATOM 117 H11X POPC 1 -0.842 -1.765 6.189 1.00 0.00 MEMB H

ATOM 118 H11Y POPC 1 -2.103 -2.740 5.330 1.00 0.00 MEMB H

ATOM 119 C312 POPC 1 -0.011 -3.066 4.691 1.00 0.00 MEMB C

ATOM 120 H12X POPC 1 0.793 -3.646 5.197 1.00 0.00 MEMB H

ATOM 121 H12Y POPC 1 0.393 -2.106 4.323 1.00 0.00 MEMB H

ATOM 122 C313 POPC 1 -0.457 -3.753 3.394 1.00 0.00 MEMB C

ATOM 123 H13X POPC 1 -0.942 -3.083 2.669 1.00 0.00 MEMB H

ATOM 124 H13Y POPC 1 -1.417 -4.241 3.583 1.00 0.00 MEMB H

ATOM 125 C314 POPC 1 0.711 -4.566 2.741 1.00 0.00 MEMB C

ATOM 126 H14X POPC 1 1.298 -5.321 3.288 1.00 0.00 MEMB H

ATOM 127 H14Y POPC 1 1.520 -3.850 2.530 1.00 0.00 MEMB H

ATOM 128 C315 POPC 1 0.270 -5.360 1.525 1.00 0.00 MEMB C

ATOM 129 H15X POPC 1 -0.162 -4.466 1.088 1.00 0.00 MEMB H

ATOM 130 H15Y POPC 1 -0.645 -5.994 1.461 1.00 0.00 MEMB H

ATOM 131 C316 POPC 1 1.398 -6.177 0.856 1.00 0.00 MEMB C

ATOM 132 H16X POPC 1 2.189 -5.576 0.412 1.00 0.00 MEMB H

ATOM 133 H16Y POPC 1 0.957 -6.903 0.121 1.00 0.00 MEMB H

ATOM 134 H16Z POPC 1 2.000 -6.737 1.572 1.00 0.00 MEMB H

ATOM 135 N POPC 2 -3.929 3.393 18.329 1.00 0.00 MEMB N

ATOM 136 C12 POPC 2 -5.157 3.543 17.432 1.00 0.00 MEMB C

ATOM 137 H12A POPC 2 -5.174 2.718 16.733 1.00 0.00 MEMB H

ATOM 138 H12B POPC 2 -5.031 4.443 16.846 1.00 0.00 MEMB H

ATOM 139 C13 POPC 2 -3.645 4.668 19.064 1.00 0.00 MEMB C

ATOM 140 H13A POPC 2 -2.759 4.579 19.694 1.00 0.00 MEMB H

ATOM 141 H13B POPC 2 -3.413 5.486 18.381 1.00 0.00 MEMB H

ATOM 142 H13C POPC 2 -4.529 4.973 19.626 1.00 0.00 MEMB H

ATOM 143 C14 POPC 2 -4.133 2.260 19.283 1.00 0.00 MEMB C

ATOM 144 H14A POPC 2 -4.941 2.473 19.982 1.00 0.00 MEMB H

ATOM 145 H14B POPC 2 -4.458 1.357 18.774 1.00 0.00 MEMB H

ATOM 146 H14C POPC 2 -3.233 2.013 19.827 1.00 0.00 MEMB H

ATOM 147 C15 POPC 2 -2.743 3.128 17.481 1.00 0.00 MEMB C

ATOM 148 H15A POPC 2 -2.860 2.223 16.896 1.00 0.00 MEMB H

ATOM 149 H15B POPC 2 -1.850 3.043 18.091 1.00 0.00 MEMB H

ATOM 150 H15C POPC 2 -2.571 3.982 16.827 1.00 0.00 MEMB H

ATOM 151 C11 POPC 2 -6.559 3.604 18.081 1.00 0.00 MEMB C

ATOM 152 H11A POPC 2 -6.824 2.597 18.473 1.00 0.00 MEMB H

ATOM 153 H11B POPC 2 -7.319 3.842 17.297 1.00 0.00 MEMB H

ATOM 154 P POPC 2 -7.993 4.766 19.848 1.00 0.00 MEMB P

ATOM 155 O13 POPC 2 -7.796 5.454 21.141 1.00 0.00 MEMB O

ATOM 156 O14 POPC 2 -8.775 3.515 19.834 1.00 0.00 MEMB O

ATOM 157 O12 POPC 2 -6.591 4.567 19.129 1.00 0.00 MEMB O

ATOM 158 O11 POPC 2 -8.703 5.759 18.821 1.00 0.00 MEMB O

ATOM 159 C1 POPC 2 -8.065 6.974 18.471 1.00 0.00 MEMB C

ATOM 160 HA POPC 2 -6.956 6.871 18.362 1.00 0.00 MEMB H

ATOM 161 HB POPC 2 -8.276 7.670 19.284 1.00 0.00 MEMB H

ATOM 162 C2 POPC 2 -8.650 7.515 17.149 1.00 0.00 MEMB C

ATOM 163 HS POPC 2 -9.706 7.803 17.366 1.00 0.00 MEMB H

ATOM 164 O21 POPC 2 -8.593 6.439 16.188 1.00 0.00 MEMB O

ATOM 165 C21 POPC 2 -9.258 6.660 15.082 1.00 0.00 MEMB C

ATOM 166 O22 POPC 2 -9.990 7.618 14.889 1.00 0.00 MEMB O

ATOM 167 C22 POPC 2 -8.908 5.606 14.032 1.00 0.00 MEMB C

ATOM 168 H2R POPC 2 -9.199 4.606 14.410 1.00 0.00 MEMB H

ATOM 169 H2S POPC 2 -7.811 5.618 13.862 1.00 0.00 MEMB H

ATOM 170 C3 POPC 2 -7.894 8.771 16.625 1.00 0.00 MEMB C

ATOM 171 HX POPC 2 -7.810 9.565 17.397 1.00 0.00 MEMB H

ATOM 172 HY POPC 2 -8.493 9.194 15.782 1.00 0.00 MEMB H

ATOM 173 O31 POPC 2 -6.580 8.364 16.207 1.00 0.00 MEMB O

ATOM 174 C31 POPC 2 -6.047 9.177 15.308 1.00 0.00 MEMB C

ATOM 175 O32 POPC 2 -6.465 10.274 15.022 1.00 0.00 MEMB O

ATOM 176 C32 POPC 2 -4.809 8.545 14.732 1.00 0.00 MEMB C

ATOM 177 H2X POPC 2 -4.062 8.487 15.552 1.00 0.00 MEMB H

ATOM 178 H2Y POPC 2 -4.402 9.220 13.948 1.00 0.00 MEMB H

ATOM 179 C23 POPC 2 -9.655 5.973 12.727 1.00 0.00 MEMB C

ATOM 180 H3R POPC 2 -9.523 7.066 12.529 1.00 0.00 MEMB H

ATOM 181 H3S POPC 2 -10.743 5.801 12.883 1.00 0.00 MEMB H

ATOM 182 C24 POPC 2 -9.163 5.225 11.482 1.00 0.00 MEMB C

ATOM 183 H4R POPC 2 -9.266 4.132 11.621 1.00 0.00 MEMB H

ATOM 184 H4S POPC 2 -8.089 5.479 11.319 1.00 0.00 MEMB H

ATOM 185 C25 POPC 2 -9.886 5.609 10.197 1.00 0.00 MEMB C

ATOM 186 H5R POPC 2 -10.988 5.482 10.291 1.00 0.00 MEMB H

ATOM 187 H5S POPC 2 -9.535 4.953 9.363 1.00 0.00 MEMB H

ATOM 188 C26 POPC 2 -9.495 7.012 9.791 1.00 0.00 MEMB C

ATOM 189 H6R POPC 2 -8.413 7.206 9.939 1.00 0.00 MEMB H

ATOM 190 H6S POPC 2 -10.012 7.771 10.391 1.00 0.00 MEMB H

ATOM 191 C27 POPC 2 -9.683 7.192 8.326 1.00 0.00 MEMB C

ATOM 192 H7R POPC 2 -10.762 7.106 8.078 1.00 0.00 MEMB H

ATOM 193 H7S POPC 2 -9.200 6.358 7.766 1.00 0.00 MEMB H

ATOM 194 C28 POPC 2 -8.999 8.469 7.939 1.00 0.00 MEMB C

ATOM 195 H8R POPC 2 -7.932 8.554 8.119 1.00 0.00 MEMB H

ATOM 196 H8S POPC 2 -9.230 9.409 8.326 1.00 0.00 MEMB H

ATOM 197 C29 POPC 2 -9.528 8.471 6.613 1.00 0.00 MEMB C

ATOM 198 H91 POPC 2 -10.191 9.235 6.191 1.00 0.00 MEMB H

ATOM 199 C210 POPC 2 -8.933 7.986 5.686 1.00 0.00 MEMB C

ATOM 200 H101 POPC 2 -9.349 8.538 4.878 1.00 0.00 MEMB H

ATOM 201 C211 POPC 2 -7.654 7.355 5.645 1.00 0.00 MEMB C

ATOM 202 H11R POPC 2 -7.487 7.303 4.551 1.00 0.00 MEMB H

ATOM 203 H11S POPC 2 -7.931 6.390 6.070 1.00 0.00 MEMB H

ATOM 204 C212 POPC 2 -6.488 8.072 6.392 1.00 0.00 MEMB C

ATOM 205 H12R POPC 2 -6.262 7.565 7.331 1.00 0.00 MEMB H

ATOM 206 H12S POPC 2 -6.628 9.063 6.754 1.00 0.00 MEMB H

ATOM 207 C213 POPC 2 -5.271 8.340 5.583 1.00 0.00 MEMB C

ATOM 208 H13R POPC 2 -5.417 7.398 5.012 1.00 0.00 MEMB H

ATOM 209 H13S POPC 2 -4.311 8.325 6.111 1.00 0.00 MEMB H

ATOM 210 C214 POPC 2 -5.151 9.772 5.057 1.00 0.00 MEMB C

ATOM 211 H14R POPC 2 -5.067 10.621 5.759 1.00 0.00 MEMB H

ATOM 212 H14S POPC 2 -5.907 10.012 4.404 1.00 0.00 MEMB H

ATOM 213 C215 POPC 2 -4.363 10.077 3.902 1.00 0.00 MEMB C

ATOM 214 H15R POPC 2 -3.527 10.255 4.566 1.00 0.00 MEMB H

ATOM 215 H15S POPC 2 -4.448 11.077 3.453 1.00 0.00 MEMB H

ATOM 216 C216 POPC 2 -4.492 8.797 3.070 1.00 0.00 MEMB C

ATOM 217 H16R POPC 2 -4.311 7.883 3.595 1.00 0.00 MEMB H

ATOM 218 H16S POPC 2 -3.623 9.373 2.729 1.00 0.00 MEMB H

ATOM 219 C217 POPC 2 -5.001 8.323 1.695 1.00 0.00 MEMB C

ATOM 220 H17R POPC 2 -4.963 9.349 1.443 1.00 0.00 MEMB H

ATOM 221 H17S POPC 2 -6.047 7.999 1.626 1.00 0.00 MEMB H

ATOM 222 C218 POPC 2 -4.304 7.577 0.520 1.00 0.00 MEMB C

ATOM 223 H18R POPC 2 -3.612 8.160 -0.122 1.00 0.00 MEMB H

ATOM 224 H18S POPC 2 -5.202 7.326 -0.054 1.00 0.00 MEMB H

ATOM 225 H18T POPC 2 -4.043 6.523 0.499 1.00 0.00 MEMB H

ATOM 226 C33 POPC 2 -5.138 7.133 14.194 1.00 0.00 MEMB C

ATOM 227 H3X POPC 2 -5.894 7.165 13.378 1.00 0.00 MEMB H

ATOM 228 H3Y POPC 2 -5.575 6.528 15.022 1.00 0.00 MEMB H

ATOM 229 C34 POPC 2 -3.862 6.453 13.704 1.00 0.00 MEMB C

ATOM 230 H4X POPC 2 -3.241 6.167 14.581 1.00 0.00 MEMB H

ATOM 231 H4Y POPC 2 -3.280 7.228 13.149 1.00 0.00 MEMB H

ATOM 232 C35 POPC 2 -4.037 5.257 12.748 1.00 0.00 MEMB C

ATOM 233 H5X POPC 2 -3.018 5.082 12.322 1.00 0.00 MEMB H

ATOM 234 H5Y POPC 2 -4.710 5.494 11.897 1.00 0.00 MEMB H

ATOM 235 C36 POPC 2 -4.450 3.908 13.357 1.00 0.00 MEMB C

ATOM 236 H6X POPC 2 -5.550 3.863 13.514 1.00 0.00 MEMB H

ATOM 237 H6Y POPC 2 -3.954 3.772 14.344 1.00 0.00 MEMB H

ATOM 238 C37 POPC 2 -3.991 2.751 12.445 1.00 0.00 MEMB C

ATOM 239 H7X POPC 2 -3.282 2.123 13.032 1.00 0.00 MEMB H

ATOM 240 H7Y POPC 2 -3.413 3.145 11.579 1.00 0.00 MEMB H

ATOM 241 C38 POPC 2 -5.132 1.867 11.916 1.00 0.00 MEMB C

ATOM 242 H8X POPC 2 -5.759 1.581 12.790 1.00 0.00 MEMB H

ATOM 243 H8Y POPC 2 -4.697 0.932 11.497 1.00 0.00 MEMB H

ATOM 244 C39 POPC 2 -6.107 2.559 10.939 1.00 0.00 MEMB C

ATOM 245 H9X POPC 2 -6.424 3.507 11.421 1.00 0.00 MEMB H

ATOM 246 H9Y POPC 2 -7.008 1.912 10.836 1.00 0.00 MEMB H

ATOM 247 C310 POPC 2 -5.613 2.905 9.524 1.00 0.00 MEMB C

ATOM 248 H10X POPC 2 -5.415 1.969 8.957 1.00 0.00 MEMB H

ATOM 249 H10Y POPC 2 -4.680 3.508 9.589 1.00 0.00 MEMB H

ATOM 250 C311 POPC 2 -6.650 3.726 8.743 1.00 0.00 MEMB C

ATOM 251 H11X POPC 2 -6.851 4.669 9.307 1.00 0.00 MEMB H

ATOM 252 H11Y POPC 2 -7.612 3.171 8.685 1.00 0.00 MEMB H

ATOM 253 C312 POPC 2 -6.214 4.109 7.320 1.00 0.00 MEMB C

ATOM 254 H12X POPC 2 -5.404 4.871 7.374 1.00 0.00 MEMB H

ATOM 255 H12Y POPC 2 -7.106 4.579 6.853 1.00 0.00 MEMB H

ATOM 256 C313 POPC 2 -5.778 2.975 6.375 1.00 0.00 MEMB C

ATOM 257 H13X POPC 2 -6.633 2.280 6.234 1.00 0.00 MEMB H

ATOM 258 H13Y POPC 2 -4.941 2.376 6.801 1.00 0.00 MEMB H

ATOM 259 C314 POPC 2 -5.340 3.537 5.008 1.00 0.00 MEMB C

ATOM 260 H14X POPC 2 -5.022 2.707 4.342 1.00 0.00 MEMB H

ATOM 261 H14Y POPC 2 -4.449 4.188 5.181 1.00 0.00 MEMB H

ATOM 262 C315 POPC 2 -6.409 4.398 4.309 1.00 0.00 MEMB C

ATOM 263 H15X POPC 2 -6.666 5.222 4.999 1.00 0.00 MEMB H

ATOM 264 H15Y POPC 2 -7.335 3.832 4.090 1.00 0.00 MEMB H

ATOM 265 C316 POPC 2 -5.892 5.099 3.065 1.00 0.00 MEMB C

ATOM 266 H16X POPC 2 -5.750 4.419 2.200 1.00 0.00 MEMB H

ATOM 267 H16Y POPC 2 -4.914 5.506 3.370 1.00 0.00 MEMB H

ATOM 268 H16Z POPC 2 -6.545 5.950 2.771 1.00 0.00 MEMB H

ATOM 269 N POPC 3 3.046 8.763 20.368 1.00 0.00 MEMB N

ATOM 270 C12 POPC 3 3.105 7.311 19.876 1.00 0.00 MEMB C

ATOM 271 H12A POPC 3 3.476 6.706 20.694 1.00 0.00 MEMB H

ATOM 272 H12B POPC 3 3.819 7.263 19.056 1.00 0.00 MEMB H

ATOM 273 C13 POPC 3 2.676 9.653 19.212 1.00 0.00 MEMB C

ATOM 274 H13A POPC 3 3.393 9.584 18.402 1.00 0.00 MEMB H

ATOM 275 H13B POPC 3 1.738 9.285 18.776 1.00 0.00 MEMB H

ATOM 276 H13C POPC 3 2.527 10.684 19.485 1.00 0.00 MEMB H

ATOM 277 C14 POPC 3 1.980 8.885 21.420 1.00 0.00 MEMB C

ATOM 278 H14A POPC 3 1.022 8.638 20.949 1.00 0.00 MEMB H

ATOM 279 H14B POPC 3 2.140 8.214 22.254 1.00 0.00 MEMB H

ATOM 280 H14C POPC 3 1.906 9.880 21.823 1.00 0.00 MEMB H

ATOM 281 C15 POPC 3 4.369 9.166 20.913 1.00 0.00 MEMB C

ATOM 282 H15A POPC 3 4.334 10.176 21.296 1.00 0.00 MEMB H

ATOM 283 H15B POPC 3 5.130 9.143 20.135 1.00 0.00 MEMB H

ATOM 284 H15C POPC 3 4.685 8.494 21.700 1.00 0.00 MEMB H

ATOM 285 C11 POPC 3 1.799 6.599 19.396 1.00 0.00 MEMB C

ATOM 286 H11A POPC 3 1.117 6.464 20.265 1.00 0.00 MEMB H

ATOM 287 H11B POPC 3 2.071 5.577 19.038 1.00 0.00 MEMB H

ATOM 288 P POPC 3 -0.022 6.750 17.451 1.00 0.00 MEMB P

ATOM 289 O13 POPC 3 -0.249 7.731 16.368 1.00 0.00 MEMB O

ATOM 290 O14 POPC 3 -1.184 6.351 18.274 1.00 0.00 MEMB O

ATOM 291 O12 POPC 3 1.152 7.338 18.369 1.00 0.00 MEMB O

ATOM 292 O11 POPC 3 0.589 5.441 16.785 1.00 0.00 MEMB O

ATOM 293 C1 POPC 3 1.891 5.537 16.207 1.00 0.00 MEMB C

ATOM 294 HA POPC 3 2.585 4.977 16.869 1.00 0.00 MEMB H

ATOM 295 HB POPC 3 2.279 6.583 16.119 1.00 0.00 MEMB H

ATOM 296 C2 POPC 3 1.991 4.936 14.780 1.00 0.00 MEMB C

ATOM 297 HS POPC 3 3.061 5.005 14.506 1.00 0.00 MEMB H

ATOM 298 O21 POPC 3 1.171 5.627 13.814 1.00 0.00 MEMB O

ATOM 299 C21 POPC 3 1.740 6.749 13.360 1.00 0.00 MEMB C

ATOM 300 O22 POPC 3 2.915 7.040 13.501 1.00 0.00 MEMB O

ATOM 301 C22 POPC 3 0.667 7.588 12.649 1.00 0.00 MEMB C

ATOM 302 H2R POPC 3 -0.003 7.950 13.459 1.00 0.00 MEMB H

ATOM 303 H2S POPC 3 0.067 6.931 11.987 1.00 0.00 MEMB H

ATOM 304 C3 POPC 3 1.625 3.438 14.776 1.00 0.00 MEMB C

ATOM 305 HX POPC 3 0.563 3.252 15.045 1.00 0.00 MEMB H

ATOM 306 HY POPC 3 2.262 2.950 15.546 1.00 0.00 MEMB H

ATOM 307 O31 POPC 3 2.015 2.893 13.519 1.00 0.00 MEMB O

ATOM 308 C31 POPC 3 1.072 2.666 12.638 1.00 0.00 MEMB C

ATOM 309 O32 POPC 3 -0.131 2.717 12.813 1.00 0.00 MEMB O

ATOM 310 C32 POPC 3 1.745 2.179 11.370 1.00 0.00 MEMB C

ATOM 311 H2X POPC 3 1.595 1.115 11.420 1.00 0.00 MEMB H

ATOM 312 H2Y POPC 3 2.834 2.350 11.400 1.00 0.00 MEMB H

ATOM 313 C23 POPC 3 1.180 8.829 11.860 1.00 0.00 MEMB C

ATOM 314 H3R POPC 3 1.849 8.523 11.032 1.00 0.00 MEMB H

ATOM 315 H3S POPC 3 1.779 9.486 12.535 1.00 0.00 MEMB H

ATOM 316 C24 POPC 3 0.004 9.636 11.277 1.00 0.00 MEMB C

ATOM 317 H4R POPC 3 -0.506 10.127 12.142 1.00 0.00 MEMB H

ATOM 318 H4S POPC 3 -0.737 8.960 10.804 1.00 0.00 MEMB H

ATOM 319 C25 POPC 3 0.383 10.751 10.284 1.00 0.00 MEMB C

ATOM 320 H5R POPC 3 1.201 11.348 10.746 1.00 0.00 MEMB H

ATOM 321 H5S POPC 3 -0.519 11.399 10.192 1.00 0.00 MEMB H

ATOM 322 C26 POPC 3 0.810 10.372 8.848 1.00 0.00 MEMB C

ATOM 323 H6R POPC 3 1.717 9.733 8.866 1.00 0.00 MEMB H

ATOM 324 H6S POPC 3 1.110 11.336 8.367 1.00 0.00 MEMB H

ATOM 325 C27 POPC 3 -0.293 9.666 8.020 1.00 0.00 MEMB C

ATOM 326 H7R POPC 3 -1.127 10.393 7.839 1.00 0.00 MEMB H

ATOM 327 H7S POPC 3 -0.709 8.847 8.631 1.00 0.00 MEMB H

ATOM 328 C28 POPC 3 0.178 9.007 6.714 1.00 0.00 MEMB C

ATOM 329 H8R POPC 3 -0.517 8.267 6.280 1.00 0.00 MEMB H

ATOM 330 H8S POPC 3 1.193 8.568 6.790 1.00 0.00 MEMB H

ATOM 331 C29 POPC 3 0.292 10.203 5.892 1.00 0.00 MEMB C

ATOM 332 H91 POPC 3 1.215 10.656 6.198 1.00 0.00 MEMB H

ATOM 333 C210 POPC 3 -0.343 10.591 4.794 1.00 0.00 MEMB C

ATOM 334 H101 POPC 3 -0.049 11.425 4.129 1.00 0.00 MEMB H

ATOM 335 C211 POPC 3 -1.321 9.819 4.170 1.00 0.00 MEMB C

ATOM 336 H11R POPC 3 -1.931 10.677 3.818 1.00 0.00 MEMB H

ATOM 337 H11S POPC 3 -1.907 9.279 4.957 1.00 0.00 MEMB H

ATOM 338 C212 POPC 3 -0.808 8.893 3.038 1.00 0.00 MEMB C

ATOM 339 H12R POPC 3 0.031 8.221 2.823 1.00 0.00 MEMB H

ATOM 340 H12S POPC 3 -0.498 9.488 2.206 1.00 0.00 MEMB H

ATOM 341 C213 POPC 3 -2.024 8.029 3.083 1.00 0.00 MEMB C

ATOM 342 H13R POPC 3 -2.108 8.300 1.976 1.00 0.00 MEMB H

ATOM 343 H13S POPC 3 -1.328 7.626 3.880 1.00 0.00 MEMB H

ATOM 344 C214 POPC 3 -2.527 6.663 2.787 1.00 0.00 MEMB C

ATOM 345 H14R POPC 3 -3.586 6.525 2.489 1.00 0.00 MEMB H

ATOM 346 H14S POPC 3 -2.596 6.211 3.792 1.00 0.00 MEMB H

ATOM 347 C215 POPC 3 -1.865 6.093 1.617 1.00 0.00 MEMB C

ATOM 348 H15R POPC 3 -0.806 6.332 1.795 1.00 0.00 MEMB H

ATOM 349 H15S POPC 3 -2.106 6.547 0.622 1.00 0.00 MEMB H

ATOM 350 C216 POPC 3 -2.390 4.701 1.743 1.00 0.00 MEMB C

ATOM 351 H16R POPC 3 -3.313 4.592 1.124 1.00 0.00 MEMB H

ATOM 352 H16S POPC 3 -2.723 4.504 2.796 1.00 0.00 MEMB H

ATOM 353 C217 POPC 3 -1.255 3.764 1.516 1.00 0.00 MEMB C

ATOM 354 H17R POPC 3 -1.744 2.774 1.375 1.00 0.00 MEMB H

ATOM 355 H17S POPC 3 -0.620 3.725 2.422 1.00 0.00 MEMB H

ATOM 356 C218 POPC 3 -0.354 4.217 0.377 1.00 0.00 MEMB C

ATOM 357 H18R POPC 3 -1.013 4.540 -0.413 1.00 0.00 MEMB H

ATOM 358 H18S POPC 3 0.283 3.383 0.018 1.00 0.00 MEMB H

ATOM 359 H18T POPC 3 0.172 5.182 0.419 1.00 0.00 MEMB H

ATOM 360 C33 POPC 3 1.137 2.787 10.109 1.00 0.00 MEMB C

ATOM 361 H3X POPC 3 0.034 2.892 10.207 1.00 0.00 MEMB H

ATOM 362 H3Y POPC 3 1.333 2.161 9.206 1.00 0.00 MEMB H

ATOM 363 C34 POPC 3 1.854 4.116 9.936 1.00 0.00 MEMB C

ATOM 364 H4X POPC 3 2.918 3.867 9.759 1.00 0.00 MEMB H

ATOM 365 H4Y POPC 3 1.817 4.687 10.891 1.00 0.00 MEMB H

ATOM 366 C35 POPC 3 1.352 4.932 8.760 1.00 0.00 MEMB C

ATOM 367 H5X POPC 3 0.268 5.149 8.892 1.00 0.00 MEMB H

ATOM 368 H5Y POPC 3 1.451 4.328 7.832 1.00 0.00 MEMB H

ATOM 369 C36 POPC 3 2.092 6.269 8.617 1.00 0.00 MEMB C

ATOM 370 H6X POPC 3 1.993 6.845 9.564 1.00 0.00 MEMB H

ATOM 371 H6Y POPC 3 1.575 6.821 7.808 1.00 0.00 MEMB H

ATOM 372 C37 POPC 3 3.592 6.218 8.288 1.00 0.00 MEMB C

ATOM 373 H7X POPC 3 4.146 5.939 9.211 1.00 0.00 MEMB H

ATOM 374 H7Y POPC 3 3.926 7.247 8.042 1.00 0.00 MEMB H

ATOM 375 C38 POPC 3 3.979 5.281 7.135 1.00 0.00 MEMB C

ATOM 376 H8X POPC 3 3.648 4.252 7.399 1.00 0.00 MEMB H

ATOM 377 H8Y POPC 3 5.085 5.271 7.047 1.00 0.00 MEMB H

ATOM 378 C39 POPC 3 3.304 5.551 5.785 1.00 0.00 MEMB C

ATOM 379 H9X POPC 3 2.223 5.711 5.971 1.00 0.00 MEMB H

ATOM 380 H9Y POPC 3 3.383 4.621 5.187 1.00 0.00 MEMB H

ATOM 381 C310 POPC 3 3.874 6.719 4.975 1.00 0.00 MEMB C

ATOM 382 H10X POPC 3 4.925 6.500 4.682 1.00 0.00 MEMB H

ATOM 383 H10Y POPC 3 3.877 7.637 5.603 1.00 0.00 MEMB H

ATOM 384 C311 POPC 3 3.059 7.012 3.707 1.00 0.00 MEMB C

ATOM 385 H11X POPC 3 2.015 7.266 3.996 1.00 0.00 MEMB H

ATOM 386 H11Y POPC 3 2.988 6.102 3.083 1.00 0.00 MEMB H

ATOM 387 C312 POPC 3 3.711 8.127 2.880 1.00 0.00 MEMB C

ATOM 388 H12X POPC 3 4.752 7.831 2.622 1.00 0.00 MEMB H

ATOM 389 H12Y POPC 3 3.797 9.043 3.490 1.00 0.00 MEMB H

ATOM 390 C313 POPC 3 2.993 8.478 1.581 1.00 0.00 MEMB C

ATOM 391 H13X POPC 3 3.560 9.312 1.101 1.00 0.00 MEMB H

ATOM 392 H13Y POPC 3 1.955 8.831 1.756 1.00 0.00 MEMB H

ATOM 393 C314 POPC 3 2.972 7.340 0.586 1.00 0.00 MEMB C

ATOM 394 H14X POPC 3 2.327 6.485 0.884 1.00 0.00 MEMB H

ATOM 395 H14Y POPC 3 4.011 6.956 0.432 1.00 0.00 MEMB H

ATOM 396 C315 POPC 3 2.471 7.840 -0.745 1.00 0.00 MEMB C

ATOM 397 H15X POPC 3 2.694 6.929 -1.349 1.00 0.00 MEMB H

ATOM 398 H15Y POPC 3 3.138 8.631 -1.145 1.00 0.00 MEMB H

ATOM 399 C316 POPC 3 0.991 8.316 -0.824 1.00 0.00 MEMB C

ATOM 400 H16X POPC 3 0.279 7.475 -0.667 1.00 0.00 MEMB H

ATOM 401 H16Y POPC 3 0.772 8.663 -1.856 1.00 0.00 MEMB H

ATOM 402 H16Z POPC 3 0.743 9.176 -0.185 1.00 0.00 MEMB H

ATOM 403 N POPC 4 6.440 2.744 20.639 1.00 0.00 MEMB N

ATOM 404 C12 POPC 4 5.920 2.132 19.350 1.00 0.00 MEMB C

ATOM 405 H12A POPC 4 5.633 2.917 18.662 1.00 0.00 MEMB H

ATOM 406 H12B POPC 4 5.074 1.515 19.589 1.00 0.00 MEMB H

ATOM 407 C13 POPC 4 7.631 3.619 20.363 1.00 0.00 MEMB C

ATOM 408 H13A POPC 4 8.470 3.054 19.941 1.00 0.00 MEMB H

ATOM 409 H13B POPC 4 7.403 4.329 19.574 1.00 0.00 MEMB H

ATOM 410 H13C POPC 4 7.995 4.142 21.232 1.00 0.00 MEMB H

ATOM 411 C14 POPC 4 5.345 3.525 21.238 1.00 0.00 MEMB C

ATOM 412 H14A POPC 4 5.668 3.911 22.207 1.00 0.00 MEMB H

ATOM 413 H14B POPC 4 5.097 4.403 20.656 1.00 0.00 MEMB H

ATOM 414 H14C POPC 4 4.490 2.895 21.391 1.00 0.00 MEMB H

ATOM 415 C15 POPC 4 6.762 1.698 21.675 1.00 0.00 MEMB C

ATOM 416 H15A POPC 4 5.927 1.032 21.809 1.00 0.00 MEMB H

ATOM 417 H15B POPC 4 6.966 2.159 22.636 1.00 0.00 MEMB H

ATOM 418 H15C POPC 4 7.640 1.127 21.386 1.00 0.00 MEMB H

ATOM 419 C11 POPC 4 6.846 1.271 18.510 1.00 0.00 MEMB C

ATOM 420 H11A POPC 4 6.239 0.626 17.833 1.00 0.00 MEMB H

ATOM 421 H11B POPC 4 7.349 0.577 19.242 1.00 0.00 MEMB H

ATOM 422 P POPC 4 9.056 1.418 17.379 1.00 0.00 MEMB P

ATOM 423 O13 POPC 4 8.842 0.382 16.330 1.00 0.00 MEMB O

ATOM 424 O14 POPC 4 9.504 1.085 18.742 1.00 0.00 MEMB O

ATOM 425 O12 POPC 4 7.693 2.145 17.710 1.00 0.00 MEMB O

ATOM 426 O11 POPC 4 10.184 2.404 16.902 1.00 0.00 MEMB O

ATOM 427 C1 POPC 4 10.595 2.556 15.558 1.00 0.00 MEMB C

ATOM 428 HA POPC 4 10.520 1.623 14.943 1.00 0.00 MEMB H

ATOM 429 HB POPC 4 11.667 2.858 15.557 1.00 0.00 MEMB H

ATOM 430 C2 POPC 4 9.731 3.683 14.976 1.00 0.00 MEMB C

ATOM 431 HS POPC 4 9.770 4.504 15.732 1.00 0.00 MEMB H

ATOM 432 O21 POPC 4 8.390 3.178 14.822 1.00 0.00 MEMB O

ATOM 433 C21 POPC 4 7.436 4.061 15.024 1.00 0.00 MEMB C

ATOM 434 O22 POPC 4 7.604 5.266 15.138 1.00 0.00 MEMB O

ATOM 435 C22 POPC 4 6.084 3.357 15.119 1.00 0.00 MEMB C

ATOM 436 H2R POPC 4 5.634 3.671 16.085 1.00 0.00 MEMB H

ATOM 437 H2S POPC 4 6.173 2.251 15.134 1.00 0.00 MEMB H

ATOM 438 C3 POPC 4 10.281 4.183 13.634 1.00 0.00 MEMB C

ATOM 439 HX POPC 4 11.313 4.593 13.768 1.00 0.00 MEMB H

ATOM 440 HY POPC 4 9.620 5.003 13.275 1.00 0.00 MEMB H

ATOM 441 O31 POPC 4 10.274 3.062 12.737 1.00 0.00 MEMB O

ATOM 442 C31 POPC 4 10.692 3.338 11.538 1.00 0.00 MEMB C

ATOM 443 O32 POPC 4 11.102 4.418 11.155 1.00 0.00 MEMB O

ATOM 444 C32 POPC 4 10.503 2.119 10.646 1.00 0.00 MEMB C

ATOM 445 H2X POPC 4 9.460 1.758 10.754 1.00 0.00 MEMB H

ATOM 446 H2Y POPC 4 11.187 1.323 11.010 1.00 0.00 MEMB H

ATOM 447 C23 POPC 4 5.165 3.802 13.976 1.00 0.00 MEMB C

ATOM 448 H3R POPC 4 5.123 4.916 13.937 1.00 0.00 MEMB H

ATOM 449 H3S POPC 4 4.170 3.415 14.263 1.00 0.00 MEMB H

ATOM 450 C24 POPC 4 5.497 3.205 12.606 1.00 0.00 MEMB C

ATOM 451 H4R POPC 4 4.576 2.863 12.099 1.00 0.00 MEMB H

ATOM 452 H4S POPC 4 6.137 2.307 12.748 1.00 0.00 MEMB H

ATOM 453 C25 POPC 4 6.159 4.218 11.672 1.00 0.00 MEMB C

ATOM 454 H5R POPC 4 6.994 4.698 12.224 1.00 0.00 MEMB H

ATOM 455 H5S POPC 4 5.431 5.016 11.395 1.00 0.00 MEMB H

ATOM 456 C26 POPC 4 6.678 3.580 10.377 1.00 0.00 MEMB C

ATOM 457 H6R POPC 4 7.262 2.676 10.645 1.00 0.00 MEMB H

ATOM 458 H6S POPC 4 7.354 4.318 9.894 1.00 0.00 MEMB H

ATOM 459 C27 POPC 4 5.524 3.234 9.417 1.00 0.00 MEMB C

ATOM 460 H7R POPC 4 4.860 4.113 9.428 1.00 0.00 MEMB H

ATOM 461 H7S POPC 4 4.927 2.393 9.835 1.00 0.00 MEMB H

ATOM 462 C28 POPC 4 5.912 2.902 7.960 1.00 0.00 MEMB C

ATOM 463 H8R POPC 4 6.521 3.710 7.497 1.00 0.00 MEMB H

ATOM 464 H8S POPC 4 4.990 2.799 7.342 1.00 0.00 MEMB H

ATOM 465 C29 POPC 4 6.621 1.587 7.985 1.00 0.00 MEMB C

ATOM 466 H91 POPC 4 7.055 1.343 8.960 1.00 0.00 MEMB H

ATOM 467 C210 POPC 4 6.710 0.706 6.981 1.00 0.00 MEMB C

ATOM 468 H101 POPC 4 7.170 -0.274 7.187 1.00 0.00 MEMB H

ATOM 469 C211 POPC 4 6.100 0.905 5.619 1.00 0.00 MEMB C

ATOM 470 H11R POPC 4 5.903 1.973 5.399 1.00 0.00 MEMB H

ATOM 471 H11S POPC 4 5.119 0.383 5.568 1.00 0.00 MEMB H

ATOM 472 C212 POPC 4 6.961 0.423 4.457 1.00 0.00 MEMB C

ATOM 473 H12R POPC 4 7.094 -0.656 4.616 1.00 0.00 MEMB H

ATOM 474 H12S POPC 4 7.988 0.839 4.443 1.00 0.00 MEMB H

ATOM 475 C213 POPC 4 6.258 0.691 3.122 1.00 0.00 MEMB C

ATOM 476 H13R POPC 4 6.470 1.723 2.782 1.00 0.00 MEMB H

ATOM 477 H13S POPC 4 5.168 0.472 3.175 1.00 0.00 MEMB H

ATOM 478 C214 POPC 4 6.847 -0.117 2.035 1.00 0.00 MEMB C

ATOM 479 H14R POPC 4 6.175 -0.388 1.182 1.00 0.00 MEMB H

ATOM 480 H14S POPC 4 7.329 -0.994 2.402 1.00 0.00 MEMB H

ATOM 481 C215 POPC 4 8.233 0.290 1.819 1.00 0.00 MEMB C

ATOM 482 H15R POPC 4 8.254 1.365 1.608 1.00 0.00 MEMB H

ATOM 483 H15S POPC 4 8.234 -0.343 0.927 1.00 0.00 MEMB H

ATOM 484 C216 POPC 4 9.500 -0.516 2.013 1.00 0.00 MEMB C

ATOM 485 H16R POPC 4 9.368 -1.620 2.045 1.00 0.00 MEMB H

ATOM 486 H16S POPC 4 9.902 -0.126 2.970 1.00 0.00 MEMB H

ATOM 487 C217 POPC 4 10.364 -0.141 0.800 1.00 0.00 MEMB C

ATOM 488 H17R POPC 4 10.008 -0.458 -0.183 1.00 0.00 MEMB H

ATOM 489 H17S POPC 4 11.239 -0.748 0.581 1.00 0.00 MEMB H

ATOM 490 C218 POPC 4 10.572 1.362 0.994 1.00 0.00 MEMB C

ATOM 491 H18R POPC 4 11.455 1.704 0.614 1.00 0.00 MEMB H

ATOM 492 H18S POPC 4 10.729 1.708 2.027 1.00 0.00 MEMB H

ATOM 493 H18T POPC 4 10.046 2.056 0.355 1.00 0.00 MEMB H

ATOM 494 C33 POPC 4 10.761 2.560 9.189 1.00 0.00 MEMB C

ATOM 495 H3X POPC 4 11.807 2.947 9.102 1.00 0.00 MEMB H

ATOM 496 H3Y POPC 4 10.066 3.387 8.931 1.00 0.00 MEMB H

ATOM 497 C34 POPC 4 10.557 1.475 8.140 1.00 0.00 MEMB C

ATOM 498 H4X POPC 4 9.502 1.182 7.971 1.00 0.00 MEMB H

ATOM 499 H4Y POPC 4 11.198 0.646 8.455 1.00 0.00 MEMB H

ATOM 500 C35 POPC 4 11.231 1.758 6.835 1.00 0.00 MEMB C

ATOM 501 H5X POPC 4 11.254 0.962 6.077 1.00 0.00 MEMB H

ATOM 502 H5Y POPC 4 12.299 1.804 7.097 1.00 0.00 MEMB H

ATOM 503 C36 POPC 4 10.503 2.758 6.012 1.00 0.00 MEMB C

ATOM 504 H6X POPC 4 10.419 3.763 6.467 1.00 0.00 MEMB H

ATOM 505 H6Y POPC 4 9.474 2.353 5.840 1.00 0.00 MEMB H

ATOM 506 C37 POPC 4 11.179 2.748 4.676 1.00 0.00 MEMB C

ATOM 507 H7X POPC 4 11.048 1.763 4.174 1.00 0.00 MEMB H

ATOM 508 H7Y POPC 4 12.269 2.975 4.727 1.00 0.00 MEMB H

ATOM 509 C38 POPC 4 10.518 3.737 3.805 1.00 0.00 MEMB C

ATOM 510 H8X POPC 4 11.226 3.647 2.966 1.00 0.00 MEMB H

ATOM 511 H8Y POPC 4 10.570 4.736 4.280 1.00 0.00 MEMB H

ATOM 512 C39 POPC 4 9.094 3.340 3.362 1.00 0.00 MEMB C

ATOM 513 H9X POPC 4 8.382 3.704 4.128 1.00 0.00 MEMB H

ATOM 514 H9Y POPC 4 9.060 2.262 3.173 1.00 0.00 MEMB H

ATOM 515 C310 POPC 4 8.753 3.969 2.035 1.00 0.00 MEMB C

ATOM 516 H10X POPC 4 9.481 3.665 1.263 1.00 0.00 MEMB H

ATOM 517 H10Y POPC 4 8.829 4.953 2.533 1.00 0.00 MEMB H

ATOM 518 C311 POPC 4 7.474 4.346 1.372 1.00 0.00 MEMB C

ATOM 519 H11X POPC 4 6.837 4.764 2.177 1.00 0.00 MEMB H

ATOM 520 H11Y POPC 4 7.259 3.284 1.151 1.00 0.00 MEMB H

ATOM 521 C312 POPC 4 7.434 5.353 0.128 1.00 0.00 MEMB C

ATOM 522 H12X POPC 4 7.743 6.388 0.282 1.00 0.00 MEMB H

ATOM 523 H12Y POPC 4 6.403 5.512 -0.168 1.00 0.00 MEMB H

ATOM 524 C313 POPC 4 8.016 5.009 -1.222 1.00 0.00 MEMB C

ATOM 525 H13X POPC 4 9.094 4.934 -1.326 1.00 0.00 MEMB H

ATOM 526 H13Y POPC 4 7.735 5.602 -2.111 1.00 0.00 MEMB H

ATOM 527 C314 POPC 4 7.832 3.614 -1.425 1.00 0.00 MEMB C

ATOM 528 H14X POPC 4 8.271 3.090 -0.551 1.00 0.00 MEMB H

ATOM 529 H14Y POPC 4 8.325 3.535 -2.397 1.00 0.00 MEMB H

ATOM 530 C315 POPC 4 6.581 3.003 -1.651 1.00 0.00 MEMB C

ATOM 531 H15X POPC 4 6.244 2.779 -0.675 1.00 0.00 MEMB H

ATOM 532 H15Y POPC 4 6.466 1.936 -1.954 1.00 0.00 MEMB H

ATOM 533 C316 POPC 4 5.926 3.700 -2.710 1.00 0.00 MEMB C

ATOM 534 H16X POPC 4 5.240 2.901 -2.913 1.00 0.00 MEMB H

ATOM 535 H16Y POPC 4 6.491 3.750 -3.657 1.00 0.00 MEMB H

ATOM 536 H16Z POPC 4 5.416 4.626 -2.430 1.00 0.00 MEMB H

ATOM 537 N POPC 5 -9.920 -2.676 19.333 1.00 0.00 MEMB N

ATOM 538 C12 POPC 5 -9.454 -3.796 20.239 1.00 0.00 MEMB C

ATOM 539 H12A POPC 5 -9.995 -4.712 19.994 1.00 0.00 MEMB H

ATOM 540 H12B POPC 5 -9.706 -3.561 21.266 1.00 0.00 MEMB H

ATOM 541 C13 POPC 5 -9.989 -3.168 17.924 1.00 0.00 MEMB C

ATOM 542 H13A POPC 5 -9.014 -3.579 17.666 1.00 0.00 MEMB H

ATOM 543 H13B POPC 5 -10.675 -4.011 17.852 1.00 0.00 MEMB H

ATOM 544 H13C POPC 5 -10.291 -2.400 17.235 1.00 0.00 MEMB H

ATOM 545 C14 POPC 5 -11.266 -2.347 19.695 1.00 0.00 MEMB C

ATOM 546 H14A POPC 5 -11.303 -2.069 20.741 1.00 0.00 MEMB H

ATOM 547 H14B POPC 5 -11.589 -1.517 19.096 1.00 0.00 MEMB H

ATOM 548 H14C POPC 5 -11.943 -3.190 19.592 1.00 0.00 MEMB H

ATOM 549 C15 POPC 5 -9.058 -1.457 19.488 1.00 0.00 MEMB C

ATOM 550 H15A POPC 5 -8.753 -1.305 20.524 1.00 0.00 MEMB H

ATOM 551 H15B POPC 5 -9.536 -0.541 19.165 1.00 0.00 MEMB H

ATOM 552 H15C POPC 5 -8.148 -1.582 18.913 1.00 0.00 MEMB H

ATOM 553 C11 POPC 5 -7.958 -4.113 20.152 1.00 0.00 MEMB C

ATOM 554 H11A POPC 5 -7.698 -4.889 20.907 1.00 0.00 MEMB H

ATOM 555 H11B POPC 5 -7.374 -3.206 20.416 1.00 0.00 MEMB H

ATOM 556 P POPC 5 -7.686 -6.054 18.442 1.00 0.00 MEMB P

ATOM 557 O13 POPC 5 -8.602 -6.762 19.363 1.00 0.00 MEMB O

ATOM 558 O14 POPC 5 -6.310 -6.584 18.347 1.00 0.00 MEMB O

ATOM 559 O12 POPC 5 -7.609 -4.519 18.836 1.00 0.00 MEMB O

ATOM 560 O11 POPC 5 -8.281 -5.926 16.977 1.00 0.00 MEMB O

ATOM 561 C1 POPC 5 -7.500 -5.283 15.946 1.00 0.00 MEMB C

ATOM 562 HA POPC 5 -6.448 -5.070 16.263 1.00 0.00 MEMB H

ATOM 563 HB POPC 5 -7.963 -4.318 15.652 1.00 0.00 MEMB H

ATOM 564 C2 POPC 5 -7.404 -6.153 14.666 1.00 0.00 MEMB C

ATOM 565 HS POPC 5 -6.642 -5.682 13.998 1.00 0.00 MEMB H

ATOM 566 O21 POPC 5 -8.720 -6.186 14.056 1.00 0.00 MEMB O

ATOM 567 C21 POPC 5 -8.799 -5.800 12.781 1.00 0.00 MEMB C

ATOM 568 O22 POPC 5 -7.878 -5.748 11.992 1.00 0.00 MEMB O

ATOM 569 C22 POPC 5 -10.206 -5.262 12.503 1.00 0.00 MEMB C

ATOM 570 H2R POPC 5 -10.171 -4.153 12.510 1.00 0.00 MEMB H

ATOM 571 H2S POPC 5 -10.884 -5.606 13.316 1.00 0.00 MEMB H

ATOM 572 C3 POPC 5 -6.877 -7.555 15.103 1.00 0.00 MEMB C

ATOM 573 HX POPC 5 -7.526 -7.962 15.899 1.00 0.00 MEMB H

ATOM 574 HY POPC 5 -5.857 -7.446 15.543 1.00 0.00 MEMB H

ATOM 575 O31 POPC 5 -6.898 -8.520 14.064 1.00 0.00 MEMB O

ATOM 576 C31 POPC 5 -5.962 -8.394 13.192 1.00 0.00 MEMB C

ATOM 577 O32 POPC 5 -5.091 -7.534 13.141 1.00 0.00 MEMB O

ATOM 578 C32 POPC 5 -6.150 -9.662 12.312 1.00 0.00 MEMB C

ATOM 579 H2X POPC 5 -7.177 -9.980 12.207 1.00 0.00 MEMB H

ATOM 580 H2Y POPC 5 -5.631 -10.479 12.824 1.00 0.00 MEMB H

ATOM 581 C23 POPC 5 -10.750 -5.706 11.130 1.00 0.00 MEMB C

ATOM 582 H3R POPC 5 -10.543 -6.787 11.066 1.00 0.00 MEMB H

ATOM 583 H3S POPC 5 -10.156 -5.311 10.285 1.00 0.00 MEMB H

ATOM 584 C24 POPC 5 -12.243 -5.285 10.986 1.00 0.00 MEMB C

ATOM 585 H4R POPC 5 -12.271 -4.191 11.169 1.00 0.00 MEMB H

ATOM 586 H4S POPC 5 -12.828 -5.783 11.795 1.00 0.00 MEMB H

ATOM 587 C25 POPC 5 -12.976 -5.506 9.634 1.00 0.00 MEMB C

ATOM 588 H5R POPC 5 -12.414 -5.042 8.795 1.00 0.00 MEMB H

ATOM 589 H5S POPC 5 -14.029 -5.160 9.642 1.00 0.00 MEMB H

ATOM 590 C26 POPC 5 -13.036 -6.964 9.311 1.00 0.00 MEMB C

ATOM 591 H6R POPC 5 -13.580 -7.545 10.074 1.00 0.00 MEMB H

ATOM 592 H6S POPC 5 -11.962 -7.087 9.534 1.00 0.00 MEMB H

ATOM 593 C27 POPC 5 -13.437 -7.408 7.875 1.00 0.00 MEMB C

ATOM 594 H7R POPC 5 -14.479 -7.103 7.673 1.00 0.00 MEMB H

ATOM 595 H7S POPC 5 -13.495 -8.522 7.831 1.00 0.00 MEMB H

ATOM 596 C28 POPC 5 -12.522 -6.912 6.722 1.00 0.00 MEMB C

ATOM 597 H8R POPC 5 -12.295 -5.849 6.802 1.00 0.00 MEMB H

ATOM 598 H8S POPC 5 -13.038 -7.130 5.760 1.00 0.00 MEMB H

ATOM 599 C29 POPC 5 -11.144 -7.443 6.721 1.00 0.00 MEMB C

ATOM 600 H91 POPC 5 -10.599 -7.359 7.659 1.00 0.00 MEMB H

ATOM 601 C210 POPC 5 -10.607 -8.214 5.793 1.00 0.00 MEMB C

ATOM 602 H101 POPC 5 -9.688 -8.775 5.982 1.00 0.00 MEMB H

ATOM 603 C211 POPC 5 -11.322 -8.638 4.618 1.00 0.00 MEMB C

ATOM 604 H11R POPC 5 -12.435 -8.652 4.629 1.00 0.00 MEMB H

ATOM 605 H11S POPC 5 -11.098 -9.594 4.180 1.00 0.00 MEMB H

ATOM 606 C212 POPC 5 -10.406 -7.522 4.075 1.00 0.00 MEMB C

ATOM 607 H12R POPC 5 -10.623 -6.465 4.145 1.00 0.00 MEMB H

ATOM 608 H12S POPC 5 -11.104 -7.578 3.265 1.00 0.00 MEMB H

ATOM 609 C213 POPC 5 -8.822 -7.692 3.812 1.00 0.00 MEMB C

ATOM 610 H13R POPC 5 -8.421 -8.677 3.528 1.00 0.00 MEMB H

ATOM 611 H13S POPC 5 -8.094 -7.837 4.618 1.00 0.00 MEMB H

ATOM 612 C214 POPC 5 -8.338 -6.751 2.701 1.00 0.00 MEMB C

ATOM 613 H14R POPC 5 -8.620 -5.733 2.970 1.00 0.00 MEMB H

ATOM 614 H14S POPC 5 -8.927 -6.778 1.769 1.00 0.00 MEMB H

ATOM 615 C215 POPC 5 -6.839 -6.986 2.544 1.00 0.00 MEMB C

ATOM 616 H15R POPC 5 -6.427 -7.817 1.999 1.00 0.00 MEMB H

ATOM 617 H15S POPC 5 -6.361 -7.043 3.522 1.00 0.00 MEMB H

ATOM 618 C216 POPC 5 -6.106 -6.192 1.588 1.00 0.00 MEMB C

ATOM 619 H16R POPC 5 -6.396 -6.502 0.568 1.00 0.00 MEMB H

ATOM 620 H16S POPC 5 -5.019 -6.423 1.747 1.00 0.00 MEMB H

ATOM 621 C217 POPC 5 -6.394 -4.841 1.939 1.00 0.00 MEMB C

ATOM 622 H17R POPC 5 -6.630 -4.788 3.006 1.00 0.00 MEMB H

ATOM 623 H17S POPC 5 -7.288 -4.616 1.280 1.00 0.00 MEMB H

ATOM 624 C218 POPC 5 -5.099 -4.196 1.641 1.00 0.00 MEMB C

ATOM 625 H18R POPC 5 -4.247 -4.848 1.278 1.00 0.00 MEMB H

ATOM 626 H18S POPC 5 -4.814 -3.161 1.762 1.00 0.00 MEMB H

ATOM 627 H18T POPC 5 -5.616 -3.895 0.742 1.00 0.00 MEMB H

ATOM 628 C33 POPC 5 -5.645 -9.579 10.894 1.00 0.00 MEMB C

ATOM 629 H3X POPC 5 -5.260 -10.506 10.421 1.00 0.00 MEMB H

ATOM 630 H3Y POPC 5 -4.850 -9.020 11.270 1.00 0.00 MEMB H

ATOM 631 C34 POPC 5 -6.066 -8.496 9.953 1.00 0.00 MEMB C

ATOM 632 H4X POPC 5 -5.819 -7.469 10.367 1.00 0.00 MEMB H

ATOM 633 H4Y POPC 5 -7.111 -8.570 9.687 1.00 0.00 MEMB H

ATOM 634 C35 POPC 5 -5.350 -8.640 8.667 1.00 0.00 MEMB C

ATOM 635 H5X POPC 5 -5.113 -9.504 8.057 1.00 0.00 MEMB H

ATOM 636 H5Y POPC 5 -4.343 -8.828 9.033 1.00 0.00 MEMB H

ATOM 637 C36 POPC 5 -5.650 -7.428 7.827 1.00 0.00 MEMB C

ATOM 638 H6X POPC 5 -4.743 -7.677 7.363 1.00 0.00 MEMB H

ATOM 639 H6Y POPC 5 -5.716 -6.381 8.123 1.00 0.00 MEMB H

ATOM 640 C37 POPC 5 -6.721 -7.264 6.837 1.00 0.00 MEMB C

ATOM 641 H7X POPC 5 -7.550 -7.260 7.577 1.00 0.00 MEMB H

ATOM 642 H7Y POPC 5 -6.573 -8.157 6.203 1.00 0.00 MEMB H

ATOM 643 C38 POPC 5 -6.686 -5.940 6.002 1.00 0.00 MEMB C

ATOM 644 H8X POPC 5 -7.502 -6.104 5.288 1.00 0.00 MEMB H

ATOM 645 H8Y POPC 5 -5.804 -5.783 5.354 1.00 0.00 MEMB H

ATOM 646 C39 POPC 5 -6.933 -4.563 6.662 1.00 0.00 MEMB C

ATOM 647 H9X POPC 5 -6.885 -3.809 5.838 1.00 0.00 MEMB H

ATOM 648 H9Y POPC 5 -6.134 -4.272 7.377 1.00 0.00 MEMB H

ATOM 649 C310 POPC 5 -8.304 -4.465 7.333 1.00 0.00 MEMB C

ATOM 650 H10X POPC 5 -8.432 -3.460 7.791 1.00 0.00 MEMB H

ATOM 651 H10Y POPC 5 -8.376 -5.234 8.134 1.00 0.00 MEMB H

ATOM 652 C311 POPC 5 -9.458 -4.684 6.355 1.00 0.00 MEMB C

ATOM 653 H11X POPC 5 -10.411 -4.597 6.918 1.00 0.00 MEMB H

ATOM 654 H11Y POPC 5 -9.404 -5.717 5.955 1.00 0.00 MEMB H

ATOM 655 C312 POPC 5 -9.425 -3.716 5.172 1.00 0.00 MEMB C

ATOM 656 H12X POPC 5 -8.596 -4.027 4.496 1.00 0.00 MEMB H

ATOM 657 H12Y POPC 5 -9.197 -2.690 5.533 1.00 0.00 MEMB H

ATOM 658 C313 POPC 5 -10.731 -3.721 4.385 1.00 0.00 MEMB C

ATOM 659 H13X POPC 5 -11.458 -3.025 4.849 1.00 0.00 MEMB H

ATOM 660 H13Y POPC 5 -11.190 -4.723 4.462 1.00 0.00 MEMB H

ATOM 661 C314 POPC 5 -10.519 -3.418 2.903 1.00 0.00 MEMB C

ATOM 662 H14X POPC 5 -9.440 -3.393 2.644 1.00 0.00 MEMB H

ATOM 663 H14Y POPC 5 -11.012 -2.453 2.648 1.00 0.00 MEMB H

ATOM 664 C315 POPC 5 -11.067 -4.506 2.013 1.00 0.00 MEMB C

ATOM 665 H15X POPC 5 -12.119 -4.635 2.354 1.00 0.00 MEMB H

ATOM 666 H15Y POPC 5 -10.646 -5.496 2.252 1.00 0.00 MEMB H

ATOM 667 C316 POPC 5 -10.867 -4.247 0.489 1.00 0.00 MEMB C

ATOM 668 H16X POPC 5 -11.222 -3.239 0.199 1.00 0.00 MEMB H

ATOM 669 H16Y POPC 5 -11.430 -4.895 -0.209 1.00 0.00 MEMB H

ATOM 670 H16Z POPC 5 -9.799 -4.389 0.249 1.00 0.00 MEMB H

ATOM 671 N POPC 6 -3.558 -10.017 20.930 1.00 0.00 MEMB N

ATOM 672 C12 POPC 6 -2.493 -9.161 20.265 1.00 0.00 MEMB C

ATOM 673 H12A POPC 6 -1.514 -9.622 20.415 1.00 0.00 MEMB H

ATOM 674 H12B POPC 6 -2.496 -8.175 20.690 1.00 0.00 MEMB H

ATOM 675 C13 POPC 6 -4.910 -9.445 20.602 1.00 0.00 MEMB C

ATOM 676 H13A POPC 6 -5.710 -9.980 21.081 1.00 0.00 MEMB H

ATOM 677 H13B POPC 6 -5.006 -8.413 20.926 1.00 0.00 MEMB H

ATOM 678 H13C POPC 6 -5.056 -9.432 19.521 1.00 0.00 MEMB H

ATOM 679 C14 POPC 6 -3.475 -11.456 20.484 1.00 0.00 MEMB C

ATOM 680 H14A POPC 6 -3.382 -11.526 19.391 1.00 0.00 MEMB H

ATOM 681 H14B POPC 6 -2.579 -11.911 20.887 1.00 0.00 MEMB H

ATOM 682 H14C POPC 6 -4.341 -12.015 20.791 1.00 0.00 MEMB H

ATOM 683 C15 POPC 6 -3.308 -9.983 22.387 1.00 0.00 MEMB C

ATOM 684 H15A POPC 6 -4.022 -10.620 22.892 1.00 0.00 MEMB H

ATOM 685 H15B POPC 6 -3.409 -8.973 22.778 1.00 0.00 MEMB H

ATOM 686 H15C POPC 6 -2.293 -10.324 22.561 1.00 0.00 MEMB H

ATOM 687 C11 POPC 6 -2.702 -8.863 18.789 1.00 0.00 MEMB C

ATOM 688 H11A POPC 6 -1.857 -8.219 18.402 1.00 0.00 MEMB H

ATOM 689 H11B POPC 6 -3.637 -8.370 18.528 1.00 0.00 MEMB H

ATOM 690 P POPC 6 -1.742 -10.541 17.356 1.00 0.00 MEMB P

ATOM 691 O13 POPC 6 -2.267 -11.832 16.939 1.00 0.00 MEMB O

ATOM 692 O14 POPC 6 -0.555 -10.424 18.242 1.00 0.00 MEMB O

ATOM 693 O12 POPC 6 -2.908 -10.069 18.157 1.00 0.00 MEMB O

ATOM 694 O11 POPC 6 -1.544 -9.555 16.117 1.00 0.00 MEMB O

ATOM 695 C1 POPC 6 -2.467 -9.674 15.039 1.00 0.00 MEMB C

ATOM 696 HA POPC 6 -2.686 -8.656 14.656 1.00 0.00 MEMB H

ATOM 697 HB POPC 6 -3.429 -10.097 15.412 1.00 0.00 MEMB H

ATOM 698 C2 POPC 6 -2.001 -10.472 13.764 1.00 0.00 MEMB C

ATOM 699 HS POPC 6 -2.020 -11.558 13.888 1.00 0.00 MEMB H

ATOM 700 O21 POPC 6 -0.862 -9.844 13.093 1.00 0.00 MEMB O

ATOM 701 C21 POPC 6 -0.187 -10.627 12.291 1.00 0.00 MEMB C

ATOM 702 O22 POPC 6 -0.110 -11.792 12.343 1.00 0.00 MEMB O

ATOM 703 C22 POPC 6 0.761 -10.140 11.279 1.00 0.00 MEMB C

ATOM 704 H2R POPC 6 1.253 -11.100 10.943 1.00 0.00 MEMB H

ATOM 705 H2S POPC 6 1.517 -9.521 11.759 1.00 0.00 MEMB H

ATOM 706 C3 POPC 6 -3.144 -10.453 12.813 1.00 0.00 MEMB C

ATOM 707 HX POPC 6 -3.962 -10.597 13.553 1.00 0.00 MEMB H

ATOM 708 HY POPC 6 -3.279 -11.292 12.093 1.00 0.00 MEMB H

ATOM 709 O31 POPC 6 -3.023 -9.145 12.256 1.00 0.00 MEMB O

ATOM 710 C31 POPC 6 -2.765 -9.112 10.960 1.00 0.00 MEMB C

ATOM 711 O32 POPC 6 -2.861 -10.074 10.216 1.00 0.00 MEMB O

ATOM 712 C32 POPC 6 -2.990 -7.627 10.617 1.00 0.00 MEMB C

ATOM 713 H2X POPC 6 -2.339 -7.054 11.219 1.00 0.00 MEMB H

ATOM 714 H2Y POPC 6 -3.943 -7.273 11.058 1.00 0.00 MEMB H

ATOM 715 C23 POPC 6 -0.035 -9.622 10.171 1.00 0.00 MEMB C

ATOM 716 H3R POPC 6 -0.385 -8.633 10.525 1.00 0.00 MEMB H

ATOM 717 H3S POPC 6 -0.822 -10.389 9.981 1.00 0.00 MEMB H

ATOM 718 C24 POPC 6 0.685 -9.649 8.882 1.00 0.00 MEMB C

ATOM 719 H4R POPC 6 1.775 -9.566 9.094 1.00 0.00 MEMB H

ATOM 720 H4S POPC 6 0.360 -8.823 8.232 1.00 0.00 MEMB H

ATOM 721 C25 POPC 6 0.371 -10.939 8.142 1.00 0.00 MEMB C

ATOM 722 H5R POPC 6 0.578 -11.845 8.771 1.00 0.00 MEMB H

ATOM 723 H5S POPC 6 1.109 -10.788 7.319 1.00 0.00 MEMB H

ATOM 724 C26 POPC 6 -0.978 -11.086 7.441 1.00 0.00 MEMB C

ATOM 725 H6R POPC 6 -0.932 -10.349 6.607 1.00 0.00 MEMB H

ATOM 726 H6S POPC 6 -1.782 -10.829 8.152 1.00 0.00 MEMB H

ATOM 727 C27 POPC 6 -1.382 -12.462 6.877 1.00 0.00 MEMB C

ATOM 728 H7R POPC 6 -1.512 -13.257 7.632 1.00 0.00 MEMB H

ATOM 729 H7S POPC 6 -0.609 -12.905 6.329 1.00 0.00 MEMB H

ATOM 730 C28 POPC 6 -2.418 -12.385 5.752 1.00 0.00 MEMB C

ATOM 731 H8R POPC 6 -2.517 -13.382 5.270 1.00 0.00 MEMB H

ATOM 732 H8S POPC 6 -2.084 -11.657 4.971 1.00 0.00 MEMB H

ATOM 733 C29 POPC 6 -3.665 -12.004 6.466 1.00 0.00 MEMB C

ATOM 734 H91 POPC 6 -3.896 -12.653 7.319 1.00 0.00 MEMB H

ATOM 735 C210 POPC 6 -4.504 -11.019 6.161 1.00 0.00 MEMB C

ATOM 736 H101 POPC 6 -5.340 -10.843 6.841 1.00 0.00 MEMB H

ATOM 737 C211 POPC 6 -4.419 -10.051 5.030 1.00 0.00 MEMB C

ATOM 738 H11R POPC 6 -3.381 -9.853 4.678 1.00 0.00 MEMB H

ATOM 739 H11S POPC 6 -4.855 -9.066 5.310 1.00 0.00 MEMB H

ATOM 740 C212 POPC 6 -5.245 -10.640 3.903 1.00 0.00 MEMB C

ATOM 741 H12R POPC 6 -6.325 -10.755 4.166 1.00 0.00 MEMB H

ATOM 742 H12S POPC 6 -4.845 -11.600 3.507 1.00 0.00 MEMB H

ATOM 743 C213 POPC 6 -5.255 -9.647 2.806 1.00 0.00 MEMB C

ATOM 744 H13R POPC 6 -5.299 -8.642 3.248 1.00 0.00 MEMB H

ATOM 745 H13S POPC 6 -6.078 -9.949 2.132 1.00 0.00 MEMB H

ATOM 746 C214 POPC 6 -4.054 -9.622 1.946 1.00 0.00 MEMB C

ATOM 747 H14R POPC 6 -4.064 -10.715 1.687 1.00 0.00 MEMB H

ATOM 748 H14S POPC 6 -3.150 -9.386 2.503 1.00 0.00 MEMB H

ATOM 749 C215 POPC 6 -4.327 -8.780 0.683 1.00 0.00 MEMB C

ATOM 750 H15R POPC 6 -4.943 -8.021 0.964 1.00 0.00 MEMB H

ATOM 751 H15S POPC 6 -4.895 -9.476 0.058 1.00 0.00 MEMB H

ATOM 752 C216 POPC 6 -3.555 -7.618 -0.096 1.00 0.00 MEMB C

ATOM 753 H16R POPC 6 -3.461 -8.559 -0.648 1.00 0.00 MEMB H

ATOM 754 H16S POPC 6 -2.712 -7.182 0.453 1.00 0.00 MEMB H

ATOM 755 C217 POPC 6 -3.057 -7.324 -1.544 1.00 0.00 MEMB C

ATOM 756 H17R POPC 6 -3.615 -6.435 -1.544 1.00 0.00 MEMB H

ATOM 757 H17S POPC 6 -3.213 -7.987 -2.383 1.00 0.00 MEMB H

ATOM 758 C218 POPC 6 -1.628 -7.080 -1.847 1.00 0.00 MEMB C

ATOM 759 H18R POPC 6 -1.628 -6.536 -2.797 1.00 0.00 MEMB H

ATOM 760 H18S POPC 6 -0.955 -7.934 -2.079 1.00 0.00 MEMB H

ATOM 761 H18T POPC 6 -1.148 -6.666 -0.942 1.00 0.00 MEMB H

ATOM 762 C33 POPC 6 -2.783 -7.155 9.179 1.00 0.00 MEMB C

ATOM 763 H3X POPC 6 -3.040 -7.821 8.353 1.00 0.00 MEMB H

ATOM 764 H3Y POPC 6 -1.724 -7.200 8.916 1.00 0.00 MEMB H

ATOM 765 C34 POPC 6 -3.615 -5.918 8.839 1.00 0.00 MEMB C

ATOM 766 H4X POPC 6 -3.435 -5.076 9.479 1.00 0.00 MEMB H

ATOM 767 H4Y POPC 6 -4.602 -5.789 9.303 1.00 0.00 MEMB H

ATOM 768 C35 POPC 6 -3.432 -5.653 7.298 1.00 0.00 MEMB C

ATOM 769 H5X POPC 6 -2.353 -5.727 7.514 1.00 0.00 MEMB H

ATOM 770 H5Y POPC 6 -3.837 -4.679 6.979 1.00 0.00 MEMB H

ATOM 771 C36 POPC 6 -3.419 -6.652 6.098 1.00 0.00 MEMB C

ATOM 772 H6X POPC 6 -4.411 -6.932 5.752 1.00 0.00 MEMB H

ATOM 773 H6Y POPC 6 -2.877 -7.588 6.370 1.00 0.00 MEMB H

ATOM 774 C37 POPC 6 -2.963 -6.260 4.773 1.00 0.00 MEMB C

ATOM 775 H7X POPC 6 -3.472 -5.349 4.380 1.00 0.00 MEMB H

ATOM 776 H7Y POPC 6 -3.178 -7.105 4.070 1.00 0.00 MEMB H

ATOM 777 C38 POPC 6 -1.526 -6.239 5.055 1.00 0.00 MEMB C

ATOM 778 H8X POPC 6 -1.095 -6.697 5.966 1.00 0.00 MEMB H

ATOM 779 H8Y POPC 6 -1.306 -5.188 5.125 1.00 0.00 MEMB H

ATOM 780 C39 POPC 6 -0.797 -6.635 3.893 1.00 0.00 MEMB C

ATOM 781 H9X POPC 6 0.187 -6.303 4.274 1.00 0.00 MEMB H

ATOM 782 H9Y POPC 6 -1.361 -6.060 3.120 1.00 0.00 MEMB H

ATOM 783 C310 POPC 6 -0.749 -8.022 3.475 1.00 0.00 MEMB C

ATOM 784 H10X POPC 6 -1.709 -8.157 2.926 1.00 0.00 MEMB H

ATOM 785 H10Y POPC 6 -0.634 -8.614 4.394 1.00 0.00 MEMB H

ATOM 786 C311 POPC 6 0.446 -8.240 2.580 1.00 0.00 MEMB C

ATOM 787 H11X POPC 6 1.301 -7.668 2.954 1.00 0.00 MEMB H

ATOM 788 H11Y POPC 6 0.073 -7.995 1.559 1.00 0.00 MEMB H

ATOM 789 C312 POPC 6 1.062 -9.569 2.521 1.00 0.00 MEMB C

ATOM 790 H12X POPC 6 1.623 -9.872 3.410 1.00 0.00 MEMB H

ATOM 791 H12Y POPC 6 1.712 -9.668 1.589 1.00 0.00 MEMB H

ATOM 792 C313 POPC 6 -0.160 -10.348 2.401 1.00 0.00 MEMB C

ATOM 793 H13X POPC 6 -0.126 -10.133 1.368 1.00 0.00 MEMB H

ATOM 794 H13Y POPC 6 -1.114 -10.219 2.936 1.00 0.00 MEMB H

ATOM 795 C314 POPC 6 0.040 -11.699 2.214 1.00 0.00 MEMB C

ATOM 796 H14X POPC 6 0.484 -11.896 3.136 1.00 0.00 MEMB H

ATOM 797 H14Y POPC 6 0.823 -11.662 1.371 1.00 0.00 MEMB H

ATOM 798 C315 POPC 6 -1.214 -12.435 1.982 1.00 0.00 MEMB C

ATOM 799 H15X POPC 6 -2.245 -12.062 2.111 1.00 0.00 MEMB H

ATOM 800 H15Y POPC 6 -1.468 -13.478 2.225 1.00 0.00 MEMB H

ATOM 801 C316 POPC 6 -0.760 -12.532 0.658 1.00 0.00 MEMB C

ATOM 802 H16X POPC 6 0.082 -12.919 0.070 1.00 0.00 MEMB H

ATOM 803 H16Y POPC 6 -0.491 -11.615 0.122 1.00 0.00 MEMB H

ATOM 804 H16Z POPC 6 -1.534 -13.189 0.363 1.00 0.00 MEMB H

ATOM 805 N POPC 7 -4.882 11.404 19.738 1.00 0.00 MEMB N

ATOM 806 C12 POPC 7 -5.487 11.791 18.390 1.00 0.00 MEMB C

ATOM 807 H12A POPC 7 -6.063 10.963 18.001 1.00 0.00 MEMB H

ATOM 808 H12B POPC 7 -4.680 11.965 17.681 1.00 0.00 MEMB H

ATOM 809 C13 POPC 7 -4.556 9.954 19.798 1.00 0.00 MEMB C

ATOM 810 H13A POPC 7 -3.907 9.631 18.993 1.00 0.00 MEMB H

ATOM 811 H13B POPC 7 -4.059 9.741 20.730 1.00 0.00 MEMB H

ATOM 812 H13C POPC 7 -5.445 9.337 19.828 1.00 0.00 MEMB H

ATOM 813 C14 POPC 7 -3.618 12.187 20.002 1.00 0.00 MEMB C

ATOM 814 H14A POPC 7 -2.895 12.054 19.202 1.00 0.00 MEMB H

ATOM 815 H14B POPC 7 -3.823 13.260 20.032 1.00 0.00 MEMB H

ATOM 816 H14C POPC 7 -3.159 11.901 20.939 1.00 0.00 MEMB H

ATOM 817 C15 POPC 7 -5.876 11.679 20.827 1.00 0.00 MEMB C

ATOM 818 H15A POPC 7 -6.716 11.002 20.718 1.00 0.00 MEMB H

ATOM 819 H15B POPC 7 -6.216 12.705 20.724 1.00 0.00 MEMB H

ATOM 820 H15C POPC 7 -5.455 11.522 21.810 1.00 0.00 MEMB H

ATOM 821 C11 POPC 7 -6.388 13.051 18.344 1.00 0.00 MEMB C

ATOM 822 H11A POPC 7 -7.391 12.854 18.773 1.00 0.00 MEMB H

ATOM 823 H11B POPC 7 -6.591 13.330 17.276 1.00 0.00 MEMB H

ATOM 824 P POPC 7 -4.937 15.209 18.356 1.00 0.00 MEMB P

ATOM 825 O13 POPC 7 -3.860 14.525 17.623 1.00 0.00 MEMB O

ATOM 826 O14 POPC 7 -4.622 16.237 19.363 1.00 0.00 MEMB O

ATOM 827 O12 POPC 7 -5.811 14.115 19.092 1.00 0.00 MEMB O

ATOM 828 O11 POPC 7 -6.026 15.753 17.350 1.00 0.00 MEMB O

ATOM 829 C1 POPC 7 -7.315 16.071 17.893 1.00 0.00 MEMB C

ATOM 830 HA POPC 7 -7.229 16.771 18.759 1.00 0.00 MEMB H

ATOM 831 HB POPC 7 -7.879 15.174 18.234 1.00 0.00 MEMB H

ATOM 832 C2 POPC 7 -8.162 16.715 16.805 1.00 0.00 MEMB C

ATOM 833 HS POPC 7 -9.208 16.856 17.200 1.00 0.00 MEMB H

ATOM 834 O21 POPC 7 -8.174 15.996 15.580 1.00 0.00 MEMB O

ATOM 835 C21 POPC 7 -8.726 14.820 15.605 1.00 0.00 MEMB C

ATOM 836 O22 POPC 7 -9.357 14.345 16.538 1.00 0.00 MEMB O

ATOM 837 C22 POPC 7 -8.335 14.138 14.283 1.00 0.00 MEMB C

ATOM 838 H2R POPC 7 -7.300 14.470 14.044 1.00 0.00 MEMB H

ATOM 839 H2S POPC 7 -9.002 14.512 13.484 1.00 0.00 MEMB H

ATOM 840 C3 POPC 7 -7.581 18.089 16.469 1.00 0.00 MEMB C

ATOM 841 HX POPC 7 -7.253 18.672 17.299 1.00 0.00 MEMB H

ATOM 842 HY POPC 7 -8.508 18.607 16.219 1.00 0.00 MEMB H

ATOM 843 O31 POPC 7 -6.542 18.183 15.501 1.00 0.00 MEMB O

ATOM 844 C31 POPC 7 -5.347 18.466 16.062 1.00 0.00 MEMB C

ATOM 845 O32 POPC 7 -5.161 18.737 17.234 1.00 0.00 MEMB O

ATOM 846 C32 POPC 7 -4.308 18.453 14.986 1.00 0.00 MEMB C

ATOM 847 H2X POPC 7 -3.480 17.833 15.385 1.00 0.00 MEMB H

ATOM 848 H2Y POPC 7 -3.978 19.507 14.862 1.00 0.00 MEMB H

ATOM 849 C23 POPC 7 -8.377 12.594 14.318 1.00 0.00 MEMB C

ATOM 850 H3R POPC 7 -9.407 12.205 14.468 1.00 0.00 MEMB H

ATOM 851 H3S POPC 7 -7.741 12.255 15.165 1.00 0.00 MEMB H

ATOM 852 C24 POPC 7 -7.795 11.979 13.053 1.00 0.00 MEMB C

ATOM 853 H4R POPC 7 -7.656 10.883 13.148 1.00 0.00 MEMB H

ATOM 854 H4S POPC 7 -6.778 12.423 12.917 1.00 0.00 MEMB H

ATOM 855 C25 POPC 7 -8.606 12.201 11.792 1.00 0.00 MEMB C

ATOM 856 H5R POPC 7 -9.126 13.182 11.817 1.00 0.00 MEMB H

ATOM 857 H5S POPC 7 -9.381 11.408 11.666 1.00 0.00 MEMB H

ATOM 858 C26 POPC 7 -7.542 12.186 10.724 1.00 0.00 MEMB C

ATOM 859 H6R POPC 7 -7.190 11.149 10.501 1.00 0.00 MEMB H

ATOM 860 H6S POPC 7 -6.671 12.802 11.027 1.00 0.00 MEMB H

ATOM 861 C27 POPC 7 -7.851 12.891 9.474 1.00 0.00 MEMB C

ATOM 862 H7R POPC 7 -6.814 13.162 9.121 1.00 0.00 MEMB H

ATOM 863 H7S POPC 7 -8.551 13.772 9.555 1.00 0.00 MEMB H

ATOM 864 C28 POPC 7 -8.410 11.857 8.641 1.00 0.00 MEMB C

ATOM 865 H8R POPC 7 -9.416 11.701 9.154 1.00 0.00 MEMB H

ATOM 866 H8S POPC 7 -7.957 10.847 8.653 1.00 0.00 MEMB H

ATOM 867 C29 POPC 7 -8.195 12.724 7.394 1.00 0.00 MEMB C

ATOM 868 H91 POPC 7 -8.966 13.481 7.414 1.00 0.00 MEMB H

ATOM 869 C210 POPC 7 -7.749 12.431 6.167 1.00 0.00 MEMB C

ATOM 870 H101 POPC 7 -8.080 12.828 5.204 1.00 0.00 MEMB H

ATOM 871 C211 POPC 7 -7.385 11.194 5.695 1.00 0.00 MEMB C

ATOM 872 H11R POPC 7 -7.186 10.678 6.629 1.00 0.00 MEMB H

ATOM 873 H11S POPC 7 -6.580 11.565 5.124 1.00 0.00 MEMB H

ATOM 874 C212 POPC 7 -8.112 10.350 4.652 1.00 0.00 MEMB C

ATOM 875 H12R POPC 7 -8.988 10.270 5.213 1.00 0.00 MEMB H

ATOM 876 H12S POPC 7 -7.562 9.406 4.479 1.00 0.00 MEMB H

ATOM 877 C213 POPC 7 -8.770 10.464 3.314 1.00 0.00 MEMB C

ATOM 878 H13R POPC 7 -9.769 10.886 3.257 1.00 0.00 MEMB H

ATOM 879 H13S POPC 7 -8.851 9.438 2.867 1.00 0.00 MEMB H

ATOM 880 C214 POPC 7 -7.962 11.304 2.517 1.00 0.00 MEMB C

ATOM 881 H14R POPC 7 -7.425 12.071 3.131 1.00 0.00 MEMB H

ATOM 882 H14S POPC 7 -8.647 11.677 1.758 1.00 0.00 MEMB H

ATOM 883 C215 POPC 7 -6.923 10.471 2.021 1.00 0.00 MEMB C

ATOM 884 H15R POPC 7 -7.187 9.492 1.566 1.00 0.00 MEMB H

ATOM 885 H15S POPC 7 -6.580 9.987 2.851 1.00 0.00 MEMB H

ATOM 886 C216 POPC 7 -5.871 11.448 1.700 1.00 0.00 MEMB C

ATOM 887 H16R POPC 7 -4.837 11.228 1.664 1.00 0.00 MEMB H

ATOM 888 H16S POPC 7 -5.599 12.330 2.242 1.00 0.00 MEMB H

ATOM 889 C217 POPC 7 -6.534 11.392 0.651 1.00 0.00 MEMB C

ATOM 890 H17R POPC 7 -6.094 12.275 -0.317 1.00 0.00 MEMB H

ATOM 891 H17S POPC 7 -7.003 12.189 0.304 1.00 0.00 MEMB H

ATOM 892 C218 POPC 7 -6.053 9.121 -0.737 1.00 0.00 MEMB C

ATOM 893 H18R POPC 7 -5.457 9.539 -1.778 1.00 0.00 MEMB H

ATOM 894 H18S POPC 7 -5.880 9.899 -1.725 1.00 0.00 MEMB H

ATOM 895 H18T POPC 7 -6.478 8.884 -1.648 1.00 0.00 MEMB H

ATOM 896 C33 POPC 7 -4.866 17.831 13.693 1.00 0.00 MEMB C

ATOM 897 H3X POPC 7 -5.799 18.339 13.365 1.00 0.00 MEMB H

ATOM 898 H3Y POPC 7 -5.128 16.771 13.922 1.00 0.00 MEMB H

ATOM 899 C34 POPC 7 -3.889 17.906 12.531 1.00 0.00 MEMB C

ATOM 900 H4X POPC 7 -2.984 17.310 12.771 1.00 0.00 MEMB H

ATOM 901 H4Y POPC 7 -3.606 18.975 12.397 1.00 0.00 MEMB H

ATOM 902 C35 POPC 7 -4.508 17.408 11.230 1.00 0.00 MEMB C

ATOM 903 H5X POPC 7 -3.796 17.627 10.405 1.00 0.00 MEMB H

ATOM 904 H5Y POPC 7 -5.449 17.978 11.040 1.00 0.00 MEMB H

ATOM 905 C36 POPC 7 -4.852 15.912 11.245 1.00 0.00 MEMB C

ATOM 906 H6X POPC 7 -5.776 15.813 11.865 1.00 0.00 MEMB H

ATOM 907 H6Y POPC 7 -4.061 15.307 11.738 1.00 0.00 MEMB H

ATOM 908 C37 POPC 7 -5.167 15.409 9.826 1.00 0.00 MEMB C

ATOM 909 H7X POPC 7 -5.375 16.308 9.206 1.00 0.00 MEMB H

ATOM 910 H7Y POPC 7 -6.107 14.827 9.873 1.00 0.00 MEMB H

ATOM 911 C38 POPC 7 -4.082 14.580 9.108 1.00 0.00 MEMB C

ATOM 912 H8X POPC 7 -4.334 13.497 9.199 1.00 0.00 MEMB H

ATOM 913 H8Y POPC 7 -3.086 14.746 9.571 1.00 0.00 MEMB H

ATOM 914 C39 POPC 7 -3.942 14.936 7.613 1.00 0.00 MEMB C

ATOM 915 H9X POPC 7 -3.810 16.035 7.506 1.00 0.00 MEMB H

ATOM 916 H9Y POPC 7 -4.904 14.676 7.130 1.00 0.00 MEMB H

ATOM 917 C310 POPC 7 -2.734 14.247 6.941 1.00 0.00 MEMB C

ATOM 918 H10X POPC 7 -2.685 13.182 7.255 1.00 0.00 MEMB H

ATOM 919 H10Y POPC 7 -1.812 14.739 7.327 1.00 0.00 MEMB H

ATOM 920 C311 POPC 7 -2.676 14.332 5.404 1.00 0.00 MEMB C

ATOM 921 H11X POPC 7 -1.613 14.192 5.092 1.00 0.00 MEMB H

ATOM 922 H11Y POPC 7 -2.973 15.341 5.051 1.00 0.00 MEMB H

ATOM 923 C312 POPC 7 -3.437 13.197 4.707 1.00 0.00 MEMB C

ATOM 924 H12X POPC 7 -3.300 12.275 5.308 1.00 0.00 MEMB H

ATOM 925 H12Y POPC 7 -3.000 13.030 3.697 1.00 0.00 MEMB H

ATOM 926 C313 POPC 7 -4.925 13.431 4.562 1.00 0.00 MEMB C

ATOM 927 H13X POPC 7 -5.398 13.611 5.545 1.00 0.00 MEMB H

ATOM 928 H13Y POPC 7 -5.471 12.585 4.134 1.00 0.00 MEMB H

ATOM 929 C314 POPC 7 -5.220 14.519 3.599 1.00 0.00 MEMB C

ATOM 930 H14X POPC 7 -5.293 14.161 2.542 1.00 0.00 MEMB H

ATOM 931 H14Y POPC 7 -4.470 15.331 3.597 1.00 0.00 MEMB H

ATOM 932 C315 POPC 7 -6.473 15.161 4.002 1.00 0.00 MEMB C

ATOM 933 H15X POPC 7 -6.667 15.501 5.036 1.00 0.00 MEMB H

ATOM 934 H15Y POPC 7 -7.305 14.418 3.821 1.00 0.00 MEMB H

ATOM 935 C316 POPC 7 -6.380 16.378 3.135 1.00 0.00 MEMB C

ATOM 936 H16X POPC 7 -6.502 16.023 2.067 1.00 0.00 MEMB H

ATOM 937 H16Y POPC 7 -5.795 17.283 3.101 1.00 0.00 MEMB H

ATOM 938 H16Z POPC 7 -7.240 16.895 3.612 1.00 0.00 MEMB H

ATOM 939 N POPC 8 -13.683 0.127 20.156 1.00 0.00 MEMB N

ATOM 940 C12 POPC 8 -13.218 1.566 20.260 1.00 0.00 MEMB C

ATOM 941 H12A POPC 8 -12.136 1.595 20.378 1.00 0.00 MEMB H

ATOM 942 H12B POPC 8 -13.641 2.032 21.145 1.00 0.00 MEMB H

ATOM 943 C13 POPC 8 -12.994 -0.675 21.177 1.00 0.00 MEMB C

ATOM 944 H13A POPC 8 -13.287 -0.434 22.169 1.00 0.00 MEMB H

ATOM 945 H13B POPC 8 -13.187 -1.734 21.019 1.00 0.00 MEMB H

ATOM 946 H13C POPC 8 -11.919 -0.516 21.103 1.00 0.00 MEMB H

ATOM 947 C14 POPC 8 -15.150 -0.034 20.360 1.00 0.00 MEMB C

ATOM 948 H14A POPC 8 -15.460 -1.022 20.033 1.00 0.00 MEMB H

ATOM 949 H14B POPC 8 -15.420 0.063 21.402 1.00 0.00 MEMB H

ATOM 950 H14C POPC 8 -15.682 0.720 19.782 1.00 0.00 MEMB H

ATOM 951 C15 POPC 8 -13.321 -0.378 18.796 1.00 0.00 MEMB C

ATOM 952 H15A POPC 8 -12.267 -0.172 18.638 1.00 0.00 MEMB H

ATOM 953 H15B POPC 8 -13.866 0.145 18.012 1.00 0.00 MEMB H

ATOM 954 H15C POPC 8 -13.505 -1.443 18.713 1.00 0.00 MEMB H

ATOM 955 C11 POPC 8 -13.506 2.474 19.051 1.00 0.00 MEMB C

ATOM 956 H11A POPC 8 -12.927 2.157 18.155 1.00 0.00 MEMB H

ATOM 957 H11B POPC 8 -13.134 3.496 19.328 1.00 0.00 MEMB H

ATOM 958 P POPC 8 -15.338 3.656 17.733 1.00 0.00 MEMB P

ATOM 959 O13 POPC 8 -14.184 4.040 16.885 1.00 0.00 MEMB O

ATOM 960 O14 POPC 8 -15.872 4.695 18.611 1.00 0.00 MEMB O

ATOM 961 O12 POPC 8 -14.890 2.469 18.726 1.00 0.00 MEMB O

ATOM 962 O11 POPC 8 -16.481 2.946 16.888 1.00 0.00 MEMB O

ATOM 963 C1 POPC 8 -16.928 1.607 17.116 1.00 0.00 MEMB C

ATOM 964 HA POPC 8 -18.037 1.566 17.099 1.00 0.00 MEMB H

ATOM 965 HB POPC 8 -16.635 1.227 18.120 1.00 0.00 MEMB H

ATOM 966 C2 POPC 8 -16.340 0.546 16.138 1.00 0.00 MEMB C

ATOM 967 HS POPC 8 -16.554 -0.413 16.487 1.00 0.00 MEMB H

ATOM 968 O21 POPC 8 -14.951 0.876 15.936 1.00 0.00 MEMB O

ATOM 969 C21 POPC 8 -14.188 0.180 15.147 1.00 0.00 MEMB C

ATOM 970 O22 POPC 8 -14.494 -0.735 14.417 1.00 0.00 MEMB O

ATOM 971 C22 POPC 8 -12.752 0.608 15.323 1.00 0.00 MEMB C

ATOM 972 H2R POPC 8 -12.332 0.224 16.274 1.00 0.00 MEMB H

ATOM 973 H2S POPC 8 -12.768 1.715 15.333 1.00 0.00 MEMB H

ATOM 974 C3 POPC 8 -17.096 0.380 14.839 1.00 0.00 MEMB C

ATOM 975 HX POPC 8 -18.107 0.038 15.128 1.00 0.00 MEMB H

ATOM 976 HY POPC 8 -16.755 -0.293 14.024 1.00 0.00 MEMB H

ATOM 977 O31 POPC 8 -17.307 1.610 14.402 1.00 0.00 MEMB O

ATOM 978 C31 POPC 8 -16.566 1.992 13.511 1.00 0.00 MEMB C

ATOM 979 O32 POPC 8 -15.536 1.583 13.004 1.00 0.00 MEMB O

ATOM 980 C32 POPC 8 -17.224 3.174 13.323 1.00 0.00 MEMB C

ATOM 981 H2X POPC 8 -16.217 3.606 13.599 1.00 0.00 MEMB H

ATOM 982 H2Y POPC 8 -17.817 3.599 14.195 1.00 0.00 MEMB H

ATOM 983 C23 POPC 8 -11.916 0.047 14.159 1.00 0.00 MEMB C

ATOM 984 H3R POPC 8 -11.888 -1.068 14.160 1.00 0.00 MEMB H

ATOM 985 H3S POPC 8 -10.873 0.413 14.279 1.00 0.00 MEMB H

ATOM 986 C24 POPC 8 -12.418 0.487 12.778 1.00 0.00 MEMB C

ATOM 987 H4R POPC 8 -12.750 1.548 12.787 1.00 0.00 MEMB H

ATOM 988 H4S POPC 8 -13.298 -0.134 12.477 1.00 0.00 MEMB H

ATOM 989 C25 POPC 8 -11.303 0.261 11.770 1.00 0.00 MEMB C

ATOM 990 H5R POPC 8 -11.137 -0.840 11.740 1.00 0.00 MEMB H

ATOM 991 H5S POPC 8 -10.374 0.749 12.144 1.00 0.00 MEMB H

ATOM 992 C26 POPC 8 -11.566 0.773 10.358 1.00 0.00 MEMB C

ATOM 993 H6R POPC 8 -10.867 0.235 9.681 1.00 0.00 MEMB H

ATOM 994 H6S POPC 8 -11.335 1.861 10.292 1.00 0.00 MEMB H

ATOM 995 C27 POPC 8 -12.999 0.598 9.859 1.00 0.00 MEMB C

ATOM 996 H7R POPC 8 -13.663 1.231 10.497 1.00 0.00 MEMB H

ATOM 997 H7S POPC 8 -13.343 -0.454 9.972 1.00 0.00 MEMB H

ATOM 998 C28 POPC 8 -13.076 1.040 8.388 1.00 0.00 MEMB C

ATOM 999 H8R POPC 8 -12.422 1.932 8.263 1.00 0.00 MEMB H

ATOM 1000 H8S POPC 8 -14.095 1.396 8.196 1.00 0.00 MEMB H

ATOM 1001 C29 POPC 8 -12.698 -0.062 7.427 1.00 0.00 MEMB C

ATOM 1002 H91 POPC 8 -11.655 -0.403 7.484 1.00 0.00 MEMB H

ATOM 1003 C210 POPC 8 -13.524 -0.687 6.572 1.00 0.00 MEMB C

ATOM 1004 H101 POPC 8 -13.136 -1.480 5.916 1.00 0.00 MEMB H

ATOM 1005 C211 POPC 8 -14.988 -0.411 6.402 1.00 0.00 MEMB C

ATOM 1006 H11R POPC 8 -15.368 0.432 7.017 1.00 0.00 MEMB H

ATOM 1007 H11S POPC 8 -15.593 -1.296 6.662 1.00 0.00 MEMB H

ATOM 1008 C212 POPC 8 -15.266 -0.034 4.974 1.00 0.00 MEMB C

ATOM 1009 H12R POPC 8 -15.031 -0.840 4.255 1.00 0.00 MEMB H

ATOM 1010 H12S POPC 8 -14.673 0.878 4.713 1.00 0.00 MEMB H

ATOM 1011 C213 POPC 8 -16.707 0.327 4.815 1.00 0.00 MEMB C

ATOM 1012 H13R POPC 8 -17.024 1.019 5.625 1.00 0.00 MEMB H

ATOM 1013 H13S POPC 8 -17.413 -0.533 4.765 1.00 0.00 MEMB H

ATOM 1014 C214 POPC 8 -16.742 1.071 3.556 1.00 0.00 MEMB C

ATOM 1015 H14R POPC 8 -16.682 0.358 2.685 1.00 0.00 MEMB H

ATOM 1016 H14S POPC 8 -15.963 1.861 3.423 1.00 0.00 MEMB H

ATOM 1017 C215 POPC 8 -17.955 1.841 3.540 1.00 0.00 MEMB C

ATOM 1018 H15R POPC 8 -17.946 2.806 4.103 1.00 0.00 MEMB H

ATOM 1019 H15S POPC 8 -18.876 1.280 3.898 1.00 0.00 MEMB H

ATOM 1020 C216 POPC 8 -17.846 1.988 2.048 1.00 0.00 MEMB C

ATOM 1021 H16R POPC 8 -18.856 2.146 2.275 1.00 0.00 MEMB H

ATOM 1022 H16S POPC 8 -18.132 1.145 1.394 1.00 0.00 MEMB H

ATOM 1023 C217 POPC 8 -16.912 3.110 1.350 1.00 0.00 MEMB C

ATOM 1024 H17R POPC 8 -16.117 2.448 0.992 1.00 0.00 MEMB H

ATOM 1025 H17S POPC 8 -16.635 3.821 2.156 1.00 0.00 MEMB H

ATOM 1026 C218 POPC 8 -17.122 4.053 0.083 1.00 0.00 MEMB C

ATOM 1027 H18R POPC 8 -17.776 3.638 -0.724 1.00 0.00 MEMB H

ATOM 1028 H18S POPC 8 -16.258 4.472 -0.447 1.00 0.00 MEMB H

ATOM 1029 H18T POPC 8 -17.553 4.993 0.478 1.00 0.00 MEMB H

ATOM 1030 C33 POPC 8 -17.791 3.328 11.868 1.00 0.00 MEMB C

ATOM 1031 H3X POPC 8 -18.272 4.316 12.039 1.00 0.00 MEMB H

ATOM 1032 H3Y POPC 8 -18.748 3.103 11.507 1.00 0.00 MEMB H

ATOM 1033 C34 POPC 8 -16.824 3.034 10.615 1.00 0.00 MEMB C

ATOM 1034 H4X POPC 8 -16.183 2.163 10.754 1.00 0.00 MEMB H

ATOM 1035 H4Y POPC 8 -16.028 3.776 10.401 1.00 0.00 MEMB H

ATOM 1036 C35 POPC 8 -17.459 2.818 9.227 1.00 0.00 MEMB C

ATOM 1037 H5X POPC 8 -18.442 3.079 9.634 1.00 0.00 MEMB H

ATOM 1038 H5Y POPC 8 -18.256 3.295 8.636 1.00 0.00 MEMB H

ATOM 1039 C36 POPC 8 -16.430 3.134 8.075 1.00 0.00 MEMB C

ATOM 1040 H6X POPC 8 -16.933 2.715 7.183 1.00 0.00 MEMB H

ATOM 1041 H6Y POPC 8 -15.549 2.579 8.422 1.00 0.00 MEMB H

ATOM 1042 C37 POPC 8 -15.625 4.347 7.487 1.00 0.00 MEMB C

ATOM 1043 H7X POPC 8 -15.017 4.880 8.223 1.00 0.00 MEMB H

ATOM 1044 H7Y POPC 8 -16.051 5.176 6.889 1.00 0.00 MEMB H

ATOM 1045 C38 POPC 8 -14.933 3.698 6.313 1.00 0.00 MEMB C

ATOM 1046 H8X POPC 8 -15.655 3.249 5.613 1.00 0.00 MEMB H

ATOM 1047 H8Y POPC 8 -14.169 3.036 6.788 1.00 0.00 MEMB H

ATOM 1048 C39 POPC 8 -14.241 4.648 5.469 1.00 0.00 MEMB C

ATOM 1049 H9X POPC 8 -13.200 4.376 5.785 1.00 0.00 MEMB H

ATOM 1050 H9Y POPC 8 -14.558 5.658 5.609 1.00 0.00 MEMB H

ATOM 1051 C310 POPC 8 -14.140 4.838 4.008 1.00 0.00 MEMB C

ATOM 1052 H10X POPC 8 -13.512 5.738 3.767 1.00 0.00 MEMB H

ATOM 1053 H10Y POPC 8 -15.103 5.071 3.524 1.00 0.00 MEMB H

ATOM 1054 C311 POPC 8 -13.240 3.754 3.637 1.00 0.00 MEMB C

ATOM 1055 H11X POPC 8 -13.672 2.833 4.059 1.00 0.00 MEMB H

ATOM 1056 H11Y POPC 8 -12.205 3.822 4.059 1.00 0.00 MEMB H

ATOM 1057 C312 POPC 8 -13.103 3.772 2.207 1.00 0.00 MEMB C

ATOM 1058 H12X POPC 8 -12.341 4.494 1.874 1.00 0.00 MEMB H

ATOM 1059 H12Y POPC 8 -14.045 3.927 1.627 1.00 0.00 MEMB H

ATOM 1060 C313 POPC 8 -12.490 2.496 1.926 1.00 0.00 MEMB C

ATOM 1061 H13X POPC 8 -11.515 2.248 2.383 1.00 0.00 MEMB H

ATOM 1062 H13Y POPC 8 -12.270 2.923 0.966 1.00 0.00 MEMB H

ATOM 1063 C314 POPC 8 -13.491 1.396 2.006 1.00 0.00 MEMB C

ATOM 1064 H14X POPC 8 -14.532 1.618 1.696 1.00 0.00 MEMB H

ATOM 1065 H14Y POPC 8 -13.487 1.105 3.080 1.00 0.00 MEMB H

ATOM 1066 C315 POPC 8 -13.120 0.174 1.310 1.00 0.00 MEMB C

ATOM 1067 H15X POPC 8 -12.026 -0.081 1.300 1.00 0.00 MEMB H

ATOM 1068 H15Y POPC 8 -13.440 0.049 0.242 1.00 0.00 MEMB H

ATOM 1069 C316 POPC 8 -13.895 -0.770 2.102 1.00 0.00 MEMB C

ATOM 1070 H16X POPC 8 -14.975 -0.900 1.853 1.00 0.00 MEMB H

ATOM 1071 H16Y POPC 8 -13.474 -1.282 2.975 1.00 0.00 MEMB H

ATOM 1072 H16Z POPC 8 -13.383 -1.375 1.445 1.00 0.00 MEMB H

ATOM 1073 N POPC 9 9.067 12.875 19.525 1.00 0.00 MEMB N

ATOM 1074 C12 POPC 9 9.780 12.452 18.247 1.00 0.00 MEMB C

ATOM 1075 H12A POPC 9 10.849 12.421 18.421 1.00 0.00 MEMB H

ATOM 1076 H12B POPC 9 9.619 13.192 17.474 1.00 0.00 MEMB H

ATOM 1077 C13 POPC 9 7.611 12.573 19.380 1.00 0.00 MEMB C

ATOM 1078 H13A POPC 9 7.186 13.177 18.588 1.00 0.00 MEMB H

ATOM 1079 H13B POPC 9 7.514 11.518 19.110 1.00 0.00 MEMB H

ATOM 1080 H13C POPC 9 7.101 12.731 20.313 1.00 0.00 MEMB H

ATOM 1081 C14 POPC 9 9.627 12.031 20.635 1.00 0.00 MEMB C

ATOM 1082 H14A POPC 9 10.683 12.204 20.809 1.00 0.00 MEMB H

ATOM 1083 H14B POPC 9 9.092 12.090 21.563 1.00 0.00 MEMB H

ATOM 1084 H14C POPC 9 9.591 10.986 20.290 1.00 0.00 MEMB H

ATOM 1085 C15 POPC 9 9.254 14.335 19.791 1.00 0.00 MEMB C

ATOM 1086 H15A POPC 9 10.297 14.608 19.934 1.00 0.00 MEMB H

ATOM 1087 H15B POPC 9 8.748 14.609 20.705 1.00 0.00 MEMB H

ATOM 1088 H15C POPC 9 8.893 14.945 18.967 1.00 0.00 MEMB H

ATOM 1089 C11 POPC 9 9.369 11.089 17.621 1.00 0.00 MEMB C

ATOM 1090 H11A POPC 9 10.118 10.841 16.831 1.00 0.00 MEMB H

ATOM 1091 H11B POPC 9 8.393 11.224 17.109 1.00 0.00 MEMB H

ATOM 1092 P POPC 9 10.290 8.860 18.705 1.00 0.00 MEMB P

ATOM 1093 O13 POPC 9 9.687 7.781 19.531 1.00 0.00 MEMB O

ATOM 1094 O14 POPC 9 11.540 9.494 19.179 1.00 0.00 MEMB O

ATOM 1095 O12 POPC 9 9.210 10.028 18.558 1.00 0.00 MEMB O

ATOM 1096 O11 POPC 9 10.562 8.305 17.239 1.00 0.00 MEMB O

ATOM 1097 C1 POPC 9 9.600 8.179 16.193 1.00 0.00 MEMB C

ATOM 1098 HA POPC 9 8.841 8.988 16.209 1.00 0.00 MEMB H

ATOM 1099 HB POPC 9 9.064 7.209 16.296 1.00 0.00 MEMB H

ATOM 1100 C2 POPC 9 10.290 8.120 14.801 1.00 0.00 MEMB C

ATOM 1101 HS POPC 9 10.769 7.108 14.798 1.00 0.00 MEMB H

ATOM 1102 O21 POPC 9 11.252 9.199 14.655 1.00 0.00 MEMB O

ATOM 1103 C21 POPC 9 12.138 8.873 13.747 1.00 0.00 MEMB C

ATOM 1104 O22 POPC 9 12.247 7.753 13.262 1.00 0.00 MEMB O

ATOM 1105 C22 POPC 9 13.019 10.126 13.432 1.00 0.00 MEMB C

ATOM 1106 H2R POPC 9 13.902 10.045 14.118 1.00 0.00 MEMB H

ATOM 1107 H2S POPC 9 12.465 11.059 13.536 1.00 0.00 MEMB H

ATOM 1108 C3 POPC 9 9.292 8.167 13.602 1.00 0.00 MEMB C

ATOM 1109 HX POPC 9 8.412 7.539 13.865 1.00 0.00 MEMB H

ATOM 1110 HY POPC 9 9.765 7.714 12.705 1.00 0.00 MEMB H

ATOM 1111 O31 POPC 9 8.972 9.526 13.267 1.00 0.00 MEMB O

ATOM 1112 C31 POPC 9 7.811 9.985 13.663 1.00 0.00 MEMB C

ATOM 1113 O32 POPC 9 6.869 9.331 14.070 1.00 0.00 MEMB O

ATOM 1114 C32 POPC 9 7.926 11.521 13.760 1.00 0.00 MEMB C

ATOM 1115 H2X POPC 9 7.908 11.738 14.849 1.00 0.00 MEMB H

ATOM 1116 H2Y POPC 9 7.014 11.954 13.314 1.00 0.00 MEMB H

ATOM 1117 C23 POPC 9 13.665 10.158 12.046 1.00 0.00 MEMB C

ATOM 1118 H3R POPC 9 13.614 9.114 11.970 1.00 0.00 MEMB H

ATOM 1119 H3S POPC 9 14.764 10.359 12.100 1.00 0.00 MEMB H

ATOM 1120 C24 POPC 9 12.996 10.617 10.732 1.00 0.00 MEMB C

ATOM 1121 H4R POPC 9 13.782 10.585 9.942 1.00 0.00 MEMB H

ATOM 1122 H4S POPC 9 12.837 11.695 10.907 1.00 0.00 MEMB H

ATOM 1123 C25 POPC 9 11.596 10.072 10.219 1.00 0.00 MEMB C

ATOM 1124 H5R POPC 9 11.421 10.629 9.267 1.00 0.00 MEMB H

ATOM 1125 H5S POPC 9 10.888 10.402 10.995 1.00 0.00 MEMB H

ATOM 1126 C26 POPC 9 11.095 8.597 10.027 1.00 0.00 MEMB C

ATOM 1127 H6R POPC 9 11.082 7.875 10.861 1.00 0.00 MEMB H

ATOM 1128 H6S POPC 9 11.761 7.995 9.592 1.00 0.00 MEMB H

ATOM 1129 C27 POPC 9 10.273 8.145 8.824 1.00 0.00 MEMB C

ATOM 1130 H7R POPC 9 10.559 7.077 8.549 1.00 0.00 MEMB H

ATOM 1131 H7S POPC 9 10.432 8.733 7.892 1.00 0.00 MEMB H

ATOM 1132 C28 POPC 9 8.967 8.290 9.500 1.00 0.00 MEMB C

ATOM 1133 H8R POPC 9 8.693 9.355 9.352 1.00 0.00 MEMB H

ATOM 1134 H8S POPC 9 9.095 8.129 10.600 1.00 0.00 MEMB H

ATOM 1135 C29 POPC 9 8.024 7.263 9.039 1.00 0.00 MEMB C

ATOM 1136 H91 POPC 9 8.077 6.338 9.632 1.00 0.00 MEMB H

ATOM 1137 C210 POPC 9 7.224 7.375 7.984 1.00 0.00 MEMB C

ATOM 1138 H101 POPC 9 6.550 6.538 7.801 1.00 0.00 MEMB H

ATOM 1139 C211 POPC 9 7.099 8.545 7.040 1.00 0.00 MEMB C

ATOM 1140 H11R POPC 9 7.772 9.395 7.302 1.00 0.00 MEMB H

ATOM 1141 H11S POPC 9 6.056 8.925 7.059 1.00 0.00 MEMB H

ATOM 1142 C212 POPC 9 7.406 8.160 5.587 1.00 0.00 MEMB C

ATOM 1143 H12R POPC 9 7.048 8.990 4.935 1.00 0.00 MEMB H

ATOM 1144 H12S POPC 9 6.856 7.241 5.292 1.00 0.00 MEMB H

ATOM 1145 C213 POPC 9 8.896 7.941 5.347 1.00 0.00 MEMB C

ATOM 1146 H13R POPC 9 9.292 7.187 6.068 1.00 0.00 MEMB H

ATOM 1147 H13S POPC 9 9.448 8.890 5.529 1.00 0.00 MEMB H

ATOM 1148 C214 POPC 9 9.181 7.412 3.952 1.00 0.00 MEMB C

ATOM 1149 H14R POPC 9 8.823 8.087 3.143 1.00 0.00 MEMB H

ATOM 1150 H14S POPC 9 8.669 6.436 3.830 1.00 0.00 MEMB H

ATOM 1151 C215 POPC 9 10.666 7.273 3.768 1.00 0.00 MEMB C

ATOM 1152 H15R POPC 9 11.168 6.586 4.482 1.00 0.00 MEMB H

ATOM 1153 H15S POPC 9 11.166 8.265 3.901 1.00 0.00 MEMB H

ATOM 1154 C216 POPC 9 11.066 7.048 2.336 1.00 0.00 MEMB C

ATOM 1155 H16R POPC 9 12.129 7.022 2.529 1.00 0.00 MEMB H

ATOM 1156 H16S POPC 9 10.904 7.967 1.842 1.00 0.00 MEMB H

ATOM 1157 C217 POPC 9 10.341 6.100 1.395 1.00 0.00 MEMB C

ATOM 1158 H17R POPC 9 9.299 6.351 1.259 1.00 0.00 MEMB H

ATOM 1159 H17S POPC 9 10.548 5.192 1.947 1.00 0.00 MEMB H

ATOM 1160 C218 POPC 9 10.754 5.594 0.062 1.00 0.00 MEMB C

ATOM 1161 H18R POPC 9 10.706 6.189 -0.839 1.00 0.00 MEMB H

ATOM 1162 H18S POPC 9 10.435 4.544 -0.158 1.00 0.00 MEMB H

ATOM 1163 H18T POPC 9 11.759 5.551 0.276 1.00 0.00 MEMB H

ATOM 1164 C33 POPC 9 9.217 12.172 13.170 1.00 0.00 MEMB C

ATOM 1165 H3X POPC 9 9.486 11.786 12.163 1.00 0.00 MEMB H

ATOM 1166 H3Y POPC 9 10.070 11.916 13.844 1.00 0.00 MEMB H

ATOM 1167 C34 POPC 9 9.133 13.705 13.131 1.00 0.00 MEMB C

ATOM 1168 H4X POPC 9 10.150 14.133 12.984 1.00 0.00 MEMB H

ATOM 1169 H4Y POPC 9 8.775 14.026 14.136 1.00 0.00 MEMB H

ATOM 1170 C35 POPC 9 8.233 14.323 12.037 1.00 0.00 MEMB C

ATOM 1171 H5X POPC 9 8.385 15.426 12.071 1.00 0.00 MEMB H

ATOM 1172 H5Y POPC 9 7.190 14.145 12.332 1.00 0.00 MEMB H

ATOM 1173 C36 POPC 9 8.436 13.777 10.603 1.00 0.00 MEMB C

ATOM 1174 H6X POPC 9 8.020 12.740 10.565 1.00 0.00 MEMB H

ATOM 1175 H6Y POPC 9 9.519 13.728 10.379 1.00 0.00 MEMB H

ATOM 1176 C37 POPC 9 7.835 14.615 9.454 1.00 0.00 MEMB C

ATOM 1177 H7X POPC 9 7.967 15.700 9.595 1.00 0.00 MEMB H

ATOM 1178 H7Y POPC 9 6.762 14.663 9.515 1.00 0.00 MEMB H

ATOM 1179 C38 POPC 9 8.099 14.067 8.021 1.00 0.00 MEMB C

ATOM 1180 H8X POPC 9 9.073 14.399 7.647 1.00 0.00 MEMB H

ATOM 1181 H8Y POPC 9 7.369 14.640 7.390 1.00 0.00 MEMB H

ATOM 1182 C39 POPC 9 7.883 12.514 7.812 1.00 0.00 MEMB C

ATOM 1183 H9X POPC 9 6.975 12.082 8.281 1.00 0.00 MEMB H

ATOM 1184 H9Y POPC 9 8.640 11.933 8.354 1.00 0.00 MEMB H

ATOM 1185 C310 POPC 9 7.757 12.080 6.342 1.00 0.00 MEMB C

ATOM 1186 H10X POPC 9 7.196 12.771 5.667 1.00 0.00 MEMB H

ATOM 1187 H10Y POPC 9 7.237 11.094 6.220 1.00 0.00 MEMB H

ATOM 1188 C311 POPC 9 9.048 12.073 5.735 1.00 0.00 MEMB C

ATOM 1189 H11X POPC 9 9.551 11.249 6.292 1.00 0.00 MEMB H

ATOM 1190 H11Y POPC 9 9.494 13.072 5.948 1.00 0.00 MEMB H

ATOM 1191 C312 POPC 9 8.811 11.845 4.270 1.00 0.00 MEMB C

ATOM 1192 H12X POPC 9 8.354 12.729 3.763 1.00 0.00 MEMB H

ATOM 1193 H12Y POPC 9 8.136 10.980 4.080 1.00 0.00 MEMB H

ATOM 1194 C313 POPC 9 10.166 11.574 3.756 1.00 0.00 MEMB C

ATOM 1195 H13X POPC 9 10.677 10.853 4.438 1.00 0.00 MEMB H

ATOM 1196 H13Y POPC 9 10.731 12.538 3.807 1.00 0.00 MEMB H

ATOM 1197 C314 POPC 9 10.104 11.074 2.339 1.00 0.00 MEMB C

ATOM 1198 H14X POPC 9 9.466 11.735 1.759 1.00 0.00 MEMB H

ATOM 1199 H14Y POPC 9 9.641 10.058 2.292 1.00 0.00 MEMB H

ATOM 1200 C315 POPC 9 11.547 11.107 1.883 1.00 0.00 MEMB C

ATOM 1201 H15X POPC 9 11.882 10.058 2.090 1.00 0.00 MEMB H

ATOM 1202 H15Y POPC 9 12.036 11.814 2.579 1.00 0.00 MEMB H

ATOM 1203 C316 POPC 9 12.109 11.798 0.594 1.00 0.00 MEMB C

ATOM 1204 H16X POPC 9 12.905 11.077 0.894 1.00 0.00 MEMB H

ATOM 1205 H16Y POPC 9 12.532 11.240 -0.285 1.00 0.00 MEMB H

ATOM 1206 H16Z POPC 9 11.098 11.868 0.089 1.00 0.00 MEMB H

ATOM 1207 N POPC 10 11.298 -6.268 18.892 1.00 0.00 MEMB N

ATOM 1208 C12 POPC 10 12.141 -5.719 17.761 1.00 0.00 MEMB C

ATOM 1209 H12A POPC 10 11.725 -4.791 17.383 1.00 0.00 MEMB H

ATOM 1210 H12B POPC 10 12.125 -6.456 16.966 1.00 0.00 MEMB H

ATOM 1211 C13 POPC 10 11.226 -5.255 19.998 1.00 0.00 MEMB C

ATOM 1212 H13A POPC 10 10.656 -5.575 20.855 1.00 0.00 MEMB H

ATOM 1213 H13B POPC 10 12.238 -4.964 20.296 1.00 0.00 MEMB H

ATOM 1214 H13C POPC 10 10.772 -4.334 19.634 1.00 0.00 MEMB H

ATOM 1215 C14 POPC 10 9.877 -6.553 18.474 1.00 0.00 MEMB C

ATOM 1216 H14A POPC 10 9.806 -7.265 17.663 1.00 0.00 MEMB H

ATOM 1217 H14B POPC 10 9.261 -6.880 19.310 1.00 0.00 MEMB H

ATOM 1218 H14C POPC 10 9.405 -5.636 18.124 1.00 0.00 MEMB H

ATOM 1219 C15 POPC 10 12.034 -7.476 19.314 1.00 0.00 MEMB C

ATOM 1220 H15A POPC 10 13.078 -7.304 19.545 1.00 0.00 MEMB H

ATOM 1221 H15B POPC 10 11.950 -8.246 18.549 1.00 0.00 MEMB H

ATOM 1222 H15C POPC 10 11.576 -7.842 20.221 1.00 0.00 MEMB H

ATOM 1223 C11 POPC 10 13.661 -5.474 18.041 1.00 0.00 MEMB C

ATOM 1224 H11A POPC 10 14.050 -4.955 17.132 1.00 0.00 MEMB H

ATOM 1225 H11B POPC 10 14.178 -6.453 18.101 1.00 0.00 MEMB H

ATOM 1226 P POPC 10 14.083 -3.178 19.233 1.00 0.00 MEMB P

ATOM 1227 O13 POPC 10 15.233 -2.694 18.418 1.00 0.00 MEMB O

ATOM 1228 O14 POPC 10 13.921 -2.650 20.605 1.00 0.00 MEMB O

ATOM 1229 O12 POPC 10 13.981 -4.767 19.242 1.00 0.00 MEMB O

ATOM 1230 O11 POPC 10 12.746 -2.883 18.473 1.00 0.00 MEMB O

ATOM 1231 C1 POPC 10 12.001 -1.728 18.828 1.00 0.00 MEMB C

ATOM 1232 HA POPC 10 12.649 -0.856 19.110 1.00 0.00 MEMB H

ATOM 1233 HB POPC 10 11.322 -1.910 19.691 1.00 0.00 MEMB H

ATOM 1234 C2 POPC 10 11.149 -1.260 17.641 1.00 0.00 MEMB C

ATOM 1235 HS POPC 10 10.817 -0.304 17.980 1.00 0.00 MEMB H

ATOM 1236 O21 POPC 10 10.096 -2.181 17.295 1.00 0.00 MEMB O

ATOM 1237 C21 POPC 10 8.885 -2.137 17.806 1.00 0.00 MEMB C

ATOM 1238 O22 POPC 10 8.417 -1.453 18.685 1.00 0.00 MEMB O

ATOM 1239 C22 POPC 10 8.064 -3.300 17.253 1.00 0.00 MEMB C

ATOM 1240 H2R POPC 10 7.121 -2.883 16.853 1.00 0.00 MEMB H

ATOM 1241 H2S POPC 10 7.816 -3.922 18.137 1.00 0.00 MEMB H

ATOM 1242 C3 POPC 10 11.965 -0.966 16.359 1.00 0.00 MEMB C

ATOM 1243 HX POPC 10 12.786 -0.262 16.648 1.00 0.00 MEMB H

ATOM 1244 HY POPC 10 11.320 -0.417 15.632 1.00 0.00 MEMB H

ATOM 1245 O31 POPC 10 12.452 -2.195 15.788 1.00 0.00 MEMB O

ATOM 1246 C31 POPC 10 13.266 -1.995 14.792 1.00 0.00 MEMB C

ATOM 1247 O32 POPC 10 13.613 -0.903 14.373 1.00 0.00 MEMB O

ATOM 1248 C32 POPC 10 13.671 -3.348 14.219 1.00 0.00 MEMB C

ATOM 1249 H2X POPC 10 12.779 -4.010 14.180 1.00 0.00 MEMB H

ATOM 1250 H2Y POPC 10 14.419 -3.809 14.896 1.00 0.00 MEMB H

ATOM 1251 C23 POPC 10 8.798 -4.079 16.153 1.00 0.00 MEMB C

ATOM 1252 H3R POPC 10 9.746 -4.492 16.559 1.00 0.00 MEMB H

ATOM 1253 H3S POPC 10 9.077 -3.345 15.361 1.00 0.00 MEMB H

ATOM 1254 C24 POPC 10 7.957 -5.197 15.538 1.00 0.00 MEMB C

ATOM 1255 H4R POPC 10 6.971 -4.794 15.215 1.00 0.00 MEMB H

ATOM 1256 H4S POPC 10 7.776 -5.980 16.308 1.00 0.00 MEMB H

ATOM 1257 C25 POPC 10 8.650 -5.861 14.340 1.00 0.00 MEMB C

ATOM 1258 H5R POPC 10 8.092 -6.785 14.071 1.00 0.00 MEMB H

ATOM 1259 H5S POPC 10 9.654 -6.163 14.706 1.00 0.00 MEMB H

ATOM 1260 C26 POPC 10 8.770 -4.973 13.079 1.00 0.00 MEMB C

ATOM 1261 H6R POPC 10 8.387 -3.951 13.299 1.00 0.00 MEMB H

ATOM 1262 H6S POPC 10 8.126 -5.394 12.273 1.00 0.00 MEMB H

ATOM 1263 C27 POPC 10 10.207 -4.856 12.536 1.00 0.00 MEMB C

ATOM 1264 H7R POPC 10 10.454 -5.751 11.921 1.00 0.00 MEMB H

ATOM 1265 H7S POPC 10 10.895 -4.862 13.409 1.00 0.00 MEMB H

ATOM 1266 C28 POPC 10 10.493 -3.574 11.719 1.00 0.00 MEMB C

ATOM 1267 H8R POPC 10 11.592 -3.557 11.555 1.00 0.00 MEMB H

ATOM 1268 H8S POPC 10 10.237 -2.670 12.316 1.00 0.00 MEMB H

ATOM 1269 C29 POPC 10 9.797 -3.522 10.386 1.00 0.00 MEMB C

ATOM 1270 H91 POPC 10 8.899 -4.142 10.289 1.00 0.00 MEMB H

ATOM 1271 C210 POPC 10 10.103 -2.696 9.380 1.00 0.00 MEMB C

ATOM 1272 H101 POPC 10 9.513 -2.740 8.451 1.00 0.00 MEMB H

ATOM 1273 C211 POPC 10 11.192 -1.669 9.401 1.00 0.00 MEMB C

ATOM 1274 H11R POPC 10 11.786 -1.628 10.338 1.00 0.00 MEMB H

ATOM 1275 H11S POPC 10 10.616 -0.738 9.435 1.00 0.00 MEMB H

ATOM 1276 C212 POPC 10 12.166 -1.803 8.212 1.00 0.00 MEMB C

ATOM 1277 H12R POPC 10 12.662 -2.794 8.216 1.00 0.00 MEMB H

ATOM 1278 H12S POPC 10 12.977 -1.035 8.299 1.00 0.00 MEMB H

ATOM 1279 C213 POPC 10 11.484 -1.645 6.855 1.00 0.00 MEMB C

ATOM 1280 H13R POPC 10 11.236 -0.611 6.851 1.00 0.00 MEMB H

ATOM 1281 H13S POPC 10 10.497 -2.117 6.725 1.00 0.00 MEMB H

ATOM 1282 C214 POPC 10 12.386 -1.969 5.684 1.00 0.00 MEMB C

ATOM 1283 H14R POPC 10 12.322 -3.060 5.482 1.00 0.00 MEMB H

ATOM 1284 H14S POPC 10 13.440 -1.673 5.894 1.00 0.00 MEMB H

ATOM 1285 C215 POPC 10 11.999 -1.291 4.403 1.00 0.00 MEMB C

ATOM 1286 H15R POPC 10 12.047 -0.188 4.514 1.00 0.00 MEMB H

ATOM 1287 H15S POPC 10 10.941 -1.524 4.131 1.00 0.00 MEMB H

ATOM 1288 C216 POPC 10 12.974 -1.792 3.327 1.00 0.00 MEMB C

ATOM 1289 H16R POPC 10 12.654 -2.805 2.983 1.00 0.00 MEMB H

ATOM 1290 H16S POPC 10 14.035 -1.762 3.616 1.00 0.00 MEMB H

ATOM 1291 C217 POPC 10 12.844 -0.931 2.150 1.00 0.00 MEMB C

ATOM 1292 H17R POPC 10 11.768 -0.989 2.290 1.00 0.00 MEMB H

ATOM 1293 H17S POPC 10 13.140 -1.312 1.148 1.00 0.00 MEMB H

ATOM 1294 C218 POPC 10 13.036 0.519 2.252 1.00 0.00 MEMB C

ATOM 1295 H18R POPC 10 13.971 0.851 1.859 1.00 0.00 MEMB H

ATOM 1296 H18S POPC 10 12.562 1.121 3.050 1.00 0.00 MEMB H

ATOM 1297 H18T POPC 10 12.791 0.990 1.370 1.00 0.00 MEMB H

ATOM 1298 C33 POPC 10 14.245 -3.145 12.812 1.00 0.00 MEMB C

ATOM 1299 H3X POPC 10 14.949 -2.281 12.802 1.00 0.00 MEMB H

ATOM 1300 H3Y POPC 10 13.428 -2.887 12.104 1.00 0.00 MEMB H

ATOM 1301 C34 POPC 10 15.011 -4.360 12.298 1.00 0.00 MEMB C

ATOM 1302 H4X POPC 10 14.448 -5.288 12.542 1.00 0.00 MEMB H

ATOM 1303 H4Y POPC 10 15.985 -4.396 12.837 1.00 0.00 MEMB H

ATOM 1304 C35 POPC 10 15.233 -4.214 10.782 1.00 0.00 MEMB C

ATOM 1305 H5X POPC 10 15.575 -3.166 10.614 1.00 0.00 MEMB H

ATOM 1306 H5Y POPC 10 14.258 -4.336 10.265 1.00 0.00 MEMB H

ATOM 1307 C36 POPC 10 16.317 -5.135 10.194 1.00 0.00 MEMB C

ATOM 1308 H6X POPC 10 16.202 -6.169 10.581 1.00 0.00 MEMB H

ATOM 1309 H6Y POPC 10 17.280 -4.731 10.590 1.00 0.00 MEMB H

ATOM 1310 C37 POPC 10 16.429 -5.169 8.649 1.00 0.00 MEMB C

ATOM 1311 H7X POPC 10 17.393 -5.675 8.405 1.00 0.00 MEMB H

ATOM 1312 H7Y POPC 10 16.508 -4.125 8.275 1.00 0.00 MEMB H

ATOM 1313 C38 POPC 10 15.306 -5.925 7.901 1.00 0.00 MEMB C

ATOM 1314 H8X POPC 10 14.328 -5.450 8.133 1.00 0.00 MEMB H

ATOM 1315 H8Y POPC 10 15.270 -6.979 8.249 1.00 0.00 MEMB H

ATOM 1316 C39 POPC 10 15.488 -6.001 6.373 1.00 0.00 MEMB C

ATOM 1317 H9X POPC 10 16.301 -6.720 6.127 1.00 0.00 MEMB H

ATOM 1318 H9Y POPC 10 15.785 -5.007 5.971 1.00 0.00 MEMB H

ATOM 1319 C310 POPC 10 14.244 -6.492 5.623 1.00 0.00 MEMB C

ATOM 1320 H10X POPC 10 13.343 -5.899 5.904 1.00 0.00 MEMB H

ATOM 1321 H10Y POPC 10 14.034 -7.555 5.879 1.00 0.00 MEMB H

ATOM 1322 C311 POPC 10 14.460 -6.353 4.118 1.00 0.00 MEMB C

ATOM 1323 H11X POPC 10 15.416 -6.815 3.803 1.00 0.00 MEMB H

ATOM 1324 H11Y POPC 10 14.492 -5.262 3.875 1.00 0.00 MEMB H

ATOM 1325 C312 POPC 10 13.337 -6.988 3.320 1.00 0.00 MEMB C

ATOM 1326 H12X POPC 10 13.312 -8.083 3.461 1.00 0.00 MEMB H

ATOM 1327 H12Y POPC 10 13.597 -6.829 2.250 1.00 0.00 MEMB H

ATOM 1328 C313 POPC 10 12.018 -6.344 3.779 1.00 0.00 MEMB C

ATOM 1329 H13X POPC 10 12.162 -5.244 3.940 1.00 0.00 MEMB H

ATOM 1330 H13Y POPC 10 11.673 -6.807 4.717 1.00 0.00 MEMB H

ATOM 1331 C314 POPC 10 10.885 -6.421 2.817 1.00 0.00 MEMB C

ATOM 1332 H14X POPC 10 9.884 -6.580 3.279 1.00 0.00 MEMB H

ATOM 1333 H14Y POPC 10 11.098 -7.288 2.156 1.00 0.00 MEMB H

ATOM 1334 C315 POPC 10 10.944 -5.098 2.086 1.00 0.00 MEMB C

ATOM 1335 H15X POPC 10 10.546 -4.177 2.517 1.00 0.00 MEMB H

ATOM 1336 H15Y POPC 10 9.998 -5.161 1.511 1.00 0.00 MEMB H

ATOM 1337 C316 POPC 10 12.367 -4.922 1.394 1.00 0.00 MEMB C

ATOM 1338 H16X POPC 10 12.439 -4.056 0.718 1.00 0.00 MEMB H

ATOM 1339 H16Y POPC 10 12.603 -5.820 0.761 1.00 0.00 MEMB H

ATOM 1340 H16Z POPC 10 13.250 -4.732 1.996 1.00 0.00 MEMB H

ATOM 1341 N POPC 11 -12.172 9.854 20.813 1.00 0.00 MEMB N

ATOM 1342 C12 POPC 11 -11.404 9.867 19.512 1.00 0.00 MEMB C

ATOM 1343 H12A POPC 11 -10.533 9.239 19.622 1.00 0.00 MEMB H

ATOM 1344 H12B POPC 11 -11.092 10.884 19.285 1.00 0.00 MEMB H

ATOM 1345 C13 POPC 11 -13.075 11.056 20.934 1.00 0.00 MEMB C

ATOM 1346 H13A POPC 11 -13.743 11.122 20.074 1.00 0.00 MEMB H

ATOM 1347 H13B POPC 11 -13.666 11.054 21.845 1.00 0.00 MEMB H

ATOM 1348 H13C POPC 11 -12.517 11.998 20.919 1.00 0.00 MEMB H

ATOM 1349 C14 POPC 11 -12.997 8.628 20.897 1.00 0.00 MEMB C

ATOM 1350 H14A POPC 11 -13.384 8.512 21.895 1.00 0.00 MEMB H

ATOM 1351 H14B POPC 11 -13.818 8.735 20.182 1.00 0.00 MEMB H

ATOM 1352 H14C POPC 11 -12.418 7.774 20.570 1.00 0.00 MEMB H

ATOM 1353 C15 POPC 11 -11.199 9.862 21.926 1.00 0.00 MEMB C

ATOM 1354 H15A POPC 11 -10.533 9.011 21.843 1.00 0.00 MEMB H

ATOM 1355 H15B POPC 11 -11.704 9.827 22.875 1.00 0.00 MEMB H

ATOM 1356 H15C POPC 11 -10.614 10.775 21.912 1.00 0.00 MEMB H

ATOM 1357 C11 POPC 11 -12.130 9.400 18.252 1.00 0.00 MEMB C

ATOM 1358 H11A POPC 11 -12.378 8.322 18.228 1.00 0.00 MEMB H

ATOM 1359 H11B POPC 11 -11.375 9.556 17.437 1.00 0.00 MEMB H

ATOM 1360 P POPC 11 -13.282 11.454 17.216 1.00 0.00 MEMB P

ATOM 1361 O13 POPC 11 -12.613 12.490 18.022 1.00 0.00 MEMB O

ATOM 1362 O14 POPC 11 -14.655 11.722 16.744 1.00 0.00 MEMB O

ATOM 1363 O12 POPC 11 -13.338 10.134 18.096 1.00 0.00 MEMB O

ATOM 1364 O11 POPC 11 -12.331 11.011 16.049 1.00 0.00 MEMB O

ATOM 1365 C1 POPC 11 -12.724 9.967 15.160 1.00 0.00 MEMB C

ATOM 1366 HA POPC 11 -13.702 9.488 15.420 1.00 0.00 MEMB H

ATOM 1367 HB POPC 11 -11.949 9.169 15.183 1.00 0.00 MEMB H

ATOM 1368 C2 POPC 11 -12.852 10.515 13.733 1.00 0.00 MEMB C

ATOM 1369 HS POPC 11 -11.824 10.740 13.355 1.00 0.00 MEMB H

ATOM 1370 O21 POPC 11 -13.686 11.671 13.689 1.00 0.00 MEMB O

ATOM 1371 C21 POPC 11 -13.171 12.856 13.936 1.00 0.00 MEMB C

ATOM 1372 O22 POPC 11 -11.992 13.142 14.043 1.00 0.00 MEMB O

ATOM 1373 C22 POPC 11 -14.340 13.846 13.973 1.00 0.00 MEMB C

ATOM 1374 H2R POPC 11 -13.984 14.851 13.774 1.00 0.00 MEMB H

ATOM 1375 H2S POPC 11 -14.679 13.919 15.022 1.00 0.00 MEMB H

ATOM 1376 C3 POPC 11 -13.525 9.477 12.820 1.00 0.00 MEMB C

ATOM 1377 HX POPC 11 -14.602 9.406 13.027 1.00 0.00 MEMB H

ATOM 1378 HY POPC 11 -13.103 8.446 12.914 1.00 0.00 MEMB H

ATOM 1379 O31 POPC 11 -13.470 9.972 11.507 1.00 0.00 MEMB O

ATOM 1380 C31 POPC 11 -12.342 9.717 10.997 1.00 0.00 MEMB C

ATOM 1381 O32 POPC 11 -11.421 9.073 11.480 1.00 0.00 MEMB O

ATOM 1382 C32 POPC 11 -12.437 10.354 9.686 1.00 0.00 MEMB C

ATOM 1383 H2X POPC 11 -12.193 11.433 9.709 1.00 0.00 MEMB H

ATOM 1384 H2Y POPC 11 -13.345 10.073 9.106 1.00 0.00 MEMB H

ATOM 1385 C23 POPC 11 -15.526 13.457 13.048 1.00 0.00 MEMB C

ATOM 1386 H3R POPC 11 -16.421 14.023 13.384 1.00 0.00 MEMB H

ATOM 1387 H3S POPC 11 -15.783 12.384 13.180 1.00 0.00 MEMB H

ATOM 1388 C24 POPC 11 -15.355 13.737 11.545 1.00 0.00 MEMB C

ATOM 1389 H4R POPC 11 -14.519 13.118 11.153 1.00 0.00 MEMB H

ATOM 1390 H4S POPC 11 -15.111 14.804 11.349 1.00 0.00 MEMB H

ATOM 1391 C25 POPC 11 -16.677 13.443 10.826 1.00 0.00 MEMB C

ATOM 1392 H5R POPC 11 -16.749 14.017 9.880 1.00 0.00 MEMB H

ATOM 1393 H5S POPC 11 -17.510 13.790 11.477 1.00 0.00 MEMB H

ATOM 1394 C26 POPC 11 -16.822 11.960 10.506 1.00 0.00 MEMB C

ATOM 1395 H6R POPC 11 -17.883 11.706 10.278 1.00 0.00 MEMB H

ATOM 1396 H6S POPC 11 -16.528 11.361 11.395 1.00 0.00 MEMB H

ATOM 1397 C27 POPC 11 -15.972 11.603 9.291 1.00 0.00 MEMB C

ATOM 1398 H7R POPC 11 -14.885 11.678 9.514 1.00 0.00 MEMB H

ATOM 1399 H7S POPC 11 -16.198 12.329 8.476 1.00 0.00 MEMB H

ATOM 1400 C28 POPC 11 -16.262 10.210 8.798 1.00 0.00 MEMB C

ATOM 1401 H8R POPC 11 -15.736 10.132 7.813 1.00 0.00 MEMB H

ATOM 1402 H8S POPC 11 -17.349 10.082 8.568 1.00 0.00 MEMB H

ATOM 1403 C29 POPC 11 -15.714 9.179 9.767 1.00 0.00 MEMB C

ATOM 1404 H91 POPC 11 -15.550 9.435 10.816 1.00 0.00 MEMB H

ATOM 1405 C210 POPC 11 -15.412 7.956 9.364 1.00 0.00 MEMB C

ATOM 1406 H101 POPC 11 -15.198 7.110 10.041 1.00 0.00 MEMB H

ATOM 1407 C211 POPC 11 -15.666 7.646 7.963 1.00 0.00 MEMB C

ATOM 1408 H11R POPC 11 -16.461 8.290 7.581 1.00 0.00 MEMB H

ATOM 1409 H11S POPC 11 -16.152 6.693 7.922 1.00 0.00 MEMB H

ATOM 1410 C212 POPC 11 -14.601 7.611 6.916 1.00 0.00 MEMB C

ATOM 1411 H12R POPC 11 -14.445 8.586 6.451 1.00 0.00 MEMB H

ATOM 1412 H12S POPC 11 -15.237 7.159 6.157 1.00 0.00 MEMB H

ATOM 1413 C213 POPC 11 -13.342 6.854 7.363 1.00 0.00 MEMB C

ATOM 1414 H13R POPC 11 -13.523 5.900 7.894 1.00 0.00 MEMB H

ATOM 1415 H13S POPC 11 -12.875 7.448 8.139 1.00 0.00 MEMB H

ATOM 1416 C214 POPC 11 -12.273 6.514 6.337 1.00 0.00 MEMB C

ATOM 1417 H14R POPC 11 -12.681 6.048 5.510 1.00 0.00 MEMB H

ATOM 1418 H14S POPC 11 -11.693 5.604 6.605 1.00 0.00 MEMB H

ATOM 1419 C215 POPC 11 -12.015 7.322 5.098 1.00 0.00 MEMB C

ATOM 1420 H15R POPC 11 -11.762 8.291 5.503 1.00 0.00 MEMB H

ATOM 1421 H15S POPC 11 -12.887 7.546 4.423 1.00 0.00 MEMB H

ATOM 1422 C216 POPC 11 -10.969 6.454 4.350 1.00 0.00 MEMB C

ATOM 1423 H16R POPC 11 -11.302 5.509 3.897 1.00 0.00 MEMB H

ATOM 1424 H16S POPC 11 -10.254 6.085 5.119 1.00 0.00 MEMB H

ATOM 1425 C217 POPC 11 -10.431 7.291 3.245 1.00 0.00 MEMB C

ATOM 1426 H17R POPC 11 -9.596 7.837 3.465 1.00 0.00 MEMB H

ATOM 1427 H17S POPC 11 -11.093 8.148 3.064 1.00 0.00 MEMB H

ATOM 1428 C218 POPC 11 -9.500 6.752 2.231 1.00 0.00 MEMB C

ATOM 1429 H18R POPC 11 -8.831 5.967 2.646 1.00 0.00 MEMB H

ATOM 1430 H18S POPC 11 -8.898 7.570 1.766 1.00 0.00 MEMB H

ATOM 1431 H18T POPC 11 -10.100 6.381 1.426 1.00 0.00 MEMB H

ATOM 1432 C33 POPC 11 -11.445 9.729 8.902 1.00 0.00 MEMB C

ATOM 1433 H3X POPC 11 -11.632 8.635 8.867 1.00 0.00 MEMB H

ATOM 1434 H3Y POPC 11 -10.484 9.834 9.466 1.00 0.00 MEMB H

ATOM 1435 C34 POPC 11 -11.674 10.346 7.523 1.00 0.00 MEMB C

ATOM 1436 H4X POPC 11 -12.560 10.459 6.906 1.00 0.00 MEMB H

ATOM 1437 H4Y POPC 11 -11.589 9.374 7.138 1.00 0.00 MEMB H

ATOM 1438 C35 POPC 11 -10.604 11.270 7.100 1.00 0.00 MEMB C

ATOM 1439 H5X POPC 11 -9.653 10.775 7.141 1.00 0.00 MEMB H

ATOM 1440 H5Y POPC 11 -10.755 11.981 7.939 1.00 0.00 MEMB H

ATOM 1441 C36 POPC 11 -10.507 11.927 5.713 1.00 0.00 MEMB C

ATOM 1442 H6X POPC 11 -10.407 11.332 4.857 1.00 0.00 MEMB H

ATOM 1443 H6Y POPC 11 -9.815 12.791 5.557 1.00 0.00 MEMB H

ATOM 1444 C37 POPC 11 -11.696 12.626 5.577 1.00 0.00 MEMB C

ATOM 1445 H7X POPC 11 -11.287 13.027 6.499 1.00 0.00 MEMB H

ATOM 1446 H7Y POPC 11 -12.575 12.265 6.018 1.00 0.00 MEMB H

ATOM 1447 C38 POPC 11 -11.738 13.722 4.576 1.00 0.00 MEMB C

ATOM 1448 H8X POPC 11 -11.133 13.341 3.738 1.00 0.00 MEMB H

ATOM 1449 H8Y POPC 11 -10.695 14.210 4.761 1.00 0.00 MEMB H

ATOM 1450 C39 POPC 11 -13.119 14.830 4.646 1.00 0.00 MEMB C

ATOM 1451 H9X POPC 11 -13.059 15.972 4.606 1.00 0.00 MEMB H

ATOM 1452 H9Y POPC 11 -13.535 15.388 5.521 1.00 0.00 MEMB H

ATOM 1453 C310 POPC 11 -14.031 14.864 3.570 1.00 0.00 MEMB C

ATOM 1454 H10X POPC 11 -14.680 15.776 3.772 1.00 0.00 MEMB H

ATOM 1455 H10Y POPC 11 -14.272 13.878 4.009 1.00 0.00 MEMB H

ATOM 1456 C311 POPC 11 -13.415 14.882 2.207 1.00 0.00 MEMB C

ATOM 1457 H11X POPC 11 -12.634 14.099 2.073 1.00 0.00 MEMB H

ATOM 1458 H11Y POPC 11 -13.028 15.871 1.846 1.00 0.00 MEMB H

ATOM 1459 C312 POPC 11 -14.504 14.563 1.314 1.00 0.00 MEMB C

ATOM 1460 H12X POPC 11 -15.259 15.333 1.597 1.00 0.00 MEMB H

ATOM 1461 H12Y POPC 11 -14.777 13.531 1.657 1.00 0.00 MEMB H

ATOM 1462 C313 POPC 11 -13.967 14.763 -0.084 1.00 0.00 MEMB C

ATOM 1463 H13X POPC 11 -13.635 15.807 -0.259 1.00 0.00 MEMB H

ATOM 1464 H13Y POPC 11 -14.825 14.494 -0.730 1.00 0.00 MEMB H

ATOM 1465 C314 POPC 11 -12.804 13.871 -0.436 1.00 0.00 MEMB C

ATOM 1466 H14X POPC 11 -11.815 14.004 0.062 1.00 0.00 MEMB H

ATOM 1467 H14Y POPC 11 -12.617 13.980 -1.535 1.00 0.00 MEMB H

ATOM 1468 C315 POPC 11 -13.262 12.504 -0.111 1.00 0.00 MEMB C

ATOM 1469 H15X POPC 11 -14.340 12.313 -0.347 1.00 0.00 MEMB H

ATOM 1470 H15Y POPC 11 -13.046 12.140 0.924 1.00 0.00 MEMB H

ATOM 1471 C316 POPC 11 -12.496 11.742 -1.030 1.00 0.00 MEMB C

ATOM 1472 H16X POPC 11 -13.189 10.874 -1.170 1.00 0.00 MEMB H

ATOM 1473 H16Y POPC 11 -11.505 11.444 -0.599 1.00 0.00 MEMB H

ATOM 1474 H16Z POPC 11 -12.310 12.088 -2.070 1.00 0.00 MEMB H

ATOM 1475 N POPC 12 4.735 16.207 20.720 1.00 0.00 MEMB N

ATOM 1476 C12 POPC 12 4.808 17.293 19.656 1.00 0.00 MEMB C

ATOM 1477 H12A POPC 12 5.840 17.514 19.420 1.00 0.00 MEMB H

ATOM 1478 H12B POPC 12 4.318 18.177 20.052 1.00 0.00 MEMB H

ATOM 1479 C13 POPC 12 5.460 16.686 21.919 1.00 0.00 MEMB C

ATOM 1480 H13A POPC 12 4.931 17.531 22.354 1.00 0.00 MEMB H

ATOM 1481 H13B POPC 12 5.515 15.900 22.661 1.00 0.00 MEMB H

ATOM 1482 H13C POPC 12 6.453 17.008 21.639 1.00 0.00 MEMB H

ATOM 1483 C14 POPC 12 3.307 15.955 21.118 1.00 0.00 MEMB C

ATOM 1484 H14A POPC 12 2.840 16.844 21.543 1.00 0.00 MEMB H

ATOM 1485 H14B POPC 12 2.700 15.695 20.257 1.00 0.00 MEMB H

ATOM 1486 H14C POPC 12 3.221 15.208 21.893 1.00 0.00 MEMB H

ATOM 1487 C15 POPC 12 5.332 14.923 20.243 1.00 0.00 MEMB C

ATOM 1488 H15A POPC 12 6.411 14.997 20.200 1.00 0.00 MEMB H

ATOM 1489 H15B POPC 12 4.941 14.692 19.259 1.00 0.00 MEMB H

ATOM 1490 H15C POPC 12 5.061 14.113 20.911 1.00 0.00 MEMB H

ATOM 1491 C11 POPC 12 4.112 17.026 18.321 1.00 0.00 MEMB C

ATOM 1492 H11A POPC 12 4.486 16.067 17.900 1.00 0.00 MEMB H

ATOM 1493 H11B POPC 12 4.385 17.836 17.594 1.00 0.00 MEMB H

ATOM 1494 P POPC 12 1.762 16.931 17.294 1.00 0.00 MEMB P

ATOM 1495 O13 POPC 12 1.943 18.162 16.494 1.00 0.00 MEMB O

ATOM 1496 O14 POPC 12 0.411 16.487 17.709 1.00 0.00 MEMB O

ATOM 1497 O12 POPC 12 2.710 16.980 18.563 1.00 0.00 MEMB O

ATOM 1498 O11 POPC 12 2.404 15.748 16.475 1.00 0.00 MEMB O

ATOM 1499 C1 POPC 12 2.291 14.377 16.838 1.00 0.00 MEMB C

ATOM 1500 HA POPC 12 1.384 14.157 17.448 1.00 0.00 MEMB H

ATOM 1501 HB POPC 12 3.188 14.119 17.434 1.00 0.00 MEMB H

ATOM 1502 C2 POPC 12 2.295 13.436 15.597 1.00 0.00 MEMB C

ATOM 1503 HS POPC 12 2.556 12.420 15.977 1.00 0.00 MEMB H

ATOM 1504 O21 POPC 12 3.285 13.893 14.667 1.00 0.00 MEMB O

ATOM 1505 C21 POPC 12 3.667 13.014 13.771 1.00 0.00 MEMB C

ATOM 1506 O22 POPC 12 3.482 11.811 13.817 1.00 0.00 MEMB O

ATOM 1507 C22 POPC 12 4.386 13.771 12.693 1.00 0.00 MEMB C

ATOM 1508 H2R POPC 12 5.452 13.778 12.964 1.00 0.00 MEMB H

ATOM 1509 H2S POPC 12 4.036 14.825 12.620 1.00 0.00 MEMB H

ATOM 1510 C3 POPC 12 0.893 13.354 14.906 1.00 0.00 MEMB C

ATOM 1511 HX POPC 12 0.139 13.318 15.724 1.00 0.00 MEMB H

ATOM 1512 HY POPC 12 0.814 12.385 14.355 1.00 0.00 MEMB H

ATOM 1513 O31 POPC 12 0.622 14.515 14.083 1.00 0.00 MEMB O

ATOM 1514 C31 POPC 12 0.784 14.369 12.780 1.00 0.00 MEMB C

ATOM 1515 O32 POPC 12 0.891 13.311 12.190 1.00 0.00 MEMB O

ATOM 1516 C32 POPC 12 0.834 15.739 12.104 1.00 0.00 MEMB C

ATOM 1517 H2X POPC 12 1.525 16.395 12.676 1.00 0.00 MEMB H

ATOM 1518 H2Y POPC 12 -0.185 16.171 12.169 1.00 0.00 MEMB H

ATOM 1519 C23 POPC 12 4.106 13.048 11.395 1.00 0.00 MEMB C

ATOM 1520 H3R POPC 12 3.065 13.183 11.033 1.00 0.00 MEMB H

ATOM 1521 H3S POPC 12 4.390 11.966 11.434 1.00 0.00 MEMB H

ATOM 1522 C24 POPC 12 4.932 13.606 10.323 1.00 0.00 MEMB C

ATOM 1523 H4R POPC 12 5.959 13.412 10.702 1.00 0.00 MEMB H

ATOM 1524 H4S POPC 12 4.806 14.709 10.189 1.00 0.00 MEMB H

ATOM 1525 C25 POPC 12 4.579 12.880 9.041 1.00 0.00 MEMB C

ATOM 1526 H5R POPC 12 3.506 12.695 8.918 1.00 0.00 MEMB H

ATOM 1527 H5S POPC 12 5.101 11.885 9.064 1.00 0.00 MEMB H

ATOM 1528 C26 POPC 12 4.927 13.753 7.855 1.00 0.00 MEMB C

ATOM 1529 H6R POPC 12 5.922 13.657 8.168 1.00 0.00 MEMB H

ATOM 1530 H6S POPC 12 4.933 14.852 7.855 1.00 0.00 MEMB H

ATOM 1531 C27 POPC 12 4.988 13.083 6.518 1.00 0.00 MEMB C

ATOM 1532 H7R POPC 12 5.668 12.281 6.780 1.00 0.00 MEMB H

ATOM 1533 H7S POPC 12 5.426 13.698 5.699 1.00 0.00 MEMB H

ATOM 1534 C28 POPC 12 4.118 11.936 6.195 1.00 0.00 MEMB C

ATOM 1535 H8R POPC 12 4.129 11.132 6.969 1.00 0.00 MEMB H

ATOM 1536 H8S POPC 12 4.563 11.544 5.239 1.00 0.00 MEMB H

ATOM 1537 C29 POPC 12 2.874 12.705 6.115 1.00 0.00 MEMB C

ATOM 1538 H91 POPC 12 2.607 13.349 6.968 1.00 0.00 MEMB H

ATOM 1539 C210 POPC 12 2.058 12.445 5.129 1.00 0.00 MEMB C

ATOM 1540 H101 POPC 12 1.026 12.811 5.214 1.00 0.00 MEMB H

ATOM 1541 C211 POPC 12 2.414 11.344 4.132 1.00 0.00 MEMB C

ATOM 1542 H11R POPC 12 1.696 10.558 3.892 1.00 0.00 MEMB H

ATOM 1543 H11S POPC 12 3.102 10.568 4.515 1.00 0.00 MEMB H

ATOM 1544 C212 POPC 12 2.479 11.882 2.748 1.00 0.00 MEMB C

ATOM 1545 H12R POPC 12 1.733 12.700 2.613 1.00 0.00 MEMB H

ATOM 1546 H12S POPC 12 2.185 11.057 2.060 1.00 0.00 MEMB H

ATOM 1547 C213 POPC 12 3.899 12.270 2.484 1.00 0.00 MEMB C

ATOM 1548 H13R POPC 12 4.087 12.067 1.412 1.00 0.00 MEMB H

ATOM 1549 H13S POPC 12 4.566 11.602 3.081 1.00 0.00 MEMB H

ATOM 1550 C214 POPC 12 4.164 13.719 2.863 1.00 0.00 MEMB C

ATOM 1551 H14R POPC 12 4.040 13.882 3.954 1.00 0.00 MEMB H

ATOM 1552 H14S POPC 12 3.432 14.391 2.367 1.00 0.00 MEMB H

ATOM 1553 C215 POPC 12 5.590 14.075 2.503 1.00 0.00 MEMB C

ATOM 1554 H15R POPC 12 5.773 13.787 1.452 1.00 0.00 MEMB H

ATOM 1555 H15S POPC 12 6.304 13.510 3.141 1.00 0.00 MEMB H

ATOM 1556 C216 POPC 12 5.898 15.524 2.756 1.00 0.00 MEMB C

ATOM 1557 H16R POPC 12 6.983 15.629 2.972 1.00 0.00 MEMB H

ATOM 1558 H16S POPC 12 5.335 15.907 3.636 1.00 0.00 MEMB H

ATOM 1559 C217 POPC 12 5.574 16.438 1.633 1.00 0.00 MEMB C

ATOM 1560 H17R POPC 12 4.525 16.291 1.280 1.00 0.00 MEMB H

ATOM 1561 H17S POPC 12 6.048 16.365 0.637 1.00 0.00 MEMB H

ATOM 1562 C218 POPC 12 5.971 17.811 2.150 1.00 0.00 MEMB C

ATOM 1563 H18R POPC 12 5.085 18.254 2.667 1.00 0.00 MEMB H

ATOM 1564 H18S POPC 12 6.256 18.364 1.237 1.00 0.00 MEMB H

ATOM 1565 H18T POPC 12 6.909 18.002 2.669 1.00 0.00 MEMB H

ATOM 1566 C33 POPC 12 1.291 15.641 10.623 1.00 0.00 MEMB C

ATOM 1567 H3X POPC 12 0.681 14.874 10.094 1.00 0.00 MEMB H

ATOM 1568 H3Y POPC 12 2.357 15.322 10.593 1.00 0.00 MEMB H

ATOM 1569 C34 POPC 12 1.156 16.992 9.906 1.00 0.00 MEMB C

ATOM 1570 H4X POPC 12 1.931 17.664 10.340 1.00 0.00 MEMB H

ATOM 1571 H4Y POPC 12 0.157 17.406 10.184 1.00 0.00 MEMB H

ATOM 1572 C35 POPC 12 1.296 17.006 8.361 1.00 0.00 MEMB C

ATOM 1573 H5X POPC 12 1.344 18.074 8.047 1.00 0.00 MEMB H

ATOM 1574 H5Y POPC 12 0.397 16.554 7.887 1.00 0.00 MEMB H

ATOM 1575 C36 POPC 12 2.529 16.306 7.761 1.00 0.00 MEMB C

ATOM 1576 H6X POPC 12 2.656 15.320 8.257 1.00 0.00 MEMB H

ATOM 1577 H6Y POPC 12 3.440 16.909 7.974 1.00 0.00 MEMB H

ATOM 1578 C37 POPC 12 2.464 16.092 6.226 1.00 0.00 MEMB C

ATOM 1579 H7X POPC 12 1.607 15.434 5.960 1.00 0.00 MEMB H

ATOM 1580 H7Y POPC 12 3.400 15.562 5.938 1.00 0.00 MEMB H

ATOM 1581 C38 POPC 12 2.381 17.391 5.400 1.00 0.00 MEMB C

ATOM 1582 H8X POPC 12 3.227 18.046 5.701 1.00 0.00 MEMB H

ATOM 1583 H8Y POPC 12 1.433 17.914 5.654 1.00 0.00 MEMB H

ATOM 1584 C39 POPC 12 2.404 17.218 3.866 1.00 0.00 MEMB C

ATOM 1585 H9X POPC 12 3.334 16.725 3.512 1.00 0.00 MEMB H

ATOM 1586 H9Y POPC 12 2.358 18.232 3.403 1.00 0.00 MEMB H

ATOM 1587 C310 POPC 12 1.231 16.400 3.332 1.00 0.00 MEMB C

ATOM 1588 H10X POPC 12 0.291 16.828 3.745 1.00 0.00 MEMB H

ATOM 1589 H10Y POPC 12 1.328 15.349 3.689 1.00 0.00 MEMB H

ATOM 1590 C311 POPC 12 1.148 16.398 1.801 1.00 0.00 MEMB C

ATOM 1591 H11X POPC 12 2.052 15.883 1.401 1.00 0.00 MEMB H

ATOM 1592 H11Y POPC 12 1.148 17.440 1.413 1.00 0.00 MEMB H

ATOM 1593 C312 POPC 12 -0.128 15.676 1.359 1.00 0.00 MEMB C

ATOM 1594 H12X POPC 12 -0.956 15.950 2.047 1.00 0.00 MEMB H

ATOM 1595 H12Y POPC 12 0.064 14.583 1.475 1.00 0.00 MEMB H

ATOM 1596 C313 POPC 12 -0.618 15.962 -0.059 1.00 0.00 MEMB C

ATOM 1597 H13X POPC 12 -0.737 17.029 -0.341 1.00 0.00 MEMB H

ATOM 1598 H13Y POPC 12 -1.618 15.467 -0.157 1.00 0.00 MEMB H

ATOM 1599 C314 POPC 12 0.295 15.341 -1.086 1.00 0.00 MEMB C

ATOM 1600 H14X POPC 12 -0.433 14.820 -1.749 1.00 0.00 MEMB H

ATOM 1601 H14Y POPC 12 0.934 14.579 -0.583 1.00 0.00 MEMB H

ATOM 1602 C315 POPC 12 1.195 16.362 -1.804 1.00 0.00 MEMB C

ATOM 1603 H15X POPC 12 1.933 16.757 -1.065 1.00 0.00 MEMB H

ATOM 1604 H15Y POPC 12 0.593 17.094 -2.388 1.00 0.00 MEMB H

ATOM 1605 C316 POPC 12 2.121 15.867 -2.834 1.00 0.00 MEMB C

ATOM 1606 H16X POPC 12 1.562 15.687 -3.789 1.00 0.00 MEMB H

ATOM 1607 H16Y POPC 12 2.528 15.048 -2.228 1.00 0.00 MEMB H

ATOM 1608 H16Z POPC 12 3.085 16.151 -3.193 1.00 0.00 MEMB H

ATOM 1609 N POPC 13 14.338 3.470 20.596 1.00 0.00 MEMB N

ATOM 1610 C12 POPC 13 15.666 2.982 20.024 1.00 0.00 MEMB C

ATOM 1611 H12A POPC 13 15.734 1.914 20.215 1.00 0.00 MEMB H

ATOM 1612 H12B POPC 13 16.499 3.409 20.578 1.00 0.00 MEMB H

ATOM 1613 C13 POPC 13 14.009 2.620 21.771 1.00 0.00 MEMB C

ATOM 1614 H13A POPC 13 12.991 2.821 22.083 1.00 0.00 MEMB H

ATOM 1615 H13B POPC 13 14.017 1.582 21.454 1.00 0.00 MEMB H

ATOM 1616 H13C POPC 13 14.694 2.742 22.595 1.00 0.00 MEMB H

ATOM 1617 C14 POPC 13 14.450 4.897 21.024 1.00 0.00 MEMB C

ATOM 1618 H14A POPC 13 15.307 5.027 21.678 1.00 0.00 MEMB H

ATOM 1619 H14B POPC 13 14.653 5.527 20.155 1.00 0.00 MEMB H

ATOM 1620 H14C POPC 13 13.551 5.230 21.525 1.00 0.00 MEMB H

ATOM 1621 C15 POPC 13 13.212 3.289 19.610 1.00 0.00 MEMB C

ATOM 1622 H15A POPC 13 13.414 2.468 18.921 1.00 0.00 MEMB H

ATOM 1623 H15B POPC 13 13.087 4.185 19.007 1.00 0.00 MEMB H

ATOM 1624 H15C POPC 13 12.273 3.069 20.109 1.00 0.00 MEMB H

ATOM 1625 C11 POPC 13 16.008 3.248 18.538 1.00 0.00 MEMB C

ATOM 1626 H11A POPC 13 15.282 2.782 17.843 1.00 0.00 MEMB H

ATOM 1627 H11B POPC 13 16.984 2.715 18.384 1.00 0.00 MEMB H

ATOM 1628 P POPC 13 17.292 5.236 17.403 1.00 0.00 MEMB P

ATOM 1629 O13 POPC 13 18.473 4.373 17.621 1.00 0.00 MEMB O

ATOM 1630 O14 POPC 13 17.425 6.685 17.696 1.00 0.00 MEMB O

ATOM 1631 O12 POPC 13 16.080 4.650 18.276 1.00 0.00 MEMB O

ATOM 1632 O11 POPC 13 16.848 5.017 15.895 1.00 0.00 MEMB O

ATOM 1633 C1 POPC 13 15.550 5.232 15.373 1.00 0.00 MEMB C

ATOM 1634 HA POPC 13 15.192 6.259 15.633 1.00 0.00 MEMB H

ATOM 1635 HB POPC 13 14.841 4.486 15.784 1.00 0.00 MEMB H

ATOM 1636 C2 POPC 13 15.577 5.115 13.818 1.00 0.00 MEMB C

ATOM 1637 HS POPC 13 14.546 5.353 13.460 1.00 0.00 MEMB H

ATOM 1638 O21 POPC 13 15.982 3.809 13.411 1.00 0.00 MEMB O

ATOM 1639 C21 POPC 13 15.400 3.197 12.399 1.00 0.00 MEMB C

ATOM 1640 O22 POPC 13 14.481 3.603 11.716 1.00 0.00 MEMB O

ATOM 1641 C22 POPC 13 16.082 1.822 12.330 1.00 0.00 MEMB C

ATOM 1642 H2R POPC 13 15.354 1.090 12.730 1.00 0.00 MEMB H

ATOM 1643 H2S POPC 13 16.966 1.834 13.006 1.00 0.00 MEMB H

ATOM 1644 C3 POPC 13 16.636 6.117 13.287 1.00 0.00 MEMB C

ATOM 1645 HX POPC 13 17.619 5.639 13.186 1.00 0.00 MEMB H

ATOM 1646 HY POPC 13 16.786 6.928 14.046 1.00 0.00 MEMB H

ATOM 1647 O31 POPC 13 16.322 6.596 11.981 1.00 0.00 MEMB O

ATOM 1648 C31 POPC 13 15.428 7.522 12.107 1.00 0.00 MEMB C

ATOM 1649 O32 POPC 13 15.012 7.948 13.179 1.00 0.00 MEMB O

ATOM 1650 C32 POPC 13 15.146 8.025 10.692 1.00 0.00 MEMB C

ATOM 1651 H2X POPC 13 15.830 7.638 9.997 1.00 0.00 MEMB H

ATOM 1652 H2Y POPC 13 15.254 9.115 10.587 1.00 0.00 MEMB H

ATOM 1653 C23 POPC 13 16.583 1.410 10.930 1.00 0.00 MEMB C

ATOM 1654 H3R POPC 13 15.716 1.321 10.240 1.00 0.00 MEMB H

ATOM 1655 H3S POPC 13 17.064 0.406 11.011 1.00 0.00 MEMB H

ATOM 1656 C24 POPC 13 17.568 2.413 10.326 1.00 0.00 MEMB C

ATOM 1657 H4R POPC 13 18.612 2.192 10.486 1.00 0.00 MEMB H

ATOM 1658 H4S POPC 13 17.487 3.387 10.842 1.00 0.00 MEMB H

ATOM 1659 C25 POPC 13 17.559 2.402 8.802 1.00 0.00 MEMB C

ATOM 1660 H5R POPC 13 16.508 2.509 8.454 1.00 0.00 MEMB H

ATOM 1661 H5S POPC 13 17.985 1.457 8.408 1.00 0.00 MEMB H

ATOM 1662 C26 POPC 13 18.344 3.555 8.239 1.00 0.00 MEMB C

ATOM 1663 H6R POPC 13 18.290 3.484 7.126 1.00 0.00 MEMB H

ATOM 1664 H6S POPC 13 19.428 3.582 8.499 1.00 0.00 MEMB H

ATOM 1665 C27 POPC 13 17.585 4.783 8.707 1.00 0.00 MEMB C

ATOM 1666 H7R POPC 13 17.740 5.034 9.788 1.00 0.00 MEMB H

ATOM 1667 H7S POPC 13 16.503 4.634 8.481 1.00 0.00 MEMB H

ATOM 1668 C28 POPC 13 17.955 5.824 7.752 1.00 0.00 MEMB C

ATOM 1669 H8R POPC 13 18.285 5.516 6.884 1.00 0.00 MEMB H

ATOM 1670 H8S POPC 13 18.988 6.121 8.406 1.00 0.00 MEMB H

ATOM 1671 C29 POPC 13 17.082 7.172 8.001 1.00 0.00 MEMB C

ATOM 1672 H91 POPC 13 17.030 7.486 8.797 1.00 0.00 MEMB H

ATOM 1673 C210 POPC 13 16.398 7.975 6.768 1.00 0.00 MEMB C

ATOM 1674 H101 POPC 13 16.098 8.943 7.235 1.00 0.00 MEMB H

ATOM 1675 C211 POPC 13 17.129 8.278 5.406 1.00 0.00 MEMB C

ATOM 1676 H11R POPC 13 17.947 7.999 4.875 1.00 0.00 MEMB H

ATOM 1677 H11S POPC 13 17.041 9.326 5.074 1.00 0.00 MEMB H

ATOM 1678 C212 POPC 13 16.166 7.188 4.803 1.00 0.00 MEMB C

ATOM 1679 H12R POPC 13 15.127 7.561 4.885 1.00 0.00 MEMB H

ATOM 1680 H12S POPC 13 16.366 6.218 5.317 1.00 0.00 MEMB H

ATOM 1681 C213 POPC 13 16.493 6.914 3.382 1.00 0.00 MEMB C

ATOM 1682 H13R POPC 13 15.861 6.058 3.085 1.00 0.00 MEMB H

ATOM 1683 H13S POPC 13 17.515 6.549 3.190 1.00 0.00 MEMB H

ATOM 1684 C214 POPC 13 16.141 8.153 2.618 1.00 0.00 MEMB C

ATOM 1685 H14R POPC 13 16.861 8.991 2.841 1.00 0.00 MEMB H

ATOM 1686 H14S POPC 13 15.136 8.524 2.888 1.00 0.00 MEMB H

ATOM 1687 C215 POPC 13 16.068 7.727 1.186 1.00 0.00 MEMB C

ATOM 1688 H15R POPC 13 15.382 8.409 0.616 1.00 0.00 MEMB H

ATOM 1689 H15S POPC 13 15.857 6.664 1.006 1.00 0.00 MEMB H

ATOM 1690 C216 POPC 13 17.394 7.969 0.656 1.00 0.00 MEMB C

ATOM 1691 H16R POPC 13 18.360 7.649 1.088 1.00 0.00 MEMB H

ATOM 1692 H16S POPC 13 17.295 8.983 0.977 1.00 0.00 MEMB H

ATOM 1693 C217 POPC 13 17.387 8.306 -0.744 1.00 0.00 MEMB C

ATOM 1694 H17R POPC 13 18.364 8.780 -0.713 1.00 0.00 MEMB H

ATOM 1695 H17S POPC 13 16.542 8.786 -1.328 1.00 0.00 MEMB H

ATOM 1696 C218 POPC 13 17.765 7.427 -1.515 1.00 0.00 MEMB C

ATOM 1697 H18R POPC 13 16.816 6.893 -1.345 1.00 0.00 MEMB H

ATOM 1698 H18S POPC 13 18.786 7.010 -1.169 1.00 0.00 MEMB H

ATOM 1699 H18T POPC 13 17.647 7.537 -2.537 1.00 0.00 MEMB H

ATOM 1700 C33 POPC 13 13.833 7.574 10.114 1.00 0.00 MEMB C

ATOM 1701 H3X POPC 13 13.093 7.880 10.853 1.00 0.00 MEMB H

ATOM 1702 H3Y POPC 13 13.540 6.524 10.274 1.00 0.00 MEMB H

ATOM 1703 C34 POPC 13 13.674 8.211 8.677 1.00 0.00 MEMB C

ATOM 1704 H4X POPC 13 14.105 9.212 8.572 1.00 0.00 MEMB H

ATOM 1705 H4Y POPC 13 12.710 8.499 8.219 1.00 0.00 MEMB H

ATOM 1706 C35 POPC 13 13.736 7.195 7.609 1.00 0.00 MEMB C

ATOM 1707 H5X POPC 13 14.749 7.081 7.684 1.00 0.00 MEMB H

ATOM 1708 H5Y POPC 13 13.461 7.546 6.593 1.00 0.00 MEMB H

ATOM 1709 C36 POPC 13 13.376 5.770 7.913 1.00 0.00 MEMB C

ATOM 1710 H6X POPC 13 12.283 5.670 8.032 1.00 0.00 MEMB H

ATOM 1711 H6Y POPC 13 13.884 5.367 8.826 1.00 0.00 MEMB H

ATOM 1712 C37 POPC 13 13.963 5.056 6.721 1.00 0.00 MEMB C

ATOM 1713 H7X POPC 13 15.022 5.339 6.558 1.00 0.00 MEMB H

ATOM 1714 H7Y POPC 13 13.354 5.355 5.836 1.00 0.00 MEMB H

ATOM 1715 C38 POPC 13 13.922 3.580 6.894 1.00 0.00 MEMB C

ATOM 1716 H8X POPC 13 12.837 3.497 6.771 1.00 0.00 MEMB H

ATOM 1717 H8Y POPC 13 14.207 3.234 7.911 1.00 0.00 MEMB H

ATOM 1718 C39 POPC 13 14.739 2.886 5.813 1.00 0.00 MEMB C

ATOM 1719 H9X POPC 13 15.802 3.156 5.992 1.00 0.00 MEMB H

ATOM 1720 H9Y POPC 13 14.441 3.310 4.828 1.00 0.00 MEMB H

ATOM 1721 C310 POPC 13 14.607 1.359 5.819 1.00 0.00 MEMB C

ATOM 1722 H10X POPC 13 13.549 1.083 5.605 1.00 0.00 MEMB H

ATOM 1723 H10Y POPC 13 14.887 0.980 6.821 1.00 0.00 MEMB H

ATOM 1724 C311 POPC 13 15.535 0.650 4.838 1.00 0.00 MEMB C

ATOM 1725 H11X POPC 13 15.250 -0.428 4.768 1.00 0.00 MEMB H

ATOM 1726 H11Y POPC 13 16.605 0.759 5.073 1.00 0.00 MEMB H

ATOM 1727 C312 POPC 13 15.414 1.206 3.479 1.00 0.00 MEMB C

ATOM 1728 H12X POPC 13 14.482 1.834 3.469 1.00 0.00 MEMB H

ATOM 1729 H12Y POPC 13 15.379 0.317 2.801 1.00 0.00 MEMB H

ATOM 1730 C313 POPC 13 16.408 2.145 2.946 1.00 0.00 MEMB C

ATOM 1731 H13X POPC 13 17.299 1.752 2.506 1.00 0.00 MEMB H

ATOM 1732 H13Y POPC 13 16.735 2.871 3.697 1.00 0.00 MEMB H

ATOM 1733 C314 POPC 13 15.802 2.901 1.872 1.00 0.00 MEMB C

ATOM 1734 H14X POPC 13 15.211 3.657 2.421 1.00 0.00 MEMB H

ATOM 1735 H14Y POPC 13 15.270 2.452 0.992 1.00 0.00 MEMB H

ATOM 1736 C315 POPC 13 16.861 3.569 1.194 1.00 0.00 MEMB C

ATOM 1737 H15X POPC 13 17.603 4.069 1.820 1.00 0.00 MEMB H

ATOM 1738 H15Y POPC 13 16.233 4.367 0.752 1.00 0.00 MEMB H

ATOM 1739 C316 POPC 13 17.423 2.544 0.228 1.00 0.00 MEMB C

ATOM 1740 H16X POPC 13 16.672 2.511 -0.583 1.00 0.00 MEMB H

ATOM 1741 H16Y POPC 13 18.464 2.260 0.420 1.00 0.00 MEMB H

ATOM 1742 H16Z POPC 13 17.986 2.764 -0.694 1.00 0.00 MEMB H

ATOM 1743 N POPC 14 -8.775 -12.523 18.829 1.00 0.00 MEMB N

ATOM 1744 C12 POPC 14 -9.862 -13.134 17.954 1.00 0.00 MEMB C

ATOM 1745 H12A POPC 14 -10.091 -14.151 18.275 1.00 0.00 MEMB H

ATOM 1746 H12B POPC 14 -9.518 -13.184 16.930 1.00 0.00 MEMB H

ATOM 1747 C13 POPC 14 -7.497 -13.276 18.758 1.00 0.00 MEMB C

ATOM 1748 H13A POPC 14 -6.732 -12.783 19.350 1.00 0.00 MEMB H

ATOM 1749 H13B POPC 14 -7.624 -14.273 19.140 1.00 0.00 MEMB H

ATOM 1750 H13C POPC 14 -7.109 -13.271 17.743 1.00 0.00 MEMB H

ATOM 1751 C14 POPC 14 -8.442 -11.168 18.292 1.00 0.00 MEMB C

ATOM 1752 H14A POPC 14 -7.481 -10.833 18.652 1.00 0.00 MEMB H

ATOM 1753 H14B POPC 14 -8.377 -11.202 17.210 1.00 0.00 MEMB H

ATOM 1754 H14C POPC 14 -9.181 -10.415 18.545 1.00 0.00 MEMB H

ATOM 1755 C15 POPC 14 -9.311 -12.477 20.239 1.00 0.00 MEMB C

ATOM 1756 H15A POPC 14 -10.288 -11.969 20.240 1.00 0.00 MEMB H

ATOM 1757 H15B POPC 14 -8.727 -11.927 20.969 1.00 0.00 MEMB H

ATOM 1758 H15C POPC 14 -9.542 -13.477 20.598 1.00 0.00 MEMB H

ATOM 1759 C11 POPC 14 -11.210 -12.361 17.936 1.00 0.00 MEMB C

ATOM 1760 H11A POPC 14 -11.797 -12.677 17.038 1.00 0.00 MEMB H

ATOM 1761 H11B POPC 14 -10.993 -11.281 17.820 1.00 0.00 MEMB H

ATOM 1762 P POPC 14 -12.828 -13.765 19.398 1.00 0.00 MEMB P

ATOM 1763 O13 POPC 14 -13.415 -13.712 20.755 1.00 0.00 MEMB O

ATOM 1764 O14 POPC 14 -12.017 -14.903 18.951 1.00 0.00 MEMB O

ATOM 1765 O12 POPC 14 -11.949 -12.493 19.153 1.00 0.00 MEMB O

ATOM 1766 O11 POPC 14 -13.958 -13.520 18.391 1.00 0.00 MEMB O

ATOM 1767 C1 POPC 14 -14.769 -12.373 18.336 1.00 0.00 MEMB C

ATOM 1768 HA POPC 14 -14.100 -11.568 17.973 1.00 0.00 MEMB H

ATOM 1769 HB POPC 14 -15.151 -12.083 19.335 1.00 0.00 MEMB H

ATOM 1770 C2 POPC 14 -16.028 -12.515 17.372 1.00 0.00 MEMB C

ATOM 1771 HS POPC 14 -16.936 -12.541 18.025 1.00 0.00 MEMB H

ATOM 1772 O21 POPC 14 -16.144 -13.578 16.596 1.00 0.00 MEMB O

ATOM 1773 C21 POPC 14 -15.878 -14.621 17.210 1.00 0.00 MEMB C

ATOM 1774 O22 POPC 14 -16.322 -15.017 18.266 1.00 0.00 MEMB O

ATOM 1775 C22 POPC 14 -15.025 -15.340 16.254 1.00 0.00 MEMB C

ATOM 1776 H2R POPC 14 -15.427 -14.904 15.334 1.00 0.00 MEMB H

ATOM 1777 H2S POPC 14 -15.349 -16.370 16.321 1.00 0.00 MEMB H

ATOM 1778 C3 POPC 14 -16.165 -11.485 16.287 1.00 0.00 MEMB C

ATOM 1779 HX POPC 14 -15.519 -10.975 16.925 1.00 0.00 MEMB H

ATOM 1780 HY POPC 14 -17.212 -11.193 16.110 1.00 0.00 MEMB H

ATOM 1781 O31 POPC 14 -15.319 -11.363 15.184 1.00 0.00 MEMB O

ATOM 1782 C31 POPC 14 -14.717 -10.198 14.991 1.00 0.00 MEMB C

ATOM 1783 O32 POPC 14 -14.526 -9.305 15.799 1.00 0.00 MEMB O

ATOM 1784 C32 POPC 14 -14.544 -10.194 13.537 1.00 0.00 MEMB C

ATOM 1785 H2X POPC 14 -15.482 -9.817 13.082 1.00 0.00 MEMB H

ATOM 1786 H2Y POPC 14 -14.356 -11.254 13.243 1.00 0.00 MEMB H

ATOM 1787 C23 POPC 14 -13.509 -15.138 16.012 1.00 0.00 MEMB C

ATOM 1788 H3R POPC 14 -12.989 -15.792 16.701 1.00 0.00 MEMB H

ATOM 1789 H3S POPC 14 -13.338 -14.075 16.245 1.00 0.00 MEMB H

ATOM 1790 C24 POPC 14 -12.834 -15.379 14.646 1.00 0.00 MEMB C

ATOM 1791 H4R POPC 14 -13.167 -16.216 13.975 1.00 0.00 MEMB H

ATOM 1792 H4S POPC 14 -11.774 -15.644 14.757 1.00 0.00 MEMB H

ATOM 1793 C25 POPC 14 -12.925 -14.058 13.939 1.00 0.00 MEMB C

ATOM 1794 H5R POPC 14 -13.068 -13.088 14.426 1.00 0.00 MEMB H

ATOM 1795 H5S POPC 14 -13.934 -14.155 13.626 1.00 0.00 MEMB H

ATOM 1796 C26 POPC 14 -12.370 -14.115 12.600 1.00 0.00 MEMB C

ATOM 1797 H6R POPC 14 -12.802 -13.242 12.031 1.00 0.00 MEMB H

ATOM 1798 H6S POPC 14 -12.717 -15.152 12.281 1.00 0.00 MEMB H

ATOM 1799 C27 POPC 14 -10.935 -14.065 12.381 1.00 0.00 MEMB C

ATOM 1800 H7R POPC 14 -10.580 -14.792 13.137 1.00 0.00 MEMB H

ATOM 1801 H7S POPC 14 -10.716 -13.002 12.621 1.00 0.00 MEMB H

ATOM 1802 C28 POPC 14 -10.495 -14.411 10.944 1.00 0.00 MEMB C

ATOM 1803 H8R POPC 14 -9.449 -14.069 10.965 1.00 0.00 MEMB H

ATOM 1804 H8S POPC 14 -11.013 -13.794 10.172 1.00 0.00 MEMB H

ATOM 1805 C29 POPC 14 -10.423 -15.878 10.616 1.00 0.00 MEMB C

ATOM 1806 H91 POPC 14 -10.375 -16.527 11.505 1.00 0.00 MEMB H

ATOM 1807 C210 POPC 14 -10.560 -16.513 9.445 1.00 0.00 MEMB C

ATOM 1808 H101 POPC 14 -10.850 -17.556 9.603 1.00 0.00 MEMB H

ATOM 1809 C211 POPC 14 -10.859 -15.991 8.016 1.00 0.00 MEMB C

ATOM 1810 H11R POPC 14 -10.714 -16.647 7.120 1.00 0.00 MEMB H

ATOM 1811 H11S POPC 14 -10.790 -14.895 7.839 1.00 0.00 MEMB H

ATOM 1812 C212 POPC 14 -12.281 -16.257 7.695 1.00 0.00 MEMB C

ATOM 1813 H12R POPC 14 -12.662 -16.316 8.758 1.00 0.00 MEMB H

ATOM 1814 H12S POPC 14 -12.613 -17.304 7.887 1.00 0.00 MEMB H

ATOM 1815 C213 POPC 14 -13.001 -15.296 6.469 1.00 0.00 MEMB C

ATOM 1816 H13R POPC 14 -13.987 -14.965 6.706 1.00 0.00 MEMB H

ATOM 1817 H13S POPC 14 -13.538 -15.898 5.739 1.00 0.00 MEMB H

ATOM 1818 C214 POPC 14 -13.196 -13.663 6.358 1.00 0.00 MEMB C

ATOM 1819 H14R POPC 14 -12.281 -13.372 6.097 1.00 0.00 MEMB H

ATOM 1820 H14S POPC 14 -13.531 -12.990 7.210 1.00 0.00 MEMB H

ATOM 1821 C215 POPC 14 -14.288 -13.019 5.577 1.00 0.00 MEMB C

ATOM 1822 H15R POPC 14 -15.147 -13.556 6.066 1.00 0.00 MEMB H

ATOM 1823 H15S POPC 14 -14.180 -13.422 4.594 1.00 0.00 MEMB H

ATOM 1824 C216 POPC 14 -14.364 -11.416 5.726 1.00 0.00 MEMB C

ATOM 1825 H16R POPC 14 -13.347 -11.061 5.640 1.00 0.00 MEMB H

ATOM 1826 H16S POPC 14 -14.579 -11.233 6.809 1.00 0.00 MEMB H

ATOM 1827 C217 POPC 14 -15.274 -10.384 4.885 1.00 0.00 MEMB C

ATOM 1828 H17R POPC 14 -15.353 -9.433 5.452 1.00 0.00 MEMB H

ATOM 1829 H17S POPC 14 -16.372 -10.493 4.920 1.00 0.00 MEMB H

ATOM 1830 C218 POPC 14 -15.039 -10.155 3.377 1.00 0.00 MEMB C

ATOM 1831 H18R POPC 14 -13.988 -9.943 3.163 1.00 0.00 MEMB H

ATOM 1832 H18S POPC 14 -15.788 -9.399 3.031 1.00 0.00 MEMB H

ATOM 1833 H18T POPC 14 -15.260 -11.014 2.711 1.00 0.00 MEMB H

ATOM 1834 C33 POPC 14 -13.331 -9.397 13.201 1.00 0.00 MEMB C

ATOM 1835 H3X POPC 14 -12.478 -9.738 13.840 1.00 0.00 MEMB H

ATOM 1836 H3Y POPC 14 -13.522 -8.313 13.379 1.00 0.00 MEMB H

ATOM 1837 C34 POPC 14 -12.940 -9.655 11.768 1.00 0.00 MEMB C

ATOM 1838 H4X POPC 14 -13.712 -9.195 11.113 1.00 0.00 MEMB H

ATOM 1839 H4Y POPC 14 -12.908 -10.736 11.519 1.00 0.00 MEMB H

ATOM 1840 C35 POPC 14 -11.552 -9.067 11.620 1.00 0.00 MEMB C

ATOM 1841 H5X POPC 14 -10.909 -9.362 12.476 1.00 0.00 MEMB H

ATOM 1842 H5Y POPC 14 -11.751 -7.990 11.742 1.00 0.00 MEMB H

ATOM 1843 C36 POPC 14 -10.792 -9.557 10.395 1.00 0.00 MEMB C

ATOM 1844 H6X POPC 14 -10.501 -10.612 10.537 1.00 0.00 MEMB H

ATOM 1845 H6Y POPC 14 -9.885 -8.933 10.186 1.00 0.00 MEMB H

ATOM 1846 C37 POPC 14 -11.634 -9.661 9.167 1.00 0.00 MEMB C

ATOM 1847 H7X POPC 14 -11.593 -8.635 8.860 1.00 0.00 MEMB H

ATOM 1848 H7Y POPC 14 -12.711 -9.890 9.234 1.00 0.00 MEMB H

ATOM 1849 C38 POPC 14 -10.936 -9.973 7.893 1.00 0.00 MEMB C

ATOM 1850 H8X POPC 14 -10.025 -9.298 7.905 1.00 0.00 MEMB H

ATOM 1851 H8Y POPC 14 -11.769 -9.751 7.164 1.00 0.00 MEMB H

ATOM 1852 C39 POPC 14 -10.422 -11.281 7.510 1.00 0.00 MEMB C

ATOM 1853 H9X POPC 14 -11.358 -11.880 7.491 1.00 0.00 MEMB H

ATOM 1854 H9Y POPC 14 -9.706 -11.455 8.332 1.00 0.00 MEMB H

ATOM 1855 C310 POPC 14 -9.684 -11.370 6.170 1.00 0.00 MEMB C

ATOM 1856 H10X POPC 14 -8.999 -12.229 6.266 1.00 0.00 MEMB H

ATOM 1857 H10Y POPC 14 -9.019 -10.533 5.855 1.00 0.00 MEMB H

ATOM 1858 C311 POPC 14 -10.646 -11.695 5.062 1.00 0.00 MEMB C

ATOM 1859 H11X POPC 14 -11.522 -11.049 5.125 1.00 0.00 MEMB H

ATOM 1860 H11Y POPC 14 -11.158 -12.613 5.128 1.00 0.00 MEMB H

ATOM 1861 C312 POPC 14 -9.942 -11.970 3.773 1.00 0.00 MEMB C

ATOM 1862 H12X POPC 14 -9.493 -12.988 3.859 1.00 0.00 MEMB H

ATOM 1863 H12Y POPC 14 -9.100 -11.286 3.582 1.00 0.00 MEMB H

ATOM 1864 C313 POPC 14 -10.878 -12.131 2.624 1.00 0.00 MEMB C

ATOM 1865 H13X POPC 14 -11.427 -11.245 2.293 1.00 0.00 MEMB H

ATOM 1866 H13Y POPC 14 -11.751 -12.752 2.879 1.00 0.00 MEMB H

ATOM 1867 C314 POPC 14 -10.032 -12.733 1.533 1.00 0.00 MEMB C

ATOM 1868 H14X POPC 14 -9.599 -13.720 1.705 1.00 0.00 MEMB H

ATOM 1869 H14Y POPC 14 -9.206 -12.118 1.136 1.00 0.00 MEMB H

ATOM 1870 C315 POPC 14 -10.891 -12.756 0.394 1.00 0.00 MEMB C

ATOM 1871 H15X POPC 14 -10.268 -13.041 -0.469 1.00 0.00 MEMB H

ATOM 1872 H15Y POPC 14 -11.169 -11.667 0.382 1.00 0.00 MEMB H

ATOM 1873 C316 POPC 14 -12.107 -13.618 0.395 1.00 0.00 MEMB C

ATOM 1874 H16X POPC 14 -12.569 -13.449 -0.600 1.00 0.00 MEMB H

ATOM 1875 H16Y POPC 14 -12.889 -13.349 1.128 1.00 0.00 MEMB H

ATOM 1876 H16Z POPC 14 -11.823 -14.687 0.439 1.00 0.00 MEMB H

ATOM 1877 N POPC 15 4.795 -17.174 19.532 1.00 0.00 MEMB N

ATOM 1878 C12 POPC 15 4.922 -15.658 19.676 1.00 0.00 MEMB C

ATOM 1879 H12A POPC 15 5.664 -15.325 18.955 1.00 0.00 MEMB H

ATOM 1880 H12B POPC 15 5.401 -15.442 20.625 1.00 0.00 MEMB H

ATOM 1881 C13 POPC 15 4.473 -17.540 18.107 1.00 0.00 MEMB C

ATOM 1882 H13A POPC 15 3.410 -17.739 17.967 1.00 0.00 MEMB H

ATOM 1883 H13B POPC 15 4.728 -16.704 17.463 1.00 0.00 MEMB H

ATOM 1884 H13C POPC 15 5.042 -18.405 17.787 1.00 0.00 MEMB H

ATOM 1885 C14 POPC 15 6.139 -17.755 19.800 1.00 0.00 MEMB C

ATOM 1886 H14A POPC 15 6.083 -18.808 20.068 1.00 0.00 MEMB H

ATOM 1887 H14B POPC 15 6.782 -17.749 18.915 1.00 0.00 MEMB H

ATOM 1888 H14C POPC 15 6.697 -17.246 20.572 1.00 0.00 MEMB H

ATOM 1889 C15 POPC 15 3.773 -17.756 20.445 1.00 0.00 MEMB C

ATOM 1890 H15A POPC 15 4.116 -17.763 21.466 1.00 0.00 MEMB H

ATOM 1891 H15B POPC 15 3.533 -18.772 20.154 1.00 0.00 MEMB H

ATOM 1892 H15C POPC 15 2.852 -17.185 20.347 1.00 0.00 MEMB H

ATOM 1893 C11 POPC 15 3.716 -14.704 19.530 1.00 0.00 MEMB C

ATOM 1894 H11A POPC 15 4.111 -13.661 19.479 1.00 0.00 MEMB H

ATOM 1895 H11B POPC 15 3.098 -14.747 20.459 1.00 0.00 MEMB H

ATOM 1896 P POPC 15 1.447 -15.335 18.405 1.00 0.00 MEMB P

ATOM 1897 O13 POPC 15 0.887 -14.892 19.704 1.00 0.00 MEMB O

ATOM 1898 O14 POPC 15 1.254 -16.734 17.987 1.00 0.00 MEMB O

ATOM 1899 O12 POPC 15 2.996 -14.978 18.335 1.00 0.00 MEMB O

ATOM 1900 O11 POPC 15 0.948 -14.401 17.281 1.00 0.00 MEMB O

ATOM 1901 C1 POPC 15 1.394 -13.043 17.296 1.00 0.00 MEMB C

ATOM 1902 HA POPC 15 2.444 -12.903 16.995 1.00 0.00 MEMB H

ATOM 1903 HB POPC 15 1.170 -12.549 18.292 1.00 0.00 MEMB H

ATOM 1904 C2 POPC 15 0.547 -12.359 16.243 1.00 0.00 MEMB C

ATOM 1905 HS POPC 15 -0.324 -12.523 16.868 1.00 0.00 MEMB H

ATOM 1906 O21 POPC 15 0.456 -13.096 15.010 1.00 0.00 MEMB O

ATOM 1907 C21 POPC 15 -0.730 -13.450 14.506 1.00 0.00 MEMB C

ATOM 1908 O22 POPC 15 -1.836 -13.355 14.977 1.00 0.00 MEMB O

ATOM 1909 C22 POPC 15 -0.563 -14.115 13.142 1.00 0.00 MEMB C

ATOM 1910 H2R POPC 15 -0.540 -15.176 13.372 1.00 0.00 MEMB H

ATOM 1911 H2S POPC 15 0.482 -13.832 12.853 1.00 0.00 MEMB H

ATOM 1912 C3 POPC 15 1.024 -10.901 15.899 1.00 0.00 MEMB C

ATOM 1913 HX POPC 15 0.901 -10.114 16.640 1.00 0.00 MEMB H

ATOM 1914 HY POPC 15 0.364 -10.501 15.085 1.00 0.00 MEMB H

ATOM 1915 O31 POPC 15 2.440 -10.886 15.690 1.00 0.00 MEMB O

ATOM 1916 C31 POPC 15 2.662 -11.099 14.442 1.00 0.00 MEMB C

ATOM 1917 O32 POPC 15 1.928 -10.870 13.541 1.00 0.00 MEMB O

ATOM 1918 C32 POPC 15 3.932 -11.773 14.211 1.00 0.00 MEMB C

ATOM 1919 H2X POPC 15 3.833 -12.714 14.794 1.00 0.00 MEMB H

ATOM 1920 H2Y POPC 15 4.684 -11.059 14.587 1.00 0.00 MEMB H

ATOM 1921 C23 POPC 15 -1.594 -13.748 11.948 1.00 0.00 MEMB C

ATOM 1922 H3R POPC 15 -1.951 -12.701 11.881 1.00 0.00 MEMB H

ATOM 1923 H3S POPC 15 -2.569 -14.209 12.068 1.00 0.00 MEMB H

ATOM 1924 C24 POPC 15 -0.887 -13.809 10.574 1.00 0.00 MEMB C

ATOM 1925 H4R POPC 15 -0.178 -12.971 10.362 1.00 0.00 MEMB H

ATOM 1926 H4S POPC 15 -1.613 -13.723 9.741 1.00 0.00 MEMB H

ATOM 1927 C25 POPC 15 0.007 -15.027 10.598 1.00 0.00 MEMB C

ATOM 1928 H5R POPC 15 -0.429 -15.734 11.346 1.00 0.00 MEMB H

ATOM 1929 H5S POPC 15 1.033 -14.777 10.929 1.00 0.00 MEMB H

ATOM 1930 C26 POPC 15 0.050 -15.711 9.281 1.00 0.00 MEMB C

ATOM 1931 H6R POPC 15 0.846 -16.487 9.267 1.00 0.00 MEMB H

ATOM 1932 H6S POPC 15 0.106 -15.039 8.399 1.00 0.00 MEMB H

ATOM 1933 C27 POPC 15 -1.225 -16.422 9.202 1.00 0.00 MEMB C

ATOM 1934 H7R POPC 15 -2.100 -15.727 9.158 1.00 0.00 MEMB H

ATOM 1935 H7S POPC 15 -1.332 -17.062 10.119 1.00 0.00 MEMB H

ATOM 1936 C28 POPC 15 -1.245 -17.345 8.039 1.00 0.00 MEMB C

ATOM 1937 H8R POPC 15 -2.302 -17.460 8.158 1.00 0.00 MEMB H

ATOM 1938 H8S POPC 15 -0.693 -18.298 8.244 1.00 0.00 MEMB H

ATOM 1939 C29 POPC 15 -1.369 -16.993 6.629 1.00 0.00 MEMB C

ATOM 1940 H91 POPC 15 -2.380 -16.728 6.271 1.00 0.00 MEMB H

ATOM 1941 C210 POPC 15 -0.399 -17.315 5.814 1.00 0.00 MEMB C

ATOM 1942 H101 POPC 15 -0.420 -16.856 4.845 1.00 0.00 MEMB H

ATOM 1943 C211 POPC 15 0.867 -18.017 6.203 1.00 0.00 MEMB C

ATOM 1944 H11R POPC 15 0.673 -19.054 6.560 1.00 0.00 MEMB H

ATOM 1945 H11S POPC 15 1.381 -17.457 7.013 1.00 0.00 MEMB H

ATOM 1946 C212 POPC 15 1.728 -18.035 4.945 1.00 0.00 MEMB C

ATOM 1947 H12R POPC 15 1.870 -16.979 4.620 1.00 0.00 MEMB H

ATOM 1948 H12S POPC 15 1.186 -18.578 4.135 1.00 0.00 MEMB H

ATOM 1949 C213 POPC 15 3.114 -18.668 5.084 1.00 0.00 MEMB C

ATOM 1950 H13R POPC 15 3.042 -19.719 5.441 1.00 0.00 MEMB H

ATOM 1951 H13S POPC 15 3.737 -18.088 5.802 1.00 0.00 MEMB H

ATOM 1952 C214 POPC 15 3.777 -18.689 3.709 1.00 0.00 MEMB C

ATOM 1953 H14R POPC 15 3.021 -19.022 2.959 1.00 0.00 MEMB H

ATOM 1954 H14S POPC 15 4.572 -19.467 3.718 1.00 0.00 MEMB H

ATOM 1955 C215 POPC 15 4.294 -17.291 3.299 1.00 0.00 MEMB C

ATOM 1956 H15R POPC 15 5.247 -17.108 3.847 1.00 0.00 MEMB H

ATOM 1957 H15S POPC 15 3.563 -16.511 3.557 1.00 0.00 MEMB H

ATOM 1958 C216 POPC 15 4.482 -17.136 1.789 1.00 0.00 MEMB C

ATOM 1959 H16R POPC 15 3.730 -17.694 1.192 1.00 0.00 MEMB H

ATOM 1960 H16S POPC 15 5.452 -17.563 1.755 1.00 0.00 MEMB H

ATOM 1961 C217 POPC 15 5.104 -15.928 1.106 1.00 0.00 MEMB C

ATOM 1962 H17R POPC 15 4.393 -15.105 1.200 1.00 0.00 MEMB H

ATOM 1963 H17S POPC 15 5.253 -16.184 0.023 1.00 0.00 MEMB H

ATOM 1964 C218 POPC 15 6.465 -15.524 1.679 1.00 0.00 MEMB C

ATOM 1965 H18R POPC 15 6.477 -15.608 2.786 1.00 0.00 MEMB H

ATOM 1966 H18S POPC 15 6.649 -14.470 1.399 1.00 0.00 MEMB H

ATOM 1967 H18T POPC 15 7.287 -16.157 1.271 1.00 0.00 MEMB H

ATOM 1968 C33 POPC 15 4.106 -12.126 12.723 1.00 0.00 MEMB C

ATOM 1969 H3X POPC 15 3.232 -12.703 12.338 1.00 0.00 MEMB H

ATOM 1970 H3Y POPC 15 4.983 -12.806 12.707 1.00 0.00 MEMB H

ATOM 1971 C34 POPC 15 4.385 -10.920 11.810 1.00 0.00 MEMB C

ATOM 1972 H4X POPC 15 5.303 -10.409 12.179 1.00 0.00 MEMB H

ATOM 1973 H4Y POPC 15 3.565 -10.173 11.883 1.00 0.00 MEMB H

ATOM 1974 C35 POPC 15 4.595 -11.333 10.340 1.00 0.00 MEMB C

ATOM 1975 H5X POPC 15 5.476 -12.008 10.329 1.00 0.00 MEMB H

ATOM 1976 H5Y POPC 15 4.846 -10.422 9.751 1.00 0.00 MEMB H

ATOM 1977 C36 POPC 15 3.407 -12.044 9.653 1.00 0.00 MEMB C

ATOM 1978 H6X POPC 15 2.597 -11.307 9.595 1.00 0.00 MEMB H

ATOM 1979 H6Y POPC 15 3.008 -12.887 10.253 1.00 0.00 MEMB H

ATOM 1980 C37 POPC 15 3.681 -12.486 8.200 1.00 0.00 MEMB C

ATOM 1981 H7X POPC 15 4.522 -13.196 8.134 1.00 0.00 MEMB H

ATOM 1982 H7Y POPC 15 3.939 -11.562 7.626 1.00 0.00 MEMB H

ATOM 1983 C38 POPC 15 2.511 -13.182 7.506 1.00 0.00 MEMB C

ATOM 1984 H8X POPC 15 1.777 -12.441 7.366 1.00 0.00 MEMB H

ATOM 1985 H8Y POPC 15 1.943 -13.937 8.077 1.00 0.00 MEMB H

ATOM 1986 C39 POPC 15 2.657 -13.387 6.022 1.00 0.00 MEMB C

ATOM 1987 H9X POPC 15 3.124 -14.360 5.795 1.00 0.00 MEMB H

ATOM 1988 H9Y POPC 15 3.213 -12.539 5.545 1.00 0.00 MEMB H

ATOM 1989 C310 POPC 15 1.308 -13.401 5.365 1.00 0.00 MEMB C

ATOM 1990 H10X POPC 15 0.852 -12.355 5.348 1.00 0.00 MEMB H

ATOM 1991 H10Y POPC 15 0.623 -14.182 5.757 1.00 0.00 MEMB H

ATOM 1992 C311 POPC 15 1.557 -13.829 4.000 1.00 0.00 MEMB C

ATOM 1993 H11X POPC 15 2.401 -14.496 3.845 1.00 0.00 MEMB H

ATOM 1994 H11Y POPC 15 1.885 -12.801 3.744 1.00 0.00 MEMB H

ATOM 1995 C312 POPC 15 0.395 -14.387 3.251 1.00 0.00 MEMB C

ATOM 1996 H12X POPC 15 -0.338 -13.635 3.643 1.00 0.00 MEMB H

ATOM 1997 H12Y POPC 15 -0.193 -15.259 3.512 1.00 0.00 MEMB H

ATOM 1998 C313 POPC 15 0.693 -14.560 1.743 1.00 0.00 MEMB C

ATOM 1999 H13X POPC 15 1.070 -13.640 1.351 1.00 0.00 MEMB H

ATOM 2000 H13Y POPC 15 -0.205 -14.658 1.191 1.00 0.00 MEMB H

ATOM 2001 C314 POPC 15 1.790 -15.291 1.050 1.00 0.00 MEMB C

ATOM 2002 H14X POPC 15 2.462 -15.629 1.817 1.00 0.00 MEMB H

ATOM 2003 H14Y POPC 15 2.522 -14.703 0.521 1.00 0.00 MEMB H

ATOM 2004 C315 POPC 15 1.358 -15.763 -0.315 1.00 0.00 MEMB C

ATOM 2005 H15X POPC 15 2.119 -15.732 -1.127 1.00 0.00 MEMB H

ATOM 2006 H15Y POPC 15 0.583 -15.036 -0.649 1.00 0.00 MEMB H

ATOM 2007 C316 POPC 15 0.876 -17.122 -0.003 1.00 0.00 MEMB C

ATOM 2008 H16X POPC 15 1.559 -17.611 0.719 1.00 0.00 MEMB H

ATOM 2009 H16Y POPC 15 0.453 -17.679 -0.851 1.00 0.00 MEMB H

ATOM 2010 H16Z POPC 15 0.051 -17.017 0.670 1.00 0.00 MEMB H

ATOM 2011 N POPC 16 10.278 -10.883 19.469 1.00 0.00 MEMB N

ATOM 2012 C12 POPC 16 11.592 -11.287 18.812 1.00 0.00 MEMB C

ATOM 2013 H12A POPC 16 12.331 -11.384 19.598 1.00 0.00 MEMB H

ATOM 2014 H12B POPC 16 11.899 -10.556 18.087 1.00 0.00 MEMB H

ATOM 2015 C13 POPC 16 10.127 -11.548 20.800 1.00 0.00 MEMB C

ATOM 2016 H13A POPC 16 9.418 -11.011 21.420 1.00 0.00 MEMB H

ATOM 2017 H13B POPC 16 9.801 -12.564 20.665 1.00 0.00 MEMB H

ATOM 2018 H13C POPC 16 11.059 -11.544 21.360 1.00 0.00 MEMB H

ATOM 2019 C14 POPC 16 10.227 -9.486 19.849 1.00 0.00 MEMB C

ATOM 2020 H14A POPC 16 11.229 -9.232 20.169 1.00 0.00 MEMB H

ATOM 2021 H14B POPC 16 9.947 -8.835 19.028 1.00 0.00 MEMB H

ATOM 2022 H14C POPC 16 9.589 -9.277 20.697 1.00 0.00 MEMB H

ATOM 2023 C15 POPC 16 9.158 -11.132 18.505 1.00 0.00 MEMB C

ATOM 2024 H15A POPC 16 9.227 -10.445 17.668 1.00 0.00 MEMB H

ATOM 2025 H15B POPC 16 8.194 -11.032 18.974 1.00 0.00 MEMB H

ATOM 2026 H15C POPC 16 9.245 -12.142 18.102 1.00 0.00 MEMB H

ATOM 2027 C11 POPC 16 11.634 -12.643 18.095 1.00 0.00 MEMB C

ATOM 2028 H11A POPC 16 12.639 -12.791 17.634 1.00 0.00 MEMB H

ATOM 2029 H11B POPC 16 10.884 -12.695 17.268 1.00 0.00 MEMB H

ATOM 2030 P POPC 16 11.216 -15.138 18.679 1.00 0.00 MEMB P

ATOM 2031 O13 POPC 16 9.760 -15.364 18.673 1.00 0.00 MEMB O

ATOM 2032 O14 POPC 16 12.069 -15.952 19.572 1.00 0.00 MEMB O

ATOM 2033 O12 POPC 16 11.430 -13.622 19.092 1.00 0.00 MEMB O

ATOM 2034 O11 POPC 16 11.792 -15.192 17.200 1.00 0.00 MEMB O

ATOM 2035 C1 POPC 16 13.179 -15.535 17.020 1.00 0.00 MEMB C

ATOM 2036 HA POPC 16 13.473 -16.436 17.617 1.00 0.00 MEMB H

ATOM 2037 HB POPC 16 13.830 -14.692 17.326 1.00 0.00 MEMB H

ATOM 2038 C2 POPC 16 13.501 -15.953 15.559 1.00 0.00 MEMB C

ATOM 2039 HS POPC 16 14.590 -16.196 15.500 1.00 0.00 MEMB H

ATOM 2040 O21 POPC 16 13.196 -14.939 14.582 1.00 0.00 MEMB O

ATOM 2041 C21 POPC 16 13.894 -13.837 14.708 1.00 0.00 MEMB C

ATOM 2042 O22 POPC 16 14.820 -13.639 15.475 1.00 0.00 MEMB O

ATOM 2043 C22 POPC 16 13.343 -12.794 13.726 1.00 0.00 MEMB C

ATOM 2044 H2R POPC 16 12.733 -12.077 14.309 1.00 0.00 MEMB H

ATOM 2045 H2S POPC 16 12.669 -13.295 12.999 1.00 0.00 MEMB H

ATOM 2046 C3 POPC 16 12.716 -17.248 15.229 1.00 0.00 MEMB C

ATOM 2047 HX POPC 16 12.953 -18.001 16.013 1.00 0.00 MEMB H

ATOM 2048 HY POPC 16 13.142 -17.611 14.292 1.00 0.00 MEMB H

ATOM 2049 O31 POPC 16 11.296 -17.001 15.204 1.00 0.00 MEMB O

ATOM 2050 C31 POPC 16 10.484 -17.308 14.235 1.00 0.00 MEMB C

ATOM 2051 O32 POPC 16 10.694 -17.996 13.260 1.00 0.00 MEMB O

ATOM 2052 C32 POPC 16 9.121 -16.715 14.514 1.00 0.00 MEMB C

ATOM 2053 H2X POPC 16 9.206 -15.957 15.318 1.00 0.00 MEMB H

ATOM 2054 H2Y POPC 16 8.505 -17.564 14.889 1.00 0.00 MEMB H

ATOM 2055 C23 POPC 16 14.464 -12.036 12.969 1.00 0.00 MEMB C

ATOM 2056 H3R POPC 16 15.120 -11.505 13.696 1.00 0.00 MEMB H

ATOM 2057 H3S POPC 16 13.975 -11.253 12.345 1.00 0.00 MEMB H

ATOM 2058 C24 POPC 16 15.310 -12.972 12.082 1.00 0.00 MEMB C

ATOM 2059 H4R POPC 16 15.948 -13.593 12.748 1.00 0.00 MEMB H

ATOM 2060 H4S POPC 16 16.062 -12.483 11.461 1.00 0.00 MEMB H

ATOM 2061 C25 POPC 16 14.500 -13.800 11.067 1.00 0.00 MEMB C

ATOM 2062 H5R POPC 16 13.885 -14.477 11.680 1.00 0.00 MEMB H

ATOM 2063 H5S POPC 16 15.205 -14.407 10.452 1.00 0.00 MEMB H

ATOM 2064 C26 POPC 16 13.510 -13.047 10.137 1.00 0.00 MEMB C

ATOM 2065 H6R POPC 16 12.917 -12.312 10.719 1.00 0.00 MEMB H

ATOM 2066 H6S POPC 16 12.874 -13.775 9.596 1.00 0.00 MEMB H

ATOM 2067 C27 POPC 16 14.181 -12.271 9.036 1.00 0.00 MEMB C

ATOM 2068 H7R POPC 16 14.603 -12.978 8.284 1.00 0.00 MEMB H

ATOM 2069 H7S POPC 16 14.993 -11.898 9.677 1.00 0.00 MEMB H

ATOM 2070 C28 POPC 16 13.394 -11.143 8.290 1.00 0.00 MEMB C

ATOM 2071 H8R POPC 16 12.582 -11.672 7.760 1.00 0.00 MEMB H

ATOM 2072 H8S POPC 16 13.998 -10.737 7.462 1.00 0.00 MEMB H

ATOM 2073 C29 POPC 16 12.927 -9.920 9.069 1.00 0.00 MEMB C

ATOM 2074 H91 POPC 16 13.635 -9.092 9.243 1.00 0.00 MEMB H

ATOM 2075 C210 POPC 16 11.773 -9.790 9.683 1.00 0.00 MEMB C

ATOM 2076 H101 POPC 16 11.602 -8.892 10.294 1.00 0.00 MEMB H

ATOM 2077 C211 POPC 16 10.744 -10.862 9.695 1.00 0.00 MEMB C

ATOM 2078 H11R POPC 16 10.363 -10.937 10.733 1.00 0.00 MEMB H

ATOM 2079 H11S POPC 16 11.155 -11.863 9.431 1.00 0.00 MEMB H

ATOM 2080 C212 POPC 16 9.576 -10.528 8.772 1.00 0.00 MEMB C

ATOM 2081 H12R POPC 16 9.160 -9.534 9.043 1.00 0.00 MEMB H

ATOM 2082 H12S POPC 16 8.811 -11.306 8.992 1.00 0.00 MEMB H

ATOM 2083 C213 POPC 16 9.837 -10.571 7.256 1.00 0.00 MEMB C

ATOM 2084 H13R POPC 16 10.488 -11.438 7.003 1.00 0.00 MEMB H

ATOM 2085 H13S POPC 16 10.351 -9.642 6.926 1.00 0.00 MEMB H

ATOM 2086 C214 POPC 16 8.491 -10.743 6.537 1.00 0.00 MEMB C

ATOM 2087 H14R POPC 16 7.867 -9.834 6.676 1.00 0.00 MEMB H

ATOM 2088 H14S POPC 16 7.963 -11.584 7.046 1.00 0.00 MEMB H

ATOM 2089 C215 POPC 16 8.575 -11.045 5.039 1.00 0.00 MEMB C

ATOM 2090 H15R POPC 16 7.541 -11.200 4.652 1.00 0.00 MEMB H

ATOM 2091 H15S POPC 16 9.134 -11.995 4.880 1.00 0.00 MEMB H

ATOM 2092 C216 POPC 16 9.258 -9.963 4.191 1.00 0.00 MEMB C

ATOM 2093 H16R POPC 16 10.289 -9.788 4.566 1.00 0.00 MEMB H

ATOM 2094 H16S POPC 16 8.721 -8.994 4.267 1.00 0.00 MEMB H

ATOM 2095 C217 POPC 16 9.347 -10.471 2.747 1.00 0.00 MEMB C

ATOM 2096 H17R POPC 16 8.320 -10.555 2.324 1.00 0.00 MEMB H

ATOM 2097 H17S POPC 16 9.768 -11.500 2.801 1.00 0.00 MEMB H

ATOM 2098 C218 POPC 16 10.263 -9.679 1.823 1.00 0.00 MEMB C

ATOM 2099 H18R POPC 16 9.834 -8.710 1.567 1.00 0.00 MEMB H

ATOM 2100 H18S POPC 16 10.469 -10.224 0.878 1.00 0.00 MEMB H

ATOM 2101 H18T POPC 16 11.211 -9.413 2.326 1.00 0.00 MEMB H

ATOM 2102 C33 POPC 16 8.513 -16.153 13.201 1.00 0.00 MEMB C

ATOM 2103 H3X POPC 16 7.503 -15.765 13.453 1.00 0.00 MEMB H

ATOM 2104 H3Y POPC 16 8.384 -16.991 12.478 1.00 0.00 MEMB H

ATOM 2105 C34 POPC 16 9.299 -14.995 12.547 1.00 0.00 MEMB C

ATOM 2106 H4X POPC 16 10.352 -15.270 12.318 1.00 0.00 MEMB H

ATOM 2107 H4Y POPC 16 9.335 -14.167 13.289 1.00 0.00 MEMB H

ATOM 2108 C35 POPC 16 8.613 -14.438 11.283 1.00 0.00 MEMB C

ATOM 2109 H5X POPC 16 8.958 -13.393 11.108 1.00 0.00 MEMB H

ATOM 2110 H5Y POPC 16 7.518 -14.400 11.470 1.00 0.00 MEMB H

ATOM 2111 C36 POPC 16 8.828 -15.260 10.008 1.00 0.00 MEMB C

ATOM 2112 H6X POPC 16 8.191 -14.838 9.197 1.00 0.00 MEMB H

ATOM 2113 H6Y POPC 16 8.504 -16.313 10.169 1.00 0.00 MEMB H

ATOM 2114 C37 POPC 16 10.259 -15.275 9.497 1.00 0.00 MEMB C

ATOM 2115 H7X POPC 16 10.989 -15.481 10.305 1.00 0.00 MEMB H

ATOM 2116 H7Y POPC 16 10.527 -14.285 9.062 1.00 0.00 MEMB H

ATOM 2117 C38 POPC 16 10.408 -16.320 8.419 1.00 0.00 MEMB C

ATOM 2118 H8X POPC 16 9.719 -16.130 7.567 1.00 0.00 MEMB H

ATOM 2119 H8Y POPC 16 10.283 -17.375 8.773 1.00 0.00 MEMB H

ATOM 2120 C39 POPC 16 11.753 -16.204 7.814 1.00 0.00 MEMB C

ATOM 2121 H9X POPC 16 12.435 -16.560 8.586 1.00 0.00 MEMB H

ATOM 2122 H9Y POPC 16 12.132 -15.178 7.687 1.00 0.00 MEMB H

ATOM 2123 C310 POPC 16 11.648 -16.930 6.499 1.00 0.00 MEMB C

ATOM 2124 H10X POPC 16 11.008 -16.473 5.729 1.00 0.00 MEMB H

ATOM 2125 H10Y POPC 16 11.334 -18.009 6.624 1.00 0.00 MEMB H

ATOM 2126 C311 POPC 16 12.950 -16.851 5.830 1.00 0.00 MEMB C

ATOM 2127 H11X POPC 16 12.892 -17.260 4.837 1.00 0.00 MEMB H

ATOM 2128 H11Y POPC 16 13.223 -17.792 6.334 1.00 0.00 MEMB H

ATOM 2129 C312 POPC 16 13.881 -15.567 6.023 1.00 0.00 MEMB C

ATOM 2130 H12X POPC 16 14.939 -15.547 5.669 1.00 0.00 MEMB H

ATOM 2131 H12Y POPC 16 14.113 -15.277 7.066 1.00 0.00 MEMB H

ATOM 2132 C313 POPC 16 13.404 -14.429 5.329 1.00 0.00 MEMB C

ATOM 2133 H13X POPC 16 14.034 -13.588 5.722 1.00 0.00 MEMB H

ATOM 2134 H13Y POPC 16 12.348 -14.391 5.665 1.00 0.00 MEMB H

ATOM 2135 C314 POPC 16 13.618 -14.674 3.859 1.00 0.00 MEMB C

ATOM 2136 H14X POPC 16 12.905 -15.447 3.493 1.00 0.00 MEMB H

ATOM 2137 H14Y POPC 16 14.649 -15.027 3.604 1.00 0.00 MEMB H

ATOM 2138 C315 POPC 16 13.335 -13.310 3.324 1.00 0.00 MEMB C

ATOM 2139 H15X POPC 16 14.182 -12.646 3.620 1.00 0.00 MEMB H

ATOM 2140 H15Y POPC 16 12.424 -12.979 3.882 1.00 0.00 MEMB H

ATOM 2141 C316 POPC 16 13.056 -13.285 1.847 1.00 0.00 MEMB C

ATOM 2142 H16X POPC 16 12.228 -13.986 1.605 1.00 0.00 MEMB H

ATOM 2143 H16Y POPC 16 13.958 -13.586 1.278 1.00 0.00 MEMB H

ATOM 2144 H16Z POPC 16 12.742 -12.257 1.573 1.00 0.00 MEMB H

ATOM 2145 N POPC 17 -17.604 -8.789 17.839 1.00 0.00 MEMB N

ATOM 2146 C12 POPC 17 -17.135 -7.401 17.550 1.00 0.00 MEMB C

ATOM 2147 H12A POPC 17 -17.969 -6.719 17.671 1.00 0.00 MEMB H

ATOM 2148 H12B POPC 17 -16.774 -7.348 16.523 1.00 0.00 MEMB H

ATOM 2149 C13 POPC 17 -18.236 -9.112 19.140 1.00 0.00 MEMB C

ATOM 2150 H13A POPC 17 -18.115 -10.180 19.353 1.00 0.00 MEMB H

ATOM 2151 H13B POPC 17 -17.664 -8.591 19.908 1.00 0.00 MEMB H

ATOM 2152 H13C POPC 17 -19.264 -8.833 19.175 1.00 0.00 MEMB H

ATOM 2153 C14 POPC 17 -18.479 -9.280 16.725 1.00 0.00 MEMB C

ATOM 2154 H14A POPC 17 -19.382 -8.682 16.716 1.00 0.00 MEMB H

ATOM 2155 H14B POPC 17 -18.023 -9.232 15.737 1.00 0.00 MEMB H

ATOM 2156 H14C POPC 17 -18.766 -10.314 16.883 1.00 0.00 MEMB H

ATOM 2157 C15 POPC 17 -16.385 -9.480 18.142 1.00 0.00 MEMB C

ATOM 2158 H15A POPC 17 -15.650 -9.335 17.333 1.00 0.00 MEMB H

ATOM 2159 H15B POPC 17 -16.636 -10.536 18.133 1.00 0.00 MEMB H

ATOM 2160 H15C POPC 17 -15.878 -9.312 19.080 1.00 0.00 MEMB H

ATOM 2161 C11 POPC 17 -15.975 -6.907 18.435 1.00 0.00 MEMB C

ATOM 2162 H11A POPC 17 -15.748 -5.881 18.057 1.00 0.00 MEMB H

ATOM 2163 H11B POPC 17 -15.019 -7.465 18.308 1.00 0.00 MEMB H

ATOM 2164 P POPC 17 -17.185 -5.767 20.420 1.00 0.00 MEMB P

ATOM 2165 O13 POPC 17 -16.144 -4.807 20.830 1.00 0.00 MEMB O

ATOM 2166 O14 POPC 17 -18.176 -6.256 21.403 1.00 0.00 MEMB O

ATOM 2167 O12 POPC 17 -16.365 -6.971 19.815 1.00 0.00 MEMB O

ATOM 2168 O11 POPC 17 -17.920 -5.242 19.167 1.00 0.00 MEMB O

ATOM 2169 C1 POPC 17 -19.260 -4.896 19.445 1.00 0.00 MEMB C

ATOM 2170 HA POPC 17 -19.390 -4.141 20.265 1.00 0.00 MEMB H

ATOM 2171 HB POPC 17 -19.906 -5.777 19.697 1.00 0.00 MEMB H

ATOM 2172 C2 POPC 17 -19.803 -4.274 18.199 1.00 0.00 MEMB C

ATOM 2173 HS POPC 17 -20.848 -4.509 18.301 1.00 0.00 MEMB H

ATOM 2174 O21 POPC 17 -19.318 -4.868 16.992 1.00 0.00 MEMB O

ATOM 2175 C21 POPC 17 -20.039 -5.916 16.721 1.00 0.00 MEMB C

ATOM 2176 O22 POPC 17 -20.778 -6.552 17.458 1.00 0.00 MEMB O

ATOM 2177 C22 POPC 17 -19.880 -6.188 15.284 1.00 0.00 MEMB C

ATOM 2178 H2R POPC 17 -19.080 -6.955 15.203 1.00 0.00 MEMB H

ATOM 2179 H2S POPC 17 -19.521 -5.305 14.731 1.00 0.00 MEMB H

ATOM 2180 C3 POPC 17 -19.497 -2.743 18.224 1.00 0.00 MEMB C

ATOM 2181 HX POPC 17 -19.884 -2.341 19.174 1.00 0.00 MEMB H

ATOM 2182 HY POPC 17 -20.045 -2.154 17.468 1.00 0.00 MEMB H

ATOM 2183 O31 POPC 17 -18.100 -2.483 18.232 1.00 0.00 MEMB O

ATOM 2184 C31 POPC 17 -17.590 -2.447 17.044 1.00 0.00 MEMB C

ATOM 2185 O32 POPC 17 -18.203 -2.329 15.997 1.00 0.00 MEMB O

ATOM 2186 C32 POPC 17 -16.094 -2.489 17.196 1.00 0.00 MEMB C

ATOM 2187 H2X POPC 17 -15.818 -3.165 18.004 1.00 0.00 MEMB H

ATOM 2188 H2Y POPC 17 -15.721 -1.516 17.566 1.00 0.00 MEMB H

ATOM 2189 C23 POPC 17 -21.187 -6.829 14.795 1.00 0.00 MEMB C

ATOM 2190 H3R POPC 17 -21.430 -7.552 15.564 1.00 0.00 MEMB H

ATOM 2191 H3S POPC 17 -20.888 -7.117 13.766 1.00 0.00 MEMB H

ATOM 2192 C24 POPC 17 -22.564 -6.282 14.470 1.00 0.00 MEMB C

ATOM 2193 H4R POPC 17 -22.921 -5.831 15.425 1.00 0.00 MEMB H

ATOM 2194 H4S POPC 17 -23.438 -6.875 14.141 1.00 0.00 MEMB H

ATOM 2195 C25 POPC 17 -22.063 -5.388 13.435 1.00 0.00 MEMB C

ATOM 2196 H5R POPC 17 -21.031 -5.046 13.639 1.00 0.00 MEMB H

ATOM 2197 H5S POPC 17 -22.999 -4.840 13.750 1.00 0.00 MEMB H

ATOM 2198 C26 POPC 17 -22.068 -4.817 12.116 1.00 0.00 MEMB C

ATOM 2199 H6R POPC 17 -21.014 -5.175 11.859 1.00 0.00 MEMB H

ATOM 2200 H6S POPC 17 -21.790 -3.805 11.769 1.00 0.00 MEMB H

ATOM 2201 C27 POPC 17 -23.366 -5.085 11.426 1.00 0.00 MEMB C

ATOM 2202 H7R POPC 17 -23.724 -4.116 11.175 1.00 0.00 MEMB H

ATOM 2203 H7S POPC 17 -24.079 -5.680 12.102 1.00 0.00 MEMB H

ATOM 2204 C28 POPC 17 -23.183 -5.449 9.794 1.00 0.00 MEMB C

ATOM 2205 H8R POPC 17 -22.562 -4.607 9.570 1.00 0.00 MEMB H

ATOM 2206 H8S POPC 17 -24.128 -5.763 9.483 1.00 0.00 MEMB H

ATOM 2207 C29 POPC 17 -22.023 -5.580 10.592 1.00 0.00 MEMB C

ATOM 2208 H91 POPC 17 -22.801 -6.824 10.942 1.00 0.00 MEMB H

ATOM 2209 C210 POPC 17 -20.805 -6.923 9.902 1.00 0.00 MEMB C

ATOM 2210 H101 POPC 17 -20.430 -7.618 10.274 1.00 0.00 MEMB H

ATOM 2211 C211 POPC 17 -20.275 -6.457 8.511 1.00 0.00 MEMB C

ATOM 2212 H11R POPC 17 -19.255 -6.367 9.057 1.00 0.00 MEMB H

ATOM 2213 H11S POPC 17 -20.851 -5.912 8.075 1.00 0.00 MEMB H

ATOM 2214 C212 POPC 17 -19.905 -7.786 7.605 1.00 0.00 MEMB C

ATOM 2215 H12R POPC 17 -19.864 -8.797 8.064 1.00 0.00 MEMB H

ATOM 2216 H12S POPC 17 -18.984 -7.545 7.058 1.00 0.00 MEMB H

ATOM 2217 C213 POPC 17 -20.908 -7.798 6.502 1.00 0.00 MEMB C

ATOM 2218 H13R POPC 17 -21.104 -6.802 6.060 1.00 0.00 MEMB H

ATOM 2219 H13S POPC 17 -21.839 -7.855 7.091 1.00 0.00 MEMB H

ATOM 2220 C214 POPC 17 -20.634 -8.839 5.362 1.00 0.00 MEMB C

ATOM 2221 H14R POPC 17 -19.872 -8.446 4.675 1.00 0.00 MEMB H

ATOM 2222 H14S POPC 17 -21.495 -8.970 4.743 1.00 0.00 MEMB H

ATOM 2223 C215 POPC 17 -20.404 -10.333 5.615 1.00 0.00 MEMB C

ATOM 2224 H15R POPC 17 -21.198 -10.956 6.062 1.00 0.00 MEMB H

ATOM 2225 H15S POPC 17 -19.622 -10.259 6.366 1.00 0.00 MEMB H

ATOM 2226 C216 POPC 17 -19.994 -11.150 4.383 1.00 0.00 MEMB C

ATOM 2227 H16R POPC 17 -19.684 -12.158 4.691 1.00 0.00 MEMB H

ATOM 2228 H16S POPC 17 -19.148 -10.598 3.925 1.00 0.00 MEMB H

ATOM 2229 C217 POPC 17 -20.988 -11.541 3.294 1.00 0.00 MEMB C

ATOM 2230 H17R POPC 17 -21.348 -10.579 3.006 1.00 0.00 MEMB H

ATOM 2231 H17S POPC 17 -21.877 -12.125 3.606 1.00 0.00 MEMB H

ATOM 2232 C218 POPC 17 -20.284 -12.188 2.094 1.00 0.00 MEMB C

ATOM 2233 H18R POPC 17 -19.863 -13.161 2.393 1.00 0.00 MEMB H

ATOM 2234 H18S POPC 17 -19.443 -11.529 1.773 1.00 0.00 MEMB H

ATOM 2235 H18T POPC 17 -20.953 -12.326 1.217 1.00 0.00 MEMB H

ATOM 2236 C33 POPC 17 -15.483 -2.791 15.835 1.00 0.00 MEMB C

ATOM 2237 H3X POPC 17 -14.431 -2.432 15.887 1.00 0.00 MEMB H

ATOM 2238 H3Y POPC 17 -16.023 -2.271 15.001 1.00 0.00 MEMB H

ATOM 2239 C34 POPC 17 -15.385 -4.267 15.543 1.00 0.00 MEMB C

ATOM 2240 H4X POPC 17 -16.411 -4.690 15.532 1.00 0.00 MEMB H

ATOM 2241 H4Y POPC 17 -14.809 -4.662 16.410 1.00 0.00 MEMB H

ATOM 2242 C35 POPC 17 -14.635 -4.604 14.242 1.00 0.00 MEMB C

ATOM 2243 H5X POPC 17 -14.479 -5.704 14.229 1.00 0.00 MEMB H

ATOM 2244 H5Y POPC 17 -13.630 -4.124 14.272 1.00 0.00 MEMB H

ATOM 2245 C36 POPC 17 -15.311 -4.130 12.932 1.00 0.00 MEMB C

ATOM 2246 H6X POPC 17 -14.581 -4.234 12.101 1.00 0.00 MEMB H

ATOM 2247 H6Y POPC 17 -15.553 -3.047 13.016 1.00 0.00 MEMB H

ATOM 2248 C37 POPC 17 -16.585 -4.888 12.529 1.00 0.00 MEMB C

ATOM 2249 H7X POPC 17 -17.416 -4.504 13.162 1.00 0.00 MEMB H

ATOM 2250 H7Y POPC 17 -16.454 -5.961 12.793 1.00 0.00 MEMB H

ATOM 2251 C38 POPC 17 -16.927 -4.802 11.011 1.00 0.00 MEMB C

ATOM 2252 H8X POPC 17 -17.923 -5.288 10.898 1.00 0.00 MEMB H

ATOM 2253 H8Y POPC 17 -16.197 -5.388 10.417 1.00 0.00 MEMB H

ATOM 2254 C39 POPC 17 -16.978 -3.380 10.401 1.00 0.00 MEMB C

ATOM 2255 H9X POPC 17 -16.046 -2.800 10.508 1.00 0.00 MEMB H

ATOM 2256 H9Y POPC 17 -17.713 -2.832 11.035 1.00 0.00 MEMB H

ATOM 2257 C310 POPC 17 -17.354 -3.264 8.903 1.00 0.00 MEMB C

ATOM 2258 H10X POPC 17 -17.648 -2.241 8.620 1.00 0.00 MEMB H

ATOM 2259 H10Y POPC 17 -18.176 -3.931 8.664 1.00 0.00 MEMB H

ATOM 2260 C311 POPC 17 -16.697 -4.008 7.784 1.00 0.00 MEMB C

ATOM 2261 H11X POPC 17 -16.874 -5.111 7.935 1.00 0.00 MEMB H

ATOM 2262 H11Y POPC 17 -15.629 -3.771 7.817 1.00 0.00 MEMB H

ATOM 2263 C312 POPC 17 -17.355 -3.584 6.459 1.00 0.00 MEMB C

ATOM 2264 H12X POPC 17 -17.008 -2.573 6.176 1.00 0.00 MEMB H

ATOM 2265 H12Y POPC 17 -18.472 -3.513 6.451 1.00 0.00 MEMB H

ATOM 2266 C313 POPC 17 -16.935 -4.574 5.412 1.00 0.00 MEMB C

ATOM 2267 H13X POPC 17 -17.475 -5.536 5.611 1.00 0.00 MEMB H

ATOM 2268 H13Y POPC 17 -15.841 -4.746 5.448 1.00 0.00 MEMB H

ATOM 2269 C314 POPC 17 -17.231 -4.043 4.044 1.00 0.00 MEMB C

ATOM 2270 H14X POPC 17 -16.891 -3.036 3.771 1.00 0.00 MEMB H

ATOM 2271 H14Y POPC 17 -18.348 -3.960 4.037 1.00 0.00 MEMB H

ATOM 2272 C315 POPC 17 -16.553 -4.918 3.008 1.00 0.00 MEMB C

ATOM 2273 H15X POPC 17 -16.745 -5.984 3.302 1.00 0.00 MEMB H

ATOM 2274 H15Y POPC 17 -15.468 -5.013 2.944 1.00 0.00 MEMB H

ATOM 2275 C316 POPC 17 -17.059 -4.481 1.639 1.00 0.00 MEMB C

ATOM 2276 H16X POPC 17 -16.559 -5.192 0.949 1.00 0.00 MEMB H

ATOM 2277 H16Y POPC 17 -16.961 -3.493 1.237 1.00 0.00 MEMB H

ATOM 2278 H16Z POPC 17 -18.171 -4.543 1.525 1.00 0.00 MEMB H

ATOM 2279 N POPC 18 -5.813 -17.661 19.727 1.00 0.00 MEMB N

ATOM 2280 C12 POPC 18 -4.602 -17.861 18.832 1.00 0.00 MEMB C

ATOM 2281 H12A POPC 18 -3.754 -18.138 19.457 1.00 0.00 MEMB H

ATOM 2282 H12B POPC 18 -4.335 -16.919 18.358 1.00 0.00 MEMB H

ATOM 2283 C13 POPC 18 -7.055 -17.666 18.948 1.00 0.00 MEMB C

ATOM 2284 H13A POPC 18 -7.907 -17.581 19.626 1.00 0.00 MEMB H

ATOM 2285 H13B POPC 18 -7.120 -16.899 18.191 1.00 0.00 MEMB H

ATOM 2286 H13C POPC 18 -7.216 -18.630 18.458 1.00 0.00 MEMB H

ATOM 2287 C14 POPC 18 -5.912 -18.793 20.715 1.00 0.00 MEMB C

ATOM 2288 H14A POPC 18 -4.955 -18.964 21.201 1.00 0.00 MEMB H

ATOM 2289 H14B POPC 18 -6.680 -18.657 21.471 1.00 0.00 MEMB H

ATOM 2290 H14C POPC 18 -6.104 -19.737 20.198 1.00 0.00 MEMB H

ATOM 2291 C15 POPC 18 -5.693 -16.354 20.405 1.00 0.00 MEMB C

ATOM 2292 H15A POPC 18 -6.572 -16.174 21.022 1.00 0.00 MEMB H

ATOM 2293 H15B POPC 18 -5.595 -15.584 19.656 1.00 0.00 MEMB H

ATOM 2294 H15C POPC 18 -4.839 -16.335 21.066 1.00 0.00 MEMB H

ATOM 2295 C11 POPC 18 -4.712 -18.919 17.712 1.00 0.00 MEMB C

ATOM 2296 H11A POPC 18 -3.721 -18.930 17.203 1.00 0.00 MEMB H

ATOM 2297 H11B POPC 18 -5.438 -18.566 16.950 1.00 0.00 MEMB H

ATOM 2298 P POPC 18 -4.052 -21.325 18.587 1.00 0.00 MEMB P

ATOM 2299 O13 POPC 18 -4.759 -22.627 18.687 1.00 0.00 MEMB O

ATOM 2300 O14 POPC 18 -3.304 -20.794 19.754 1.00 0.00 MEMB O

ATOM 2301 O12 POPC 18 -5.125 -20.205 18.189 1.00 0.00 MEMB O

ATOM 2302 O11 POPC 18 -3.215 -21.312 17.230 1.00 0.00 MEMB O

ATOM 2303 C1 POPC 18 -1.945 -20.666 17.070 1.00 0.00 MEMB C

ATOM 2304 HA POPC 18 -1.126 -21.346 17.268 1.00 0.00 MEMB H

ATOM 2305 HB POPC 18 -1.801 -19.809 17.753 1.00 0.00 MEMB H

ATOM 2306 C2 POPC 18 -1.533 -20.333 15.626 1.00 0.00 MEMB C

ATOM 2307 HS POPC 18 -0.450 -20.054 15.668 1.00 0.00 MEMB H

ATOM 2308 O21 POPC 18 -2.343 -19.248 15.184 1.00 0.00 MEMB O

ATOM 2309 C21 POPC 18 -1.936 -18.665 14.099 1.00 0.00 MEMB C

ATOM 2310 O22 POPC 18 -0.874 -18.828 13.521 1.00 0.00 MEMB O

ATOM 2311 C22 POPC 18 -3.077 -17.773 13.663 1.00 0.00 MEMB C

ATOM 2312 H2R POPC 18 -2.804 -16.735 13.935 1.00 0.00 MEMB H

ATOM 2313 H2S POPC 18 -4.013 -18.065 14.192 1.00 0.00 MEMB H

ATOM 2314 C3 POPC 18 -1.666 -21.560 14.689 1.00 0.00 MEMB C

ATOM 2315 HX POPC 18 -1.154 -22.416 15.179 1.00 0.00 MEMB H

ATOM 2316 HY POPC 18 -1.150 -21.333 13.728 1.00 0.00 MEMB H

ATOM 2317 O31 POPC 18 -3.038 -21.923 14.529 1.00 0.00 MEMB O

ATOM 2318 C31 POPC 18 -3.617 -21.699 13.391 1.00 0.00 MEMB C

ATOM 2319 O32 POPC 18 -3.082 -21.314 12.361 1.00 0.00 MEMB O

ATOM 2320 C32 POPC 18 -5.082 -21.997 13.579 1.00 0.00 MEMB C

ATOM 2321 H2X POPC 18 -5.419 -21.506 14.524 1.00 0.00 MEMB H

ATOM 2322 H2Y POPC 18 -5.200 -23.092 13.705 1.00 0.00 MEMB H

ATOM 2323 C23 POPC 18 -3.284 -17.966 12.164 1.00 0.00 MEMB C

ATOM 2324 H3R POPC 18 -3.788 -18.944 12.000 1.00 0.00 MEMB H

ATOM 2325 H3S POPC 18 -2.289 -18.026 11.666 1.00 0.00 MEMB H

ATOM 2326 C24 POPC 18 -4.097 -16.838 11.525 1.00 0.00 MEMB C

ATOM 2327 H4R POPC 18 -3.568 -15.879 11.725 1.00 0.00 MEMB H

ATOM 2328 H4S POPC 18 -5.108 -16.791 11.964 1.00 0.00 MEMB H

ATOM 2329 C25 POPC 18 -4.173 -16.996 10.010 1.00 0.00 MEMB C

ATOM 2330 H5R POPC 18 -3.196 -17.434 9.813 1.00 0.00 MEMB H

ATOM 2331 H5S POPC 18 -4.292 -16.038 9.463 1.00 0.00 MEMB H

ATOM 2332 C26 POPC 18 -4.965 -18.139 9.409 1.00 0.00 MEMB C

ATOM 2333 H6R POPC 18 -6.033 -17.859 9.414 1.00 0.00 MEMB H

ATOM 2334 H6S POPC 18 -4.826 -19.012 10.084 1.00 0.00 MEMB H

ATOM 2335 C27 POPC 18 -4.502 -18.465 7.956 1.00 0.00 MEMB C

ATOM 2336 H7R POPC 18 -4.346 -17.525 7.391 1.00 0.00 MEMB H

ATOM 2337 H7S POPC 18 -5.351 -18.959 7.441 1.00 0.00 MEMB H

ATOM 2338 C28 POPC 18 -3.280 -19.403 7.804 1.00 0.00 MEMB C

ATOM 2339 H8R POPC 18 -2.424 -19.290 8.489 1.00 0.00 MEMB H

ATOM 2340 H8S POPC 18 -2.895 -19.447 6.763 1.00 0.00 MEMB H

ATOM 2341 C29 POPC 18 -3.806 -20.606 8.361 1.00 0.00 MEMB C

ATOM 2342 H91 POPC 18 -3.484 -20.720 9.426 1.00 0.00 MEMB H

ATOM 2343 C210 POPC 18 -4.209 -21.647 7.655 1.00 0.00 MEMB C

ATOM 2344 H101 POPC 18 -4.247 -22.447 8.388 1.00 0.00 MEMB H

ATOM 2345 C211 POPC 18 -4.257 -22.003 6.115 1.00 0.00 MEMB C

ATOM 2346 H11R POPC 18 -3.805 -21.116 5.622 1.00 0.00 MEMB H

ATOM 2347 H11S POPC 18 -5.288 -21.914 5.802 1.00 0.00 MEMB H

ATOM 2348 C212 POPC 18 -3.778 -23.465 5.443 1.00 0.00 MEMB C

ATOM 2349 H12R POPC 18 -4.020 -24.285 6.051 1.00 0.00 MEMB H

ATOM 2350 H12S POPC 18 -2.748 -23.829 5.221 1.00 0.00 MEMB H

ATOM 2351 C213 POPC 18 -4.201 -24.189 4.317 1.00 0.00 MEMB C

ATOM 2352 H13R POPC 18 -5.247 -24.444 4.480 1.00 0.00 MEMB H

ATOM 2353 H13S POPC 18 -3.569 -25.147 4.182 1.00 0.00 MEMB H

ATOM 2354 C214 POPC 18 -3.797 -23.124 3.421 1.00 0.00 MEMB C

ATOM 2355 H14R POPC 18 -2.684 -23.107 3.268 1.00 0.00 MEMB H

ATOM 2356 H14S POPC 18 -4.230 -22.136 3.652 1.00 0.00 MEMB H

ATOM 2357 C215 POPC 18 -4.297 -23.521 2.241 1.00 0.00 MEMB C

ATOM 2358 H15R POPC 18 -5.287 -23.837 2.187 1.00 0.00 MEMB H

ATOM 2359 H15S POPC 18 -3.877 -24.541 2.360 1.00 0.00 MEMB H

ATOM 2360 C216 POPC 18 -3.644 -22.704 1.186 1.00 0.00 MEMB C

ATOM 2361 H16R POPC 18 -4.166 -23.060 0.297 1.00 0.00 MEMB H

ATOM 2362 H16S POPC 18 -2.584 -22.782 0.985 1.00 0.00 MEMB H

ATOM 2363 C217 POPC 18 -4.149 -21.656 0.527 1.00 0.00 MEMB C

ATOM 2364 H17R POPC 18 -3.433 -20.996 1.063 1.00 0.00 MEMB H

ATOM 2365 H17S POPC 18 -5.260 -21.829 0.788 1.00 0.00 MEMB H

ATOM 2366 C218 POPC 18 -3.811 -21.565 -0.913 1.00 0.00 MEMB C

ATOM 2367 H18R POPC 18 -2.798 -21.265 -1.192 1.00 0.00 MEMB H

ATOM 2368 H18S POPC 18 -4.259 -20.658 -0.921 1.00 0.00 MEMB H

ATOM 2369 H18T POPC 18 -4.452 -22.160 -1.592 1.00 0.00 MEMB H

ATOM 2370 C33 POPC 18 -5.937 -21.511 12.406 1.00 0.00 MEMB C

ATOM 2371 H3X POPC 18 -5.832 -20.420 12.198 1.00 0.00 MEMB H

ATOM 2372 H3Y POPC 18 -6.924 -21.797 12.797 1.00 0.00 MEMB H

ATOM 2373 C34 POPC 18 -6.051 -22.382 11.172 1.00 0.00 MEMB C

ATOM 2374 H4X POPC 18 -6.283 -23.409 11.548 1.00 0.00 MEMB H

ATOM 2375 H4Y POPC 18 -5.084 -22.338 10.644 1.00 0.00 MEMB H

ATOM 2376 C35 POPC 18 -7.193 -21.985 10.220 1.00 0.00 MEMB C

ATOM 2377 H5X POPC 18 -8.160 -22.237 10.663 1.00 0.00 MEMB H

ATOM 2378 H5Y POPC 18 -7.187 -22.692 9.377 1.00 0.00 MEMB H

ATOM 2379 C36 POPC 18 -7.112 -20.520 9.734 1.00 0.00 MEMB C

ATOM 2380 H6X POPC 18 -6.197 -20.443 9.132 1.00 0.00 MEMB H

ATOM 2381 H6Y POPC 18 -7.069 -19.806 10.587 1.00 0.00 MEMB H

ATOM 2382 C37 POPC 18 -8.234 -20.027 8.828 1.00 0.00 MEMB C

ATOM 2383 H7X POPC 18 -8.206 -18.943 8.597 1.00 0.00 MEMB H

ATOM 2384 H7Y POPC 18 -9.102 -20.159 9.481 1.00 0.00 MEMB H

ATOM 2385 C38 POPC 18 -8.364 -20.862 7.548 1.00 0.00 MEMB C

ATOM 2386 H8X POPC 18 -9.344 -21.073 7.092 1.00 0.00 MEMB H

ATOM 2387 H8Y POPC 18 -8.184 -21.833 7.922 1.00 0.00 MEMB H

ATOM 2388 C39 POPC 18 -7.442 -20.494 6.417 1.00 0.00 MEMB C

ATOM 2389 H9X POPC 18 -7.456 -21.283 5.655 1.00 0.00 MEMB H

ATOM 2390 H9Y POPC 18 -6.425 -20.537 6.823 1.00 0.00 MEMB H

ATOM 2391 C310 POPC 18 -7.788 -19.145 5.791 1.00 0.00 MEMB C

ATOM 2392 H10X POPC 18 -7.614 -18.344 6.520 1.00 0.00 MEMB H

ATOM 2393 H10Y POPC 18 -8.802 -18.926 5.529 1.00 0.00 MEMB H

ATOM 2394 C311 POPC 18 -7.174 -18.908 4.451 1.00 0.00 MEMB C

ATOM 2395 H11X POPC 18 -7.864 -18.439 3.713 1.00 0.00 MEMB H

ATOM 2396 H11Y POPC 18 -6.915 -19.905 4.067 1.00 0.00 MEMB H

ATOM 2397 C312 POPC 18 -5.976 -18.087 4.788 1.00 0.00 MEMB C

ATOM 2398 H12X POPC 18 -5.288 -18.820 5.297 1.00 0.00 MEMB H

ATOM 2399 H12Y POPC 18 -6.208 -17.209 5.400 1.00 0.00 MEMB H

ATOM 2400 C313 POPC 18 -5.208 -17.520 3.688 1.00 0.00 MEMB C

ATOM 2401 H13X POPC 18 -5.527 -16.608 3.145 1.00 0.00 MEMB H

ATOM 2402 H13Y POPC 18 -5.561 -17.988 2.841 1.00 0.00 MEMB H

ATOM 2403 C314 POPC 18 -3.777 -18.046 3.952 1.00 0.00 MEMB C

ATOM 2404 H14X POPC 18 -3.541 -19.136 3.996 1.00 0.00 MEMB H

ATOM 2405 H14Y POPC 18 -3.263 -17.398 4.677 1.00 0.00 MEMB H

ATOM 2406 C315 POPC 18 -3.064 -17.960 2.753 1.00 0.00 MEMB C

ATOM 2407 H15X POPC 18 -3.439 -16.899 2.841 1.00 0.00 MEMB H

ATOM 2408 H15Y POPC 18 -3.606 -18.321 1.844 1.00 0.00 MEMB H

ATOM 2409 C316 POPC 18 -1.518 -18.053 2.676 1.00 0.00 MEMB C

ATOM 2410 H16X POPC 18 -0.954 -17.418 3.325 1.00 0.00 MEMB H

ATOM 2411 H16Y POPC 18 -1.102 -17.314 2.004 1.00 0.00 MEMB H

ATOM 2412 H16Z POPC 18 -1.088 -18.803 1.988 1.00 0.00 MEMB H

ATOM 2413 N POPC 19 -9.071 19.907 19.839 1.00 0.00 MEMB N

ATOM 2414 C12 POPC 19 -9.966 21.096 19.672 1.00 0.00 MEMB C

ATOM 2415 H12A POPC 19 -9.380 21.868 19.179 1.00 0.00 MEMB H

ATOM 2416 H12B POPC 19 -10.264 21.411 20.658 1.00 0.00 MEMB H

ATOM 2417 C13 POPC 19 -8.923 19.350 18.541 1.00 0.00 MEMB C

ATOM 2418 H13A POPC 19 -8.306 18.445 18.588 1.00 0.00 MEMB H

ATOM 2419 H13B POPC 19 -9.850 19.006 18.077 1.00 0.00 MEMB H

ATOM 2420 H13C POPC 19 -8.374 20.004 17.867 1.00 0.00 MEMB H

ATOM 2421 C14 POPC 19 -7.748 20.272 20.406 1.00 0.00 MEMB C

ATOM 2422 H14A POPC 19 -7.867 20.436 21.475 1.00 0.00 MEMB H

ATOM 2423 H14B POPC 19 -7.016 19.476 20.290 1.00 0.00 MEMB H

ATOM 2424 H14C POPC 19 -7.334 21.168 19.964 1.00 0.00 MEMB H

ATOM 2425 C15 POPC 19 -9.640 18.781 20.631 1.00 0.00 MEMB C

ATOM 2426 H15A POPC 19 -9.767 19.078 21.650 1.00 0.00 MEMB H

ATOM 2427 H15B POPC 19 -8.989 17.910 20.561 1.00 0.00 MEMB H

ATOM 2428 H15C POPC 19 -10.584 18.480 20.164 1.00 0.00 MEMB H

ATOM 2429 C11 POPC 19 -11.242 20.962 18.806 1.00 0.00 MEMB C

ATOM 2430 H11A POPC 19 -10.984 20.894 17.719 1.00 0.00 MEMB H

ATOM 2431 H11B POPC 19 -11.791 21.914 18.951 1.00 0.00 MEMB H

ATOM 2432 P POPC 19 -12.572 18.815 18.236 1.00 0.00 MEMB P

ATOM 2433 O13 POPC 19 -13.868 18.411 18.708 1.00 0.00 MEMB O

ATOM 2434 O14 POPC 19 -11.508 17.817 18.162 1.00 0.00 MEMB O

ATOM 2435 O12 POPC 19 -12.068 19.902 19.254 1.00 0.00 MEMB O

ATOM 2436 O11 POPC 19 -12.817 19.591 16.882 1.00 0.00 MEMB O

ATOM 2437 C1 POPC 19 -13.560 18.978 15.815 1.00 0.00 MEMB C

ATOM 2438 HA POPC 19 -14.050 19.781 15.219 1.00 0.00 MEMB H

ATOM 2439 HB POPC 19 -14.361 18.275 16.152 1.00 0.00 MEMB H

ATOM 2440 C2 POPC 19 -12.589 18.277 14.858 1.00 0.00 MEMB C

ATOM 2441 HS POPC 19 -12.289 17.308 15.340 1.00 0.00 MEMB H

ATOM 2442 O21 POPC 19 -11.514 19.217 14.682 1.00 0.00 MEMB O

ATOM 2443 C21 POPC 19 -10.356 18.637 14.612 1.00 0.00 MEMB C

ATOM 2444 O22 POPC 19 -10.154 17.439 14.678 1.00 0.00 MEMB O

ATOM 2445 C22 POPC 19 -9.290 19.715 14.372 1.00 0.00 MEMB C

ATOM 2446 H2R POPC 19 -8.287 19.301 14.548 1.00 0.00 MEMB H

ATOM 2447 H2S POPC 19 -9.405 20.581 15.051 1.00 0.00 MEMB H

ATOM 2448 C3 POPC 19 -13.224 17.969 13.486 1.00 0.00 MEMB C

ATOM 2449 HX POPC 19 -13.755 18.883 13.153 1.00 0.00 MEMB H

ATOM 2450 HY POPC 19 -13.967 17.148 13.595 1.00 0.00 MEMB H

ATOM 2451 O31 POPC 19 -12.218 17.725 12.502 1.00 0.00 MEMB O

ATOM 2452 C31 POPC 19 -12.248 16.586 11.898 1.00 0.00 MEMB C

ATOM 2453 O32 POPC 19 -12.951 15.630 12.118 1.00 0.00 MEMB O

ATOM 2454 C32 POPC 19 -11.439 16.717 10.690 1.00 0.00 MEMB C

ATOM 2455 H2X POPC 19 -10.404 17.040 10.885 1.00 0.00 MEMB H

ATOM 2456 H2Y POPC 19 -11.969 17.436 10.011 1.00 0.00 MEMB H

ATOM 2457 C23 POPC 19 -9.076 19.993 12.902 1.00 0.00 MEMB C

ATOM 2458 H3R POPC 19 -9.901 20.635 12.544 1.00 0.00 MEMB H

ATOM 2459 H3S POPC 19 -9.101 19.007 12.374 1.00 0.00 MEMB H

ATOM 2460 C24 POPC 19 -7.690 20.600 12.639 1.00 0.00 MEMB C

ATOM 2461 H4R POPC 19 -6.899 19.994 13.136 1.00 0.00 MEMB H

ATOM 2462 H4S POPC 19 -7.630 21.603 13.112 1.00 0.00 MEMB H

ATOM 2463 C25 POPC 19 -7.388 20.664 11.133 1.00 0.00 MEMB C

ATOM 2464 H5R POPC 19 -7.224 19.625 10.773 1.00 0.00 MEMB H

ATOM 2465 H5S POPC 19 -6.440 21.231 10.997 1.00 0.00 MEMB H

ATOM 2466 C26 POPC 19 -8.508 21.281 10.265 1.00 0.00 MEMB C

ATOM 2467 H6R POPC 19 -9.471 20.750 10.417 1.00 0.00 MEMB H

ATOM 2468 H6S POPC 19 -8.244 21.219 9.191 1.00 0.00 MEMB H

ATOM 2469 C27 POPC 19 -8.716 22.745 10.604 1.00 0.00 MEMB C

ATOM 2470 H7R POPC 19 -7.782 23.281 10.315 1.00 0.00 MEMB H

ATOM 2471 H7S POPC 19 -8.818 22.733 11.709 1.00 0.00 MEMB H

ATOM 2472 C28 POPC 19 -9.955 23.470 10.059 1.00 0.00 MEMB C

ATOM 2473 H8R POPC 19 -10.097 23.209 8.996 1.00 0.00 MEMB H

ATOM 2474 H8S POPC 19 -9.784 24.554 10.003 1.00 0.00 MEMB H

ATOM 2475 C29 POPC 19 -11.152 23.204 10.939 1.00 0.00 MEMB C

ATOM 2476 H91 POPC 19 -11.194 23.752 11.898 1.00 0.00 MEMB H

ATOM 2477 C210 POPC 19 -12.113 22.323 10.631 1.00 0.00 MEMB C

ATOM 2478 H101 POPC 19 -12.941 22.164 11.337 1.00 0.00 MEMB H

ATOM 2479 C211 POPC 19 -12.129 21.486 9.374 1.00 0.00 MEMB C

ATOM 2480 H11R POPC 19 -12.316 20.431 9.655 1.00 0.00 MEMB H

ATOM 2481 H11S POPC 19 -11.141 21.490 8.868 1.00 0.00 MEMB H

ATOM 2482 C212 POPC 19 -13.198 21.968 8.366 1.00 0.00 MEMB C

ATOM 2483 H12R POPC 19 -12.979 23.009 8.041 1.00 0.00 MEMB H

ATOM 2484 H12S POPC 19 -14.168 21.984 8.915 1.00 0.00 MEMB H

ATOM 2485 C213 POPC 19 -13.389 21.067 7.123 1.00 0.00 MEMB C

ATOM 2486 H13R POPC 19 -14.346 21.326 6.615 1.00 0.00 MEMB H

ATOM 2487 H13S POPC 19 -13.491 20.023 7.486 1.00 0.00 MEMB H

ATOM 2488 C214 POPC 19 -12.266 21.118 6.067 1.00 0.00 MEMB C

ATOM 2489 H14R POPC 19 -11.281 21.049 6.578 1.00 0.00 MEMB H

ATOM 2490 H14S POPC 19 -12.294 22.088 5.522 1.00 0.00 MEMB H

ATOM 2491 C215 POPC 19 -12.345 19.942 5.078 1.00 0.00 MEMB C

ATOM 2492 H15R POPC 19 -13.352 19.860 4.614 1.00 0.00 MEMB H

ATOM 2493 H15S POPC 19 -12.203 19.017 5.683 1.00 0.00 MEMB H

ATOM 2494 C216 POPC 19 -11.263 19.954 3.979 1.00 0.00 MEMB C

ATOM 2495 H16R POPC 19 -11.477 19.109 3.287 1.00 0.00 MEMB H

ATOM 2496 H16S POPC 19 -10.281 19.756 4.462 1.00 0.00 MEMB H

ATOM 2497 C217 POPC 19 -11.151 21.239 3.139 1.00 0.00 MEMB C

ATOM 2498 H17R POPC 19 -10.821 22.081 3.788 1.00 0.00 MEMB H

ATOM 2499 H17S POPC 19 -12.161 21.496 2.746 1.00 0.00 MEMB H

ATOM 2500 C218 POPC 19 -10.195 21.099 1.943 1.00 0.00 MEMB C

ATOM 2501 H18R POPC 19 -9.139 21.304 2.218 1.00 0.00 MEMB H

ATOM 2502 H18S POPC 19 -10.476 21.806 1.133 1.00 0.00 MEMB H

ATOM 2503 H18T POPC 19 -10.256 20.068 1.532 1.00 0.00 MEMB H

ATOM 2504 C33 POPC 19 -11.432 15.372 10.078 1.00 0.00 MEMB C

ATOM 2505 H3X POPC 19 -12.412 14.807 10.095 1.00 0.00 MEMB H

ATOM 2506 H3Y POPC 19 -10.629 14.683 10.425 1.00 0.00 MEMB H

ATOM 2507 C34 POPC 19 -11.210 15.745 8.704 1.00 0.00 MEMB C

ATOM 2508 H4X POPC 19 -10.148 15.926 8.393 1.00 0.00 MEMB H

ATOM 2509 H4Y POPC 19 -11.884 16.543 8.289 1.00 0.00 MEMB H

ATOM 2510 C35 POPC 19 -11.575 14.548 8.117 1.00 0.00 MEMB C

ATOM 2511 H5X POPC 19 -12.558 13.974 8.292 1.00 0.00 MEMB H

ATOM 2512 H5Y POPC 19 -10.829 13.778 8.408 1.00 0.00 MEMB H

ATOM 2513 C36 POPC 19 -11.476 15.111 6.735 1.00 0.00 MEMB C

ATOM 2514 H6X POPC 19 -12.398 14.494 6.602 1.00 0.00 MEMB H

ATOM 2515 H6Y POPC 19 -10.453 14.700 6.645 1.00 0.00 MEMB H

ATOM 2516 C37 POPC 19 -11.017 16.266 5.661 1.00 0.00 MEMB C

ATOM 2517 H7X POPC 19 -11.049 17.268 6.031 1.00 0.00 MEMB H

ATOM 2518 H7Y POPC 19 -12.003 16.336 6.103 1.00 0.00 MEMB H

ATOM 2519 C38 POPC 19 -11.138 16.283 3.897 1.00 0.00 MEMB C

ATOM 2520 H8X POPC 19 -11.886 16.916 3.399 1.00 0.00 MEMB H

ATOM 2521 H8Y POPC 19 -11.571 15.464 3.402 1.00 0.00 MEMB H

ATOM 2522 C39 POPC 19 -10.509 15.520 2.719 1.00 0.00 MEMB C

ATOM 2523 H9X POPC 19 -11.049 15.578 1.697 1.00 0.00 MEMB H

ATOM 2524 H9Y POPC 19 -10.113 14.456 2.641 1.00 0.00 MEMB H

ATOM 2525 C310 POPC 19 -9.654 16.587 3.042 1.00 0.00 MEMB C

ATOM 2526 H10X POPC 19 -9.149 16.497 4.068 1.00 0.00 MEMB H

ATOM 2527 H10Y POPC 19 -10.042 17.631 2.846 1.00 0.00 MEMB H

ATOM 2528 C311 POPC 19 -8.763 16.177 2.135 1.00 0.00 MEMB C

ATOM 2529 H11X POPC 19 -8.343 15.187 2.483 1.00 0.00 MEMB H

ATOM 2530 H11Y POPC 19 -8.234 17.155 2.106 1.00 0.00 MEMB H

ATOM 2531 C312 POPC 19 -8.932 15.976 0.794 1.00 0.00 MEMB C

ATOM 2532 H12X POPC 19 -9.527 15.042 0.685 1.00 0.00 MEMB H

ATOM 2533 H12Y POPC 19 -7.851 15.863 0.536 1.00 0.00 MEMB H

ATOM 2534 C313 POPC 19 -9.418 17.150 0.150 1.00 0.00 MEMB C

ATOM 2535 H13X POPC 19 -8.747 17.988 0.444 1.00 0.00 MEMB H

ATOM 2536 H13Y POPC 19 -10.454 17.293 0.535 1.00 0.00 MEMB H

ATOM 2537 C314 POPC 19 -9.302 16.887 -1.303 1.00 0.00 MEMB C

ATOM 2538 H14X POPC 19 -9.905 16.010 -1.634 1.00 0.00 MEMB H

ATOM 2539 H14Y POPC 19 -8.256 16.867 -1.683 1.00 0.00 MEMB H

ATOM 2540 C315 POPC 19 -10.121 17.858 -1.927 1.00 0.00 MEMB C

ATOM 2541 H15X POPC 19 -11.120 17.746 -1.424 1.00 0.00 MEMB H

ATOM 2542 H15Y POPC 19 -10.145 17.642 -3.016 1.00 0.00 MEMB H

ATOM 2543 C316 POPC 19 -9.646 19.234 -1.771 1.00 0.00 MEMB C

ATOM 2544 H16X POPC 19 -8.784 19.279 -2.450 1.00 0.00 MEMB H

ATOM 2545 H16Y POPC 19 -9.545 19.479 -0.684 1.00 0.00 MEMB H

ATOM 2546 H16Z POPC 19 -10.144 20.060 -2.255 1.00 0.00 MEMB H

ATOM 2547 N POPC 20 -22.673 7.936 20.166 1.00 0.00 MEMB N

ATOM 2548 C12 POPC 20 -22.341 8.028 18.681 1.00 0.00 MEMB C

ATOM 2549 H12A POPC 20 -23.120 8.548 18.156 1.00 0.00 MEMB H

ATOM 2550 H12B POPC 20 -22.295 7.013 18.280 1.00 0.00 MEMB H

ATOM 2551 C13 POPC 20 -23.987 7.259 20.350 1.00 0.00 MEMB C

ATOM 2552 H13A POPC 20 -23.934 6.226 20.030 1.00 0.00 MEMB H

ATOM 2553 H13B POPC 20 -24.254 7.269 21.409 1.00 0.00 MEMB H

ATOM 2554 H13C POPC 20 -24.766 7.760 19.792 1.00 0.00 MEMB H

ATOM 2555 C14 POPC 20 -21.631 7.088 20.823 1.00 0.00 MEMB C

ATOM 2556 H14A POPC 20 -21.776 6.993 21.887 1.00 0.00 MEMB H

ATOM 2557 H14B POPC 20 -21.591 6.100 20.373 1.00 0.00 MEMB H

ATOM 2558 H14C POPC 20 -20.637 7.503 20.626 1.00 0.00 MEMB H

ATOM 2559 C15 POPC 20 -22.688 9.289 20.827 1.00 0.00 MEMB C

ATOM 2560 H15A POPC 20 -23.544 9.875 20.539 1.00 0.00 MEMB H

ATOM 2561 H15B POPC 20 -21.782 9.835 20.567 1.00 0.00 MEMB H

ATOM 2562 H15C POPC 20 -22.729 9.190 21.911 1.00 0.00 MEMB H

ATOM 2563 C11 POPC 20 -21.013 8.692 18.257 1.00 0.00 MEMB C

ATOM 2564 H11A POPC 20 -20.926 9.724 18.651 1.00 0.00 MEMB H

ATOM 2565 H11B POPC 20 -20.992 8.767 17.144 1.00 0.00 MEMB H

ATOM 2566 P POPC 20 -19.302 6.757 17.881 1.00 0.00 MEMB P

ATOM 2567 O13 POPC 20 -20.401 5.805 17.614 1.00 0.00 MEMB O

ATOM 2568 O14 POPC 20 -18.147 6.291 18.657 1.00 0.00 MEMB O

ATOM 2569 O12 POPC 20 -19.910 7.957 18.732 1.00 0.00 MEMB O

ATOM 2570 O11 POPC 20 -18.875 7.500 16.545 1.00 0.00 MEMB O

ATOM 2571 C1 POPC 20 -18.095 8.707 16.578 1.00 0.00 MEMB C

ATOM 2572 HA POPC 20 -17.036 8.457 16.811 1.00 0.00 MEMB H

ATOM 2573 HB POPC 20 -18.471 9.399 17.369 1.00 0.00 MEMB H

ATOM 2574 C2 POPC 20 -18.189 9.545 15.252 1.00 0.00 MEMB C

ATOM 2575 HS POPC 20 -17.827 10.571 15.456 1.00 0.00 MEMB H

ATOM 2576 O21 POPC 20 -19.565 9.547 14.841 1.00 0.00 MEMB O

ATOM 2577 C21 POPC 20 -19.930 10.181 13.763 1.00 0.00 MEMB C

ATOM 2578 O22 POPC 20 -19.230 10.792 12.976 1.00 0.00 MEMB O

ATOM 2579 C22 POPC 20 -21.435 10.014 13.636 1.00 0.00 MEMB C

ATOM 2580 H2R POPC 20 -21.824 10.912 13.116 1.00 0.00 MEMB H

ATOM 2581 H2S POPC 20 -21.870 9.974 14.657 1.00 0.00 MEMB H

ATOM 2582 C3 POPC 20 -17.263 8.996 14.159 1.00 0.00 MEMB C

ATOM 2583 HX POPC 20 -16.235 9.163 14.571 1.00 0.00 MEMB H

ATOM 2584 HY POPC 20 -17.315 9.448 13.155 1.00 0.00 MEMB H

ATOM 2585 O31 POPC 20 -17.565 7.650 13.965 1.00 0.00 MEMB O

ATOM 2586 C31 POPC 20 -16.488 7.008 13.801 1.00 0.00 MEMB C

ATOM 2587 O32 POPC 20 -15.329 7.358 13.748 1.00 0.00 MEMB O

ATOM 2588 C32 POPC 20 -16.863 5.748 13.555 1.00 0.00 MEMB C

ATOM 2589 H2X POPC 20 -17.966 5.549 13.673 1.00 0.00 MEMB H

ATOM 2590 H2Y POPC 20 -16.394 5.247 14.445 1.00 0.00 MEMB H

ATOM 2591 C23 POPC 20 -21.748 8.700 12.886 1.00 0.00 MEMB C

ATOM 2592 H3R POPC 20 -22.836 8.488 12.976 1.00 0.00 MEMB H

ATOM 2593 H3S POPC 20 -21.207 7.855 13.368 1.00 0.00 MEMB H

ATOM 2594 C24 POPC 20 -21.447 8.820 11.386 1.00 0.00 MEMB C

ATOM 2595 H4R POPC 20 -20.376 9.054 11.195 1.00 0.00 MEMB H

ATOM 2596 H4S POPC 20 -22.056 9.686 11.033 1.00 0.00 MEMB H

ATOM 2597 C25 POPC 20 -21.849 7.623 10.517 1.00 0.00 MEMB C

ATOM 2598 H5R POPC 20 -22.933 7.432 10.681 1.00 0.00 MEMB H

ATOM 2599 H5S POPC 20 -21.284 6.705 10.780 1.00 0.00 MEMB H

ATOM 2600 C26 POPC 20 -21.531 7.938 9.052 1.00 0.00 MEMB C

ATOM 2601 H6R POPC 20 -20.436 8.034 8.924 1.00 0.00 MEMB H

ATOM 2602 H6S POPC 20 -21.949 8.951 8.844 1.00 0.00 MEMB H

ATOM 2603 C27 POPC 20 -22.027 6.867 8.043 1.00 0.00 MEMB C

ATOM 2604 H7R POPC 20 -21.730 5.854 8.351 1.00 0.00 MEMB H

ATOM 2605 H7S POPC 20 -21.523 7.061 7.045 1.00 0.00 MEMB H

ATOM 2606 C28 POPC 20 -23.447 6.758 7.899 1.00 0.00 MEMB C

ATOM 2607 H8R POPC 20 -24.014 7.149 8.909 1.00 0.00 MEMB H

ATOM 2608 H8S POPC 20 -24.026 5.955 7.473 1.00 0.00 MEMB H

ATOM 2609 C29 POPC 20 -23.831 8.037 7.328 1.00 0.00 MEMB C

ATOM 2610 H91 POPC 20 -23.028 8.870 7.404 1.00 0.00 MEMB H

ATOM 2611 C210 POPC 20 -23.994 8.198 5.976 1.00 0.00 MEMB C

ATOM 2612 H101 POPC 20 -24.754 9.023 6.085 1.00 0.00 MEMB H

ATOM 2613 C211 POPC 20 -24.555 7.192 5.061 1.00 0.00 MEMB C

ATOM 2614 H11R POPC 20 -25.434 7.794 4.898 1.00 0.00 MEMB H

ATOM 2615 H11S POPC 20 -24.417 6.179 5.243 1.00 0.00 MEMB H

ATOM 2616 C212 POPC 20 -24.372 7.386 3.531 1.00 0.00 MEMB C

ATOM 2617 H12R POPC 20 -24.871 8.069 2.809 1.00 0.00 MEMB H

ATOM 2618 H12S POPC 20 -25.206 6.799 3.459 1.00 0.00 MEMB H

ATOM 2619 C213 POPC 20 -23.067 6.888 3.285 1.00 0.00 MEMB C

ATOM 2620 H13R POPC 20 -22.709 6.218 4.119 1.00 0.00 MEMB H

ATOM 2621 H13S POPC 20 -22.415 7.767 3.155 1.00 0.00 MEMB H

ATOM 2622 C214 POPC 20 -23.143 6.086 2.112 1.00 0.00 MEMB C

ATOM 2623 H14R POPC 20 -23.655 6.545 1.215 1.00 0.00 MEMB H

ATOM 2624 H14S POPC 20 -23.413 5.059 2.364 1.00 0.00 MEMB H

ATOM 2625 C215 POPC 20 -21.823 5.743 1.852 1.00 0.00 MEMB C

ATOM 2626 H15R POPC 20 -21.315 6.730 1.799 1.00 0.00 MEMB H

ATOM 2627 H15S POPC 20 -22.169 5.301 0.895 1.00 0.00 MEMB H

ATOM 2628 C216 POPC 20 -21.042 4.779 2.728 1.00 0.00 MEMB C

ATOM 2629 H16R POPC 20 -21.707 3.973 3.091 1.00 0.00 MEMB H

ATOM 2630 H16S POPC 20 -20.749 5.318 3.653 1.00 0.00 MEMB H

ATOM 2631 C217 POPC 20 -19.713 4.223 2.119 1.00 0.00 MEMB C

ATOM 2632 H17R POPC 20 -19.447 3.687 3.041 1.00 0.00 MEMB H

ATOM 2633 H17S POPC 20 -18.885 4.942 2.074 1.00 0.00 MEMB H

ATOM 2634 C218 POPC 20 -19.730 3.365 0.755 1.00 0.00 MEMB C

ATOM 2635 H18R POPC 20 -19.066 2.582 0.386 1.00 0.00 MEMB H

ATOM 2636 H18S POPC 20 -20.009 3.509 -0.287 1.00 0.00 MEMB H

ATOM 2637 H18T POPC 20 -20.601 3.030 0.972 1.00 0.00 MEMB H

ATOM 2638 C33 POPC 20 -16.263 5.587 12.122 1.00 0.00 MEMB C

ATOM 2639 H3X POPC 20 -15.954 4.561 12.058 1.00 0.00 MEMB H

ATOM 2640 H3Y POPC 20 -15.181 5.516 11.931 1.00 0.00 MEMB H

ATOM 2641 C34 POPC 20 -17.286 6.214 11.060 1.00 0.00 MEMB C

ATOM 2642 H4X POPC 20 -16.987 7.247 11.069 1.00 0.00 MEMB H

ATOM 2643 H4Y POPC 20 -18.357 6.206 11.354 1.00 0.00 MEMB H

ATOM 2644 C35 POPC 20 -17.438 5.660 9.620 1.00 0.00 MEMB C

ATOM 2645 H5X POPC 20 -18.115 4.875 9.906 1.00 0.00 MEMB H

ATOM 2646 H5Y POPC 20 -16.413 5.394 9.384 1.00 0.00 MEMB H

ATOM 2647 C36 POPC 20 -18.372 6.052 8.415 1.00 0.00 MEMB C

ATOM 2648 H6X POPC 20 -18.123 7.109 8.262 1.00 0.00 MEMB H

ATOM 2649 H6Y POPC 20 -19.413 6.018 8.782 1.00 0.00 MEMB H

ATOM 2650 C37 POPC 20 -18.474 5.150 7.101 1.00 0.00 MEMB C

ATOM 2651 H7X POPC 20 -19.148 4.290 7.232 1.00 0.00 MEMB H

ATOM 2652 H7Y POPC 20 -17.689 4.551 6.665 1.00 0.00 MEMB H

ATOM 2653 C38 POPC 20 -18.675 5.867 5.782 1.00 0.00 MEMB C

ATOM 2654 H8X POPC 20 -19.701 6.241 5.881 1.00 0.00 MEMB H

ATOM 2655 H8Y POPC 20 -18.551 5.137 4.937 1.00 0.00 MEMB H

ATOM 2656 C39 POPC 20 -17.621 6.917 5.496 1.00 0.00 MEMB C

ATOM 2657 H9X POPC 20 -16.681 6.388 5.229 1.00 0.00 MEMB H

ATOM 2658 H9Y POPC 20 -17.530 7.621 6.320 1.00 0.00 MEMB H

ATOM 2659 C310 POPC 20 -17.900 7.824 4.349 1.00 0.00 MEMB C

ATOM 2660 H10X POPC 20 -17.902 7.122 3.499 1.00 0.00 MEMB H

ATOM 2661 H10Y POPC 20 -17.080 8.578 4.247 1.00 0.00 MEMB H

ATOM 2662 C311 POPC 20 -19.216 8.546 4.562 1.00 0.00 MEMB C

ATOM 2663 H11X POPC 20 -19.181 9.134 5.497 1.00 0.00 MEMB H

ATOM 2664 H11Y POPC 20 -20.030 7.782 4.615 1.00 0.00 MEMB H

ATOM 2665 C312 POPC 20 -19.433 9.528 3.441 1.00 0.00 MEMB C

ATOM 2666 H12X POPC 20 -20.004 8.902 2.781 1.00 0.00 MEMB H

ATOM 2667 H12Y POPC 20 -18.515 9.955 3.005 1.00 0.00 MEMB H

ATOM 2668 C313 POPC 20 -20.589 10.452 3.397 1.00 0.00 MEMB C

ATOM 2669 H13X POPC 20 -20.465 11.094 4.284 1.00 0.00 MEMB H

ATOM 2670 H13Y POPC 20 -21.481 9.768 3.474 1.00 0.00 MEMB H

ATOM 2671 C314 POPC 20 -20.728 11.281 2.107 1.00 0.00 MEMB C

ATOM 2672 H14X POPC 20 -19.755 11.437 1.605 1.00 0.00 MEMB H

ATOM 2673 H14Y POPC 20 -21.147 12.262 2.427 1.00 0.00 MEMB H

ATOM 2674 C315 POPC 20 -21.778 10.771 1.132 1.00 0.00 MEMB C

ATOM 2675 H15X POPC 20 -22.204 11.594 0.508 1.00 0.00 MEMB H

ATOM 2676 H15Y POPC 20 -22.550 10.283 1.744 1.00 0.00 MEMB H

ATOM 2677 C316 POPC 20 -21.424 9.642 0.251 1.00 0.00 MEMB C

ATOM 2678 H16X POPC 20 -21.191 8.740 0.830 1.00 0.00 MEMB H

ATOM 2679 H16Y POPC 20 -20.720 9.922 -0.562 1.00 0.00 MEMB H

ATOM 2680 H16Z POPC 20 -22.316 9.240 -0.151 1.00 0.00 MEMB H

ATOM 2681 N POPC 21 19.460 -2.554 17.747 1.00 0.00 MEMB N

ATOM 2682 C12 POPC 21 19.448 -1.474 18.820 1.00 0.00 MEMB C

ATOM 2683 H12A POPC 21 18.570 -1.618 19.439 1.00 0.00 MEMB H

ATOM 2684 H12B POPC 21 19.358 -0.498 18.359 1.00 0.00 MEMB H

ATOM 2685 C13 POPC 21 19.333 -3.895 18.410 1.00 0.00 MEMB C

ATOM 2686 H13A POPC 21 18.338 -4.016 18.828 1.00 0.00 MEMB H

ATOM 2687 H13B POPC 21 19.504 -4.701 17.709 1.00 0.00 MEMB H

ATOM 2688 H13C POPC 21 20.070 -3.974 19.208 1.00 0.00 MEMB H

ATOM 2689 C14 POPC 21 18.336 -2.312 16.800 1.00 0.00 MEMB C

ATOM 2690 H14A POPC 21 18.406 -3.001 15.973 1.00 0.00 MEMB H

ATOM 2691 H14B POPC 21 17.359 -2.414 17.281 1.00 0.00 MEMB H

ATOM 2692 H14C POPC 21 18.424 -1.307 16.401 1.00 0.00 MEMB H

ATOM 2693 C15 POPC 21 20.735 -2.457 16.978 1.00 0.00 MEMB C

ATOM 2694 H15A POPC 21 20.752 -1.517 16.445 1.00 0.00 MEMB H

ATOM 2695 H15B POPC 21 20.878 -3.268 16.283 1.00 0.00 MEMB H

ATOM 2696 H15C POPC 21 21.588 -2.419 17.670 1.00 0.00 MEMB H

ATOM 2697 C11 POPC 21 20.621 -1.410 19.832 1.00 0.00 MEMB C

ATOM 2698 H11A POPC 21 20.691 -2.392 20.346 1.00 0.00 MEMB H

ATOM 2699 H11B POPC 21 20.375 -0.623 20.581 1.00 0.00 MEMB H

ATOM 2700 P POPC 21 23.127 -0.983 20.205 1.00 0.00 MEMB P

ATOM 2701 O13 POPC 21 23.520 -2.294 20.743 1.00 0.00 MEMB O

ATOM 2702 O14 POPC 21 22.934 -0.003 21.292 1.00 0.00 MEMB O

ATOM 2703 O12 POPC 21 21.858 -1.088 19.235 1.00 0.00 MEMB O

ATOM 2704 O11 POPC 21 24.110 -0.348 19.142 1.00 0.00 MEMB O

ATOM 2705 C1 POPC 21 24.693 -0.833 17.962 1.00 0.00 MEMB C

ATOM 2706 HA POPC 21 24.227 -1.712 17.493 1.00 0.00 MEMB H

ATOM 2707 HB POPC 21 25.704 -0.949 18.301 1.00 0.00 MEMB H

ATOM 2708 C2 POPC 21 24.573 -0.072 16.646 1.00 0.00 MEMB C

ATOM 2709 HS POPC 21 25.306 0.768 16.720 1.00 0.00 MEMB H

ATOM 2710 O21 POPC 21 23.199 0.245 16.598 1.00 0.00 MEMB O

ATOM 2711 C21 POPC 21 22.953 1.457 16.271 1.00 0.00 MEMB C

ATOM 2712 O22 POPC 21 23.769 2.309 15.963 1.00 0.00 MEMB O

ATOM 2713 C22 POPC 21 21.442 1.537 16.134 1.00 0.00 MEMB C

ATOM 2714 H2R POPC 21 21.134 2.593 16.260 1.00 0.00 MEMB H

ATOM 2715 H2S POPC 21 21.027 0.934 16.974 1.00 0.00 MEMB H

ATOM 2716 C3 POPC 21 24.895 -0.914 15.390 1.00 0.00 MEMB C

ATOM 2717 HX POPC 21 24.369 -1.871 15.517 1.00 0.00 MEMB H

ATOM 2718 HY POPC 21 25.896 -1.226 15.293 1.00 0.00 MEMB H

ATOM 2719 O31 POPC 21 24.444 -0.303 14.177 1.00 0.00 MEMB O

ATOM 2720 C31 POPC 21 25.356 0.419 13.559 1.00 0.00 MEMB C

ATOM 2721 O32 POPC 21 26.538 0.488 13.817 1.00 0.00 MEMB O

ATOM 2722 C32 POPC 21 24.684 1.083 12.382 1.00 0.00 MEMB C

ATOM 2723 H2X POPC 21 24.046 1.909 12.754 1.00 0.00 MEMB H

ATOM 2724 H2Y POPC 21 24.072 0.315 11.864 1.00 0.00 MEMB H

ATOM 2725 C23 POPC 21 21.025 0.958 14.757 1.00 0.00 MEMB C

ATOM 2726 H3R POPC 21 19.982 0.574 14.814 1.00 0.00 MEMB H

ATOM 2727 H3S POPC 21 21.676 0.089 14.502 1.00 0.00 MEMB H

ATOM 2728 C24 POPC 21 21.112 2.009 13.639 1.00 0.00 MEMB C

ATOM 2729 H4R POPC 21 22.001 2.664 13.808 1.00 0.00 MEMB H

ATOM 2730 H4S POPC 21 20.225 2.681 13.669 1.00 0.00 MEMB H

ATOM 2731 C25 POPC 21 21.300 1.416 12.240 1.00 0.00 MEMB C

ATOM 2732 H5R POPC 21 22.144 0.708 12.240 1.00 0.00 MEMB H

ATOM 2733 H5S POPC 21 21.547 2.270 11.562 1.00 0.00 MEMB H

ATOM 2734 C26 POPC 21 20.097 0.677 11.668 1.00 0.00 MEMB C

ATOM 2735 H6R POPC 21 19.247 1.321 11.965 1.00 0.00 MEMB H

ATOM 2736 H6S POPC 21 19.863 -0.283 12.159 1.00 0.00 MEMB H

ATOM 2737 C27 POPC 21 20.235 0.509 10.133 1.00 0.00 MEMB C

ATOM 2738 H7R POPC 21 20.454 1.518 9.705 1.00 0.00 MEMB H

ATOM 2739 H7S POPC 21 19.271 0.176 9.687 1.00 0.00 MEMB H

ATOM 2740 C28 POPC 21 21.361 -0.406 9.620 1.00 0.00 MEMB C

ATOM 2741 H8R POPC 21 22.345 0.012 9.916 1.00 0.00 MEMB H

ATOM 2742 H8S POPC 21 21.310 -0.320 8.512 1.00 0.00 MEMB H

ATOM 2743 C29 POPC 21 21.245 -1.839 10.073 1.00 0.00 MEMB C

ATOM 2744 H91 POPC 21 21.202 -1.978 11.164 1.00 0.00 MEMB H

ATOM 2745 C210 POPC 21 21.233 -2.925 9.277 1.00 0.00 MEMB C

ATOM 2746 H101 POPC 21 21.181 -3.916 9.758 1.00 0.00 MEMB H

ATOM 2747 C211 POPC 21 21.337 -2.909 7.762 1.00 0.00 MEMB C

ATOM 2748 H11R POPC 21 22.236 -3.399 7.404 1.00 0.00 MEMB H

ATOM 2749 H11S POPC 21 21.436 -1.894 7.332 1.00 0.00 MEMB H

ATOM 2750 C212 POPC 21 20.425 -3.786 6.915 1.00 0.00 MEMB C

ATOM 2751 H12R POPC 21 20.488 -4.848 7.248 1.00 0.00 MEMB H

ATOM 2752 H12S POPC 21 20.852 -3.754 5.881 1.00 0.00 MEMB H

ATOM 2753 C213 POPC 21 18.988 -3.313 6.877 1.00 0.00 MEMB C

ATOM 2754 H13R POPC 21 18.480 -3.586 7.815 1.00 0.00 MEMB H

ATOM 2755 H13S POPC 21 18.510 -3.910 6.075 1.00 0.00 MEMB H

ATOM 2756 C214 POPC 21 18.792 -1.807 6.643 1.00 0.00 MEMB C

ATOM 2757 H14R POPC 21 19.267 -1.149 7.393 1.00 0.00 MEMB H

ATOM 2758 H14S POPC 21 17.688 -1.638 6.633 1.00 0.00 MEMB H

ATOM 2759 C215 POPC 21 19.311 -1.385 5.314 1.00 0.00 MEMB C

ATOM 2760 H15R POPC 21 20.392 -1.604 5.150 1.00 0.00 MEMB H

ATOM 2761 H15S POPC 21 19.151 -0.299 5.097 1.00 0.00 MEMB H

ATOM 2762 C216 POPC 21 18.564 -2.197 4.342 1.00 0.00 MEMB C

ATOM 2763 H16R POPC 21 17.452 -1.991 4.380 1.00 0.00 MEMB H

ATOM 2764 H16S POPC 21 18.708 -3.299 4.290 1.00 0.00 MEMB H

ATOM 2765 C217 POPC 21 19.248 -1.830 3.128 1.00 0.00 MEMB C

ATOM 2766 H17R POPC 21 19.566 -0.768 2.969 1.00 0.00 MEMB H

ATOM 2767 H17S POPC 21 18.245 -2.036 2.716 1.00 0.00 MEMB H

ATOM 2768 C218 POPC 21 20.180 -2.830 2.503 1.00 0.00 MEMB C

ATOM 2769 H18R POPC 21 19.668 -3.803 2.376 1.00 0.00 MEMB H

ATOM 2770 H18S POPC 21 21.083 -2.838 3.100 1.00 0.00 MEMB H

ATOM 2771 H18T POPC 21 20.481 -2.581 1.464 1.00 0.00 MEMB H

ATOM 2772 C33 POPC 21 25.720 1.621 11.406 1.00 0.00 MEMB C

ATOM 2773 H3X POPC 21 26.502 0.844 11.234 1.00 0.00 MEMB H

ATOM 2774 H3Y POPC 21 26.230 2.521 11.821 1.00 0.00 MEMB H

ATOM 2775 C34 POPC 21 25.075 1.967 10.083 1.00 0.00 MEMB C

ATOM 2776 H4X POPC 21 24.435 2.864 10.198 1.00 0.00 MEMB H

ATOM 2777 H4Y POPC 21 24.410 1.143 9.735 1.00 0.00 MEMB H

ATOM 2778 C35 POPC 21 26.170 2.211 9.063 1.00 0.00 MEMB C

ATOM 2779 H5X POPC 21 26.854 1.349 9.080 1.00 0.00 MEMB H

ATOM 2780 H5Y POPC 21 26.747 3.126 9.329 1.00 0.00 MEMB H

ATOM 2781 C36 POPC 21 25.576 2.277 7.677 1.00 0.00 MEMB C

ATOM 2782 H6X POPC 21 24.763 2.965 7.602 1.00 0.00 MEMB H

ATOM 2783 H6Y POPC 21 25.043 1.338 7.457 1.00 0.00 MEMB H

ATOM 2784 C37 POPC 21 26.532 2.676 6.581 1.00 0.00 MEMB C

ATOM 2785 H7X POPC 21 27.122 1.785 6.652 1.00 0.00 MEMB H

ATOM 2786 H7Y POPC 21 27.267 3.485 6.794 1.00 0.00 MEMB H

ATOM 2787 C38 POPC 21 25.776 2.868 5.250 1.00 0.00 MEMB C

ATOM 2788 H8X POPC 21 26.492 3.236 4.507 1.00 0.00 MEMB H

ATOM 2789 H8Y POPC 21 25.220 3.788 5.095 1.00 0.00 MEMB H

ATOM 2790 C39 POPC 21 24.622 1.840 4.912 1.00 0.00 MEMB C

ATOM 2791 H9X POPC 21 24.238 2.135 3.923 1.00 0.00 MEMB H

ATOM 2792 H9Y POPC 21 23.821 2.010 5.649 1.00 0.00 MEMB H

ATOM 2793 C310 POPC 21 24.917 0.312 4.909 1.00 0.00 MEMB C

ATOM 2794 H10X POPC 21 25.385 0.173 5.865 1.00 0.00 MEMB H

ATOM 2795 H10Y POPC 21 25.573 -0.159 4.142 1.00 0.00 MEMB H

ATOM 2796 C311 POPC 21 23.835 -0.704 4.799 1.00 0.00 MEMB C

ATOM 2797 H11X POPC 21 23.311 -0.686 5.767 1.00 0.00 MEMB H

ATOM 2798 H11Y POPC 21 24.384 -1.655 4.594 1.00 0.00 MEMB H

ATOM 2799 C312 POPC 21 22.908 -0.547 3.648 1.00 0.00 MEMB C

ATOM 2800 H12X POPC 21 22.451 0.466 3.749 1.00 0.00 MEMB H

ATOM 2801 H12Y POPC 21 22.157 -1.347 3.712 1.00 0.00 MEMB H

ATOM 2802 C313 POPC 21 23.588 -0.643 2.306 1.00 0.00 MEMB C

ATOM 2803 H13X POPC 21 23.992 -1.598 1.934 1.00 0.00 MEMB H

ATOM 2804 H13Y POPC 21 24.519 -0.154 2.506 1.00 0.00 MEMB H

ATOM 2805 C314 POPC 21 22.564 -0.098 1.346 1.00 0.00 MEMB C

ATOM 2806 H14X POPC 21 22.023 0.840 1.614 1.00 0.00 MEMB H

ATOM 2807 H14Y POPC 21 21.923 -0.968 1.508 1.00 0.00 MEMB H

ATOM 2808 C315 POPC 21 22.930 0.006 -0.099 1.00 0.00 MEMB C

ATOM 2809 H15X POPC 21 23.970 0.255 -0.058 1.00 0.00 MEMB H

ATOM 2810 H15Y POPC 21 22.638 1.020 -0.443 1.00 0.00 MEMB H

ATOM 2811 C316 POPC 21 22.600 -1.210 -1.041 1.00 0.00 MEMB C

ATOM 2812 H16X POPC 21 23.137 -1.191 -1.951 1.00 0.00 MEMB H

ATOM 2813 H16Y POPC 21 21.845 -1.149 -1.777 1.00 0.00 MEMB H

ATOM 2814 H16Z POPC 21 22.129 -2.115 -0.709 1.00 0.00 MEMB H

ATOM 2815 N POPC 22 -3.040 23.139 21.260 1.00 0.00 MEMB N

ATOM 2816 C12 POPC 22 -2.087 23.591 20.141 1.00 0.00 MEMB C

ATOM 2817 H12A POPC 22 -1.065 23.623 20.499 1.00 0.00 MEMB H

ATOM 2818 H12B POPC 22 -2.364 24.592 19.830 1.00 0.00 MEMB H

ATOM 2819 C13 POPC 22 -2.941 21.656 21.419 1.00 0.00 MEMB C

ATOM 2820 H13A POPC 22 -1.908 21.353 21.613 1.00 0.00 MEMB H

ATOM 2821 H13B POPC 22 -3.563 21.264 22.211 1.00 0.00 MEMB H

ATOM 2822 H13C POPC 22 -3.238 21.194 20.475 1.00 0.00 MEMB H

ATOM 2823 C14 POPC 22 -2.764 23.768 22.591 1.00 0.00 MEMB C

ATOM 2824 H14A POPC 22 -2.781 24.852 22.540 1.00 0.00 MEMB H

ATOM 2825 H14B POPC 22 -3.488 23.432 23.326 1.00 0.00 MEMB H

ATOM 2826 H14C POPC 22 -1.795 23.444 22.956 1.00 0.00 MEMB H

ATOM 2827 C15 POPC 22 -4.448 23.492 20.891 1.00 0.00 MEMB C

ATOM 2828 H15A POPC 22 -4.664 23.154 19.872 1.00 0.00 MEMB H

ATOM 2829 H15B POPC 22 -4.596 24.567 20.896 1.00 0.00 MEMB H

ATOM 2830 H15C POPC 22 -5.144 23.054 21.594 1.00 0.00 MEMB H

ATOM 2831 C11 POPC 22 -2.056 22.808 18.793 1.00 0.00 MEMB C

ATOM 2832 H11A POPC 22 -1.722 21.751 18.896 1.00 0.00 MEMB H

ATOM 2833 H11B POPC 22 -1.271 23.304 18.165 1.00 0.00 MEMB H

ATOM 2834 P POPC 22 -3.391 22.370 16.633 1.00 0.00 MEMB P

ATOM 2835 O13 POPC 22 -4.811 22.416 16.206 1.00 0.00 MEMB O

ATOM 2836 O14 POPC 22 -2.663 21.115 16.464 1.00 0.00 MEMB O

ATOM 2837 O12 POPC 22 -3.333 22.851 18.159 1.00 0.00 MEMB O

ATOM 2838 O11 POPC 22 -2.556 23.475 15.874 1.00 0.00 MEMB O

ATOM 2839 C1 POPC 22 -2.810 24.862 16.034 1.00 0.00 MEMB C

ATOM 2840 HA POPC 22 -2.170 25.307 16.823 1.00 0.00 MEMB H

ATOM 2841 HB POPC 22 -3.862 24.965 16.378 1.00 0.00 MEMB H

ATOM 2842 C2 POPC 22 -2.576 25.630 14.692 1.00 0.00 MEMB C

ATOM 2843 HS POPC 22 -3.038 26.649 14.716 1.00 0.00 MEMB H

ATOM 2844 O21 POPC 22 -3.020 24.784 13.631 1.00 0.00 MEMB O

ATOM 2845 C21 POPC 22 -4.228 25.034 13.364 1.00 0.00 MEMB C

ATOM 2846 O22 POPC 22 -4.833 26.055 13.632 1.00 0.00 MEMB O

ATOM 2847 C22 POPC 22 -4.806 23.994 12.488 1.00 0.00 MEMB C

ATOM 2848 H2R POPC 22 -5.638 23.602 13.083 1.00 0.00 MEMB H

ATOM 2849 H2S POPC 22 -4.174 23.123 12.312 1.00 0.00 MEMB H

ATOM 2850 C3 POPC 22 -1.127 25.974 14.350 1.00 0.00 MEMB C

ATOM 2851 HX POPC 22 -0.743 26.658 15.130 1.00 0.00 MEMB H

ATOM 2852 HY POPC 22 -1.132 26.521 13.370 1.00 0.00 MEMB H

ATOM 2853 O31 POPC 22 -0.351 24.782 14.346 1.00 0.00 MEMB O

ATOM 2854 C31 POPC 22 0.290 24.474 13.252 1.00 0.00 MEMB C

ATOM 2855 O32 POPC 22 0.412 25.158 12.250 1.00 0.00 MEMB O

ATOM 2856 C32 POPC 22 0.850 23.088 13.445 1.00 0.00 MEMB C

ATOM 2857 H2X POPC 22 1.268 23.012 14.474 1.00 0.00 MEMB H

ATOM 2858 H2Y POPC 22 1.667 22.942 12.715 1.00 0.00 MEMB H

ATOM 2859 C23 POPC 22 -5.068 24.828 11.250 1.00 0.00 MEMB C

ATOM 2860 H3R POPC 22 -5.490 25.830 11.513 1.00 0.00 MEMB H

ATOM 2861 H3S POPC 22 -5.735 24.358 10.495 1.00 0.00 MEMB H

ATOM 2862 C24 POPC 22 -3.771 25.186 10.624 1.00 0.00 MEMB C

ATOM 2863 H4R POPC 22 -3.193 24.290 10.327 1.00 0.00 MEMB H

ATOM 2864 H4S POPC 22 -3.151 25.862 11.267 1.00 0.00 MEMB H

ATOM 2865 C25 POPC 22 -4.040 25.936 9.408 1.00 0.00 MEMB C

ATOM 2866 H5R POPC 22 -4.515 26.922 9.674 1.00 0.00 MEMB H

ATOM 2867 H5S POPC 22 -4.716 25.397 8.709 1.00 0.00 MEMB H

ATOM 2868 C26 POPC 22 -2.684 26.104 8.812 1.00 0.00 MEMB C

ATOM 2869 H6R POPC 22 -2.123 25.267 8.375 1.00 0.00 MEMB H

ATOM 2870 H6S POPC 22 -2.038 26.525 9.621 1.00 0.00 MEMB H

ATOM 2871 C27 POPC 22 -3.005 26.968 7.680 1.00 0.00 MEMB C

ATOM 2872 H7R POPC 22 -2.173 27.195 7.049 1.00 0.00 MEMB H

ATOM 2873 H7S POPC 22 -3.525 27.800 8.219 1.00 0.00 MEMB H

ATOM 2874 C28 POPC 22 -3.930 26.502 6.645 1.00 0.00 MEMB C

ATOM 2875 H8R POPC 22 -4.252 27.112 5.827 1.00 0.00 MEMB H

ATOM 2876 H8S POPC 22 -4.785 26.656 7.254 1.00 0.00 MEMB H

ATOM 2877 C29 POPC 22 -3.672 25.268 5.914 1.00 0.00 MEMB C

ATOM 2878 H91 POPC 22 -4.489 24.570 5.987 1.00 0.00 MEMB H

ATOM 2879 C210 POPC 22 -2.602 24.993 5.180 1.00 0.00 MEMB C

ATOM 2880 H101 POPC 22 -2.484 24.069 4.632 1.00 0.00 MEMB H

ATOM 2881 C211 POPC 22 -1.442 25.813 4.924 1.00 0.00 MEMB C

ATOM 2882 H11R POPC 22 -1.691 26.792 4.608 1.00 0.00 MEMB H

ATOM 2883 H11S POPC 22 -1.167 25.628 3.887 1.00 0.00 MEMB H

ATOM 2884 C212 POPC 22 -0.356 25.704 6.025 1.00 0.00 MEMB C

ATOM 2885 H12R POPC 22 -0.688 26.081 6.997 1.00 0.00 MEMB H

ATOM 2886 H12S POPC 22 0.525 26.339 5.986 1.00 0.00 MEMB H

ATOM 2887 C213 POPC 22 0.435 24.422 5.971 1.00 0.00 MEMB C

ATOM 2888 H13R POPC 22 -0.275 23.687 6.320 1.00 0.00 MEMB H

ATOM 2889 H13S POPC 22 1.345 24.615 6.623 1.00 0.00 MEMB H

ATOM 2890 C214 POPC 22 0.922 23.976 4.598 1.00 0.00 MEMB C

ATOM 2891 H14R POPC 22 1.840 24.565 4.796 1.00 0.00 MEMB H

ATOM 2892 H14S POPC 22 0.360 24.223 3.664 1.00 0.00 MEMB H

ATOM 2893 C215 POPC 22 1.560 22.639 4.301 1.00 0.00 MEMB C

ATOM 2894 H15R POPC 22 0.651 22.009 4.306 1.00 0.00 MEMB H

ATOM 2895 H15S POPC 22 2.302 22.520 5.111 1.00 0.00 MEMB H

ATOM 2896 C216 POPC 22 2.259 22.352 2.950 1.00 0.00 MEMB C

ATOM 2897 H16R POPC 22 2.722 21.363 3.049 1.00 0.00 MEMB H

ATOM 2898 H16S POPC 22 3.155 22.925 2.608 1.00 0.00 MEMB H

ATOM 2899 C217 POPC 22 1.286 22.345 1.826 1.00 0.00 MEMB C

ATOM 2900 H17R POPC 22 0.907 23.313 1.438 1.00 0.00 MEMB H

ATOM 2901 H17S POPC 22 0.320 21.889 2.065 1.00 0.00 MEMB H

ATOM 2902 C218 POPC 22 1.947 21.609 0.715 1.00 0.00 MEMB C

ATOM 2903 H18R POPC 22 2.847 22.007 0.198 1.00 0.00 MEMB H

ATOM 2904 H18S POPC 22 1.263 21.983 0.014 1.00 0.00 MEMB H

ATOM 2905 H18T POPC 22 1.986 20.520 0.794 1.00 0.00 MEMB H

ATOM 2906 C33 POPC 22 -0.242 22.032 13.214 1.00 0.00 MEMB C

ATOM 2907 H3X POPC 22 -1.140 22.195 13.858 1.00 0.00 MEMB H

ATOM 2908 H3Y POPC 22 0.193 21.047 13.504 1.00 0.00 MEMB H

ATOM 2909 C34 POPC 22 -0.628 21.933 11.737 1.00 0.00 MEMB C

ATOM 2910 H4X POPC 22 0.314 21.872 11.146 1.00 0.00 MEMB H

ATOM 2911 H4Y POPC 22 -1.170 22.844 11.397 1.00 0.00 MEMB H

ATOM 2912 C35 POPC 22 -1.477 20.682 11.521 1.00 0.00 MEMB C

ATOM 2913 H5X POPC 22 -2.402 20.766 12.131 1.00 0.00 MEMB H

ATOM 2914 H5Y POPC 22 -0.903 19.806 11.911 1.00 0.00 MEMB H

ATOM 2915 C36 POPC 22 -1.885 20.386 10.074 1.00 0.00 MEMB C

ATOM 2916 H6X POPC 22 -2.616 19.547 10.136 1.00 0.00 MEMB H

ATOM 2917 H6Y POPC 22 -0.987 20.038 9.518 1.00 0.00 MEMB H

ATOM 2918 C37 POPC 22 -2.544 21.537 9.296 1.00 0.00 MEMB C

ATOM 2919 H7X POPC 22 -1.779 22.320 9.085 1.00 0.00 MEMB H

ATOM 2920 H7Y POPC 22 -3.343 22.011 9.910 1.00 0.00 MEMB H

ATOM 2921 C38 POPC 22 -3.098 21.058 7.938 1.00 0.00 MEMB C

ATOM 2922 H8X POPC 22 -3.811 20.217 8.071 1.00 0.00 MEMB H

ATOM 2923 H8Y POPC 22 -2.236 20.662 7.354 1.00 0.00 MEMB H

ATOM 2924 C39 POPC 22 -3.718 22.191 7.109 1.00 0.00 MEMB C

ATOM 2925 H9X POPC 22 -2.937 22.982 7.030 1.00 0.00 MEMB H

ATOM 2926 H9Y POPC 22 -4.600 22.656 7.599 1.00 0.00 MEMB H

ATOM 2927 C310 POPC 22 -4.097 21.800 5.673 1.00 0.00 MEMB C

ATOM 2928 H10X POPC 22 -3.191 21.422 5.152 1.00 0.00 MEMB H

ATOM 2929 H10Y POPC 22 -4.392 22.750 5.172 1.00 0.00 MEMB H

ATOM 2930 C311 POPC 22 -5.264 20.798 5.530 1.00 0.00 MEMB C

ATOM 2931 H11X POPC 22 -6.176 21.296 5.932 1.00 0.00 MEMB H

ATOM 2932 H11Y POPC 22 -5.060 19.887 6.123 1.00 0.00 MEMB H

ATOM 2933 C312 POPC 22 -5.555 20.338 4.092 1.00 0.00 MEMB C

ATOM 2934 H12X POPC 22 -6.504 19.767 4.012 1.00 0.00 MEMB H

ATOM 2935 H12Y POPC 22 -4.720 19.723 3.688 1.00 0.00 MEMB H

ATOM 2936 C313 POPC 22 -5.661 21.509 3.188 1.00 0.00 MEMB C

ATOM 2937 H13X POPC 22 -5.950 22.379 3.827 1.00 0.00 MEMB H

ATOM 2938 H13Y POPC 22 -6.466 21.421 2.417 1.00 0.00 MEMB H

ATOM 2939 C314 POPC 22 -4.311 21.742 2.536 1.00 0.00 MEMB C

ATOM 2940 H14X POPC 22 -3.978 21.008 1.777 1.00 0.00 MEMB H

ATOM 2941 H14Y POPC 22 -3.484 22.023 3.227 1.00 0.00 MEMB H

ATOM 2942 C315 POPC 22 -4.603 22.980 1.893 1.00 0.00 MEMB C

ATOM 2943 H15X POPC 22 -4.580 23.682 2.751 1.00 0.00 MEMB H

ATOM 2944 H15Y POPC 22 -5.586 22.874 1.341 1.00 0.00 MEMB H

ATOM 2945 C316 POPC 22 -3.700 23.406 0.890 1.00 0.00 MEMB C

ATOM 2946 H16X POPC 22 -4.051 24.215 0.220 1.00 0.00 MEMB H

ATOM 2947 H16Y POPC 22 -3.878 22.509 0.252 1.00 0.00 MEMB H

ATOM 2948 H16Z POPC 22 -2.639 23.586 1.170 1.00 0.00 MEMB H

ATOM 2949 N POPC 23 16.970 12.906 17.660 1.00 0.00 MEMB N

ATOM 2950 C12 POPC 23 16.926 12.659 19.154 1.00 0.00 MEMB C

ATOM 2951 H12A POPC 23 17.532 11.792 19.392 1.00 0.00 MEMB H

ATOM 2952 H12B POPC 23 15.903 12.407 19.397 1.00 0.00 MEMB H

ATOM 2953 C13 POPC 23 15.918 13.899 17.287 1.00 0.00 MEMB C

ATOM 2954 H13A POPC 23 14.925 13.496 17.420 1.00 0.00 MEMB H

ATOM 2955 H13B POPC 23 15.997 14.772 17.942 1.00 0.00 MEMB H

ATOM 2956 H13C POPC 23 16.030 14.235 16.268 1.00 0.00 MEMB H

ATOM 2957 C14 POPC 23 18.305 13.450 17.280 1.00 0.00 MEMB C

ATOM 2958 H14A POPC 23 18.504 14.369 17.848 1.00 0.00 MEMB H

ATOM 2959 H14B POPC 23 19.100 12.743 17.466 1.00 0.00 MEMB H

ATOM 2960 H14C POPC 23 18.334 13.736 16.232 1.00 0.00 MEMB H

ATOM 2961 C15 POPC 23 16.699 11.637 16.940 1.00 0.00 MEMB C

ATOM 2962 H15A POPC 23 15.741 11.208 17.233 1.00 0.00 MEMB H

ATOM 2963 H15B POPC 23 17.460 10.899 17.153 1.00 0.00 MEMB H

ATOM 2964 H15C POPC 23 16.708 11.792 15.871 1.00 0.00 MEMB H

ATOM 2965 C11 POPC 23 17.380 13.836 20.054 1.00 0.00 MEMB C

ATOM 2966 H11A POPC 23 18.438 14.078 19.789 1.00 0.00 MEMB H

ATOM 2967 H11B POPC 23 17.399 13.522 21.121 1.00 0.00 MEMB H

ATOM 2968 P POPC 23 17.165 16.392 20.086 1.00 0.00 MEMB P

ATOM 2969 O13 POPC 23 17.980 16.404 21.309 1.00 0.00 MEMB O

ATOM 2970 O14 POPC 23 16.162 17.470 19.936 1.00 0.00 MEMB O

ATOM 2971 O12 POPC 23 16.502 14.962 19.922 1.00 0.00 MEMB O

ATOM 2972 O11 POPC 23 18.129 16.368 18.814 1.00 0.00 MEMB O

ATOM 2973 C1 POPC 23 18.684 17.557 18.240 1.00 0.00 MEMB C

ATOM 2974 HA POPC 23 19.737 17.420 17.929 1.00 0.00 MEMB H

ATOM 2975 HB POPC 23 18.654 18.416 18.954 1.00 0.00 MEMB H

ATOM 2976 C2 POPC 23 17.859 17.943 17.004 1.00 0.00 MEMB C

ATOM 2977 HS POPC 23 16.879 18.061 17.442 1.00 0.00 MEMB H

ATOM 2978 O21 POPC 23 17.900 16.850 16.013 1.00 0.00 MEMB O

ATOM 2979 C21 POPC 23 17.257 17.057 14.809 1.00 0.00 MEMB C

ATOM 2980 O22 POPC 23 16.964 18.151 14.434 1.00 0.00 MEMB O

ATOM 2981 C22 POPC 23 16.836 15.758 14.067 1.00 0.00 MEMB C

ATOM 2982 H2R POPC 23 16.837 14.912 14.776 1.00 0.00 MEMB H

ATOM 2983 H2S POPC 23 17.612 15.644 13.305 1.00 0.00 MEMB H

ATOM 2984 C3 POPC 23 18.322 19.337 16.424 1.00 0.00 MEMB C

ATOM 2985 HX POPC 23 18.353 20.025 17.238 1.00 0.00 MEMB H

ATOM 2986 HY POPC 23 17.527 19.802 15.782 1.00 0.00 MEMB H

ATOM 2987 O31 POPC 23 19.704 19.489 16.095 1.00 0.00 MEMB O

ATOM 2988 C31 POPC 23 20.001 19.779 14.861 1.00 0.00 MEMB C

ATOM 2989 O32 POPC 23 19.122 20.051 14.037 1.00 0.00 MEMB O

ATOM 2990 C32 POPC 23 21.678 19.055 14.575 1.00 0.00 MEMB C

ATOM 2991 H2X POPC 23 22.181 19.266 15.427 1.00 0.00 MEMB H

ATOM 2992 H2Y POPC 23 21.620 20.037 13.972 1.00 0.00 MEMB H

ATOM 2993 C23 POPC 23 15.699 15.653 13.007 1.00 0.00 MEMB C

ATOM 2994 H3R POPC 23 14.728 15.632 13.501 1.00 0.00 MEMB H

ATOM 2995 H3S POPC 23 15.785 14.642 12.551 1.00 0.00 MEMB H

ATOM 2996 C24 POPC 23 15.726 16.629 11.815 1.00 0.00 MEMB C

ATOM 2997 H4R POPC 23 15.773 17.694 12.108 1.00 0.00 MEMB H

ATOM 2998 H4S POPC 23 14.745 16.537 11.303 1.00 0.00 MEMB H

ATOM 2999 C25 POPC 23 16.865 16.374 10.821 1.00 0.00 MEMB C

ATOM 3000 H5R POPC 23 16.860 15.342 10.442 1.00 0.00 MEMB H

ATOM 3001 H5S POPC 23 17.846 16.608 11.310 1.00 0.00 MEMB H

ATOM 3002 C26 POPC 23 16.755 17.244 9.601 1.00 0.00 MEMB C

ATOM 3003 H6R POPC 23 15.696 17.524 9.351 1.00 0.00 MEMB H

ATOM 3004 H6S POPC 23 17.296 16.876 8.722 1.00 0.00 MEMB H

ATOM 3005 C27 POPC 23 17.429 18.495 9.943 1.00 0.00 MEMB C

ATOM 3006 H7R POPC 23 18.452 18.588 10.287 1.00 0.00 MEMB H

ATOM 3007 H7S POPC 23 16.823 18.722 10.788 1.00 0.00 MEMB H

ATOM 3008 C28 POPC 23 17.142 19.484 8.946 1.00 0.00 MEMB C

ATOM 3009 H8R POPC 23 17.627 20.341 9.488 1.00 0.00 MEMB H

ATOM 3010 H8S POPC 23 16.058 19.639 8.828 1.00 0.00 MEMB H

ATOM 3011 C29 POPC 23 17.743 19.144 7.658 1.00 0.00 MEMB C

ATOM 3012 H91 POPC 23 18.817 19.382 7.636 1.00 0.00 MEMB H

ATOM 3013 C210 POPC 23 17.028 19.178 6.535 1.00 0.00 MEMB C

ATOM 3014 H101 POPC 23 17.650 19.153 5.634 1.00 0.00 MEMB H

ATOM 3015 C211 POPC 23 15.554 19.541 6.317 1.00 0.00 MEMB C

ATOM 3016 H11R POPC 23 15.320 20.415 5.671 1.00 0.00 MEMB H

ATOM 3017 H11S POPC 23 14.970 19.874 7.209 1.00 0.00 MEMB H

ATOM 3018 C212 POPC 23 14.819 18.444 5.633 1.00 0.00 MEMB C

ATOM 3019 H12R POPC 23 13.840 18.919 5.363 1.00 0.00 MEMB H

ATOM 3020 H12S POPC 23 14.735 17.754 6.489 1.00 0.00 MEMB H

ATOM 3021 C213 POPC 23 15.457 17.595 4.499 1.00 0.00 MEMB C

ATOM 3022 H13R POPC 23 14.814 16.752 4.247 1.00 0.00 MEMB H

ATOM 3023 H13S POPC 23 16.536 17.382 4.669 1.00 0.00 MEMB H

ATOM 3024 C214 POPC 23 15.474 18.229 3.172 1.00 0.00 MEMB C

ATOM 3025 H14R POPC 23 15.990 18.979 3.770 1.00 0.00 MEMB H

ATOM 3026 H14S POPC 23 14.757 18.924 2.960 1.00 0.00 MEMB H

ATOM 3027 C215 POPC 23 16.077 17.585 1.864 1.00 0.00 MEMB C

ATOM 3028 H15R POPC 23 15.404 16.744 1.625 1.00 0.00 MEMB H

ATOM 3029 H15S POPC 23 17.148 17.353 2.013 1.00 0.00 MEMB H

ATOM 3030 C216 POPC 23 16.175 18.294 0.482 1.00 0.00 MEMB C

ATOM 3031 H16R POPC 23 16.107 19.323 0.608 1.00 0.00 MEMB H

ATOM 3032 H16S POPC 23 15.445 18.823 -0.003 1.00 0.00 MEMB H

ATOM 3033 C217 POPC 23 16.985 17.668 -0.618 1.00 0.00 MEMB C

ATOM 3034 H17R POPC 23 16.402 16.733 -0.765 1.00 0.00 MEMB H

ATOM 3035 H17S POPC 23 18.021 17.728 -0.250 1.00 0.00 MEMB H

ATOM 3036 C218 POPC 23 17.066 18.163 -2.013 1.00 0.00 MEMB C

ATOM 3037 H18R POPC 23 17.797 18.979 -2.283 1.00 0.00 MEMB H

ATOM 3038 H18S POPC 23 16.045 18.335 -2.177 1.00 0.00 MEMB H

ATOM 3039 H18T POPC 23 16.585 17.820 -2.894 1.00 0.00 MEMB H

ATOM 3040 C33 POPC 23 22.308 17.256 13.756 1.00 0.00 MEMB C

ATOM 3041 H3X POPC 23 21.586 16.701 12.897 1.00 0.00 MEMB H

ATOM 3042 H3Y POPC 23 22.260 16.337 14.253 1.00 0.00 MEMB H

ATOM 3043 C34 POPC 23 23.313 16.968 12.588 1.00 0.00 MEMB C

ATOM 3044 H4X POPC 23 24.224 16.881 13.129 1.00 0.00 MEMB H

ATOM 3045 H4Y POPC 23 23.527 17.816 11.945 1.00 0.00 MEMB H

ATOM 3046 C35 POPC 23 23.001 15.938 11.477 1.00 0.00 MEMB C

ATOM 3047 H5X POPC 23 23.889 15.854 10.803 1.00 0.00 MEMB H

ATOM 3048 H5Y POPC 23 22.161 16.199 10.843 1.00 0.00 MEMB H

ATOM 3049 C36 POPC 23 22.054 14.838 11.675 1.00 0.00 MEMB C

ATOM 3050 H6X POPC 23 21.095 14.791 12.206 1.00 0.00 MEMB H

ATOM 3051 H6Y POPC 23 22.756 14.544 12.400 1.00 0.00 MEMB H

ATOM 3052 C37 POPC 23 21.801 14.270 10.336 1.00 0.00 MEMB C

ATOM 3053 H7X POPC 23 21.621 13.292 10.616 1.00 0.00 MEMB H

ATOM 3054 H7Y POPC 23 22.706 14.311 9.673 1.00 0.00 MEMB H

ATOM 3055 C38 POPC 23 20.415 13.925 9.814 1.00 0.00 MEMB C

ATOM 3056 H8X POPC 23 19.661 14.148 10.587 1.00 0.00 MEMB H

ATOM 3057 H8Y POPC 23 20.222 12.865 9.493 1.00 0.00 MEMB H

ATOM 3058 C39 POPC 23 20.502 14.693 8.590 1.00 0.00 MEMB C

ATOM 3059 H9X POPC 23 21.523 14.642 8.128 1.00 0.00 MEMB H

ATOM 3060 H9Y POPC 23 20.335 15.659 9.111 1.00 0.00 MEMB H

ATOM 3061 C310 POPC 23 19.473 14.421 7.582 1.00 0.00 MEMB C

ATOM 3062 H10X POPC 23 18.652 13.935 8.151 1.00 0.00 MEMB H

ATOM 3063 H10Y POPC 23 19.836 13.752 6.780 1.00 0.00 MEMB H

ATOM 3064 C311 POPC 23 19.093 15.743 6.975 1.00 0.00 MEMB C

ATOM 3065 H11X POPC 23 19.835 16.077 6.225 1.00 0.00 MEMB H

ATOM 3066 H11Y POPC 23 19.088 16.528 7.774 1.00 0.00 MEMB H

ATOM 3067 C312 POPC 23 17.705 15.657 6.384 1.00 0.00 MEMB C

ATOM 3068 H12X POPC 23 17.506 16.695 6.097 1.00 0.00 MEMB H

ATOM 3069 H12Y POPC 23 17.017 15.372 7.210 1.00 0.00 MEMB H

ATOM 3070 C313 POPC 23 17.444 14.709 5.197 1.00 0.00 MEMB C

ATOM 3071 H13X POPC 23 16.394 14.908 4.883 1.00 0.00 MEMB H

ATOM 3072 H13Y POPC 23 17.489 13.649 5.525 1.00 0.00 MEMB H

ATOM 3073 C314 POPC 23 18.357 14.921 3.983 1.00 0.00 MEMB C

ATOM 3074 H14X POPC 23 19.415 14.773 4.303 1.00 0.00 MEMB H

ATOM 3075 H14Y POPC 23 18.259 15.972 3.636 1.00 0.00 MEMB H

ATOM 3076 C315 POPC 23 18.121 13.966 2.793 1.00 0.00 MEMB C

ATOM 3077 H15X POPC 23 18.230 12.910 3.126 1.00 0.00 MEMB H

ATOM 3078 H15Y POPC 23 18.957 14.187 2.088 1.00 0.00 MEMB H

ATOM 3079 C316 POPC 23 16.813 14.083 2.000 1.00 0.00 MEMB C

ATOM 3080 H16X POPC 23 16.468 15.127 1.942 1.00 0.00 MEMB H

ATOM 3081 H16Y POPC 23 15.994 13.478 2.440 1.00 0.00 MEMB H

ATOM 3082 H16Z POPC 23 16.983 13.718 0.963 1.00 0.00 MEMB H

ATOM 3083 N POPC 24 -23.722 1.085 18.866 1.00 0.00 MEMB N

ATOM 3084 C12 POPC 24 -22.801 0.370 19.837 1.00 0.00 MEMB C

ATOM 3085 H12A POPC 24 -23.345 0.066 20.732 1.00 0.00 MEMB H

ATOM 3086 H12B POPC 24 -21.993 1.017 20.130 1.00 0.00 MEMB H

ATOM 3087 C13 POPC 24 -22.940 1.593 17.704 1.00 0.00 MEMB C

ATOM 3088 H13A POPC 24 -23.602 2.061 16.991 1.00 0.00 MEMB H

ATOM 3089 H13B POPC 24 -22.193 2.305 18.037 1.00 0.00 MEMB H

ATOM 3090 H13C POPC 24 -22.420 0.777 17.209 1.00 0.00 MEMB H

ATOM 3091 C14 POPC 24 -24.760 0.134 18.377 1.00 0.00 MEMB C

ATOM 3092 H14A POPC 24 -25.343 -0.243 19.207 1.00 0.00 MEMB H

ATOM 3093 H14B POPC 24 -25.416 0.543 17.626 1.00 0.00 MEMB H

ATOM 3094 H14C POPC 24 -24.274 -0.758 17.969 1.00 0.00 MEMB H

ATOM 3095 C15 POPC 24 -24.400 2.234 19.523 1.00 0.00 MEMB C

ATOM 3096 H15A POPC 24 -25.059 1.913 20.332 1.00 0.00 MEMB H

ATOM 3097 H15B POPC 24 -25.001 2.752 18.787 1.00 0.00 MEMB H

ATOM 3098 H15C POPC 24 -23.643 2.901 19.893 1.00 0.00 MEMB H

ATOM 3099 C11 POPC 24 -22.214 -0.946 19.355 1.00 0.00 MEMB C

ATOM 3100 H11A POPC 24 -21.386 -1.221 20.049 1.00 0.00 MEMB H

ATOM 3101 H11B POPC 24 -21.741 -0.820 18.343 1.00 0.00 MEMB H

ATOM 3102 P POPC 24 -22.854 -3.394 19.200 1.00 0.00 MEMB P

ATOM 3103 O13 POPC 24 -23.917 -4.404 18.981 1.00 0.00 MEMB O

ATOM 3104 O14 POPC 24 -21.819 -3.712 20.212 1.00 0.00 MEMB O

ATOM 3105 O12 POPC 24 -23.294 -1.911 19.426 1.00 0.00 MEMB O

ATOM 3106 O11 POPC 24 -22.224 -2.986 17.836 1.00 0.00 MEMB O

ATOM 3107 C1 POPC 24 -22.383 -3.502 16.566 1.00 0.00 MEMB C

ATOM 3108 HA POPC 24 -23.445 -3.582 16.265 1.00 0.00 MEMB H

ATOM 3109 HB POPC 24 -22.061 -4.560 16.585 1.00 0.00 MEMB H

ATOM 3110 C2 POPC 24 -21.463 -2.695 15.540 1.00 0.00 MEMB C

ATOM 3111 HS POPC 24 -20.407 -3.082 15.689 1.00 0.00 MEMB H

ATOM 3112 O21 POPC 24 -21.369 -1.329 15.573 1.00 0.00 MEMB O

ATOM 3113 C21 POPC 24 -20.547 -0.884 14.609 1.00 0.00 MEMB C

ATOM 3114 O22 POPC 24 -19.717 -1.468 13.937 1.00 0.00 MEMB O

ATOM 3115 C22 POPC 24 -20.817 0.519 14.728 1.00 0.00 MEMB C

ATOM 3116 H2R POPC 24 -20.493 0.692 15.783 1.00 0.00 MEMB H

ATOM 3117 H2S POPC 24 -21.910 0.686 14.597 1.00 0.00 MEMB H

ATOM 3118 C3 POPC 24 -21.927 -2.876 14.176 1.00 0.00 MEMB C

ATOM 3119 HX POPC 24 -21.667 -3.791 14.671 1.00 0.00 MEMB H

ATOM 3120 HY POPC 24 -21.250 -2.863 13.288 1.00 0.00 MEMB H

ATOM 3121 O31 POPC 24 -23.346 -2.796 13.972 1.00 0.00 MEMB O

ATOM 3122 C31 POPC 24 -23.686 -2.403 12.779 1.00 0.00 MEMB C

ATOM 3123 O32 POPC 24 -23.001 -1.742 12.022 1.00 0.00 MEMB O

ATOM 3124 C32 POPC 24 -25.086 -2.935 12.498 1.00 0.00 MEMB C

ATOM 3125 H2X POPC 24 -25.623 -3.218 13.410 1.00 0.00 MEMB H

ATOM 3126 H2Y POPC 24 -25.319 -3.731 11.777 1.00 0.00 MEMB H

ATOM 3127 C23 POPC 24 -19.977 1.257 13.747 1.00 0.00 MEMB C

ATOM 3128 H3R POPC 24 -19.063 0.735 13.405 1.00 0.00 MEMB H

ATOM 3129 H3S POPC 24 -19.700 2.181 14.309 1.00 0.00 MEMB H

ATOM 3130 C24 POPC 24 -20.732 1.654 12.515 1.00 0.00 MEMB C

ATOM 3131 H4R POPC 24 -20.212 2.487 12.120 1.00 0.00 MEMB H

ATOM 3132 H4S POPC 24 -21.650 2.121 12.845 1.00 0.00 MEMB H

ATOM 3133 C25 POPC 24 -20.863 0.642 11.380 1.00 0.00 MEMB C

ATOM 3134 H5R POPC 24 -21.583 1.011 10.618 1.00 0.00 MEMB H

ATOM 3135 H5S POPC 24 -21.261 -0.294 11.798 1.00 0.00 MEMB H

ATOM 3136 C26 POPC 24 -19.568 0.323 10.651 1.00 0.00 MEMB C

ATOM 3137 H6R POPC 24 -18.800 -0.022 11.380 1.00 0.00 MEMB H

ATOM 3138 H6S POPC 24 -19.185 1.221 10.135 1.00 0.00 MEMB H

ATOM 3139 C27 POPC 24 -19.767 -0.801 9.641 1.00 0.00 MEMB C

ATOM 3140 H7R POPC 24 -18.775 -0.844 9.153 1.00 0.00 MEMB H

ATOM 3141 H7S POPC 24 -20.481 -0.540 8.837 1.00 0.00 MEMB H

ATOM 3142 C28 POPC 24 -20.220 -2.095 10.359 1.00 0.00 MEMB C

ATOM 3143 H8R POPC 24 -21.249 -2.071 10.697 1.00 0.00 MEMB H

ATOM 3144 H8S POPC 24 -19.571 -2.191 11.259 1.00 0.00 MEMB H

ATOM 3145 C29 POPC 24 -20.100 -3.316 9.554 1.00 0.00 MEMB C

ATOM 3146 H91 POPC 24 -19.453 -4.071 10.039 1.00 0.00 MEMB H

ATOM 3147 C210 POPC 24 -20.633 -3.633 8.374 1.00 0.00 MEMB C

ATOM 3148 H101 POPC 24 -20.004 -4.300 7.749 1.00 0.00 MEMB H

ATOM 3149 C211 POPC 24 -21.550 -2.836 7.541 1.00 0.00 MEMB C

ATOM 3150 H11R POPC 24 -21.076 -2.545 6.586 1.00 0.00 MEMB H

ATOM 3151 H11S POPC 24 -21.834 -1.952 8.137 1.00 0.00 MEMB H

ATOM 3152 C212 POPC 24 -22.835 -3.621 7.250 1.00 0.00 MEMB C

ATOM 3153 H12R POPC 24 -23.662 -2.923 7.093 1.00 0.00 MEMB H

ATOM 3154 H12S POPC 24 -23.242 -4.180 8.046 1.00 0.00 MEMB H

ATOM 3155 C213 POPC 24 -22.790 -4.599 6.109 1.00 0.00 MEMB C

ATOM 3156 H13R POPC 24 -23.260 -5.566 6.409 1.00 0.00 MEMB H

ATOM 3157 H13S POPC 24 -21.729 -4.834 5.880 1.00 0.00 MEMB H

ATOM 3158 C214 POPC 24 -23.610 -4.039 4.938 1.00 0.00 MEMB C

ATOM 3159 H14R POPC 24 -23.176 -3.063 4.628 1.00 0.00 MEMB H

ATOM 3160 H14S POPC 24 -24.685 -3.956 5.198 1.00 0.00 MEMB H

ATOM 3161 C215 POPC 24 -23.461 -5.019 3.817 1.00 0.00 MEMB C

ATOM 3162 H15R POPC 24 -23.392 -5.986 4.295 1.00 0.00 MEMB H

ATOM 3163 H15S POPC 24 -22.434 -4.831 3.606 1.00 0.00 MEMB H

ATOM 3164 C216 POPC 24 -24.188 -4.906 2.472 1.00 0.00 MEMB C

ATOM 3165 H16R POPC 24 -23.908 -5.750 1.798 1.00 0.00 MEMB H

ATOM 3166 H16S POPC 24 -23.864 -3.967 1.962 1.00 0.00 MEMB H

ATOM 3167 C217 POPC 24 -25.685 -5.024 2.550 1.00 0.00 MEMB C

ATOM 3168 H17R POPC 24 -25.996 -4.525 3.488 1.00 0.00 MEMB H

ATOM 3169 H17S POPC 24 -25.966 -6.104 2.591 1.00 0.00 MEMB H

ATOM 3170 C218 POPC 24 -26.376 -4.361 1.374 1.00 0.00 MEMB C

ATOM 3171 H18R POPC 24 -26.283 -4.982 0.457 1.00 0.00 MEMB H

ATOM 3172 H18S POPC 24 -25.934 -3.357 1.179 1.00 0.00 MEMB H

ATOM 3173 H18T POPC 24 -27.450 -4.248 1.616 1.00 0.00 MEMB H

ATOM 3174 C33 POPC 24 -25.643 -1.842 11.738 1.00 0.00 MEMB C

ATOM 3175 H3X POPC 24 -25.014 -1.231 11.037 1.00 0.00 MEMB H

ATOM 3176 H3Y POPC 24 -25.990 -1.336 12.665 1.00 0.00 MEMB H

ATOM 3177 C34 POPC 24 -26.835 -2.193 10.977 1.00 0.00 MEMB C

ATOM 3178 H4X POPC 24 -27.101 -1.194 10.580 1.00 0.00 MEMB H

ATOM 3179 H4Y POPC 24 -27.615 -2.617 11.653 1.00 0.00 MEMB H

ATOM 3180 C35 POPC 24 -26.743 -2.973 9.731 1.00 0.00 MEMB C

ATOM 3181 H5X POPC 24 -27.740 -2.662 9.399 1.00 0.00 MEMB H

ATOM 3182 H5Y POPC 24 -26.687 -4.055 9.968 1.00 0.00 MEMB H

ATOM 3183 C36 POPC 24 -25.935 -2.450 8.584 1.00 0.00 MEMB C

ATOM 3184 H6X POPC 24 -26.082 -2.976 7.622 1.00 0.00 MEMB H

ATOM 3185 H6Y POPC 24 -24.908 -2.713 8.857 1.00 0.00 MEMB H

ATOM 3186 C37 POPC 24 -26.245 -1.001 8.203 1.00 0.00 MEMB C

ATOM 3187 H7X POPC 24 -26.163 -0.319 9.070 1.00 0.00 MEMB H

ATOM 3188 H7Y POPC 24 -27.281 -0.851 7.828 1.00 0.00 MEMB H

ATOM 3189 C38 POPC 24 -25.198 -0.662 7.160 1.00 0.00 MEMB C

ATOM 3190 H8X POPC 24 -25.367 -1.244 6.227 1.00 0.00 MEMB H

ATOM 3191 H8Y POPC 24 -24.250 -1.020 7.628 1.00 0.00 MEMB H

ATOM 3192 C39 POPC 24 -25.091 0.822 6.876 1.00 0.00 MEMB C

ATOM 3193 H9X POPC 24 -25.106 1.362 7.841 1.00 0.00 MEMB H

ATOM 3194 H9Y POPC 24 -25.964 1.155 6.276 1.00 0.00 MEMB H

ATOM 3195 C310 POPC 24 -23.797 1.207 6.176 1.00 0.00 MEMB C

ATOM 3196 H10X POPC 24 -22.914 0.853 6.757 1.00 0.00 MEMB H

ATOM 3197 H10Y POPC 24 -23.764 2.322 6.141 1.00 0.00 MEMB H

ATOM 3198 C311 POPC 24 -23.693 0.663 4.762 1.00 0.00 MEMB C

ATOM 3199 H11X POPC 24 -24.583 0.936 4.153 1.00 0.00 MEMB H

ATOM 3200 H11Y POPC 24 -23.611 -0.446 4.735 1.00 0.00 MEMB H

ATOM 3201 C312 POPC 24 -22.474 1.242 4.086 1.00 0.00 MEMB C

ATOM 3202 H12X POPC 24 -21.544 0.969 4.632 1.00 0.00 MEMB H

ATOM 3203 H12Y POPC 24 -22.551 2.356 4.139 1.00 0.00 MEMB H

ATOM 3204 C313 POPC 24 -22.447 0.663 2.674 1.00 0.00 MEMB C

ATOM 3205 H13X POPC 24 -23.384 0.863 2.123 1.00 0.00 MEMB H

ATOM 3206 H13Y POPC 24 -22.093 -0.383 2.631 1.00 0.00 MEMB H

ATOM 3207 C314 POPC 24 -21.469 1.343 1.853 1.00 0.00 MEMB C

ATOM 3208 H14X POPC 24 -20.488 1.246 2.350 1.00 0.00 MEMB H

ATOM 3209 H14Y POPC 24 -21.827 2.386 2.037 1.00 0.00 MEMB H

ATOM 3210 C315 POPC 24 -21.461 1.132 0.300 1.00 0.00 MEMB C

ATOM 3211 H15X POPC 24 -20.658 1.619 -0.283 1.00 0.00 MEMB H

ATOM 3212 H15Y POPC 24 -22.256 1.703 -0.212 1.00 0.00 MEMB H

ATOM 3213 C316 POPC 24 -20.885 -0.153 -0.156 1.00 0.00 MEMB C

ATOM 3214 H16X POPC 24 -19.872 -0.235 0.327 1.00 0.00 MEMB H

ATOM 3215 H16Y POPC 24 -20.747 -0.112 -1.266 1.00 0.00 MEMB H

ATOM 3216 H16Z POPC 24 -21.599 -0.905 0.199 1.00 0.00 MEMB H

ATOM 3217 N POPC 25 10.888 21.350 20.921 1.00 0.00 MEMB N

ATOM 3218 C12 POPC 25 10.443 22.006 19.613 1.00 0.00 MEMB C

ATOM 3219 H12A POPC 25 11.032 22.893 19.446 1.00 0.00 MEMB H

ATOM 3220 H12B POPC 25 9.418 22.316 19.751 1.00 0.00 MEMB H

ATOM 3221 C13 POPC 25 10.073 20.112 21.197 1.00 0.00 MEMB C

ATOM 3222 H13A POPC 25 10.220 19.730 22.199 1.00 0.00 MEMB H

ATOM 3223 H13B POPC 25 9.023 20.300 21.053 1.00 0.00 MEMB H

ATOM 3224 H13C POPC 25 10.344 19.336 20.478 1.00 0.00 MEMB H

ATOM 3225 C14 POPC 25 12.343 20.997 20.897 1.00 0.00 MEMB C

ATOM 3226 H14A POPC 25 12.941 21.794 20.489 1.00 0.00 MEMB H

ATOM 3227 H14B POPC 25 12.710 20.749 21.886 1.00 0.00 MEMB H

ATOM 3228 H14C POPC 25 12.506 20.141 20.246 1.00 0.00 MEMB H

ATOM 3229 C15 POPC 25 10.696 22.315 22.040 1.00 0.00 MEMB C

ATOM 3230 H15A POPC 25 9.660 22.585 22.171 1.00 0.00 MEMB H

ATOM 3231 H15B POPC 25 11.275 23.208 21.854 1.00 0.00 MEMB H

ATOM 3232 H15C POPC 25 11.029 21.848 22.963 1.00 0.00 MEMB H

ATOM 3233 C11 POPC 25 10.459 21.272 18.245 1.00 0.00 MEMB C

ATOM 3234 H11A POPC 25 11.043 21.901 17.531 1.00 0.00 MEMB H

ATOM 3235 H11B POPC 25 9.405 21.228 17.880 1.00 0.00 MEMB H

ATOM 3236 P POPC 25 10.783 18.937 17.143 1.00 0.00 MEMB P

ATOM 3237 O13 POPC 25 10.053 19.533 16.005 1.00 0.00 MEMB O

ATOM 3238 O14 POPC 25 10.248 17.699 17.761 1.00 0.00 MEMB O

ATOM 3239 O12 POPC 25 11.009 19.977 18.322 1.00 0.00 MEMB O

ATOM 3240 O11 POPC 25 12.270 18.704 16.723 1.00 0.00 MEMB O

ATOM 3241 C1 POPC 25 13.188 18.482 17.785 1.00 0.00 MEMB C

ATOM 3242 HA POPC 25 13.085 17.450 18.209 1.00 0.00 MEMB H

ATOM 3243 HB POPC 25 13.178 19.238 18.599 1.00 0.00 MEMB H

ATOM 3244 C2 POPC 25 14.558 18.492 17.210 1.00 0.00 MEMB C

ATOM 3245 HS POPC 25 15.248 18.548 18.076 1.00 0.00 MEMB H

ATOM 3246 O21 POPC 25 14.894 19.558 16.324 1.00 0.00 MEMB O

ATOM 3247 C21 POPC 25 14.739 20.750 16.802 1.00 0.00 MEMB C

ATOM 3248 O22 POPC 25 14.736 21.080 17.974 1.00 0.00 MEMB O

ATOM 3249 C22 POPC 25 14.705 21.740 15.703 1.00 0.00 MEMB C

ATOM 3250 H2R POPC 25 15.738 21.695 15.320 1.00 0.00 MEMB H

ATOM 3251 H2S POPC 25 14.698 22.749 16.127 1.00 0.00 MEMB H

ATOM 3252 C3 POPC 25 14.686 17.145 16.484 1.00 0.00 MEMB C

ATOM 3253 HX POPC 25 14.741 16.271 17.157 1.00 0.00 MEMB H

ATOM 3254 HY POPC 25 15.673 17.209 16.236 1.00 0.00 MEMB H

ATOM 3255 O31 POPC 25 13.869 16.901 15.323 1.00 0.00 MEMB O

ATOM 3256 C31 POPC 25 13.827 17.708 14.264 1.00 0.00 MEMB C

ATOM 3257 O32 POPC 25 14.602 18.563 13.921 1.00 0.00 MEMB O

ATOM 3258 C32 POPC 25 12.703 17.293 13.364 1.00 0.00 MEMB C

ATOM 3259 H2X POPC 25 11.835 17.072 14.011 1.00 0.00 MEMB H

ATOM 3260 H2Y POPC 25 12.994 16.389 12.791 1.00 0.00 MEMB H

ATOM 3261 C23 POPC 25 13.431 21.433 14.822 1.00 0.00 MEMB C

ATOM 3262 H3R POPC 25 12.545 21.544 15.462 1.00 0.00 MEMB H

ATOM 3263 H3S POPC 25 13.544 20.397 14.461 1.00 0.00 MEMB H

ATOM 3264 C24 POPC 25 13.203 22.227 13.527 1.00 0.00 MEMB C

ATOM 3265 H4R POPC 25 13.934 21.696 12.873 1.00 0.00 MEMB H

ATOM 3266 H4S POPC 25 13.349 23.280 13.846 1.00 0.00 MEMB H

ATOM 3267 C25 POPC 25 12.021 22.476 12.595 1.00 0.00 MEMB C

ATOM 3268 H5R POPC 25 11.185 22.713 13.275 1.00 0.00 MEMB H

ATOM 3269 H5S POPC 25 11.863 21.564 11.984 1.00 0.00 MEMB H

ATOM 3270 C26 POPC 25 12.187 23.711 11.619 1.00 0.00 MEMB C

ATOM 3271 H6R POPC 25 12.473 24.638 12.150 1.00 0.00 MEMB H

ATOM 3272 H6S POPC 25 11.192 23.909 11.178 1.00 0.00 MEMB H

ATOM 3273 C27 POPC 25 13.159 23.492 10.447 1.00 0.00 MEMB C

ATOM 3274 H7R POPC 25 12.976 22.469 10.068 1.00 0.00 MEMB H

ATOM 3275 H7S POPC 25 14.183 23.565 10.800 1.00 0.00 MEMB H

ATOM 3276 C28 POPC 25 13.390 24.545 9.356 1.00 0.00 MEMB C

ATOM 3277 H8R POPC 25 14.385 24.353 8.889 1.00 0.00 MEMB H

ATOM 3278 H8S POPC 25 13.514 25.541 9.829 1.00 0.00 MEMB H

ATOM 3279 C29 POPC 25 12.313 24.461 8.325 1.00 0.00 MEMB C

ATOM 3280 H91 POPC 25 11.472 25.131 8.485 1.00 0.00 MEMB H

ATOM 3281 C210 POPC 25 12.297 23.629 7.281 1.00 0.00 MEMB C

ATOM 3282 H101 POPC 25 11.486 23.672 6.542 1.00 0.00 MEMB H

ATOM 3283 C211 POPC 25 13.370 22.649 6.980 1.00 0.00 MEMB C

ATOM 3284 H11R POPC 25 13.046 21.584 6.864 1.00 0.00 MEMB H

ATOM 3285 H11S POPC 25 14.201 22.676 7.707 1.00 0.00 MEMB H

ATOM 3286 C212 POPC 25 13.897 23.053 5.643 1.00 0.00 MEMB C

ATOM 3287 H12R POPC 25 14.647 22.260 5.628 1.00 0.00 MEMB H

ATOM 3288 H12S POPC 25 14.474 24.011 5.573 1.00 0.00 MEMB H

ATOM 3289 C213 POPC 25 12.952 22.667 4.509 1.00 0.00 MEMB C

ATOM 3290 H13R POPC 25 11.929 23.010 4.619 1.00 0.00 MEMB H

ATOM 3291 H13S POPC 25 13.049 21.573 4.313 1.00 0.00 MEMB H

ATOM 3292 C214 POPC 25 13.206 23.336 3.268 1.00 0.00 MEMB C

ATOM 3293 H14R POPC 25 13.266 24.496 3.358 1.00 0.00 MEMB H

ATOM 3294 H14S POPC 25 12.843 22.956 2.297 1.00 0.00 MEMB H

ATOM 3295 C215 POPC 25 14.458 22.897 2.997 1.00 0.00 MEMB C

ATOM 3296 H15R POPC 25 14.972 23.730 3.587 1.00 0.00 MEMB H

ATOM 3297 H15S POPC 25 14.484 23.712 2.186 1.00 0.00 MEMB H

ATOM 3298 C216 POPC 25 15.171 21.187 2.875 1.00 0.00 MEMB C

ATOM 3299 H16R POPC 25 14.500 20.898 2.115 1.00 0.00 MEMB H

ATOM 3300 H16S POPC 25 14.813 20.719 3.773 1.00 0.00 MEMB H

ATOM 3301 C217 POPC 25 16.888 20.366 2.355 1.00 0.00 MEMB C

ATOM 3302 H17R POPC 25 17.261 19.423 2.722 1.00 0.00 MEMB H

ATOM 3303 H17S POPC 25 17.663 20.695 2.853 1.00 0.00 MEMB H

ATOM 3304 C218 POPC 25 18.048 20.187 1.152 1.00 0.00 MEMB C

ATOM 3305 H18R POPC 25 17.507 20.338 0.363 1.00 0.00 MEMB H

ATOM 3306 H18S POPC 25 18.947 19.561 0.996 1.00 0.00 MEMB H

ATOM 3307 H18T POPC 25 18.479 21.001 0.646 1.00 0.00 MEMB H

ATOM 3308 C33 POPC 25 12.465 18.474 12.406 1.00 0.00 MEMB C

ATOM 3309 H3X POPC 25 13.410 18.869 11.980 1.00 0.00 MEMB H

ATOM 3310 H3Y POPC 25 12.013 19.299 13.005 1.00 0.00 MEMB H

ATOM 3311 C34 POPC 25 11.631 18.053 11.203 1.00 0.00 MEMB C

ATOM 3312 H4X POPC 25 10.590 17.967 11.580 1.00 0.00 MEMB H

ATOM 3313 H4Y POPC 25 11.986 17.051 10.871 1.00 0.00 MEMB H

ATOM 3314 C35 POPC 25 11.709 18.999 9.995 1.00 0.00 MEMB C

ATOM 3315 H5X POPC 25 12.711 18.940 9.512 1.00 0.00 MEMB H

ATOM 3316 H5Y POPC 25 11.597 20.040 10.375 1.00 0.00 MEMB H

ATOM 3317 C36 POPC 25 10.646 18.680 8.920 1.00 0.00 MEMB C

ATOM 3318 H6X POPC 25 10.715 19.422 8.092 1.00 0.00 MEMB H

ATOM 3319 H6Y POPC 25 9.642 18.806 9.387 1.00 0.00 MEMB H

ATOM 3320 C37 POPC 25 10.754 17.265 8.295 1.00 0.00 MEMB C

ATOM 3321 H7X POPC 25 9.917 17.124 7.574 1.00 0.00 MEMB H

ATOM 3322 H7Y POPC 25 10.635 16.505 9.096 1.00 0.00 MEMB H

ATOM 3323 C38 POPC 25 12.088 17.018 7.566 1.00 0.00 MEMB C

ATOM 3324 H8X POPC 25 12.870 16.795 8.325 1.00 0.00 MEMB H

ATOM 3325 H8Y POPC 25 12.375 17.971 7.071 1.00 0.00 MEMB H

ATOM 3326 C39 POPC 25 12.126 15.897 6.503 1.00 0.00 MEMB C

ATOM 3327 H9X POPC 25 11.284 15.178 6.570 1.00 0.00 MEMB H

ATOM 3328 H9Y POPC 25 13.072 15.333 6.672 1.00 0.00 MEMB H

ATOM 3329 C310 POPC 25 12.192 16.457 5.080 1.00 0.00 MEMB C

ATOM 3330 H10X POPC 25 12.715 15.756 4.393 1.00 0.00 MEMB H

ATOM 3331 H10Y POPC 25 12.845 17.329 5.119 1.00 0.00 MEMB H

ATOM 3332 C311 POPC 25 10.796 16.852 4.564 1.00 0.00 MEMB C

ATOM 3333 H11X POPC 25 10.308 17.566 5.265 1.00 0.00 MEMB H

ATOM 3334 H11Y POPC 25 10.228 15.933 4.358 1.00 0.00 MEMB H

ATOM 3335 C312 POPC 25 10.826 17.586 3.266 1.00 0.00 MEMB C

ATOM 3336 H12X POPC 25 11.383 16.917 2.557 1.00 0.00 MEMB H

ATOM 3337 H12Y POPC 25 11.354 18.470 3.692 1.00 0.00 MEMB H

ATOM 3338 C313 POPC 25 9.597 18.217 2.584 1.00 0.00 MEMB C

ATOM 3339 H13X POPC 25 9.328 19.168 3.093 1.00 0.00 MEMB H

ATOM 3340 H13Y POPC 25 8.870 17.413 2.772 1.00 0.00 MEMB H

ATOM 3341 C314 POPC 25 9.549 18.413 1.039 1.00 0.00 MEMB C

ATOM 3342 H14X POPC 25 8.517 18.450 0.659 1.00 0.00 MEMB H

ATOM 3343 H14Y POPC 25 10.099 17.553 0.579 1.00 0.00 MEMB H

ATOM 3344 C315 POPC 25 10.090 19.716 0.466 1.00 0.00 MEMB C

ATOM 3345 H15X POPC 25 11.021 19.310 0.646 1.00 0.00 MEMB H

ATOM 3346 H15Y POPC 25 10.196 20.632 1.064 1.00 0.00 MEMB H

ATOM 3347 C316 POPC 25 10.099 19.951 -1.039 1.00 0.00 MEMB C

ATOM 3348 H16X POPC 25 10.842 20.762 -1.219 1.00 0.00 MEMB H

ATOM 3349 H16Y POPC 25 9.166 20.316 -1.450 1.00 0.00 MEMB H

ATOM 3350 H16Z POPC 25 10.514 19.072 -1.605 1.00 0.00 MEMB H

ATOM 3351 N POPC 26 17.082 -10.451 19.039 1.00 0.00 MEMB N

ATOM 3352 C12 POPC 26 18.058 -11.625 18.933 1.00 0.00 MEMB C

ATOM 3353 H12A POPC 26 17.620 -12.386 18.297 1.00 0.00 MEMB H

ATOM 3354 H12B POPC 26 18.188 -12.035 19.926 1.00 0.00 MEMB H

ATOM 3355 C13 POPC 26 16.907 -9.823 17.697 1.00 0.00 MEMB C

ATOM 3356 H13A POPC 26 16.256 -10.415 17.063 1.00 0.00 MEMB H

ATOM 3357 H13B POPC 26 16.518 -8.815 17.775 1.00 0.00 MEMB H

ATOM 3358 H13C POPC 26 17.879 -9.785 17.202 1.00 0.00 MEMB H

ATOM 3359 C14 POPC 26 15.759 -10.981 19.504 1.00 0.00 MEMB C

ATOM 3360 H14A POPC 26 15.378 -11.709 18.793 1.00 0.00 MEMB H

ATOM 3361 H14B POPC 26 15.839 -11.481 20.463 1.00 0.00 MEMB H

ATOM 3362 H14C POPC 26 15.042 -10.182 19.583 1.00 0.00 MEMB H

ATOM 3363 C15 POPC 26 17.651 -9.461 20.013 1.00 0.00 MEMB C

ATOM 3364 H15A POPC 26 17.863 -9.962 20.951 1.00 0.00 MEMB H

ATOM 3365 H15B POPC 26 17.016 -8.611 20.219 1.00 0.00 MEMB H

ATOM 3366 H15C POPC 26 18.634 -9.128 19.671 1.00 0.00 MEMB H

ATOM 3367 C11 POPC 26 19.499 -11.388 18.405 1.00 0.00 MEMB C

ATOM 3368 H11A POPC 26 19.443 -10.750 17.493 1.00 0.00 MEMB H

ATOM 3369 H11B POPC 26 19.880 -12.386 18.081 1.00 0.00 MEMB H

ATOM 3370 P POPC 26 21.727 -10.108 19.043 1.00 0.00 MEMB P

ATOM 3371 O13 POPC 26 22.743 -11.085 18.583 1.00 0.00 MEMB O

ATOM 3372 O14 POPC 26 22.081 -9.168 20.138 1.00 0.00 MEMB O

ATOM 3373 O12 POPC 26 20.358 -10.857 19.427 1.00 0.00 MEMB O

ATOM 3374 O11 POPC 26 21.171 -9.256 17.836 1.00 0.00 MEMB O

ATOM 3375 C1 POPC 26 21.904 -9.043 16.636 1.00 0.00 MEMB C

ATOM 3376 HA POPC 26 21.945 -9.981 16.033 1.00 0.00 MEMB H

ATOM 3377 HB POPC 26 22.941 -8.725 16.870 1.00 0.00 MEMB H

ATOM 3378 C2 POPC 26 21.195 -7.956 15.786 1.00 0.00 MEMB C

ATOM 3379 HS POPC 26 21.230 -7.006 16.365 1.00 0.00 MEMB H

ATOM 3380 O21 POPC 26 19.832 -8.384 15.596 1.00 0.00 MEMB O

ATOM 3381 C21 POPC 26 18.981 -7.505 15.127 1.00 0.00 MEMB C

ATOM 3382 O22 POPC 26 19.161 -6.303 15.032 1.00 0.00 MEMB O

ATOM 3383 C22 POPC 26 17.683 -8.240 14.766 1.00 0.00 MEMB C

ATOM 3384 H2R POPC 26 16.859 -7.768 15.333 1.00 0.00 MEMB H

ATOM 3385 H2S POPC 26 17.760 -9.304 15.078 1.00 0.00 MEMB H

ATOM 3386 C3 POPC 26 21.887 -7.691 14.418 1.00 0.00 MEMB C

ATOM 3387 HX POPC 26 22.984 -7.570 14.579 1.00 0.00 MEMB H

ATOM 3388 HY POPC 26 21.485 -6.750 13.984 1.00 0.00 MEMB H

ATOM 3389 O31 POPC 26 21.571 -8.776 13.551 1.00 0.00 MEMB O

ATOM 3390 C31 POPC 26 22.464 -9.209 12.724 1.00 0.00 MEMB C

ATOM 3391 O32 POPC 26 23.559 -8.719 12.488 1.00 0.00 MEMB O

ATOM 3392 C32 POPC 26 21.896 -10.518 12.181 1.00 0.00 MEMB C

ATOM 3393 H2X POPC 26 20.818 -10.398 11.955 1.00 0.00 MEMB H

ATOM 3394 H2Y POPC 26 22.137 -11.336 12.884 1.00 0.00 MEMB H

ATOM 3395 C23 POPC 26 17.406 -8.187 13.252 1.00 0.00 MEMB C

ATOM 3396 H3R POPC 26 17.655 -7.176 12.858 1.00 0.00 MEMB H

ATOM 3397 H3S POPC 26 16.317 -8.316 13.066 1.00 0.00 MEMB H

ATOM 3398 C24 POPC 26 18.210 -9.279 12.526 1.00 0.00 MEMB C

ATOM 3399 H4R POPC 26 18.074 -10.267 13.027 1.00 0.00 MEMB H

ATOM 3400 H4S POPC 26 19.284 -9.011 12.600 1.00 0.00 MEMB H

ATOM 3401 C25 POPC 26 17.919 -9.422 11.036 1.00 0.00 MEMB C

ATOM 3402 H5R POPC 26 18.517 -10.245 10.604 1.00 0.00 MEMB H

ATOM 3403 H5S POPC 26 18.142 -8.484 10.491 1.00 0.00 MEMB H

ATOM 3404 C26 POPC 26 16.511 -9.811 10.731 1.00 0.00 MEMB C

ATOM 3405 H6R POPC 26 15.777 -9.114 11.189 1.00 0.00 MEMB H

ATOM 3406 H6S POPC 26 16.306 -10.742 11.235 1.00 0.00 MEMB H

ATOM 3407 C27 POPC 26 16.264 -9.909 9.225 1.00 0.00 MEMB C

ATOM 3408 H7R POPC 26 16.344 -8.921 8.740 1.00 0.00 MEMB H

ATOM 3409 H7S POPC 26 15.214 -10.044 9.347 1.00 0.00 MEMB H

ATOM 3410 C28 POPC 26 16.804 -11.072 8.341 1.00 0.00 MEMB C

ATOM 3411 H8R POPC 26 16.447 -12.107 8.476 1.00 0.00 MEMB H

ATOM 3412 H8S POPC 26 17.858 -11.256 8.569 1.00 0.00 MEMB H

ATOM 3413 C29 POPC 26 16.463 -10.731 6.917 1.00 0.00 MEMB C

ATOM 3414 H91 POPC 26 15.628 -10.048 6.717 1.00 0.00 MEMB H

ATOM 3415 C210 POPC 26 17.065 -11.250 5.880 1.00 0.00 MEMB C

ATOM 3416 H101 POPC 26 16.785 -10.876 4.883 1.00 0.00 MEMB H

ATOM 3417 C211 POPC 26 18.168 -12.245 6.043 1.00 0.00 MEMB C

ATOM 3418 H11R POPC 26 17.970 -13.043 6.782 1.00 0.00 MEMB H

ATOM 3419 H11S POPC 26 19.138 -11.733 6.263 1.00 0.00 MEMB H

ATOM 3420 C212 POPC 26 18.322 -12.960 4.767 1.00 0.00 MEMB C

ATOM 3421 H12R POPC 26 17.476 -13.548 4.349 1.00 0.00 MEMB H

ATOM 3422 H12S POPC 26 19.115 -13.715 4.910 1.00 0.00 MEMB H

ATOM 3423 C213 POPC 26 18.659 -11.927 3.777 1.00 0.00 MEMB C

ATOM 3424 H13R POPC 26 19.431 -11.164 3.977 1.00 0.00 MEMB H

ATOM 3425 H13S POPC 26 17.813 -11.348 3.379 1.00 0.00 MEMB H

ATOM 3426 C214 POPC 26 19.084 -12.763 2.750 1.00 0.00 MEMB C

ATOM 3427 H14R POPC 26 19.131 -11.970 2.012 1.00 0.00 MEMB H

ATOM 3428 H14S POPC 26 18.488 -13.660 2.388 1.00 0.00 MEMB H

ATOM 3429 C215 POPC 26 20.458 -13.007 2.854 1.00 0.00 MEMB C

ATOM 3430 H15R POPC 26 20.433 -13.790 2.066 1.00 0.00 MEMB H

ATOM 3431 H15S POPC 26 20.624 -13.988 3.347 1.00 0.00 MEMB H

ATOM 3432 C216 POPC 26 21.517 -11.653 2.701 1.00 0.00 MEMB C

ATOM 3433 H16R POPC 26 22.479 -11.729 3.234 1.00 0.00 MEMB H

ATOM 3434 H16S POPC 26 21.253 -11.138 3.629 1.00 0.00 MEMB H

ATOM 3435 C217 POPC 26 22.145 -10.819 1.298 1.00 0.00 MEMB C

ATOM 3436 H17R POPC 26 22.935 -10.093 1.556 1.00 0.00 MEMB H

ATOM 3437 H17S POPC 26 21.228 -10.314 1.001 1.00 0.00 MEMB H

ATOM 3438 C218 POPC 26 22.688 -11.230 -0.218 1.00 0.00 MEMB C

ATOM 3439 H18R POPC 26 23.344 -12.034 -0.606 1.00 0.00 MEMB H

ATOM 3440 H18S POPC 26 22.748 -10.627 -1.127 1.00 0.00 MEMB H

ATOM 3441 H18T POPC 26 21.935 -11.871 -0.406 1.00 0.00 MEMB H

ATOM 3442 C33 POPC 26 22.526 -10.828 10.866 1.00 0.00 MEMB C

ATOM 3443 H3X POPC 26 23.581 -11.099 11.082 1.00 0.00 MEMB H

ATOM 3444 H3Y POPC 26 22.482 -9.828 10.383 1.00 0.00 MEMB H

ATOM 3445 C34 POPC 26 21.840 -11.868 9.961 1.00 0.00 MEMB C

ATOM 3446 H4X POPC 26 21.808 -12.868 10.425 1.00 0.00 MEMB H

ATOM 3447 H4Y POPC 26 22.481 -12.210 9.155 1.00 0.00 MEMB H

ATOM 3448 C35 POPC 26 20.527 -11.409 9.289 1.00 0.00 MEMB C

ATOM 3449 H5X POPC 26 19.805 -11.604 10.103 1.00 0.00 MEMB H

ATOM 3450 H5Y POPC 26 20.292 -12.091 8.441 1.00 0.00 MEMB H

ATOM 3451 C36 POPC 26 20.305 -9.937 8.801 1.00 0.00 MEMB C

ATOM 3452 H6X POPC 26 20.542 -9.203 9.597 1.00 0.00 MEMB H

ATOM 3453 H6Y POPC 26 19.220 -9.832 8.588 1.00 0.00 MEMB H

ATOM 3454 C37 POPC 26 20.963 -9.531 7.478 1.00 0.00 MEMB C

ATOM 3455 H7X POPC 26 20.840 -10.354 6.738 1.00 0.00 MEMB H

ATOM 3456 H7Y POPC 26 22.027 -9.347 7.711 1.00 0.00 MEMB H

ATOM 3457 C38 POPC 26 20.605 -8.185 6.855 1.00 0.00 MEMB C

ATOM 3458 H8X POPC 26 21.425 -7.953 6.127 1.00 0.00 MEMB H

ATOM 3459 H8Y POPC 26 20.642 -7.422 7.659 1.00 0.00 MEMB H

ATOM 3460 C39 POPC 26 19.272 -8.167 6.115 1.00 0.00 MEMB C

ATOM 3461 H9X POPC 26 18.678 -7.281 6.429 1.00 0.00 MEMB H

ATOM 3462 H9Y POPC 26 18.685 -9.080 6.347 1.00 0.00 MEMB H

ATOM 3463 C310 POPC 26 19.461 -8.096 4.606 1.00 0.00 MEMB C

ATOM 3464 H10X POPC 26 20.109 -8.954 4.306 1.00 0.00 MEMB H

ATOM 3465 H10Y POPC 26 20.003 -7.154 4.367 1.00 0.00 MEMB H

ATOM 3466 C311 POPC 26 18.115 -8.149 3.863 1.00 0.00 MEMB C

ATOM 3467 H11X POPC 26 17.380 -7.475 4.339 1.00 0.00 MEMB H

ATOM 3468 H11Y POPC 26 17.744 -9.196 3.882 1.00 0.00 MEMB H

ATOM 3469 C312 POPC 26 18.188 -7.764 2.411 1.00 0.00 MEMB C

ATOM 3470 H12X POPC 26 18.592 -6.722 2.305 1.00 0.00 MEMB H

ATOM 3471 H12Y POPC 26 17.229 -7.812 1.856 1.00 0.00 MEMB H

ATOM 3472 C313 POPC 26 19.065 -8.758 1.724 1.00 0.00 MEMB C

ATOM 3473 H13X POPC 26 19.054 -9.838 1.907 1.00 0.00 MEMB H

ATOM 3474 H13Y POPC 26 20.003 -8.562 2.234 1.00 0.00 MEMB H

ATOM 3475 C314 POPC 26 19.040 -8.366 0.283 1.00 0.00 MEMB C

ATOM 3476 H14X POPC 26 19.999 -8.809 -0.061 1.00 0.00 MEMB H

ATOM 3477 H14Y POPC 26 18.974 -7.267 0.184 1.00 0.00 MEMB H

ATOM 3478 C315 POPC 26 18.125 -8.937 -0.800 1.00 0.00 MEMB C

ATOM 3479 H15X POPC 26 17.309 -9.459 -0.302 1.00 0.00 MEMB H

ATOM 3480 H15Y POPC 26 18.905 -9.538 -1.286 1.00 0.00 MEMB H

ATOM 3481 C316 POPC 26 17.848 -8.489 -2.238 1.00 0.00 MEMB C

ATOM 3482 H16X POPC 26 17.294 -9.312 -2.739 1.00 0.00 MEMB H

ATOM 3483 H16Y POPC 26 18.762 -8.404 -2.877 1.00 0.00 MEMB H

ATOM 3484 H16Z POPC 26 17.341 -7.530 -2.206 1.00 0.00 MEMB H

ATOM 3485 N POPC 27 -17.216 17.856 20.244 1.00 0.00 MEMB N

ATOM 3486 C12 POPC 27 -17.580 16.390 20.453 1.00 0.00 MEMB C

ATOM 3487 H12A POPC 27 -18.270 16.300 21.279 1.00 0.00 MEMB H

ATOM 3488 H12B POPC 27 -16.655 15.899 20.713 1.00 0.00 MEMB H

ATOM 3489 C13 POPC 27 -18.317 18.711 19.726 1.00 0.00 MEMB C

ATOM 3490 H13A POPC 27 -18.917 18.091 19.063 1.00 0.00 MEMB H

ATOM 3491 H13B POPC 27 -18.929 19.085 20.532 1.00 0.00 MEMB H

ATOM 3492 H13C POPC 27 -17.923 19.545 19.156 1.00 0.00 MEMB H

ATOM 3493 C14 POPC 27 -16.648 18.552 21.444 1.00 0.00 MEMB C

ATOM 3494 H14A POPC 27 -15.999 17.915 22.032 1.00 0.00 MEMB H

ATOM 3495 H14B POPC 27 -16.087 19.434 21.154 1.00 0.00 MEMB H

ATOM 3496 H14C POPC 27 -17.463 18.859 22.096 1.00 0.00 MEMB H

ATOM 3497 C15 POPC 27 -16.255 17.774 19.159 1.00 0.00 MEMB C

ATOM 3498 H15A POPC 27 -15.263 17.462 19.512 1.00 0.00 MEMB H

ATOM 3499 H15B POPC 27 -16.015 18.739 18.723 1.00 0.00 MEMB H

ATOM 3500 H15C POPC 27 -16.534 17.087 18.373 1.00 0.00 MEMB H

ATOM 3501 C11 POPC 27 -18.139 15.586 19.229 1.00 0.00 MEMB C

ATOM 3502 H11A POPC 27 -18.267 14.524 19.534 1.00 0.00 MEMB H

ATOM 3503 H11B POPC 27 -17.432 15.560 18.363 1.00 0.00 MEMB H

ATOM 3504 P POPC 27 -20.270 15.455 17.694 1.00 0.00 MEMB P

ATOM 3505 O13 POPC 27 -21.152 14.424 18.289 1.00 0.00 MEMB O

ATOM 3506 O14 POPC 27 -19.410 15.082 16.557 1.00 0.00 MEMB O

ATOM 3507 O12 POPC 27 -19.387 16.129 18.835 1.00 0.00 MEMB O

ATOM 3508 O11 POPC 27 -21.159 16.682 17.241 1.00 0.00 MEMB O

ATOM 3509 C1 POPC 27 -20.849 18.031 17.604 1.00 0.00 MEMB C

ATOM 3510 HA POPC 27 -19.799 18.267 17.307 1.00 0.00 MEMB H

ATOM 3511 HB POPC 27 -20.945 18.153 18.704 1.00 0.00 MEMB H

ATOM 3512 C2 POPC 27 -21.803 19.048 16.899 1.00 0.00 MEMB C

ATOM 3513 HS POPC 27 -22.719 19.146 17.532 1.00 0.00 MEMB H

ATOM 3514 O21 POPC 27 -22.096 18.514 15.595 1.00 0.00 MEMB O

ATOM 3515 C21 POPC 27 -23.155 19.028 15.006 1.00 0.00 MEMB C

ATOM 3516 O22 POPC 27 -23.943 19.795 15.522 1.00 0.00 MEMB O

ATOM 3517 C22 POPC 27 -23.130 18.616 13.547 1.00 0.00 MEMB C

ATOM 3518 H2R POPC 27 -23.471 17.568 13.461 1.00 0.00 MEMB H

ATOM 3519 H2S POPC 27 -22.071 18.682 13.218 1.00 0.00 MEMB H

ATOM 3520 C3 POPC 27 -21.186 20.467 16.745 1.00 0.00 MEMB C

ATOM 3521 HX POPC 27 -21.081 20.932 17.745 1.00 0.00 MEMB H

ATOM 3522 HY POPC 27 -21.873 21.141 16.171 1.00 0.00 MEMB H

ATOM 3523 O31 POPC 27 -19.871 20.325 16.167 1.00 0.00 MEMB O

ATOM 3524 C31 POPC 27 -19.874 20.346 14.828 1.00 0.00 MEMB C

ATOM 3525 O32 POPC 27 -20.791 20.816 14.179 1.00 0.00 MEMB O

ATOM 3526 C32 POPC 27 -18.434 19.864 14.394 1.00 0.00 MEMB C

ATOM 3527 H2X POPC 27 -18.300 18.839 14.753 1.00 0.00 MEMB H

ATOM 3528 H2Y POPC 27 -17.752 20.503 15.004 1.00 0.00 MEMB H

ATOM 3529 C23 POPC 27 -24.010 19.507 12.654 1.00 0.00 MEMB C

ATOM 3530 H3R POPC 27 -23.578 20.537 12.641 1.00 0.00 MEMB H

ATOM 3531 H3S POPC 27 -25.038 19.579 13.073 1.00 0.00 MEMB H

ATOM 3532 C24 POPC 27 -24.072 18.948 11.232 1.00 0.00 MEMB C

ATOM 3533 H4R POPC 27 -24.894 19.462 10.672 1.00 0.00 MEMB H

ATOM 3534 H4S POPC 27 -24.223 17.854 11.182 1.00 0.00 MEMB H

ATOM 3535 C25 POPC 27 -22.761 19.177 10.522 1.00 0.00 MEMB C

ATOM 3536 H5R POPC 27 -21.861 19.156 11.166 1.00 0.00 MEMB H

ATOM 3537 H5S POPC 27 -22.904 20.212 10.268 1.00 0.00 MEMB H

ATOM 3538 C26 POPC 27 -22.566 18.431 9.205 1.00 0.00 MEMB C

ATOM 3539 H6R POPC 27 -22.426 17.356 9.299 1.00 0.00 MEMB H

ATOM 3540 H6S POPC 27 -21.577 18.826 8.835 1.00 0.00 MEMB H

ATOM 3541 C27 POPC 27 -23.655 18.765 8.166 1.00 0.00 MEMB C

ATOM 3542 H7R POPC 27 -23.089 18.812 7.208 1.00 0.00 MEMB H

ATOM 3543 H7S POPC 27 -24.161 19.729 8.423 1.00 0.00 MEMB H

ATOM 3544 C28 POPC 27 -24.797 17.829 7.890 1.00 0.00 MEMB C

ATOM 3545 H8R POPC 27 -25.464 17.858 8.777 1.00 0.00 MEMB H

ATOM 3546 H8S POPC 27 -24.313 16.840 7.791 1.00 0.00 MEMB H

ATOM 3547 C29 POPC 27 -25.553 18.258 6.648 1.00 0.00 MEMB C

ATOM 3548 H91 POPC 27 -26.327 19.025 6.815 1.00 0.00 MEMB H

ATOM 3549 C210 POPC 27 -25.317 17.866 5.384 1.00 0.00 MEMB C

ATOM 3550 H101 POPC 27 -25.992 18.240 4.594 1.00 0.00 MEMB H

ATOM 3551 C211 POPC 27 -24.241 16.909 4.911 1.00 0.00 MEMB C

ATOM 3552 H11R POPC 27 -24.683 16.138 4.236 1.00 0.00 MEMB H

ATOM 3553 H11S POPC 27 -23.677 16.407 5.721 1.00 0.00 MEMB H

ATOM 3554 C212 POPC 27 -23.153 17.613 4.132 1.00 0.00 MEMB C

ATOM 3555 H12R POPC 27 -22.196 17.065 4.068 1.00 0.00 MEMB H

ATOM 3556 H12S POPC 27 -22.934 18.631 4.559 1.00 0.00 MEMB H

ATOM 3557 C213 POPC 27 -23.631 17.786 2.729 1.00 0.00 MEMB C

ATOM 3558 H13R POPC 27 -22.806 18.380 2.351 1.00 0.00 MEMB H

ATOM 3559 H13S POPC 27 -24.524 18.429 2.842 1.00 0.00 MEMB H

ATOM 3560 C214 POPC 27 -23.811 16.656 1.684 1.00 0.00 MEMB C

ATOM 3561 H14R POPC 27 -24.022 17.161 0.723 1.00 0.00 MEMB H

ATOM 3562 H14S POPC 27 -24.757 16.122 1.812 1.00 0.00 MEMB H

ATOM 3563 C215 POPC 27 -22.679 15.619 1.481 1.00 0.00 MEMB C

ATOM 3564 H15R POPC 27 -22.641 14.943 2.325 1.00 0.00 MEMB H

ATOM 3565 H15S POPC 27 -21.612 15.821 1.522 1.00 0.00 MEMB H

ATOM 3566 C216 POPC 27 -22.612 15.025 0.095 1.00 0.00 MEMB C

ATOM 3567 H16R POPC 27 -23.646 14.675 -0.177 1.00 0.00 MEMB H

ATOM 3568 H16S POPC 27 -21.851 14.229 0.133 1.00 0.00 MEMB H

ATOM 3569 C217 POPC 27 -22.121 15.865 -1.105 1.00 0.00 MEMB C

ATOM 3570 H17R POPC 27 -21.444 16.618 -0.916 1.00 0.00 MEMB H

ATOM 3571 H17S POPC 27 -23.137 16.061 -1.432 1.00 0.00 MEMB H

ATOM 3572 C218 POPC 27 -21.096 15.621 -2.211 1.00 0.00 MEMB C

ATOM 3573 H18R POPC 27 -20.509 14.737 -2.012 1.00 0.00 MEMB H

ATOM 3574 H18S POPC 27 -20.911 15.862 -3.246 1.00 0.00 MEMB H

ATOM 3575 H18T POPC 27 -22.015 15.595 -2.827 1.00 0.00 MEMB H

ATOM 3576 C33 POPC 27 -17.919 19.936 12.917 1.00 0.00 MEMB C

ATOM 3577 H3X POPC 27 -16.828 20.031 12.755 1.00 0.00 MEMB H

ATOM 3578 H3Y POPC 27 -18.048 20.885 12.505 1.00 0.00 MEMB H

ATOM 3579 C34 POPC 27 -18.675 19.180 11.857 1.00 0.00 MEMB C

ATOM 3580 H4X POPC 27 -18.743 19.776 10.910 1.00 0.00 MEMB H

ATOM 3581 H4Y POPC 27 -19.727 18.972 12.159 1.00 0.00 MEMB H

ATOM 3582 C35 POPC 27 -17.817 18.022 11.590 1.00 0.00 MEMB C

ATOM 3583 H5X POPC 27 -17.592 17.584 12.590 1.00 0.00 MEMB H

ATOM 3584 H5Y POPC 27 -16.876 18.404 11.121 1.00 0.00 MEMB H

ATOM 3585 C36 POPC 27 -18.578 17.061 10.738 1.00 0.00 MEMB C

ATOM 3586 H6X POPC 27 -19.643 17.008 11.066 1.00 0.00 MEMB H

ATOM 3587 H6Y POPC 27 -18.103 16.076 10.925 1.00 0.00 MEMB H

ATOM 3588 C37 POPC 27 -18.502 17.426 9.260 1.00 0.00 MEMB C

ATOM 3589 H7X POPC 27 -17.571 18.008 9.063 1.00 0.00 MEMB H

ATOM 3590 H7Y POPC 27 -19.361 18.084 9.007 1.00 0.00 MEMB H

ATOM 3591 C38 POPC 27 -18.506 16.120 8.468 1.00 0.00 MEMB C

ATOM 3592 H8X POPC 27 -19.422 15.554 8.742 1.00 0.00 MEMB H

ATOM 3593 H8Y POPC 27 -17.639 15.516 8.818 1.00 0.00 MEMB H

ATOM 3594 C39 POPC 27 -18.377 16.255 6.947 1.00 0.00 MEMB C

ATOM 3595 H9X POPC 27 -18.294 15.221 6.538 1.00 0.00 MEMB H

ATOM 3596 H9Y POPC 27 -17.437 16.801 6.726 1.00 0.00 MEMB H

ATOM 3597 C310 POPC 27 -19.552 16.971 6.270 1.00 0.00 MEMB C

ATOM 3598 H10X POPC 27 -19.788 17.893 6.842 1.00 0.00 MEMB H

ATOM 3599 H10Y POPC 27 -20.439 16.299 6.351 1.00 0.00 MEMB H

ATOM 3600 C311 POPC 27 -19.339 17.319 4.777 1.00 0.00 MEMB C

ATOM 3601 H11X POPC 27 -20.278 17.796 4.416 1.00 0.00 MEMB H

ATOM 3602 H11Y POPC 27 -19.215 16.378 4.197 1.00 0.00 MEMB H

ATOM 3603 C312 POPC 27 -18.133 18.230 4.422 1.00 0.00 MEMB C

ATOM 3604 H12X POPC 27 -17.220 17.872 4.931 1.00 0.00 MEMB H

ATOM 3605 H12Y POPC 27 -18.349 19.277 4.729 1.00 0.00 MEMB H

ATOM 3606 C313 POPC 27 -17.720 18.198 2.942 1.00 0.00 MEMB C

ATOM 3607 H13X POPC 27 -17.742 17.127 2.592 1.00 0.00 MEMB H

ATOM 3608 H13Y POPC 27 -16.724 18.623 2.740 1.00 0.00 MEMB H

ATOM 3609 C314 POPC 27 -18.698 18.970 2.097 1.00 0.00 MEMB C

ATOM 3610 H14X POPC 27 -19.505 18.339 2.502 1.00 0.00 MEMB H

ATOM 3611 H14Y POPC 27 -18.530 18.746 1.022 1.00 0.00 MEMB H

ATOM 3612 C315 POPC 27 -19.131 20.444 2.434 1.00 0.00 MEMB C

ATOM 3613 H15X POPC 27 -18.149 20.913 2.579 1.00 0.00 MEMB H

ATOM 3614 H15Y POPC 27 -19.760 20.375 3.340 1.00 0.00 MEMB H

ATOM 3615 C316 POPC 27 -19.890 21.374 1.438 1.00 0.00 MEMB C

ATOM 3616 H16X POPC 27 -19.460 21.247 0.430 1.00 0.00 MEMB H

ATOM 3617 H16Y POPC 27 -20.605 22.144 1.637 1.00 0.00 MEMB H

ATOM 3618 H16Z POPC 27 -20.906 21.115 1.281 1.00 0.00 MEMB H

ATOM 3619 N POPC 28 9.807 -23.898 19.759 1.00 0.00 MEMB N

ATOM 3620 C12 POPC 28 10.248 -23.198 18.456 1.00 0.00 MEMB C

ATOM 3621 H12A POPC 28 9.474 -23.395 17.724 1.00 0.00 MEMB H

ATOM 3622 H12B POPC 28 11.171 -23.620 18.096 1.00 0.00 MEMB H

ATOM 3623 C13 POPC 28 9.517 -25.351 19.580 1.00 0.00 MEMB C

ATOM 3624 H13A POPC 28 9.374 -25.813 20.557 1.00 0.00 MEMB H

ATOM 3625 H13B POPC 28 8.582 -25.487 19.050 1.00 0.00 MEMB H

ATOM 3626 H13C POPC 28 10.323 -25.884 19.101 1.00 0.00 MEMB H

ATOM 3627 C14 POPC 28 10.807 -23.787 20.883 1.00 0.00 MEMB C

ATOM 3628 H14A POPC 28 10.391 -24.226 21.785 1.00 0.00 MEMB H

ATOM 3629 H14B POPC 28 11.747 -24.297 20.677 1.00 0.00 MEMB H

ATOM 3630 H14C POPC 28 11.011 -22.748 21.126 1.00 0.00 MEMB H

ATOM 3631 C15 POPC 28 8.528 -23.248 20.198 1.00 0.00 MEMB C

ATOM 3632 H15A POPC 28 8.147 -23.663 21.113 1.00 0.00 MEMB H

ATOM 3633 H15B POPC 28 7.781 -23.258 19.416 1.00 0.00 MEMB H

ATOM 3634 H15C POPC 28 8.697 -22.177 20.367 1.00 0.00 MEMB H

ATOM 3635 C11 POPC 28 10.462 -21.645 18.335 1.00 0.00 MEMB C

ATOM 3636 H11A POPC 28 10.498 -21.414 17.236 1.00 0.00 MEMB H

ATOM 3637 H11B POPC 28 11.465 -21.368 18.722 1.00 0.00 MEMB H

ATOM 3638 P POPC 28 8.390 -20.011 18.256 1.00 0.00 MEMB P

ATOM 3639 O13 POPC 28 8.818 -18.613 18.101 1.00 0.00 MEMB O

ATOM 3640 O14 POPC 28 7.135 -20.248 19.008 1.00 0.00 MEMB O

ATOM 3641 O12 POPC 28 9.480 -20.868 19.015 1.00 0.00 MEMB O

ATOM 3642 O11 POPC 28 8.281 -20.686 16.839 1.00 0.00 MEMB O

ATOM 3643 C1 POPC 28 7.082 -21.292 16.367 1.00 0.00 MEMB C

ATOM 3644 HA POPC 28 6.868 -20.900 15.350 1.00 0.00 MEMB H

ATOM 3645 HB POPC 28 6.185 -21.058 16.994 1.00 0.00 MEMB H

ATOM 3646 C2 POPC 28 7.199 -22.842 16.305 1.00 0.00 MEMB C

ATOM 3647 HS POPC 28 7.272 -23.184 17.361 1.00 0.00 MEMB H

ATOM 3648 O21 POPC 28 8.382 -23.243 15.579 1.00 0.00 MEMB O

ATOM 3649 C21 POPC 28 8.649 -24.541 15.567 1.00 0.00 MEMB C

ATOM 3650 O22 POPC 28 8.027 -25.406 16.159 1.00 0.00 MEMB O

ATOM 3651 C22 POPC 28 9.999 -24.744 14.885 1.00 0.00 MEMB C

ATOM 3652 H2R POPC 28 10.403 -25.732 15.176 1.00 0.00 MEMB H

ATOM 3653 H2S POPC 28 10.694 -23.953 15.247 1.00 0.00 MEMB H

ATOM 3654 C3 POPC 28 5.898 -23.401 15.651 1.00 0.00 MEMB C

ATOM 3655 HX POPC 28 5.084 -23.135 16.362 1.00 0.00 MEMB H

ATOM 3656 HY POPC 28 5.888 -24.509 15.592 1.00 0.00 MEMB H

ATOM 3657 O31 POPC 28 5.615 -22.771 14.380 1.00 0.00 MEMB O

ATOM 3658 C31 POPC 28 6.081 -23.398 13.298 1.00 0.00 MEMB C

ATOM 3659 O32 POPC 28 6.735 -24.423 13.305 1.00 0.00 MEMB O

ATOM 3660 C32 POPC 28 5.668 -22.618 12.036 1.00 0.00 MEMB C

ATOM 3661 H2X POPC 28 5.977 -21.563 12.203 1.00 0.00 MEMB H

ATOM 3662 H2Y POPC 28 4.565 -22.637 11.953 1.00 0.00 MEMB H

ATOM 3663 C23 POPC 28 9.883 -24.655 13.354 1.00 0.00 MEMB C

ATOM 3664 H3R POPC 28 9.319 -23.730 13.081 1.00 0.00 MEMB H

ATOM 3665 H3S POPC 28 9.305 -25.520 12.962 1.00 0.00 MEMB H

ATOM 3666 C24 POPC 28 11.281 -24.569 12.732 1.00 0.00 MEMB C

ATOM 3667 H4R POPC 28 11.894 -25.426 13.087 1.00 0.00 MEMB H

ATOM 3668 H4S POPC 28 11.741 -23.638 13.138 1.00 0.00 MEMB H

ATOM 3669 C25 POPC 28 11.316 -24.488 11.193 1.00 0.00 MEMB C

ATOM 3670 H5R POPC 28 10.931 -25.426 10.737 1.00 0.00 MEMB H

ATOM 3671 H5S POPC 28 12.378 -24.382 10.873 1.00 0.00 MEMB H

ATOM 3672 C26 POPC 28 10.517 -23.293 10.655 1.00 0.00 MEMB C

ATOM 3673 H6R POPC 28 10.597 -22.466 11.398 1.00 0.00 MEMB H

ATOM 3674 H6S POPC 28 9.447 -23.580 10.594 1.00 0.00 MEMB H

ATOM 3675 C27 POPC 28 11.007 -22.716 9.314 1.00 0.00 MEMB C

ATOM 3676 H7R POPC 28 10.588 -23.283 8.452 1.00 0.00 MEMB H

ATOM 3677 H7S POPC 28 12.116 -22.783 9.253 1.00 0.00 MEMB H

ATOM 3678 C28 POPC 28 10.675 -21.221 9.198 1.00 0.00 MEMB C

ATOM 3679 H8R POPC 28 11.240 -20.813 8.332 1.00 0.00 MEMB H

ATOM 3680 H8S POPC 28 11.062 -20.688 10.095 1.00 0.00 MEMB H

ATOM 3681 C29 POPC 28 9.186 -21.043 9.059 1.00 0.00 MEMB C

ATOM 3682 H91 POPC 28 8.583 -21.404 9.908 1.00 0.00 MEMB H

ATOM 3683 C210 POPC 28 8.565 -20.600 7.954 1.00 0.00 MEMB C

ATOM 3684 H101 POPC 28 7.467 -20.575 7.932 1.00 0.00 MEMB H

ATOM 3685 C211 POPC 28 9.256 -20.208 6.674 1.00 0.00 MEMB C

ATOM 3686 H11R POPC 28 10.237 -19.733 6.886 1.00 0.00 MEMB H

ATOM 3687 H11S POPC 28 8.644 -19.460 6.128 1.00 0.00 MEMB H

ATOM 3688 C212 POPC 28 9.510 -21.444 5.795 1.00 0.00 MEMB C

ATOM 3689 H12R POPC 28 8.534 -21.866 5.466 1.00 0.00 MEMB H

ATOM 3690 H12S POPC 28 10.017 -22.224 6.409 1.00 0.00 MEMB H

ATOM 3691 C213 POPC 28 10.401 -21.175 4.572 1.00 0.00 MEMB C

ATOM 3692 H13R POPC 28 11.454 -21.383 4.862 1.00 0.00 MEMB H

ATOM 3693 H13S POPC 28 10.338 -20.114 4.251 1.00 0.00 MEMB H

ATOM 3694 C214 POPC 28 10.014 -22.035 3.373 1.00 0.00 MEMB C

ATOM 3695 H14R POPC 28 8.929 -21.874 3.177 1.00 0.00 MEMB H

ATOM 3696 H14S POPC 28 10.181 -23.108 3.612 1.00 0.00 MEMB H

ATOM 3697 C215 POPC 28 10.778 -21.740 2.081 1.00 0.00 MEMB C

ATOM 3698 H15R POPC 28 10.566 -20.705 1.732 1.00 0.00 MEMB H

ATOM 3699 H15S POPC 28 10.525 -22.435 1.251 1.00 0.00 MEMB H

ATOM 3700 C216 POPC 28 12.246 -21.961 2.262 1.00 0.00 MEMB C

ATOM 3701 H16R POPC 28 12.476 -22.990 2.612 1.00 0.00 MEMB H

ATOM 3702 H16S POPC 28 12.549 -21.259 3.072 1.00 0.00 MEMB H

ATOM 3703 C217 POPC 28 12.955 -21.702 0.940 1.00 0.00 MEMB C

ATOM 3704 H17R POPC 28 12.516 -20.793 0.447 1.00 0.00 MEMB H

ATOM 3705 H17S POPC 28 12.927 -22.584 0.289 1.00 0.00 MEMB H

ATOM 3706 C218 POPC 28 14.348 -21.334 1.234 1.00 0.00 MEMB C

ATOM 3707 H18R POPC 28 15.053 -22.081 1.652 1.00 0.00 MEMB H

ATOM 3708 H18S POPC 28 14.035 -20.716 2.061 1.00 0.00 MEMB H

ATOM 3709 H18T POPC 28 14.776 -20.662 0.482 1.00 0.00 MEMB H

ATOM 3710 C33 POPC 28 6.313 -23.148 10.718 1.00 0.00 MEMB C

ATOM 3711 H3X POPC 28 7.406 -23.266 10.851 1.00 0.00 MEMB H

ATOM 3712 H3Y POPC 28 6.152 -22.366 9.939 1.00 0.00 MEMB H

ATOM 3713 C34 POPC 28 5.710 -24.462 10.176 1.00 0.00 MEMB C

ATOM 3714 H4X POPC 28 4.756 -24.221 9.662 1.00 0.00 MEMB H

ATOM 3715 H4Y POPC 28 5.485 -25.148 11.024 1.00 0.00 MEMB H

ATOM 3716 C35 POPC 28 6.551 -25.268 9.165 1.00 0.00 MEMB C

ATOM 3717 H5X POPC 28 5.867 -26.041 8.734 1.00 0.00 MEMB H

ATOM 3718 H5Y POPC 28 7.385 -25.812 9.662 1.00 0.00 MEMB H

ATOM 3719 C36 POPC 28 7.123 -24.486 7.985 1.00 0.00 MEMB C

ATOM 3720 H6X POPC 28 7.951 -23.816 8.309 1.00 0.00 MEMB H

ATOM 3721 H6Y POPC 28 6.323 -23.851 7.542 1.00 0.00 MEMB H

ATOM 3722 C37 POPC 28 7.599 -25.485 6.925 1.00 0.00 MEMB C

ATOM 3723 H7X POPC 28 6.893 -26.345 6.887 1.00 0.00 MEMB H

ATOM 3724 H7Y POPC 28 8.602 -25.887 7.191 1.00 0.00 MEMB H

ATOM 3725 C38 POPC 28 7.619 -24.898 5.530 1.00 0.00 MEMB C

ATOM 3726 H8X POPC 28 8.519 -24.246 5.444 1.00 0.00 MEMB H

ATOM 3727 H8Y POPC 28 6.727 -24.264 5.331 1.00 0.00 MEMB H

ATOM 3728 C39 POPC 28 7.713 -25.977 4.466 1.00 0.00 MEMB C

ATOM 3729 H9X POPC 28 6.748 -26.502 4.264 1.00 0.00 MEMB H

ATOM 3730 H9Y POPC 28 8.454 -26.752 4.755 1.00 0.00 MEMB H

ATOM 3731 C310 POPC 28 8.249 -25.294 3.241 1.00 0.00 MEMB C

ATOM 3732 H10X POPC 28 8.418 -26.104 2.491 1.00 0.00 MEMB H

ATOM 3733 H10Y POPC 28 9.238 -24.848 3.379 1.00 0.00 MEMB H

ATOM 3734 C311 POPC 28 7.395 -24.118 2.773 1.00 0.00 MEMB C

ATOM 3735 H11X POPC 28 7.124 -23.247 3.369 1.00 0.00 MEMB H

ATOM 3736 H11Y POPC 28 6.356 -24.516 2.847 1.00 0.00 MEMB H

ATOM 3737 C312 POPC 28 8.059 -23.746 1.423 1.00 0.00 MEMB C

ATOM 3738 H12X POPC 28 8.569 -24.468 0.738 1.00 0.00 MEMB H

ATOM 3739 H12Y POPC 28 8.846 -23.102 1.679 1.00 0.00 MEMB H

ATOM 3740 C313 POPC 28 7.240 -23.181 0.360 1.00 0.00 MEMB C

ATOM 3741 H13X POPC 28 6.447 -22.736 0.968 1.00 0.00 MEMB H

ATOM 3742 H13Y POPC 28 6.632 -23.797 -0.312 1.00 0.00 MEMB H

ATOM 3743 C314 POPC 28 8.350 -22.597 -0.370 1.00 0.00 MEMB C

ATOM 3744 H14X POPC 28 8.981 -23.043 -1.166 1.00 0.00 MEMB H

ATOM 3745 H14Y POPC 28 8.902 -21.839 0.283 1.00 0.00 MEMB H

ATOM 3746 C315 POPC 28 7.618 -21.989 -1.316 1.00 0.00 MEMB C

ATOM 3747 H15X POPC 28 8.200 -21.077 -1.387 1.00 0.00 MEMB H

ATOM 3748 H15Y POPC 28 7.773 -21.004 -0.895 1.00 0.00 MEMB H

ATOM 3749 C316 POPC 28 6.379 -22.620 -2.196 1.00 0.00 MEMB C

ATOM 3750 H16X POPC 28 6.069 -22.215 -3.185 1.00 0.00 MEMB H

ATOM 3751 H16Y POPC 28 5.441 -22.099 -2.005 1.00 0.00 MEMB H

ATOM 3752 H16Z POPC 28 6.610 -23.243 -3.066 1.00 0.00 MEMB H

ATOM 3753 N POPC 29 23.084 10.287 19.751 1.00 0.00 MEMB N

ATOM 3754 C12 POPC 29 23.156 9.100 20.695 1.00 0.00 MEMB C

ATOM 3755 H12A POPC 29 22.204 8.975 21.192 1.00 0.00 MEMB H

ATOM 3756 H12B POPC 29 23.924 9.313 21.430 1.00 0.00 MEMB H

ATOM 3757 C13 POPC 29 22.040 10.048 18.703 1.00 0.00 MEMB C

ATOM 3758 H13A POPC 29 22.023 10.858 17.990 1.00 0.00 MEMB H

ATOM 3759 H13B POPC 29 22.263 9.112 18.171 1.00 0.00 MEMB H

ATOM 3760 H13C POPC 29 21.053 9.889 19.116 1.00 0.00 MEMB H

ATOM 3761 C14 POPC 29 22.766 11.559 20.460 1.00 0.00 MEMB C

ATOM 3762 H14A POPC 29 22.893 12.412 19.802 1.00 0.00 MEMB H

ATOM 3763 H14B POPC 29 21.741 11.553 20.813 1.00 0.00 MEMB H

ATOM 3764 H14C POPC 29 23.405 11.757 21.314 1.00 0.00 MEMB H

ATOM 3765 C15 POPC 29 24.383 10.404 19.008 1.00 0.00 MEMB C

ATOM 3766 H15A POPC 29 24.473 9.533 18.348 1.00 0.00 MEMB H

ATOM 3767 H15B POPC 29 25.236 10.364 19.671 1.00 0.00 MEMB H

ATOM 3768 H15C POPC 29 24.408 11.291 18.392 1.00 0.00 MEMB H

ATOM 3769 C11 POPC 29 23.534 7.725 20.091 1.00 0.00 MEMB C

ATOM 3770 H11A POPC 29 23.537 6.980 20.902 1.00 0.00 MEMB H

ATOM 3771 H11B POPC 29 24.565 7.810 19.685 1.00 0.00 MEMB H

ATOM 3772 P POPC 29 23.143 6.350 17.926 1.00 0.00 MEMB P

ATOM 3773 O13 POPC 29 22.176 6.477 16.824 1.00 0.00 MEMB O

ATOM 3774 O14 POPC 29 23.447 5.022 18.483 1.00 0.00 MEMB O

ATOM 3775 O12 POPC 29 22.615 7.276 19.118 1.00 0.00 MEMB O

ATOM 3776 O11 POPC 29 24.533 6.993 17.538 1.00 0.00 MEMB O

ATOM 3777 C1 POPC 29 25.198 6.735 16.299 1.00 0.00 MEMB C

ATOM 3778 HA POPC 29 24.710 5.942 15.678 1.00 0.00 MEMB H

ATOM 3779 HB POPC 29 26.247 6.426 16.496 1.00 0.00 MEMB H

ATOM 3780 C2 POPC 29 25.227 8.050 15.513 1.00 0.00 MEMB C

ATOM 3781 HS POPC 29 25.731 8.788 16.183 1.00 0.00 MEMB H

ATOM 3782 O21 POPC 29 23.862 8.382 15.304 1.00 0.00 MEMB O

ATOM 3783 C21 POPC 29 23.609 9.638 15.287 1.00 0.00 MEMB C

ATOM 3784 O22 POPC 29 24.421 10.551 15.260 1.00 0.00 MEMB O

ATOM 3785 C22 POPC 29 22.110 9.790 15.324 1.00 0.00 MEMB C

ATOM 3786 H2R POPC 29 21.887 10.828 15.553 1.00 0.00 MEMB H

ATOM 3787 H2S POPC 29 21.680 9.220 16.166 1.00 0.00 MEMB H

ATOM 3788 C3 POPC 29 25.983 7.987 14.161 1.00 0.00 MEMB C

ATOM 3789 HX POPC 29 26.960 7.500 14.356 1.00 0.00 MEMB H

ATOM 3790 HY POPC 29 26.196 9.023 13.797 1.00 0.00 MEMB H

ATOM 3791 O31 POPC 29 25.244 7.176 13.229 1.00 0.00 MEMB O

ATOM 3792 C31 POPC 29 24.668 7.790 12.217 1.00 0.00 MEMB C

ATOM 3793 O32 POPC 29 24.905 8.919 11.824 1.00 0.00 MEMB O

ATOM 3794 C32 POPC 29 23.674 6.833 11.573 1.00 0.00 MEMB C

ATOM 3795 H2X POPC 29 22.801 6.721 12.247 1.00 0.00 MEMB H

ATOM 3796 H2Y POPC 29 24.159 5.840 11.492 1.00 0.00 MEMB H

ATOM 3797 C23 POPC 29 21.575 9.313 13.968 1.00 0.00 MEMB C

ATOM 3798 H3R POPC 29 21.773 8.223 13.871 1.00 0.00 MEMB H

ATOM 3799 H3S POPC 29 22.161 9.763 13.161 1.00 0.00 MEMB H

ATOM 3800 C24 POPC 29 20.083 9.560 13.783 1.00 0.00 MEMB C

ATOM 3801 H4R POPC 29 19.836 10.640 13.714 1.00 0.00 MEMB H

ATOM 3802 H4S POPC 29 19.638 9.158 14.726 1.00 0.00 MEMB H

ATOM 3803 C25 POPC 29 19.479 8.734 12.634 1.00 0.00 MEMB C

ATOM 3804 H5R POPC 29 18.381 8.684 12.793 1.00 0.00 MEMB H

ATOM 3805 H5S POPC 29 19.901 7.708 12.733 1.00 0.00 MEMB H

ATOM 3806 C26 POPC 29 19.708 9.234 11.211 1.00 0.00 MEMB C

ATOM 3807 H6R POPC 29 20.789 9.251 10.948 1.00 0.00 MEMB H

ATOM 3808 H6S POPC 29 19.288 10.255 11.065 1.00 0.00 MEMB H

ATOM 3809 C27 POPC 29 19.085 8.367 10.124 1.00 0.00 MEMB C

ATOM 3810 H7R POPC 29 17.980 8.604 10.201 1.00 0.00 MEMB H

ATOM 3811 H7S POPC 29 19.133 7.285 10.415 1.00 0.00 MEMB H

ATOM 3812 C28 POPC 29 19.956 8.598 8.834 1.00 0.00 MEMB C

ATOM 3813 H8R POPC 29 20.882 8.227 8.892 1.00 0.00 MEMB H

ATOM 3814 H8S POPC 29 19.829 9.814 8.766 1.00 0.00 MEMB H

ATOM 3815 C29 POPC 29 19.316 8.566 7.507 1.00 0.00 MEMB C

ATOM 3816 H91 POPC 29 18.451 8.997 7.582 1.00 0.00 MEMB H

ATOM 3817 C210 POPC 29 19.552 7.557 6.363 1.00 0.00 MEMB C

ATOM 3818 H101 POPC 29 18.966 7.051 5.913 1.00 0.00 MEMB H

ATOM 3819 C211 POPC 29 21.021 7.251 5.998 1.00 0.00 MEMB C

ATOM 3820 H11R POPC 29 20.868 6.065 5.691 1.00 0.00 MEMB H

ATOM 3821 H11S POPC 29 21.643 7.404 6.743 1.00 0.00 MEMB H

ATOM 3822 C212 POPC 29 20.890 8.066 4.680 1.00 0.00 MEMB C

ATOM 3823 H12R POPC 29 21.946 8.255 4.448 1.00 0.00 MEMB H

ATOM 3824 H12S POPC 29 20.464 9.141 4.726 1.00 0.00 MEMB H

ATOM 3825 C213 POPC 29 20.209 7.560 3.365 1.00 0.00 MEMB C

ATOM 3826 H13R POPC 29 20.021 8.446 2.706 1.00 0.00 MEMB H

ATOM 3827 H13S POPC 29 19.228 7.122 3.560 1.00 0.00 MEMB H

ATOM 3828 C214 POPC 29 21.068 6.572 2.579 1.00 0.00 MEMB C

ATOM 3829 H14R POPC 29 21.103 5.583 3.084 1.00 0.00 MEMB H

ATOM 3830 H14S POPC 29 22.078 7.031 2.576 1.00 0.00 MEMB H

ATOM 3831 C215 POPC 29 20.639 6.379 1.122 1.00 0.00 MEMB C

ATOM 3832 H15R POPC 29 20.320 7.363 0.708 1.00 0.00 MEMB H

ATOM 3833 H15S POPC 29 19.786 5.682 1.088 1.00 0.00 MEMB H

ATOM 3834 C216 POPC 29 21.757 5.908 0.201 1.00 0.00 MEMB C

ATOM 3835 H16R POPC 29 22.687 6.464 0.453 1.00 0.00 MEMB H

ATOM 3836 H16S POPC 29 21.478 6.162 -0.851 1.00 0.00 MEMB H

ATOM 3837 C217 POPC 29 22.021 4.415 0.181 1.00 0.00 MEMB C

ATOM 3838 H17R POPC 29 21.130 3.879 -0.223 1.00 0.00 MEMB H

ATOM 3839 H17S POPC 29 22.228 3.998 1.195 1.00 0.00 MEMB H

ATOM 3840 C218 POPC 29 23.171 4.175 -0.779 1.00 0.00 MEMB C

ATOM 3841 H18R POPC 29 24.133 4.606 -0.401 1.00 0.00 MEMB H

ATOM 3842 H18S POPC 29 22.975 4.529 -1.801 1.00 0.00 MEMB H

ATOM 3843 H18T POPC 29 23.237 3.096 -0.961 1.00 0.00 MEMB H

ATOM 3844 C33 POPC 29 23.231 7.372 10.203 1.00 0.00 MEMB C

ATOM 3845 H3X POPC 29 24.112 7.674 9.589 1.00 0.00 MEMB H

ATOM 3846 H3Y POPC 29 22.624 8.295 10.332 1.00 0.00 MEMB H

ATOM 3847 C34 POPC 29 22.381 6.385 9.404 1.00 0.00 MEMB C

ATOM 3848 H4X POPC 29 22.253 6.857 8.413 1.00 0.00 MEMB H

ATOM 3849 H4Y POPC 29 21.388 6.239 9.874 1.00 0.00 MEMB H

ATOM 3850 C35 POPC 29 23.004 5.043 9.139 1.00 0.00 MEMB C

ATOM 3851 H5X POPC 29 22.309 4.406 8.538 1.00 0.00 MEMB H

ATOM 3852 H5Y POPC 29 23.207 4.499 10.081 1.00 0.00 MEMB H

ATOM 3853 C36 POPC 29 24.298 5.214 8.388 1.00 0.00 MEMB C

ATOM 3854 H6X POPC 29 24.874 4.366 8.776 1.00 0.00 MEMB H

ATOM 3855 H6Y POPC 29 25.010 5.998 8.681 1.00 0.00 MEMB H

ATOM 3856 C37 POPC 29 24.075 5.116 6.880 1.00 0.00 MEMB C

ATOM 3857 H7X POPC 29 23.208 4.495 6.559 1.00 0.00 MEMB H

ATOM 3858 H7Y POPC 29 24.998 4.598 6.650 1.00 0.00 MEMB H

ATOM 3859 C38 POPC 29 24.326 6.334 6.031 1.00 0.00 MEMB C

ATOM 3860 H8X POPC 29 23.719 7.153 6.456 1.00 0.00 MEMB H

ATOM 3861 H8Y POPC 29 23.994 6.139 4.983 1.00 0.00 MEMB H

ATOM 3862 C39 POPC 29 25.812 6.715 6.072 1.00 0.00 MEMB C

ATOM 3863 H9X POPC 29 26.467 6.008 5.510 1.00 0.00 MEMB H

ATOM 3864 H9Y POPC 29 26.176 6.744 7.120 1.00 0.00 MEMB H

ATOM 3865 C310 POPC 29 25.963 8.103 5.521 1.00 0.00 MEMB C

ATOM 3866 H10X POPC 29 26.970 8.485 5.784 1.00 0.00 MEMB H

ATOM 3867 H10Y POPC 29 25.228 8.762 6.044 1.00 0.00 MEMB H

ATOM 3868 C311 POPC 29 25.704 8.064 4.017 1.00 0.00 MEMB C

ATOM 3869 H11X POPC 29 25.072 7.240 3.660 1.00 0.00 MEMB H

ATOM 3870 H11Y POPC 29 26.621 7.906 3.463 1.00 0.00 MEMB H

ATOM 3871 C312 POPC 29 25.195 9.379 3.550 1.00 0.00 MEMB C

ATOM 3872 H12X POPC 29 25.949 10.037 4.014 1.00 0.00 MEMB H

ATOM 3873 H12Y POPC 29 24.243 9.627 4.040 1.00 0.00 MEMB H

ATOM 3874 C313 POPC 29 25.122 9.490 2.029 1.00 0.00 MEMB C

ATOM 3875 H13X POPC 29 24.669 8.578 1.584 1.00 0.00 MEMB H

ATOM 3876 H13Y POPC 29 26.110 9.608 1.538 1.00 0.00 MEMB H

ATOM 3877 C314 POPC 29 24.239 10.620 1.561 1.00 0.00 MEMB C

ATOM 3878 H14X POPC 29 24.710 10.764 0.588 1.00 0.00 MEMB H

ATOM 3879 H14Y POPC 29 24.282 11.682 1.534 1.00 0.00 MEMB H

ATOM 3880 C315 POPC 29 22.776 10.569 2.033 1.00 0.00 MEMB C

ATOM 3881 H15X POPC 29 22.773 10.183 3.054 1.00 0.00 MEMB H

ATOM 3882 H15Y POPC 29 22.175 9.866 1.454 1.00 0.00 MEMB H

ATOM 3883 C316 POPC 29 22.034 11.876 2.177 1.00 0.00 MEMB C

ATOM 3884 H16X POPC 29 21.776 12.507 1.401 1.00 0.00 MEMB H

ATOM 3885 H16Y POPC 29 22.728 12.721 2.310 1.00 0.00 MEMB H

ATOM 3886 H16Z POPC 29 20.938 11.870 2.183 1.00 0.00 MEMB H

ATOM 3887 N POPC 30 -19.001 -15.183 21.039 1.00 0.00 MEMB N

ATOM 3888 C12 POPC 30 -19.366 -16.597 20.652 1.00 0.00 MEMB C

ATOM 3889 H12A POPC 30 -19.999 -17.031 21.419 1.00 0.00 MEMB H

ATOM 3890 H12B POPC 30 -18.472 -17.223 20.683 1.00 0.00 MEMB H

ATOM 3891 C13 POPC 30 -20.007 -14.178 20.607 1.00 0.00 MEMB C

ATOM 3892 H13A POPC 30 -19.940 -14.068 19.522 1.00 0.00 MEMB H

ATOM 3893 H13B POPC 30 -20.980 -14.546 20.845 1.00 0.00 MEMB H

ATOM 3894 H13C POPC 30 -19.796 -13.236 21.100 1.00 0.00 MEMB H

ATOM 3895 C14 POPC 30 -18.871 -15.161 22.495 1.00 0.00 MEMB C

ATOM 3896 H14A POPC 30 -19.784 -15.478 22.971 1.00 0.00 MEMB H

ATOM 3897 H14B POPC 30 -18.090 -15.844 22.801 1.00 0.00 MEMB H

ATOM 3898 H14C POPC 30 -18.588 -14.168 22.824 1.00 0.00 MEMB H

ATOM 3899 C15 POPC 30 -17.726 -14.790 20.422 1.00 0.00 MEMB C

ATOM 3900 H15A POPC 30 -17.852 -14.863 19.324 1.00 0.00 MEMB H

ATOM 3901 H15B POPC 30 -16.911 -15.494 20.569 1.00 0.00 MEMB H

ATOM 3902 H15C POPC 30 -17.403 -13.783 20.612 1.00 0.00 MEMB H

ATOM 3903 C11 POPC 30 -20.017 -16.818 19.289 1.00 0.00 MEMB C

ATOM 3904 H11A POPC 30 -21.012 -16.319 19.273 1.00 0.00 MEMB H

ATOM 3905 H11B POPC 30 -20.183 -17.912 19.143 1.00 0.00 MEMB H

ATOM 3906 P POPC 30 -19.671 -15.969 16.849 1.00 0.00 MEMB P

ATOM 3907 O13 POPC 30 -19.092 -14.676 16.449 1.00 0.00 MEMB O

ATOM 3908 O14 POPC 30 -21.105 -16.062 16.900 1.00 0.00 MEMB O

ATOM 3909 O12 POPC 30 -19.150 -16.312 18.292 1.00 0.00 MEMB O

ATOM 3910 O11 POPC 30 -19.049 -17.149 15.979 1.00 0.00 MEMB O

ATOM 3911 C1 POPC 30 -17.657 -16.863 15.817 1.00 0.00 MEMB C

ATOM 3912 HA POPC 30 -17.244 -17.744 16.128 1.00 0.00 MEMB H

ATOM 3913 HB POPC 30 -17.234 -16.326 16.646 1.00 0.00 MEMB H

ATOM 3914 C2 POPC 30 -17.185 -16.382 14.403 1.00 0.00 MEMB C

ATOM 3915 HS POPC 30 -17.399 -15.295 14.495 1.00 0.00 MEMB H

ATOM 3916 O21 POPC 30 -17.917 -17.116 13.494 1.00 0.00 MEMB O

ATOM 3917 C21 POPC 30 -18.909 -16.450 13.099 1.00 0.00 MEMB C

ATOM 3918 O22 POPC 30 -19.231 -15.301 13.331 1.00 0.00 MEMB O

ATOM 3919 C22 POPC 30 -19.401 -17.344 12.037 1.00 0.00 MEMB C

ATOM 3920 H2R POPC 30 -19.651 -18.313 12.528 1.00 0.00 MEMB H

ATOM 3921 H2S POPC 30 -18.551 -17.427 11.329 1.00 0.00 MEMB H

ATOM 3922 C3 POPC 30 -15.777 -16.481 13.728 1.00 0.00 MEMB C

ATOM 3923 HX POPC 30 -15.172 -17.399 13.731 1.00 0.00 MEMB H

ATOM 3924 HY POPC 30 -14.969 -16.184 14.260 1.00 0.00 MEMB H

ATOM 3925 O31 POPC 30 -15.677 -15.666 12.576 1.00 0.00 MEMB O

ATOM 3926 C31 POPC 30 -15.902 -14.352 12.717 1.00 0.00 MEMB C

ATOM 3927 O32 POPC 30 -16.206 -13.716 13.696 1.00 0.00 MEMB O

ATOM 3928 C32 POPC 30 -15.387 -13.627 11.570 1.00 0.00 MEMB C

ATOM 3929 H2X POPC 30 -14.848 -12.712 11.895 1.00 0.00 MEMB H

ATOM 3930 H2Y POPC 30 -16.282 -13.392 10.972 1.00 0.00 MEMB H

ATOM 3931 C23 POPC 30 -20.597 -16.689 11.413 1.00 0.00 MEMB C

ATOM 3932 H3R POPC 30 -21.275 -16.401 12.253 1.00 0.00 MEMB H

ATOM 3933 H3S POPC 30 -21.092 -17.471 10.811 1.00 0.00 MEMB H

ATOM 3934 C24 POPC 30 -20.209 -15.464 10.571 1.00 0.00 MEMB C

ATOM 3935 H4R POPC 30 -19.212 -15.071 10.880 1.00 0.00 MEMB H

ATOM 3936 H4S POPC 30 -20.946 -14.656 10.785 1.00 0.00 MEMB H

ATOM 3937 C25 POPC 30 -20.155 -15.776 9.078 1.00 0.00 MEMB C

ATOM 3938 H5R POPC 30 -21.025 -16.415 8.816 1.00 0.00 MEMB H

ATOM 3939 H5S POPC 30 -19.215 -16.325 8.840 1.00 0.00 MEMB H

ATOM 3940 C26 POPC 30 -20.244 -14.552 8.184 1.00 0.00 MEMB C

ATOM 3941 H6R POPC 30 -21.186 -13.990 8.381 1.00 0.00 MEMB H

ATOM 3942 H6S POPC 30 -20.270 -14.905 7.125 1.00 0.00 MEMB H

ATOM 3943 C27 POPC 30 -19.049 -13.633 8.309 1.00 0.00 MEMB C

ATOM 3944 H7R POPC 30 -18.126 -14.173 7.991 1.00 0.00 MEMB H

ATOM 3945 H7S POPC 30 -18.898 -13.220 9.329 1.00 0.00 MEMB H

ATOM 3946 C28 POPC 30 -19.271 -12.502 7.365 1.00 0.00 MEMB C

ATOM 3947 H8R POPC 30 -20.181 -11.921 7.531 1.00 0.00 MEMB H

ATOM 3948 H8S POPC 30 -19.378 -12.966 6.357 1.00 0.00 MEMB H

ATOM 3949 C29 POPC 30 -18.081 -11.657 7.544 1.00 0.00 MEMB C

ATOM 3950 H91 POPC 30 -17.996 -10.810 8.245 1.00 0.00 MEMB H

ATOM 3951 C210 POPC 30 -17.193 -11.823 6.641 1.00 0.00 MEMB C

ATOM 3952 H101 POPC 30 -16.420 -11.069 6.794 1.00 0.00 MEMB H

ATOM 3953 C211 POPC 30 -17.268 -12.791 5.438 1.00 0.00 MEMB C

ATOM 3954 H11R POPC 30 -17.239 -13.860 5.750 1.00 0.00 MEMB H

ATOM 3955 H11S POPC 30 -16.320 -12.551 5.185 1.00 0.00 MEMB H

ATOM 3956 C212 POPC 30 -17.163 -12.598 3.862 1.00 0.00 MEMB C

ATOM 3957 H12R POPC 30 -16.770 -11.666 3.572 1.00 0.00 MEMB H

ATOM 3958 H12S POPC 30 -18.128 -12.364 3.486 1.00 0.00 MEMB H

ATOM 3959 C213 POPC 30 -16.220 -13.406 2.936 1.00 0.00 MEMB C

ATOM 3960 H13R POPC 30 -15.150 -13.594 3.117 1.00 0.00 MEMB H

ATOM 3961 H13S POPC 30 -16.141 -12.874 1.957 1.00 0.00 MEMB H

ATOM 3962 C214 POPC 30 -16.991 -14.660 2.850 1.00 0.00 MEMB C

ATOM 3963 H14R POPC 30 -17.990 -14.340 2.486 1.00 0.00 MEMB H

ATOM 3964 H14S POPC 30 -17.048 -15.058 3.890 1.00 0.00 MEMB H

ATOM 3965 C215 POPC 30 -16.434 -15.659 1.918 1.00 0.00 MEMB C

ATOM 3966 H15R POPC 30 -17.106 -16.534 1.828 1.00 0.00 MEMB H

ATOM 3967 H15S POPC 30 -15.492 -16.004 2.284 1.00 0.00 MEMB H

ATOM 3968 C216 POPC 30 -15.729 -15.101 0.731 1.00 0.00 MEMB C

ATOM 3969 H16R POPC 30 -14.886 -14.415 0.986 1.00 0.00 MEMB H

ATOM 3970 H16S POPC 30 -16.464 -14.608 0.064 1.00 0.00 MEMB H

ATOM 3971 C217 POPC 30 -14.960 -16.213 0.110 1.00 0.00 MEMB C

ATOM 3972 H17R POPC 30 -15.786 -16.902 -0.131 1.00 0.00 MEMB H

ATOM 3973 H17S POPC 30 -14.250 -16.825 0.707 1.00 0.00 MEMB H

ATOM 3974 C218 POPC 30 -14.266 -15.573 -1.051 1.00 0.00 MEMB C

ATOM 3975 H18R POPC 30 -13.750 -16.287 -1.700 1.00 0.00 MEMB H

ATOM 3976 H18S POPC 30 -13.518 -14.883 -0.869 1.00 0.00 MEMB H

ATOM 3977 H18T POPC 30 -15.032 -14.993 -1.602 1.00 0.00 MEMB H

ATOM 3978 C33 POPC 30 -14.508 -14.389 10.697 1.00 0.00 MEMB C

ATOM 3979 H3X POPC 30 -14.616 -15.488 10.708 1.00 0.00 MEMB H

ATOM 3980 H3Y POPC 30 -13.452 -14.464 10.830 1.00 0.00 MEMB H

ATOM 3981 C34 POPC 30 -14.511 -13.528 9.511 1.00 0.00 MEMB C

ATOM 3982 H4X POPC 30 -13.824 -12.622 9.503 1.00 0.00 MEMB H

ATOM 3983 H4Y POPC 30 -15.451 -13.420 8.955 1.00 0.00 MEMB H

ATOM 3984 C35 POPC 30 -13.887 -14.559 8.861 1.00 0.00 MEMB C

ATOM 3985 H5X POPC 30 -12.820 -14.627 9.254 1.00 0.00 MEMB H

ATOM 3986 H5Y POPC 30 -12.977 -14.117 8.433 1.00 0.00 MEMB H

ATOM 3987 C36 POPC 30 -14.824 -15.912 8.489 1.00 0.00 MEMB C

ATOM 3988 H6X POPC 30 -14.866 -16.669 9.281 1.00 0.00 MEMB H

ATOM 3989 H6Y POPC 30 -14.377 -16.680 7.838 1.00 0.00 MEMB H

ATOM 3990 C37 POPC 30 -15.874 -16.017 7.354 1.00 0.00 MEMB C

ATOM 3991 H7X POPC 30 -15.656 -15.666 6.309 1.00 0.00 MEMB H

ATOM 3992 H7Y POPC 30 -16.708 -15.479 7.765 1.00 0.00 MEMB H

ATOM 3993 C38 POPC 30 -16.148 -17.463 6.985 1.00 0.00 MEMB C

ATOM 3994 H8X POPC 30 -16.494 -17.904 7.933 1.00 0.00 MEMB H

ATOM 3995 H8Y POPC 30 -15.240 -18.029 6.634 1.00 0.00 MEMB H

ATOM 3996 C39 POPC 30 -17.139 -17.486 5.854 1.00 0.00 MEMB C

ATOM 3997 H9X POPC 30 -16.582 -17.402 4.897 1.00 0.00 MEMB H

ATOM 3998 H9Y POPC 30 -17.808 -16.606 5.887 1.00 0.00 MEMB H

ATOM 3999 C310 POPC 30 -17.970 -18.732 5.912 1.00 0.00 MEMB C

ATOM 4000 H10X POPC 30 -18.653 -18.762 6.764 1.00 0.00 MEMB H

ATOM 4001 H10Y POPC 30 -17.363 -19.619 6.017 1.00 0.00 MEMB H

ATOM 4002 C311 POPC 30 -18.684 -18.968 4.636 1.00 0.00 MEMB C

ATOM 4003 H11X POPC 30 -18.668 -20.044 4.352 1.00 0.00 MEMB H

ATOM 4004 H11Y POPC 30 -18.185 -18.382 3.834 1.00 0.00 MEMB H

ATOM 4005 C312 POPC 30 -20.067 -18.586 4.910 1.00 0.00 MEMB C

ATOM 4006 H12X POPC 30 -20.056 -17.588 5.392 1.00 0.00 MEMB H

ATOM 4007 H12Y POPC 30 -20.455 -19.319 5.660 1.00 0.00 MEMB H

ATOM 4008 C313 POPC 30 -20.760 -18.559 3.589 1.00 0.00 MEMB C

ATOM 4009 H13X POPC 30 -21.739 -18.170 3.797 1.00 0.00 MEMB H

ATOM 4010 H13Y POPC 30 -20.870 -19.522 3.074 1.00 0.00 MEMB H

ATOM 4011 C314 POPC 30 -20.180 -17.636 2.563 1.00 0.00 MEMB C

ATOM 4012 H14X POPC 30 -19.251 -18.052 2.117 1.00 0.00 MEMB H

ATOM 4013 H14Y POPC 30 -20.080 -16.648 3.053 1.00 0.00 MEMB H

ATOM 4014 C315 POPC 30 -21.228 -17.487 1.531 1.00 0.00 MEMB C

ATOM 4015 H15X POPC 30 -21.099 -16.672 0.817 1.00 0.00 MEMB H

ATOM 4016 H15Y POPC 30 -22.108 -17.196 2.103 1.00 0.00 MEMB H

ATOM 4017 C316 POPC 30 -21.529 -18.738 0.816 1.00 0.00 MEMB C

ATOM 4018 H16X POPC 30 -21.921 -19.564 1.412 1.00 0.00 MEMB H

ATOM 4019 H16Y POPC 30 -20.693 -19.084 0.153 1.00 0.00 MEMB H

ATOM 4020 H16Z POPC 30 -22.432 -18.492 0.257 1.00 0.00 MEMB H

ATOM 4021 N POPC 31 5.407 25.341 20.957 1.00 0.00 MEMB N

ATOM 4022 C12 POPC 31 4.378 26.258 20.300 1.00 0.00 MEMB C

ATOM 4023 H12A POPC 31 4.099 27.032 20.999 1.00 0.00 MEMB H

ATOM 4024 H12B POPC 31 3.503 25.657 20.065 1.00 0.00 MEMB H

ATOM 4025 C13 POPC 31 5.532 24.138 20.101 1.00 0.00 MEMB C

ATOM 4026 H13A POPC 31 6.519 23.724 20.170 1.00 0.00 MEMB H

ATOM 4027 H13B POPC 31 4.777 23.418 20.366 1.00 0.00 MEMB H

ATOM 4028 H13C POPC 31 5.348 24.446 19.063 1.00 0.00 MEMB H

ATOM 4029 C14 POPC 31 6.740 26.015 21.058 1.00 0.00 MEMB C

ATOM 4030 H14A POPC 31 7.488 25.316 21.397 1.00 0.00 MEMB H

ATOM 4031 H14B POPC 31 6.993 26.385 20.068 1.00 0.00 MEMB H

ATOM 4032 H14C POPC 31 6.704 26.854 21.753 1.00 0.00 MEMB H

ATOM 4033 C15 POPC 31 4.981 24.895 22.306 1.00 0.00 MEMB C

ATOM 4034 H15A POPC 31 5.744 24.286 22.763 1.00 0.00 MEMB H

ATOM 4035 H15B POPC 31 4.059 24.318 22.281 1.00 0.00 MEMB H

ATOM 4036 H15C POPC 31 4.817 25.756 22.951 1.00 0.00 MEMB H

ATOM 4037 C11 POPC 31 4.777 26.964 18.971 1.00 0.00 MEMB C

ATOM 4038 H11A POPC 31 5.695 27.562 19.147 1.00 0.00 MEMB H

ATOM 4039 H11B POPC 31 3.984 27.707 18.697 1.00 0.00 MEMB H

ATOM 4040 P POPC 31 3.851 25.652 16.907 1.00 0.00 MEMB P

ATOM 4041 O13 POPC 31 2.759 25.167 17.781 1.00 0.00 MEMB O

ATOM 4042 O14 POPC 31 4.376 24.749 15.866 1.00 0.00 MEMB O

ATOM 4043 O12 POPC 31 5.027 26.035 17.911 1.00 0.00 MEMB O

ATOM 4044 O11 POPC 31 3.440 27.052 16.286 1.00 0.00 MEMB O

ATOM 4045 C1 POPC 31 4.456 27.810 15.626 1.00 0.00 MEMB C

ATOM 4046 HA POPC 31 4.878 27.244 14.757 1.00 0.00 MEMB H

ATOM 4047 HB POPC 31 5.257 28.142 16.318 1.00 0.00 MEMB H

ATOM 4048 C2 POPC 31 3.886 29.062 14.980 1.00 0.00 MEMB C

ATOM 4049 HS POPC 31 3.367 29.653 15.785 1.00 0.00 MEMB H

ATOM 4050 O21 POPC 31 3.092 28.580 13.946 1.00 0.00 MEMB O

ATOM 4051 C21 POPC 31 1.936 28.933 13.978 1.00 0.00 MEMB C

ATOM 4052 O22 POPC 31 1.393 29.656 14.818 1.00 0.00 MEMB O

ATOM 4053 C22 POPC 31 1.489 28.191 12.690 1.00 0.00 MEMB C

ATOM 4054 H2R POPC 31 0.458 27.964 12.687 1.00 0.00 MEMB H

ATOM 4055 H2S POPC 31 1.729 27.136 12.824 1.00 0.00 MEMB H

ATOM 4056 C3 POPC 31 4.964 29.899 14.353 1.00 0.00 MEMB C

ATOM 4057 HX POPC 31 5.738 29.884 15.084 1.00 0.00 MEMB H

ATOM 4058 HY POPC 31 4.829 30.958 14.270 1.00 0.00 MEMB H

ATOM 4059 O31 POPC 31 5.380 29.269 13.141 1.00 0.00 MEMB O

ATOM 4060 C31 POPC 31 5.520 29.973 12.066 1.00 0.00 MEMB C

ATOM 4061 O32 POPC 31 5.592 31.176 11.978 1.00 0.00 MEMB O

ATOM 4062 C32 POPC 31 5.776 28.983 10.926 1.00 0.00 MEMB C

ATOM 4063 H2X POPC 31 5.247 28.039 11.014 1.00 0.00 MEMB H

ATOM 4064 H2Y POPC 31 6.829 28.774 11.163 1.00 0.00 MEMB H

ATOM 4065 C23 POPC 31 2.079 28.662 11.308 1.00 0.00 MEMB C

ATOM 4066 H3R POPC 31 3.163 28.497 11.318 1.00 0.00 MEMB H

ATOM 4067 H3S POPC 31 2.011 29.779 11.285 1.00 0.00 MEMB H

ATOM 4068 C24 POPC 31 1.551 27.873 10.033 1.00 0.00 MEMB C

ATOM 4069 H4R POPC 31 0.494 27.802 9.804 1.00 0.00 MEMB H

ATOM 4070 H4S POPC 31 1.564 26.802 10.131 1.00 0.00 MEMB H

ATOM 4071 C25 POPC 31 1.946 28.485 8.701 1.00 0.00 MEMB C

ATOM 4072 H5R POPC 31 3.034 28.420 8.584 1.00 0.00 MEMB H

ATOM 4073 H5S POPC 31 1.591 29.486 9.038 1.00 0.00 MEMB H

ATOM 4074 C26 POPC 31 1.085 28.297 7.419 1.00 0.00 MEMB C

ATOM 4075 H6R POPC 31 0.412 29.173 7.273 1.00 0.00 MEMB H

ATOM 4076 H6S POPC 31 0.557 27.387 7.685 1.00 0.00 MEMB H

ATOM 4077 C27 POPC 31 1.695 28.279 6.023 1.00 0.00 MEMB C

ATOM 4078 H7R POPC 31 2.677 27.878 6.018 1.00 0.00 MEMB H

ATOM 4079 H7S POPC 31 1.769 29.292 5.771 1.00 0.00 MEMB H

ATOM 4080 C28 POPC 31 1.021 28.720 4.728 1.00 0.00 MEMB C

ATOM 4081 H8R POPC 31 1.692 29.132 3.935 1.00 0.00 MEMB H

ATOM 4082 H8S POPC 31 0.106 29.355 4.919 1.00 0.00 MEMB H

ATOM 4083 C29 POPC 31 0.457 27.757 4.068 1.00 0.00 MEMB C

ATOM 4084 H91 POPC 31 -0.208 27.622 4.876 1.00 0.00 MEMB H

ATOM 4085 C210 POPC 31 1.138 26.813 3.616 1.00 0.00 MEMB C

ATOM 4086 H101 POPC 31 0.561 25.957 3.607 1.00 0.00 MEMB H

ATOM 4087 C211 POPC 31 2.560 26.642 3.730 1.00 0.00 MEMB C

ATOM 4088 H11R POPC 31 3.034 26.352 4.706 1.00 0.00 MEMB H

ATOM 4089 H11S POPC 31 3.002 27.514 3.289 1.00 0.00 MEMB H

ATOM 4090 C212 POPC 31 2.531 25.435 2.864 1.00 0.00 MEMB C

ATOM 4091 H12R POPC 31 2.633 24.498 3.221 1.00 0.00 MEMB H

ATOM 4092 H12S POPC 31 3.547 25.165 3.170 1.00 0.00 MEMB H

ATOM 4093 C213 POPC 31 1.915 25.491 1.444 1.00 0.00 MEMB C

ATOM 4094 H13R POPC 31 2.083 26.556 1.216 1.00 0.00 MEMB H

ATOM 4095 H13S POPC 31 0.830 25.266 1.589 1.00 0.00 MEMB H

ATOM 4096 C214 POPC 31 2.396 24.662 0.216 1.00 0.00 MEMB C

ATOM 4097 H14R POPC 31 2.681 23.735 0.557 1.00 0.00 MEMB H

ATOM 4098 H14S POPC 31 3.407 24.726 -0.156 1.00 0.00 MEMB H

ATOM 4099 C215 POPC 31 1.584 24.868 -1.037 1.00 0.00 MEMB C

ATOM 4100 H15R POPC 31 2.030 25.861 -1.249 1.00 0.00 MEMB H

ATOM 4101 H15S POPC 31 0.502 24.810 -0.875 1.00 0.00 MEMB H

ATOM 4102 C216 POPC 31 1.938 24.403 -2.427 1.00 0.00 MEMB C

ATOM 4103 H16R POPC 31 1.251 24.628 -3.248 1.00 0.00 MEMB H

ATOM 4104 H16S POPC 31 2.252 23.316 -2.284 1.00 0.00 MEMB H

ATOM 4105 C217 POPC 31 3.160 24.980 -3.032 1.00 0.00 MEMB C

ATOM 4106 H17R POPC 31 3.475 24.111 -3.518 1.00 0.00 MEMB H

ATOM 4107 H17S POPC 31 3.361 24.753 -2.045 1.00 0.00 MEMB H

ATOM 4108 C218 POPC 31 3.771 26.425 -3.383 1.00 0.00 MEMB C

ATOM 4109 H18R POPC 31 4.682 26.602 -4.032 1.00 0.00 MEMB H

ATOM 4110 H18S POPC 31 4.054 27.114 -2.560 1.00 0.00 MEMB H

ATOM 4111 H18T POPC 31 2.848 26.379 -3.824 1.00 0.00 MEMB H

ATOM 4112 C33 POPC 31 5.510 29.550 9.505 1.00 0.00 MEMB C

ATOM 4113 H3X POPC 31 5.709 30.618 9.673 1.00 0.00 MEMB H

ATOM 4114 H3Y POPC 31 4.509 29.766 9.372 1.00 0.00 MEMB H

ATOM 4115 C34 POPC 31 6.001 28.779 8.198 1.00 0.00 MEMB C

ATOM 4116 H4X POPC 31 5.858 27.705 8.275 1.00 0.00 MEMB H

ATOM 4117 H4Y POPC 31 7.055 28.754 7.971 1.00 0.00 MEMB H

ATOM 4118 C35 POPC 31 5.933 29.369 6.774 1.00 0.00 MEMB C

ATOM 4119 H5X POPC 31 6.590 28.833 6.063 1.00 0.00 MEMB H

ATOM 4120 H5Y POPC 31 6.104 30.454 6.658 1.00 0.00 MEMB H

ATOM 4121 C36 POPC 31 4.613 29.269 6.293 1.00 0.00 MEMB C

ATOM 4122 H6X POPC 31 4.296 29.935 7.104 1.00 0.00 MEMB H

ATOM 4123 H6Y POPC 31 4.393 28.221 6.355 1.00 0.00 MEMB H

ATOM 4124 C37 POPC 31 4.356 29.882 4.987 1.00 0.00 MEMB C

ATOM 4125 H7X POPC 31 4.599 30.890 5.370 1.00 0.00 MEMB H

ATOM 4126 H7Y POPC 31 3.324 29.964 4.631 1.00 0.00 MEMB H

ATOM 4127 C38 POPC 31 5.311 29.661 3.880 1.00 0.00 MEMB C

ATOM 4128 H8X POPC 31 6.321 29.584 4.198 1.00 0.00 MEMB H

ATOM 4129 H8Y POPC 31 5.438 30.451 3.148 1.00 0.00 MEMB H

ATOM 4130 C39 POPC 31 5.142 28.597 3.064 1.00 0.00 MEMB C

ATOM 4131 H9X POPC 31 4.129 28.786 2.642 1.00 0.00 MEMB H

ATOM 4132 H9Y POPC 31 5.243 27.841 3.886 1.00 0.00 MEMB H

ATOM 4133 C310 POPC 31 6.340 28.856 2.103 1.00 0.00 MEMB C

ATOM 4134 H10X POPC 31 7.235 29.338 2.524 1.00 0.00 MEMB H

ATOM 4135 H10Y POPC 31 5.963 29.446 1.232 1.00 0.00 MEMB H

ATOM 4136 C311 POPC 31 7.051 27.614 1.754 1.00 0.00 MEMB C

ATOM 4137 H11X POPC 31 7.268 27.547 0.693 1.00 0.00 MEMB H

ATOM 4138 H11Y POPC 31 6.093 27.315 1.879 1.00 0.00 MEMB H

ATOM 4139 C312 POPC 31 7.607 26.680 2.830 1.00 0.00 MEMB C

ATOM 4140 H12X POPC 31 6.930 26.611 3.716 1.00 0.00 MEMB H

ATOM 4141 H12Y POPC 31 8.538 27.211 3.047 1.00 0.00 MEMB H

ATOM 4142 C313 POPC 31 7.994 25.254 2.424 1.00 0.00 MEMB C

ATOM 4143 H13X POPC 31 8.687 24.791 3.134 1.00 0.00 MEMB H

ATOM 4144 H13Y POPC 31 8.416 25.384 1.396 1.00 0.00 MEMB H

ATOM 4145 C314 POPC 31 6.903 24.216 2.445 1.00 0.00 MEMB C

ATOM 4146 H14X POPC 31 6.617 24.614 1.473 1.00 0.00 MEMB H

ATOM 4147 H14Y POPC 31 6.028 24.234 3.117 1.00 0.00 MEMB H

ATOM 4148 C315 POPC 31 7.034 22.824 2.058 1.00 0.00 MEMB C

ATOM 4149 H15X POPC 31 7.499 22.072 2.685 1.00 0.00 MEMB H

ATOM 4150 H15Y POPC 31 7.783 22.872 1.237 1.00 0.00 MEMB H

ATOM 4151 C316 POPC 31 5.586 22.393 1.760 1.00 0.00 MEMB C

ATOM 4152 H16X POPC 31 4.853 22.744 0.986 1.00 0.00 MEMB H

ATOM 4153 H16Y POPC 31 4.964 21.966 2.546 1.00 0.00 MEMB H

ATOM 4154 H16Z POPC 31 6.020 21.659 1.102 1.00 0.00 MEMB H

ATOM 4155 N POPC 32 1.879 -26.946 19.497 1.00 0.00 MEMB N

ATOM 4156 C12 POPC 32 2.428 -25.720 20.212 1.00 0.00 MEMB C

ATOM 4157 H12A POPC 32 3.497 -25.721 20.103 1.00 0.00 MEMB H

ATOM 4158 H12B POPC 32 2.150 -25.773 21.250 1.00 0.00 MEMB H

ATOM 4159 C13 POPC 32 2.214 -26.856 18.036 1.00 0.00 MEMB C

ATOM 4160 H13A POPC 32 1.751 -25.969 17.601 1.00 0.00 MEMB H

ATOM 4161 H13B POPC 32 3.286 -26.798 17.907 1.00 0.00 MEMB H

ATOM 4162 H13C POPC 32 1.871 -27.722 17.478 1.00 0.00 MEMB H

ATOM 4163 C14 POPC 32 2.522 -28.180 20.025 1.00 0.00 MEMB C

ATOM 4164 H14A POPC 32 3.558 -28.206 19.731 1.00 0.00 MEMB H

ATOM 4165 H14B POPC 32 2.425 -28.257 21.097 1.00 0.00 MEMB H

ATOM 4166 H14C POPC 32 2.066 -29.070 19.602 1.00 0.00 MEMB H

ATOM 4167 C15 POPC 32 0.392 -27.082 19.679 1.00 0.00 MEMB C

ATOM 4168 H15A POPC 32 -0.121 -26.343 19.070 1.00 0.00 MEMB H

ATOM 4169 H15B POPC 32 0.072 -26.879 20.693 1.00 0.00 MEMB H

ATOM 4170 H15C POPC 32 0.028 -28.076 19.441 1.00 0.00 MEMB H

ATOM 4171 C11 POPC 32 2.047 -24.306 19.760 1.00 0.00 MEMB C

ATOM 4172 H11A POPC 32 2.729 -23.983 18.936 1.00 0.00 MEMB H

ATOM 4173 H11B POPC 32 2.166 -23.589 20.590 1.00 0.00 MEMB H

ATOM 4174 P POPC 32 0.251 -23.250 18.326 1.00 0.00 MEMB P

ATOM 4175 O13 POPC 32 1.262 -22.188 18.124 1.00 0.00 MEMB O

ATOM 4176 O14 POPC 32 -1.126 -22.966 18.727 1.00 0.00 MEMB O

ATOM 4177 O12 POPC 32 0.701 -24.280 19.405 1.00 0.00 MEMB O

ATOM 4178 O11 POPC 32 0.287 -24.179 17.074 1.00 0.00 MEMB O

ATOM 4179 C1 POPC 32 -0.815 -25.047 16.894 1.00 0.00 MEMB C

ATOM 4180 HA POPC 32 -1.801 -24.516 16.936 1.00 0.00 MEMB H

ATOM 4181 HB POPC 32 -0.819 -25.850 17.658 1.00 0.00 MEMB H

ATOM 4182 C2 POPC 32 -0.683 -25.689 15.523 1.00 0.00 MEMB C

ATOM 4183 HS POPC 32 0.209 -26.361 15.540 1.00 0.00 MEMB H

ATOM 4184 O21 POPC 32 -0.574 -24.670 14.508 1.00 0.00 MEMB O

ATOM 4185 C21 POPC 32 0.216 -24.971 13.494 1.00 0.00 MEMB C

ATOM 4186 O22 POPC 32 0.853 -26.001 13.370 1.00 0.00 MEMB O

ATOM 4187 C22 POPC 32 0.179 -23.816 12.488 1.00 0.00 MEMB C

ATOM 4188 H2R POPC 32 -0.027 -22.892 13.063 1.00 0.00 MEMB H

ATOM 4189 H2S POPC 32 -0.665 -23.975 11.788 1.00 0.00 MEMB H

ATOM 4190 C3 POPC 32 -1.922 -26.549 15.316 1.00 0.00 MEMB C

ATOM 4191 HX POPC 32 -2.829 -25.992 15.654 1.00 0.00 MEMB H

ATOM 4192 HY POPC 32 -1.807 -27.459 15.950 1.00 0.00 MEMB H

ATOM 4193 O31 POPC 32 -1.965 -26.900 13.946 1.00 0.00 MEMB O

ATOM 4194 C31 POPC 32 -3.028 -26.541 13.277 1.00 0.00 MEMB C

ATOM 4195 O32 POPC 32 -4.006 -25.960 13.678 1.00 0.00 MEMB O

ATOM 4196 C32 POPC 32 -2.988 -27.194 11.953 1.00 0.00 MEMB C

ATOM 4197 H2X POPC 32 -3.740 -27.987 12.127 1.00 0.00 MEMB H

ATOM 4198 H2Y POPC 32 -1.953 -27.551 11.762 1.00 0.00 MEMB H

ATOM 4199 C23 POPC 32 1.532 -23.609 11.767 1.00 0.00 MEMB C

ATOM 4200 H3R POPC 32 2.350 -23.656 12.523 1.00 0.00 MEMB H

ATOM 4201 H3S POPC 32 1.559 -22.584 11.332 1.00 0.00 MEMB H

ATOM 4202 C24 POPC 32 1.864 -24.616 10.656 1.00 0.00 MEMB C

ATOM 4203 H4R POPC 32 2.091 -25.602 11.123 1.00 0.00 MEMB H

ATOM 4204 H4S POPC 32 2.774 -24.258 10.131 1.00 0.00 MEMB H

ATOM 4205 C25 POPC 32 0.791 -24.849 9.593 1.00 0.00 MEMB C

ATOM 4206 H5R POPC 32 0.457 -23.880 9.159 1.00 0.00 MEMB H

ATOM 4207 H5S POPC 32 -0.082 -25.310 10.110 1.00 0.00 MEMB H

ATOM 4208 C26 POPC 32 1.278 -25.816 8.489 1.00 0.00 MEMB C

ATOM 4209 H6R POPC 32 0.501 -26.609 8.425 1.00 0.00 MEMB H

ATOM 4210 H6S POPC 32 2.220 -26.319 8.797 1.00 0.00 MEMB H

ATOM 4211 C27 POPC 32 1.459 -25.150 7.105 1.00 0.00 MEMB C

ATOM 4212 H7R POPC 32 2.261 -24.384 7.153 1.00 0.00 MEMB H

ATOM 4213 H7S POPC 32 0.502 -24.630 6.878 1.00 0.00 MEMB H

ATOM 4214 C28 POPC 32 1.726 -26.082 5.901 1.00 0.00 MEMB C

ATOM 4215 H8R POPC 32 2.752 -26.507 5.922 1.00 0.00 MEMB H

ATOM 4216 H8S POPC 32 1.660 -25.453 4.982 1.00 0.00 MEMB H

ATOM 4217 C29 POPC 32 0.691 -27.174 5.941 1.00 0.00 MEMB C

ATOM 4218 H91 POPC 32 0.844 -27.913 6.737 1.00 0.00 MEMB H

ATOM 4219 C210 POPC 32 -0.354 -27.344 5.132 1.00 0.00 MEMB C

ATOM 4220 H101 POPC 32 -1.081 -28.143 5.346 1.00 0.00 MEMB H

ATOM 4221 C211 POPC 32 -0.748 -26.494 3.976 1.00 0.00 MEMB C

ATOM 4222 H11R POPC 32 -0.215 -25.522 3.863 1.00 0.00 MEMB H

ATOM 4223 H11S POPC 32 -1.818 -26.249 4.135 1.00 0.00 MEMB H

ATOM 4224 C212 POPC 32 -0.516 -27.302 2.688 1.00 0.00 MEMB C

ATOM 4225 H12R POPC 32 -0.699 -28.378 2.832 1.00 0.00 MEMB H

ATOM 4226 H12S POPC 32 0.522 -27.170 2.327 1.00 0.00 MEMB H

ATOM 4227 C213 POPC 32 -1.428 -26.713 1.642 1.00 0.00 MEMB C

ATOM 4228 H13R POPC 32 -1.016 -25.700 1.470 1.00 0.00 MEMB H

ATOM 4229 H13S POPC 32 -2.297 -26.423 2.243 1.00 0.00 MEMB H

ATOM 4230 C214 POPC 32 -1.853 -27.481 0.327 1.00 0.00 MEMB C

ATOM 4231 H14R POPC 32 -2.403 -26.986 -0.490 1.00 0.00 MEMB H

ATOM 4232 H14S POPC 32 -2.516 -28.334 0.428 1.00 0.00 MEMB H

ATOM 4233 C215 POPC 32 -0.834 -27.763 -0.558 1.00 0.00 MEMB C

ATOM 4234 H15R POPC 32 -1.100 -28.180 -1.546 1.00 0.00 MEMB H

ATOM 4235 H15S POPC 32 -0.671 -28.554 0.154 1.00 0.00 MEMB H

ATOM 4236 C216 POPC 32 0.105 -26.651 -0.548 1.00 0.00 MEMB C

ATOM 4237 H16R POPC 32 0.562 -26.331 0.420 1.00 0.00 MEMB H

ATOM 4238 H16S POPC 32 -0.193 -25.690 -0.958 1.00 0.00 MEMB H

ATOM 4239 C217 POPC 32 1.151 -27.086 -1.428 1.00 0.00 MEMB C

ATOM 4240 H17R POPC 32 1.988 -27.577 -1.021 1.00 0.00 MEMB H

ATOM 4241 H17S POPC 32 1.486 -26.036 -1.454 1.00 0.00 MEMB H

ATOM 4242 C218 POPC 32 1.127 -27.883 -2.713 1.00 0.00 MEMB C

ATOM 4243 H18R POPC 32 0.232 -28.421 -3.018 1.00 0.00 MEMB H

ATOM 4244 H18S POPC 32 2.036 -28.254 -3.227 1.00 0.00 MEMB H

ATOM 4245 H18T POPC 32 1.284 -26.972 -3.235 1.00 0.00 MEMB H

ATOM 4246 C33 POPC 32 -3.626 -26.458 10.810 1.00 0.00 MEMB C

ATOM 4247 H3X POPC 32 -3.374 -25.393 10.918 1.00 0.00 MEMB H

ATOM 4248 H3Y POPC 32 -4.736 -26.526 10.974 1.00 0.00 MEMB H

ATOM 4249 C34 POPC 32 -3.240 -27.074 9.443 1.00 0.00 MEMB C

ATOM 4250 H4X POPC 32 -3.352 -28.171 9.342 1.00 0.00 MEMB H

ATOM 4251 H4Y POPC 32 -2.170 -26.904 9.226 1.00 0.00 MEMB H

ATOM 4252 C35 POPC 32 -4.174 -26.517 8.408 1.00 0.00 MEMB C

ATOM 4253 H5X POPC 32 -5.239 -26.564 8.770 1.00 0.00 MEMB H

ATOM 4254 H5Y POPC 32 -4.164 -27.050 7.430 1.00 0.00 MEMB H

ATOM 4255 C36 POPC 32 -3.752 -25.077 8.207 1.00 0.00 MEMB C

ATOM 4256 H6X POPC 32 -3.795 -24.454 9.114 1.00 0.00 MEMB H

ATOM 4257 H6Y POPC 32 -4.654 -24.750 7.637 1.00 0.00 MEMB H

ATOM 4258 C37 POPC 32 -2.287 -24.831 7.553 1.00 0.00 MEMB C

ATOM 4259 H7X POPC 32 -2.308 -25.526 6.683 1.00 0.00 MEMB H

ATOM 4260 H7Y POPC 32 -1.435 -25.077 8.184 1.00 0.00 MEMB H

ATOM 4261 C38 POPC 32 -1.839 -23.308 7.218 1.00 0.00 MEMB C

ATOM 4262 H8X POPC 32 -1.961 -22.327 7.632 1.00 0.00 MEMB H

ATOM 4263 H8Y POPC 32 -2.896 -23.391 7.395 1.00 0.00 MEMB H

ATOM 4264 C39 POPC 32 -1.092 -22.649 5.914 1.00 0.00 MEMB C

ATOM 4265 H9X POPC 32 -0.873 -23.415 5.165 1.00 0.00 MEMB H

ATOM 4266 H9Y POPC 32 -0.155 -22.354 6.391 1.00 0.00 MEMB H

ATOM 4267 C310 POPC 32 -1.553 -21.471 4.905 1.00 0.00 MEMB C

ATOM 4268 H10X POPC 32 -1.786 -20.486 5.330 1.00 0.00 MEMB H

ATOM 4269 H10Y POPC 32 -2.471 -21.555 4.332 1.00 0.00 MEMB H

ATOM 4270 C311 POPC 32 -0.644 -21.231 3.686 1.00 0.00 MEMB C

ATOM 4271 H11X POPC 32 0.306 -20.849 4.089 1.00 0.00 MEMB H

ATOM 4272 H11Y POPC 32 -1.112 -20.454 3.043 1.00 0.00 MEMB H

ATOM 4273 C312 POPC 32 -0.388 -22.356 2.704 1.00 0.00 MEMB C

ATOM 4274 H12X POPC 32 -1.236 -22.736 2.141 1.00 0.00 MEMB H

ATOM 4275 H12Y POPC 32 0.131 -23.243 3.113 1.00 0.00 MEMB H

ATOM 4276 C313 POPC 32 0.376 -21.824 1.563 1.00 0.00 MEMB C

ATOM 4277 H13X POPC 32 1.306 -21.351 1.977 1.00 0.00 MEMB H

ATOM 4278 H13Y POPC 32 -0.136 -21.036 0.960 1.00 0.00 MEMB H

ATOM 4279 C314 POPC 32 0.615 -23.067 0.694 1.00 0.00 MEMB C

ATOM 4280 H14X POPC 32 -0.191 -23.635 0.220 1.00 0.00 MEMB H

ATOM 4281 H14Y POPC 32 1.367 -23.707 1.247 1.00 0.00 MEMB H

ATOM 4282 C315 POPC 32 1.287 -22.730 -0.547 1.00 0.00 MEMB C

ATOM 4283 H15X POPC 32 2.036 -23.539 -0.703 1.00 0.00 MEMB H

ATOM 4284 H15Y POPC 32 1.651 -21.890 0.035 1.00 0.00 MEMB H

ATOM 4285 C316 POPC 32 0.896 -22.010 -1.816 1.00 0.00 MEMB C

ATOM 4286 H16X POPC 32 0.128 -22.596 -2.297 1.00 0.00 MEMB H

ATOM 4287 H16Y POPC 32 1.747 -22.101 -2.537 1.00 0.00 MEMB H

ATOM 4288 H16Z POPC 32 0.678 -20.928 -1.692 1.00 0.00 MEMB H

ATOM 4289 N POPC 33 -13.068 -23.199 17.120 1.00 0.00 MEMB N

ATOM 4290 C12 POPC 33 -14.276 -22.423 17.630 1.00 0.00 MEMB C

ATOM 4291 H12A POPC 33 -14.968 -23.130 18.077 1.00 0.00 MEMB H

ATOM 4292 H12B POPC 33 -14.762 -21.962 16.777 1.00 0.00 MEMB H

ATOM 4293 C13 POPC 33 -12.681 -24.198 18.153 1.00 0.00 MEMB C

ATOM 4294 H13A POPC 33 -11.893 -24.843 17.802 1.00 0.00 MEMB H

ATOM 4295 H13B POPC 33 -12.342 -23.658 19.043 1.00 0.00 MEMB H

ATOM 4296 H13C POPC 33 -13.525 -24.805 18.442 1.00 0.00 MEMB H

ATOM 4297 C14 POPC 33 -13.462 -23.887 15.867 1.00 0.00 MEMB C

ATOM 4298 H14A POPC 33 -12.771 -24.681 15.632 1.00 0.00 MEMB H

ATOM 4299 H14B POPC 33 -14.461 -24.307 15.951 1.00 0.00 MEMB H

ATOM 4300 H14C POPC 33 -13.462 -23.182 15.039 1.00 0.00 MEMB H

ATOM 4301 C15 POPC 33 -11.859 -22.354 16.868 1.00 0.00 MEMB C

ATOM 4302 H15A POPC 33 -12.042 -21.582 16.134 1.00 0.00 MEMB H

ATOM 4303 H15B POPC 33 -11.036 -22.963 16.515 1.00 0.00 MEMB H

ATOM 4304 H15C POPC 33 -11.556 -21.873 17.800 1.00 0.00 MEMB H

ATOM 4305 C11 POPC 33 -14.096 -21.328 18.698 1.00 0.00 MEMB C

ATOM 4306 H11A POPC 33 -15.107 -20.923 18.956 1.00 0.00 MEMB H

ATOM 4307 H11B POPC 33 -13.508 -20.527 18.189 1.00 0.00 MEMB H

ATOM 4308 P POPC 33 -12.851 -20.766 20.918 1.00 0.00 MEMB P

ATOM 4309 O13 POPC 33 -11.529 -21.322 21.312 1.00 0.00 MEMB O

ATOM 4310 O14 POPC 33 -13.842 -20.471 21.989 1.00 0.00 MEMB O

ATOM 4311 O12 POPC 33 -13.476 -21.819 19.881 1.00 0.00 MEMB O

ATOM 4312 O11 POPC 33 -12.692 -19.468 20.023 1.00 0.00 MEMB O

ATOM 4313 C1 POPC 33 -11.388 -18.981 19.763 1.00 0.00 MEMB C

ATOM 4314 HA POPC 33 -10.794 -19.795 19.321 1.00 0.00 MEMB H

ATOM 4315 HB POPC 33 -10.942 -18.593 20.706 1.00 0.00 MEMB H

ATOM 4316 C2 POPC 33 -11.324 -17.874 18.691 1.00 0.00 MEMB C

ATOM 4317 HS POPC 33 -11.656 -16.958 19.201 1.00 0.00 MEMB H

ATOM 4318 O21 POPC 33 -12.105 -18.203 17.567 1.00 0.00 MEMB O

ATOM 4319 C21 POPC 33 -13.347 -17.918 17.689 1.00 0.00 MEMB C

ATOM 4320 O22 POPC 33 -13.923 -17.344 18.597 1.00 0.00 MEMB O

ATOM 4321 C22 POPC 33 -13.948 -18.344 16.359 1.00 0.00 MEMB C

ATOM 4322 H2R POPC 33 -13.235 -18.945 15.791 1.00 0.00 MEMB H

ATOM 4323 H2S POPC 33 -14.008 -17.482 15.700 1.00 0.00 MEMB H

ATOM 4324 C3 POPC 33 -9.930 -17.585 18.125 1.00 0.00 MEMB C

ATOM 4325 HX POPC 33 -9.410 -17.146 18.993 1.00 0.00 MEMB H

ATOM 4326 HY POPC 33 -9.991 -16.780 17.354 1.00 0.00 MEMB H

ATOM 4327 O31 POPC 33 -9.230 -18.767 17.653 1.00 0.00 MEMB O

ATOM 4328 C31 POPC 33 -9.322 -18.970 16.357 1.00 0.00 MEMB C

ATOM 4329 O32 POPC 33 -9.956 -18.312 15.552 1.00 0.00 MEMB O

ATOM 4330 C32 POPC 33 -8.393 -20.099 15.963 1.00 0.00 MEMB C

ATOM 4331 H2X POPC 33 -8.136 -20.738 16.829 1.00 0.00 MEMB H

ATOM 4332 H2Y POPC 33 -7.454 -19.655 15.562 1.00 0.00 MEMB H

ATOM 4333 C23 POPC 33 -15.332 -18.964 16.548 1.00 0.00 MEMB C

ATOM 4334 H3R POPC 33 -15.871 -18.253 17.232 1.00 0.00 MEMB H

ATOM 4335 H3S POPC 33 -15.268 -19.872 17.139 1.00 0.00 MEMB H

ATOM 4336 C24 POPC 33 -16.060 -19.376 15.237 1.00 0.00 MEMB C

ATOM 4337 H4R POPC 33 -16.336 -18.541 14.634 1.00 0.00 MEMB H

ATOM 4338 H4S POPC 33 -17.103 -19.704 15.411 1.00 0.00 MEMB H

ATOM 4339 C25 POPC 33 -15.205 -19.993 14.159 1.00 0.00 MEMB C

ATOM 4340 H5R POPC 33 -15.176 -21.064 14.415 1.00 0.00 MEMB H

ATOM 4341 H5S POPC 33 -14.189 -19.570 14.206 1.00 0.00 MEMB H

ATOM 4342 C26 POPC 33 -15.604 -19.656 12.693 1.00 0.00 MEMB C

ATOM 4343 H6R POPC 33 -14.877 -20.242 12.080 1.00 0.00 MEMB H

ATOM 4344 H6S POPC 33 -15.589 -18.609 12.368 1.00 0.00 MEMB H

ATOM 4345 C27 POPC 33 -16.983 -20.133 12.346 1.00 0.00 MEMB C

ATOM 4346 H7R POPC 33 -17.881 -19.812 12.913 1.00 0.00 MEMB H

ATOM 4347 H7S POPC 33 -16.596 -21.056 12.739 1.00 0.00 MEMB H

ATOM 4348 C28 POPC 33 -17.248 -20.801 11.039 1.00 0.00 MEMB C

ATOM 4349 H8R POPC 33 -18.033 -21.598 11.101 1.00 0.00 MEMB H

ATOM 4350 H8S POPC 33 -16.257 -21.162 10.678 1.00 0.00 MEMB H

ATOM 4351 C29 POPC 33 -17.740 -19.828 10.155 1.00 0.00 MEMB C

ATOM 4352 H91 POPC 33 -18.237 -19.032 10.661 1.00 0.00 MEMB H

ATOM 4353 C210 POPC 33 -17.699 -19.951 8.867 1.00 0.00 MEMB C

ATOM 4354 H101 POPC 33 -18.001 -19.033 8.397 1.00 0.00 MEMB H

ATOM 4355 C211 POPC 33 -17.151 -21.095 8.049 1.00 0.00 MEMB C

ATOM 4356 H11R POPC 33 -17.932 -21.322 7.294 1.00 0.00 MEMB H

ATOM 4357 H11S POPC 33 -16.967 -22.042 8.607 1.00 0.00 MEMB H

ATOM 4358 C212 POPC 33 -15.819 -20.807 7.344 1.00 0.00 MEMB C

ATOM 4359 H12R POPC 33 -14.977 -20.793 8.063 1.00 0.00 MEMB H

ATOM 4360 H12S POPC 33 -15.792 -19.796 6.973 1.00 0.00 MEMB H

ATOM 4361 C213 POPC 33 -15.589 -21.762 6.158 1.00 0.00 MEMB C

ATOM 4362 H13R POPC 33 -15.811 -22.752 6.616 1.00 0.00 MEMB H

ATOM 4363 H13S POPC 33 -14.526 -21.719 5.857 1.00 0.00 MEMB H

ATOM 4364 C214 POPC 33 -16.495 -21.687 4.897 1.00 0.00 MEMB C

ATOM 4365 H14R POPC 33 -17.563 -21.690 5.224 1.00 0.00 MEMB H

ATOM 4366 H14S POPC 33 -16.360 -22.621 4.314 1.00 0.00 MEMB H

ATOM 4367 C215 POPC 33 -16.313 -20.531 3.886 1.00 0.00 MEMB C

ATOM 4368 H15R POPC 33 -16.440 -19.575 4.393 1.00 0.00 MEMB H

ATOM 4369 H15S POPC 33 -17.080 -20.602 3.080 1.00 0.00 MEMB H

ATOM 4370 C216 POPC 33 -14.987 -20.088 3.335 1.00 0.00 MEMB C

ATOM 4371 H16R POPC 33 -14.640 -20.840 2.609 1.00 0.00 MEMB H

ATOM 4372 H16S POPC 33 -14.265 -19.963 4.167 1.00 0.00 MEMB H

ATOM 4373 C217 POPC 33 -15.148 -18.668 2.755 1.00 0.00 MEMB C

ATOM 4374 H17R POPC 33 -16.220 -18.430 2.516 1.00 0.00 MEMB H

ATOM 4375 H17S POPC 33 -14.571 -18.688 1.813 1.00 0.00 MEMB H

ATOM 4376 C218 POPC 33 -14.427 -17.631 3.620 1.00 0.00 MEMB C

ATOM 4377 H18R POPC 33 -14.469 -17.905 4.678 1.00 0.00 MEMB H

ATOM 4378 H18S POPC 33 -14.835 -16.627 3.745 1.00 0.00 MEMB H

ATOM 4379 H18T POPC 33 -13.405 -17.512 3.207 1.00 0.00 MEMB H

ATOM 4380 C33 POPC 33 -9.111 -20.936 14.906 1.00 0.00 MEMB C

ATOM 4381 H3X POPC 33 -10.214 -20.903 15.012 1.00 0.00 MEMB H

ATOM 4382 H3Y POPC 33 -8.768 -21.991 15.025 1.00 0.00 MEMB H

ATOM 4383 C34 POPC 33 -8.770 -20.438 13.515 1.00 0.00 MEMB C

ATOM 4384 H4X POPC 33 -7.679 -20.362 13.551 1.00 0.00 MEMB H

ATOM 4385 H4Y POPC 33 -9.004 -19.386 13.269 1.00 0.00 MEMB H

ATOM 4386 C35 POPC 33 -9.415 -21.297 12.430 1.00 0.00 MEMB C

ATOM 4387 H5X POPC 33 -9.107 -22.371 12.468 1.00 0.00 MEMB H

ATOM 4388 H5Y POPC 33 -9.102 -20.828 11.476 1.00 0.00 MEMB H

ATOM 4389 C36 POPC 33 -10.926 -21.123 12.386 1.00 0.00 MEMB C

ATOM 4390 H6X POPC 33 -11.073 -20.023 12.286 1.00 0.00 MEMB H

ATOM 4391 H6Y POPC 33 -11.334 -21.435 13.365 1.00 0.00 MEMB H

ATOM 4392 C37 POPC 33 -11.663 -21.880 11.258 1.00 0.00 MEMB C

ATOM 4393 H7X POPC 33 -12.736 -21.589 11.330 1.00 0.00 MEMB H

ATOM 4394 H7Y POPC 33 -11.580 -22.971 11.434 1.00 0.00 MEMB H

ATOM 4395 C38 POPC 33 -11.231 -21.564 9.815 1.00 0.00 MEMB C

ATOM 4396 H8X POPC 33 -11.798 -22.197 9.100 1.00 0.00 MEMB H

ATOM 4397 H8Y POPC 33 -10.167 -21.852 9.666 1.00 0.00 MEMB H

ATOM 4398 C39 POPC 33 -11.515 -20.075 9.542 1.00 0.00 MEMB C

ATOM 4399 H9X POPC 33 -10.839 -19.491 10.211 1.00 0.00 MEMB H

ATOM 4400 H9Y POPC 33 -12.558 -19.832 9.820 1.00 0.00 MEMB H

ATOM 4401 C310 POPC 33 -11.268 -19.560 8.131 1.00 0.00 MEMB C

ATOM 4402 H10X POPC 33 -10.309 -19.779 7.799 1.00 0.00 MEMB H

ATOM 4403 H10Y POPC 33 -11.056 -18.503 8.060 1.00 0.00 MEMB H

ATOM 4404 C311 POPC 33 -12.216 -19.951 7.034 1.00 0.00 MEMB C

ATOM 4405 H11X POPC 33 -13.218 -19.635 7.376 1.00 0.00 MEMB H

ATOM 4406 H11Y POPC 33 -12.186 -21.048 6.864 1.00 0.00 MEMB H

ATOM 4407 C312 POPC 33 -11.854 -19.173 5.769 1.00 0.00 MEMB C

ATOM 4408 H12X POPC 33 -11.487 -18.156 6.019 1.00 0.00 MEMB H

ATOM 4409 H12Y POPC 33 -12.758 -19.049 5.161 1.00 0.00 MEMB H

ATOM 4410 C313 POPC 33 -10.812 -19.857 4.912 1.00 0.00 MEMB C

ATOM 4411 H13X POPC 33 -10.009 -20.216 5.559 1.00 0.00 MEMB H

ATOM 4412 H13Y POPC 33 -10.417 -19.160 4.135 1.00 0.00 MEMB H

ATOM 4413 C314 POPC 33 -11.187 -21.208 4.403 1.00 0.00 MEMB C

ATOM 4414 H14X POPC 33 -12.235 -21.146 4.073 1.00 0.00 MEMB H

ATOM 4415 H14Y POPC 33 -11.087 -21.946 5.247 1.00 0.00 MEMB H

ATOM 4416 C315 POPC 33 -10.349 -21.614 3.207 1.00 0.00 MEMB C

ATOM 4417 H15X POPC 33 -10.535 -20.995 2.321 1.00 0.00 MEMB H

ATOM 4418 H15Y POPC 33 -10.707 -22.654 3.015 1.00 0.00 MEMB H

ATOM 4419 C316 POPC 33 -8.857 -21.600 3.368 1.00 0.00 MEMB C

ATOM 4420 H16X POPC 33 -8.495 -22.323 2.626 1.00 0.00 MEMB H

ATOM 4421 H16Y POPC 33 -8.659 -21.932 4.401 1.00 0.00 MEMB H

ATOM 4422 H16Z POPC 33 -8.178 -20.815 3.076 1.00 0.00 MEMB H

ATOM 4423 N POPC 34 17.946 -19.330 20.876 1.00 0.00 MEMB N

ATOM 4424 C12 POPC 34 17.567 -17.996 20.260 1.00 0.00 MEMB C

ATOM 4425 H12A POPC 34 17.703 -17.247 21.022 1.00 0.00 MEMB H

ATOM 4426 H12B POPC 34 16.496 -18.038 20.028 1.00 0.00 MEMB H

ATOM 4427 C13 POPC 34 19.359 -19.370 21.372 1.00 0.00 MEMB C

ATOM 4428 H13A POPC 34 20.079 -19.177 20.578 1.00 0.00 MEMB H

ATOM 4429 H13B POPC 34 19.517 -18.623 22.143 1.00 0.00 MEMB H

ATOM 4430 H13C POPC 34 19.625 -20.328 21.815 1.00 0.00 MEMB H

ATOM 4431 C14 POPC 34 17.035 -19.569 22.035 1.00 0.00 MEMB C

ATOM 4432 H14A POPC 34 17.022 -20.631 22.247 1.00 0.00 MEMB H

ATOM 4433 H14B POPC 34 17.394 -19.045 22.915 1.00 0.00 MEMB H

ATOM 4434 H14C POPC 34 16.022 -19.243 21.802 1.00 0.00 MEMB H

ATOM 4435 C15 POPC 34 17.740 -20.418 19.866 1.00 0.00 MEMB C

ATOM 4436 H15A POPC 34 16.690 -20.552 19.620 1.00 0.00 MEMB H

ATOM 4437 H15B POPC 34 18.220 -21.350 20.118 1.00 0.00 MEMB H

ATOM 4438 H15C POPC 34 18.163 -20.087 18.907 1.00 0.00 MEMB H

ATOM 4439 C11 POPC 34 18.275 -17.510 18.963 1.00 0.00 MEMB C

ATOM 4440 H11A POPC 34 19.358 -17.373 19.161 1.00 0.00 MEMB H

ATOM 4441 H11B POPC 34 17.843 -16.513 18.694 1.00 0.00 MEMB H

ATOM 4442 P POPC 34 16.824 -18.521 17.044 1.00 0.00 MEMB P

ATOM 4443 O13 POPC 34 16.602 -17.256 16.322 1.00 0.00 MEMB O

ATOM 4444 O14 POPC 34 15.800 -19.036 17.981 1.00 0.00 MEMB O

ATOM 4445 O12 POPC 34 18.170 -18.418 17.876 1.00 0.00 MEMB O

ATOM 4446 O11 POPC 34 17.131 -19.692 16.039 1.00 0.00 MEMB O

ATOM 4447 C1 POPC 34 17.342 -20.981 16.628 1.00 0.00 MEMB C

ATOM 4448 HA POPC 34 16.512 -21.298 17.305 1.00 0.00 MEMB H

ATOM 4449 HB POPC 34 18.299 -20.999 17.206 1.00 0.00 MEMB H

ATOM 4450 C2 POPC 34 17.466 -22.057 15.540 1.00 0.00 MEMB C

ATOM 4451 HS POPC 34 18.022 -22.889 16.016 1.00 0.00 MEMB H

ATOM 4452 O21 POPC 34 18.156 -21.570 14.362 1.00 0.00 MEMB O

ATOM 4453 C21 POPC 34 19.322 -20.963 14.620 1.00 0.00 MEMB C

ATOM 4454 O22 POPC 34 20.015 -21.110 15.589 1.00 0.00 MEMB O

ATOM 4455 C22 POPC 34 19.771 -19.951 13.612 1.00 0.00 MEMB C

ATOM 4456 H2R POPC 34 20.682 -20.323 13.101 1.00 0.00 MEMB H

ATOM 4457 H2S POPC 34 20.034 -19.065 14.238 1.00 0.00 MEMB H

ATOM 4458 C3 POPC 34 16.061 -22.620 15.134 1.00 0.00 MEMB C

ATOM 4459 HX POPC 34 15.664 -23.135 16.032 1.00 0.00 MEMB H

ATOM 4460 HY POPC 34 16.155 -23.396 14.341 1.00 0.00 MEMB H

ATOM 4461 O31 POPC 34 15.095 -21.613 14.796 1.00 0.00 MEMB O

ATOM 4462 C31 POPC 34 15.072 -21.267 13.520 1.00 0.00 MEMB C

ATOM 4463 O32 POPC 34 15.745 -21.761 12.632 1.00 0.00 MEMB O

ATOM 4464 C32 POPC 34 13.971 -20.264 13.316 1.00 0.00 MEMB C

ATOM 4465 H2X POPC 34 13.706 -19.794 14.287 1.00 0.00 MEMB H

ATOM 4466 H2Y POPC 34 13.074 -20.800 12.937 1.00 0.00 MEMB H

ATOM 4467 C23 POPC 34 18.653 -19.647 12.642 1.00 0.00 MEMB C

ATOM 4468 H3R POPC 34 17.723 -19.416 13.220 1.00 0.00 MEMB H

ATOM 4469 H3S POPC 34 18.488 -20.569 12.037 1.00 0.00 MEMB H

ATOM 4470 C24 POPC 34 19.007 -18.476 11.743 1.00 0.00 MEMB C

ATOM 4471 H4R POPC 34 19.816 -18.791 11.051 1.00 0.00 MEMB H

ATOM 4472 H4S POPC 34 19.382 -17.618 12.349 1.00 0.00 MEMB H

ATOM 4473 C25 POPC 34 17.794 -18.037 10.936 1.00 0.00 MEMB C

ATOM 4474 H5R POPC 34 16.943 -17.894 11.643 1.00 0.00 MEMB H

ATOM 4475 H5S POPC 34 17.498 -18.852 10.240 1.00 0.00 MEMB H

ATOM 4476 C26 POPC 34 18.037 -16.718 10.197 1.00 0.00 MEMB C

ATOM 4477 H6R POPC 34 18.305 -15.946 10.949 1.00 0.00 MEMB H

ATOM 4478 H6S POPC 34 17.083 -16.424 9.708 1.00 0.00 MEMB H

ATOM 4479 C27 POPC 34 19.108 -16.755 9.103 1.00 0.00 MEMB C

ATOM 4480 H7R POPC 34 20.017 -17.313 9.416 1.00 0.00 MEMB H

ATOM 4481 H7S POPC 34 19.436 -15.713 8.892 1.00 0.00 MEMB H

ATOM 4482 C28 POPC 34 18.498 -17.296 7.809 1.00 0.00 MEMB C

ATOM 4483 H8R POPC 34 17.781 -16.533 7.459 1.00 0.00 MEMB H

ATOM 4484 H8S POPC 34 17.919 -18.226 8.024 1.00 0.00 MEMB H

ATOM 4485 C29 POPC 34 19.533 -17.502 6.750 1.00 0.00 MEMB C

ATOM 4486 H91 POPC 34 19.604 -16.714 5.988 1.00 0.00 MEMB H

ATOM 4487 C210 POPC 34 20.307 -18.590 6.685 1.00 0.00 MEMB C

ATOM 4488 H101 POPC 34 21.082 -18.654 5.902 1.00 0.00 MEMB H

ATOM 4489 C211 POPC 34 20.210 -19.726 7.680 1.00 0.00 MEMB C

ATOM 4490 H11R POPC 34 19.734 -19.421 8.635 1.00 0.00 MEMB H

ATOM 4491 H11S POPC 34 21.240 -20.026 7.962 1.00 0.00 MEMB H

ATOM 4492 C212 POPC 34 19.351 -20.926 7.198 1.00 0.00 MEMB C

ATOM 4493 H12R POPC 34 18.431 -20.631 6.657 1.00 0.00 MEMB H

ATOM 4494 H12S POPC 34 19.169 -21.629 8.043 1.00 0.00 MEMB H

ATOM 4495 C213 POPC 34 20.108 -21.703 6.170 1.00 0.00 MEMB C

ATOM 4496 H13R POPC 34 20.992 -21.721 6.752 1.00 0.00 MEMB H

ATOM 4497 H13S POPC 34 20.291 -20.995 5.340 1.00 0.00 MEMB H

ATOM 4498 C214 POPC 34 19.757 -23.195 5.928 1.00 0.00 MEMB C

ATOM 4499 H14R POPC 34 18.691 -23.146 5.766 1.00 0.00 MEMB H

ATOM 4500 H14S POPC 34 20.103 -23.826 6.794 1.00 0.00 MEMB H

ATOM 4501 C215 POPC 34 20.193 -23.929 4.649 1.00 0.00 MEMB C

ATOM 4502 H15R POPC 34 20.253 -25.035 4.658 1.00 0.00 MEMB H

ATOM 4503 H15S POPC 34 21.266 -23.863 4.945 1.00 0.00 MEMB H

ATOM 4504 C216 POPC 34 19.699 -23.252 3.233 1.00 0.00 MEMB C

ATOM 4505 H16R POPC 34 20.169 -23.613 2.284 1.00 0.00 MEMB H

ATOM 4506 H16S POPC 34 19.722 -22.230 2.865 1.00 0.00 MEMB H

ATOM 4507 C217 POPC 34 18.471 -23.555 2.787 1.00 0.00 MEMB C

ATOM 4508 H17R POPC 34 17.754 -23.110 3.491 1.00 0.00 MEMB H

ATOM 4509 H17S POPC 34 18.894 -24.552 3.111 1.00 0.00 MEMB H

ATOM 4510 C218 POPC 34 18.294 -23.320 1.260 1.00 0.00 MEMB C

ATOM 4511 H18R POPC 34 18.089 -22.264 1.087 1.00 0.00 MEMB H

ATOM 4512 H18S POPC 34 17.437 -23.864 0.845 1.00 0.00 MEMB H

ATOM 4513 H18T POPC 34 19.143 -23.635 0.613 1.00 0.00 MEMB H

ATOM 4514 C33 POPC 34 14.448 -19.203 12.313 1.00 0.00 MEMB C

ATOM 4515 H3X POPC 34 14.998 -19.643 11.452 1.00 0.00 MEMB H

ATOM 4516 H3Y POPC 34 15.146 -18.534 12.870 1.00 0.00 MEMB H

ATOM 4517 C34 POPC 34 13.229 -18.478 11.766 1.00 0.00 MEMB C

ATOM 4518 H4X POPC 34 12.670 -18.242 12.652 1.00 0.00 MEMB H

ATOM 4519 H4Y POPC 34 12.563 -19.138 11.176 1.00 0.00 MEMB H

ATOM 4520 C35 POPC 34 13.460 -17.158 11.066 1.00 0.00 MEMB C

ATOM 4521 H5X POPC 34 13.921 -16.446 11.786 1.00 0.00 MEMB H

ATOM 4522 H5Y POPC 34 12.466 -16.735 10.799 1.00 0.00 MEMB H

ATOM 4523 C36 POPC 34 14.321 -17.342 9.822 1.00 0.00 MEMB C

ATOM 4524 H6X POPC 34 15.345 -17.576 10.149 1.00 0.00 MEMB H

ATOM 4525 H6Y POPC 34 14.362 -16.348 9.318 1.00 0.00 MEMB H

ATOM 4526 C37 POPC 34 13.893 -18.507 8.875 1.00 0.00 MEMB C

ATOM 4527 H7X POPC 34 12.884 -18.360 8.438 1.00 0.00 MEMB H

ATOM 4528 H7Y POPC 34 13.713 -19.441 9.430 1.00 0.00 MEMB H

ATOM 4529 C38 POPC 34 14.914 -18.820 7.731 1.00 0.00 MEMB C

ATOM 4530 H8X POPC 34 14.385 -19.534 7.058 1.00 0.00 MEMB H

ATOM 4531 H8Y POPC 34 15.894 -19.143 8.098 1.00 0.00 MEMB H

ATOM 4532 C39 POPC 34 15.390 -17.643 6.900 1.00 0.00 MEMB C

ATOM 4533 H9X POPC 34 15.954 -16.794 7.287 1.00 0.00 MEMB H

ATOM 4534 H9Y POPC 34 14.567 -17.123 7.386 1.00 0.00 MEMB H

ATOM 4535 C310 POPC 34 15.512 -17.788 5.311 1.00 0.00 MEMB C

ATOM 4536 H10X POPC 34 16.602 -17.826 5.239 1.00 0.00 MEMB H

ATOM 4537 H10Y POPC 34 15.318 -16.938 4.617 1.00 0.00 MEMB H

ATOM 4538 C311 POPC 34 14.739 -18.783 4.409 1.00 0.00 MEMB C

ATOM 4539 H11X POPC 34 13.714 -18.634 3.995 1.00 0.00 MEMB H

ATOM 4540 H11Y POPC 34 14.698 -19.770 4.892 1.00 0.00 MEMB H

ATOM 4541 C312 POPC 34 15.445 -18.737 3.066 1.00 0.00 MEMB C

ATOM 4542 H12X POPC 34 15.072 -18.019 2.297 1.00 0.00 MEMB H

ATOM 4543 H12Y POPC 34 15.145 -19.683 2.757 1.00 0.00 MEMB H

ATOM 4544 C313 POPC 34 16.909 -19.043 3.237 1.00 0.00 MEMB C

ATOM 4545 H13X POPC 34 16.915 -19.539 4.240 1.00 0.00 MEMB H

ATOM 4546 H13Y POPC 34 17.490 -18.108 3.279 1.00 0.00 MEMB H

ATOM 4547 C314 POPC 34 17.487 -20.189 2.425 1.00 0.00 MEMB C

ATOM 4548 H14X POPC 34 16.729 -20.947 2.132 1.00 0.00 MEMB H

ATOM 4549 H14Y POPC 34 18.290 -20.684 2.992 1.00 0.00 MEMB H

ATOM 4550 C315 POPC 34 18.112 -19.755 1.181 1.00 0.00 MEMB C

ATOM 4551 H15X POPC 34 18.783 -20.496 0.690 1.00 0.00 MEMB H

ATOM 4552 H15Y POPC 34 18.734 -18.842 1.324 1.00 0.00 MEMB H

ATOM 4553 C316 POPC 34 16.966 -19.628 0.259 1.00 0.00 MEMB C

ATOM 4554 H16X POPC 34 16.460 -20.455 -0.236 1.00 0.00 MEMB H

ATOM 4555 H16Y POPC 34 17.652 -19.512 -0.562 1.00 0.00 MEMB H

ATOM 4556 H16Z POPC 34 16.295 -18.727 0.335 1.00 0.00 MEMB H

ATOM 4557 N POPC 35 -25.684 -8.962 20.715 1.00 0.00 MEMB N

ATOM 4558 C12 POPC 35 -24.333 -9.425 20.186 1.00 0.00 MEMB C

ATOM 4559 H12A POPC 35 -24.012 -10.271 20.774 1.00 0.00 MEMB H

ATOM 4560 H12B POPC 35 -23.648 -8.602 20.303 1.00 0.00 MEMB H

ATOM 4561 C13 POPC 35 -26.610 -10.141 20.883 1.00 0.00 MEMB C

ATOM 4562 H13A POPC 35 -27.480 -9.898 21.471 1.00 0.00 MEMB H

ATOM 4563 H13B POPC 35 -26.941 -10.483 19.891 1.00 0.00 MEMB H

ATOM 4564 H13C POPC 35 -26.088 -10.962 21.356 1.00 0.00 MEMB H

ATOM 4565 C14 POPC 35 -25.508 -8.319 22.052 1.00 0.00 MEMB C

ATOM 4566 H14A POPC 35 -25.404 -9.055 22.852 1.00 0.00 MEMB H

ATOM 4567 H14B POPC 35 -24.638 -7.673 22.102 1.00 0.00 MEMB H

ATOM 4568 H14C POPC 35 -26.360 -7.699 22.298 1.00 0.00 MEMB H

ATOM 4569 C15 POPC 35 -26.253 -7.956 19.737 1.00 0.00 MEMB C

ATOM 4570 H15A POPC 35 -25.509 -7.227 19.422 1.00 0.00 MEMB H

ATOM 4571 H15B POPC 35 -27.129 -7.488 20.129 1.00 0.00 MEMB H

ATOM 4572 H15C POPC 35 -26.617 -8.432 18.817 1.00 0.00 MEMB H

ATOM 4573 C11 POPC 35 -24.205 -9.825 18.705 1.00 0.00 MEMB C

ATOM 4574 H11A POPC 35 -23.244 -10.361 18.512 1.00 0.00 MEMB H

ATOM 4575 H11B POPC 35 -24.195 -8.917 18.063 1.00 0.00 MEMB H

ATOM 4576 P POPC 35 -26.248 -10.350 17.158 1.00 0.00 MEMB P

ATOM 4577 O13 POPC 35 -25.656 -9.444 16.175 1.00 0.00 MEMB O

ATOM 4578 O14 POPC 35 -27.528 -10.036 17.820 1.00 0.00 MEMB O

ATOM 4579 O12 POPC 35 -25.288 -10.647 18.388 1.00 0.00 MEMB O

ATOM 4580 O11 POPC 35 -26.275 -11.662 16.411 1.00 0.00 MEMB O

ATOM 4581 C1 POPC 35 -25.336 -12.586 16.856 1.00 0.00 MEMB C

ATOM 4582 HA POPC 35 -26.183 -13.011 17.306 1.00 0.00 MEMB H

ATOM 4583 HB POPC 35 -24.689 -12.455 17.750 1.00 0.00 MEMB H

ATOM 4584 C2 POPC 35 -24.403 -13.375 15.902 1.00 0.00 MEMB C

ATOM 4585 HS POPC 35 -24.072 -14.354 16.308 1.00 0.00 MEMB H

ATOM 4586 O21 POPC 35 -23.387 -12.502 15.827 1.00 0.00 MEMB O

ATOM 4587 C21 POPC 35 -22.343 -13.007 15.332 1.00 0.00 MEMB C

ATOM 4588 O22 POPC 35 -22.024 -14.169 15.279 1.00 0.00 MEMB O

ATOM 4589 C22 POPC 35 -21.648 -11.858 14.771 1.00 0.00 MEMB C

ATOM 4590 H2R POPC 35 -21.319 -12.090 13.741 1.00 0.00 MEMB H

ATOM 4591 H2S POPC 35 -20.805 -11.692 15.471 1.00 0.00 MEMB H

ATOM 4592 C3 POPC 35 -24.888 -13.817 14.574 1.00 0.00 MEMB C

ATOM 4593 HX POPC 35 -25.469 -14.674 14.863 1.00 0.00 MEMB H

ATOM 4594 HY POPC 35 -24.251 -14.418 13.889 1.00 0.00 MEMB H

ATOM 4595 O31 POPC 35 -25.694 -12.764 14.091 1.00 0.00 MEMB O

ATOM 4596 C31 POPC 35 -25.568 -12.524 12.855 1.00 0.00 MEMB C

ATOM 4597 O32 POPC 35 -24.839 -13.137 12.088 1.00 0.00 MEMB O

ATOM 4598 C32 POPC 35 -26.501 -11.367 12.516 1.00 0.00 MEMB C

ATOM 4599 H2X POPC 35 -25.826 -10.574 12.161 1.00 0.00 MEMB H

ATOM 4600 H2Y POPC 35 -27.052 -11.045 13.428 1.00 0.00 MEMB H

ATOM 4601 C23 POPC 35 -22.570 -10.643 14.749 1.00 0.00 MEMB C

ATOM 4602 H3R POPC 35 -23.109 -10.413 15.717 1.00 0.00 MEMB H

ATOM 4603 H3S POPC 35 -23.356 -10.659 13.960 1.00 0.00 MEMB H

ATOM 4604 C24 POPC 35 -21.660 -9.527 14.628 1.00 0.00 MEMB C

ATOM 4605 H4R POPC 35 -21.152 -9.408 15.618 1.00 0.00 MEMB H

ATOM 4606 H4S POPC 35 -22.449 -8.755 14.449 1.00 0.00 MEMB H

ATOM 4607 C25 POPC 35 -20.593 -9.535 13.551 1.00 0.00 MEMB C

ATOM 4608 H5R POPC 35 -19.831 -10.312 13.704 1.00 0.00 MEMB H

ATOM 4609 H5S POPC 35 -19.953 -8.632 13.572 1.00 0.00 MEMB H

ATOM 4610 C26 POPC 35 -21.232 -9.720 12.202 1.00 0.00 MEMB C

ATOM 4611 H6R POPC 35 -22.040 -10.473 12.122 1.00 0.00 MEMB H

ATOM 4612 H6S POPC 35 -20.398 -9.786 11.464 1.00 0.00 MEMB H

ATOM 4613 C27 POPC 35 -22.024 -8.630 11.750 1.00 0.00 MEMB C

ATOM 4614 H7R POPC 35 -21.210 -7.854 11.891 1.00 0.00 MEMB H

ATOM 4615 H7S POPC 35 -22.715 -8.045 12.394 1.00 0.00 MEMB H

ATOM 4616 C28 POPC 35 -22.537 -9.101 10.219 1.00 0.00 MEMB C

ATOM 4617 H8R POPC 35 -21.661 -9.110 9.556 1.00 0.00 MEMB H

ATOM 4618 H8S POPC 35 -22.879 -8.781 9.137 1.00 0.00 MEMB H

ATOM 4619 C29 POPC 35 -23.910 -9.219 9.809 1.00 0.00 MEMB C

ATOM 4620 H91 POPC 35 -24.662 -8.529 10.267 1.00 0.00 MEMB H

ATOM 4621 C210 POPC 35 -24.028 -9.731 8.576 1.00 0.00 MEMB C

ATOM 4622 H101 POPC 35 -25.020 -9.993 8.237 1.00 0.00 MEMB H

ATOM 4623 C211 POPC 35 -22.856 -10.039 7.640 1.00 0.00 MEMB C

ATOM 4624 H11R POPC 35 -22.394 -11.019 7.880 1.00 0.00 MEMB H

ATOM 4625 H11S POPC 35 -21.872 -9.555 7.761 1.00 0.00 MEMB H

ATOM 4626 C212 POPC 35 -23.400 -9.732 6.208 1.00 0.00 MEMB C

ATOM 4627 H12R POPC 35 -24.324 -10.315 6.146 1.00 0.00 MEMB H

ATOM 4628 H12S POPC 35 -22.820 -10.249 5.438 1.00 0.00 MEMB H

ATOM 4629 C213 POPC 35 -23.625 -8.258 5.666 1.00 0.00 MEMB C

ATOM 4630 H13R POPC 35 -22.790 -7.741 5.147 1.00 0.00 MEMB H

ATOM 4631 H13S POPC 35 -23.836 -7.539 6.477 1.00 0.00 MEMB H

ATOM 4632 C214 POPC 35 -24.543 -8.202 4.467 1.00 0.00 MEMB C

ATOM 4633 H14R POPC 35 -24.565 -7.260 3.910 1.00 0.00 MEMB H

ATOM 4634 H14S POPC 35 -25.549 -8.285 4.890 1.00 0.00 MEMB H

ATOM 4635 C215 POPC 35 -24.187 -9.191 3.349 1.00 0.00 MEMB C

ATOM 4636 H15R POPC 35 -25.021 -9.057 2.629 1.00 0.00 MEMB H

ATOM 4637 H15S POPC 35 -24.066 -10.202 3.758 1.00 0.00 MEMB H

ATOM 4638 C216 POPC 35 -22.960 -8.891 2.505 1.00 0.00 MEMB C

ATOM 4639 H16R POPC 35 -22.063 -8.895 3.087 1.00 0.00 MEMB H

ATOM 4640 H16S POPC 35 -23.087 -7.791 2.369 1.00 0.00 MEMB H

ATOM 4641 C217 POPC 35 -22.614 -9.855 1.243 1.00 0.00 MEMB C

ATOM 4642 H17R POPC 35 -23.481 -9.782 0.631 1.00 0.00 MEMB H

ATOM 4643 H17S POPC 35 -22.758 -10.884 1.562 1.00 0.00 MEMB H

ATOM 4644 C218 POPC 35 -21.259 -9.851 0.371 1.00 0.00 MEMB C

ATOM 4645 H18R POPC 35 -21.097 -10.420 -0.548 1.00 0.00 MEMB H

ATOM 4646 H18S POPC 35 -20.377 -9.885 1.055 1.00 0.00 MEMB H

ATOM 4647 H18T POPC 35 -21.133 -8.923 -0.219 1.00 0.00 MEMB H

ATOM 4648 C33 POPC 35 -27.524 -11.757 11.447 1.00 0.00 MEMB C

ATOM 4649 H3X POPC 35 -28.179 -12.504 11.929 1.00 0.00 MEMB H

ATOM 4650 H3Y POPC 35 -26.982 -12.069 10.536 1.00 0.00 MEMB H

ATOM 4651 C34 POPC 35 -28.551 -10.753 11.009 1.00 0.00 MEMB C

ATOM 4652 H4X POPC 35 -28.019 -9.946 10.461 1.00 0.00 MEMB H

ATOM 4653 H4Y POPC 35 -28.963 -10.398 11.979 1.00 0.00 MEMB H

ATOM 4654 C35 POPC 35 -29.730 -11.315 10.185 1.00 0.00 MEMB C

ATOM 4655 H5X POPC 35 -30.303 -10.439 9.806 1.00 0.00 MEMB H

ATOM 4656 H5Y POPC 35 -30.404 -11.906 10.848 1.00 0.00 MEMB H

ATOM 4657 C36 POPC 35 -29.388 -12.212 8.987 1.00 0.00 MEMB C

ATOM 4658 H6X POPC 35 -30.323 -12.439 8.424 1.00 0.00 MEMB H

ATOM 4659 H6Y POPC 35 -29.030 -13.195 9.342 1.00 0.00 MEMB H

ATOM 4660 C37 POPC 35 -28.395 -11.583 8.006 1.00 0.00 MEMB C

ATOM 4661 H7X POPC 35 -27.420 -11.383 8.500 1.00 0.00 MEMB H

ATOM 4662 H7Y POPC 35 -28.802 -10.595 7.691 1.00 0.00 MEMB H

ATOM 4663 C38 POPC 35 -28.184 -12.489 6.784 1.00 0.00 MEMB C

ATOM 4664 H8X POPC 35 -29.165 -12.663 6.286 1.00 0.00 MEMB H

ATOM 4665 H8Y POPC 35 -27.763 -13.469 7.102 1.00 0.00 MEMB H

ATOM 4666 C39 POPC 35 -27.281 -11.866 5.732 1.00 0.00 MEMB C

ATOM 4667 H9X POPC 35 -26.233 -11.838 6.104 1.00 0.00 MEMB H

ATOM 4668 H9Y POPC 35 -27.635 -10.819 5.580 1.00 0.00 MEMB H

ATOM 4669 C310 POPC 35 -27.349 -12.566 4.371 1.00 0.00 MEMB C

ATOM 4670 H10X POPC 35 -28.410 -12.645 4.037 1.00 0.00 MEMB H

ATOM 4671 H10Y POPC 35 -26.926 -13.594 4.423 1.00 0.00 MEMB H

ATOM 4672 C311 POPC 35 -26.569 -11.726 3.363 1.00 0.00 MEMB C

ATOM 4673 H11X POPC 35 -25.532 -11.627 3.752 1.00 0.00 MEMB H

ATOM 4674 H11Y POPC 35 -27.019 -10.707 3.336 1.00 0.00 MEMB H

ATOM 4675 C312 POPC 35 -26.534 -12.299 1.946 1.00 0.00 MEMB C

ATOM 4676 H12X POPC 35 -27.563 -12.565 1.618 1.00 0.00 MEMB H

ATOM 4677 H12Y POPC 35 -25.916 -13.224 1.961 1.00 0.00 MEMB H

ATOM 4678 C313 POPC 35 -25.918 -11.308 0.951 1.00 0.00 MEMB C

ATOM 4679 H13X POPC 35 -25.111 -10.783 1.492 1.00 0.00 MEMB H

ATOM 4680 H13Y POPC 35 -26.680 -10.543 0.666 1.00 0.00 MEMB H

ATOM 4681 C314 POPC 35 -25.333 -11.974 -0.311 1.00 0.00 MEMB C

ATOM 4682 H14X POPC 35 -24.693 -12.837 -0.051 1.00 0.00 MEMB H

ATOM 4683 H14Y POPC 35 -24.615 -11.299 -0.816 1.00 0.00 MEMB H

ATOM 4684 C315 POPC 35 -26.494 -12.419 -1.216 1.00 0.00 MEMB C

ATOM 4685 H15X POPC 35 -27.191 -11.570 -1.382 1.00 0.00 MEMB H

ATOM 4686 H15Y POPC 35 -27.008 -13.215 -0.632 1.00 0.00 MEMB H

ATOM 4687 C316 POPC 35 -26.183 -13.014 -2.576 1.00 0.00 MEMB C

ATOM 4688 H16X POPC 35 -27.069 -13.314 -3.153 1.00 0.00 MEMB H

ATOM 4689 H16Y POPC 35 -25.656 -13.978 -2.408 1.00 0.00 MEMB H

ATOM 4690 H16Z POPC 35 -25.567 -12.301 -3.185 1.00 0.00 MEMB H

ATOM 4691 N POPC 36 -28.912 5.244 21.185 1.00 0.00 MEMB N

ATOM 4692 C12 POPC 36 -28.276 4.050 20.487 1.00 0.00 MEMB C

ATOM 4693 H12A POPC 36 -28.808 3.151 20.778 1.00 0.00 MEMB H

ATOM 4694 H12B POPC 36 -27.263 3.954 20.849 1.00 0.00 MEMB H

ATOM 4695 C13 POPC 36 -30.338 5.405 20.767 1.00 0.00 MEMB C

ATOM 4696 H13A POPC 36 -30.799 6.264 21.236 1.00 0.00 MEMB H

ATOM 4697 H13B POPC 36 -30.346 5.554 19.682 1.00 0.00 MEMB H

ATOM 4698 H13C POPC 36 -30.928 4.521 20.920 1.00 0.00 MEMB H

ATOM 4699 C14 POPC 36 -28.878 5.025 22.655 1.00 0.00 MEMB C

ATOM 4700 H14A POPC 36 -29.245 5.901 23.175 1.00 0.00 MEMB H

ATOM 4701 H14B POPC 36 -29.518 4.191 22.931 1.00 0.00 MEMB H

ATOM 4702 H14C POPC 36 -27.867 4.822 22.988 1.00 0.00 MEMB H

ATOM 4703 C15 POPC 36 -28.240 6.540 20.832 1.00 0.00 MEMB C

ATOM 4704 H15A POPC 36 -27.201 6.531 21.082 1.00 0.00 MEMB H

ATOM 4705 H15B POPC 36 -28.679 7.361 21.393 1.00 0.00 MEMB H

ATOM 4706 H15C POPC 36 -28.385 6.748 19.765 1.00 0.00 MEMB H

ATOM 4707 C11 POPC 36 -28.150 4.022 18.958 1.00 0.00 MEMB C

ATOM 4708 H11A POPC 36 -27.726 3.031 18.671 1.00 0.00 MEMB H

ATOM 4709 H11B POPC 36 -27.463 4.831 18.615 1.00 0.00 MEMB H

ATOM 4710 P POPC 36 -29.522 4.590 16.881 1.00 0.00 MEMB P

ATOM 4711 O13 POPC 36 -28.835 5.869 16.629 1.00 0.00 MEMB O

ATOM 4712 O14 POPC 36 -30.884 4.285 16.417 1.00 0.00 MEMB O

ATOM 4713 O12 POPC 36 -29.426 4.150 18.385 1.00 0.00 MEMB O

ATOM 4714 O11 POPC 36 -28.534 3.567 16.289 1.00 0.00 MEMB O

ATOM 4715 C1 POPC 36 -29.220 2.546 15.686 1.00 0.00 MEMB C

ATOM 4716 HA POPC 36 -29.607 2.835 14.676 1.00 0.00 MEMB H

ATOM 4717 HB POPC 36 -30.126 2.135 16.253 1.00 0.00 MEMB H

ATOM 4718 C2 POPC 36 -28.199 1.501 15.665 1.00 0.00 MEMB C

ATOM 4719 HS POPC 36 -28.046 1.081 16.708 1.00 0.00 MEMB H

ATOM 4720 O21 POPC 36 -26.965 1.671 14.981 1.00 0.00 MEMB O

ATOM 4721 C21 POPC 36 -26.131 0.648 15.078 1.00 0.00 MEMB C

ATOM 4722 O22 POPC 36 -26.180 -0.274 15.873 1.00 0.00 MEMB O

ATOM 4723 C22 POPC 36 -25.026 0.977 14.133 1.00 0.00 MEMB C

ATOM 4724 H2R POPC 36 -24.215 0.242 14.284 1.00 0.00 MEMB H

ATOM 4725 H2S POPC 36 -24.715 1.997 14.436 1.00 0.00 MEMB H

ATOM 4726 C3 POPC 36 -29.006 0.671 14.960 1.00 0.00 MEMB C

ATOM 4727 HX POPC 36 -29.866 0.346 15.660 1.00 0.00 MEMB H

ATOM 4728 HY POPC 36 -28.306 -0.109 15.103 1.00 0.00 MEMB H

ATOM 4729 O31 POPC 36 -29.316 1.090 13.526 1.00 0.00 MEMB O

ATOM 4730 C31 POPC 36 -28.933 0.410 12.409 1.00 0.00 MEMB C

ATOM 4731 O32 POPC 36 -27.906 -0.156 12.285 1.00 0.00 MEMB O

ATOM 4732 C32 POPC 36 -29.980 0.014 11.390 1.00 0.00 MEMB C

ATOM 4733 H2X POPC 36 -30.444 0.983 11.264 1.00 0.00 MEMB H

ATOM 4734 H2Y POPC 36 -30.788 -0.699 11.620 1.00 0.00 MEMB H

ATOM 4735 C23 POPC 36 -25.435 1.159 12.666 1.00 0.00 MEMB C

ATOM 4736 H3R POPC 36 -26.260 1.868 12.495 1.00 0.00 MEMB H

ATOM 4737 H3S POPC 36 -25.937 0.355 12.222 1.00 0.00 MEMB H

ATOM 4738 C24 POPC 36 -24.206 1.196 11.788 1.00 0.00 MEMB C

ATOM 4739 H4R POPC 36 -24.401 0.766 10.779 1.00 0.00 MEMB H

ATOM 4740 H4S POPC 36 -23.439 0.537 12.253 1.00 0.00 MEMB H

ATOM 4741 C25 POPC 36 -23.689 2.606 11.662 1.00 0.00 MEMB C

ATOM 4742 H5R POPC 36 -22.820 2.579 10.971 1.00 0.00 MEMB H

ATOM 4743 H5S POPC 36 -23.365 2.937 12.671 1.00 0.00 MEMB H

ATOM 4744 C26 POPC 36 -24.706 3.589 11.108 1.00 0.00 MEMB C

ATOM 4745 H6R POPC 36 -24.261 4.603 11.165 1.00 0.00 MEMB H

ATOM 4746 H6S POPC 36 -25.634 3.576 11.717 1.00 0.00 MEMB H

ATOM 4747 C27 POPC 36 -25.019 3.269 9.652 1.00 0.00 MEMB C

ATOM 4748 H7R POPC 36 -25.416 2.229 9.612 1.00 0.00 MEMB H

ATOM 4749 H7S POPC 36 -24.070 3.289 9.068 1.00 0.00 MEMB H

ATOM 4750 C28 POPC 36 -26.105 4.140 9.007 1.00 0.00 MEMB C

ATOM 4751 H8R POPC 36 -27.084 3.790 9.390 1.00 0.00 MEMB H

ATOM 4752 H8S POPC 36 -26.053 3.887 7.920 1.00 0.00 MEMB H

ATOM 4753 C29 POPC 36 -26.081 5.627 9.198 1.00 0.00 MEMB C

ATOM 4754 H91 POPC 36 -25.446 6.039 10.005 1.00 0.00 MEMB H

ATOM 4755 C210 POPC 36 -26.733 6.467 8.378 1.00 0.00 MEMB C

ATOM 4756 H101 POPC 36 -26.440 7.536 8.422 1.00 0.00 MEMB H

ATOM 4757 C211 POPC 36 -27.581 6.065 7.162 1.00 0.00 MEMB C

ATOM 4758 H11R POPC 36 -28.470 6.707 6.994 1.00 0.00 MEMB H

ATOM 4759 H11S POPC 36 -27.676 4.973 6.954 1.00 0.00 MEMB H

ATOM 4760 C212 POPC 36 -26.805 6.403 5.945 1.00 0.00 MEMB C

ATOM 4761 H12R POPC 36 -25.936 5.721 6.075 1.00 0.00 MEMB H

ATOM 4762 H12S POPC 36 -26.589 7.473 6.234 1.00 0.00 MEMB H

ATOM 4763 C213 POPC 36 -27.335 6.445 4.457 1.00 0.00 MEMB C

ATOM 4764 H13R POPC 36 -26.962 7.312 3.866 1.00 0.00 MEMB H

ATOM 4765 H13S POPC 36 -28.340 6.884 4.501 1.00 0.00 MEMB H

ATOM 4766 C214 POPC 36 -27.020 5.231 3.466 1.00 0.00 MEMB C

ATOM 4767 H14R POPC 36 -27.554 5.475 2.522 1.00 0.00 MEMB H

ATOM 4768 H14S POPC 36 -27.286 4.305 3.978 1.00 0.00 MEMB H

ATOM 4769 C215 POPC 36 -25.619 4.783 2.922 1.00 0.00 MEMB C

ATOM 4770 H15R POPC 36 -24.862 4.657 3.738 1.00 0.00 MEMB H

ATOM 4771 H15S POPC 36 -25.389 5.626 2.245 1.00 0.00 MEMB H

ATOM 4772 C216 POPC 36 -25.350 3.979 1.655 1.00 0.00 MEMB C

ATOM 4773 H16R POPC 36 -24.274 3.764 1.411 1.00 0.00 MEMB H

ATOM 4774 H16S POPC 36 -25.724 4.522 0.754 1.00 0.00 MEMB H

ATOM 4775 C217 POPC 36 -25.925 2.690 1.965 1.00 0.00 MEMB C

ATOM 4776 H17R POPC 36 -27.006 2.911 2.094 1.00 0.00 MEMB H

ATOM 4777 H17S POPC 36 -25.467 2.393 2.941 1.00 0.00 MEMB H

ATOM 4778 C218 POPC 36 -25.572 1.743 0.862 1.00 0.00 MEMB C

ATOM 4779 H18R POPC 36 -25.921 0.738 1.158 1.00 0.00 MEMB H

ATOM 4780 H18S POPC 36 -24.471 1.730 0.700 1.00 0.00 MEMB H

ATOM 4781 H18T POPC 36 -26.031 2.082 -0.087 1.00 0.00 MEMB H

ATOM 4782 C33 POPC 36 -29.479 -0.964 10.303 1.00 0.00 MEMB C

ATOM 4783 H3X POPC 36 -29.221 -1.952 10.727 1.00 0.00 MEMB H

ATOM 4784 H3Y POPC 36 -28.643 -0.507 9.749 1.00 0.00 MEMB H

ATOM 4785 C34 POPC 36 -30.580 -1.156 9.370 1.00 0.00 MEMB C

ATOM 4786 H4X POPC 36 -31.394 -1.797 9.829 1.00 0.00 MEMB H

ATOM 4787 H4Y POPC 36 -30.541 -1.543 8.336 1.00 0.00 MEMB H

ATOM 4788 C35 POPC 36 -30.753 0.304 9.099 1.00 0.00 MEMB C

ATOM 4789 H5X POPC 36 -31.285 0.999 9.713 1.00 0.00 MEMB H

ATOM 4790 H5Y POPC 36 -31.689 -0.223 8.886 1.00 0.00 MEMB H

ATOM 4791 C36 POPC 36 -29.743 1.113 8.118 1.00 0.00 MEMB C

ATOM 4792 H6X POPC 36 -29.232 0.396 7.447 1.00 0.00 MEMB H

ATOM 4793 H6Y POPC 36 -29.017 1.511 8.859 1.00 0.00 MEMB H

ATOM 4794 C37 POPC 36 -30.064 2.436 7.243 1.00 0.00 MEMB C

ATOM 4795 H7X POPC 36 -30.394 3.282 7.865 1.00 0.00 MEMB H

ATOM 4796 H7Y POPC 36 -30.906 2.436 6.518 1.00 0.00 MEMB H

ATOM 4797 C38 POPC 36 -29.268 2.728 5.983 1.00 0.00 MEMB C

ATOM 4798 H8X POPC 36 -29.290 1.832 5.312 1.00 0.00 MEMB H

ATOM 4799 H8Y POPC 36 -28.239 2.918 6.337 1.00 0.00 MEMB H

ATOM 4800 C39 POPC 36 -29.780 4.001 5.233 1.00 0.00 MEMB C

ATOM 4801 H9X POPC 36 -29.052 4.149 4.432 1.00 0.00 MEMB H

ATOM 4802 H9Y POPC 36 -29.714 4.840 5.952 1.00 0.00 MEMB H

ATOM 4803 C310 POPC 36 -31.180 4.207 4.576 1.00 0.00 MEMB C

ATOM 4804 H10X POPC 36 -31.067 5.016 3.803 1.00 0.00 MEMB H

ATOM 4805 H10Y POPC 36 -31.967 4.452 5.305 1.00 0.00 MEMB H

ATOM 4806 C311 POPC 36 -31.741 3.096 3.779 1.00 0.00 MEMB C

ATOM 4807 H11X POPC 36 -32.789 3.175 3.419 1.00 0.00 MEMB H

ATOM 4808 H11Y POPC 36 -31.609 2.167 4.311 1.00 0.00 MEMB H

ATOM 4809 C312 POPC 36 -30.923 2.980 2.595 1.00 0.00 MEMB C

ATOM 4810 H12X POPC 36 -30.602 1.923 2.830 1.00 0.00 MEMB H

ATOM 4811 H12Y POPC 36 -29.865 3.281 2.670 1.00 0.00 MEMB H

ATOM 4812 C313 POPC 36 -31.832 3.333 1.331 1.00 0.00 MEMB C

ATOM 4813 H13X POPC 36 -32.900 3.088 1.441 1.00 0.00 MEMB H

ATOM 4814 H13Y POPC 36 -31.973 2.715 0.422 1.00 0.00 MEMB H

ATOM 4815 C314 POPC 36 -31.312 4.404 0.476 1.00 0.00 MEMB C

ATOM 4816 H14X POPC 36 -31.830 5.182 1.060 1.00 0.00 MEMB H

ATOM 4817 H14Y POPC 36 -31.724 4.324 -0.570 1.00 0.00 MEMB H

ATOM 4818 C315 POPC 36 -29.778 4.602 0.513 1.00 0.00 MEMB C

ATOM 4819 H15X POPC 36 -29.405 4.777 1.526 1.00 0.00 MEMB H

ATOM 4820 H15Y POPC 36 -29.697 5.462 -0.149 1.00 0.00 MEMB H

ATOM 4821 C316 POPC 36 -28.774 3.711 -0.131 1.00 0.00 MEMB C

ATOM 4822 H16X POPC 36 -27.792 4.059 -0.381 1.00 0.00 MEMB H

ATOM 4823 H16Y POPC 36 -29.322 3.308 -1.009 1.00 0.00 MEMB H

ATOM 4824 H16Z POPC 36 -28.357 2.877 0.413 1.00 0.00 MEMB H

ATOM 4825 N POPC 37 -25.373 12.480 19.240 1.00 0.00 MEMB N

ATOM 4826 C12 POPC 37 -25.934 13.301 18.072 1.00 0.00 MEMB C

ATOM 4827 H12A POPC 37 -26.931 12.952 17.802 1.00 0.00 MEMB H

ATOM 4828 H12B POPC 37 -25.306 13.121 17.211 1.00 0.00 MEMB H

ATOM 4829 C13 POPC 37 -26.101 12.766 20.513 1.00 0.00 MEMB C

ATOM 4830 H13A POPC 37 -25.554 12.421 21.384 1.00 0.00 MEMB H

ATOM 4831 H13B POPC 37 -26.203 13.855 20.552 1.00 0.00 MEMB H

ATOM 4832 H13C POPC 37 -27.099 12.348 20.528 1.00 0.00 MEMB H

ATOM 4833 C14 POPC 37 -25.423 11.022 18.898 1.00 0.00 MEMB C

ATOM 4834 H14A POPC 37 -24.771 10.826 18.050 1.00 0.00 MEMB H

ATOM 4835 H14B POPC 37 -25.084 10.435 19.735 1.00 0.00 MEMB H

ATOM 4836 H14C POPC 37 -26.432 10.713 18.634 1.00 0.00 MEMB H

ATOM 4837 C15 POPC 37 -23.954 12.904 19.450 1.00 0.00 MEMB C

ATOM 4838 H15A POPC 37 -23.897 13.997 19.481 1.00 0.00 MEMB H

ATOM 4839 H15B POPC 37 -23.301 12.619 18.632 1.00 0.00 MEMB H

ATOM 4840 H15C POPC 37 -23.540 12.509 20.369 1.00 0.00 MEMB H

ATOM 4841 C11 POPC 37 -26.043 14.849 18.209 1.00 0.00 MEMB C

ATOM 4842 H11A POPC 37 -26.290 15.276 17.203 1.00 0.00 MEMB H

ATOM 4843 H11B POPC 37 -25.048 15.291 18.446 1.00 0.00 MEMB H

ATOM 4844 P POPC 37 -28.548 15.413 18.823 1.00 0.00 MEMB P

ATOM 4845 O13 POPC 37 -28.790 16.591 17.979 1.00 0.00 MEMB O

ATOM 4846 O14 POPC 37 -29.386 15.222 20.035 1.00 0.00 MEMB O

ATOM 4847 O12 POPC 37 -27.001 15.230 19.209 1.00 0.00 MEMB O

ATOM 4848 O11 POPC 37 -28.771 14.104 17.939 1.00 0.00 MEMB O

ATOM 4849 C1 POPC 37 -29.383 13.023 18.650 1.00 0.00 MEMB C

ATOM 4850 HA POPC 37 -30.398 13.306 19.016 1.00 0.00 MEMB H

ATOM 4851 HB POPC 37 -28.747 12.700 19.506 1.00 0.00 MEMB H

ATOM 4852 C2 POPC 37 -29.581 11.768 17.807 1.00 0.00 MEMB C

ATOM 4853 HS POPC 37 -29.930 10.972 18.518 1.00 0.00 MEMB H

ATOM 4854 O21 POPC 37 -28.348 11.427 17.135 1.00 0.00 MEMB O

ATOM 4855 C21 POPC 37 -28.404 10.165 16.755 1.00 0.00 MEMB C

ATOM 4856 O22 POPC 37 -29.033 9.294 17.326 1.00 0.00 MEMB O

ATOM 4857 C22 POPC 37 -27.516 9.947 15.555 1.00 0.00 MEMB C

ATOM 4858 H2R POPC 37 -26.700 9.259 15.848 1.00 0.00 MEMB H

ATOM 4859 H2S POPC 37 -27.089 10.909 15.207 1.00 0.00 MEMB H

ATOM 4860 C3 POPC 37 -30.721 11.917 16.771 1.00 0.00 MEMB C

ATOM 4861 HX POPC 37 -31.680 12.126 17.308 1.00 0.00 MEMB H

ATOM 4862 HY POPC 37 -30.722 10.950 16.266 1.00 0.00 MEMB H

ATOM 4863 O31 POPC 37 -30.449 12.794 15.702 1.00 0.00 MEMB O

ATOM 4864 C31 POPC 37 -31.438 13.515 15.235 1.00 0.00 MEMB C

ATOM 4865 O32 POPC 37 -32.592 13.536 15.636 1.00 0.00 MEMB O

ATOM 4866 C32 POPC 37 -30.859 14.345 14.134 1.00 0.00 MEMB C

ATOM 4867 H2X POPC 37 -31.290 14.012 13.169 1.00 0.00 MEMB H

ATOM 4868 H2Y POPC 37 -29.756 14.190 14.116 1.00 0.00 MEMB H

ATOM 4869 C23 POPC 37 -28.342 9.314 14.434 1.00 0.00 MEMB C

ATOM 4870 H3R POPC 37 -29.195 8.728 14.837 1.00 0.00 MEMB H

ATOM 4871 H3S POPC 37 -27.719 8.546 13.932 1.00 0.00 MEMB H

ATOM 4872 C24 POPC 37 -28.822 10.341 13.397 1.00 0.00 MEMB C

ATOM 4873 H4R POPC 37 -27.944 10.688 12.812 1.00 0.00 MEMB H

ATOM 4874 H4S POPC 37 -29.267 11.233 13.899 1.00 0.00 MEMB H

ATOM 4875 C25 POPC 37 -29.854 9.663 12.490 1.00 0.00 MEMB C

ATOM 4876 H5R POPC 37 -30.737 9.507 13.080 1.00 0.00 MEMB H

ATOM 4877 H5S POPC 37 -29.560 8.631 12.228 1.00 0.00 MEMB H

ATOM 4878 C26 POPC 37 -30.570 10.456 11.411 1.00 0.00 MEMB C

ATOM 4879 H6R POPC 37 -31.147 11.283 11.894 1.00 0.00 MEMB H

ATOM 4880 H6S POPC 37 -31.317 9.776 10.931 1.00 0.00 MEMB H

ATOM 4881 C27 POPC 37 -29.678 11.152 10.421 1.00 0.00 MEMB C

ATOM 4882 H7R POPC 37 -29.019 11.844 10.987 1.00 0.00 MEMB H

ATOM 4883 H7S POPC 37 -30.386 11.764 9.810 1.00 0.00 MEMB H

ATOM 4884 C28 POPC 37 -28.914 10.186 9.500 1.00 0.00 MEMB C

ATOM 4885 H8R POPC 37 -28.826 10.723 8.531 1.00 0.00 MEMB H

ATOM 4886 H8S POPC 37 -29.588 9.319 9.312 1.00 0.00 MEMB H

ATOM 4887 C29 POPC 37 -27.561 9.654 9.930 1.00 0.00 MEMB C

ATOM 4888 H91 POPC 37 -27.607 8.680 10.448 1.00 0.00 MEMB H

ATOM 4889 C210 POPC 37 -26.357 10.255 9.780 1.00 0.00 MEMB C

ATOM 4890 H101 POPC 37 -25.451 9.688 10.056 1.00 0.00 MEMB H

ATOM 4891 C211 POPC 37 -26.110 11.612 9.147 1.00 0.00 MEMB C

ATOM 4892 H11R POPC 37 -26.158 12.376 9.950 1.00 0.00 MEMB H

ATOM 4893 H11S POPC 37 -26.916 11.838 8.414 1.00 0.00 MEMB H

ATOM 4894 C212 POPC 37 -24.778 11.899 8.421 1.00 0.00 MEMB C

ATOM 4895 H12R POPC 37 -23.912 11.919 9.126 1.00 0.00 MEMB H

ATOM 4896 H12S POPC 37 -24.810 12.847 7.848 1.00 0.00 MEMB H

ATOM 4897 C213 POPC 37 -24.490 10.849 7.400 1.00 0.00 MEMB C

ATOM 4898 H13R POPC 37 -25.443 10.486 6.954 1.00 0.00 MEMB H

ATOM 4899 H13S POPC 37 -24.121 10.104 8.129 1.00 0.00 MEMB H

ATOM 4900 C214 POPC 37 -23.500 11.310 6.310 1.00 0.00 MEMB C

ATOM 4901 H14R POPC 37 -22.518 11.561 6.764 1.00 0.00 MEMB H

ATOM 4902 H14S POPC 37 -23.956 12.187 5.814 1.00 0.00 MEMB H

ATOM 4903 C215 POPC 37 -23.129 10.476 5.094 1.00 0.00 MEMB C

ATOM 4904 H15R POPC 37 -22.455 9.615 5.372 1.00 0.00 MEMB H

ATOM 4905 H15S POPC 37 -22.465 11.086 4.508 1.00 0.00 MEMB H

ATOM 4906 C216 POPC 37 -24.223 10.081 4.082 1.00 0.00 MEMB C

ATOM 4907 H16R POPC 37 -25.085 9.563 4.501 1.00 0.00 MEMB H

ATOM 4908 H16S POPC 37 -23.676 9.499 3.315 1.00 0.00 MEMB H

ATOM 4909 C217 POPC 37 -25.296 10.910 3.501 1.00 0.00 MEMB C

ATOM 4910 H17R POPC 37 -25.574 11.607 4.309 1.00 0.00 MEMB H

ATOM 4911 H17S POPC 37 -26.190 10.294 3.168 1.00 0.00 MEMB H

ATOM 4912 C218 POPC 37 -24.607 11.367 2.296 1.00 0.00 MEMB C

ATOM 4913 H18R POPC 37 -25.179 12.132 1.776 1.00 0.00 MEMB H

ATOM 4914 H18S POPC 37 -24.463 10.462 1.640 1.00 0.00 MEMB H

ATOM 4915 H18T POPC 37 -23.606 11.730 2.648 1.00 0.00 MEMB H

ATOM 4916 C33 POPC 37 -31.111 15.826 14.392 1.00 0.00 MEMB C

ATOM 4917 H3X POPC 37 -30.865 16.092 15.450 1.00 0.00 MEMB H

ATOM 4918 H3Y POPC 37 -32.193 16.046 14.246 1.00 0.00 MEMB H

ATOM 4919 C34 POPC 37 -30.215 16.691 13.498 1.00 0.00 MEMB C

ATOM 4920 H4X POPC 37 -29.164 16.588 13.841 1.00 0.00 MEMB H

ATOM 4921 H4Y POPC 37 -30.448 17.774 13.577 1.00 0.00 MEMB H

ATOM 4922 C35 POPC 37 -30.312 16.283 12.033 1.00 0.00 MEMB C

ATOM 4923 H5X POPC 37 -31.362 16.421 11.724 1.00 0.00 MEMB H

ATOM 4924 H5Y POPC 37 -30.076 15.214 11.889 1.00 0.00 MEMB H

ATOM 4925 C36 POPC 37 -29.344 17.083 11.175 1.00 0.00 MEMB C

ATOM 4926 H6X POPC 37 -28.325 17.043 11.600 1.00 0.00 MEMB H

ATOM 4927 H6Y POPC 37 -29.640 18.152 11.304 1.00 0.00 MEMB H

ATOM 4928 C37 POPC 37 -29.334 16.618 9.693 1.00 0.00 MEMB C

ATOM 4929 H7X POPC 37 -28.831 17.411 9.098 1.00 0.00 MEMB H

ATOM 4930 H7Y POPC 37 -30.378 16.517 9.309 1.00 0.00 MEMB H

ATOM 4931 C38 POPC 37 -28.684 15.256 9.431 1.00 0.00 MEMB C

ATOM 4932 H8X POPC 37 -29.403 14.467 9.742 1.00 0.00 MEMB H

ATOM 4933 H8Y POPC 37 -27.794 15.187 10.095 1.00 0.00 MEMB H

ATOM 4934 C39 POPC 37 -28.247 15.048 7.961 1.00 0.00 MEMB C

ATOM 4935 H9X POPC 37 -27.395 14.331 7.934 1.00 0.00 MEMB H

ATOM 4936 H9Y POPC 37 -27.877 16.020 7.568 1.00 0.00 MEMB H

ATOM 4937 C310 POPC 37 -29.349 14.555 7.001 1.00 0.00 MEMB C

ATOM 4938 H10X POPC 37 -30.237 15.204 7.163 1.00 0.00 MEMB H

ATOM 4939 H10Y POPC 37 -29.635 13.501 7.222 1.00 0.00 MEMB H

ATOM 4940 C311 POPC 37 -28.968 14.665 5.517 1.00 0.00 MEMB C

ATOM 4941 H11X POPC 37 -27.899 14.407 5.347 1.00 0.00 MEMB H

ATOM 4942 H11Y POPC 37 -29.110 15.720 5.188 1.00 0.00 MEMB H

ATOM 4943 C312 POPC 37 -29.817 13.808 4.581 1.00 0.00 MEMB C

ATOM 4944 H12X POPC 37 -30.848 14.218 4.562 1.00 0.00 MEMB H

ATOM 4945 H12Y POPC 37 -29.878 12.740 4.886 1.00 0.00 MEMB H

ATOM 4946 C313 POPC 37 -29.243 13.892 3.181 1.00 0.00 MEMB C

ATOM 4947 H13X POPC 37 -28.177 13.555 3.232 1.00 0.00 MEMB H

ATOM 4948 H13Y POPC 37 -29.220 14.956 2.860 1.00 0.00 MEMB H

ATOM 4949 C314 POPC 37 -30.011 13.054 2.142 1.00 0.00 MEMB C

ATOM 4950 H14X POPC 37 -29.982 11.984 2.424 1.00 0.00 MEMB H

ATOM 4951 H14Y POPC 37 -29.223 13.143 1.360 1.00 0.00 MEMB H

ATOM 4952 C315 POPC 37 -31.451 13.640 1.743 1.00 0.00 MEMB C

ATOM 4953 H15X POPC 37 -31.220 14.629 1.317 1.00 0.00 MEMB H

ATOM 4954 H15Y POPC 37 -32.033 13.884 2.630 1.00 0.00 MEMB H

ATOM 4955 C316 POPC 37 -32.512 13.095 0.718 1.00 0.00 MEMB C

ATOM 4956 H16X POPC 37 -32.651 12.027 0.616 1.00 0.00 MEMB H

ATOM 4957 H16Y POPC 37 -32.220 13.361 -0.296 1.00 0.00 MEMB H

ATOM 4958 H16Z POPC 37 -33.554 13.418 0.822 1.00 0.00 MEMB H

ATOM 4959 N POPC 38 -8.005 30.647 19.111 1.00 0.00 MEMB N

ATOM 4960 C12 POPC 38 -8.677 29.730 20.108 1.00 0.00 MEMB C

ATOM 4961 H12A POPC 38 -8.642 30.237 21.065 1.00 0.00 MEMB H

ATOM 4962 H12B POPC 38 -8.109 28.807 20.233 1.00 0.00 MEMB H

ATOM 4963 C13 POPC 38 -8.606 32.019 19.150 1.00 0.00 MEMB C

ATOM 4964 H13A POPC 38 -8.656 32.418 20.154 1.00 0.00 MEMB H

ATOM 4965 H13B POPC 38 -8.011 32.727 18.572 1.00 0.00 MEMB H

ATOM 4966 H13C POPC 38 -9.602 31.968 18.720 1.00 0.00 MEMB H

ATOM 4967 C14 POPC 38 -6.595 30.729 19.502 1.00 0.00 MEMB C

ATOM 4968 H14A POPC 38 -6.446 30.713 20.575 1.00 0.00 MEMB H

ATOM 4969 H14B POPC 38 -6.057 29.862 19.117 1.00 0.00 MEMB H

ATOM 4970 H14C POPC 38 -6.150 31.633 19.098 1.00 0.00 MEMB H

ATOM 4971 C15 POPC 38 -8.092 30.171 17.687 1.00 0.00 MEMB C

ATOM 4972 H15A POPC 38 -7.892 29.102 17.547 1.00 0.00 MEMB H

ATOM 4973 H15B POPC 38 -7.430 30.739 17.051 1.00 0.00 MEMB H

ATOM 4974 H15C POPC 38 -9.111 30.293 17.326 1.00 0.00 MEMB H

ATOM 4975 C11 POPC 38 -10.144 29.368 19.836 1.00 0.00 MEMB C

ATOM 4976 H11A POPC 38 -10.666 30.335 19.715 1.00 0.00 MEMB H

ATOM 4977 H11B POPC 38 -10.600 28.869 20.720 1.00 0.00 MEMB H

ATOM 4978 P POPC 38 -9.927 27.038 18.685 1.00 0.00 MEMB P

ATOM 4979 O13 POPC 38 -11.065 26.185 18.247 1.00 0.00 MEMB O

ATOM 4980 O14 POPC 38 -9.312 26.706 19.984 1.00 0.00 MEMB O

ATOM 4981 O12 POPC 38 -10.320 28.583 18.660 1.00 0.00 MEMB O

ATOM 4982 O11 POPC 38 -8.750 27.045 17.647 1.00 0.00 MEMB O

ATOM 4983 C1 POPC 38 -8.576 26.109 16.596 1.00 0.00 MEMB C

ATOM 4984 HA POPC 38 -9.159 26.421 15.698 1.00 0.00 MEMB H

ATOM 4985 HB POPC 38 -8.882 25.070 16.867 1.00 0.00 MEMB H

ATOM 4986 C2 POPC 38 -7.113 26.085 16.127 1.00 0.00 MEMB C

ATOM 4987 HS POPC 38 -6.458 25.646 16.920 1.00 0.00 MEMB H

ATOM 4988 O21 POPC 38 -6.724 27.443 15.742 1.00 0.00 MEMB O

ATOM 4989 C21 POPC 38 -5.433 27.655 15.731 1.00 0.00 MEMB C

ATOM 4990 O22 POPC 38 -4.609 27.024 16.358 1.00 0.00 MEMB O

ATOM 4991 C22 POPC 38 -5.058 28.945 14.999 1.00 0.00 MEMB C

ATOM 4992 H2R POPC 38 -4.068 29.187 15.381 1.00 0.00 MEMB H

ATOM 4993 H2S POPC 38 -5.654 29.731 15.460 1.00 0.00 MEMB H

ATOM 4994 C3 POPC 38 -7.125 25.162 14.897 1.00 0.00 MEMB C

ATOM 4995 HX POPC 38 -7.736 24.242 15.026 1.00 0.00 MEMB H

ATOM 4996 HY POPC 38 -6.118 24.702 14.818 1.00 0.00 MEMB H

ATOM 4997 O31 POPC 38 -7.582 26.016 13.792 1.00 0.00 MEMB O

ATOM 4998 C31 POPC 38 -7.712 25.402 12.647 1.00 0.00 MEMB C

ATOM 4999 O32 POPC 38 -7.549 24.222 12.493 1.00 0.00 MEMB O

ATOM 5000 C32 POPC 38 -8.280 26.237 11.496 1.00 0.00 MEMB C

ATOM 5001 H2X POPC 38 -8.578 27.238 11.828 1.00 0.00 MEMB H

ATOM 5002 H2Y POPC 38 -9.210 25.655 11.393 1.00 0.00 MEMB H

ATOM 5003 C23 POPC 38 -5.106 28.986 13.441 1.00 0.00 MEMB C

ATOM 5004 H3R POPC 38 -4.664 28.105 12.938 1.00 0.00 MEMB H

ATOM 5005 H3S POPC 38 -4.468 29.801 13.036 1.00 0.00 MEMB H

ATOM 5006 C24 POPC 38 -6.532 29.220 12.934 1.00 0.00 MEMB C

ATOM 5007 H4R POPC 38 -7.104 28.289 13.136 1.00 0.00 MEMB H

ATOM 5008 H4S POPC 38 -6.488 29.366 11.833 1.00 0.00 MEMB H

ATOM 5009 C25 POPC 38 -7.292 30.378 13.613 1.00 0.00 MEMB C

ATOM 5010 H5R POPC 38 -6.788 31.348 13.404 1.00 0.00 MEMB H

ATOM 5011 H5S POPC 38 -7.316 30.242 14.712 1.00 0.00 MEMB H

ATOM 5012 C26 POPC 38 -8.767 30.441 13.203 1.00 0.00 MEMB C

ATOM 5013 H6R POPC 38 -9.345 30.955 14.009 1.00 0.00 MEMB H

ATOM 5014 H6S POPC 38 -9.173 29.408 13.126 1.00 0.00 MEMB H

ATOM 5015 C27 POPC 38 -8.899 31.203 11.882 1.00 0.00 MEMB C

ATOM 5016 H7R POPC 38 -8.234 30.730 11.133 1.00 0.00 MEMB H

ATOM 5017 H7S POPC 38 -8.545 32.244 12.069 1.00 0.00 MEMB H

ATOM 5018 C28 POPC 38 -10.316 31.272 11.323 1.00 0.00 MEMB C

ATOM 5019 H8R POPC 38 -10.417 32.161 10.655 1.00 0.00 MEMB H

ATOM 5020 H8S POPC 38 -10.936 31.456 12.194 1.00 0.00 MEMB H

ATOM 5021 C29 POPC 38 -10.753 29.987 10.685 1.00 0.00 MEMB C

ATOM 5022 H91 POPC 38 -10.614 29.061 11.251 1.00 0.00 MEMB H

ATOM 5023 C210 POPC 38 -11.245 29.879 9.465 1.00 0.00 MEMB C

ATOM 5024 H101 POPC 38 -11.603 28.905 9.095 1.00 0.00 MEMB H

ATOM 5025 C211 POPC 38 -11.460 30.993 8.521 1.00 0.00 MEMB C

ATOM 5026 H11R POPC 38 -11.688 32.002 8.950 1.00 0.00 MEMB H

ATOM 5027 H11S POPC 38 -12.382 30.563 8.163 1.00 0.00 MEMB H

ATOM 5028 C212 POPC 38 -10.549 31.105 7.294 1.00 0.00 MEMB C

ATOM 5029 H12R POPC 38 -9.538 31.348 7.666 1.00 0.00 MEMB H

ATOM 5030 H12S POPC 38 -10.892 31.943 6.638 1.00 0.00 MEMB H

ATOM 5031 C213 POPC 38 -10.510 29.820 6.458 1.00 0.00 MEMB C

ATOM 5032 H13R POPC 38 -11.370 29.158 6.720 1.00 0.00 MEMB H

ATOM 5033 H13S POPC 38 -9.578 29.282 6.729 1.00 0.00 MEMB H

ATOM 5034 C214 POPC 38 -10.545 30.053 4.942 1.00 0.00 MEMB C

ATOM 5035 H14R POPC 38 -9.759 30.775 4.633 1.00 0.00 MEMB H

ATOM 5036 H14S POPC 38 -11.530 30.502 4.692 1.00 0.00 MEMB H

ATOM 5037 C215 POPC 38 -10.372 28.720 4.195 1.00 0.00 MEMB C

ATOM 5038 H15R POPC 38 -11.201 28.048 4.512 1.00 0.00 MEMB H

ATOM 5039 H15S POPC 38 -9.397 28.270 4.413 1.00 0.00 MEMB H

ATOM 5040 C216 POPC 38 -10.481 28.788 2.693 1.00 0.00 MEMB C

ATOM 5041 H16R POPC 38 -11.459 29.267 2.591 1.00 0.00 MEMB H

ATOM 5042 H16S POPC 38 -10.551 27.813 2.173 1.00 0.00 MEMB H

ATOM 5043 C217 POPC 38 -9.378 29.518 1.982 1.00 0.00 MEMB C

ATOM 5044 H17R POPC 38 -8.484 28.896 1.778 1.00 0.00 MEMB H

ATOM 5045 H17S POPC 38 -9.057 30.410 2.579 1.00 0.00 MEMB H

ATOM 5046 C218 POPC 38 -9.963 29.913 0.672 1.00 0.00 MEMB C

ATOM 5047 H18R POPC 38 -9.400 30.378 -0.129 1.00 0.00 MEMB H

ATOM 5048 H18S POPC 38 -10.463 30.903 0.844 1.00 0.00 MEMB H

ATOM 5049 H18T POPC 38 -10.231 29.179 -0.097 1.00 0.00 MEMB H

ATOM 5050 C33 POPC 38 -7.573 26.188 10.091 1.00 0.00 MEMB C

ATOM 5051 H3X POPC 38 -7.642 25.197 9.594 1.00 0.00 MEMB H

ATOM 5052 H3Y POPC 38 -6.509 26.432 10.144 1.00 0.00 MEMB H

ATOM 5053 C34 POPC 38 -7.937 27.335 9.157 1.00 0.00 MEMB C

ATOM 5054 H4X POPC 38 -7.867 28.246 9.801 1.00 0.00 MEMB H

ATOM 5055 H4Y POPC 38 -8.962 27.184 8.784 1.00 0.00 MEMB H

ATOM 5056 C35 POPC 38 -6.943 27.545 8.001 1.00 0.00 MEMB C

ATOM 5057 H5X POPC 38 -5.940 27.674 8.465 1.00 0.00 MEMB H

ATOM 5058 H5Y POPC 38 -7.116 28.526 7.518 1.00 0.00 MEMB H

ATOM 5059 C36 POPC 38 -6.946 26.446 6.909 1.00 0.00 MEMB C

ATOM 5060 H6X POPC 38 -6.651 25.444 7.290 1.00 0.00 MEMB H

ATOM 5061 H6Y POPC 38 -6.206 26.719 6.124 1.00 0.00 MEMB H

ATOM 5062 C37 POPC 38 -8.075 26.458 5.933 1.00 0.00 MEMB C

ATOM 5063 H7X POPC 38 -8.068 27.486 5.507 1.00 0.00 MEMB H

ATOM 5064 H7Y POPC 38 -8.999 26.281 6.507 1.00 0.00 MEMB H

ATOM 5065 C38 POPC 38 -7.870 25.433 4.809 1.00 0.00 MEMB C

ATOM 5066 H8X POPC 38 -8.729 25.527 4.110 1.00 0.00 MEMB H

ATOM 5067 H8Y POPC 38 -7.859 24.407 5.237 1.00 0.00 MEMB H

ATOM 5068 C39 POPC 38 -6.630 25.599 3.928 1.00 0.00 MEMB C

ATOM 5069 H9X POPC 38 -5.709 25.189 4.396 1.00 0.00 MEMB H

ATOM 5070 H9Y POPC 38 -6.445 26.669 3.699 1.00 0.00 MEMB H

ATOM 5071 C310 POPC 38 -6.858 24.874 2.618 1.00 0.00 MEMB C

ATOM 5072 H10X POPC 38 -7.882 24.893 2.219 1.00 0.00 MEMB H

ATOM 5073 H10Y POPC 38 -6.738 23.796 2.828 1.00 0.00 MEMB H

ATOM 5074 C311 POPC 38 -5.870 25.310 1.558 1.00 0.00 MEMB C

ATOM 5075 H11X POPC 38 -6.002 24.659 0.661 1.00 0.00 MEMB H

ATOM 5076 H11Y POPC 38 -4.834 25.312 2.000 1.00 0.00 MEMB H

ATOM 5077 C312 POPC 38 -5.722 26.657 1.050 1.00 0.00 MEMB C

ATOM 5078 H12X POPC 38 -5.301 27.249 1.878 1.00 0.00 MEMB H

ATOM 5079 H12Y POPC 38 -6.730 27.019 0.819 1.00 0.00 MEMB H

ATOM 5080 C313 POPC 38 -4.706 26.616 -0.104 1.00 0.00 MEMB C

ATOM 5081 H13X POPC 38 -5.082 26.068 -0.991 1.00 0.00 MEMB H

ATOM 5082 H13Y POPC 38 -3.753 26.092 0.146 1.00 0.00 MEMB H

ATOM 5083 C314 POPC 38 -4.356 28.048 -0.368 1.00 0.00 MEMB C

ATOM 5084 H14X POPC 38 -3.649 28.142 -1.220 1.00 0.00 MEMB H

ATOM 5085 H14Y POPC 38 -3.930 28.605 0.490 1.00 0.00 MEMB H

ATOM 5086 C315 POPC 38 -5.585 28.873 -0.475 1.00 0.00 MEMB C

ATOM 5087 H15X POPC 38 -5.094 29.723 -0.981 1.00 0.00 MEMB H

ATOM 5088 H15Y POPC 38 -6.145 29.118 0.446 1.00 0.00 MEMB H

ATOM 5089 C316 POPC 38 -6.506 28.418 -1.471 1.00 0.00 MEMB C

ATOM 5090 H16X POPC 38 -5.678 28.313 -2.162 1.00 0.00 MEMB H

ATOM 5091 H16Y POPC 38 -7.195 29.214 -1.835 1.00 0.00 MEMB H

ATOM 5092 H16Z POPC 38 -6.967 27.415 -1.347 1.00 0.00 MEMB H

ATOM 5093 N POPC 39 -8.026 -25.912 19.230 1.00 0.00 MEMB N

ATOM 5094 C12 POPC 39 -7.956 -26.897 18.068 1.00 0.00 MEMB C

ATOM 5095 H12A POPC 39 -7.159 -26.573 17.395 1.00 0.00 MEMB H

ATOM 5096 H12B POPC 39 -8.878 -26.859 17.505 1.00 0.00 MEMB H

ATOM 5097 C13 POPC 39 -6.671 -25.805 19.874 1.00 0.00 MEMB C

ATOM 5098 H13A POPC 39 -5.862 -25.869 19.149 1.00 0.00 MEMB H

ATOM 5099 H13B POPC 39 -6.532 -24.865 20.389 1.00 0.00 MEMB H

ATOM 5100 H13C POPC 39 -6.506 -26.635 20.558 1.00 0.00 MEMB H

ATOM 5101 C14 POPC 39 -8.350 -24.556 18.727 1.00 0.00 MEMB C

ATOM 5102 H14A POPC 39 -8.188 -23.837 19.520 1.00 0.00 MEMB H

ATOM 5103 H14B POPC 39 -7.712 -24.277 17.894 1.00 0.00 MEMB H

ATOM 5104 H14C POPC 39 -9.383 -24.492 18.427 1.00 0.00 MEMB H

ATOM 5105 C15 POPC 39 -9.040 -26.339 20.253 1.00 0.00 MEMB C

ATOM 5106 H15A POPC 39 -10.060 -26.149 19.924 1.00 0.00 MEMB H

ATOM 5107 H15B POPC 39 -8.900 -25.815 21.187 1.00 0.00 MEMB H

ATOM 5108 H15C POPC 39 -8.903 -27.403 20.463 1.00 0.00 MEMB H

ATOM 5109 C11 POPC 39 -7.734 -28.402 18.372 1.00 0.00 MEMB C

ATOM 5110 H11A POPC 39 -7.680 -28.904 17.373 1.00 0.00 MEMB H

ATOM 5111 H11B POPC 39 -8.630 -28.811 18.881 1.00 0.00 MEMB H

ATOM 5112 P POPC 39 -5.657 -29.887 18.802 1.00 0.00 MEMB P

ATOM 5113 O13 POPC 39 -6.264 -31.196 19.160 1.00 0.00 MEMB O

ATOM 5114 O14 POPC 39 -4.292 -29.622 19.291 1.00 0.00 MEMB O

ATOM 5115 O12 POPC 39 -6.585 -28.651 19.184 1.00 0.00 MEMB O

ATOM 5116 O11 POPC 39 -5.588 -29.819 17.232 1.00 0.00 MEMB O

ATOM 5117 C1 POPC 39 -4.786 -28.766 16.704 1.00 0.00 MEMB C

ATOM 5118 HA POPC 39 -3.733 -28.844 17.074 1.00 0.00 MEMB H

ATOM 5119 HB POPC 39 -5.165 -27.766 17.018 1.00 0.00 MEMB H

ATOM 5120 C2 POPC 39 -4.763 -28.895 15.164 1.00 0.00 MEMB C

ATOM 5121 HS POPC 39 -4.026 -28.149 14.829 1.00 0.00 MEMB H

ATOM 5122 O21 POPC 39 -6.050 -28.662 14.567 1.00 0.00 MEMB O

ATOM 5123 C21 POPC 39 -6.576 -27.474 14.763 1.00 0.00 MEMB C

ATOM 5124 O22 POPC 39 -6.038 -26.474 15.180 1.00 0.00 MEMB O

ATOM 5125 C22 POPC 39 -8.066 -27.492 14.541 1.00 0.00 MEMB C

ATOM 5126 H2R POPC 39 -8.390 -26.429 14.579 1.00 0.00 MEMB H

ATOM 5127 H2S POPC 39 -8.510 -28.028 15.401 1.00 0.00 MEMB H

ATOM 5128 C3 POPC 39 -4.249 -30.302 14.742 1.00 0.00 MEMB C

ATOM 5129 HX POPC 39 -4.997 -31.050 15.081 1.00 0.00 MEMB H

ATOM 5130 HY POPC 39 -3.333 -30.481 15.345 1.00 0.00 MEMB H

ATOM 5131 O31 POPC 39 -4.034 -30.482 13.316 1.00 0.00 MEMB O

ATOM 5132 C31 POPC 39 -2.906 -29.959 12.821 1.00 0.00 MEMB C

ATOM 5133 O32 POPC 39 -2.066 -29.393 13.499 1.00 0.00 MEMB O

ATOM 5134 C32 POPC 39 -2.738 -30.113 11.297 1.00 0.00 MEMB C

ATOM 5135 H2X POPC 39 -1.937 -30.837 11.099 1.00 0.00 MEMB H

ATOM 5136 H2Y POPC 39 -2.270 -29.213 10.875 1.00 0.00 MEMB H

ATOM 5137 C23 POPC 39 -8.448 -28.137 13.203 1.00 0.00 MEMB C

ATOM 5138 H3R POPC 39 -8.469 -29.239 13.372 1.00 0.00 MEMB H

ATOM 5139 H3S POPC 39 -7.689 -27.924 12.417 1.00 0.00 MEMB H

ATOM 5140 C24 POPC 39 -9.822 -27.659 12.726 1.00 0.00 MEMB C

ATOM 5141 H4R POPC 39 -10.461 -27.543 13.626 1.00 0.00 MEMB H

ATOM 5142 H4S POPC 39 -10.268 -28.450 12.082 1.00 0.00 MEMB H

ATOM 5143 C25 POPC 39 -9.809 -26.314 11.978 1.00 0.00 MEMB C

ATOM 5144 H5R POPC 39 -9.104 -25.611 12.475 1.00 0.00 MEMB H

ATOM 5145 H5S POPC 39 -10.827 -25.865 12.034 1.00 0.00 MEMB H

ATOM 5146 C26 POPC 39 -9.433 -26.459 10.502 1.00 0.00 MEMB C

ATOM 5147 H6R POPC 39 -10.193 -27.075 9.969 1.00 0.00 MEMB H

ATOM 5148 H6S POPC 39 -8.445 -26.962 10.416 1.00 0.00 MEMB H

ATOM 5149 C27 POPC 39 -9.320 -25.141 9.753 1.00 0.00 MEMB C

ATOM 5150 H7R POPC 39 -8.549 -24.508 10.257 1.00 0.00 MEMB H

ATOM 5151 H7S POPC 39 -10.289 -24.590 9.818 1.00 0.00 MEMB H

ATOM 5152 C28 POPC 39 -8.949 -25.441 8.292 1.00 0.00 MEMB C

ATOM 5153 H8R POPC 39 -9.780 -25.949 7.765 1.00 0.00 MEMB H

ATOM 5154 H8S POPC 39 -8.010 -26.023 8.210 1.00 0.00 MEMB H

ATOM 5155 C29 POPC 39 -8.745 -24.149 7.656 1.00 0.00 MEMB C

ATOM 5156 H91 POPC 39 -8.936 -23.363 8.387 1.00 0.00 MEMB H

ATOM 5157 C210 POPC 39 -8.447 -23.892 6.386 1.00 0.00 MEMB C

ATOM 5158 H101 POPC 39 -8.454 -22.844 6.086 1.00 0.00 MEMB H

ATOM 5159 C211 POPC 39 -8.252 -24.904 5.287 1.00 0.00 MEMB C

ATOM 5160 H11R POPC 39 -9.011 -25.696 5.375 1.00 0.00 MEMB H

ATOM 5161 H11S POPC 39 -7.195 -25.250 5.227 1.00 0.00 MEMB H

ATOM 5162 C212 POPC 39 -8.511 -24.314 3.933 1.00 0.00 MEMB C

ATOM 5163 H12R POPC 39 -7.774 -23.491 4.094 1.00 0.00 MEMB H

ATOM 5164 H12S POPC 39 -9.566 -24.059 3.728 1.00 0.00 MEMB H

ATOM 5165 C213 POPC 39 -7.802 -24.720 2.702 1.00 0.00 MEMB C

ATOM 5166 H13R POPC 39 -7.554 -25.778 2.845 1.00 0.00 MEMB H

ATOM 5167 H13S POPC 39 -6.894 -24.150 2.628 1.00 0.00 MEMB H

ATOM 5168 C214 POPC 39 -8.413 -24.398 1.377 1.00 0.00 MEMB C

ATOM 5169 H14R POPC 39 -8.178 -23.412 0.884 1.00 0.00 MEMB H

ATOM 5170 H14S POPC 39 -9.523 -24.485 1.398 1.00 0.00 MEMB H

ATOM 5171 C215 POPC 39 -7.722 -25.563 0.678 1.00 0.00 MEMB C

ATOM 5172 H15R POPC 39 -7.951 -26.467 1.217 1.00 0.00 MEMB H

ATOM 5173 H15S POPC 39 -6.669 -25.299 0.535 1.00 0.00 MEMB H

ATOM 5174 C216 POPC 39 -8.462 -26.032 -0.458 1.00 0.00 MEMB C

ATOM 5175 H16R POPC 39 -8.362 -25.179 -1.092 1.00 0.00 MEMB H

ATOM 5176 H16S POPC 39 -8.968 -25.110 -0.442 1.00 0.00 MEMB H

ATOM 5177 C217 POPC 39 -8.131 -27.252 -1.382 1.00 0.00 MEMB C

ATOM 5178 H17R POPC 39 -7.044 -27.324 -1.562 1.00 0.00 MEMB H

ATOM 5179 H17S POPC 39 -8.613 -27.161 -2.384 1.00 0.00 MEMB H

ATOM 5180 C218 POPC 39 -8.774 -28.565 -1.521 1.00 0.00 MEMB C

ATOM 5181 H18R POPC 39 -9.829 -28.433 -1.873 1.00 0.00 MEMB H

ATOM 5182 H18S POPC 39 -8.655 -28.945 -0.509 1.00 0.00 MEMB H

ATOM 5183 H18T POPC 39 -8.215 -29.088 -2.355 1.00 0.00 MEMB H

ATOM 5184 C33 POPC 39 -4.050 -30.361 10.517 1.00 0.00 MEMB C

ATOM 5185 H3X POPC 39 -4.433 -31.351 10.824 1.00 0.00 MEMB H

ATOM 5186 H3Y POPC 39 -3.773 -30.385 9.436 1.00 0.00 MEMB H

ATOM 5187 C34 POPC 39 -5.194 -29.347 10.745 1.00 0.00 MEMB C

ATOM 5188 H4X POPC 39 -4.935 -28.361 10.397 1.00 0.00 MEMB H

ATOM 5189 H4Y POPC 39 -5.445 -29.226 11.816 1.00 0.00 MEMB H

ATOM 5190 C35 POPC 39 -6.417 -29.462 9.865 1.00 0.00 MEMB C

ATOM 5191 H5X POPC 39 -6.092 -29.289 8.815 1.00 0.00 MEMB H

ATOM 5192 H5Y POPC 39 -7.135 -28.645 10.114 1.00 0.00 MEMB H

ATOM 5193 C36 POPC 39 -7.089 -30.801 10.009 1.00 0.00 MEMB C

ATOM 5194 H6X POPC 39 -7.398 -30.937 11.069 1.00 0.00 MEMB H

ATOM 5195 H6Y POPC 39 -6.366 -31.609 9.761 1.00 0.00 MEMB H

ATOM 5196 C37 POPC 39 -8.318 -30.848 9.112 1.00 0.00 MEMB C

ATOM 5197 H7X POPC 39 -8.850 -29.869 9.144 1.00 0.00 MEMB H

ATOM 5198 H7Y POPC 39 -8.978 -31.635 9.538 1.00 0.00 MEMB H

ATOM 5199 C38 POPC 39 -7.992 -31.224 7.665 1.00 0.00 MEMB C

ATOM 5200 H8X POPC 39 -8.791 -31.915 7.311 1.00 0.00 MEMB H

ATOM 5201 H8Y POPC 39 -7.030 -31.780 7.640 1.00 0.00 MEMB H

ATOM 5202 C39 POPC 39 -7.928 -30.031 6.703 1.00 0.00 MEMB C

ATOM 5203 H9X POPC 39 -7.107 -29.348 7.004 1.00 0.00 MEMB H

ATOM 5204 H9Y POPC 39 -8.865 -29.434 6.772 1.00 0.00 MEMB H

ATOM 5205 C310 POPC 39 -7.787 -30.499 5.247 1.00 0.00 MEMB C

ATOM 5206 H10X POPC 39 -8.595 -31.231 5.034 1.00 0.00 MEMB H

ATOM 5207 H10Y POPC 39 -6.802 -30.998 5.085 1.00 0.00 MEMB H

ATOM 5208 C311 POPC 39 -7.975 -29.351 4.257 1.00 0.00 MEMB C

ATOM 5209 H11X POPC 39 -7.084 -28.688 4.416 1.00 0.00 MEMB H

ATOM 5210 H11Y POPC 39 -8.855 -28.749 4.532 1.00 0.00 MEMB H

ATOM 5211 C312 POPC 39 -8.141 -29.810 2.785 1.00 0.00 MEMB C

ATOM 5212 H12X POPC 39 -8.781 -29.118 2.192 1.00 0.00 MEMB H

ATOM 5213 H12Y POPC 39 -8.396 -30.875 2.617 1.00 0.00 MEMB H

ATOM 5214 C313 POPC 39 -6.807 -29.717 2.188 1.00 0.00 MEMB C

ATOM 5215 H13X POPC 39 -6.503 -30.373 1.337 1.00 0.00 MEMB H

ATOM 5216 H13Y POPC 39 -6.240 -29.950 3.098 1.00 0.00 MEMB H

ATOM 5217 C314 POPC 39 -6.569 -28.324 1.868 1.00 0.00 MEMB C

ATOM 5218 H14X POPC 39 -6.645 -27.580 2.703 1.00 0.00 MEMB H

ATOM 5219 H14Y POPC 39 -7.249 -28.099 1.021 1.00 0.00 MEMB H

ATOM 5220 C315 POPC 39 -5.210 -28.240 1.472 1.00 0.00 MEMB C

ATOM 5221 H15X POPC 39 -5.538 -27.617 0.650 1.00 0.00 MEMB H

ATOM 5222 H15Y POPC 39 -4.683 -28.869 0.725 1.00 0.00 MEMB H

ATOM 5223 C316 POPC 39 -4.389 -27.687 2.685 1.00 0.00 MEMB C

ATOM 5224 H16X POPC 39 -4.120 -28.307 3.541 1.00 0.00 MEMB H

ATOM 5225 H16Y POPC 39 -4.286 -26.635 2.963 1.00 0.00 MEMB H

ATOM 5226 H16Z POPC 39 -3.497 -27.752 2.109 1.00 0.00 MEMB H

ATOM 5227 N POPC 40 26.777 -8.874 17.548 1.00 0.00 MEMB N

ATOM 5228 C12 POPC 40 27.795 -7.826 17.878 1.00 0.00 MEMB C

ATOM 5229 H12A POPC 40 27.391 -6.836 17.706 1.00 0.00 MEMB H

ATOM 5230 H12B POPC 40 28.625 -7.989 17.192 1.00 0.00 MEMB H

ATOM 5231 C13 POPC 40 25.467 -8.829 18.302 1.00 0.00 MEMB C

ATOM 5232 H13A POPC 40 24.786 -9.647 18.061 1.00 0.00 MEMB H

ATOM 5233 H13B POPC 40 25.613 -8.868 19.383 1.00 0.00 MEMB H

ATOM 5234 H13C POPC 40 24.945 -7.901 18.102 1.00 0.00 MEMB H

ATOM 5235 C14 POPC 40 26.420 -8.892 16.086 1.00 0.00 MEMB C

ATOM 5236 H14A POPC 40 27.294 -9.033 15.468 1.00 0.00 MEMB H

ATOM 5237 H14B POPC 40 25.700 -9.678 15.855 1.00 0.00 MEMB H

ATOM 5238 H14C POPC 40 25.953 -7.954 15.813 1.00 0.00 MEMB H

ATOM 5239 C15 POPC 40 27.530 -10.062 17.951 1.00 0.00 MEMB C

ATOM 5240 H15A POPC 40 28.058 -10.151 18.881 1.00 0.00 MEMB H

ATOM 5241 H15B POPC 40 28.093 -10.440 17.094 1.00 0.00 MEMB H

ATOM 5242 H15C POPC 40 26.751 -10.818 18.095 1.00 0.00 MEMB H

ATOM 5243 C11 POPC 40 28.409 -7.926 19.310 1.00 0.00 MEMB C

ATOM 5244 H11A POPC 40 29.291 -7.255 19.365 1.00 0.00 MEMB H

ATOM 5245 H11B POPC 40 28.842 -8.911 19.579 1.00 0.00 MEMB H

ATOM 5246 P POPC 40 26.814 -6.220 20.484 1.00 0.00 MEMB P

ATOM 5247 O13 POPC 40 27.008 -5.843 21.903 1.00 0.00 MEMB O

ATOM 5248 O14 POPC 40 25.440 -6.244 19.924 1.00 0.00 MEMB O

ATOM 5249 O12 POPC 40 27.444 -7.658 20.313 1.00 0.00 MEMB O

ATOM 5250 O11 POPC 40 27.815 -5.352 19.574 1.00 0.00 MEMB O

ATOM 5251 C1 POPC 40 27.716 -3.936 19.715 1.00 0.00 MEMB C

ATOM 5252 HA POPC 40 28.684 -3.469 19.413 1.00 0.00 MEMB H

ATOM 5253 HB POPC 40 27.573 -3.592 20.764 1.00 0.00 MEMB H

ATOM 5254 C2 POPC 40 26.600 -3.363 18.814 1.00 0.00 MEMB C

ATOM 5255 HS POPC 40 26.073 -2.589 19.437 1.00 0.00 MEMB H

ATOM 5256 O21 POPC 40 25.733 -4.311 18.167 1.00 0.00 MEMB O

ATOM 5257 C21 POPC 40 24.474 -4.104 17.997 1.00 0.00 MEMB C

ATOM 5258 O22 POPC 40 23.699 -3.389 18.542 1.00 0.00 MEMB O

ATOM 5259 C22 POPC 40 23.804 -4.982 17.001 1.00 0.00 MEMB C

ATOM 5260 H2R POPC 40 22.864 -5.166 17.587 1.00 0.00 MEMB H

ATOM 5261 H2S POPC 40 24.415 -5.882 16.897 1.00 0.00 MEMB H

ATOM 5262 C3 POPC 40 27.270 -2.685 17.690 1.00 0.00 MEMB C

ATOM 5263 HX POPC 40 27.973 -1.922 18.071 1.00 0.00 MEMB H

ATOM 5264 HY POPC 40 26.479 -2.098 17.224 1.00 0.00 MEMB H

ATOM 5265 O31 POPC 40 27.744 -3.743 16.763 1.00 0.00 MEMB O

ATOM 5266 C31 POPC 40 27.831 -3.108 15.601 1.00 0.00 MEMB C

ATOM 5267 O32 POPC 40 27.730 -1.890 15.639 1.00 0.00 MEMB O

ATOM 5268 C32 POPC 40 28.638 -4.083 14.547 1.00 0.00 MEMB C

ATOM 5269 H2X POPC 40 27.858 -4.611 14.017 1.00 0.00 MEMB H

ATOM 5270 H2Y POPC 40 29.211 -4.743 15.224 1.00 0.00 MEMB H

ATOM 5271 C23 POPC 40 23.338 -4.310 15.689 1.00 0.00 MEMB C

ATOM 5272 H3R POPC 40 22.860 -3.313 15.807 1.00 0.00 MEMB H

ATOM 5273 H3S POPC 40 22.559 -4.976 15.257 1.00 0.00 MEMB H

ATOM 5274 C24 POPC 40 24.474 -4.220 14.688 1.00 0.00 MEMB C

ATOM 5275 H4R POPC 40 24.757 -5.264 14.437 1.00 0.00 MEMB H

ATOM 5276 H4S POPC 40 25.347 -3.764 15.208 1.00 0.00 MEMB H

ATOM 5277 C25 POPC 40 24.065 -3.388 13.460 1.00 0.00 MEMB C

ATOM 5278 H5R POPC 40 23.700 -2.387 13.744 1.00 0.00 MEMB H

ATOM 5279 H5S POPC 40 23.182 -3.836 12.966 1.00 0.00 MEMB H

ATOM 5280 C26 POPC 40 25.190 -3.196 12.446 1.00 0.00 MEMB C

ATOM 5281 H6R POPC 40 26.134 -3.054 12.999 1.00 0.00 MEMB H

ATOM 5282 H6S POPC 40 25.000 -2.261 11.867 1.00 0.00 MEMB H

ATOM 5283 C27 POPC 40 25.218 -4.412 11.512 1.00 0.00 MEMB C

ATOM 5284 H7R POPC 40 24.194 -4.500 11.075 1.00 0.00 MEMB H

ATOM 5285 H7S POPC 40 25.429 -5.313 12.128 1.00 0.00 MEMB H

ATOM 5286 C28 POPC 40 26.200 -4.392 10.342 1.00 0.00 MEMB C

ATOM 5287 H8R POPC 40 26.009 -5.280 9.703 1.00 0.00 MEMB H

ATOM 5288 H8S POPC 40 27.239 -4.516 10.710 1.00 0.00 MEMB H

ATOM 5289 C29 POPC 40 26.046 -3.123 9.560 1.00 0.00 MEMB C

ATOM 5290 H91 POPC 40 26.378 -2.230 10.040 1.00 0.00 MEMB H

ATOM 5291 C210 POPC 40 25.373 -2.840 8.482 1.00 0.00 MEMB C

ATOM 5292 H101 POPC 40 25.338 -1.787 8.144 1.00 0.00 MEMB H

ATOM 5293 C211 POPC 40 24.566 -3.857 7.820 1.00 0.00 MEMB C

ATOM 5294 H11R POPC 40 23.681 -3.569 8.377 1.00 0.00 MEMB H

ATOM 5295 H11S POPC 40 24.749 -4.901 8.124 1.00 0.00 MEMB H

ATOM 5296 C212 POPC 40 24.525 -3.772 6.300 1.00 0.00 MEMB C

ATOM 5297 H12R POPC 40 24.300 -2.731 6.065 1.00 0.00 MEMB H

ATOM 5298 H12S POPC 40 23.682 -4.370 5.894 1.00 0.00 MEMB H

ATOM 5299 C213 POPC 40 25.844 -4.234 5.658 1.00 0.00 MEMB C

ATOM 5300 H13R POPC 40 26.236 -5.040 6.320 1.00 0.00 MEMB H

ATOM 5301 H13S POPC 40 26.569 -3.395 5.701 1.00 0.00 MEMB H

ATOM 5302 C214 POPC 40 25.686 -4.776 4.215 1.00 0.00 MEMB C

ATOM 5303 H14R POPC 40 24.906 -5.563 4.281 1.00 0.00 MEMB H

ATOM 5304 H14S POPC 40 26.607 -5.292 3.881 1.00 0.00 MEMB H

ATOM 5305 C215 POPC 40 25.326 -3.652 3.200 1.00 0.00 MEMB C

ATOM 5306 H15R POPC 40 26.062 -2.841 3.352 1.00 0.00 MEMB H

ATOM 5307 H15S POPC 40 24.312 -3.310 3.502 1.00 0.00 MEMB H

ATOM 5308 C216 POPC 40 25.368 -3.925 1.652 1.00 0.00 MEMB C

ATOM 5309 H16R POPC 40 26.112 -4.730 1.471 1.00 0.00 MEMB H

ATOM 5310 H16S POPC 40 25.537 -3.044 1.016 1.00 0.00 MEMB H

ATOM 5311 C217 POPC 40 24.067 -4.427 1.072 1.00 0.00 MEMB C

ATOM 5312 H17R POPC 40 24.149 -4.942 2.018 1.00 0.00 MEMB H

ATOM 5313 H17S POPC 40 24.067 -5.215 0.294 1.00 0.00 MEMB H

ATOM 5314 C218 POPC 40 22.718 -3.764 1.260 1.00 0.00 MEMB C

ATOM 5315 H18R POPC 40 22.580 -3.605 2.343 1.00 0.00 MEMB H

ATOM 5316 H18S POPC 40 21.840 -4.371 0.933 1.00 0.00 MEMB H

ATOM 5317 H18T POPC 40 22.657 -2.820 0.784 1.00 0.00 MEMB H

ATOM 5318 C33 POPC 40 29.792 -3.936 13.420 1.00 0.00 MEMB C

ATOM 5319 H3X POPC 40 30.621 -3.160 13.295 1.00 0.00 MEMB H

ATOM 5320 H3Y POPC 40 30.279 -3.061 12.921 1.00 0.00 MEMB H

ATOM 5321 C34 POPC 40 30.288 -5.132 13.018 1.00 0.00 MEMB C

ATOM 5322 H4X POPC 40 30.841 -5.650 13.789 1.00 0.00 MEMB H

ATOM 5323 H4Y POPC 40 31.029 -4.620 12.520 1.00 0.00 MEMB H

ATOM 5324 C35 POPC 40 29.822 -5.463 11.677 1.00 0.00 MEMB C

ATOM 5325 H5X POPC 40 29.355 -4.522 11.313 1.00 0.00 MEMB H

ATOM 5326 H5Y POPC 40 28.877 -5.942 11.838 1.00 0.00 MEMB H

ATOM 5327 C36 POPC 40 30.962 -6.114 10.836 1.00 0.00 MEMB C

ATOM 5328 H6X POPC 40 31.511 -6.765 11.519 1.00 0.00 MEMB H

ATOM 5329 H6Y POPC 40 31.936 -5.629 10.549 1.00 0.00 MEMB H

ATOM 5330 C37 POPC 40 30.441 -6.362 9.430 1.00 0.00 MEMB C

ATOM 5331 H7X POPC 40 31.251 -6.468 8.669 1.00 0.00 MEMB H

ATOM 5332 H7Y POPC 40 29.833 -5.536 9.012 1.00 0.00 MEMB H

ATOM 5333 C38 POPC 40 29.151 -7.071 9.340 1.00 0.00 MEMB C

ATOM 5334 H8X POPC 40 28.245 -6.412 9.473 1.00 0.00 MEMB H

ATOM 5335 H8Y POPC 40 29.252 -7.724 10.221 1.00 0.00 MEMB H

ATOM 5336 C39 POPC 40 29.232 -7.711 7.981 1.00 0.00 MEMB C

ATOM 5337 H9X POPC 40 28.679 -8.657 8.069 1.00 0.00 MEMB H

ATOM 5338 H9Y POPC 40 30.307 -7.966 7.795 1.00 0.00 MEMB H

ATOM 5339 C310 POPC 40 28.786 -6.884 6.770 1.00 0.00 MEMB C

ATOM 5340 H10X POPC 40 29.201 -5.846 6.728 1.00 0.00 MEMB H

ATOM 5341 H10Y POPC 40 27.680 -6.790 6.722 1.00 0.00 MEMB H

ATOM 5342 C311 POPC 40 29.288 -7.621 5.545 1.00 0.00 MEMB C

ATOM 5343 H11X POPC 40 29.095 -8.713 5.591 1.00 0.00 MEMB H

ATOM 5344 H11Y POPC 40 30.392 -7.464 5.447 1.00 0.00 MEMB H

ATOM 5345 C312 POPC 40 28.675 -7.094 4.305 1.00 0.00 MEMB C

ATOM 5346 H12X POPC 40 28.674 -5.977 4.335 1.00 0.00 MEMB H

ATOM 5347 H12Y POPC 40 27.650 -7.470 4.060 1.00 0.00 MEMB H

ATOM 5348 C313 POPC 40 29.594 -7.427 3.203 1.00 0.00 MEMB C

ATOM 5349 H13X POPC 40 29.603 -8.541 3.011 1.00 0.00 MEMB H

ATOM 5350 H13Y POPC 40 30.651 -7.139 3.337 1.00 0.00 MEMB H

ATOM 5351 C314 POPC 40 29.119 -6.463 2.148 1.00 0.00 MEMB C

ATOM 5352 H14X POPC 40 29.912 -5.853 1.725 1.00 0.00 MEMB H

ATOM 5353 H14Y POPC 40 28.247 -5.833 2.416 1.00 0.00 MEMB H

ATOM 5354 C315 POPC 40 28.569 -7.234 1.115 1.00 0.00 MEMB C

ATOM 5355 H15X POPC 40 28.050 -8.003 1.735 1.00 0.00 MEMB H

ATOM 5356 H15Y POPC 40 29.152 -8.158 0.919 1.00 0.00 MEMB H

ATOM 5357 C316 POPC 40 27.976 -6.381 -0.205 1.00 0.00 MEMB C

ATOM 5358 H16X POPC 40 28.386 -6.847 -1.113 1.00 0.00 MEMB H

ATOM 5359 H16Y POPC 40 27.237 -5.574 -0.153 1.00 0.00 MEMB H

ATOM 5360 H16Z POPC 40 27.119 -6.726 -0.825 1.00 0.00 MEMB H

ATOM 5361 N POPC 41 -14.933 24.611 18.253 1.00 0.00 MEMB N

ATOM 5362 C12 POPC 41 -14.919 25.487 19.486 1.00 0.00 MEMB C

ATOM 5363 H12A POPC 41 -13.959 25.998 19.477 1.00 0.00 MEMB H

ATOM 5364 H12B POPC 41 -14.973 24.846 20.357 1.00 0.00 MEMB H

ATOM 5365 C13 POPC 41 -16.232 23.862 18.142 1.00 0.00 MEMB C

ATOM 5366 H13A POPC 41 -16.384 23.219 19.003 1.00 0.00 MEMB H

ATOM 5367 H13B POPC 41 -17.071 24.559 18.112 1.00 0.00 MEMB H

ATOM 5368 H13C POPC 41 -16.260 23.243 17.255 1.00 0.00 MEMB H

ATOM 5369 C14 POPC 41 -14.693 25.462 17.041 1.00 0.00 MEMB C

ATOM 5370 H14A POPC 41 -15.554 26.087 16.824 1.00 0.00 MEMB H

ATOM 5371 H14B POPC 41 -13.830 26.111 17.201 1.00 0.00 MEMB H

ATOM 5372 H14C POPC 41 -14.470 24.854 16.176 1.00 0.00 MEMB H

ATOM 5373 C15 POPC 41 -13.796 23.641 18.323 1.00 0.00 MEMB C

ATOM 5374 H15A POPC 41 -13.840 23.049 19.232 1.00 0.00 MEMB H

ATOM 5375 H15B POPC 41 -12.848 24.179 18.296 1.00 0.00 MEMB H

ATOM 5376 H15C POPC 41 -13.817 22.945 17.489 1.00 0.00 MEMB H

ATOM 5377 C11 POPC 41 -16.024 26.559 19.664 1.00 0.00 MEMB C

ATOM 5378 H11A POPC 41 -16.073 27.155 18.721 1.00 0.00 MEMB H

ATOM 5379 H11B POPC 41 -15.669 27.242 20.472 1.00 0.00 MEMB H

ATOM 5380 P POPC 41 -18.656 26.655 19.613 1.00 0.00 MEMB P

ATOM 5381 O13 POPC 41 -18.738 27.986 20.266 1.00 0.00 MEMB O

ATOM 5382 O14 POPC 41 -19.800 25.715 19.760 1.00 0.00 MEMB O

ATOM 5383 O12 POPC 41 -17.279 25.972 20.048 1.00 0.00 MEMB O

ATOM 5384 O11 POPC 41 -18.425 26.908 18.102 1.00 0.00 MEMB O

ATOM 5385 C1 POPC 41 -19.600 26.874 17.302 1.00 0.00 MEMB C

ATOM 5386 HA POPC 41 -20.164 27.831 17.384 1.00 0.00 MEMB H

ATOM 5387 HB POPC 41 -20.294 26.020 17.512 1.00 0.00 MEMB H

ATOM 5388 C2 POPC 41 -19.128 26.616 15.905 1.00 0.00 MEMB C

ATOM 5389 HS POPC 41 -20.076 26.457 15.314 1.00 0.00 MEMB H

ATOM 5390 O21 POPC 41 -18.160 25.573 15.818 1.00 0.00 MEMB O

ATOM 5391 C21 POPC 41 -18.624 24.568 15.219 1.00 0.00 MEMB C

ATOM 5392 O22 POPC 41 -19.802 24.243 15.134 1.00 0.00 MEMB O

ATOM 5393 C22 POPC 41 -17.444 23.857 14.612 1.00 0.00 MEMB C

ATOM 5394 H2R POPC 41 -16.853 23.392 15.432 1.00 0.00 MEMB H

ATOM 5395 H2S POPC 41 -16.799 24.461 13.949 1.00 0.00 MEMB H

ATOM 5396 C3 POPC 41 -18.404 27.793 15.320 1.00 0.00 MEMB C

ATOM 5397 HX POPC 41 -17.766 28.162 16.143 1.00 0.00 MEMB H

ATOM 5398 HY POPC 41 -19.190 28.558 15.142 1.00 0.00 MEMB H

ATOM 5399 O31 POPC 41 -17.534 27.420 14.194 1.00 0.00 MEMB O

ATOM 5400 C31 POPC 41 -18.300 27.033 13.155 1.00 0.00 MEMB C

ATOM 5401 O32 POPC 41 -19.502 26.909 13.282 1.00 0.00 MEMB O

ATOM 5402 C32 POPC 41 -17.509 27.065 11.784 1.00 0.00 MEMB C

ATOM 5403 H2X POPC 41 -16.551 26.595 12.002 1.00 0.00 MEMB H

ATOM 5404 H2Y POPC 41 -17.242 28.062 11.381 1.00 0.00 MEMB H

ATOM 5405 C23 POPC 41 -18.038 22.743 13.825 1.00 0.00 MEMB C

ATOM 5406 H3R POPC 41 -18.788 22.311 14.491 1.00 0.00 MEMB H

ATOM 5407 H3S POPC 41 -17.156 22.069 13.727 1.00 0.00 MEMB H

ATOM 5408 C24 POPC 41 -18.690 23.042 12.462 1.00 0.00 MEMB C

ATOM 5409 H4R POPC 41 -17.846 22.995 11.736 1.00 0.00 MEMB H

ATOM 5410 H4S POPC 41 -19.155 24.039 12.503 1.00 0.00 MEMB H

ATOM 5411 C25 POPC 41 -19.809 22.114 11.883 1.00 0.00 MEMB C

ATOM 5412 H5R POPC 41 -20.763 22.386 12.334 1.00 0.00 MEMB H

ATOM 5413 H5S POPC 41 -19.905 21.079 12.174 1.00 0.00 MEMB H

ATOM 5414 C26 POPC 41 -19.739 22.079 10.358 1.00 0.00 MEMB C

ATOM 5415 H6R POPC 41 -20.476 21.343 9.961 1.00 0.00 MEMB H

ATOM 5416 H6S POPC 41 -18.769 21.704 9.977 1.00 0.00 MEMB H

ATOM 5417 C27 POPC 41 -19.960 23.546 10.000 1.00 0.00 MEMB C

ATOM 5418 H7R POPC 41 -19.062 24.093 10.146 1.00 0.00 MEMB H

ATOM 5419 H7S POPC 41 -20.583 23.948 10.838 1.00 0.00 MEMB H

ATOM 5420 C28 POPC 41 -20.539 23.913 8.634 1.00 0.00 MEMB C

ATOM 5421 H8R POPC 41 -21.009 24.913 8.535 1.00 0.00 MEMB H

ATOM 5422 H8S POPC 41 -21.481 23.459 8.452 1.00 0.00 MEMB H

ATOM 5423 C29 POPC 41 -19.785 23.214 7.576 1.00 0.00 MEMB C

ATOM 5424 H91 POPC 41 -19.300 22.290 7.906 1.00 0.00 MEMB H

ATOM 5425 C210 POPC 41 -20.068 23.269 6.299 1.00 0.00 MEMB C

ATOM 5426 H101 POPC 41 -19.794 22.397 5.678 1.00 0.00 MEMB H

ATOM 5427 C211 POPC 41 -21.198 24.060 5.768 1.00 0.00 MEMB C

ATOM 5428 H11R POPC 41 -22.021 24.403 6.424 1.00 0.00 MEMB H

ATOM 5429 H11S POPC 41 -21.524 23.304 5.045 1.00 0.00 MEMB H

ATOM 5430 C212 POPC 41 -21.183 25.015 4.725 1.00 0.00 MEMB C

ATOM 5431 H12R POPC 41 -20.557 25.831 5.118 1.00 0.00 MEMB H

ATOM 5432 H12S POPC 41 -22.229 25.345 4.455 1.00 0.00 MEMB H

ATOM 5433 C213 POPC 41 -20.568 24.247 3.633 1.00 0.00 MEMB C

ATOM 5434 H13R POPC 41 -21.108 23.405 3.159 1.00 0.00 MEMB H

ATOM 5435 H13S POPC 41 -19.614 23.748 3.906 1.00 0.00 MEMB H

ATOM 5436 C214 POPC 41 -20.537 25.295 2.742 1.00 0.00 MEMB C

ATOM 5437 H14R POPC 41 -20.074 26.226 3.131 1.00 0.00 MEMB H

ATOM 5438 H14S POPC 41 -21.628 25.487 2.425 1.00 0.00 MEMB H

ATOM 5439 C215 POPC 41 -19.692 24.491 1.844 1.00 0.00 MEMB C

ATOM 5440 H15R POPC 41 -20.140 25.318 1.301 1.00 0.00 MEMB H

ATOM 5441 H15S POPC 41 -20.248 23.779 1.276 1.00 0.00 MEMB H

ATOM 5442 C216 POPC 41 -18.057 24.248 1.926 1.00 0.00 MEMB C

ATOM 5443 H16R POPC 41 -17.867 23.669 2.840 1.00 0.00 MEMB H

ATOM 5444 H16S POPC 41 -17.587 25.238 2.059 1.00 0.00 MEMB H

ATOM 5445 C217 POPC 41 -17.086 23.717 0.697 1.00 0.00 MEMB C

ATOM 5446 H17R POPC 41 -16.287 24.357 0.264 1.00 0.00 MEMB H

ATOM 5447 H17S POPC 41 -17.534 23.695 -0.308 1.00 0.00 MEMB H

ATOM 5448 C218 POPC 41 -16.890 22.265 0.627 1.00 0.00 MEMB C

ATOM 5449 H18R POPC 41 -16.054 21.869 1.185 1.00 0.00 MEMB H

ATOM 5450 H18S POPC 41 -16.988 21.810 -0.350 1.00 0.00 MEMB H

ATOM 5451 H18T POPC 41 -17.828 21.974 1.001 1.00 0.00 MEMB H

ATOM 5452 C33 POPC 41 -18.282 26.284 10.704 1.00 0.00 MEMB C

ATOM 5453 H3X POPC 41 -19.172 26.705 10.252 1.00 0.00 MEMB H

ATOM 5454 H3Y POPC 41 -18.746 25.489 11.289 1.00 0.00 MEMB H

ATOM 5455 C34 POPC 41 -17.680 25.963 9.343 1.00 0.00 MEMB C

ATOM 5456 H4X POPC 41 -17.381 26.876 8.787 1.00 0.00 MEMB H

ATOM 5457 H4Y POPC 41 -18.508 25.511 8.736 1.00 0.00 MEMB H

ATOM 5458 C35 POPC 41 -16.548 24.995 9.460 1.00 0.00 MEMB C

ATOM 5459 H5X POPC 41 -16.918 24.089 9.991 1.00 0.00 MEMB H

ATOM 5460 H5Y POPC 41 -15.789 25.496 10.097 1.00 0.00 MEMB H

ATOM 5461 C36 POPC 41 -15.910 24.685 8.106 1.00 0.00 MEMB C

ATOM 5462 H6X POPC 41 -15.252 23.807 8.260 1.00 0.00 MEMB H

ATOM 5463 H6Y POPC 41 -15.289 25.558 7.823 1.00 0.00 MEMB H

ATOM 5464 C37 POPC 41 -16.878 24.467 6.937 1.00 0.00 MEMB C

ATOM 5465 H7X POPC 41 -17.592 25.318 6.851 1.00 0.00 MEMB H

ATOM 5466 H7Y POPC 41 -17.443 23.543 7.152 1.00 0.00 MEMB H

ATOM 5467 C38 POPC 41 -16.210 24.274 5.568 1.00 0.00 MEMB C

ATOM 5468 H8X POPC 41 -17.032 24.078 4.839 1.00 0.00 MEMB H

ATOM 5469 H8Y POPC 41 -15.595 23.352 5.612 1.00 0.00 MEMB H

ATOM 5470 C39 POPC 41 -15.372 25.464 5.048 1.00 0.00 MEMB C

ATOM 5471 H9X POPC 41 -14.307 25.161 4.936 1.00 0.00 MEMB H

ATOM 5472 H9Y POPC 41 -15.426 26.328 5.743 1.00 0.00 MEMB H

ATOM 5473 C310 POPC 41 -15.877 25.907 3.674 1.00 0.00 MEMB C

ATOM 5474 H10X POPC 41 -16.958 26.164 3.785 1.00 0.00 MEMB H

ATOM 5475 H10Y POPC 41 -15.766 25.021 3.010 1.00 0.00 MEMB H

ATOM 5476 C311 POPC 41 -15.172 27.090 3.008 1.00 0.00 MEMB C

ATOM 5477 H11X POPC 41 -14.224 26.751 2.532 1.00 0.00 MEMB H

ATOM 5478 H11Y POPC 41 -14.918 27.820 3.808 1.00 0.00 MEMB H

ATOM 5479 C312 POPC 41 -16.073 27.767 1.951 1.00 0.00 MEMB C

ATOM 5480 H12X POPC 41 -17.064 27.976 2.402 1.00 0.00 MEMB H

ATOM 5481 H12Y POPC 41 -16.187 27.115 1.057 1.00 0.00 MEMB H

ATOM 5482 C313 POPC 41 -15.509 29.092 1.483 1.00 0.00 MEMB C

ATOM 5483 H13X POPC 41 -14.576 28.817 0.944 1.00 0.00 MEMB H

ATOM 5484 H13Y POPC 41 -15.218 29.654 2.397 1.00 0.00 MEMB H

ATOM 5485 C314 POPC 41 -16.416 30.033 0.661 1.00 0.00 MEMB C

ATOM 5486 H14X POPC 41 -15.919 31.032 0.566 1.00 0.00 MEMB H

ATOM 5487 H14Y POPC 41 -17.406 30.153 1.139 1.00 0.00 MEMB H

ATOM 5488 C315 POPC 41 -16.730 29.566 -0.733 1.00 0.00 MEMB C

ATOM 5489 H15X POPC 41 -17.523 30.159 -1.206 1.00 0.00 MEMB H

ATOM 5490 H15Y POPC 41 -17.214 28.566 -0.694 1.00 0.00 MEMB H

ATOM 5491 C316 POPC 41 -15.482 29.457 -1.554 1.00 0.00 MEMB C

ATOM 5492 H16X POPC 41 -15.754 28.921 -2.485 1.00 0.00 MEMB H

ATOM 5493 H16Y POPC 41 -14.715 29.021 -0.910 1.00 0.00 MEMB H

ATOM 5494 H16Z POPC 41 -14.759 30.127 -1.987 1.00 0.00 MEMB H

ATOM 5495 N POPC 42 29.044 2.466 21.029 1.00 0.00 MEMB N

ATOM 5496 C12 POPC 42 29.474 3.436 19.916 1.00 0.00 MEMB C

ATOM 5497 H12A POPC 42 30.553 3.550 19.945 1.00 0.00 MEMB H

ATOM 5498 H12B POPC 42 29.032 4.406 20.105 1.00 0.00 MEMB H

ATOM 5499 C13 POPC 42 29.603 1.098 20.762 1.00 0.00 MEMB C

ATOM 5500 H13A POPC 42 29.333 0.390 21.526 1.00 0.00 MEMB H

ATOM 5501 H13B POPC 42 29.205 0.771 19.793 1.00 0.00 MEMB H

ATOM 5502 H13C POPC 42 30.675 1.114 20.611 1.00 0.00 MEMB H

ATOM 5503 C14 POPC 42 29.482 2.913 22.380 1.00 0.00 MEMB C

ATOM 5504 H14A POPC 42 29.082 3.896 22.591 1.00 0.00 MEMB H

ATOM 5505 H14B POPC 42 29.101 2.252 23.157 1.00 0.00 MEMB H

ATOM 5506 H14C POPC 42 30.557 2.941 22.476 1.00 0.00 MEMB H

ATOM 5507 C15 POPC 42 27.554 2.320 21.054 1.00 0.00 MEMB C

ATOM 5508 H15A POPC 42 27.186 1.969 20.090 1.00 0.00 MEMB H

ATOM 5509 H15B POPC 42 27.069 3.268 21.265 1.00 0.00 MEMB H

ATOM 5510 H15C POPC 42 27.245 1.608 21.809 1.00 0.00 MEMB H

ATOM 5511 C11 POPC 42 29.138 3.088 18.440 1.00 0.00 MEMB C

ATOM 5512 H11A POPC 42 29.627 3.802 17.736 1.00 0.00 MEMB H

ATOM 5513 H11B POPC 42 28.035 3.176 18.291 1.00 0.00 MEMB H

ATOM 5514 P POPC 42 28.880 1.001 16.984 1.00 0.00 MEMB P

ATOM 5515 O13 POPC 42 28.748 1.876 15.800 1.00 0.00 MEMB O

ATOM 5516 O14 POPC 42 27.680 0.407 17.626 1.00 0.00 MEMB O

ATOM 5517 O12 POPC 42 29.621 1.800 18.121 1.00 0.00 MEMB O

ATOM 5518 O11 POPC 42 30.028 -0.052 16.781 1.00 0.00 MEMB O

ATOM 5519 C1 POPC 42 30.465 -0.802 17.947 1.00 0.00 MEMB C

ATOM 5520 HA POPC 42 29.659 -1.390 18.397 1.00 0.00 MEMB H

ATOM 5521 HB POPC 42 30.949 -0.177 18.693 1.00 0.00 MEMB H

ATOM 5522 C2 POPC 42 31.463 -1.971 17.689 1.00 0.00 MEMB C

ATOM 5523 HS POPC 42 31.934 -2.179 18.640 1.00 0.00 MEMB H

ATOM 5524 O21 POPC 42 32.416 -1.565 16.679 1.00 0.00 MEMB O

ATOM 5525 C21 POPC 42 32.956 -0.465 16.955 1.00 0.00 MEMB C

ATOM 5526 O22 POPC 42 33.435 -0.205 18.055 1.00 0.00 MEMB O

ATOM 5527 C22 POPC 42 33.027 0.445 15.758 1.00 0.00 MEMB C

ATOM 5528 H2R POPC 42 32.588 0.155 14.770 1.00 0.00 MEMB H

ATOM 5529 H2S POPC 42 34.113 0.557 15.809 1.00 0.00 MEMB H

ATOM 5530 C3 POPC 42 30.708 -3.211 17.248 1.00 0.00 MEMB C

ATOM 5531 HX POPC 42 29.965 -3.767 17.843 1.00 0.00 MEMB H

ATOM 5532 HY POPC 42 31.324 -4.096 16.913 1.00 0.00 MEMB H

ATOM 5533 O31 POPC 42 29.907 -2.392 16.391 1.00 0.00 MEMB O

ATOM 5534 C31 POPC 42 30.398 -2.138 15.144 1.00 0.00 MEMB C

ATOM 5535 O32 POPC 42 31.389 -2.887 15.062 1.00 0.00 MEMB O

ATOM 5536 C32 POPC 42 29.308 -1.366 13.776 1.00 0.00 MEMB C

ATOM 5537 H2X POPC 42 29.848 -0.817 13.056 1.00 0.00 MEMB H

ATOM 5538 H2Y POPC 42 28.295 -1.723 13.768 1.00 0.00 MEMB H

ATOM 5539 C23 POPC 42 32.505 1.796 15.912 1.00 0.00 MEMB C

ATOM 5540 H3R POPC 42 32.717 2.082 16.965 1.00 0.00 MEMB H

ATOM 5541 H3S POPC 42 31.420 1.717 15.691 1.00 0.00 MEMB H

ATOM 5542 C24 POPC 42 33.214 2.683 14.900 1.00 0.00 MEMB C

ATOM 5543 H4R POPC 42 34.304 2.708 15.085 1.00 0.00 MEMB H

ATOM 5544 H4S POPC 42 32.784 3.648 15.207 1.00 0.00 MEMB H

ATOM 5545 C25 POPC 42 32.970 2.429 13.398 1.00 0.00 MEMB C

ATOM 5546 H5R POPC 42 33.111 1.378 13.106 1.00 0.00 MEMB H

ATOM 5547 H5S POPC 42 33.685 2.964 12.753 1.00 0.00 MEMB H

ATOM 5548 C26 POPC 42 31.623 2.999 12.975 1.00 0.00 MEMB C

ATOM 5549 H6R POPC 42 31.693 4.108 13.142 1.00 0.00 MEMB H

ATOM 5550 H6S POPC 42 30.837 2.611 13.650 1.00 0.00 MEMB H

ATOM 5551 C27 POPC 42 31.195 2.657 11.536 1.00 0.00 MEMB C

ATOM 5552 H7R POPC 42 30.348 3.350 11.302 1.00 0.00 MEMB H

ATOM 5553 H7S POPC 42 30.970 1.593 11.387 1.00 0.00 MEMB H

ATOM 5554 C28 POPC 42 32.208 2.924 10.476 1.00 0.00 MEMB C

ATOM 5555 H8R POPC 42 33.198 2.434 10.573 1.00 0.00 MEMB H

ATOM 5556 H8S POPC 42 32.400 3.889 11.014 1.00 0.00 MEMB H

ATOM 5557 C29 POPC 42 31.679 3.008 9.014 1.00 0.00 MEMB C

ATOM 5558 H91 POPC 42 31.369 4.000 8.651 1.00 0.00 MEMB H

ATOM 5559 C210 POPC 42 31.844 2.134 8.009 1.00 0.00 MEMB C

ATOM 5560 H101 POPC 42 31.743 2.462 6.966 1.00 0.00 MEMB H

ATOM 5561 C211 POPC 42 32.596 0.863 8.123 1.00 0.00 MEMB C

ATOM 5562 H11R POPC 42 32.050 -0.058 8.227 1.00 0.00 MEMB H

ATOM 5563 H11S POPC 42 33.046 0.996 9.136 1.00 0.00 MEMB H

ATOM 5564 C212 POPC 42 33.728 0.730 7.075 1.00 0.00 MEMB C

ATOM 5565 H12R POPC 42 34.499 -0.076 7.139 1.00 0.00 MEMB H

ATOM 5566 H12S POPC 42 34.102 1.606 7.527 1.00 0.00 MEMB H

ATOM 5567 C213 POPC 42 33.820 0.349 5.671 1.00 0.00 MEMB C

ATOM 5568 H13R POPC 42 34.866 0.043 5.302 1.00 0.00 MEMB H

ATOM 5569 H13S POPC 42 33.405 1.244 5.269 1.00 0.00 MEMB H

ATOM 5570 C214 POPC 42 32.898 -0.761 5.663 1.00 0.00 MEMB C

ATOM 5571 H14R POPC 42 31.907 -0.331 5.932 1.00 0.00 MEMB H

ATOM 5572 H14S POPC 42 33.247 -1.398 6.521 1.00 0.00 MEMB H

ATOM 5573 C215 POPC 42 32.957 -1.504 4.358 1.00 0.00 MEMB C

ATOM 5574 H15R POPC 42 32.409 -2.457 4.385 1.00 0.00 MEMB H

ATOM 5575 H15S POPC 42 34.022 -1.754 4.179 1.00 0.00 MEMB H

ATOM 5576 C216 POPC 42 32.300 -0.705 3.254 1.00 0.00 MEMB C

ATOM 5577 H16R POPC 42 32.948 0.174 3.101 1.00 0.00 MEMB H

ATOM 5578 H16S POPC 42 31.337 -0.296 3.619 1.00 0.00 MEMB H

ATOM 5579 C217 POPC 42 32.068 -1.521 1.967 1.00 0.00 MEMB C

ATOM 5580 H17R POPC 42 31.973 -0.770 1.150 1.00 0.00 MEMB H

ATOM 5581 H17S POPC 42 31.160 -2.150 2.031 1.00 0.00 MEMB H

ATOM 5582 C218 POPC 42 33.215 -2.452 1.637 1.00 0.00 MEMB C

ATOM 5583 H18R POPC 42 34.026 -1.866 2.057 1.00 0.00 MEMB H

ATOM 5584 H18S POPC 42 33.424 -2.588 0.561 1.00 0.00 MEMB H

ATOM 5585 H18T POPC 42 33.255 -3.421 2.168 1.00 0.00 MEMB H

ATOM 5586 C33 POPC 42 28.482 -2.061 12.289 1.00 0.00 MEMB C

ATOM 5587 H3X POPC 42 27.933 -3.043 12.380 1.00 0.00 MEMB H

ATOM 5588 H3Y POPC 42 29.326 -2.444 11.726 1.00 0.00 MEMB H

ATOM 5589 C34 POPC 42 28.414 -1.716 10.813 1.00 0.00 MEMB C

ATOM 5590 H4X POPC 42 27.515 -1.093 10.888 1.00 0.00 MEMB H

ATOM 5591 H4Y POPC 42 28.286 -2.679 10.198 1.00 0.00 MEMB H

ATOM 5592 C35 POPC 42 29.561 -1.057 10.098 1.00 0.00 MEMB C

ATOM 5593 H5X POPC 42 30.245 -1.638 9.507 1.00 0.00 MEMB H

ATOM 5594 H5Y POPC 42 30.225 -0.548 10.808 1.00 0.00 MEMB H

ATOM 5595 C36 POPC 42 29.053 -0.491 8.801 1.00 0.00 MEMB C

ATOM 5596 H6X POPC 42 29.914 0.044 8.365 1.00 0.00 MEMB H

ATOM 5597 H6Y POPC 42 28.279 0.177 9.191 1.00 0.00 MEMB H

ATOM 5598 C37 POPC 42 28.506 -1.568 7.813 1.00 0.00 MEMB C

ATOM 5599 H7X POPC 42 27.423 -1.610 7.983 1.00 0.00 MEMB H

ATOM 5600 H7Y POPC 42 28.889 -2.593 8.013 1.00 0.00 MEMB H

ATOM 5601 C38 POPC 42 28.732 -1.340 6.306 1.00 0.00 MEMB C

ATOM 5602 H8X POPC 42 28.380 -2.213 5.713 1.00 0.00 MEMB H

ATOM 5603 H8Y POPC 42 29.790 -1.103 6.098 1.00 0.00 MEMB H

ATOM 5604 C39 POPC 42 27.950 -0.169 5.775 1.00 0.00 MEMB C

ATOM 5605 H9X POPC 42 28.478 0.680 6.243 1.00 0.00 MEMB H

ATOM 5606 H9Y POPC 42 27.005 -0.349 6.314 1.00 0.00 MEMB H

ATOM 5607 C310 POPC 42 27.992 -0.008 4.220 1.00 0.00 MEMB C

ATOM 5608 H10X POPC 42 27.626 -0.939 3.745 1.00 0.00 MEMB H

ATOM 5609 H10Y POPC 42 29.067 0.155 4.011 1.00 0.00 MEMB H

ATOM 5610 C311 POPC 42 27.401 1.233 3.496 1.00 0.00 MEMB C

ATOM 5611 H11X POPC 42 27.835 2.159 3.862 1.00 0.00 MEMB H

ATOM 5612 H11Y POPC 42 26.371 1.367 3.619 1.00 0.00 MEMB H

ATOM 5613 C312 POPC 42 26.947 1.240 2.035 1.00 0.00 MEMB C

ATOM 5614 H12X POPC 42 27.068 0.188 1.729 1.00 0.00 MEMB H

ATOM 5615 H12Y POPC 42 27.652 1.972 1.609 1.00 0.00 MEMB H

ATOM 5616 C313 POPC 42 25.577 1.900 1.650 1.00 0.00 MEMB C

ATOM 5617 H13X POPC 42 25.191 2.679 2.322 1.00 0.00 MEMB H

ATOM 5618 H13Y POPC 42 24.716 1.253 1.529 1.00 0.00 MEMB H

ATOM 5619 C314 POPC 42 25.556 2.521 0.266 1.00 0.00 MEMB C

ATOM 5620 H14X POPC 42 25.906 3.547 0.293 1.00 0.00 MEMB H

ATOM 5621 H14Y POPC 42 24.487 2.351 0.024 1.00 0.00 MEMB H

ATOM 5622 C315 POPC 42 26.328 1.734 -0.728 1.00 0.00 MEMB C

ATOM 5623 H15X POPC 42 25.665 0.936 -0.421 1.00 0.00 MEMB H

ATOM 5624 H15Y POPC 42 27.347 1.475 -0.395 1.00 0.00 MEMB H

ATOM 5625 C316 POPC 42 26.284 1.874 -2.251 1.00 0.00 MEMB C

ATOM 5626 H16X POPC 42 25.325 1.650 -2.765 1.00 0.00 MEMB H

ATOM 5627 H16Y POPC 42 27.006 1.135 -2.689 1.00 0.00 MEMB H

ATOM 5628 H16Z POPC 42 26.591 2.896 -2.473 1.00 0.00 MEMB H

ATOM 5629 N POPC 43 17.546 21.872 19.557 1.00 0.00 MEMB N

ATOM 5630 C12 POPC 43 19.062 21.875 19.609 1.00 0.00 MEMB C

ATOM 5631 H12A POPC 43 19.447 21.766 18.587 1.00 0.00 MEMB H

ATOM 5632 H12B POPC 43 19.428 21.007 20.149 1.00 0.00 MEMB H

ATOM 5633 C13 POPC 43 17.092 22.854 18.511 1.00 0.00 MEMB C

ATOM 5634 H13A POPC 43 17.666 22.751 17.593 1.00 0.00 MEMB H

ATOM 5635 H13B POPC 43 16.042 22.701 18.290 1.00 0.00 MEMB H

ATOM 5636 H13C POPC 43 17.283 23.874 18.841 1.00 0.00 MEMB H

ATOM 5637 C14 POPC 43 17.127 20.521 19.171 1.00 0.00 MEMB C

ATOM 5638 H14A POPC 43 17.286 20.458 18.113 1.00 0.00 MEMB H

ATOM 5639 H14B POPC 43 17.662 19.735 19.689 1.00 0.00 MEMB H

ATOM 5640 H14C POPC 43 16.061 20.364 19.259 1.00 0.00 MEMB H

ATOM 5641 C15 POPC 43 16.834 22.153 20.838 1.00 0.00 MEMB C

ATOM 5642 H15A POPC 43 16.968 21.346 21.555 1.00 0.00 MEMB H

ATOM 5643 H15B POPC 43 15.770 22.233 20.632 1.00 0.00 MEMB H

ATOM 5644 H15C POPC 43 17.195 23.067 21.289 1.00 0.00 MEMB H

ATOM 5645 C11 POPC 43 19.753 23.115 20.194 1.00 0.00 MEMB C

ATOM 5646 H11A POPC 43 20.833 22.855 20.261 1.00 0.00 MEMB H

ATOM 5647 H11B POPC 43 19.371 23.271 21.223 1.00 0.00 MEMB H

ATOM 5648 P POPC 43 20.474 24.514 18.156 1.00 0.00 MEMB P

ATOM 5649 O13 POPC 43 20.772 23.201 17.561 1.00 0.00 MEMB O

ATOM 5650 O14 POPC 43 21.586 25.383 18.591 1.00 0.00 MEMB O

ATOM 5651 O12 POPC 43 19.541 24.293 19.412 1.00 0.00 MEMB O

ATOM 5652 O11 POPC 43 19.524 25.321 17.197 1.00 0.00 MEMB O

ATOM 5653 C1 POPC 43 19.078 26.592 17.665 1.00 0.00 MEMB C

ATOM 5654 HA POPC 43 19.950 27.256 17.881 1.00 0.00 MEMB H

ATOM 5655 HB POPC 43 18.481 26.483 18.599 1.00 0.00 MEMB H

ATOM 5656 C2 POPC 43 18.183 27.287 16.613 1.00 0.00 MEMB C

ATOM 5657 HS POPC 43 17.478 27.962 17.139 1.00 0.00 MEMB H

ATOM 5658 O21 POPC 43 17.393 26.394 15.881 1.00 0.00 MEMB O

ATOM 5659 C21 POPC 43 16.322 25.972 16.480 1.00 0.00 MEMB C

ATOM 5660 O22 POPC 43 15.957 26.186 17.623 1.00 0.00 MEMB O

ATOM 5661 C22 POPC 43 15.591 25.169 15.444 1.00 0.00 MEMB C

ATOM 5662 H2R POPC 43 14.506 25.175 15.671 1.00 0.00 MEMB H

ATOM 5663 H2S POPC 43 16.002 24.147 15.560 1.00 0.00 MEMB H

ATOM 5664 C3 POPC 43 19.055 28.111 15.628 1.00 0.00 MEMB C

ATOM 5665 HX POPC 43 20.025 27.582 15.497 1.00 0.00 MEMB H

ATOM 5666 HY POPC 43 19.390 29.065 15.996 1.00 0.00 MEMB H

ATOM 5667 O31 POPC 43 18.332 28.457 14.450 1.00 0.00 MEMB O

ATOM 5668 C31 POPC 43 18.685 27.677 13.450 1.00 0.00 MEMB C

ATOM 5669 O32 POPC 43 19.629 26.911 13.445 1.00 0.00 MEMB O

ATOM 5670 C32 POPC 43 17.849 27.969 12.251 1.00 0.00 MEMB C

ATOM 5671 H2X POPC 43 18.369 28.832 11.927 1.00 0.00 MEMB H

ATOM 5672 H2Y POPC 43 16.816 28.309 12.423 1.00 0.00 MEMB H

ATOM 5673 C23 POPC 43 15.929 25.680 14.012 1.00 0.00 MEMB C

ATOM 5674 H3R POPC 43 16.987 25.426 13.760 1.00 0.00 MEMB H

ATOM 5675 H3S POPC 43 15.757 26.770 13.926 1.00 0.00 MEMB H

ATOM 5676 C24 POPC 43 15.162 24.994 12.935 1.00 0.00 MEMB C

ATOM 5677 H4R POPC 43 15.352 25.295 11.888 1.00 0.00 MEMB H

ATOM 5678 H4S POPC 43 14.080 25.156 13.159 1.00 0.00 MEMB H

ATOM 5679 C25 POPC 43 15.507 23.548 13.010 1.00 0.00 MEMB C

ATOM 5680 H5R POPC 43 14.998 23.012 12.234 1.00 0.00 MEMB H

ATOM 5681 H5S POPC 43 15.213 23.235 14.012 1.00 0.00 MEMB H

ATOM 5682 C26 POPC 43 16.802 22.879 12.984 1.00 0.00 MEMB C

ATOM 5683 H6R POPC 43 17.363 23.240 13.871 1.00 0.00 MEMB H

ATOM 5684 H6S POPC 43 17.300 23.054 12.021 1.00 0.00 MEMB H

ATOM 5685 C27 POPC 43 16.572 21.385 13.167 1.00 0.00 MEMB C

ATOM 5686 H7R POPC 43 15.801 20.829 13.741 1.00 0.00 MEMB H

ATOM 5687 H7S POPC 43 17.463 21.249 13.751 1.00 0.00 MEMB H

ATOM 5688 C28 POPC 43 16.575 20.575 11.954 1.00 0.00 MEMB C

ATOM 5689 H8R POPC 43 16.518 19.540 12.397 1.00 0.00 MEMB H

ATOM 5690 H8S POPC 43 17.585 20.637 11.503 1.00 0.00 MEMB H

ATOM 5691 C29 POPC 43 15.426 20.791 10.995 1.00 0.00 MEMB C

ATOM 5692 H91 POPC 43 14.622 20.052 11.112 1.00 0.00 MEMB H

ATOM 5693 C210 POPC 43 15.392 21.610 9.918 1.00 0.00 MEMB C

ATOM 5694 H101 POPC 43 14.495 21.491 9.291 1.00 0.00 MEMB H

ATOM 5695 C211 POPC 43 16.496 22.539 9.396 1.00 0.00 MEMB C

ATOM 5696 H11R POPC 43 17.287 22.578 10.157 1.00 0.00 MEMB H

ATOM 5697 H11S POPC 43 16.096 23.542 9.175 1.00 0.00 MEMB H

ATOM 5698 C212 POPC 43 17.395 22.375 8.165 1.00 0.00 MEMB C

ATOM 5699 H12R POPC 43 17.799 21.410 8.059 1.00 0.00 MEMB H

ATOM 5700 H12S POPC 43 18.405 22.794 8.145 1.00 0.00 MEMB H

ATOM 5701 C213 POPC 43 16.705 22.376 6.890 1.00 0.00 MEMB C

ATOM 5702 H13R POPC 43 16.242 23.325 6.593 1.00 0.00 MEMB H

ATOM 5703 H13S POPC 43 15.873 21.682 7.071 1.00 0.00 MEMB H

ATOM 5704 C214 POPC 43 17.813 21.976 5.967 1.00 0.00 MEMB C

ATOM 5705 H14R POPC 43 18.496 21.116 6.173 1.00 0.00 MEMB H

ATOM 5706 H14S POPC 43 18.431 22.862 5.773 1.00 0.00 MEMB H

ATOM 5707 C215 POPC 43 17.359 21.626 4.696 1.00 0.00 MEMB C

ATOM 5708 H15R POPC 43 16.770 20.685 4.597 1.00 0.00 MEMB H

ATOM 5709 H15S POPC 43 18.332 21.432 4.282 1.00 0.00 MEMB H

ATOM 5710 C216 POPC 43 16.921 22.829 3.948 1.00 0.00 MEMB C

ATOM 5711 H16R POPC 43 17.048 23.759 4.466 1.00 0.00 MEMB H

ATOM 5712 H16S POPC 43 16.003 22.545 4.459 1.00 0.00 MEMB H

ATOM 5713 C217 POPC 43 16.898 23.170 2.373 1.00 0.00 MEMB C

ATOM 5714 H17R POPC 43 16.817 24.237 2.336 1.00 0.00 MEMB H

ATOM 5715 H17S POPC 43 16.051 23.353 1.782 1.00 0.00 MEMB H

ATOM 5716 C218 POPC 43 16.340 22.626 1.029 1.00 0.00 MEMB C

ATOM 5717 H18R POPC 43 17.311 22.313 0.932 1.00 0.00 MEMB H

ATOM 5718 H18S POPC 43 16.294 21.644 0.685 1.00 0.00 MEMB H

ATOM 5719 H18T POPC 43 15.647 21.758 0.939 1.00 0.00 MEMB H

ATOM 5720 C33 POPC 43 18.156 27.069 11.060 1.00 0.00 MEMB C

ATOM 5721 H3X POPC 43 18.199 26.027 11.428 1.00 0.00 MEMB H

ATOM 5722 H3Y POPC 43 19.171 27.351 10.683 1.00 0.00 MEMB H

ATOM 5723 C34 POPC 43 17.094 27.247 9.976 1.00 0.00 MEMB C

ATOM 5724 H4X POPC 43 16.979 28.326 9.745 1.00 0.00 MEMB H

ATOM 5725 H4Y POPC 43 16.134 26.906 10.425 1.00 0.00 MEMB H

ATOM 5726 C35 POPC 43 17.322 26.452 8.677 1.00 0.00 MEMB C

ATOM 5727 H5X POPC 43 16.405 26.578 8.057 1.00 0.00 MEMB H

ATOM 5728 H5Y POPC 43 17.411 25.379 8.948 1.00 0.00 MEMB H

ATOM 5729 C36 POPC 43 18.570 26.852 7.860 1.00 0.00 MEMB C

ATOM 5730 H6X POPC 43 19.396 26.975 8.589 1.00 0.00 MEMB H

ATOM 5731 H6Y POPC 43 18.400 27.838 7.369 1.00 0.00 MEMB H

ATOM 5732 C37 POPC 43 19.034 25.796 6.819 1.00 0.00 MEMB C

ATOM 5733 H7X POPC 43 18.281 25.742 6.005 1.00 0.00 MEMB H

ATOM 5734 H7Y POPC 43 19.090 24.818 7.338 1.00 0.00 MEMB H

ATOM 5735 C38 POPC 43 20.415 26.047 6.173 1.00 0.00 MEMB C

ATOM 5736 H8X POPC 43 21.186 26.145 6.955 1.00 0.00 MEMB H

ATOM 5737 H8Y POPC 43 20.346 26.975 5.572 1.00 0.00 MEMB H

ATOM 5738 C39 POPC 43 21.029 24.951 5.306 1.00 0.00 MEMB C

ATOM 5739 H9X POPC 43 21.250 24.048 5.909 1.00 0.00 MEMB H

ATOM 5740 H9Y POPC 43 21.984 25.325 4.854 1.00 0.00 MEMB H

ATOM 5741 C310 POPC 43 20.179 24.612 4.121 1.00 0.00 MEMB C

ATOM 5742 H10X POPC 43 19.244 24.114 4.446 1.00 0.00 MEMB H

ATOM 5743 H10Y POPC 43 20.768 23.831 3.584 1.00 0.00 MEMB H

ATOM 5744 C311 POPC 43 19.922 25.918 3.320 1.00 0.00 MEMB C

ATOM 5745 H11X POPC 43 20.879 26.413 3.066 1.00 0.00 MEMB H

ATOM 5746 H11Y POPC 43 19.218 26.573 3.865 1.00 0.00 MEMB H

ATOM 5747 C312 POPC 43 19.260 25.652 2.026 1.00 0.00 MEMB C

ATOM 5748 H12X POPC 43 18.358 25.114 2.328 1.00 0.00 MEMB H

ATOM 5749 H12Y POPC 43 20.092 25.057 1.569 1.00 0.00 MEMB H

ATOM 5750 C313 POPC 43 18.928 26.555 0.901 1.00 0.00 MEMB C

ATOM 5751 H13X POPC 43 18.382 27.433 1.271 1.00 0.00 MEMB H

ATOM 5752 H13Y POPC 43 18.184 25.999 0.261 1.00 0.00 MEMB H

ATOM 5753 C314 POPC 43 20.226 26.779 0.098 1.00 0.00 MEMB C

ATOM 5754 H14X POPC 43 20.842 25.859 -0.044 1.00 0.00 MEMB H

ATOM 5755 H14Y POPC 43 20.837 27.489 0.683 1.00 0.00 MEMB H

ATOM 5756 C315 POPC 43 19.959 27.222 -1.305 1.00 0.00 MEMB C

ATOM 5757 H15X POPC 43 19.868 26.395 -2.046 1.00 0.00 MEMB H

ATOM 5758 H15Y POPC 43 20.770 27.912 -1.641 1.00 0.00 MEMB H

ATOM 5759 C316 POPC 43 18.621 27.857 -1.338 1.00 0.00 MEMB C

ATOM 5760 H16X POPC 43 18.344 28.469 -0.476 1.00 0.00 MEMB H

ATOM 5761 H16Y POPC 43 17.807 27.161 -1.382 1.00 0.00 MEMB H

ATOM 5762 H16Z POPC 43 18.639 28.437 -2.257 1.00 0.00 MEMB H

ATOM 5763 N POPC 44 26.666 16.352 20.638 1.00 0.00 MEMB N

ATOM 5764 C12 POPC 44 26.952 16.840 19.209 1.00 0.00 MEMB C

ATOM 5765 H12A POPC 44 26.996 15.993 18.539 1.00 0.00 MEMB H

ATOM 5766 H12B POPC 44 27.917 17.334 19.190 1.00 0.00 MEMB H

ATOM 5767 C13 POPC 44 25.414 15.524 20.657 1.00 0.00 MEMB C

ATOM 5768 H13A POPC 44 24.903 15.557 21.613 1.00 0.00 MEMB H

ATOM 5769 H13B POPC 44 24.712 15.903 19.907 1.00 0.00 MEMB H

ATOM 5770 H13C POPC 44 25.618 14.488 20.425 1.00 0.00 MEMB H

ATOM 5771 C14 POPC 44 27.748 15.510 21.185 1.00 0.00 MEMB C

ATOM 5772 H14A POPC 44 28.643 16.086 21.395 1.00 0.00 MEMB H

ATOM 5773 H14B POPC 44 27.436 15.062 22.124 1.00 0.00 MEMB H

ATOM 5774 H14C POPC 44 27.957 14.711 20.497 1.00 0.00 MEMB H

ATOM 5775 C15 POPC 44 26.538 17.530 21.543 1.00 0.00 MEMB C

ATOM 5776 H15A POPC 44 27.453 18.122 21.559 1.00 0.00 MEMB H

ATOM 5777 H15B POPC 44 26.307 17.242 22.563 1.00 0.00 MEMB H

ATOM 5778 H15C POPC 44 25.736 18.155 21.159 1.00 0.00 MEMB H

ATOM 5779 C11 POPC 44 25.980 17.844 18.505 1.00 0.00 MEMB C

ATOM 5780 H11A POPC 44 26.322 17.877 17.438 1.00 0.00 MEMB H

ATOM 5781 H11B POPC 44 26.116 18.865 18.923 1.00 0.00 MEMB H

ATOM 5782 P POPC 44 23.614 17.665 17.359 1.00 0.00 MEMB P

ATOM 5783 O13 POPC 44 24.118 18.742 16.474 1.00 0.00 MEMB O

ATOM 5784 O14 POPC 44 22.221 17.720 17.882 1.00 0.00 MEMB O

ATOM 5785 O12 POPC 44 24.607 17.475 18.609 1.00 0.00 MEMB O

ATOM 5786 O11 POPC 44 23.803 16.283 16.616 1.00 0.00 MEMB O

ATOM 5787 C1 POPC 44 23.359 15.098 17.281 1.00 0.00 MEMB C

ATOM 5788 HA POPC 44 22.746 15.320 18.170 1.00 0.00 MEMB H

ATOM 5789 HB POPC 44 24.255 14.539 17.619 1.00 0.00 MEMB H

ATOM 5790 C2 POPC 44 22.502 14.140 16.411 1.00 0.00 MEMB C

ATOM 5791 HS POPC 44 22.334 13.196 16.973 1.00 0.00 MEMB H

ATOM 5792 O21 POPC 44 23.271 13.930 15.179 1.00 0.00 MEMB O

ATOM 5793 C21 POPC 44 22.775 13.049 14.316 1.00 0.00 MEMB C

ATOM 5794 O22 POPC 44 21.649 12.591 14.413 1.00 0.00 MEMB O

ATOM 5795 C22 POPC 44 23.705 12.681 13.119 1.00 0.00 MEMB C

ATOM 5796 H2R POPC 44 24.500 11.973 13.285 1.00 0.00 MEMB H

ATOM 5797 H2S POPC 44 24.248 13.618 12.847 1.00 0.00 MEMB H

ATOM 5798 C3 POPC 44 21.025 14.685 16.181 1.00 0.00 MEMB C

ATOM 5799 HX POPC 44 20.559 14.953 17.142 1.00 0.00 MEMB H

ATOM 5800 HY POPC 44 20.428 13.895 15.699 1.00 0.00 MEMB H

ATOM 5801 O31 POPC 44 20.827 15.930 15.576 1.00 0.00 MEMB O

ATOM 5802 C31 POPC 44 19.975 16.008 14.447 1.00 0.00 MEMB C

ATOM 5803 O32 POPC 44 19.433 14.996 14.082 1.00 0.00 MEMB O

ATOM 5804 C32 POPC 44 19.897 17.552 13.618 1.00 0.00 MEMB C

ATOM 5805 H2X POPC 44 20.145 17.661 14.774 1.00 0.00 MEMB H

ATOM 5806 H2Y POPC 44 19.028 17.942 13.297 1.00 0.00 MEMB H

ATOM 5807 C23 POPC 44 22.806 12.058 11.999 1.00 0.00 MEMB C

ATOM 5808 H3R POPC 44 21.872 12.624 12.173 1.00 0.00 MEMB H

ATOM 5809 H3S POPC 44 22.151 11.228 12.169 1.00 0.00 MEMB H

ATOM 5810 C24 POPC 44 23.428 11.922 10.536 1.00 0.00 MEMB C

ATOM 5811 H4R POPC 44 24.132 11.088 10.591 1.00 0.00 MEMB H

ATOM 5812 H4S POPC 44 24.018 12.850 10.336 1.00 0.00 MEMB H

ATOM 5813 C25 POPC 44 22.441 11.656 9.316 1.00 0.00 MEMB C

ATOM 5814 H5R POPC 44 22.165 12.631 8.901 1.00 0.00 MEMB H

ATOM 5815 H5S POPC 44 21.488 11.187 9.609 1.00 0.00 MEMB H

ATOM 5816 C26 POPC 44 22.837 11.406 7.876 1.00 0.00 MEMB C

ATOM 5817 H6R POPC 44 22.645 10.335 7.700 1.00 0.00 MEMB H

ATOM 5818 H6S POPC 44 23.903 11.701 7.764 1.00 0.00 MEMB H

ATOM 5819 C27 POPC 44 22.033 12.232 6.828 1.00 0.00 MEMB C

ATOM 5820 H7R POPC 44 22.224 13.326 6.951 1.00 0.00 MEMB H

ATOM 5821 H7S POPC 44 20.930 12.086 6.850 1.00 0.00 MEMB H

ATOM 5822 C28 POPC 44 22.563 11.855 5.489 1.00 0.00 MEMB C

ATOM 5823 H8R POPC 44 22.041 10.942 5.110 1.00 0.00 MEMB H

ATOM 5824 H8S POPC 44 23.619 11.581 5.646 1.00 0.00 MEMB H

ATOM 5825 C29 POPC 44 22.449 13.058 4.614 1.00 0.00 MEMB C

ATOM 5826 H91 POPC 44 21.450 13.150 4.145 1.00 0.00 MEMB H

ATOM 5827 C210 POPC 44 23.442 13.886 4.232 1.00 0.00 MEMB C

ATOM 5828 H101 POPC 44 23.254 14.430 3.284 1.00 0.00 MEMB H

ATOM 5829 C211 POPC 44 24.911 13.815 4.608 1.00 0.00 MEMB C

ATOM 5830 H11R POPC 44 24.921 14.000 5.686 1.00 0.00 MEMB H

ATOM 5831 H11S POPC 44 25.431 14.610 4.028 1.00 0.00 MEMB H

ATOM 5832 C212 POPC 44 25.859 12.606 4.486 1.00 0.00 MEMB C

ATOM 5833 H12R POPC 44 25.466 11.720 5.041 1.00 0.00 MEMB H

ATOM 5834 H12S POPC 44 26.946 12.713 4.632 1.00 0.00 MEMB H

ATOM 5835 C213 POPC 44 25.835 12.246 3.097 1.00 0.00 MEMB C

ATOM 5836 H13R POPC 44 24.753 12.040 3.183 1.00 0.00 MEMB H

ATOM 5837 H13S POPC 44 26.436 11.350 2.824 1.00 0.00 MEMB H

ATOM 5838 C214 POPC 44 26.260 13.169 2.023 1.00 0.00 MEMB C

ATOM 5839 H14R POPC 44 25.814 14.158 1.865 1.00 0.00 MEMB H

ATOM 5840 H14S POPC 44 25.794 12.636 1.243 1.00 0.00 MEMB H

ATOM 5841 C215 POPC 44 27.704 12.831 1.746 1.00 0.00 MEMB C

ATOM 5842 H15R POPC 44 27.810 11.876 1.202 1.00 0.00 MEMB H

ATOM 5843 H15S POPC 44 28.193 12.687 2.717 1.00 0.00 MEMB H

ATOM 5844 C216 POPC 44 28.454 13.854 0.998 1.00 0.00 MEMB C

ATOM 5845 H16R POPC 44 29.478 13.449 0.955 1.00 0.00 MEMB H

ATOM 5846 H16S POPC 44 28.277 14.814 1.513 1.00 0.00 MEMB H

ATOM 5847 C217 POPC 44 27.995 14.044 -0.370 1.00 0.00 MEMB C

ATOM 5848 H17R POPC 44 28.196 14.902 -1.032 1.00 0.00 MEMB H

ATOM 5849 H17S POPC 44 26.917 14.147 -0.187 1.00 0.00 MEMB H

ATOM 5850 C218 POPC 44 28.557 13.057 -1.304 1.00 0.00 MEMB C

ATOM 5851 H18R POPC 44 28.328 12.006 -1.164 1.00 0.00 MEMB H

ATOM 5852 H18S POPC 44 29.626 13.017 -1.238 1.00 0.00 MEMB H

ATOM 5853 H18T POPC 44 28.256 13.365 -2.326 1.00 0.00 MEMB H

ATOM 5854 C33 POPC 44 20.915 18.653 12.264 1.00 0.00 MEMB C

ATOM 5855 H3X POPC 44 22.043 18.846 12.488 1.00 0.00 MEMB H

ATOM 5856 H3Y POPC 44 21.806 19.407 12.268 1.00 0.00 MEMB H

ATOM 5857 C34 POPC 44 21.011 18.133 10.856 1.00 0.00 MEMB C

ATOM 5858 H4X POPC 44 20.209 18.746 10.535 1.00 0.00 MEMB H

ATOM 5859 H4Y POPC 44 20.550 17.150 10.847 1.00 0.00 MEMB H

ATOM 5860 C35 POPC 44 22.242 18.158 9.845 1.00 0.00 MEMB C

ATOM 5861 H5X POPC 44 22.969 17.317 9.734 1.00 0.00 MEMB H

ATOM 5862 H5Y POPC 44 23.115 18.777 10.051 1.00 0.00 MEMB H

ATOM 5863 C36 POPC 44 21.784 17.631 8.518 1.00 0.00 MEMB C

ATOM 5864 H6X POPC 44 20.746 17.960 8.351 1.00 0.00 MEMB H

ATOM 5865 H6Y POPC 44 21.769 16.539 8.443 1.00 0.00 MEMB H

ATOM 5866 C37 POPC 44 22.886 17.661 7.511 1.00 0.00 MEMB C

ATOM 5867 H7X POPC 44 23.657 16.862 7.708 1.00 0.00 MEMB H

ATOM 5868 H7Y POPC 44 23.368 18.641 7.655 1.00 0.00 MEMB H

ATOM 5869 C38 POPC 44 22.226 17.369 6.199 1.00 0.00 MEMB C

ATOM 5870 H8X POPC 44 21.342 18.039 6.108 1.00 0.00 MEMB H

ATOM 5871 H8Y POPC 44 21.860 16.312 6.207 1.00 0.00 MEMB H

ATOM 5872 C39 POPC 44 23.215 17.562 5.084 1.00 0.00 MEMB C

ATOM 5873 H9X POPC 44 24.038 16.823 5.226 1.00 0.00 MEMB H

ATOM 5874 H9Y POPC 44 23.646 18.585 5.146 1.00 0.00 MEMB H

ATOM 5875 C310 POPC 44 22.544 17.349 3.747 1.00 0.00 MEMB C

ATOM 5876 H10X POPC 44 21.837 18.186 3.540 1.00 0.00 MEMB H

ATOM 5877 H10Y POPC 44 21.939 16.414 3.799 1.00 0.00 MEMB H

ATOM 5878 C311 POPC 44 23.579 17.210 2.644 1.00 0.00 MEMB C

ATOM 5879 H11X POPC 44 24.121 16.246 2.730 1.00 0.00 MEMB H

ATOM 5880 H11Y POPC 44 24.336 18.028 2.672 1.00 0.00 MEMB H

ATOM 5881 C312 POPC 44 22.901 17.250 1.315 1.00 0.00 MEMB C

ATOM 5882 H12X POPC 44 22.530 18.289 1.155 1.00 0.00 MEMB H

ATOM 5883 H12Y POPC 44 22.006 16.593 1.258 1.00 0.00 MEMB H

ATOM 5884 C313 POPC 44 23.835 16.887 0.200 1.00 0.00 MEMB C

ATOM 5885 H13X POPC 44 23.951 15.785 0.095 1.00 0.00 MEMB H

ATOM 5886 H13Y POPC 44 24.850 17.347 0.257 1.00 0.00 MEMB H

ATOM 5887 C314 POPC 44 23.076 17.444 -0.939 1.00 0.00 MEMB C

ATOM 5888 H14X POPC 44 22.469 18.339 -0.758 1.00 0.00 MEMB H

ATOM 5889 H14Y POPC 44 22.220 16.812 -1.158 1.00 0.00 MEMB H

ATOM 5890 C315 POPC 44 23.902 17.942 -2.024 1.00 0.00 MEMB C

ATOM 5891 H15X POPC 44 24.835 18.600 -1.878 1.00 0.00 MEMB H

ATOM 5892 H15Y POPC 44 23.151 18.140 -2.793 1.00 0.00 MEMB H

ATOM 5893 C316 POPC 44 24.478 17.096 -2.742 1.00 0.00 MEMB C

ATOM 5894 H16X POPC 44 24.606 17.366 -3.775 1.00 0.00 MEMB H

ATOM 5895 H16Y POPC 44 24.078 16.409 -3.380 1.00 0.00 MEMB H

ATOM 5896 H16Z POPC 44 25.179 16.655 -2.054 1.00 0.00 MEMB H

ATOM 5897 N POPC 45 -30.602 -2.596 20.810 1.00 0.00 MEMB N

ATOM 5898 C12 POPC 45 -31.911 -2.608 20.069 1.00 0.00 MEMB C

ATOM 5899 H12A POPC 45 -32.348 -1.606 20.021 1.00 0.00 MEMB H

ATOM 5900 H12B POPC 45 -32.633 -3.212 20.602 1.00 0.00 MEMB H

ATOM 5901 C13 POPC 45 -29.550 -1.961 19.947 1.00 0.00 MEMB C

ATOM 5902 H13A POPC 45 -29.765 -0.909 19.791 1.00 0.00 MEMB H

ATOM 5903 H13B POPC 45 -28.564 -2.079 20.371 1.00 0.00 MEMB H

ATOM 5904 H13C POPC 45 -29.539 -2.440 18.966 1.00 0.00 MEMB H

ATOM 5905 C14 POPC 45 -30.794 -1.805 22.040 1.00 0.00 MEMB C

ATOM 5906 H14A POPC 45 -31.606 -2.231 22.626 1.00 0.00 MEMB H

ATOM 5907 H14B POPC 45 -29.883 -1.787 22.617 1.00 0.00 MEMB H

ATOM 5908 H14C POPC 45 -31.056 -0.789 21.772 1.00 0.00 MEMB H

ATOM 5909 C15 POPC 45 -30.202 -3.984 21.213 1.00 0.00 MEMB C

ATOM 5910 H15A POPC 45 -30.989 -4.450 21.790 1.00 0.00 MEMB H

ATOM 5911 H15B POPC 45 -29.287 -3.958 21.785 1.00 0.00 MEMB H

ATOM 5912 H15C POPC 45 -30.051 -4.595 20.324 1.00 0.00 MEMB H

ATOM 5913 C11 POPC 45 -31.791 -3.152 18.649 1.00 0.00 MEMB C

ATOM 5914 H11A POPC 45 -31.240 -2.446 17.974 1.00 0.00 MEMB H

ATOM 5915 H11B POPC 45 -32.783 -3.218 18.262 1.00 0.00 MEMB H

ATOM 5916 P POPC 45 -31.486 -5.262 17.215 1.00 0.00 MEMB P

ATOM 5917 O13 POPC 45 -32.850 -5.175 16.658 1.00 0.00 MEMB O

ATOM 5918 O14 POPC 45 -30.917 -6.597 17.443 1.00 0.00 MEMB O

ATOM 5919 O12 POPC 45 -31.355 -4.499 18.627 1.00 0.00 MEMB O

ATOM 5920 O11 POPC 45 -30.485 -4.448 16.330 1.00 0.00 MEMB O

ATOM 5921 C1 POPC 45 -29.190 -4.242 16.916 1.00 0.00 MEMB C

ATOM 5922 HA POPC 45 -28.838 -4.999 17.630 1.00 0.00 MEMB H

ATOM 5923 HB POPC 45 -29.340 -3.361 17.563 1.00 0.00 MEMB H

ATOM 5924 C2 POPC 45 -28.086 -4.016 15.818 1.00 0.00 MEMB C

ATOM 5925 HS POPC 45 -27.212 -3.479 16.256 1.00 0.00 MEMB H

ATOM 5926 O21 POPC 45 -28.566 -3.251 14.775 1.00 0.00 MEMB O

ATOM 5927 C21 POPC 45 -29.142 -2.249 15.271 1.00 0.00 MEMB C

ATOM 5928 O22 POPC 45 -28.879 -1.698 16.343 1.00 0.00 MEMB O

ATOM 5929 C22 POPC 45 -29.682 -1.666 13.977 1.00 0.00 MEMB C

ATOM 5930 H2R POPC 45 -29.576 -2.266 13.083 1.00 0.00 MEMB H

ATOM 5931 H2S POPC 45 -28.676 -1.285 13.845 1.00 0.00 MEMB H

ATOM 5932 C3 POPC 45 -27.570 -5.230 15.088 1.00 0.00 MEMB C

ATOM 5933 HX POPC 45 -27.046 -5.839 15.839 1.00 0.00 MEMB H

ATOM 5934 HY POPC 45 -26.885 -4.874 14.273 1.00 0.00 MEMB H

ATOM 5935 O31 POPC 45 -28.691 -5.930 14.558 1.00 0.00 MEMB O

ATOM 5936 C31 POPC 45 -28.471 -6.762 13.588 1.00 0.00 MEMB C

ATOM 5937 O32 POPC 45 -27.413 -6.937 12.999 1.00 0.00 MEMB O

ATOM 5938 C32 POPC 45 -29.752 -7.572 13.437 1.00 0.00 MEMB C

ATOM 5939 H2X POPC 45 -30.634 -6.978 13.753 1.00 0.00 MEMB H

ATOM 5940 H2Y POPC 45 -29.618 -8.412 14.155 1.00 0.00 MEMB H

ATOM 5941 C23 POPC 45 -30.980 -0.756 13.963 1.00 0.00 MEMB C

ATOM 5942 H3R POPC 45 -31.388 -0.129 13.156 1.00 0.00 MEMB H

ATOM 5943 H3S POPC 45 -31.112 0.236 14.359 1.00 0.00 MEMB H

ATOM 5944 C24 POPC 45 -31.846 -1.740 14.272 1.00 0.00 MEMB C

ATOM 5945 H4R POPC 45 -32.822 -1.454 14.572 1.00 0.00 MEMB H

ATOM 5946 H4S POPC 45 -31.497 -2.320 15.144 1.00 0.00 MEMB H

ATOM 5947 C25 POPC 45 -31.971 -2.332 12.932 1.00 0.00 MEMB C

ATOM 5948 H5R POPC 45 -31.156 -2.896 12.449 1.00 0.00 MEMB H

ATOM 5949 H5S POPC 45 -32.483 -1.627 12.215 1.00 0.00 MEMB H

ATOM 5950 C26 POPC 45 -32.871 -3.311 13.279 1.00 0.00 MEMB C

ATOM 5951 H6R POPC 45 -33.695 -2.589 13.408 1.00 0.00 MEMB H

ATOM 5952 H6S POPC 45 -32.607 -3.820 14.262 1.00 0.00 MEMB H

ATOM 5953 C27 POPC 45 -33.098 -4.064 12.047 1.00 0.00 MEMB C

ATOM 5954 H7R POPC 45 -33.447 -4.770 12.828 1.00 0.00 MEMB H

ATOM 5955 H7S POPC 45 -32.628 -4.992 11.766 1.00 0.00 MEMB H

ATOM 5956 C28 POPC 45 -34.010 -3.188 10.996 1.00 0.00 MEMB C

ATOM 5957 H8R POPC 45 -33.245 -2.588 10.504 1.00 0.00 MEMB H

ATOM 5958 H8S POPC 45 -34.611 -2.418 11.517 1.00 0.00 MEMB H

ATOM 5959 C29 POPC 45 -34.950 -3.609 9.867 1.00 0.00 MEMB C

ATOM 5960 H91 POPC 45 -36.003 -3.843 9.963 1.00 0.00 MEMB H

ATOM 5961 C210 POPC 45 -34.827 -3.692 8.597 1.00 0.00 MEMB C

ATOM 5962 H101 POPC 45 -35.460 -4.402 8.033 1.00 0.00 MEMB H

ATOM 5963 C211 POPC 45 -33.651 -3.357 7.951 1.00 0.00 MEMB C

ATOM 5964 H11R POPC 45 -32.927 -4.092 8.343 1.00 0.00 MEMB H

ATOM 5965 H11S POPC 45 -33.516 -2.322 8.325 1.00 0.00 MEMB H

ATOM 5966 C212 POPC 45 -34.369 -3.587 6.616 1.00 0.00 MEMB C

ATOM 5967 H12R POPC 45 -34.044 -2.745 6.135 1.00 0.00 MEMB H

ATOM 5968 H12S POPC 45 -35.466 -3.409 6.407 1.00 0.00 MEMB H

ATOM 5969 C213 POPC 45 -33.948 -4.832 5.882 1.00 0.00 MEMB C

ATOM 5970 H13R POPC 45 -34.339 -4.698 4.849 1.00 0.00 MEMB H

ATOM 5971 H13S POPC 45 -34.430 -5.727 6.333 1.00 0.00 MEMB H

ATOM 5972 C214 POPC 45 -32.432 -4.982 5.838 1.00 0.00 MEMB C

ATOM 5973 H14R POPC 45 -31.982 -5.045 6.852 1.00 0.00 MEMB H

ATOM 5974 H14S POPC 45 -32.012 -4.072 5.351 1.00 0.00 MEMB H

ATOM 5975 C215 POPC 45 -32.039 -6.232 5.057 1.00 0.00 MEMB C

ATOM 5976 H15R POPC 45 -32.495 -7.118 5.560 1.00 0.00 MEMB H

ATOM 5977 H15S POPC 45 -30.935 -6.334 5.097 1.00 0.00 MEMB H

ATOM 5978 C216 POPC 45 -32.546 -6.190 3.613 1.00 0.00 MEMB C

ATOM 5979 H16R POPC 45 -32.749 -5.146 3.294 1.00 0.00 MEMB H

ATOM 5980 H16S POPC 45 -33.539 -6.690 3.624 1.00 0.00 MEMB H

ATOM 5981 C217 POPC 45 -31.636 -6.867 2.577 1.00 0.00 MEMB C

ATOM 5982 H17R POPC 45 -32.276 -7.094 1.733 1.00 0.00 MEMB H

ATOM 5983 H17S POPC 45 -31.230 -7.833 2.937 1.00 0.00 MEMB H

ATOM 5984 C218 POPC 45 -30.539 -6.014 1.947 1.00 0.00 MEMB C

ATOM 5985 H18R POPC 45 -29.660 -5.964 2.618 1.00 0.00 MEMB H

ATOM 5986 H18S POPC 45 -30.923 -4.990 1.753 1.00 0.00 MEMB H

ATOM 5987 H18T POPC 45 -30.211 -6.449 0.975 1.00 0.00 MEMB H

ATOM 5988 C33 POPC 45 -29.946 -8.157 12.034 1.00 0.00 MEMB C

ATOM 5989 H3X POPC 45 -30.622 -9.038 12.141 1.00 0.00 MEMB H

ATOM 5990 H3Y POPC 45 -28.952 -8.467 11.652 1.00 0.00 MEMB H

ATOM 5991 C34 POPC 45 -30.554 -7.238 10.993 1.00 0.00 MEMB C

ATOM 5992 H4X POPC 45 -31.578 -6.947 11.302 1.00 0.00 MEMB H

ATOM 5993 H4Y POPC 45 -30.607 -7.768 10.009 1.00 0.00 MEMB H

ATOM 5994 C35 POPC 45 -29.761 -5.980 10.758 1.00 0.00 MEMB C

ATOM 5995 H5X POPC 45 -28.659 -6.174 10.754 1.00 0.00 MEMB H

ATOM 5996 H5Y POPC 45 -29.947 -5.246 11.579 1.00 0.00 MEMB H

ATOM 5997 C36 POPC 45 -30.198 -5.399 9.416 1.00 0.00 MEMB C

ATOM 5998 H6X POPC 45 -31.291 -5.190 9.381 1.00 0.00 MEMB H

ATOM 5999 H6Y POPC 45 -29.851 -6.007 8.556 1.00 0.00 MEMB H

ATOM 6000 C37 POPC 45 -29.578 -4.071 9.327 1.00 0.00 MEMB C

ATOM 6001 H7X POPC 45 -28.668 -4.208 9.925 1.00 0.00 MEMB H

ATOM 6002 H7Y POPC 45 -30.222 -3.469 9.996 1.00 0.00 MEMB H

ATOM 6003 C38 POPC 45 -29.450 -3.458 7.912 1.00 0.00 MEMB C

ATOM 6004 H8X POPC 45 -29.024 -2.428 8.041 1.00 0.00 MEMB H

ATOM 6005 H8Y POPC 45 -30.440 -3.394 7.431 1.00 0.00 MEMB H

ATOM 6006 C39 POPC 45 -28.343 -3.851 6.971 1.00 0.00 MEMB C

ATOM 6007 H9X POPC 45 -28.454 -4.854 6.551 1.00 0.00 MEMB H

ATOM 6008 H9Y POPC 45 -27.583 -4.001 7.729 1.00 0.00 MEMB H

ATOM 6009 C310 POPC 45 -28.051 -2.742 5.913 1.00 0.00 MEMB C

ATOM 6010 H10X POPC 45 -27.050 -2.857 5.447 1.00 0.00 MEMB H

ATOM 6011 H10Y POPC 45 -28.076 -1.689 6.276 1.00 0.00 MEMB H

ATOM 6012 C311 POPC 45 -29.125 -2.804 4.852 1.00 0.00 MEMB C

ATOM 6013 H11X POPC 45 -30.095 -2.472 5.294 1.00 0.00 MEMB H

ATOM 6014 H11Y POPC 45 -29.228 -3.862 4.530 1.00 0.00 MEMB H

ATOM 6015 C312 POPC 45 -28.825 -2.017 3.600 1.00 0.00 MEMB C

ATOM 6016 H12X POPC 45 -27.798 -2.168 3.211 1.00 0.00 MEMB H

ATOM 6017 H12Y POPC 45 -28.946 -0.925 3.819 1.00 0.00 MEMB H

ATOM 6018 C313 POPC 45 -29.784 -2.520 2.537 1.00 0.00 MEMB C

ATOM 6019 H13X POPC 45 -30.745 -2.720 3.040 1.00 0.00 MEMB H

ATOM 6020 H13Y POPC 45 -29.512 -3.431 1.976 1.00 0.00 MEMB H

ATOM 6021 C314 POPC 45 -29.779 -1.523 1.456 1.00 0.00 MEMB C

ATOM 6022 H14X POPC 45 -29.643 -0.571 2.022 1.00 0.00 MEMB H

ATOM 6023 H14Y POPC 45 -30.703 -1.531 0.821 1.00 0.00 MEMB H

ATOM 6024 C315 POPC 45 -28.660 -1.797 0.529 1.00 0.00 MEMB C

ATOM 6025 H15X POPC 45 -27.699 -1.636 0.995 1.00 0.00 MEMB H

ATOM 6026 H15Y POPC 45 -28.180 -1.074 -0.090 1.00 0.00 MEMB H

ATOM 6027 C316 POPC 45 -29.167 -2.253 -0.775 1.00 0.00 MEMB C

ATOM 6028 H16X POPC 45 -29.822 -1.511 -1.285 1.00 0.00 MEMB H

ATOM 6029 H16Y POPC 45 -29.591 -3.245 -0.670 1.00 0.00 MEMB H

ATOM 6030 H16Z POPC 45 -28.453 -2.288 -1.574 1.00 0.00 MEMB H

ATOM 6031 N POPC 46 13.068 28.375 20.902 1.00 0.00 MEMB N

ATOM 6032 C12 POPC 46 13.570 27.872 19.549 1.00 0.00 MEMB C

ATOM 6033 H12A POPC 46 12.866 27.140 19.181 1.00 0.00 MEMB H

ATOM 6034 H12B POPC 46 14.530 27.388 19.679 1.00 0.00 MEMB H

ATOM 6035 C13 POPC 46 13.833 29.589 21.331 1.00 0.00 MEMB C

ATOM 6036 H13A POPC 46 14.860 29.360 21.529 1.00 0.00 MEMB H

ATOM 6037 H13B POPC 46 13.805 30.317 20.521 1.00 0.00 MEMB H

ATOM 6038 H13C POPC 46 13.420 30.017 22.238 1.00 0.00 MEMB H

ATOM 6039 C14 POPC 46 11.597 28.696 20.833 1.00 0.00 MEMB C

ATOM 6040 H14A POPC 46 11.281 29.337 21.656 1.00 0.00 MEMB H

ATOM 6041 H14B POPC 46 11.368 29.205 19.897 1.00 0.00 MEMB H

ATOM 6042 H14C POPC 46 10.998 27.800 20.870 1.00 0.00 MEMB H

ATOM 6043 C15 POPC 46 13.248 27.295 21.902 1.00 0.00 MEMB C

ATOM 6044 H15A POPC 46 13.032 27.660 22.906 1.00 0.00 MEMB H

ATOM 6045 H15B POPC 46 14.262 26.911 21.907 1.00 0.00 MEMB H

ATOM 6046 H15C POPC 46 12.570 26.488 21.669 1.00 0.00 MEMB H

ATOM 6047 C11 POPC 46 13.810 28.876 18.402 1.00 0.00 MEMB C

ATOM 6048 H11A POPC 46 14.235 28.327 17.529 1.00 0.00 MEMB H

ATOM 6049 H11B POPC 46 14.581 29.614 18.728 1.00 0.00 MEMB H

ATOM 6050 P POPC 46 12.752 30.761 17.071 1.00 0.00 MEMB P

ATOM 6051 O13 POPC 46 12.899 30.360 15.655 1.00 0.00 MEMB O

ATOM 6052 O14 POPC 46 13.745 31.655 17.688 1.00 0.00 MEMB O

ATOM 6053 O12 POPC 46 12.596 29.495 18.010 1.00 0.00 MEMB O

ATOM 6054 O11 POPC 46 11.332 31.362 17.384 1.00 0.00 MEMB O

ATOM 6055 C1 POPC 46 10.218 30.554 17.037 1.00 0.00 MEMB C

ATOM 6056 HA POPC 46 9.375 30.840 17.707 1.00 0.00 MEMB H

ATOM 6057 HB POPC 46 10.355 29.455 17.143 1.00 0.00 MEMB H

ATOM 6058 C2 POPC 46 9.744 30.893 15.632 1.00 0.00 MEMB C

ATOM 6059 HS POPC 46 8.710 30.553 15.671 1.00 0.00 MEMB H

ATOM 6060 O21 POPC 46 10.389 30.241 14.548 1.00 0.00 MEMB O

ATOM 6061 C21 POPC 46 9.917 29.058 14.254 1.00 0.00 MEMB C

ATOM 6062 O22 POPC 46 8.921 28.542 14.730 1.00 0.00 MEMB O

ATOM 6063 C22 POPC 46 10.803 28.434 13.199 1.00 0.00 MEMB C

ATOM 6064 H2R POPC 46 10.439 27.408 12.991 1.00 0.00 MEMB H

ATOM 6065 H2S POPC 46 11.827 28.420 13.619 1.00 0.00 MEMB H

ATOM 6066 C3 POPC 46 9.856 32.415 15.402 1.00 0.00 MEMB C

ATOM 6067 HX POPC 46 9.566 32.995 16.291 1.00 0.00 MEMB H

ATOM 6068 HY POPC 46 9.135 32.585 14.599 1.00 0.00 MEMB H

ATOM 6069 O31 POPC 46 11.086 32.906 15.070 1.00 0.00 MEMB O

ATOM 6070 C31 POPC 46 11.308 32.804 13.826 1.00 0.00 MEMB C

ATOM 6071 O32 POPC 46 10.504 32.794 12.903 1.00 0.00 MEMB O

ATOM 6072 C32 POPC 46 12.802 32.798 13.758 1.00 0.00 MEMB C

ATOM 6073 H2X POPC 46 13.030 31.935 13.106 1.00 0.00 MEMB H

ATOM 6074 H2Y POPC 46 13.219 32.760 14.759 1.00 0.00 MEMB H

ATOM 6075 C23 POPC 46 10.834 29.289 11.936 1.00 0.00 MEMB C

ATOM 6076 H3R POPC 46 11.666 28.984 11.268 1.00 0.00 MEMB H

ATOM 6077 H3S POPC 46 10.975 30.368 12.187 1.00 0.00 MEMB H

ATOM 6078 C24 POPC 46 9.574 29.134 11.144 1.00 0.00 MEMB C

ATOM 6079 H4R POPC 46 8.701 29.154 11.846 1.00 0.00 MEMB H

ATOM 6080 H4S POPC 46 9.520 28.143 10.636 1.00 0.00 MEMB H

ATOM 6081 C25 POPC 46 9.514 30.288 10.143 1.00 0.00 MEMB C

ATOM 6082 H5R POPC 46 10.317 30.412 9.407 1.00 0.00 MEMB H

ATOM 6083 H5S POPC 46 9.415 31.217 10.721 1.00 0.00 MEMB H

ATOM 6084 C26 POPC 46 8.287 30.024 9.400 1.00 0.00 MEMB C

ATOM 6085 H6R POPC 46 7.656 30.178 10.289 1.00 0.00 MEMB H

ATOM 6086 H6S POPC 46 8.313 28.946 9.173 1.00 0.00 MEMB H

ATOM 6087 C27 POPC 46 7.663 31.001 8.413 1.00 0.00 MEMB C

ATOM 6088 H7R POPC 46 7.201 31.548 9.182 1.00 0.00 MEMB H

ATOM 6089 H7S POPC 46 6.615 31.217 8.148 1.00 0.00 MEMB H

ATOM 6090 C28 POPC 46 8.484 31.575 7.356 1.00 0.00 MEMB C

ATOM 6091 H8R POPC 46 9.404 31.980 7.768 1.00 0.00 MEMB H

ATOM 6092 H8S POPC 46 7.827 32.367 6.989 1.00 0.00 MEMB H

ATOM 6093 C29 POPC 46 8.750 30.548 6.355 1.00 0.00 MEMB C

ATOM 6094 H91 POPC 46 9.270 29.649 6.698 1.00 0.00 MEMB H

ATOM 6095 C210 POPC 46 8.119 30.614 5.206 1.00 0.00 MEMB C

ATOM 6096 H101 POPC 46 8.087 29.686 4.615 1.00 0.00 MEMB H

ATOM 6097 C211 POPC 46 7.115 31.729 4.897 1.00 0.00 MEMB C

ATOM 6098 H11R POPC 46 6.512 32.216 5.700 1.00 0.00 MEMB H

ATOM 6099 H11S POPC 46 6.190 31.471 4.550 1.00 0.00 MEMB H

ATOM 6100 C212 POPC 46 7.448 32.404 3.637 1.00 0.00 MEMB C

ATOM 6101 H12R POPC 46 8.326 32.810 4.089 1.00 0.00 MEMB H

ATOM 6102 H12S POPC 46 6.775 33.183 3.261 1.00 0.00 MEMB H

ATOM 6103 C213 POPC 46 7.852 31.652 2.426 1.00 0.00 MEMB C

ATOM 6104 H13R POPC 46 6.992 31.186 1.918 1.00 0.00 MEMB H

ATOM 6105 H13S POPC 46 8.630 30.924 2.705 1.00 0.00 MEMB H

ATOM 6106 C214 POPC 46 8.393 32.708 1.518 1.00 0.00 MEMB C

ATOM 6107 H14R POPC 46 7.494 33.343 1.385 1.00 0.00 MEMB H

ATOM 6108 H14S POPC 46 8.573 32.243 0.529 1.00 0.00 MEMB H

ATOM 6109 C215 POPC 46 9.661 33.352 2.201 1.00 0.00 MEMB C

ATOM 6110 H15R POPC 46 10.333 32.493 2.092 1.00 0.00 MEMB H

ATOM 6111 H15S POPC 46 9.608 33.626 3.247 1.00 0.00 MEMB H

ATOM 6112 C216 POPC 46 10.387 34.614 1.669 1.00 0.00 MEMB C

ATOM 6113 H16R POPC 46 11.048 35.251 2.254 1.00 0.00 MEMB H

ATOM 6114 H16S POPC 46 9.729 35.467 1.432 1.00 0.00 MEMB H

ATOM 6115 C217 POPC 46 11.200 33.915 0.609 1.00 0.00 MEMB C

ATOM 6116 H17R POPC 46 10.321 33.460 0.307 1.00 0.00 MEMB H

ATOM 6117 H17S POPC 46 11.500 32.881 0.568 1.00 0.00 MEMB H

ATOM 6118 C218 POPC 46 12.254 34.600 -0.228 1.00 0.00 MEMB C

ATOM 6119 H18R POPC 46 12.572 35.626 -0.395 1.00 0.00 MEMB H

ATOM 6120 H18S POPC 46 11.401 35.175 -0.222 1.00 0.00 MEMB H

ATOM 6121 H18T POPC 46 12.738 34.167 -1.096 1.00 0.00 MEMB H

ATOM 6122 C33 POPC 46 13.332 34.045 13.166 1.00 0.00 MEMB C

ATOM 6123 H3X POPC 46 13.299 35.062 13.344 1.00 0.00 MEMB H

ATOM 6124 H3Y POPC 46 12.515 34.072 12.519 1.00 0.00 MEMB H

ATOM 6125 C34 POPC 46 14.716 34.387 12.918 1.00 0.00 MEMB C

ATOM 6126 H4X POPC 46 15.000 34.747 13.882 1.00 0.00 MEMB H

ATOM 6127 H4Y POPC 46 15.027 35.329 12.381 1.00 0.00 MEMB H

ATOM 6128 C35 POPC 46 15.304 33.182 12.401 1.00 0.00 MEMB C

ATOM 6129 H5X POPC 46 15.133 32.377 13.140 1.00 0.00 MEMB H

ATOM 6130 H5Y POPC 46 16.227 33.806 12.457 1.00 0.00 MEMB H

ATOM 6131 C36 POPC 46 15.217 32.695 10.957 1.00 0.00 MEMB C

ATOM 6132 H6X POPC 46 15.727 33.528 10.410 1.00 0.00 MEMB H

ATOM 6133 H6Y POPC 46 14.181 32.375 10.767 1.00 0.00 MEMB H

ATOM 6134 C37 POPC 46 15.888 31.474 10.357 1.00 0.00 MEMB C

ATOM 6135 H7X POPC 46 15.733 30.747 11.157 1.00 0.00 MEMB H

ATOM 6136 H7Y POPC 46 16.869 31.838 9.982 1.00 0.00 MEMB H

ATOM 6137 C38 POPC 46 15.343 30.721 9.156 1.00 0.00 MEMB C

ATOM 6138 H8X POPC 46 16.098 30.089 8.641 1.00 0.00 MEMB H

ATOM 6139 H8Y POPC 46 15.281 31.560 8.531 1.00 0.00 MEMB H

ATOM 6140 C39 POPC 46 14.013 30.020 9.402 1.00 0.00 MEMB C

ATOM 6141 H9X POPC 46 13.232 30.717 9.750 1.00 0.00 MEMB H

ATOM 6142 H9Y POPC 46 14.194 29.335 10.262 1.00 0.00 MEMB H

ATOM 6143 C310 POPC 46 13.479 29.167 8.234 1.00 0.00 MEMB C

ATOM 6144 H10X POPC 46 12.706 28.545 8.738 1.00 0.00 MEMB H

ATOM 6145 H10Y POPC 46 14.297 28.524 7.847 1.00 0.00 MEMB H

ATOM 6146 C311 POPC 46 12.742 29.857 7.069 1.00 0.00 MEMB C

ATOM 6147 H11X POPC 46 13.414 30.535 6.504 1.00 0.00 MEMB H

ATOM 6148 H11Y POPC 46 11.891 30.432 7.519 1.00 0.00 MEMB H

ATOM 6149 C312 POPC 46 12.158 28.934 5.987 1.00 0.00 MEMB C

ATOM 6150 H12X POPC 46 11.219 29.408 5.615 1.00 0.00 MEMB H

ATOM 6151 H12Y POPC 46 11.917 27.964 6.466 1.00 0.00 MEMB H

ATOM 6152 C313 POPC 46 13.080 28.673 4.774 1.00 0.00 MEMB C

ATOM 6153 H13X POPC 46 12.826 27.683 4.352 1.00 0.00 MEMB H

ATOM 6154 H13Y POPC 46 14.142 28.680 5.111 1.00 0.00 MEMB H

ATOM 6155 C314 POPC 46 12.891 29.671 3.627 1.00 0.00 MEMB C

ATOM 6156 H14X POPC 46 13.070 30.601 4.186 1.00 0.00 MEMB H

ATOM 6157 H14Y POPC 46 11.832 29.707 3.294 1.00 0.00 MEMB H

ATOM 6158 C315 POPC 46 13.811 29.484 2.383 1.00 0.00 MEMB C

ATOM 6159 H15X POPC 46 14.110 28.415 2.349 1.00 0.00 MEMB H

ATOM 6160 H15Y POPC 46 14.754 30.068 2.501 1.00 0.00 MEMB H

ATOM 6161 C316 POPC 46 13.187 29.833 1.011 1.00 0.00 MEMB C

ATOM 6162 H16X POPC 46 12.150 29.450 0.926 1.00 0.00 MEMB H

ATOM 6163 H16Y POPC 46 13.785 29.420 0.168 1.00 0.00 MEMB H

ATOM 6164 H16Z POPC 46 13.141 30.927 0.856 1.00 0.00 MEMB H

ATOM 6165 N POPC 47 -3.171 31.909 19.850 1.00 0.00 MEMB N

ATOM 6166 C12 POPC 47 -1.800 31.374 20.214 1.00 0.00 MEMB C

ATOM 6167 H12A POPC 47 -1.892 30.644 21.005 1.00 0.00 MEMB H

ATOM 6168 H12B POPC 47 -1.239 32.221 20.594 1.00 0.00 MEMB H

ATOM 6169 C13 POPC 47 -3.779 30.964 18.888 1.00 0.00 MEMB C

ATOM 6170 H13A POPC 47 -3.787 29.949 19.271 1.00 0.00 MEMB H

ATOM 6171 H13B POPC 47 -4.772 31.286 18.658 1.00 0.00 MEMB H

ATOM 6172 H13C POPC 47 -3.221 30.985 17.958 1.00 0.00 MEMB H

ATOM 6173 C14 POPC 47 -4.006 32.094 21.066 1.00 0.00 MEMB C

ATOM 6174 H14A POPC 47 -3.491 32.758 21.759 1.00 0.00 MEMB H

ATOM 6175 H14B POPC 47 -4.938 32.600 20.858 1.00 0.00 MEMB H

ATOM 6176 H14C POPC 47 -4.164 31.161 21.587 1.00 0.00 MEMB H

ATOM 6177 C15 POPC 47 -3.012 33.249 19.180 1.00 0.00 MEMB C

ATOM 6178 H15A POPC 47 -2.424 33.129 18.266 1.00 0.00 MEMB H

ATOM 6179 H15B POPC 47 -2.422 33.942 19.782 1.00 0.00 MEMB H

ATOM 6180 H15C POPC 47 -3.963 33.729 18.973 1.00 0.00 MEMB H

ATOM 6181 C11 POPC 47 -0.933 30.795 19.096 1.00 0.00 MEMB C

ATOM 6182 H11A POPC 47 -1.411 29.924 18.602 1.00 0.00 MEMB H

ATOM 6183 H11B POPC 47 0.025 30.415 19.511 1.00 0.00 MEMB H

ATOM 6184 P POPC 47 0.454 32.839 18.172 1.00 0.00 MEMB P

ATOM 6185 O13 POPC 47 1.689 32.132 18.563 1.00 0.00 MEMB O

ATOM 6186 O14 POPC 47 0.025 33.999 18.984 1.00 0.00 MEMB O

ATOM 6187 O12 POPC 47 -0.735 31.801 18.134 1.00 0.00 MEMB O

ATOM 6188 O11 POPC 47 0.414 33.212 16.668 1.00 0.00 MEMB O

ATOM 6189 C1 POPC 47 -0.610 33.987 16.091 1.00 0.00 MEMB C

ATOM 6190 HA POPC 47 -0.091 34.722 15.440 1.00 0.00 MEMB H

ATOM 6191 HB POPC 47 -1.200 34.562 16.841 1.00 0.00 MEMB H

ATOM 6192 C2 POPC 47 -1.607 33.169 15.230 1.00 0.00 MEMB C

ATOM 6193 HS POPC 47 -2.620 33.256 15.694 1.00 0.00 MEMB H

ATOM 6194 O21 POPC 47 -1.231 31.822 15.067 1.00 0.00 MEMB O

ATOM 6195 C21 POPC 47 -2.225 31.107 14.634 1.00 0.00 MEMB C

ATOM 6196 O22 POPC 47 -3.318 31.499 14.273 1.00 0.00 MEMB O

ATOM 6197 C22 POPC 47 -1.789 29.723 14.720 1.00 0.00 MEMB C

ATOM 6198 H2R POPC 47 -2.512 28.928 14.501 1.00 0.00 MEMB H

ATOM 6199 H2S POPC 47 -1.212 29.552 15.667 1.00 0.00 MEMB H

ATOM 6200 C3 POPC 47 -1.741 33.837 13.883 1.00 0.00 MEMB C

ATOM 6201 HX POPC 47 -1.885 34.796 14.365 1.00 0.00 MEMB H

ATOM 6202 HY POPC 47 -2.632 33.560 13.282 1.00 0.00 MEMB H

ATOM 6203 O31 POPC 47 -0.570 33.988 13.119 1.00 0.00 MEMB O

ATOM 6204 C31 POPC 47 -0.628 33.307 11.979 1.00 0.00 MEMB C

ATOM 6205 O32 POPC 47 -1.567 32.744 11.481 1.00 0.00 MEMB O

ATOM 6206 C32 POPC 47 0.748 33.216 11.524 1.00 0.00 MEMB C

ATOM 6207 H2X POPC 47 0.987 32.660 12.446 1.00 0.00 MEMB H

ATOM 6208 H2Y POPC 47 1.128 34.258 11.665 1.00 0.00 MEMB H

ATOM 6209 C23 POPC 47 -0.939 29.599 13.529 1.00 0.00 MEMB C

ATOM 6210 H3R POPC 47 -0.544 28.650 13.969 1.00 0.00 MEMB H

ATOM 6211 H3S POPC 47 -0.146 30.382 13.448 1.00 0.00 MEMB H

ATOM 6212 C24 POPC 47 -1.650 29.503 12.179 1.00 0.00 MEMB C

ATOM 6213 H4R POPC 47 -2.116 30.462 11.914 1.00 0.00 MEMB H

ATOM 6214 H4S POPC 47 -2.273 28.582 12.118 1.00 0.00 MEMB H

ATOM 6215 C25 POPC 47 -0.680 29.400 11.051 1.00 0.00 MEMB C

ATOM 6216 H5R POPC 47 -0.521 28.303 11.156 1.00 0.00 MEMB H

ATOM 6217 H5S POPC 47 0.140 30.030 11.399 1.00 0.00 MEMB H

ATOM 6218 C26 POPC 47 -0.877 29.870 9.604 1.00 0.00 MEMB C

ATOM 6219 H6R POPC 47 -0.307 29.215 8.972 1.00 0.00 MEMB H

ATOM 6220 H6S POPC 47 -0.419 30.786 9.240 1.00 0.00 MEMB H

ATOM 6221 C27 POPC 47 -2.095 29.408 8.962 1.00 0.00 MEMB C

ATOM 6222 H7R POPC 47 -2.951 29.714 9.558 1.00 0.00 MEMB H

ATOM 6223 H7S POPC 47 -1.941 28.352 9.280 1.00 0.00 MEMB H

ATOM 6224 C28 POPC 47 -2.103 29.593 7.391 1.00 0.00 MEMB C

ATOM 6225 H8R POPC 47 -1.444 28.794 6.969 1.00 0.00 MEMB H

ATOM 6226 H8S POPC 47 -1.473 30.422 7.084 1.00 0.00 MEMB H

ATOM 6227 C29 POPC 47 -3.503 29.715 6.746 1.00 0.00 MEMB C

ATOM 6228 H91 POPC 47 -4.284 29.370 7.439 1.00 0.00 MEMB H

ATOM 6229 C210 POPC 47 -3.911 29.391 5.497 1.00 0.00 MEMB C

ATOM 6230 H101 POPC 47 -4.915 28.933 5.395 1.00 0.00 MEMB H

ATOM 6231 C211 POPC 47 -2.991 28.804 4.503 1.00 0.00 MEMB C

ATOM 6232 H11R POPC 47 -2.701 28.030 5.182 1.00 0.00 MEMB H

ATOM 6233 H11S POPC 47 -2.069 29.359 4.330 1.00 0.00 MEMB H

ATOM 6234 C212 POPC 47 -3.535 28.004 3.304 1.00 0.00 MEMB C

ATOM 6235 H12R POPC 47 -4.234 28.702 2.796 1.00 0.00 MEMB H

ATOM 6236 H12S POPC 47 -4.094 27.081 3.593 1.00 0.00 MEMB H

ATOM 6237 C213 POPC 47 -2.512 27.526 2.275 1.00 0.00 MEMB C

ATOM 6238 H13R POPC 47 -3.078 27.257 1.421 1.00 0.00 MEMB H

ATOM 6239 H13S POPC 47 -2.033 26.540 2.375 1.00 0.00 MEMB H

ATOM 6240 C214 POPC 47 -1.643 28.640 1.784 1.00 0.00 MEMB C

ATOM 6241 H14R POPC 47 -0.776 28.703 2.428 1.00 0.00 MEMB H

ATOM 6242 H14S POPC 47 -2.106 29.640 1.806 1.00 0.00 MEMB H

ATOM 6243 C215 POPC 47 -1.171 28.412 0.400 1.00 0.00 MEMB C

ATOM 6244 H15R POPC 47 -1.344 29.388 -0.111 1.00 0.00 MEMB H

ATOM 6245 H15S POPC 47 -1.695 27.605 -0.174 1.00 0.00 MEMB H

ATOM 6246 C216 POPC 47 0.230 27.935 0.586 1.00 0.00 MEMB C

ATOM 6247 H16R POPC 47 0.148 26.991 1.131 1.00 0.00 MEMB H

ATOM 6248 H16S POPC 47 0.983 28.652 1.000 1.00 0.00 MEMB H

ATOM 6249 C217 POPC 47 0.751 27.608 -0.667 1.00 0.00 MEMB C

ATOM 6250 H17R POPC 47 0.286 26.723 -1.182 1.00 0.00 MEMB H

ATOM 6251 H17S POPC 47 1.888 27.564 -0.585 1.00 0.00 MEMB H

ATOM 6252 C218 POPC 47 0.354 28.749 -1.440 1.00 0.00 MEMB C

ATOM 6253 H18R POPC 47 0.828 29.673 -1.224 1.00 0.00 MEMB H

ATOM 6254 H18S POPC 47 -0.561 28.811 -2.054 1.00 0.00 MEMB H

ATOM 6255 H18T POPC 47 0.970 28.214 -2.062 1.00 0.00 MEMB H

ATOM 6256 C33 POPC 47 1.361 32.280 10.310 1.00 0.00 MEMB C

ATOM 6257 H3X POPC 47 0.886 32.905 9.527 1.00 0.00 MEMB H

ATOM 6258 H3Y POPC 47 0.877 31.357 10.392 1.00 0.00 MEMB H

ATOM 6259 C34 POPC 47 2.993 31.717 9.911 1.00 0.00 MEMB C

ATOM 6260 H4X POPC 47 3.442 30.928 10.504 1.00 0.00 MEMB H

ATOM 6261 H4Y POPC 47 3.986 32.167 10.137 1.00 0.00 MEMB H

ATOM 6262 C35 POPC 47 3.577 32.117 8.568 1.00 0.00 MEMB C

ATOM 6263 H5X POPC 47 4.689 32.165 8.360 1.00 0.00 MEMB H

ATOM 6264 H5Y POPC 47 3.152 32.986 8.136 1.00 0.00 MEMB H

ATOM 6265 C36 POPC 47 2.654 31.230 8.091 1.00 0.00 MEMB C

ATOM 6266 H6X POPC 47 1.621 31.258 8.439 1.00 0.00 MEMB H

ATOM 6267 H6Y POPC 47 3.155 30.327 8.465 1.00 0.00 MEMB H

ATOM 6268 C37 POPC 47 2.239 31.227 6.753 1.00 0.00 MEMB C

ATOM 6269 H7X POPC 47 2.467 30.222 6.895 1.00 0.00 MEMB H

ATOM 6270 H7Y POPC 47 2.910 31.319 5.988 1.00 0.00 MEMB H

ATOM 6271 C38 POPC 47 0.919 31.802 6.309 1.00 0.00 MEMB C

ATOM 6272 H8X POPC 47 0.981 32.892 6.436 1.00 0.00 MEMB H

ATOM 6273 H8Y POPC 47 0.138 31.445 6.996 1.00 0.00 MEMB H

ATOM 6274 C39 POPC 47 0.722 31.585 4.799 1.00 0.00 MEMB C

ATOM 6275 H9X POPC 47 1.310 30.788 4.557 1.00 0.00 MEMB H

ATOM 6276 H9Y POPC 47 1.287 31.918 3.938 1.00 0.00 MEMB H

ATOM 6277 C310 POPC 47 -0.543 31.833 4.124 1.00 0.00 MEMB C

ATOM 6278 H10X POPC 47 -1.276 31.109 4.497 1.00 0.00 MEMB H

ATOM 6279 H10Y POPC 47 -0.376 31.676 3.022 1.00 0.00 MEMB H

ATOM 6280 C311 POPC 47 -0.770 33.288 4.390 1.00 0.00 MEMB C

ATOM 6281 H11X POPC 47 -0.096 33.895 3.737 1.00 0.00 MEMB H

ATOM 6282 H11Y POPC 47 -0.521 33.582 5.429 1.00 0.00 MEMB H

ATOM 6283 C312 POPC 47 -2.208 33.595 4.196 1.00 0.00 MEMB C

ATOM 6284 H12X POPC 47 -2.277 34.613 4.640 1.00 0.00 MEMB H

ATOM 6285 H12Y POPC 47 -2.890 32.926 4.761 1.00 0.00 MEMB H

ATOM 6286 C313 POPC 47 -2.570 33.528 2.722 1.00 0.00 MEMB C

ATOM 6287 H13X POPC 47 -2.557 32.551 2.231 1.00 0.00 MEMB H

ATOM 6288 H13Y POPC 47 -1.723 34.034 2.215 1.00 0.00 MEMB H

ATOM 6289 C314 POPC 47 -3.947 34.127 2.598 1.00 0.00 MEMB C

ATOM 6290 H14X POPC 47 -4.716 33.665 3.256 1.00 0.00 MEMB H

ATOM 6291 H14Y POPC 47 -4.334 34.166 1.550 1.00 0.00 MEMB H

ATOM 6292 C315 POPC 47 -3.728 35.523 3.012 1.00 0.00 MEMB C

ATOM 6293 H15X POPC 47 -3.715 35.817 4.084 1.00 0.00 MEMB H

ATOM 6294 H15Y POPC 47 -4.585 35.998 2.646 1.00 0.00 MEMB H

ATOM 6295 C316 POPC 47 -2.673 36.123 2.140 1.00 0.00 MEMB C

ATOM 6296 H16X POPC 47 -2.441 37.083 2.539 1.00 0.00 MEMB H

ATOM 6297 H16Y POPC 47 -3.074 36.154 1.095 1.00 0.00 MEMB H

ATOM 6298 H16Z POPC 47 -1.628 35.874 2.239 1.00 0.00 MEMB H

ATOM 6299 N POPC 48 24.945 -17.927 18.017 1.00 0.00 MEMB N

ATOM 6300 C12 POPC 48 26.027 -16.933 17.603 1.00 0.00 MEMB C

ATOM 6301 H12A POPC 48 25.589 -15.941 17.482 1.00 0.00 MEMB H

ATOM 6302 H12B POPC 48 26.407 -17.218 16.629 1.00 0.00 MEMB H

ATOM 6303 C13 POPC 48 24.201 -17.391 19.195 1.00 0.00 MEMB C

ATOM 6304 H13A POPC 48 23.668 -16.478 18.938 1.00 0.00 MEMB H

ATOM 6305 H13B POPC 48 23.484 -18.128 19.534 1.00 0.00 MEMB H

ATOM 6306 H13C POPC 48 24.894 -17.133 20.002 1.00 0.00 MEMB H

ATOM 6307 C14 POPC 48 23.975 -18.187 16.919 1.00 0.00 MEMB C

ATOM 6308 H14A POPC 48 24.512 -18.490 16.030 1.00 0.00 MEMB H

ATOM 6309 H14B POPC 48 23.320 -19.011 17.198 1.00 0.00 MEMB H

ATOM 6310 H14C POPC 48 23.348 -17.329 16.717 1.00 0.00 MEMB H

ATOM 6311 C15 POPC 48 25.549 -19.257 18.343 1.00 0.00 MEMB C

ATOM 6312 H15A POPC 48 26.242 -19.163 19.175 1.00 0.00 MEMB H

ATOM 6313 H15B POPC 48 26.079 -19.624 17.471 1.00 0.00 MEMB H

ATOM 6314 H15C POPC 48 24.789 -19.985 18.614 1.00 0.00 MEMB H

ATOM 6315 C11 POPC 48 27.256 -16.785 18.552 1.00 0.00 MEMB C

ATOM 6316 H11A POPC 48 28.070 -16.224 18.034 1.00 0.00 MEMB H

ATOM 6317 H11B POPC 48 27.682 -17.790 18.744 1.00 0.00 MEMB H

ATOM 6318 P POPC 48 27.094 -14.639 20.070 1.00 0.00 MEMB P

ATOM 6319 O13 POPC 48 28.515 -14.237 20.102 1.00 0.00 MEMB O

ATOM 6320 O14 POPC 48 26.207 -14.188 21.167 1.00 0.00 MEMB O

ATOM 6321 O12 POPC 48 26.922 -16.202 19.814 1.00 0.00 MEMB O

ATOM 6322 O11 POPC 48 26.500 -14.138 18.700 1.00 0.00 MEMB O

ATOM 6323 C1 POPC 48 25.632 -13.022 18.579 1.00 0.00 MEMB C

ATOM 6324 HA POPC 48 26.027 -12.119 19.093 1.00 0.00 MEMB H

ATOM 6325 HB POPC 48 24.652 -13.228 19.068 1.00 0.00 MEMB H

ATOM 6326 C2 POPC 48 25.348 -12.751 17.074 1.00 0.00 MEMB C

ATOM 6327 HS POPC 48 24.640 -11.880 17.073 1.00 0.00 MEMB H

ATOM 6328 O21 POPC 48 24.816 -13.901 16.433 1.00 0.00 MEMB O

ATOM 6329 C21 POPC 48 24.484 -13.658 15.168 1.00 0.00 MEMB C

ATOM 6330 O22 POPC 48 24.294 -12.561 14.676 1.00 0.00 MEMB O

ATOM 6331 C22 POPC 48 24.191 -15.000 14.534 1.00 0.00 MEMB C

ATOM 6332 H2R POPC 48 23.115 -15.114 14.787 1.00 0.00 MEMB H

ATOM 6333 H2S POPC 48 24.746 -15.812 15.041 1.00 0.00 MEMB H

ATOM 6334 C3 POPC 48 26.557 -12.345 16.203 1.00 0.00 MEMB C

ATOM 6335 HX POPC 48 26.808 -11.604 16.759 1.00 0.00 MEMB H

ATOM 6336 HY POPC 48 26.363 -11.632 15.366 1.00 0.00 MEMB H

ATOM 6337 O31 POPC 48 27.803 -13.033 16.325 1.00 0.00 MEMB O

ATOM 6338 C31 POPC 48 28.292 -13.167 15.134 1.00 0.00 MEMB C

ATOM 6339 O32 POPC 48 27.776 -12.756 14.108 1.00 0.00 MEMB O

ATOM 6340 C32 POPC 48 29.585 -13.892 15.245 1.00 0.00 MEMB C

ATOM 6341 H2X POPC 48 29.477 -14.720 15.980 1.00 0.00 MEMB H

ATOM 6342 H2Y POPC 48 30.358 -13.178 15.600 1.00 0.00 MEMB H

ATOM 6343 C23 POPC 48 24.339 -14.995 12.994 1.00 0.00 MEMB C

ATOM 6344 H3R POPC 48 23.766 -14.116 12.623 1.00 0.00 MEMB H

ATOM 6345 H3S POPC 48 23.834 -15.905 12.604 1.00 0.00 MEMB H

ATOM 6346 C24 POPC 48 25.765 -14.960 12.405 1.00 0.00 MEMB C

ATOM 6347 H4R POPC 48 26.187 -15.987 12.325 1.00 0.00 MEMB H

ATOM 6348 H4S POPC 48 26.444 -14.362 13.045 1.00 0.00 MEMB H

ATOM 6349 C25 POPC 48 25.863 -14.331 11.001 1.00 0.00 MEMB C

ATOM 6350 H5R POPC 48 25.315 -14.940 10.250 1.00 0.00 MEMB H

ATOM 6351 H5S POPC 48 26.941 -14.331 10.720 1.00 0.00 MEMB H

ATOM 6352 C26 POPC 48 25.340 -12.889 10.906 1.00 0.00 MEMB C

ATOM 6353 H6R POPC 48 25.562 -12.347 11.855 1.00 0.00 MEMB H

ATOM 6354 H6S POPC 48 24.238 -12.975 10.823 1.00 0.00 MEMB H

ATOM 6355 C27 POPC 48 25.963 -12.161 9.691 1.00 0.00 MEMB C

ATOM 6356 H7R POPC 48 25.877 -12.813 8.799 1.00 0.00 MEMB H

ATOM 6357 H7S POPC 48 27.042 -12.045 9.934 1.00 0.00 MEMB H

ATOM 6358 C28 POPC 48 25.495 -10.731 9.330 1.00 0.00 MEMB C

ATOM 6359 H8R POPC 48 26.247 -10.237 8.699 1.00 0.00 MEMB H

ATOM 6360 H8S POPC 48 25.431 -10.169 10.298 1.00 0.00 MEMB H

ATOM 6361 C29 POPC 48 24.218 -10.583 8.576 1.00 0.00 MEMB C

ATOM 6362 H91 POPC 48 23.589 -9.820 9.006 1.00 0.00 MEMB H

ATOM 6363 C210 POPC 48 23.682 -11.134 7.493 1.00 0.00 MEMB C

ATOM 6364 H101 POPC 48 22.589 -11.160 7.461 1.00 0.00 MEMB H

ATOM 6365 C211 POPC 48 24.185 -12.138 6.584 1.00 0.00 MEMB C

ATOM 6366 H11R POPC 48 25.276 -12.051 6.675 1.00 0.00 MEMB H

ATOM 6367 H11S POPC 48 23.839 -11.704 5.624 1.00 0.00 MEMB H

ATOM 6368 C212 POPC 48 23.799 -13.632 6.717 1.00 0.00 MEMB C

ATOM 6369 H12R POPC 48 24.080 -14.087 7.682 1.00 0.00 MEMB H

ATOM 6370 H12S POPC 48 24.436 -14.094 5.930 1.00 0.00 MEMB H

ATOM 6371 C213 POPC 48 22.372 -14.045 6.403 1.00 0.00 MEMB C

ATOM 6372 H13R POPC 48 21.631 -13.710 7.167 1.00 0.00 MEMB H

ATOM 6373 H13S POPC 48 22.263 -15.150 6.276 1.00 0.00 MEMB H

ATOM 6374 C214 POPC 48 21.987 -13.483 5.070 1.00 0.00 MEMB C

ATOM 6375 H14R POPC 48 21.861 -12.387 5.230 1.00 0.00 MEMB H

ATOM 6376 H14S POPC 48 20.963 -13.862 5.084 1.00 0.00 MEMB H

ATOM 6377 C215 POPC 48 22.954 -13.972 3.875 1.00 0.00 MEMB C

ATOM 6378 H15R POPC 48 23.203 -15.028 4.010 1.00 0.00 MEMB H

ATOM 6379 H15S POPC 48 23.806 -13.306 4.096 1.00 0.00 MEMB H

ATOM 6380 C216 POPC 48 22.869 -14.036 2.286 1.00 0.00 MEMB C

ATOM 6381 H16R POPC 48 22.097 -13.529 1.706 1.00 0.00 MEMB H

ATOM 6382 H16S POPC 48 22.462 -14.917 1.785 1.00 0.00 MEMB H

ATOM 6383 C217 POPC 48 23.773 -13.075 1.598 1.00 0.00 MEMB C

ATOM 6384 H17R POPC 48 23.880 -12.049 2.040 1.00 0.00 MEMB H

ATOM 6385 H17S POPC 48 23.307 -13.228 0.607 1.00 0.00 MEMB H

ATOM 6386 C218 POPC 48 25.040 -13.263 0.974 1.00 0.00 MEMB C

ATOM 6387 H18R POPC 48 25.462 -13.544 1.936 1.00 0.00 MEMB H

ATOM 6388 H18S POPC 48 25.306 -12.233 0.676 1.00 0.00 MEMB H

ATOM 6389 H18T POPC 48 24.879 -13.957 0.101 1.00 0.00 MEMB H

ATOM 6390 C33 POPC 48 29.868 -14.413 13.842 1.00 0.00 MEMB C

ATOM 6391 H3X POPC 48 29.947 -13.559 13.128 1.00 0.00 MEMB H

ATOM 6392 H3Y POPC 48 28.991 -15.023 13.516 1.00 0.00 MEMB H

ATOM 6393 C34 POPC 48 31.146 -15.239 13.816 1.00 0.00 MEMB C

ATOM 6394 H4X POPC 48 31.141 -15.934 14.684 1.00 0.00 MEMB H

ATOM 6395 H4Y POPC 48 32.013 -14.553 13.937 1.00 0.00 MEMB H

ATOM 6396 C35 POPC 48 31.330 -16.013 12.509 1.00 0.00 MEMB C

ATOM 6397 H5X POPC 48 32.243 -16.643 12.589 1.00 0.00 MEMB H

ATOM 6398 H5Y POPC 48 31.511 -15.279 11.691 1.00 0.00 MEMB H

ATOM 6399 C36 POPC 48 30.130 -16.901 12.143 1.00 0.00 MEMB C

ATOM 6400 H6X POPC 48 29.257 -16.273 11.857 1.00 0.00 MEMB H

ATOM 6401 H6Y POPC 48 29.830 -17.497 13.033 1.00 0.00 MEMB H

ATOM 6402 C37 POPC 48 30.443 -17.871 10.999 1.00 0.00 MEMB C

ATOM 6403 H7X POPC 48 29.560 -18.530 10.845 1.00 0.00 MEMB H

ATOM 6404 H7Y POPC 48 31.285 -18.522 11.331 1.00 0.00 MEMB H

ATOM 6405 C38 POPC 48 30.840 -17.193 9.672 1.00 0.00 MEMB C

ATOM 6406 H8X POPC 48 30.835 -17.958 8.864 1.00 0.00 MEMB H

ATOM 6407 H8Y POPC 48 31.881 -16.809 9.773 1.00 0.00 MEMB H

ATOM 6408 C39 POPC 48 29.981 -16.003 9.206 1.00 0.00 MEMB C

ATOM 6409 H9X POPC 48 30.497 -15.549 8.327 1.00 0.00 MEMB H

ATOM 6410 H9Y POPC 48 29.927 -15.212 9.984 1.00 0.00 MEMB H

ATOM 6411 C310 POPC 48 28.577 -16.396 8.737 1.00 0.00 MEMB C

ATOM 6412 H10X POPC 48 27.968 -16.720 9.608 1.00 0.00 MEMB H

ATOM 6413 H10Y POPC 48 28.699 -17.275 8.066 1.00 0.00 MEMB H

ATOM 6414 C311 POPC 48 27.899 -15.238 7.983 1.00 0.00 MEMB C

ATOM 6415 H11X POPC 48 28.606 -14.847 7.217 1.00 0.00 MEMB H

ATOM 6416 H11Y POPC 48 27.715 -14.397 8.686 1.00 0.00 MEMB H

ATOM 6417 C312 POPC 48 26.591 -15.587 7.255 1.00 0.00 MEMB C

ATOM 6418 H12X POPC 48 26.300 -14.682 6.676 1.00 0.00 MEMB H

ATOM 6419 H12Y POPC 48 25.775 -15.790 7.981 1.00 0.00 MEMB H

ATOM 6420 C313 POPC 48 26.690 -16.764 6.276 1.00 0.00 MEMB C

ATOM 6421 H13X POPC 48 25.658 -17.081 6.002 1.00 0.00 MEMB H

ATOM 6422 H13Y POPC 48 27.159 -17.645 6.757 1.00 0.00 MEMB H

ATOM 6423 C314 POPC 48 27.395 -16.384 4.969 1.00 0.00 MEMB C

ATOM 6424 H14X POPC 48 27.573 -17.324 4.397 1.00 0.00 MEMB H

ATOM 6425 H14Y POPC 48 28.393 -15.923 5.135 1.00 0.00 MEMB H

ATOM 6426 C315 POPC 48 26.491 -15.483 4.117 1.00 0.00 MEMB C

ATOM 6427 H15X POPC 48 26.638 -14.404 4.350 1.00 0.00 MEMB H

ATOM 6428 H15Y POPC 48 25.434 -15.715 4.387 1.00 0.00 MEMB H

ATOM 6429 C316 POPC 48 26.662 -15.770 2.632 1.00 0.00 MEMB C

ATOM 6430 H16X POPC 48 25.961 -15.219 2.021 1.00 0.00 MEMB H

ATOM 6431 H16Y POPC 48 26.410 -16.822 2.407 1.00 0.00 MEMB H

ATOM 6432 H16Z POPC 48 27.679 -15.514 2.278 1.00 0.00 MEMB H

ATOM 6433 N POPC 49 -23.828 23.959 19.491 1.00 0.00 MEMB N

ATOM 6434 C12 POPC 49 -23.798 22.435 19.379 1.00 0.00 MEMB C

ATOM 6435 H12A POPC 49 -22.998 22.071 20.015 1.00 0.00 MEMB H

ATOM 6436 H12B POPC 49 -23.541 22.151 18.366 1.00 0.00 MEMB H

ATOM 6437 C13 POPC 49 -24.689 24.572 18.432 1.00 0.00 MEMB C

ATOM 6438 H13A POPC 49 -24.779 25.628 18.622 1.00 0.00 MEMB H

ATOM 6439 H13B POPC 49 -24.236 24.438 17.455 1.00 0.00 MEMB H

ATOM 6440 H13C POPC 49 -25.678 24.113 18.437 1.00 0.00 MEMB H

ATOM 6441 C14 POPC 49 -24.337 24.340 20.839 1.00 0.00 MEMB C

ATOM 6442 H14A POPC 49 -23.727 23.856 21.592 1.00 0.00 MEMB H

ATOM 6443 H14B POPC 49 -24.284 25.408 21.003 1.00 0.00 MEMB H

ATOM 6444 H14C POPC 49 -25.368 24.029 20.938 1.00 0.00 MEMB H

ATOM 6445 C15 POPC 49 -22.435 24.470 19.332 1.00 0.00 MEMB C

ATOM 6446 H15A POPC 49 -22.365 25.552 19.383 1.00 0.00 MEMB H

ATOM 6447 H15B POPC 49 -21.980 24.169 18.394 1.00 0.00 MEMB H

ATOM 6448 H15C POPC 49 -21.786 24.118 20.141 1.00 0.00 MEMB H

ATOM 6449 C11 POPC 49 -25.065 21.625 19.733 1.00 0.00 MEMB C

ATOM 6450 H11A POPC 49 -25.401 21.828 20.767 1.00 0.00 MEMB H

ATOM 6451 H11B POPC 49 -24.756 20.546 19.719 1.00 0.00 MEMB H

ATOM 6452 P POPC 49 -27.084 20.728 18.334 1.00 0.00 MEMB P

ATOM 6453 O13 POPC 49 -28.449 21.020 18.804 1.00 0.00 MEMB O

ATOM 6454 O14 POPC 49 -26.479 19.439 18.702 1.00 0.00 MEMB O

ATOM 6455 O12 POPC 49 -26.117 21.922 18.817 1.00 0.00 MEMB O

ATOM 6456 O11 POPC 49 -27.058 20.824 16.769 1.00 0.00 MEMB O

ATOM 6457 C1 POPC 49 -26.108 21.604 16.078 1.00 0.00 MEMB C

ATOM 6458 HA POPC 49 -25.777 21.020 15.196 1.00 0.00 MEMB H

ATOM 6459 HB POPC 49 -25.206 21.850 16.684 1.00 0.00 MEMB H

ATOM 6460 C2 POPC 49 -26.765 22.883 15.571 1.00 0.00 MEMB C

ATOM 6461 HS POPC 49 -27.325 23.315 16.428 1.00 0.00 MEMB H

ATOM 6462 O21 POPC 49 -27.620 22.502 14.499 1.00 0.00 MEMB O

ATOM 6463 C21 POPC 49 -28.229 23.423 13.818 1.00 0.00 MEMB C

ATOM 6464 O22 POPC 49 -28.510 24.556 14.180 1.00 0.00 MEMB O

ATOM 6465 C22 POPC 49 -28.617 22.760 12.520 1.00 0.00 MEMB C

ATOM 6466 H2R POPC 49 -29.619 23.070 12.334 1.00 0.00 MEMB H

ATOM 6467 H2S POPC 49 -28.577 21.652 12.588 1.00 0.00 MEMB H

ATOM 6468 C3 POPC 49 -25.727 23.847 15.021 1.00 0.00 MEMB C

ATOM 6469 HX POPC 49 -24.895 24.036 15.718 1.00 0.00 MEMB H

ATOM 6470 HY POPC 49 -26.239 24.814 14.798 1.00 0.00 MEMB H

ATOM 6471 O31 POPC 49 -25.216 23.198 13.861 1.00 0.00 MEMB O

ATOM 6472 C31 POPC 49 -24.836 24.040 12.952 1.00 0.00 MEMB C

ATOM 6473 O32 POPC 49 -24.780 25.252 13.070 1.00 0.00 MEMB O

ATOM 6474 C32 POPC 49 -24.483 23.279 11.748 1.00 0.00 MEMB C

ATOM 6475 H2X POPC 49 -24.965 23.666 10.832 1.00 0.00 MEMB H

ATOM 6476 H2Y POPC 49 -24.651 22.183 11.850 1.00 0.00 MEMB H

ATOM 6477 C23 POPC 49 -28.190 23.319 11.190 1.00 0.00 MEMB C

ATOM 6478 H3R POPC 49 -27.123 23.550 11.281 1.00 0.00 MEMB H

ATOM 6479 H3S POPC 49 -28.758 24.265 10.981 1.00 0.00 MEMB H

ATOM 6480 C24 POPC 49 -28.524 22.236 10.161 1.00 0.00 MEMB C

ATOM 6481 H4R POPC 49 -29.604 21.947 10.162 1.00 0.00 MEMB H

ATOM 6482 H4S POPC 49 -28.018 21.285 10.426 1.00 0.00 MEMB H

ATOM 6483 C25 POPC 49 -28.117 22.642 8.762 1.00 0.00 MEMB C

ATOM 6484 H5R POPC 49 -28.790 23.437 8.415 1.00 0.00 MEMB H

ATOM 6485 H5S POPC 49 -28.130 21.715 8.167 1.00 0.00 MEMB H

ATOM 6486 C26 POPC 49 -26.733 23.216 8.578 1.00 0.00 MEMB C

ATOM 6487 H6R POPC 49 -26.062 22.456 9.025 1.00 0.00 MEMB H

ATOM 6488 H6S POPC 49 -26.665 24.163 9.146 1.00 0.00 MEMB H

ATOM 6489 C27 POPC 49 -26.318 23.454 7.108 1.00 0.00 MEMB C

ATOM 6490 H7R POPC 49 -26.274 22.476 6.575 1.00 0.00 MEMB H

ATOM 6491 H7S POPC 49 -25.302 23.890 7.134 1.00 0.00 MEMB H

ATOM 6492 C28 POPC 49 -27.140 24.419 6.228 1.00 0.00 MEMB C

ATOM 6493 H8R POPC 49 -28.141 24.022 6.124 1.00 0.00 MEMB H

ATOM 6494 H8S POPC 49 -26.717 24.363 5.200 1.00 0.00 MEMB H

ATOM 6495 C29 POPC 49 -27.254 25.836 6.712 1.00 0.00 MEMB C

ATOM 6496 H91 POPC 49 -27.892 25.963 7.601 1.00 0.00 MEMB H

ATOM 6497 C210 POPC 49 -26.707 26.919 6.134 1.00 0.00 MEMB C

ATOM 6498 H101 POPC 49 -26.820 27.888 6.644 1.00 0.00 MEMB H

ATOM 6499 C211 POPC 49 -25.861 26.940 4.879 1.00 0.00 MEMB C

ATOM 6500 H11R POPC 49 -25.783 25.951 4.383 1.00 0.00 MEMB H

ATOM 6501 H11S POPC 49 -24.839 27.302 5.102 1.00 0.00 MEMB H

ATOM 6502 C212 POPC 49 -26.384 27.845 3.799 1.00 0.00 MEMB C

ATOM 6503 H12R POPC 49 -25.587 28.054 3.036 1.00 0.00 MEMB H

ATOM 6504 H12S POPC 49 -26.571 28.853 4.245 1.00 0.00 MEMB H

ATOM 6505 C213 POPC 49 -27.606 27.199 3.081 1.00 0.00 MEMB C

ATOM 6506 H13R POPC 49 -28.124 27.787 2.318 1.00 0.00 MEMB H

ATOM 6507 H13S POPC 49 -28.413 26.999 3.808 1.00 0.00 MEMB H

ATOM 6508 C214 POPC 49 -27.253 26.113 2.074 1.00 0.00 MEMB C

ATOM 6509 H14R POPC 49 -28.113 25.422 2.003 1.00 0.00 MEMB H

ATOM 6510 H14S POPC 49 -26.256 25.735 2.343 1.00 0.00 MEMB H

ATOM 6511 C215 POPC 49 -27.046 26.539 0.648 1.00 0.00 MEMB C

ATOM 6512 H15R POPC 49 -26.337 26.009 0.035 1.00 0.00 MEMB H

ATOM 6513 H15S POPC 49 -26.510 27.519 0.750 1.00 0.00 MEMB H

ATOM 6514 C216 POPC 49 -28.305 26.557 -0.216 1.00 0.00 MEMB C

ATOM 6515 H16R POPC 49 -27.973 26.817 -1.259 1.00 0.00 MEMB H

ATOM 6516 H16S POPC 49 -28.912 27.307 0.346 1.00 0.00 MEMB H

ATOM 6517 C217 POPC 49 -29.352 25.682 -0.636 1.00 0.00 MEMB C

ATOM 6518 H17R POPC 49 -28.904 24.690 -0.638 1.00 0.00 MEMB H

ATOM 6519 H17S POPC 49 -29.699 26.052 -1.669 1.00 0.00 MEMB H

ATOM 6520 C218 POPC 49 -30.487 25.927 0.284 1.00 0.00 MEMB C

ATOM 6521 H18R POPC 49 -30.828 27.005 0.334 1.00 0.00 MEMB H

ATOM 6522 H18S POPC 49 -30.396 25.552 1.309 1.00 0.00 MEMB H

ATOM 6523 H18T POPC 49 -31.163 25.307 -0.301 1.00 0.00 MEMB H

ATOM 6524 C33 POPC 49 -23.036 23.478 11.629 1.00 0.00 MEMB C

ATOM 6525 H3X POPC 49 -22.466 23.179 12.544 1.00 0.00 MEMB H

ATOM 6526 H3Y POPC 49 -22.717 24.538 11.430 1.00 0.00 MEMB H

ATOM 6527 C34 POPC 49 -22.693 22.513 10.527 1.00 0.00 MEMB C

ATOM 6528 H4X POPC 49 -22.098 21.706 10.967 1.00 0.00 MEMB H

ATOM 6529 H4Y POPC 49 -22.140 23.297 10.119 1.00 0.00 MEMB H

ATOM 6530 C35 POPC 49 -23.378 22.272 9.161 1.00 0.00 MEMB C

ATOM 6531 H5X POPC 49 -23.602 23.261 8.705 1.00 0.00 MEMB H

ATOM 6532 H5Y POPC 49 -24.291 21.711 9.400 1.00 0.00 MEMB H

ATOM 6533 C36 POPC 49 -22.549 21.470 8.100 1.00 0.00 MEMB C

ATOM 6534 H6X POPC 49 -22.226 20.585 8.562 1.00 0.00 MEMB H

ATOM 6535 H6Y POPC 49 -21.484 21.633 7.954 1.00 0.00 MEMB H

ATOM 6536 C37 POPC 49 -23.320 21.181 6.785 1.00 0.00 MEMB C

ATOM 6537 H7X POPC 49 -24.389 21.055 6.983 1.00 0.00 MEMB H

ATOM 6538 H7Y POPC 49 -22.821 20.348 6.211 1.00 0.00 MEMB H

ATOM 6539 C38 POPC 49 -23.303 22.380 5.987 1.00 0.00 MEMB C

ATOM 6540 H8X POPC 49 -22.245 22.049 5.909 1.00 0.00 MEMB H

ATOM 6541 H8Y POPC 49 -23.486 23.239 6.653 1.00 0.00 MEMB H

ATOM 6542 C39 POPC 49 -23.792 22.658 4.577 1.00 0.00 MEMB C

ATOM 6543 H9X POPC 49 -23.682 23.744 4.331 1.00 0.00 MEMB H

ATOM 6544 H9Y POPC 49 -24.864 22.407 4.562 1.00 0.00 MEMB H

ATOM 6545 C310 POPC 49 -23.013 22.026 3.457 1.00 0.00 MEMB C

ATOM 6546 H10X POPC 49 -22.065 21.505 3.754 1.00 0.00 MEMB H

ATOM 6547 H10Y POPC 49 -22.743 22.739 2.635 1.00 0.00 MEMB H

ATOM 6548 C311 POPC 49 -24.008 21.240 2.724 1.00 0.00 MEMB C

ATOM 6549 H11X POPC 49 -24.787 21.866 2.238 1.00 0.00 MEMB H

ATOM 6550 H11Y POPC 49 -24.406 20.422 3.361 1.00 0.00 MEMB H

ATOM 6551 C312 POPC 49 -23.271 20.689 1.634 1.00 0.00 MEMB C

ATOM 6552 H12X POPC 49 -22.478 20.164 2.190 1.00 0.00 MEMB H

ATOM 6553 H12Y POPC 49 -22.762 21.397 0.938 1.00 0.00 MEMB H

ATOM 6554 C313 POPC 49 -24.207 19.862 0.850 1.00 0.00 MEMB C

ATOM 6555 H13X POPC 49 -24.993 19.307 1.330 1.00 0.00 MEMB H

ATOM 6556 H13Y POPC 49 -23.651 18.995 0.552 1.00 0.00 MEMB H

ATOM 6557 C314 POPC 49 -24.668 20.722 -0.243 1.00 0.00 MEMB C

ATOM 6558 H14X POPC 49 -23.700 20.979 -0.727 1.00 0.00 MEMB H

ATOM 6559 H14Y POPC 49 -25.077 21.689 0.075 1.00 0.00 MEMB H

ATOM 6560 C315 POPC 49 -25.713 20.049 -1.093 1.00 0.00 MEMB C

ATOM 6561 H15X POPC 49 -25.655 18.995 -1.425 1.00 0.00 MEMB H

ATOM 6562 H15Y POPC 49 -26.046 20.532 -2.004 1.00 0.00 MEMB H

ATOM 6563 C316 POPC 49 -26.707 19.710 -0.414 1.00 0.00 MEMB C

ATOM 6564 H16X POPC 49 -27.367 19.236 -1.204 1.00 0.00 MEMB H

ATOM 6565 H16Y POPC 49 -26.995 20.710 -0.047 1.00 0.00 MEMB H

ATOM 6566 H16Z POPC 49 -26.382 18.913 0.261 1.00 0.00 MEMB H

ATOM 6567 N POPC 50 -22.292 -23.060 17.975 1.00 0.00 MEMB N

ATOM 6568 C12 POPC 50 -20.867 -23.235 18.483 1.00 0.00 MEMB C

ATOM 6569 H12A POPC 50 -20.381 -22.272 18.392 1.00 0.00 MEMB H

ATOM 6570 H12B POPC 50 -20.330 -23.936 17.835 1.00 0.00 MEMB H

ATOM 6571 C13 POPC 50 -22.941 -21.976 18.773 1.00 0.00 MEMB C

ATOM 6572 H13A POPC 50 -23.908 -21.689 18.348 1.00 0.00 MEMB H

ATOM 6573 H13B POPC 50 -23.124 -22.300 19.789 1.00 0.00 MEMB H

ATOM 6574 H13C POPC 50 -22.326 -21.083 18.820 1.00 0.00 MEMB H

ATOM 6575 C14 POPC 50 -22.302 -22.743 16.501 1.00 0.00 MEMB C

ATOM 6576 H14A POPC 50 -21.873 -21.778 16.252 1.00 0.00 MEMB H

ATOM 6577 H14B POPC 50 -21.773 -23.506 15.929 1.00 0.00 MEMB H

ATOM 6578 H14C POPC 50 -23.321 -22.722 16.112 1.00 0.00 MEMB H

ATOM 6579 C15 POPC 50 -23.070 -24.321 18.197 1.00 0.00 MEMB C

ATOM 6580 H15A POPC 50 -22.554 -25.197 17.814 1.00 0.00 MEMB H
[truncated: 716,023 more chars]
