# Supplementary material for: Benzaldehyde, A New Absorption Promoter, Accelerating Absorption on Low Bioavailability Drugs Through Membrane Permeability
Source: Front Pharmacol. 2021 May 28;12:663743. doi: 10.3389/fphar.2021.663743 (PMC8194254; doi:10.3389/fphar.2021.663743)
Supplement: Supplementary file 1 [file DataSheet1.zip › Supplementary file 13.DOCX]

;;

;; Generated by CHARMM-GUI FF-Converter

;;

;; Correspondance:

;; jul316@lehigh.edu or wonpil@lehigh.edu

;;

;; GROMACS topology file for TIP3

;;

[ moleculetype ]

; name nrexcl

TIP3 2

[ atoms ]

; nr type resnr residu atom cgnr charge mass

1 OT 1 TIP3 OH2 1 -0.834000 15.9994 ; qtot -0.834

2 HT 1 TIP3 H1 2 0.417000 1.0080 ; qtot -0.417

3 HT 1 TIP3 H2 3 0.417000 1.0080 ; qtot 0.000

[ settles ]

; OW funct doh dhh

1 1 9.572000e-02 1.513900e-01

[ exclusions ]

1 2 3

2 1 3

3 1 2
