# Supplementary material for: Benzaldehyde, A New Absorption Promoter, Accelerating Absorption on Low Bioavailability Drugs Through Membrane Permeability
Source: Front Pharmacol. 2021 May 28;12:663743. doi: 10.3389/fphar.2021.663743 (PMC8194254; doi:10.3389/fphar.2021.663743)
Supplement: Supplementary file 1 [file DataSheet1.zip › Supplementary file 2.DOCX]

| 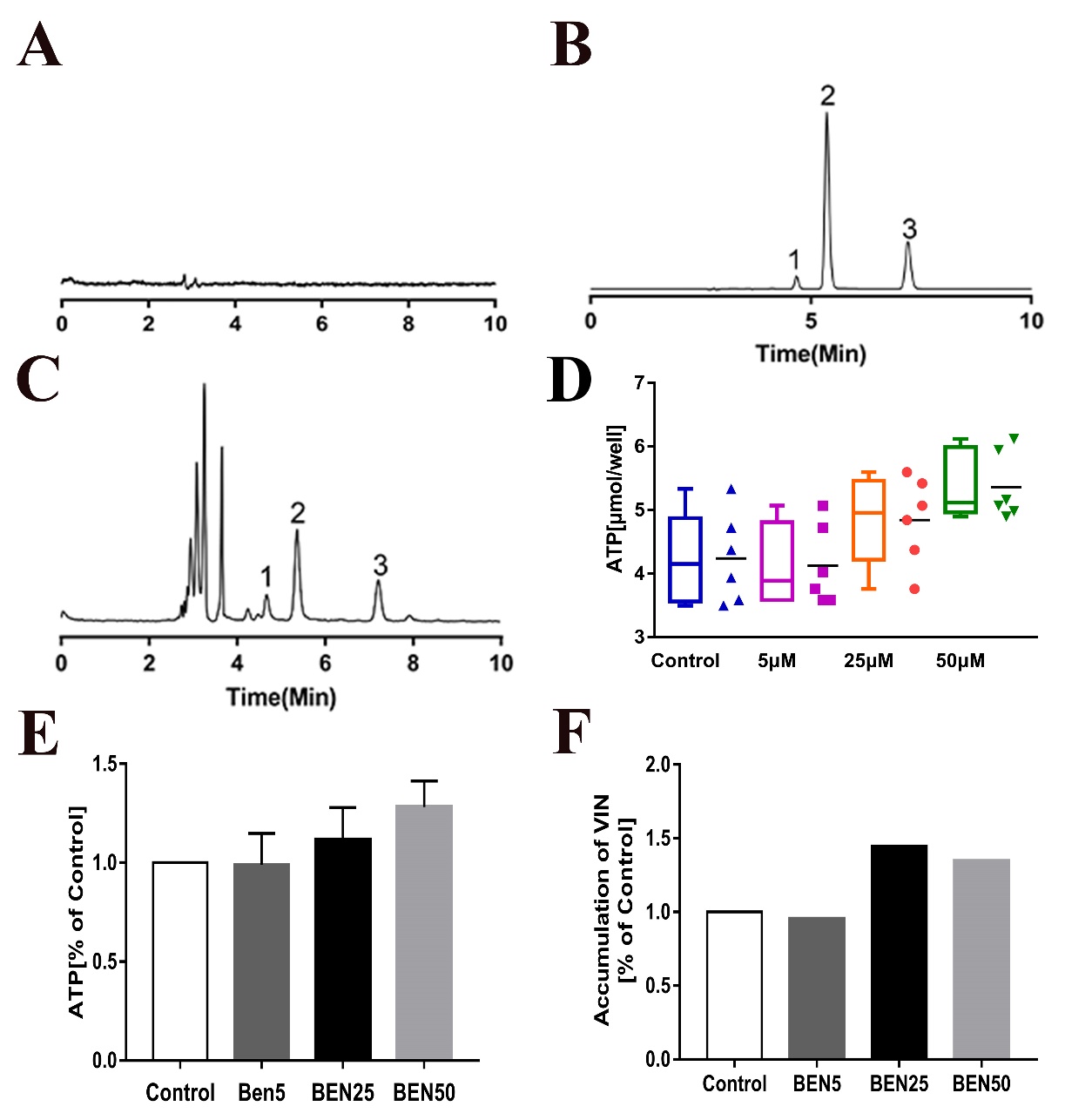 |
| --- |
| **Figure S2 Chromatograms of ATP and accumulation rate of VIN**  (A) blank flow phase sample; (B) ATP 10μM, ADP 1μM, and AMP 100μM; (C) sample from Caco-2 cell administrating 50μM benzaldehyde; (D) The concentration of ATP with or without benzaldehyde (mean±SD, n = 6); (E) the ATP growth rate of Benzaldehyde groups compared with control group; (F) the accumulation rate of VIN in Benzaldehyde groups compared with control group |
|  |
| 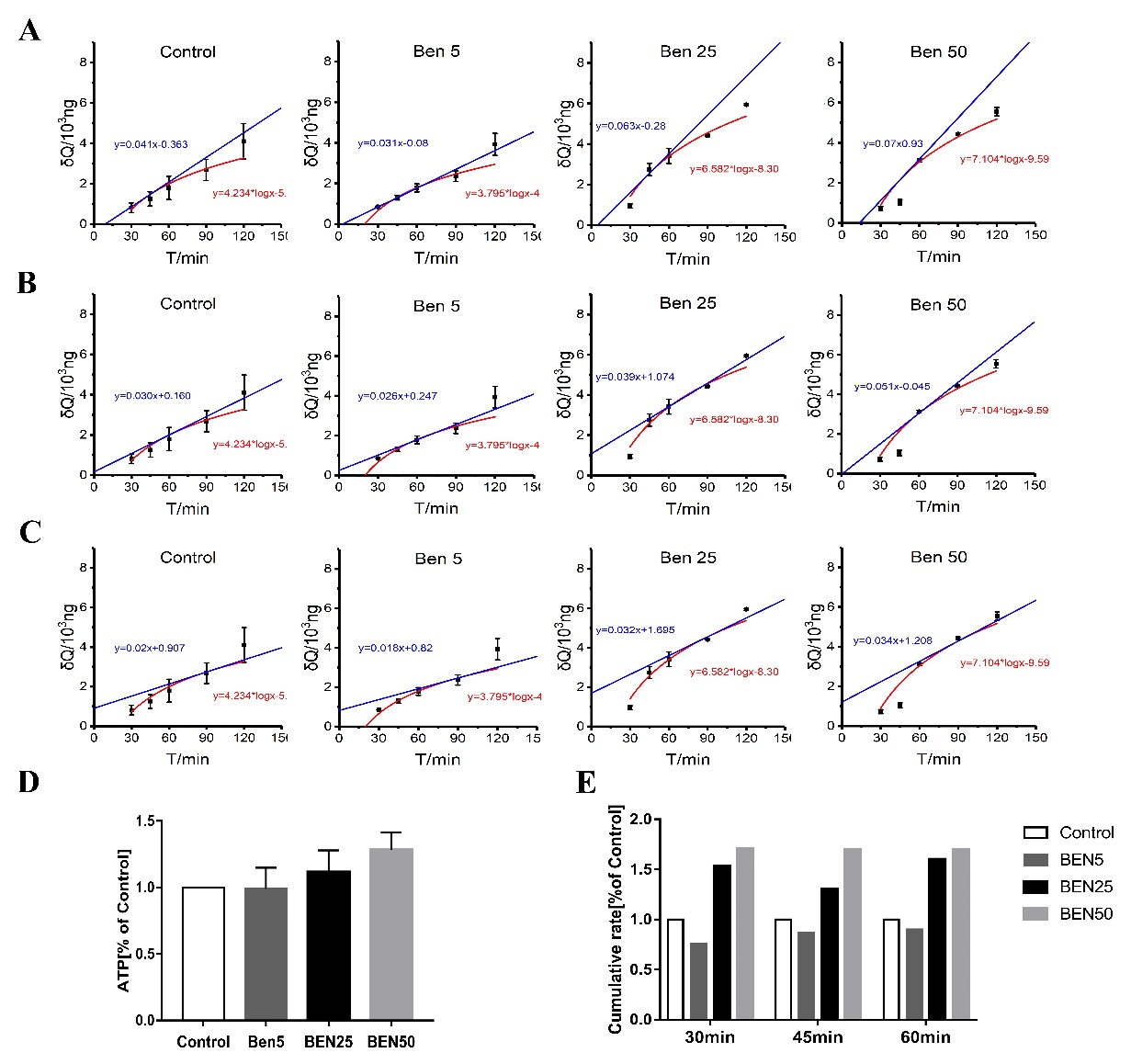 |
| **Figure S3. The accumulation rate of VIN**  (A) The accumulation rate of VIN at 30min; (B) The accumulation rate of VIN at 45min; (C) The accumulation rate of VIN at 60min; (D) the ATP growth rate of Benzaldehyde groups compared with control group; (E) the accumulation rate of VIN in Benzaldehyde groups compared with control group. |
