# Supplementary material for: Benzaldehyde, A New Absorption Promoter, Accelerating Absorption on Low Bioavailability Drugs Through Membrane Permeability
Source: Front Pharmacol. 2021 May 28;12:663743. doi: 10.3389/fphar.2021.663743 (PMC8194254; doi:10.3389/fphar.2021.663743)
Supplement: Supplementary file 1 [file DataSheet1.zip › Supplementary file 3.DOCX]

| 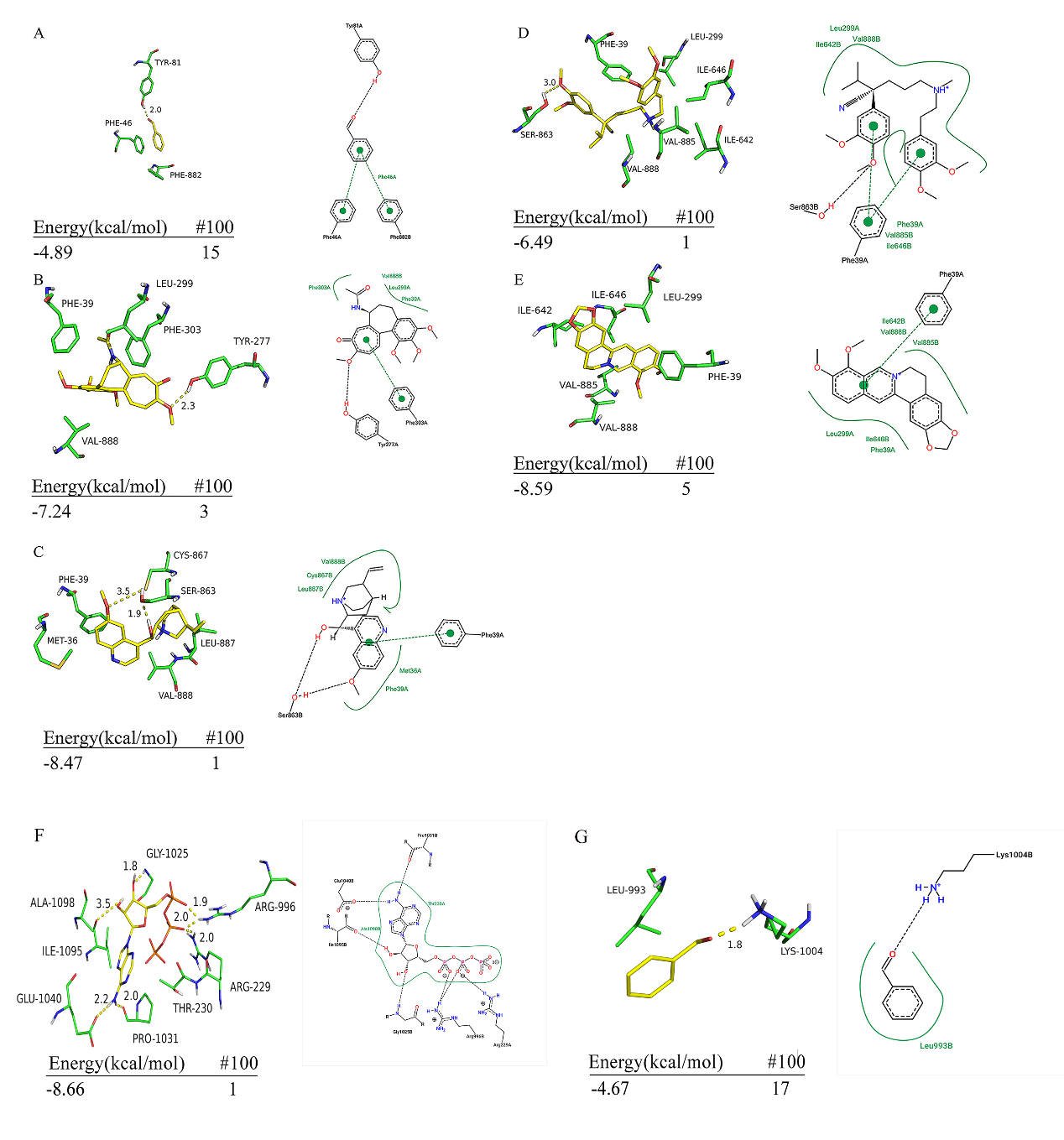 |
| --- |
| **Figure S3. Drug docking results of benzaldehyde with P-gp drug binding site or P-gp ATP binding site**  A-E: Drug docking models in the human P-gp drug site binding pocket with benzaldehyde, colchicine, quinidine, verapamil and berberine; F-G: Drug docking models in the human P-gp ATP site binding pocket with ATP and benzaldehyde. The energy of the binding pose and the number of runs were shown below. The information of H-bonds and hydrophobic interactions between drugs and P-gp are highlighted in the right. Color code: drug=yellow; residues=green; O=red; S= orange; N= blue; H=white. |
