# Supplementary material for: Benzaldehyde, A New Absorption Promoter, Accelerating Absorption on Low Bioavailability Drugs Through Membrane Permeability
Source: Front Pharmacol. 2021 May 28;12:663743. doi: 10.3389/fphar.2021.663743 (PMC8194254; doi:10.3389/fphar.2021.663743)
Supplement: Supplementary file 1 [file DataSheet1.zip › Supplementary file 4.DOCX]

2 8 14 5 ; CG2R61 CG2O4 HGR52

[ dihedrals ]

; ai aj ak al funct c0 c1 c2 c3 c4 c5

4 2 3 5 9 ; CG2R61 CG2R61 CG2R61 CG2R61

4 2 3 9 9 ; CG2R61 CG2R61 CG2R61 HGR61

8 2 3 5 9 ; CG2O4 CG2R61 CG2R61 CG2R61

8 2 3 9 9 ; CG2O4 CG2R61 CG2R61 HGR61

3 2 4 6 9 ; CG2R61 CG2R61 CG2R61 CG2R61

3 2 4 10 9 ; CG2R61 CG2R61 CG2R61 HGR61

8 2 4 6 9 ; CG2O4 CG2R61 CG2R61 CG2R61

8 2 4 10 9 ; CG2O4 CG2R61 CG2R61 HGR61

3 2 8 1 9 ; CG2R61 CG2R61 CG2O4 OG2D1

3 2 8 14 9 ; CG2R61 CG2R61 CG2O4 HGR52

4 2 8 1 9 ; CG2R61 CG2R61 CG2O4 OG2D1

4 2 8 14 9 ; CG2R61 CG2R61 CG2O4 HGR52

2 3 5 7 9 ; CG2R61 CG2R61 CG2R61 CG2R61

2 3 5 11 9 ; CG2R61 CG2R61 CG2R61 HGR61

9 3 5 7 9 ; HGR61 CG2R61 CG2R61 CG2R61

9 3 5 11 9 ; HGR61 CG2R61 CG2R61 HGR61

2 4 6 7 9 ; CG2R61 CG2R61 CG2R61 CG2R61

2 4 6 12 9 ; CG2R61 CG2R61 CG2R61 HGR61

10 4 6 7 9 ; HGR61 CG2R61 CG2R61 CG2R61

10 4 6 12 9 ; HGR61 CG2R61 CG2R61 HGR61

3 5 7 6 9 ; CG2R61 CG2R61 CG2R61 CG2R61

3 5 7 13 9 ; CG2R61 CG2R61 CG2R61 HGR61

11 5 7 6 9 ; HGR61 CG2R61 CG2R61 CG2R61

11 5 7 13 9 ; HGR61 CG2R61 CG2R61 HGR61

4 6 7 5 9 ; CG2R61 CG2R61 CG2R61 CG2R61

4 6 7 13 9 ; CG2R61 CG2R61 CG2R61 HGR61

12 6 7 5 9 ; HGR61 CG2R61 CG2R61 CG2R61

12 6 7 13 9 ; HGR61 CG2R61 CG2R61 HGR61

[ dihedrals ]

; ai aj ak al funct c0 c1 c2 c3

8 2 1 14 2
