# Supplementary material for: Benzaldehyde, A New Absorption Promoter, Accelerating Absorption on Low Bioavailability Drugs Through Membrane Permeability
Source: Front Pharmacol. 2021 May 28;12:663743. doi: 10.3389/fphar.2021.663743 (PMC8194254; doi:10.3389/fphar.2021.663743)
Supplement: Supplementary file 1 [file DataSheet1.zip › Supplementary file 5.DOCX]

#include "charmm36-feb2021.ff/forcefield.itp"

; additional params for the molecule

#include "benzar.prm"

#include "benzar.itp"

#include "charmm36-feb2021.ff/tip3p.itp"

#ifdef POSRES_WATER

; Position restraint for each water oxygen

[ position_restraints ]

; i funct fcx fcy fcz

1 1 1000 1000 1000

#endif

; Include topology for ions

#include "charmm36-feb2021.ff/ions.itp"

[ system ]

; Name

mol

[ molecules ]

; Compound #mols

benzar 1
