# Supplementary material for: Benzaldehyde, A New Absorption Promoter, Accelerating Absorption on Low Bioavailability Drugs Through Membrane Permeability
Source: Front Pharmacol. 2021 May 28;12:663743. doi: 10.3389/fphar.2021.663743 (PMC8194254; doi:10.3389/fphar.2021.663743)
Supplement: Supplementary file 1 [file DataSheet1.zip › Supplementary file 6.DOCX]

POPC_Ben in water

25540

1POPC N 1 3.296 3.024 5.089

1POPC C12 2 3.364 2.992 4.956

1POPC H12A 3 3.375 2.885 4.948

1POPC H12B 4 3.464 3.032 4.957

1POPC C13 5 3.170 2.945 5.101

1POPC H13A 6 3.112 2.974 5.188

1POPC H13B 7 3.109 2.964 5.013

1POPC H13C 8 3.189 2.839 5.103

1POPC C14 9 3.386 2.991 5.204

1POPC H14A 10 3.336 3.009 5.298

1POPC H14B 11 3.416 2.887 5.202

1POPC H14C 12 3.476 3.051 5.203

1POPC C15 13 3.257 3.169 5.093

1POPC H15A 14 3.188 3.189 5.012

1POPC H15B 15 3.342 3.234 5.082

1POPC H15C 16 3.206 3.193 5.184

1POPC C11 17 3.294 3.039 4.826

1POPC H11A 18 3.355 3.009 4.739

1POPC H11B 19 3.290 3.150 4.824

1POPC P 20 3.125 2.859 4.736

1POPC O13 21 3.107 2.905 4.597

1POPC O14 22 3.009 2.795 4.802

1POPC O12 23 3.160 2.988 4.820

1POPC O11 24 3.255 2.767 4.755

1POPC C1 25 3.250 2.661 4.852

1POPC HA 26 3.154 2.605 4.846

1POPC HB 27 3.258 2.704 4.954

1POPC C2 28 3.362 2.552 4.838

1POPC HS 29 3.377 2.507 4.939

1POPC O21 30 3.488 2.604 4.792

1POPC C21 31 3.540 2.703 4.858

1POPC O22 32 3.512 2.738 4.972

1POPC C22 33 3.642 2.774 4.769

1POPC H2R 34 3.734 2.712 4.762

1POPC H2S 35 3.667 2.871 4.816

1POPC C3 36 3.320 2.435 4.745

1POPC HX 37 3.229 2.388 4.787

1POPC HY 38 3.399 2.357 4.745

1POPC O31 39 3.289 2.480 4.612

1POPC C31 40 3.390 2.485 4.527

1POPC O32 41 3.506 2.455 4.552

1POPC C32 42 3.341 2.532 4.390

1POPC H2X 43 3.324 2.440 4.330

1POPC H2Y 44 3.423 2.590 4.343

1POPC C23 45 3.585 2.798 4.627

1POPC H3R 46 3.476 2.820 4.635

1POPC H3S 47 3.595 2.705 4.567

1POPC C24 48 3.650 2.915 4.552

1POPC H4R 49 3.760 2.903 4.550

1POPC H4S 50 3.628 3.010 4.605

1POPC C25 51 3.600 2.926 4.407

1POPC H5R 52 3.631 2.837 4.349

1POPC H5S 53 3.649 3.014 4.360

1POPC C26 54 3.447 2.940 4.397

1POPC H6R 55 3.414 3.029 4.455

1POPC H6S 56 3.398 2.851 4.440

1POPC C27 57 3.397 2.954 4.253

1POPC H7R 58 3.286 2.965 4.258

1POPC H7S 59 3.418 2.860 4.197

1POPC C28 60 3.459 3.072 4.176

1POPC H8R 61 3.567 3.051 4.160

1POPC H8S 62 3.453 3.163 4.240

1POPC C29 63 3.390 3.096 4.045

1POPC H91 64 3.306 3.167 4.047

1POPC C210 65 3.414 3.034 3.930

1POPC H101 66 3.356 3.067 3.842

1POPC C211 67 3.515 2.926 3.903

1POPC H11R 68 3.463 2.828 3.892

1POPC H11S 69 3.587 2.915 3.987

1POPC C212 70 3.593 2.953 3.773

1POPC H12R 71 3.665 2.870 3.759

1POPC H12S 72 3.652 3.047 3.782

1POPC C213 73 3.500 2.962 3.651

1POPC H13R 74 3.449 3.061 3.651

1POPC H13S 75 3.422 2.883 3.660

1POPC C214 76 3.572 2.945 3.517

1POPC H14R 77 3.663 3.008 3.519

1POPC H14S 78 3.506 2.982 3.436

1POPC C215 79 3.606 2.798 3.488

1POPC H15R 80 3.514 2.737 3.502

1POPC H15S 81 3.681 2.762 3.562

1POPC C216 82 3.658 2.774 3.346

1POPC H16R 83 3.581 2.807 3.273

1POPC H16S 84 3.675 2.665 3.331

1POPC C217 85 3.787 2.849 3.315

1POPC H17R 86 3.767 2.953 3.350

1POPC H17S 87 3.804 2.853 3.205

1POPC C218 88 3.914 2.797 3.383

1POPC H18R 89 3.998 2.868 3.368

1POPC H18S 90 3.943 2.698 3.340

1POPC H18T 91 3.897 2.785 3.492

1POPC C33 92 3.211 2.615 4.392

1POPC H3X 93 3.225 2.705 4.456

1POPC H3Y 94 3.128 2.555 4.435

1POPC C34 95 3.169 2.662 4.251

1POPC H4X 96 3.144 2.572 4.190

1POPC H4Y 97 3.255 2.713 4.202

1POPC C35 98 3.049 2.758 4.255

1POPC H5X 99 3.071 2.843 4.322

1POPC H5Y 100 2.964 2.702 4.299

1POPC C36 101 3.008 2.810 4.116

1POPC H6X 102 2.903 2.844 4.122

1POPC H6Y 103 3.013 2.724 4.045

1POPC C37 104 3.094 2.925 4.061

1POPC H7X 105 3.193 2.885 4.031

1POPC H7Y 106 3.110 2.997 4.144

1POPC C38 107 3.032 3.006 3.944

1POPC H8X 108 3.095 3.094 3.921

1POPC H8Y 109 2.934 3.044 3.980

1POPC C39 110 3.006 2.926 3.815

1POPC H9X 111 2.917 2.967 3.763

1POPC H9Y 112 2.982 2.823 3.848

1POPC C310 113 3.123 2.917 3.716

1POPC H10X 114 3.159 2.812 3.715

1POPC H10Y 115 3.207 2.979 3.751

1POPC C311 116 3.091 2.958 3.570

1POPC H11X 117 3.064 3.066 3.566

1POPC H11Y 118 3.002 2.901 3.535

1POPC C312 119 3.211 2.930 3.478

1POPC H12X 120 3.264 2.843 3.521

1POPC H12Y 121 3.281 3.016 3.480

1POPC C313 122 3.180 2.892 3.332

1POPC H13X 123 3.143 2.979 3.275

1POPC H13Y 124 3.100 2.815 3.332

1POPC C314 125 3.306 2.833 3.265

1POPC H14X 126 3.358 2.767 3.339

1POPC H14Y 127 3.376 2.915 3.239

1POPC C315 128 3.278 2.746 3.142

1POPC H15X 129 3.232 2.811 3.064

1POPC H15Y 130 3.203 2.669 3.169

1POPC C316 131 3.400 2.673 3.086

1POPC H16X 132 3.477 2.745 3.052

1POPC H16Y 133 3.370 2.609 3.000

1POPC H16Z 134 3.445 2.607 3.163

2POPC N 135 2.794 3.545 4.812

2POPC C12 136 2.666 3.567 4.731

2POPC H12A 137 2.659 3.488 4.658

2POPC H12B 138 2.675 3.661 4.678

2POPC C13 139 2.822 3.667 4.894

2POPC H13A 140 2.907 3.653 4.960

2POPC H13B 141 2.848 3.752 4.832

2POPC H13C 142 2.734 3.692 4.951

2POPC C14 143 2.773 3.428 4.902

2POPC H14A 144 2.690 3.448 4.968

2POPC H14B 145 2.749 3.340 4.843

2POPC H14C 146 2.861 3.405 4.960

2POPC C15 147 2.912 3.519 4.723

2POPC H15A 148 2.899 3.426 4.669

2POPC H15B 149 3.002 3.511 4.782

2POPC H15C 150 2.927 3.599 4.653

2POPC C11 151 2.532 3.570 4.810

2POPC H11A 152 2.504 3.465 4.836

2POPC H11B 153 2.453 3.610 4.743

2POPC P 154 2.418 3.677 5.022

2POPC O13 155 2.470 3.741 5.145

2POPC O14 156 2.338 3.553 5.041

2POPC O12 157 2.542 3.648 4.929

2POPC O11 158 2.335 3.784 4.942

2POPC C1 159 2.399 3.897 4.881

2POPC HA 160 2.504 3.877 4.853

2POPC HB 161 2.396 3.984 4.950

2POPC C2 162 2.327 3.936 4.749

2POPC HS 163 2.224 3.969 4.776

2POPC O21 164 2.322 3.820 4.664

2POPC C21 165 2.243 3.836 4.560

2POPC O22 166 2.165 3.927 4.543

2POPC C22 167 2.271 3.723 4.462

2POPC H2R 168 2.240 3.626 4.507

2POPC H2S 169 2.381 3.722 4.443

2POPC C3 170 2.399 4.055 4.676

2POPC HX 171 2.419 4.133 4.752

2POPC HY 172 2.331 4.098 4.599

2POPC O31 173 2.520 4.007 4.613

2POPC C31 174 2.603 4.103 4.573

2POPC O32 175 2.592 4.221 4.603

2POPC C32 176 2.718 4.045 4.491

2POPC H2X 177 2.804 4.031 4.559

2POPC H2Y 178 2.746 4.121 4.415

2POPC C23 179 2.198 3.748 4.329

2POPC H3R 180 2.217 3.853 4.301

2POPC H3S 181 2.088 3.737 4.341

2POPC C24 182 2.248 3.655 4.217

2POPC H4R 183 2.212 3.552 4.235

2POPC H4S 184 2.359 3.655 4.220

2POPC C25 185 2.206 3.703 4.078

2POPC H5R 186 2.095 3.701 4.068

2POPC H5S 187 2.248 3.634 4.002

2POPC C26 188 2.257 3.845 4.050

2POPC H6R 189 2.365 3.850 4.076

2POPC H6S 190 2.203 3.920 4.112

2POPC C27 191 2.240 3.885 3.905

2POPC H7R 192 2.131 3.900 3.886

2POPC H7S 193 2.275 3.803 3.840

2POPC C28 194 2.318 4.013 3.876

2POPC H8R 195 2.425 3.995 3.900

2POPC H8S 196 2.286 4.095 3.945

2POPC C29 197 2.298 4.050 3.732

2POPC H91 198 2.216 4.120 3.711

2POPC C210 199 2.370 4.000 3.632

2POPC H101 200 2.345 4.026 3.528

2POPC C211 201 2.483 3.902 3.647

2POPC H11R 202 2.506 3.857 3.548

2POPC H11S 203 2.450 3.820 3.714

2POPC C212 204 2.613 3.961 3.704

2POPC H12R 205 2.664 3.882 3.763

2POPC H12S 206 2.591 4.045 3.774

2POPC C213 207 2.709 4.009 3.595

2POPC H13R 208 2.732 3.922 3.530

2POPC H13S 209 2.804 4.043 3.641

2POPC C214 210 2.651 4.122 3.510

2POPC H14R 211 2.628 4.207 3.578

2POPC H14S 212 2.556 4.090 3.463

2POPC C215 213 2.744 4.171 3.400

2POPC H15R 214 2.828 4.222 3.451

2POPC H15S 215 2.698 4.250 3.338

2POPC C216 216 2.790 4.062 3.302

2POPC H16R 217 2.842 3.980 3.357

2POPC H16S 218 2.863 4.107 3.232

2POPC C217 219 2.676 4.000 3.221

2POPC H17R 220 2.610 4.081 3.183

2POPC H17S 221 2.612 3.938 3.288

2POPC C218 222 2.726 3.918 3.102

2POPC H18R 223 2.795 3.979 3.040

2POPC H18S 224 2.640 3.886 3.039

2POPC H18T 225 2.780 3.828 3.137

2POPC C33 226 2.687 3.910 4.419

2POPC H3X 227 2.612 3.927 4.339

2POPC H3Y 228 2.645 3.836 4.490

2POPC C34 229 2.816 3.853 4.357

2POPC H4X 230 2.887 3.825 4.438

2POPC H4Y 231 2.863 3.933 4.297

2POPC C35 232 2.795 3.732 4.263

2POPC H5X 233 2.893 3.717 4.212

2POPC H5Y 234 2.721 3.757 4.185

2POPC C36 235 2.758 3.601 4.333

2POPC H6X 236 2.650 3.601 4.359

2POPC H6Y 237 2.815 3.595 4.428

2POPC C37 238 2.793 3.477 4.249

2POPC H7X 239 2.863 3.414 4.308

2POPC H7Y 240 2.849 3.508 4.158

2POPC C38 241 2.674 3.389 4.207

2POPC H8X 242 2.621 3.357 4.299

2POPC H8Y 243 2.712 3.298 4.157

2POPC C39 244 2.574 3.457 4.113

2POPC H9X 245 2.542 3.555 4.155

2POPC H9Y 246 2.483 3.393 4.106

2POPC C310 247 2.625 3.480 3.970

2POPC H10X 248 2.666 3.385 3.929

2POPC H10Y 249 2.707 3.555 3.972

2POPC C311 250 2.513 3.532 3.878

2POPC H11X 251 2.468 3.620 3.927

2POPC H11Y 252 2.434 3.454 3.869

2POPC C312 253 2.559 3.575 3.738

2POPC H12X 254 2.634 3.656 3.748

2POPC H12Y 255 2.469 3.616 3.687

2POPC C313 256 2.616 3.465 3.648

2POPC H13X 257 2.538 3.388 3.629

2POPC H13Y 258 2.702 3.415 3.699

2POPC C314 259 2.666 3.520 3.513

2POPC H14X 260 2.713 3.438 3.454

2POPC H14Y 261 2.745 3.596 3.532

2POPC C315 262 2.557 3.584 3.427

2POPC H15X 263 2.504 3.662 3.486

2POPC H15Y 264 2.481 3.507 3.401

2POPC C316 265 2.610 3.649 3.299

2POPC H16X 266 2.650 3.573 3.229

2POPC H16Y 267 2.692 3.720 3.325

2POPC H16Z 268 2.530 3.707 3.248

3POPC N 269 3.491 4.079 5.030

3POPC C12 270 3.499 3.933 4.986

3POPC H12A 271 3.552 3.878 5.062

3POPC H12B 272 3.556 3.929 4.894

3POPC C13 273 3.448 4.161 4.911

3POPC H13A 274 3.521 4.158 4.832

3POPC H13B 275 3.359 4.116 4.870

3POPC H13C 276 3.427 4.263 4.937

3POPC C14 277 3.387 4.093 5.137

3POPC H14A 278 3.294 4.051 5.103

3POPC H14B 279 3.414 4.039 5.227

3POPC H14C 280 3.370 4.196 5.164

3POPC C15 281 3.624 4.127 5.079

3POPC H15A 282 3.619 4.231 5.107

3POPC H15B 283 3.700 4.117 5.003

3POPC H15C 284 3.655 4.071 5.166

3POPC C11 285 3.365 3.861 4.960

3POPC H11A 286 3.308 3.850 5.055

3POPC H11B 287 3.389 3.758 4.925

3POPC P 288 3.163 3.861 4.798

3POPC O13 289 3.096 3.955 4.706

3POPC O14 290 3.082 3.806 4.909

3POPC O12 291 3.288 3.932 4.864

3POPC O11 292 3.233 3.743 4.717

3POPC C1 293 3.357 3.768 4.647

3POPC HA 294 3.441 3.723 4.704

3POPC HB 295 3.380 3.876 4.635

3POPC C2 296 3.359 3.704 4.505

3POPC HS 297 3.464 3.705 4.469

3POPC O21 298 3.272 3.775 4.415

3POPC C21 299 3.326 3.887 4.369

3POPC O22 300 3.441 3.922 4.389

3POPC C22 301 3.221 3.962 4.289

3POPC H2R 302 3.144 3.997 4.361

3POPC H2S 303 3.174 3.892 4.217

3POPC C3 304 3.317 3.555 4.509

3POPC HX 305 3.208 3.548 4.532

3POPC HY 306 3.372 3.507 4.593

3POPC O31 307 3.356 3.490 4.387

3POPC C31 308 3.261 3.481 4.294

3POPC O32 309 3.143 3.501 4.310

3POPC C32 310 3.324 3.426 4.166

3POPC H2X 311 3.286 3.322 4.160

3POPC H2Y 312 3.435 3.424 4.174

3POPC C23 313 3.281 4.082 4.213

3POPC H3R 314 3.346 4.045 4.131

3POPC H3S 315 3.345 4.144 4.280

3POPC C24 316 3.170 4.172 4.157

3POPC H4R 317 3.120 4.222 4.243

3POPC H4S 318 3.094 4.110 4.105

3POPC C25 319 3.224 4.280 4.062

3POPC H5R 320 3.307 4.334 4.112

3POPC H5S 321 3.144 4.353 4.042

3POPC C26 322 3.274 4.225 3.928

3POPC H6R 323 3.362 4.158 3.942

3POPC H6S 324 3.309 4.312 3.868

3POPC C27 325 3.165 4.148 3.852

3POPC H7R 326 3.067 4.199 3.866

3POPC H7S 327 3.155 4.046 3.897

3POPC C28 328 3.193 4.133 3.702

3POPC H8R 329 3.122 4.059 3.662

3POPC H8S 330 3.296 4.093 3.688

3POPC C29 331 3.179 4.266 3.634

3POPC H91 332 3.215 4.353 3.691

3POPC C210 333 3.126 4.287 3.513

3POPC H101 334 3.123 4.390 3.474

3POPC C211 335 3.073 4.182 3.421

3POPC H11R 336 3.022 4.231 3.335

3POPC H11S 337 2.996 4.128 3.480

3POPC C212 338 3.169 4.074 3.368

3POPC H12R 339 3.272 4.103 3.394

3POPC H12S 340 3.164 4.069 3.257

3POPC C213 341 3.139 3.933 3.434

3POPC H13R 342 3.045 3.965 3.383

3POPC H13S 343 3.229 3.892 3.385

3POPC C214 344 3.062 3.802 3.394

3POPC H14R 345 2.959 3.800 3.434

3POPC H14S 346 3.115 3.716 3.442

3POPC C215 347 3.058 3.781 3.243

3POPC H15R 348 3.160 3.793 3.200

3POPC H15S 349 2.995 3.859 3.197

3POPC C216 350 2.999 3.644 3.211

3POPC H16R 351 2.941 3.655 3.118

3POPC H16S 352 2.928 3.612 3.290

3POPC C217 353 3.106 3.536 3.191

3POPC H17R 354 3.057 3.439 3.169

3POPC H17S 355 3.165 3.523 3.284

3POPC C218 356 3.200 3.571 3.076

3POPC H18R 357 3.149 3.628 2.996

3POPC H18S 358 3.243 3.478 3.032

3POPC H18T 359 3.282 3.638 3.112

3POPC C33 360 3.281 3.501 4.038

3POPC H3X 361 3.171 3.519 4.038

3POPC H3Y 362 3.304 3.438 3.950

3POPC C34 363 3.355 3.634 4.018

3POPC H4X 364 3.464 3.614 4.006

3POPC H4Y 365 3.342 3.699 4.108

3POPC C35 366 3.303 3.710 3.895

3POPC H5X 367 3.195 3.732 3.910

3POPC H5Y 368 3.310 3.645 3.805

3POPC C36 369 3.375 3.843 3.867

3POPC H6X 370 3.360 3.911 3.954

3POPC H6Y 371 3.326 3.891 3.780

3POPC C37 372 3.526 3.832 3.839

3POPC H7X 373 3.578 3.805 3.934

3POPC H7Y 374 3.564 3.932 3.809

3POPC C38 375 3.563 3.729 3.731

3POPC H8X 376 3.526 3.629 3.762

3POPC H8Y 377 3.674 3.722 3.723

3POPC C39 378 3.508 3.761 3.592

3POPC H9X 379 3.400 3.789 3.599

3POPC H9Y 380 3.513 3.670 3.529

3POPC C310 381 3.586 3.872 3.520

3POPC H10X 382 3.687 3.834 3.493

3POPC H10Y 383 3.598 3.959 3.588

3POPC C311 384 3.513 3.919 3.395

3POPC H11X 385 3.420 3.970 3.429

3POPC H11Y 386 3.483 3.831 3.334

3POPC C312 387 3.594 4.017 3.310

3POPC H12X 388 3.690 3.968 3.279

3POPC H12Y 389 3.619 4.105 3.372

3POPC C313 390 3.518 4.063 3.184

3POPC H13X 391 3.576 4.145 3.137

3POPC H13Y 392 3.419 4.104 3.213

3POPC C314 393 3.499 3.952 3.080

3POPC H14X 394 3.429 3.875 3.118

3POPC H14Y 395 3.597 3.904 3.063

3POPC C315 396 3.447 4.006 2.947

3POPC H15X 397 3.467 3.928 2.870

3POPC H15Y 398 3.506 4.096 2.918

3POPC C316 399 3.297 4.040 2.946

3POPC H16X 400 3.237 3.951 2.972

3POPC H16Y 401 3.266 4.076 2.846

3POPC H16Z 402 3.275 4.120 3.020

4POPC N 403 3.833 3.483 5.064

4POPC C12 404 3.769 3.439 4.933

4POPC H12A 405 3.742 3.527 4.876

4POPC H12B 406 3.679 3.384 4.954

4POPC C13 407 3.953 3.569 5.036

4POPC H13A 408 4.026 3.509 4.983

4POPC H13B 409 3.931 3.652 4.970

4POPC H13C 410 4.000 3.605 5.126

4POPC C14 411 3.737 3.559 5.149

4POPC H14A 412 3.785 3.586 5.242

4POPC H14B 413 3.706 3.650 5.100

4POPC H14C 414 3.650 3.500 5.174

4POPC C15 415 3.884 3.364 5.141

4POPC H15A 416 3.809 3.288 5.156

4POPC H15B 417 3.920 3.394 5.238

4POPC H15C 418 3.966 3.320 5.087

4POPC C11 419 3.854 3.353 4.836

4POPC H11A 420 3.790 3.319 4.751

4POPC H11B 421 3.886 3.261 4.889

4POPC P 422 4.106 3.366 4.782

4POPC O13 423 4.102 3.244 4.699

4POPC O14 424 4.143 3.348 4.924

4POPC O12 425 3.961 3.431 4.782

4POPC O11 426 4.201 3.476 4.721

4POPC C1 427 4.238 3.484 4.583

4POPC HA 428 4.224 3.388 4.528

4POPC HB 429 4.346 3.510 4.576

4POPC C2 430 4.158 3.595 4.511

4POPC HS 431 4.160 3.683 4.579

4POPC O21 432 4.025 3.544 4.490

4POPC C21 433 3.927 3.621 4.528

4POPC O22 434 3.936 3.738 4.563

4POPC C22 435 3.797 3.541 4.529

4POPC H2R 436 3.749 3.562 4.627

4POPC H2S 437 3.822 3.432 4.526

4POPC C3 438 4.220 3.640 4.376

4POPC HX 439 4.320 3.685 4.395

4POPC HY 440 4.154 3.720 4.336

4POPC O31 441 4.234 3.529 4.284

4POPC C31 442 4.234 3.564 4.156

4POPC O32 443 4.225 3.677 4.113

4POPC C32 444 4.240 3.437 4.072

4POPC H2X 445 4.149 3.378 4.094

4POPC H2Y 446 4.329 3.378 4.103

4POPC C23 447 3.701 3.578 4.415

4POPC H3R 448 3.683 3.687 4.410

4POPC H3S 449 3.604 3.529 4.438

4POPC C24 450 3.745 3.524 4.278

4POPC H4R 451 3.653 3.502 4.220

4POPC H4S 452 3.801 3.429 4.291

4POPC C25 453 3.830 3.624 4.198

4POPC H5R 454 3.920 3.653 4.256

4POPC H5S 455 3.771 3.716 4.179

4POPC C26 456 3.875 3.565 4.064

4POPC H6R 457 3.932 3.471 4.083

4POPC H6S 458 3.945 3.636 4.014

4POPC C27 459 3.758 3.536 3.969

4POPC H7R 460 3.700 3.630 3.958

4POPC H7S 461 3.690 3.460 4.012

4POPC C28 462 3.804 3.492 3.830

4POPC H8R 463 3.879 3.564 3.791

4POPC H8S 464 3.715 3.497 3.763

4POPC C29 465 3.861 3.352 3.833

4POPC H91 466 3.909 3.322 3.927

4POPC C210 467 3.850 3.264 3.733

4POPC H101 468 3.894 3.164 3.745

4POPC C211 469 3.787 3.292 3.599

4POPC H11R 470 3.765 3.399 3.583

4POPC H11S 471 3.691 3.237 3.591

4POPC C212 472 3.880 3.246 3.486

4POPC H12R 473 3.902 3.138 3.502

4POPC H12S 474 3.976 3.301 3.488

4POPC C213 475 3.812 3.264 3.350

4POPC H13R 476 3.826 3.367 3.312

4POPC H13S 477 3.703 3.248 3.364

4POPC C214 478 3.859 3.159 3.249

4POPC H14R 479 3.770 3.119 3.197

4POPC H14S 480 3.911 3.076 3.302

4POPC C215 481 3.958 3.208 3.146

4POPC H15R 482 3.945 3.317 3.129

4POPC H15S 483 3.930 3.155 3.052

4POPC C216 484 4.102 3.167 3.177

4POPC H16R 485 4.111 3.057 3.174

4POPC H16S 486 4.130 3.201 3.279

4POPC C217 487 4.197 3.228 3.075

4POPC H17R 488 4.161 3.208 2.972

4POPC H17S 489 4.297 3.179 3.083

4POPC C218 490 4.210 3.379 3.096

4POPC H18R 491 4.274 3.427 3.019

4POPC H18S 492 4.252 3.399 3.196

4POPC H18T 493 4.111 3.428 3.088

4POPC C33 494 4.247 3.465 3.920

4POPC H3X 495 4.344 3.514 3.896

4POPC H3Y 496 4.165 3.534 3.890

4POPC C34 497 4.232 3.333 3.842

4POPC H4X 498 4.133 3.290 3.866

4POPC H4Y 499 4.311 3.264 3.881

4POPC C35 500 4.249 3.339 3.690

4POPC H5X 501 4.198 3.252 3.644

4POPC H5Y 502 4.357 3.328 3.667

4POPC C36 503 4.197 3.465 3.621

4POPC H6X 504 4.234 3.557 3.672

4POPC H6Y 505 4.086 3.467 3.624

4POPC C37 506 4.248 3.467 3.477

4POPC H7X 507 4.224 3.370 3.427

4POPC H7Y 508 4.358 3.476 3.481

4POPC C38 509 4.192 3.583 3.396

4POPC H8X 510 4.260 3.592 3.308

4POPC H8Y 511 4.198 3.678 3.452

4POPC C39 512 4.051 3.561 3.339

4POPC H9X 513 3.975 3.579 3.418

4POPC H9Y 514 4.040 3.456 3.305

4POPC C310 515 4.033 3.656 3.221

4POPC H10X 516 4.116 3.640 3.148

4POPC H10Y 517 4.041 3.759 3.261

4POPC C311 518 3.902 3.651 3.145

4POPC H11X 519 3.821 3.663 3.219

4POPC H11Y 520 3.890 3.552 3.096

4POPC C312 521 3.893 3.765 3.041

4POPC H12X 522 3.914 3.861 3.093

4POPC H12Y 523 3.791 3.775 3.000

4POPC C313 524 3.990 3.753 2.922

4POPC H13X 525 4.093 3.770 2.960

4POPC H13Y 526 3.968 3.835 2.850

4POPC C314 527 3.993 3.618 2.848

4POPC H14X 528 4.030 3.538 2.917

4POPC H14Y 529 4.067 3.625 2.765

4POPC C315 530 3.859 3.576 2.790

4POPC H15X 531 3.790 3.550 2.874

4POPC H15Y 532 3.872 3.484 2.730

4POPC C316 533 3.795 3.684 2.703

4POPC H16X 534 3.693 3.650 2.675

4POPC H16Y 535 3.855 3.702 2.611

4POPC H16Z 536 3.782 3.780 2.758

5POPC N 537 2.199 2.914 4.940

5POPC C12 538 2.267 2.819 5.038

5POPC H12A 539 2.230 2.719 5.020

5POPC H12B 540 2.239 2.846 5.139

5POPC C13 541 2.214 2.863 4.800

5POPC H13A 542 2.318 2.842 4.780

5POPC H13B 543 2.163 2.769 4.786

5POPC H13C 544 2.178 2.934 4.726

5POPC C14 545 2.054 2.923 4.972

5POPC H14A 546 2.037 2.952 5.075

5POPC H14B 547 2.004 2.994 4.908

5POPC H14C 548 2.005 2.827 4.959

5POPC C15 549 2.264 3.050 4.946

5POPC H15A 550 2.254 3.096 5.043

5POPC H15B 551 2.222 3.118 4.873

5POPC H15C 552 2.369 3.040 4.924

5POPC C11 553 2.422 2.808 5.034

5POPC H11A 554 2.456 2.745 5.119

5POPC H11B 555 2.466 2.908 5.049

5POPC P 556 2.438 2.603 4.875

5POPC O13 557 2.327 2.555 4.961

5POPC O14 558 2.563 2.525 4.880

5POPC O12 559 2.469 2.755 4.910

5POPC O11 560 2.384 2.619 4.728

5POPC C1 561 2.464 2.667 4.619

5POPC HA 562 2.573 2.661 4.641

5POPC HB 563 2.439 2.773 4.598

5POPC C2 564 2.439 2.584 4.490

5POPC HS 565 2.519 2.609 4.417

5POPC O21 566 2.309 2.619 4.440

5POPC C21 567 2.293 2.614 4.309

5POPC O22 568 2.375 2.577 4.227

5POPC C22 569 2.153 2.669 4.278

5POPC H2R 570 2.150 2.775 4.311

5POPC H2S 571 2.081 2.611 4.339

5POPC C3 572 2.451 2.432 4.527

5POPC HX 573 2.385 2.415 4.615

5POPC HY 574 2.555 2.414 4.563

5POPC O31 575 2.410 2.340 4.425

5POPC C31 576 2.505 2.314 4.335

5POPC O32 577 2.612 2.370 4.328

5POPC C32 578 2.459 2.192 4.253

5POPC H2X 579 2.348 2.194 4.248

5POPC H2Y 580 2.491 2.101 4.309

5POPC C23 581 2.110 2.663 4.129

5POPC H3R 582 2.123 2.560 4.090

5POPC H3S 583 2.172 2.730 4.067

5POPC C24 584 1.962 2.703 4.117

5POPC H4R 585 1.953 2.811 4.142

5POPC H4S 586 1.903 2.646 4.193

5POPC C25 587 1.898 2.680 3.980

5POPC H5R 588 1.969 2.713 3.902

5POPC H5S 589 1.807 2.743 3.971

5POPC C26 590 1.855 2.533 3.959

5POPC H6R 591 1.779 2.506 4.036

5POPC H6S 592 1.943 2.468 3.977

5POPC C27 593 1.798 2.502 3.819

5POPC H7R 594 1.698 2.550 3.809

5POPC H7S 595 1.781 2.393 3.811

5POPC C28 596 1.885 2.550 3.702

5POPC H8R 597 1.893 2.661 3.703

5POPC H8S 598 1.835 2.523 3.607

5POPC C29 599 2.021 2.488 3.716

5POPC H91 600 2.072 2.507 3.811

5POPC C210 601 2.086 2.414 3.625

5POPC H101 602 2.187 2.377 3.649

5POPC C211 603 2.042 2.379 3.487

5POPC H11R 604 1.933 2.396 3.470

5POPC H11S 605 2.063 2.272 3.465

5POPC C212 606 2.120 2.471 3.394

5POPC H12R 607 2.101 2.576 3.427

5POPC H12S 608 2.083 2.462 3.290

5POPC C213 609 2.272 2.448 3.391

5POPC H13R 610 2.292 2.343 3.360

5POPC H13S 611 2.311 2.458 3.495

5POPC C214 612 2.345 2.551 3.304

5POPC H14R 613 2.323 2.652 3.346

5POPC H14S 614 2.307 2.547 3.200

5POPC C215 615 2.497 2.533 3.303

5POPC H15R 616 2.516 2.436 3.252

5POPC H15S 617 2.536 2.524 3.406

5POPC C216 618 2.576 2.642 3.230

5POPC H16R 619 2.549 2.642 3.122

5POPC H16S 620 2.684 2.617 3.237

5POPC C217 621 2.557 2.783 3.287

5POPC H17R 622 2.539 2.774 3.397

5POPC H17S 623 2.467 2.828 3.241

5POPC C218 624 2.676 2.877 3.265

5POPC H18R 625 2.768 2.820 3.239

5POPC H18S 626 2.697 2.937 3.356

5POPC H18T 627 2.656 2.948 3.182

5POPC C33 628 2.516 2.188 4.110

5POPC H3X 629 2.468 2.104 4.055

5POPC H3Y 630 2.625 2.167 4.113

5POPC C34 631 2.491 2.318 4.035

5POPC H4X 632 2.558 2.396 4.078

5POPC H4Y 633 2.388 2.356 4.050

5POPC C35 634 2.519 2.309 3.885

5POPC H5X 635 2.443 2.248 3.833

5POPC H5Y 636 2.618 2.259 3.872

5POPC C36 637 2.529 2.449 3.826

5POPC H6X 638 2.632 2.456 3.786

5POPC H6Y 639 2.516 2.525 3.907

5POPC C37 640 2.435 2.485 3.713

5POPC H7X 641 2.331 2.487 3.751

5POPC H7Y 642 2.444 2.404 3.638

5POPC C38 643 2.469 2.619 3.645

5POPC H8X 644 2.402 2.633 3.558

5POPC H8Y 645 2.572 2.616 3.605

5POPC C39 646 2.458 2.746 3.732

5POPC H9X 647 2.492 2.832 3.671

5POPC H9Y 648 2.527 2.738 3.819

5POPC C310 649 2.315 2.776 3.782

5POPC H10X 650 2.317 2.875 3.833

5POPC H10Y 651 2.289 2.700 3.859

5POPC C311 652 2.206 2.777 3.674

5POPC H11X 653 2.109 2.805 3.720

5POPC H11Y 654 2.196 2.673 3.634

5POPC C312 655 2.234 2.867 3.553

5POPC H12X 656 2.327 2.832 3.504

5POPC H12Y 657 2.253 2.971 3.587

5POPC C313 658 2.120 2.866 3.450

5POPC H13X 659 2.048 2.948 3.473

5POPC H13Y 660 2.063 2.771 3.463

5POPC C314 661 2.167 2.875 3.304

5POPC H14X 662 2.277 2.861 3.302

5POPC H14Y 663 2.148 2.978 3.265

5POPC C315 664 2.105 2.771 3.210

5POPC H15X 665 1.995 2.781 3.208

5POPC H15Y 666 2.123 2.670 3.253

5POPC C316 667 2.162 2.765 3.067

5POPC H16X 668 2.144 2.861 3.014

5POPC H16Y 669 2.113 2.684 3.009

5POPC H16Z 670 2.271 2.746 3.069

6POPC N 671 2.810 2.226 5.103

6POPC C12 672 2.912 2.318 5.036

6POPC H12A 673 3.005 2.264 5.026

6POPC H12B 674 2.928 2.405 5.097

6POPC C13 675 2.672 2.279 5.074

6POPC H13A 676 2.595 2.214 5.113

6POPC H13B 677 2.658 2.377 5.117

6POPC H13C 678 2.660 2.291 4.968

6POPC C14 679 2.824 2.088 5.046

6POPC H14A 680 2.808 2.092 4.939

6POPC H14B 681 2.924 2.050 5.058

6POPC H14C 682 2.755 2.018 5.090

6POPC C15 683 2.831 2.221 5.251

6POPC H15A 684 2.755 2.161 5.298

6POPC H15B 685 2.825 2.319 5.295

6POPC H15C 686 2.927 2.177 5.275

6POPC C11 687 2.880 2.371 4.893

6POPC H11A 688 2.963 2.436 4.859

6POPC H11B 689 2.790 2.435 4.895

6POPC P 690 2.978 2.179 4.750

6POPC O13 691 2.921 2.051 4.703

6POPC O14 692 3.081 2.174 4.857

6POPC O12 693 2.857 2.265 4.801

6POPC O11 694 3.036 2.263 4.629

6POPC C1 695 2.962 2.284 4.509

6POPC HA 696 2.973 2.390 4.475

6POPC HB 697 2.853 2.267 4.523

6POPC C2 698 3.011 2.192 4.393

6POPC HS 699 3.038 2.095 4.440

6POPC O21 700 3.121 2.255 4.321

6POPC C21 701 3.198 2.165 4.260

6POPC O22 702 3.204 2.047 4.290

6POPC C22 703 3.273 2.224 4.140

6POPC H2R 704 3.339 2.141 4.105

6POPC H2S 705 3.338 2.307 4.174

6POPC C3 706 2.897 2.168 4.292

6POPC HX 707 2.810 2.124 4.345

6POPC HY 708 2.931 2.096 4.215

6POPC O31 709 2.861 2.297 4.239

6POPC C31 710 2.854 2.310 4.109

6POPC O32 711 2.856 2.219 4.028

6POPC C32 712 2.850 2.459 4.076

6POPC H2X 713 2.921 2.513 4.142

6POPC H2Y 714 2.747 2.494 4.101

6POPC C23 715 3.181 2.267 4.024

6POPC H3R 716 3.142 2.369 4.045

6POPC H3S 717 3.094 2.198 4.018

6POPC C24 718 3.248 2.265 3.886

6POPC H4R 719 3.357 2.287 3.897

6POPC H4S 720 3.205 2.345 3.821

6POPC C25 721 3.230 2.131 3.813

6POPC H5R 722 3.248 2.049 3.886

6POPC H5S 723 3.305 2.122 3.732

6POPC C26 724 3.090 2.112 3.751

6POPC H6R 725 3.082 2.174 3.659

6POPC H6S 726 3.011 2.146 3.821

6POPC C27 727 3.062 1.964 3.716

6POPC H7R 728 3.059 1.904 3.809

6POPC H7S 729 3.148 1.927 3.656

6POPC C28 730 2.935 1.942 3.633

6POPC H8R 731 2.935 1.837 3.596

6POPC H8S 732 2.941 2.007 3.543

6POPC C29 733 2.809 1.966 3.712

6POPC H91 734 2.796 1.901 3.800

6POPC C210 735 2.716 2.059 3.686

6POPC H101 736 2.627 2.067 3.751

6POPC C211 737 2.719 2.158 3.572

6POPC H11R 738 2.823 2.184 3.543

6POPC H11S 739 2.671 2.253 3.604

6POPC C212 740 2.646 2.104 3.449

6POPC H12R 741 2.541 2.078 3.477

6POPC H12S 742 2.695 2.012 3.411

6POPC C213 743 2.639 2.208 3.336

6POPC H13R 744 2.616 2.306 3.382

6POPC H13S 745 2.556 2.181 3.268

6POPC C214 746 2.770 2.219 3.256

6POPC H14R 747 2.795 2.120 3.214

6POPC H14S 748 2.852 2.249 3.324

6POPC C215 749 2.762 2.319 3.140

6POPC H15R 750 2.662 2.369 3.137

6POPC H15S 751 2.770 2.262 3.045

6POPC C216 752 2.870 2.427 3.143

6POPC H16R 753 2.968 2.380 3.165

6POPC H16S 754 2.852 2.499 3.226

6POPC C217 755 2.878 2.503 3.010

6POPC H17R 756 2.793 2.574 3.008

6POPC H17S 757 2.864 2.432 2.926

6POPC C218 758 3.009 2.577 2.990

6POPC H18R 759 3.013 2.619 2.887

6POPC H18S 760 3.096 2.509 3.002

6POPC H18T 761 3.018 2.660 3.063

6POPC C33 762 2.881 2.487 3.927

6POPC H3X 763 2.843 2.402 3.867

6POPC H3Y 764 2.990 2.495 3.911

6POPC C34 765 2.811 2.614 3.875

6POPC H4X 766 2.845 2.702 3.933

6POPC H4Y 767 2.703 2.609 3.899

6POPC C35 768 2.832 2.640 3.725

6POPC H5X 769 2.934 2.684 3.717

6POPC H5Y 770 2.761 2.718 3.690

6POPC C36 771 2.830 2.519 3.630

6POPC H6X 772 2.727 2.483 3.610

6POPC H6Y 773 2.887 2.434 3.672

6POPC C37 774 2.896 2.551 3.496

6POPC H7X 775 2.842 2.632 3.443

6POPC H7Y 776 2.890 2.459 3.434

6POPC C38 777 3.043 2.590 3.518

6POPC H8X 778 3.081 2.541 3.610

6POPC H8Y 779 3.048 2.700 3.535

6POPC C39 780 3.133 2.552 3.402

6POPC H9X 781 3.229 2.607 3.413

6POPC H9Y 782 3.084 2.582 3.307

6POPC C310 783 3.161 2.403 3.403

6POPC H10X 784 3.069 2.350 3.371

6POPC H10Y 785 3.182 2.370 3.507

6POPC C311 786 3.281 2.367 3.317

6POPC H11X 787 3.369 2.426 3.350

6POPC H11Y 788 3.261 2.393 3.210

6POPC C312 789 3.312 2.218 3.330

6POPC H12X 790 3.320 2.193 3.438

6POPC H12Y 791 3.410 2.198 3.282

6POPC C313 792 3.203 2.133 3.264

6POPC H13X 793 3.237 2.108 3.162

6POPC H13Y 794 3.108 2.191 3.255

6POPC C314 795 3.167 2.005 3.338

6POPC H14X 796 3.167 2.022 3.447

6POPC H14Y 797 3.248 1.933 3.312

6POPC C315 798 3.032 1.946 3.295

6POPC H15X 799 2.954 2.020 3.322

6POPC H15Y 800 3.012 1.853 3.352

6POPC C316 801 3.021 1.915 3.146

6POPC H16X 802 3.059 1.813 3.121

6POPC H16Y 803 3.080 1.987 3.085

6POPC H16Z 804 2.915 1.920 3.112

7POPC N 805 2.701 4.306 4.974

7POPC C12 806 2.645 4.370 4.847

7POPC H12A 807 2.604 4.295 4.781

7POPC H12B 808 2.727 4.415 4.793

7POPC C13 809 2.726 4.159 4.958

7POPC H13A 810 2.797 4.139 4.879

7POPC H13B 811 2.763 4.117 5.050

7POPC H13C 812 2.634 4.107 4.937

7POPC C14 813 2.830 4.376 5.005

7POPC H14A 814 2.904 4.356 4.930

7POPC H14B 815 2.813 4.482 5.001

7POPC H14C 816 2.867 4.349 5.103

7POPC C15 817 2.605 4.327 5.087

7POPC H15A 818 2.513 4.273 5.070

7POPC H15B 819 2.580 4.432 5.091

7POPC H15C 820 2.645 4.298 5.183

7POPC C11 821 2.538 4.481 4.862

7POPC H11A 822 2.441 4.435 4.890

7POPC H11B 823 2.522 4.528 4.763

7POPC P 824 2.668 4.698 4.931

7POPC O13 825 2.798 4.641 4.887

7POPC O14 826 2.670 4.782 5.053

7POPC O12 827 2.572 4.577 4.963

7POPC O11 828 2.592 4.770 4.813

7POPC C1 829 2.452 4.796 4.826

7POPC HA 830 2.435 4.864 4.912

7POPC HB 831 2.394 4.704 4.845

7POPC C2 832 2.395 4.867 4.700

7POPC HS 833 2.289 4.890 4.723

7POPC O21 834 2.400 4.787 4.580

7POPC C21 835 2.321 4.681 4.579

7POPC O22 836 2.247 4.647 4.670

7POPC C22 837 2.338 4.608 4.445

7POPC H2R 838 2.441 4.628 4.409

7POPC H2S 839 2.266 4.653 4.374

7POPC C3 840 2.466 5.004 4.678

7POPC HX 841 2.500 5.042 4.777

7POPC HY 842 2.387 5.073 4.642

7POPC O31 843 2.571 5.004 4.581

7POPC C31 844 2.696 5.014 4.625

7POPC O32 845 2.731 5.019 4.742

7POPC C32 846 2.789 5.021 4.504

7POPC H2X 847 2.883 4.968 4.530

7POPC H2Y 848 2.813 5.129 4.488

7POPC C23 849 2.315 4.455 4.453

7POPC H3R 850 2.206 4.432 4.445

7POPC H3S 851 2.350 4.417 4.552

7POPC C24 852 2.393 4.377 4.345

7POPC H4R 853 2.379 4.268 4.358

7POPC H4S 854 2.501 4.399 4.362

7POPC C25 855 2.357 4.416 4.201

7POPC H5R 856 2.327 4.523 4.198

7POPC H5S 857 2.270 4.357 4.166

7POPC C26 858 2.476 4.400 4.105

7POPC H6R 859 2.491 4.293 4.078

7POPC H6S 860 2.568 4.435 4.157

7POPC C27 861 2.463 4.487 3.979

7POPC H7R 862 2.562 4.491 3.930

7POPC H7S 863 2.436 4.589 4.013

7POPC C28 864 2.359 4.438 3.879

7POPC H8R 865 2.260 4.433 3.929

7POPC H8S 866 2.384 4.334 3.849

7POPC C29 867 2.353 4.534 3.762

7POPC H91 868 2.328 4.637 3.790

7POPC C210 869 2.373 4.506 3.633

7POPC H101 870 2.363 4.586 3.558

7POPC C211 871 2.412 4.373 3.576

7POPC H11R 872 2.442 4.301 3.655

7POPC H11S 873 2.502 4.387 3.512

7POPC C212 874 2.302 4.304 3.492

7POPC H12R 875 2.218 4.271 3.557

7POPC H12S 876 2.348 4.210 3.454

7POPC C213 877 2.247 4.381 3.372

7POPC H13R 878 2.194 4.474 3.403

7POPC H13S 879 2.175 4.316 3.319

7POPC C214 880 2.356 4.422 3.274

7POPC H14R 881 2.417 4.505 3.317

7POPC H14S 882 2.309 4.460 3.181

7POPC C215 883 2.448 4.304 3.244

7POPC H15R 884 2.389 4.213 3.221

7POPC H15S 885 2.508 4.282 3.335

7POPC C216 886 2.540 4.335 3.128

7POPC H16R 887 2.635 4.277 3.130

7POPC H16S 888 2.567 4.442 3.132

7POPC C217 889 2.463 4.304 3.000

7POPC H17R 890 2.505 4.380 2.931

7POPC H17S 891 2.355 4.327 3.012

7POPC C218 892 2.480 4.163 2.944

7POPC H18R 893 2.587 4.136 2.933

7POPC H18S 894 2.432 4.156 2.844

7POPC H18T 895 2.432 4.088 3.010

7POPC C33 896 2.726 4.963 4.375

7POPC H3X 897 2.633 5.018 4.351

7POPC H3Y 898 2.699 4.857 4.390

7POPC C34 899 2.822 4.978 4.257

7POPC H4X 900 2.910 4.913 4.273

7POPC H4Y 901 2.856 5.083 4.256

7POPC C35 902 2.757 4.949 4.122

7POPC H5X 903 2.836 4.955 4.044

7POPC H5Y 904 2.681 5.026 4.099

7POPC C36 905 2.693 4.810 4.110

7POPC H6X 906 2.595 4.811 4.161

7POPC H6Y 907 2.756 4.734 4.160

7POPC C37 908 2.678 4.774 3.962

7POPC H7X 909 2.668 4.868 3.904

7POPC H7Y 910 2.584 4.717 3.946

7POPC C38 911 2.797 4.692 3.912

7POPC H8X 912 2.788 4.587 3.950

7POPC H8Y 913 2.891 4.734 3.955

7POPC C39 914 2.807 4.691 3.760

7POPC H9X 915 2.810 4.796 3.724

7POPC H9Y 916 2.715 4.644 3.719

7POPC C310 917 2.932 4.617 3.711

7POPC H10X 918 2.929 4.512 3.746

7POPC H10Y 919 3.020 4.664 3.760

7POPC C311 920 2.953 4.622 3.559

7POPC H11X 921 3.062 4.609 3.541

7POPC H11Y 922 2.926 4.722 3.519

7POPC C312 923 2.882 4.508 3.483

7POPC H12X 924 2.891 4.415 3.542

7POPC H12Y 925 2.934 4.493 3.386

7POPC C313 926 2.734 4.531 3.451

7POPC H13X 927 2.677 4.541 3.546

7POPC H13Y 928 2.693 4.442 3.399

7POPC C314 929 2.710 4.651 3.360

7POPC H14X 930 2.668 4.618 3.263

7POPC H14Y 931 2.807 4.701 3.339

7POPC C315 932 2.616 4.751 3.425

7POPC H15X 933 2.642 4.764 3.532

7POPC H15Y 934 2.512 4.711 3.422

7POPC C316 935 2.625 4.886 3.355

7POPC H16X 936 2.616 4.873 3.245

7POPC H16Y 937 2.723 4.933 3.377

7POPC H16Z 938 2.543 4.952 3.390

8POPC N 939 1.822 3.236 5.046

8POPC C12 940 1.875 3.379 5.055

8POPC H12A 941 1.981 3.378 5.076

8POPC H12B 942 1.829 3.431 5.138

8POPC C13 943 1.884 3.152 5.153

8POPC H13A 944 1.858 3.190 5.250

8POPC H13B 945 1.850 3.050 5.147

8POPC H13C 946 1.992 3.155 5.145

8POPC C14 947 1.673 3.239 5.063

8POPC H14A 948 1.628 3.142 5.046

8POPC H14B 949 1.643 3.276 5.160

8POPC H14C 950 1.633 3.309 4.991

8POPC C15 951 1.852 3.186 4.909

8POPC H15A 952 1.957 3.192 4.886

8POPC H15B 953 1.803 3.252 4.839

8POPC H15C 954 1.813 3.086 4.892

8POPC C11 955 1.854 3.467 4.929

8POPC H11A 956 1.928 3.438 4.851

8POPC H11B 957 1.875 3.572 4.956

8POPC P 958 1.648 3.567 4.808

8POPC O13 959 1.741 3.623 4.707

8POPC O14 960 1.602 3.654 4.918

8POPC O12 961 1.723 3.448 4.877

8POPC O11 962 1.527 3.492 4.737

8POPC C1 963 1.504 3.351 4.746

8POPC HA 964 1.398 3.331 4.717

8POPC HB 965 1.512 3.317 4.851

8POPC C2 966 1.593 3.257 4.656

8POPC HS 967 1.606 3.163 4.713

8POPC O21 968 1.722 3.315 4.626

8POPC C21 969 1.812 3.234 4.574

8POPC O22 970 1.807 3.113 4.575

8POPC C22 971 1.934 3.315 4.531

8POPC H2R 972 1.990 3.336 4.625

8POPC H2S 973 1.900 3.412 4.489

8POPC C3 974 1.520 3.218 4.525

8POPC HX 975 1.422 3.172 4.552

8POPC HY 976 1.574 3.141 4.466

8POPC O31 977 1.494 3.337 4.450

8POPC C31 978 1.583 3.371 4.360

8POPC O32 979 1.679 3.304 4.325

8POPC C32 980 1.546 3.507 4.306

8POPC H2X 981 1.640 3.562 4.283

8POPC H2Y 982 1.489 3.560 4.385

8POPC C23 983 2.027 3.242 4.431

8POPC H3R 984 2.031 3.133 4.450

8POPC H3S 985 2.130 3.282 4.445

8POPC C24 986 1.987 3.269 4.286

8POPC H4R 987 1.973 3.378 4.272

8POPC H4S 988 1.889 3.220 4.264

8POPC C25 989 2.089 3.220 4.183

8POPC H5R 990 2.081 3.109 4.174

8POPC H5S 991 2.192 3.244 4.217

8POPC C26 992 2.069 3.283 4.044

8POPC H6R 993 2.138 3.233 3.973

8POPC H6S 994 2.096 3.390 4.047

8POPC C27 995 1.924 3.272 3.991

8POPC H7R 996 1.863 3.351 4.040

8POPC H7S 997 1.880 3.175 4.020

8POPC C28 998 1.915 3.290 3.838

8POPC H8R 999 1.977 3.377 3.808

8POPC H8S 1000 1.810 3.316 3.814

8POPC C29 1001 1.959 3.164 3.767

8POPC H91 1002 2.067 3.142 3.773

8POPC C210 1003 1.878 3.076 3.704

8POPC H101 1004 1.922 2.988 3.655

8POPC C211 1005 1.729 3.090 3.687

8POPC H11R 1006 1.685 3.160 3.762

8POPC H11S 1007 1.678 2.993 3.704

8POPC C212 1008 1.700 3.138 3.545

8POPC H12R 1009 1.714 3.051 3.477

8POPC H12S 1010 1.774 3.216 3.517

8POPC C213 1011 1.563 3.199 3.512

8POPC H13R 1012 1.541 3.284 3.581

8POPC H13S 1013 1.480 3.126 3.523

8POPC C214 1014 1.573 3.252 3.369

8POPC H14R 1015 1.603 3.168 3.303

8POPC H14S 1016 1.653 3.329 3.367

8POPC C215 1017 1.450 3.316 3.306

8POPC H15R 1018 1.417 3.404 3.366

8POPC H15S 1019 1.367 3.243 3.303

8POPC C216 1020 1.488 3.359 3.163

8POPC H16R 1021 1.398 3.361 3.098

8POPC H16S 1022 1.554 3.283 3.117

8POPC C217 1023 1.558 3.496 3.160

8POPC H17R 1024 1.667 3.477 3.151

8POPC H17S 1025 1.542 3.548 3.257

8POPC C218 1026 1.514 3.589 3.047

8POPC H18R 1027 1.469 3.530 2.965

8POPC H18S 1028 1.600 3.646 3.006

8POPC H18T 1029 1.439 3.662 3.084

8POPC C33 1030 1.459 3.490 4.182

8POPC H3X 1031 1.399 3.582 4.167

8POPC H3Y 1032 1.386 3.409 4.201

8POPC C34 1033 1.541 3.459 4.056

8POPC H4X 1034 1.611 3.374 4.075

8POPC H4Y 1035 1.603 3.548 4.033

8POPC C35 1036 1.452 3.423 3.937

8POPC H5X 1037 1.411 3.321 3.953

8POPC H5Y 1038 1.368 3.495 3.930

8POPC C36 1039 1.521 3.415 3.802

8POPC H6X 1040 1.442 3.416 3.724

8POPC H6Y 1041 1.573 3.317 3.796

8POPC C37 1042 1.623 3.524 3.773

8POPC H7X 1043 1.716 3.508 3.831

8POPC H7Y 1044 1.585 3.623 3.806

8POPC C38 1045 1.653 3.528 3.623

8POPC H8X 1046 1.558 3.527 3.565

8POPC H8Y 1047 1.711 3.437 3.597

8POPC C39 1048 1.731 3.653 3.588

8POPC H9X 1049 1.820 3.654 3.655

8POPC H9Y 1050 1.667 3.741 3.613

8POPC C310 1051 1.775 3.663 3.442

8POPC H10X 1052 1.814 3.765 3.421

8POPC H10Y 1053 1.688 3.646 3.376

8POPC C311 1054 1.886 3.561 3.413

8POPC H11X 1055 1.845 3.462 3.441

8POPC H11Y 1056 1.974 3.579 3.478

8POPC C312 1057 1.931 3.559 3.267

8POPC H12X 1058 2.000 3.643 3.249

8POPC H12Y 1059 1.843 3.573 3.200

8POPC C313 1060 1.999 3.428 3.230

8POPC H13X 1061 2.081 3.407 3.303

8POPC H13Y 1062 2.046 3.441 3.129

8POPC C314 1063 1.901 3.311 3.227

8POPC H14X 1064 1.817 3.330 3.157

8POPC H14Y 1065 1.857 3.301 3.328

8POPC C315 1066 1.963 3.175 3.195

8POPC H15X 1067 2.070 3.176 3.225

8POPC H15Y 1068 1.957 3.154 3.086

8POPC C316 1069 1.888 3.068 3.273

8POPC H16X 1070 1.780 3.071 3.250

8POPC H16Y 1071 1.903 3.084 3.382

8POPC H16Z 1072 1.925 2.966 3.249

9POPC N 1073 4.112 4.510 4.946

9POPC C12 1074 4.186 4.456 4.824

9POPC H12A 1075 4.288 4.435 4.853

9POPC H12B 1076 4.188 4.532 4.748

9POPC C13 1077 3.966 4.482 4.931

9POPC H13A 1078 3.925 4.540 4.849

9POPC H13B 1079 3.955 4.378 4.905

9POPC H13C 1080 3.913 4.501 5.023

9POPC C14 1081 4.162 4.436 5.067

9POPC H14A 1082 4.267 4.452 5.084

9POPC H14B 1083 4.106 4.461 5.156

9POPC H14C 1084 4.151 4.330 5.049

9POPC C15 1085 4.136 4.657 4.962

9POPC H15A 1086 4.241 4.679 4.969

9POPC H15B 1087 4.088 4.694 5.052

9POPC H15C 1088 4.098 4.713 4.879

9POPC C11 1089 4.129 4.327 4.760

9POPC H11A 1090 4.202 4.290 4.685

9POPC H11B 1091 4.037 4.351 4.703

9POPC P 1092 4.199 4.115 4.900

9POPC O13 1093 4.125 4.025 4.991

9POPC O14 1094 4.317 4.183 4.960

9POPC O12 1095 4.096 4.228 4.858

9POPC O11 1096 4.236 4.044 4.764

9POPC C1 1097 4.136 4.019 4.664

9POPC HA 1098 4.055 4.094 4.664

9POPC HB 1099 4.089 3.920 4.682

9POPC C2 1100 4.195 4.017 4.519

9POPC HS 1101 4.249 3.920 4.514

9POPC O21 1102 4.286 4.127 4.495

9POPC C21 1103 4.364 4.104 4.390

9POPC O22 1104 4.379 3.996 4.336

9POPC C22 1105 4.430 4.236 4.346

9POPC H2R 1106 4.504 4.261 4.425

9POPC H2S 1107 4.353 4.316 4.342

9POPC C3 1108 4.082 4.015 4.409

9POPC HX 1109 3.994 3.958 4.445

9POPC HY 1110 4.123 3.959 4.322

9POPC O31 1111 4.047 4.148 4.363

9POPC C31 1112 3.951 4.212 4.428

9POPC O32 1113 3.880 4.165 4.515

9POPC C32 1114 3.947 4.359 4.384

9POPC H2X 1115 3.904 4.416 4.469

9POPC H2Y 1116 3.878 4.367 4.297

9POPC C23 1117 4.504 4.227 4.210

9POPC H3R 1118 4.523 4.121 4.184

9POPC H3S 1119 4.604 4.274 4.223

9POPC C24 1120 4.439 4.297 4.088

9POPC H4R 1121 4.508 4.281 4.003

9POPC H4S 1122 4.436 4.407 4.106

9POPC C25 1123 4.299 4.251 4.041

9POPC H5R 1124 4.292 4.271 3.932

9POPC H5S 1125 4.221 4.315 4.089

9POPC C26 1126 4.262 4.104 4.067

9POPC H6R 1127 4.209 4.096 4.164

9POPC H6S 1128 4.354 4.043 4.079

9POPC C27 1129 4.178 4.037 3.957

9POPC H7R 1130 4.206 3.930 3.959

9POPC H7S 1131 4.205 4.076 3.856

9POPC C28 1132 4.027 4.050 3.981

9POPC H8R 1133 3.993 4.152 3.950

9POPC H8S 1134 4.008 4.044 4.091

9POPC C29 1135 3.951 3.939 3.913

9POPC H91 1136 3.955 3.843 3.967

9POPC C210 1137 3.884 3.945 3.797

9POPC H101 1138 3.835 3.854 3.760

9POPC C211 1139 3.869 4.065 3.707

9POPC H11R 1140 3.921 4.154 3.749

9POPC H11S 1141 3.762 4.091 3.698

9POPC C212 1142 3.925 4.038 3.566

9POPC H12R 1143 3.894 4.122 3.500

9POPC H12S 1144 3.881 3.945 3.525

9POPC C213 1145 4.078 4.027 3.560

9POPC H13R 1146 4.112 3.944 3.625

9POPC H13S 1147 4.124 4.121 3.599

9POPC C214 1148 4.126 4.002 3.416

9POPC H14R 1149 4.090 4.085 3.351

9POPC H14S 1150 4.076 3.909 3.382

9POPC C215 1151 4.277 3.987 3.400

9POPC H15R 1152 4.309 3.893 3.450

9POPC H15S 1153 4.328 4.071 3.451

9POPC C216 1154 4.323 3.984 3.253

9POPC H16R 1155 4.431 3.960 3.249

9POPC H16S 1156 4.309 4.085 3.207

9POPC C217 1157 4.252 3.882 3.166

9POPC H17R 1158 4.144 3.909 3.157

9POPC H17S 1159 4.259 3.786 3.221

9POPC C218 1160 4.310 3.862 3.026

9POPC H18R 1161 4.258 3.926 2.952

9POPC H18S 1162 4.295 3.757 2.995

9POPC H18T 1163 4.419 3.884 3.023

9POPC C33 1164 4.086 4.415 4.348

9POPC H3X 1165 4.119 4.370 4.252

9POPC H3Y 1166 4.159 4.383 4.426

9POPC C34 1167 4.095 4.568 4.339

9POPC H4X 1168 4.202 4.596 4.329

9POPC H4Y 1169 4.059 4.612 4.435

9POPC C35 1170 4.020 4.634 4.222

9POPC H5X 1171 4.038 4.744 4.226

9POPC H5Y 1172 3.910 4.619 4.234

9POPC C36 1173 4.063 4.584 4.084

9POPC H6X 1174 4.028 4.479 4.072

9POPC H6Y 1175 4.174 4.583 4.077

9POPC C37 1176 4.008 4.670 3.970

9POPC H7X 1177 4.045 4.774 3.981

9POPC H7Y 1178 3.897 4.676 3.977

9POPC C38 1179 4.046 4.618 3.830

9POPC H8X 1180 4.155 4.629 3.817

9POPC H8Y 1181 3.995 4.681 3.754

9POPC C39 1182 4.009 4.471 3.806

9POPC H9X 1183 3.910 4.450 3.850

9POPC H9Y 1184 4.082 4.404 3.857

9POPC C310 1185 4.004 4.436 3.656

9POPC H10X 1186 3.943 4.511 3.603

9POPC H10Y 1187 3.955 4.337 3.643

9POPC C311 1188 4.143 4.429 3.593

9POPC H11X 1189 4.202 4.351 3.646

9POPC H11Y 1190 4.194 4.526 3.605

9POPC C312 1191 4.138 4.395 3.444

9POPC H12X 1192 4.084 4.475 3.389

9POPC H12Y 1193 4.082 4.300 3.428

9POPC C313 1194 4.280 4.380 3.391

9POPC H13X 1195 4.330 4.296 3.444

9POPC H13Y 1196 4.336 4.473 3.414

9POPC C314 1197 4.290 4.355 3.240

9POPC H14X 1198 4.245 4.441 3.187

9POPC H14Y 1199 4.230 4.266 3.212

9POPC C315 1200 4.437 4.342 3.197

9POPC H15X 1201 4.457 4.240 3.158

9POPC H15Y 1202 4.500 4.353 3.288

9POPC C316 1203 4.481 4.447 3.095

9POPC H16X 1204 4.589 4.473 3.110

9POPC H16Y 1205 4.469 4.410 2.991

9POPC H16Z 1206 4.422 4.540 3.106

10POPC N 1207 4.323 2.600 4.884

10POPC C12 1208 4.405 2.663 4.771

10POPC H12A 1209 4.385 2.769 4.768

10POPC H12B 1210 4.373 2.624 4.675

10POPC C13 1211 4.312 2.702 4.994

10POPC H13A 1212 4.255 2.666 5.079

10POPC H13B 1213 4.411 2.730 5.028

10POPC H13C 1214 4.267 2.793 4.958

10POPC C14 1215 4.187 2.562 4.836

10POPC H14A 1216 4.190 2.486 4.759

10POPC H14B 1217 4.123 2.526 4.915

10POPC H14C 1218 4.136 2.647 4.793

10POPC C15 1219 4.398 2.484 4.940

10POPC H15A 1220 4.496 2.518 4.967

10POPC H15B 1221 4.409 2.406 4.866

10POPC H15C 1222 4.350 2.446 5.029

10POPC C11 1223 4.560 2.649 4.779

10POPC H11A 1224 4.606 2.704 4.694

10POPC H11B 1225 4.589 2.542 4.763

10POPC P 1226 4.637 2.847 4.927

10POPC O13 1227 4.767 2.884 4.868

10POPC O14 1228 4.616 2.876 5.071

10POPC O12 1229 4.611 2.692 4.905

10POPC O11 1230 4.518 2.909 4.843

10POPC C1 1231 4.430 3.007 4.896

10POPC HA 1232 4.486 3.099 4.922

10POPC HB 1233 4.378 2.972 4.988

10POPC C2 1234 4.321 3.048 4.792

10POPC HS 1235 4.267 3.134 4.837

10POPC O21 1236 4.230 2.940 4.759

10POPC C21 1237 4.117 2.937 4.825

10POPC O22 1238 4.099 2.976 4.940

10POPC C22 1239 4.011 2.862 4.743

10POPC H2R 1240 3.933 2.936 4.715

10POPC H2S 1241 3.965 2.787 4.811

10POPC C3 1242 4.384 3.098 4.659

10POPC HX 1243 4.461 3.174 4.685

10POPC HY 1244 4.305 3.147 4.599

10POPC O31 1245 4.439 2.987 4.585

10POPC C31 1246 4.536 3.016 4.501

10POPC O32 1247 4.583 3.127 4.481

10POPC C32 1248 4.581 2.886 4.436

10POPC H2X 1249 4.499 2.812 4.441

10POPC H2Y 1250 4.667 2.848 4.496

10POPC C23 1251 4.062 2.792 4.614

10POPC H3R 1252 4.163 2.748 4.631

10POPC H3S 1253 4.074 2.868 4.534

10POPC C24 1254 3.969 2.679 4.567

10POPC H4R 1255 3.872 2.722 4.535

10POPC H4S 1256 3.948 2.610 4.652

10POPC C25 1257 4.025 2.596 4.450

10POPC H5R 1258 3.952 2.516 4.425

10POPC H5S 1259 4.119 2.546 4.481

10POPC C26 1260 4.050 2.679 4.324

10POPC H6R 1261 4.011 2.782 4.339

10POPC H6S 1262 3.994 2.636 4.238

10POPC C27 1263 4.199 2.688 4.287

10POPC H7R 1264 4.232 2.592 4.241

10POPC H7S 1265 4.259 2.703 4.380

10POPC C28 1266 4.231 2.805 4.193

10POPC H8R 1267 4.340 2.808 4.176

10POPC H8S 1268 4.204 2.899 4.246

10POPC C29 1269 4.158 2.797 4.062

10POPC H91 1270 4.082 2.718 4.051

10POPC C210 1271 4.180 2.881 3.960

10POPC H101 1272 4.123 2.870 3.866

10POPC C211 1273 4.280 2.994 3.962

10POPC H11R 1274 4.326 3.009 4.062

10POPC H11S 1275 4.226 3.088 3.939

10POPC C212 1276 4.394 2.973 3.861

10POPC H12R 1277 4.446 2.877 3.881

10POPC H12S 1278 4.469 3.054 3.875

10POPC C213 1279 4.347 2.976 3.715

10POPC H13R 1280 4.297 3.074 3.698

10POPC H13S 1281 4.273 2.897 3.695

10POPC C214 1282 4.463 2.963 3.616

10POPC H14R 1283 4.490 2.855 3.605

10POPC H14S 1284 4.553 3.015 3.656

10POPC C215 1285 4.429 3.023 3.480

10POPC H15R 1286 4.414 3.133 3.492

10POPC H15S 1287 4.333 2.981 3.443

10POPC C216 1288 4.539 2.997 3.377

10POPC H16R 1289 4.551 2.887 3.366

10POPC H16S 1290 4.636 3.037 3.413

10POPC C217 1291 4.505 3.058 3.241

10POPC H17R 1292 4.399 3.037 3.219

10POPC H17S 1293 4.566 3.010 3.162

10POPC C218 1294 4.530 3.208 3.237

10POPC H18R 1295 4.637 3.229 3.218

10POPC H18S 1296 4.499 3.256 3.333

10POPC H18T 1297 4.470 3.257 3.157

10POPC C33 1298 4.624 2.903 4.289

10POPC H3X 1299 4.696 2.987 4.279

10POPC H3Y 1300 4.536 2.927 4.226

10POPC C34 1301 4.691 2.775 4.237

10POPC H4X 1302 4.628 2.687 4.259

10POPC H4Y 1303 4.788 2.762 4.290

10POPC C35 1304 4.717 2.781 4.087

10POPC H5X 1305 4.758 2.881 4.060

10POPC H5Y 1306 4.620 2.770 4.034

10POPC C36 1307 4.816 2.675 4.041

10POPC H6X 1308 4.785 2.574 4.076

10POPC H6Y 1309 4.915 2.697 4.086

10POPC C37 1310 4.830 2.674 3.889

10POPC H7X 1311 4.923 2.619 3.860

10POPC H7Y 1312 4.840 2.779 3.854

10POPC C38 1313 4.711 2.611 3.818

10POPC H8X 1314 4.619 2.670 3.838

10POPC H8Y 1315 4.694 2.508 3.857

10POPC C39 1316 4.732 2.602 3.667

10POPC H9X 1317 4.826 2.547 3.647

10POPC H9Y 1318 4.743 2.705 3.625

10POPC C310 1319 4.615 2.532 3.598

10POPC H10X 1320 4.522 2.578 3.637

10POPC H10Y 1321 4.614 2.424 3.627

10POPC C311 1322 4.618 2.544 3.446

10POPC H11X 1323 4.709 2.495 3.406

10POPC H11Y 1324 4.622 2.651 3.418

10POPC C312 1325 4.497 2.478 3.378

10POPC H12X 1326 4.498 2.369 3.396

10POPC H12Y 1327 4.506 2.492 3.268

10POPC C313 1328 4.363 2.536 3.427

10POPC H13X 1329 4.381 2.628 3.487

10POPC H13Y 1330 4.315 2.462 3.494

10POPC C314 1331 4.266 2.575 3.315

10POPC H14X 1332 4.163 2.570 3.355

10POPC H14Y 1333 4.273 2.502 3.231

10POPC C315 1334 4.290 2.717 3.261

10POPC H15X 1335 4.295 2.788 3.346

10POPC H15Y 1336 4.201 2.744 3.201

10POPC C316 1337 4.416 2.730 3.174

10POPC H16X 1338 4.417 2.829 3.122

10POPC H16Y 1339 4.419 2.651 3.097

10POPC H16Z 1340 4.508 2.722 3.235

11POPC N 1341 1.974 4.176 5.124

11POPC C12 1342 2.050 4.184 4.993

11POPC H12A 1343 2.139 4.123 4.997

11POPC H12B 1344 2.079 4.287 4.976

11POPC C13 1345 1.881 4.294 5.132

11POPC H13A 1346 1.814 4.289 5.047

11POPC H13B 1347 1.823 4.297 5.223

11POPC H13C 1348 1.934 4.387 5.118

11POPC C14 1349 1.891 4.051 5.123

11POPC H14A 1350 1.830 4.042 5.212

11POPC H14B 1351 1.826 4.055 5.037

11POPC H14C 1352 1.953 3.963 5.111

11POPC C15 1353 2.067 4.178 5.241

11POPC H15A 1354 2.137 4.096 5.237

11POPC H15B 1355 2.013 4.171 5.334

11POPC H15C 1356 2.124 4.270 5.243

11POPC C11 1357 1.972 4.143 4.866

11POPC H11A 1358 1.943 4.036 4.867

11POPC H11B 1359 2.042 4.152 4.780

11POPC P 1360 1.872 4.370 4.791

11POPC O13 1361 1.967 4.442 4.879

11POPC O14 1362 1.737 4.431 4.778

11POPC O12 1363 1.855 4.224 4.850

11POPC O11 1364 1.937 4.341 4.651

11POPC C1 1365 1.900 4.226 4.574

11POPC HA 1366 1.803 4.182 4.605

11POPC HB 1367 1.978 4.147 4.582

11POPC C2 1368 1.889 4.261 4.425

11POPC HS 1369 1.990 4.290 4.390

11POPC O21 1370 1.792 4.366 4.411

11POPC C21 1371 1.844 4.488 4.406

11POPC O22 1372 1.959 4.517 4.381

11POPC C22 1373 1.732 4.588 4.436

11POPC H2R 1374 1.774 4.690 4.421

11POPC H2S 1375 1.705 4.575 4.543

11POPC C3 1376 1.841 4.139 4.343

11POPC HX 1377 1.737 4.117 4.375

11POPC HY 1378 1.903 4.050 4.365

11POPC O31 1379 1.838 4.172 4.203

11POPC C31 1380 1.960 4.185 4.154

11POPC O32 1381 2.062 4.138 4.200

11POPC C32 1382 1.953 4.273 4.031

11POPC H2X 1383 1.992 4.374 4.056

11POPC H2Y 1384 1.847 4.283 4.000

11POPC C23 1385 1.609 4.565 4.346

11POPC H3R 1386 1.529 4.635 4.380

11POPC H3S 1387 1.570 4.461 4.360

11POPC C24 1388 1.636 4.590 4.197

11POPC H4R 1389 1.721 4.527 4.161

11POPC H4S 1390 1.666 4.696 4.185

11POPC C25 1391 1.513 4.565 4.110

11POPC H5R 1392 1.516 4.632 4.022

11POPC H5S 1393 1.423 4.591 4.171

11POPC C26 1394 1.497 4.420 4.062

11POPC H6R 1395 1.389 4.398 4.048

11POPC H6S 1396 1.535 4.350 4.140

11POPC C27 1397 1.567 4.397 3.928

11POPC H7R 1398 1.676 4.417 3.938

11POPC H7S 1399 1.527 4.471 3.855

11POPC C28 1400 1.542 4.257 3.871

11POPC H8R 1401 1.574 4.258 3.765

11POPC H8S 1402 1.433 4.236 3.873

11POPC C29 1403 1.617 4.151 3.949

11POPC H91 1404 1.635 4.173 4.055

11POPC C210 1405 1.668 4.039 3.896

11POPC H101 1406 1.721 3.968 3.962

11POPC C211 1407 1.648 3.993 3.754

11POPC H11R 1408 1.575 4.058 3.701

11POPC H11S 1409 1.604 3.891 3.756

11POPC C212 1410 1.772 3.988 3.665

11POPC H12R 1411 1.804 4.091 3.639

11POPC H12S 1412 1.743 3.939 3.569

11POPC C213 1413 1.889 3.910 3.727

11POPC H13R 1414 1.850 3.820 3.779

11POPC H13S 1415 1.934 3.973 3.807

11POPC C214 1416 1.998 3.868 3.628

11POPC H14R 1417 1.972 3.769 3.585

11POPC H14S 1418 2.094 3.852 3.682

11POPC C215 1419 2.022 3.962 3.509

11POPC H15R 1420 2.064 4.058 3.544

11POPC H15S 1421 1.928 3.983 3.454

11POPC C216 1422 2.120 3.895 3.413

11POPC H16R 1423 2.070 3.810 3.363

11POPC H16S 1424 2.204 3.852 3.471

11POPC C217 1425 2.176 3.992 3.310

11POPC H17R 1426 2.220 4.079 3.362

11POPC H17S 1427 2.095 4.032 3.246

11POPC C218 1428 2.283 3.925 3.224

11POPC H18R 1429 2.359 3.876 3.288

11POPC H18S 1430 2.332 3.999 3.158

11POPC H18T 1431 2.234 3.848 3.161

11POPC C33 1432 2.034 4.207 3.920

11POPC H3X 1433 2.035 4.097 3.933

11POPC H3Y 1434 2.140 4.241 3.926

11POPC C34 1435 1.976 4.238 3.783

11POPC H4X 1436 1.865 4.243 3.788

11POPC H4Y 1437 2.003 4.156 3.714

11POPC C35 1438 2.034 4.365 3.728

11POPC H5X 1439 2.145 4.356 3.730

11POPC H5Y 1440 2.007 4.450 3.795

11POPC C36 1441 1.987 4.392 3.586

11POPC H6X 1442 1.998 4.302 3.522

11POPC H6Y 1443 2.055 4.470 3.545

11POPC C37 1444 1.845 4.445 3.582

11POPC H7X 1445 1.835 4.520 3.664

11POPC H7Y 1446 1.772 4.364 3.602

11POPC C38 1447 1.815 4.515 3.450

11POPC H8X 1448 1.786 4.439 3.374

11POPC H8Y 1449 1.906 4.566 3.413

11POPC C39 1450 1.704 4.619 3.467

11POPC H9X 1451 1.744 4.706 3.523

11POPC H9Y 1452 1.623 4.575 3.529

11POPC C310 1453 1.643 4.665 3.334

11POPC H10X 1454 1.570 4.746 3.353

11POPC H10Y 1455 1.587 4.578 3.293

11POPC C311 1456 1.744 4.710 3.227

11POPC H11X 1457 1.827 4.637 3.220

11POPC H11Y 1458 1.787 4.809 3.254

11POPC C312 1459 1.676 4.719 3.090

11POPC H12X 1460 1.597 4.797 3.096

11POPC H12Y 1461 1.625 4.623 3.070

11POPC C313 1462 1.772 4.751 2.974

11POPC H13X 1463 1.816 4.852 2.988

11POPC H13Y 1464 1.711 4.754 2.881

11POPC C314 1465 1.883 4.648 2.951

11POPC H14X 1466 1.961 4.657 3.030

11POPC H14Y 1467 1.932 4.672 2.854

11POPC C315 1468 1.831 4.504 2.946

11POPC H15X 1469 1.731 4.504 2.898

11POPC H15Y 1470 1.817 4.466 3.049

11POPC C316 1471 1.922 4.411 2.868

11POPC H16X 1472 1.875 4.311 2.858

11POPC H16Y 1473 2.020 4.400 2.919

11POPC H16Z 1474 1.940 4.450 2.765

12POPC N 1475 3.666 4.851 5.080

12POPC C12 1476 3.668 4.952 4.965

12POPC H12A 1477 3.770 4.980 4.943

12POPC H12B 1478 3.616 5.041 4.996

12POPC C13 1479 3.744 4.901 5.198

12POPC H13A 1480 3.702 4.993 5.235

12POPC H13B 1481 3.743 4.829 5.278

12POPC H13C 1482 3.847 4.919 5.171

12POPC C14 1483 3.523 4.830 5.122

12POPC H14A 1484 3.479 4.922 5.158

12POPC H14B 1485 3.465 4.804 5.036

12POPC H14C 1486 3.512 4.754 5.198

12POPC C15 1487 3.722 4.721 5.030

12POPC H15A 1488 3.822 4.735 4.991

12POPC H15B 1489 3.661 4.687 4.948

12POPC H15C 1490 3.724 4.646 5.108

12POPC C11 1491 3.602 4.909 4.832

12POPC H11A 1492 3.656 4.823 4.786

12POPC H11B 1493 3.610 4.994 4.761

12POPC P 1494 3.349 4.899 4.756

12POPC O13 1495 3.371 5.031 4.692

12POPC O14 1496 3.224 4.877 4.833

12POPC O12 1497 3.467 4.872 4.857

12POPC O11 1498 3.373 4.783 4.653

12POPC C1 1499 3.385 4.649 4.700

12POPC HA 1500 3.297 4.621 4.762

12POPC HB 1501 3.477 4.637 4.760

12POPC C2 1502 3.392 4.548 4.582

12POPC HS 1503 3.402 4.447 4.628

12POPC O21 1504 3.509 4.577 4.503

12POPC C21 1505 3.536 4.485 4.412

12POPC O22 1506 3.499 4.369 4.417

12POPC C22 1507 3.617 4.548 4.299

12POPC H2R 1508 3.725 4.541 4.326

12POPC H2S 1509 3.589 4.656 4.292

12POPC C3 1510 3.262 4.548 4.496

12POPC HX 1511 3.176 4.541 4.566

12POPC HY 1512 3.260 4.455 4.435

12POPC O31 1513 3.248 4.669 4.419

12POPC C31 1514 3.251 4.658 4.287

12POPC O32 1515 3.253 4.553 4.225

12POPC C32 1516 3.256 4.796 4.222

12POPC H2X 1517 3.334 4.856 4.274

12POPC H2Y 1518 3.157 4.844 4.239

12POPC C23 1519 3.588 4.479 4.165

12POPC H3R 1520 3.478 4.476 4.148

12POPC H3S 1521 3.623 4.373 4.168

12POPC C24 1522 3.651 4.550 4.045

12POPC H4R 1523 3.761 4.546 4.057

12POPC H4S 1524 3.619 4.657 4.044

12POPC C25 1525 3.610 4.483 3.914

12POPC H5R 1526 3.499 4.475 3.909

12POPC H5S 1527 3.651 4.380 3.915

12POPC C26 1528 3.660 4.555 3.788

12POPC H6R 1529 3.760 4.600 3.808

12POPC H6S 1530 3.592 4.640 3.763

12POPC C27 1531 3.672 4.460 3.667

12POPC H7R 1532 3.747 4.383 3.694

12POPC H7S 1533 3.710 4.516 3.579

12POPC C28 1534 3.543 4.384 3.630

12POPC H8R 1535 3.503 4.333 3.720

12POPC H8S 1536 3.573 4.303 3.560

12POPC C29 1537 3.440 4.476 3.571

12POPC H91 1538 3.410 4.558 3.637

12POPC C210 1539 3.382 4.466 3.450

12POPC H101 1540 3.307 4.541 3.421

12POPC C211 1541 3.405 4.359 3.345

12POPC H11R 1542 3.311 4.302 3.332

12POPC H11S 1543 3.483 4.286 3.376

12POPC C212 1544 3.440 4.409 3.204

12POPC H12R 1545 3.376 4.496 3.175

12POPC H12S 1546 3.417 4.327 3.132

12POPC C213 1547 3.588 4.443 3.188

12POPC H13R 1548 3.613 4.448 3.080

12POPC H13S 1549 3.649 4.363 3.235

12POPC C214 1550 3.621 4.578 3.252

12POPC H14R 1551 3.582 4.585 3.355

12POPC H14S 1552 3.569 4.655 3.191

12POPC C215 1553 3.770 4.604 3.262

12POPC H15R 1554 3.814 4.598 3.160

12POPC H15S 1555 3.817 4.525 3.324

12POPC C216 1556 3.800 4.741 3.325

12POPC H16R 1557 3.910 4.752 3.332

12POPC H16S 1558 3.759 4.742 3.429

12POPC C217 1559 3.739 4.859 3.248

12POPC H17R 1560 3.631 4.842 3.231

12POPC H17S 1561 3.784 4.863 3.146

12POPC C218 1562 3.753 4.995 3.317

12POPC H18R 1563 3.654 5.043 3.330

12POPC H18S 1564 3.815 5.063 3.255

12POPC H18T 1565 3.802 4.985 3.416

12POPC C33 1566 3.286 4.787 4.071

12POPC H3X 1567 3.209 4.723 4.022

12POPC H3Y 1568 3.385 4.738 4.056

12POPC C34 1569 3.285 4.925 4.003

12POPC H4X 1570 3.371 4.984 4.040

12POPC H4Y 1571 3.193 4.978 4.034

12POPC C35 1572 3.289 4.918 3.850

12POPC H5X 1573 3.283 5.022 3.810

12POPC H5Y 1574 3.199 4.863 3.814

12POPC C36 1575 3.414 4.850 3.793

12POPC H6X 1576 3.424 4.750 3.840

12POPC H6Y 1577 3.505 4.909 3.820

12POPC C37 1578 3.410 4.830 3.641

12POPC H7X 1579 3.319 4.769 3.619

12POPC H7Y 1580 3.499 4.772 3.609

12POPC C38 1581 3.406 4.960 3.559

12POPC H8X 1582 3.497 5.019 3.581

12POPC H8Y 1583 3.318 5.021 3.588

12POPC C39 1584 3.399 4.932 3.409

12POPC H9X 1585 3.490 4.875 3.378

12POPC H9Y 1586 3.398 5.028 3.354

12POPC C310 1587 3.275 4.851 3.369

12POPC H10X 1588 3.186 4.895 3.420

12POPC H10Y 1589 3.286 4.746 3.403

12POPC C311 1590 3.249 4.852 3.218

12POPC H11X 1591 3.337 4.807 3.166

12POPC H11Y 1592 3.241 4.957 3.184

12POPC C312 1593 3.122 4.775 3.180

12POPC H12X 1594 3.042 4.801 3.253

12POPC H12Y 1595 3.141 4.666 3.190

12POPC C313 1596 3.070 4.805 3.038

12POPC H13X 1597 3.053 4.914 3.024

12POPC H13Y 1598 2.972 4.754 3.026

12POPC C314 1599 3.163 4.755 2.927

12POPC H14X 1600 3.104 4.731 2.836

12POPC H14Y 1601 3.210 4.660 2.961

12POPC C315 1602 3.271 4.856 2.888

12POPC H15X 1603 3.307 4.912 2.977

12POPC H15Y 1604 3.228 4.932 2.819

12POPC C316 1605 3.391 4.789 2.823

12POPC H16X 1606 3.366 4.764 2.718

12POPC H16Y 1607 3.418 4.696 2.878

12POPC H16Z 1608 3.480 4.855 2.824

13POPC N 1609 4.617 3.532 5.063

13POPC C12 1610 4.747 3.487 4.996

13POPC H12A 1611 4.748 3.379 4.991

13POPC H12B 1612 4.830 3.516 5.060

13POPC C13 1613 4.595 3.452 5.187

13POPC H13A 1614 4.498 3.470 5.231

13POPC H13B 1615 4.601 3.346 5.166

13POPC H13C 1616 4.670 3.474 5.261

13POPC C14 1617 4.629 3.677 5.098

13POPC H14A 1618 4.707 3.694 5.171

13POPC H14B 1619 4.658 3.731 5.010

13POPC H14C 1620 4.535 3.718 5.135

13POPC C15 1621 4.503 3.514 4.967

13POPC H15A 1622 4.493 3.411 4.937

13POPC H15B 1623 4.521 3.573 4.879

13POPC H15C 1624 4.409 3.546 5.010

13POPC C11 1625 4.778 3.544 4.856

13POPC H11A 1626 4.707 3.502 4.782

13POPC H11B 1627 4.879 3.508 4.826

13POPC P 1628 4.870 3.778 4.778

13POPC O13 1629 5.007 3.757 4.829

13POPC O14 1630 4.819 3.917 4.781

13POPC O12 1631 4.769 3.686 4.856

13POPC O11 1632 4.859 3.720 4.632

13POPC C1 1633 4.733 3.722 4.565

13POPC HA 1634 4.683 3.821 4.576

13POPC HB 1635 4.665 3.644 4.604

13POPC C2 1636 4.751 3.701 4.413

13POPC HS 1637 4.656 3.733 4.366

13POPC O21 1638 4.780 3.562 4.388

13POPC C21 1639 4.728 3.512 4.276

13POPC O22 1640 4.642 3.565 4.208

13POPC C22 1641 4.792 3.376 4.248

13POPC H2R 1642 4.725 3.298 4.290

13POPC H2S 1643 4.890 3.371 4.300

13POPC C3 1644 4.867 3.789 4.358

13POPC HX 1645 4.959 3.727 4.355

13POPC HY 1646 4.889 3.871 4.431

13POPC O31 1647 4.837 3.840 4.226

13POPC C31 1648 4.763 3.950 4.227

13POPC O32 1649 4.723 4.009 4.326

13POPC C32 1650 4.741 3.999 4.084

13POPC H2X 1651 4.838 3.986 4.031

13POPC H2Y 1652 4.719 4.107 4.090

13POPC C23 1653 4.813 3.351 4.096

13POPC H3R 1654 4.715 3.350 4.045

13POPC H3S 1655 4.860 3.251 4.082

13POPC C24 1656 4.903 3.458 4.031

13POPC H4R 1657 5.001 3.464 4.082

13POPC H4S 1658 4.852 3.556 4.043

13POPC C25 1659 4.925 3.431 3.881

13POPC H5R 1660 4.825 3.423 3.834

13POPC H5S 1661 4.977 3.334 3.867

13POPC C26 1662 5.003 3.542 3.809

13POPC H6R 1663 5.010 3.517 3.701

13POPC H6S 1664 5.107 3.548 3.849

13POPC C27 1665 4.935 3.679 3.825

13POPC H7R 1666 4.944 3.710 3.931

13POPC H7S 1667 4.827 3.667 3.802

13POPC C28 1668 4.996 3.787 3.736

13POPC H8R 1669 4.990 3.760 3.628

13POPC H8S 1670 5.104 3.795 3.761

13POPC C29 1671 4.927 3.917 3.769

13POPC H91 1672 4.939 3.942 3.875

13POPC C210 1673 4.862 4.005 3.691

13POPC H101 1674 4.822 4.096 3.739

13POPC C211 1675 4.840 4.001 3.542

13POPC H11R 1676 4.937 4.014 3.490

13POPC H11S 1677 4.777 4.088 3.514

13POPC C212 1678 4.774 3.875 3.485

13POPC H12R 1679 4.665 3.875 3.507

13POPC H12S 1680 4.817 3.782 3.529

13POPC C213 1681 4.793 3.870 3.335

13POPC H13R 1682 4.754 3.774 3.295

13POPC H13S 1683 4.901 3.876 3.310

13POPC C214 1684 4.716 3.984 3.269

13POPC H14R 1685 4.759 4.082 3.296

13POPC H14S 1686 4.612 3.982 3.309

13POPC C215 1687 4.717 3.970 3.118

13POPC H15R 1688 4.648 4.046 3.076

13POPC H15S 1689 4.684 3.870 3.083

13POPC C216 1690 4.858 3.992 3.066

13POPC H16R 1691 4.916 3.897 3.064

13POPC H16S 1692 4.912 4.060 3.135

13POPC C217 1693 4.851 4.059 2.931

13POPC H17R 1694 4.955 4.093 2.911

13POPC H17S 1695 4.784 4.147 2.941

13POPC C218 1696 4.804 3.973 2.815

13POPC H18R 1697 4.715 3.912 2.840

13POPC H18S 1698 4.889 3.906 2.789

13POPC H18T 1699 4.785 4.034 2.724

13POPC C33 1700 4.628 3.924 4.010

13POPC H3X 1701 4.544 3.900 4.079

13POPC H3Y 1702 4.670 3.827 3.974

13POPC C34 1703 4.569 4.005 3.893

13POPC H4X 1704 4.644 4.081 3.863

13POPC H4Y 1705 4.481 4.063 3.928

13POPC C35 1706 4.529 3.927 3.767

13POPC H5X 1707 4.622 3.922 3.706

13POPC H5Y 1708 4.456 3.985 3.706

13POPC C36 1709 4.480 3.782 3.782

13POPC H6X 1710 4.370 3.776 3.766

13POPC H6Y 1711 4.499 3.743 3.885

13POPC C37 1712 4.555 3.692 3.682

13POPC H7X 1713 4.661 3.724 3.679

13POPC H7Y 1714 4.513 3.705 3.580

13POPC C38 1715 4.560 3.544 3.719

13POPC H8X 1716 4.457 3.502 3.711

13POPC H8Y 1717 4.595 3.532 3.823

13POPC C39 1718 4.655 3.469 3.624

13POPC H9X 1719 4.757 3.512 3.638

13POPC H9Y 1720 4.624 3.491 3.519

13POPC C310 1721 4.661 3.318 3.647

13POPC H10X 1722 4.568 3.272 3.605

13POPC H10Y 1723 4.663 3.296 3.755

13POPC C311 1724 4.783 3.252 3.580

13POPC H11X 1725 4.772 3.142 3.589

13POPC H11Y 1726 4.876 3.281 3.633

13POPC C312 1727 4.795 3.288 3.432

13POPC H12X 1728 4.694 3.321 3.400

13POPC H12Y 1729 4.822 3.199 3.372

13POPC C313 1730 4.894 3.402 3.399

13POPC H13X 1731 4.988 3.356 3.362

13POPC H13Y 1732 4.919 3.460 3.491

13POPC C314 1733 4.833 3.498 3.296

13POPC H14X 1734 4.771 3.573 3.352

13POPC H14Y 1735 4.764 3.442 3.230

13POPC C315 1736 4.934 3.570 3.206

13POPC H15X 1737 5.011 3.619 3.269

13POPC H15Y 1738 4.882 3.650 3.149

13POPC C316 1739 4.997 3.473 3.107

13POPC H16X 1740 4.922 3.440 3.032

13POPC H16Y 1741 5.036 3.383 3.160

13POPC H16Z 1742 5.084 3.517 3.055

14POPC N 1743 2.320 1.973 4.880

14POPC C12 1744 2.197 1.929 4.800

14POPC H12A 1745 2.164 1.833 4.838

14POPC H12B 1746 2.223 1.917 4.696

14POPC C13 1747 2.436 1.881 4.855

14POPC H13A 1748 2.522 1.914 4.912

14POPC H13B 1749 2.411 1.781 4.885

14POPC H13C 1750 2.464 1.881 4.751

14POPC C14 1751 2.356 2.113 4.843

14POPC H14A 1752 2.441 2.149 4.901

14POPC H14B 1753 2.380 2.123 4.738

14POPC H14C 1754 2.273 2.179 4.864

14POPC C15 1755 2.284 1.969 5.026

14POPC H15A 1756 2.192 2.024 5.039

14POPC H15B 1757 2.359 2.016 5.089

14POPC H15C 1758 2.263 1.869 5.060

14POPC C11 1759 2.072 2.021 4.809

14POPC H11A 1760 1.997 1.988 4.734

14POPC H11B 1761 2.098 2.125 4.780

14POPC P 1762 1.938 1.894 4.989

14POPC O13 1763 1.879 1.929 5.120

14POPC O14 1764 2.030 1.777 4.981

14POPC O12 1765 2.017 2.022 4.941

14POPC O11 1766 1.828 1.885 4.880

14POPC C1 1767 1.729 1.986 4.864

14POPC HA 1768 1.776 2.079 4.827

14POPC HB 1769 1.678 2.007 4.960

14POPC C2 1770 1.614 1.949 4.764

14POPC HS 1771 1.522 1.927 4.822

14POPC O21 1772 1.645 1.838 4.683

14POPC C21 1773 1.668 1.724 4.737

14POPC O22 1774 1.635 1.683 4.847

14POPC C22 1775 1.751 1.651 4.632

14POPC H2R 1776 1.720 1.693 4.534

14POPC H2S 1777 1.723 1.544 4.634

14POPC C3 1778 1.583 2.061 4.663

14POPC HX 1779 1.578 2.157 4.720

14POPC HY 1780 1.484 2.037 4.618

14POPC O31 1781 1.683 2.076 4.559

14POPC C31 1782 1.650 2.174 4.477

14POPC O32 1783 1.570 2.263 4.502

14POPC C32 1784 1.719 2.153 4.343

14POPC H2X 1785 1.647 2.180 4.264

14POPC H2Y 1786 1.743 2.045 4.333

14POPC C23 1787 1.903 1.682 4.636

14POPC H3R 1788 1.953 1.614 4.708

14POPC H3S 1789 1.919 1.786 4.671

14POPC C24 1790 1.966 1.672 4.496

14POPC H4R 1791 1.928 1.580 4.446

14POPC H4S 1792 2.076 1.662 4.505

14POPC C25 1793 1.936 1.794 4.406

14POPC H5R 1794 1.985 1.885 4.447

14POPC H5S 1795 1.826 1.814 4.407

14POPC C26 1796 1.976 1.774 4.259

14POPC H6R 1797 1.924 1.851 4.198

14POPC H6S 1798 1.941 1.674 4.226

14POPC C27 1799 2.127 1.789 4.233

14POPC H7R 1800 2.183 1.716 4.295

14POPC H7S 1801 2.157 1.890 4.266

14POPC C28 1802 2.164 1.773 4.083

14POPC H8R 1803 2.267 1.815 4.071

14POPC H8S 1804 2.096 1.838 4.024

14POPC C29 1805 2.163 1.631 4.034

14POPC H91 1806 2.177 1.555 4.112

14POPC C210 1807 2.146 1.590 3.906

14POPC H101 1808 2.150 1.482 3.885

14POPC C211 1809 2.119 1.677 3.785

14POPC H11R 1810 2.213 1.696 3.729

14POPC H11S 1811 2.080 1.775 3.819

14POPC C212 1812 2.016 1.616 3.688

14POPC H12R 1813 1.932 1.570 3.745

14POPC H12S 1814 2.065 1.533 3.632

14POPC C213 1815 1.952 1.712 3.584

14POPC H13R 1816 1.869 1.658 3.533

14POPC H13S 1817 2.031 1.728 3.507

14POPC C214 1818 1.898 1.848 3.633

14POPC H14R 1819 1.978 1.902 3.690

14POPC H14S 1820 1.812 1.834 3.702

14POPC C215 1821 1.846 1.943 3.524

14POPC H15R 1822 1.748 1.905 3.490

14POPC H15S 1823 1.915 1.942 3.436

14POPC C216 1824 1.830 2.087 3.574

14POPC H16R 1825 1.931 2.134 3.578

14POPC H16S 1826 1.790 2.086 3.678

14POPC C217 1827 1.741 2.179 3.488

14POPC H17R 1828 1.756 2.284 3.521

14POPC H17S 1829 1.634 2.156 3.509

14POPC C218 1830 1.766 2.169 3.337

14POPC H18R 1831 1.871 2.196 3.315

14POPC H18S 1832 1.699 2.239 3.282

14POPC H18T 1833 1.746 2.066 3.300

14POPC C33 1834 1.848 2.236 4.326

14POPC H3X 1835 1.918 2.214 4.409

14POPC H3Y 1836 1.825 2.345 4.327

14POPC C34 1837 1.914 2.198 4.193

14POPC H4X 1838 1.846 2.226 4.110

14POPC H4Y 1839 1.928 2.088 4.190

14POPC C35 1840 2.049 2.265 4.174

14POPC H5X 1841 2.109 2.260 4.267

14POPC H5Y 1842 2.032 2.373 4.153

14POPC C36 1843 2.131 2.203 4.060

14POPC H6X 1844 2.182 2.110 4.095

14POPC H6Y 1845 2.209 2.277 4.031

14POPC C37 1846 2.052 2.169 3.933

14POPC H7X 1847 1.979 2.251 3.915

14POPC H7Y 1848 1.994 2.075 3.946

14POPC C38 1849 2.142 2.162 3.810

14POPC H8X 1850 2.205 2.254 3.807

14POPC H8Y 1851 2.076 2.162 3.721

14POPC C39 1852 2.237 2.042 3.800

14POPC H9X 1853 2.179 1.949 3.819

14POPC H9Y 1854 2.314 2.051 3.879

14POPC C310 1855 2.305 2.031 3.661

14POPC H10X 1856 2.386 1.956 3.667

14POPC H10Y 1857 2.350 2.129 3.634

14POPC C311 1858 2.207 1.988 3.551

14POPC H11X 1859 2.126 2.063 3.540

14POPC H11Y 1860 2.161 1.892 3.584

14POPC C312 1861 2.268 1.962 3.413

14POPC H12X 1862 2.347 1.885 3.425

14POPC H12Y 1863 2.314 2.056 3.374

14POPC C313 1864 2.161 1.912 3.314

14POPC H13X 1865 2.095 1.998 3.289

14POPC H13Y 1866 2.096 1.837 3.364

14POPC C314 1867 2.210 1.844 3.186

14POPC H14X 1868 2.232 1.737 3.208

14POPC H14Y 1869 2.305 1.890 3.150

14POPC C315 1870 2.106 1.851 3.072

14POPC H15X 1871 2.141 1.790 2.986

14POPC H15Y 1872 2.102 1.956 3.036

14POPC C316 1873 1.963 1.811 3.111

14POPC H16X 1874 1.893 1.850 3.034

14POPC H16Y 1875 1.932 1.856 3.207

14POPC H16Z 1876 1.953 1.701 3.117

15POPC N 1877 3.677 1.476 4.956

15POPC C12 1878 3.695 1.627 4.962

15POPC H12A 1879 3.746 1.658 4.872

15POPC H12B 1880 3.762 1.655 5.041

15POPC C13 1881 3.634 1.437 4.817

15POPC H13A 1882 3.541 1.486 4.795

15POPC H13B 1883 3.708 1.466 4.744

15POPC H13C 1884 3.619 1.331 4.811

15POPC C14 1885 3.807 1.408 4.985

15POPC H14A 1886 3.797 1.301 4.977

15POPC H14B 1887 3.882 1.433 4.912

15POPC H14C 1888 3.848 1.430 5.082

15POPC C15 1889 3.569 1.425 5.046

15POPC H15A 1890 3.587 1.447 5.151

15POPC H15B 1891 3.558 1.318 5.034

15POPC H15C 1892 3.475 1.468 5.018

15POPC C11 1893 3.567 1.714 4.973

15POPC H11A 1894 3.596 1.821 4.965

15POPC H11B 1895 3.523 1.700 5.074

15POPC P 1896 3.325 1.651 4.891

15POPC O13 1897 3.286 1.701 5.025

15POPC O14 1898 3.305 1.506 4.865

15POPC O12 1899 3.478 1.682 4.867

15POPC O11 1900 3.256 1.736 4.778

15POPC C1 1901 3.316 1.862 4.747

15POPC HA 1902 3.420 1.850 4.710

15POPC HB 1903 3.317 1.928 4.836

15POPC C2 1904 3.240 1.938 4.635

15POPC HS 1905 3.135 1.952 4.669

15POPC O21 1906 3.249 1.864 4.513

15POPC C21 1907 3.141 1.801 4.471

15POPC O22 1908 3.035 1.791 4.529

15POPC C22 1909 3.172 1.739 4.335

15POPC H2R 1910 3.174 1.629 4.347

15POPC H2S 1911 3.274 1.772 4.306

15POPC C3 1912 3.306 2.078 4.615

15POPC HX 1913 3.293 2.136 4.709

15POPC HY 1914 3.250 2.132 4.536

15POPC O31 1915 3.447 2.063 4.588

15POPC C31 1916 3.491 2.110 4.472

15POPC O32 1917 3.440 2.199 4.407

15POPC C32 1918 3.619 2.035 4.434

15POPC H2X 1919 3.623 1.942 4.494

15POPC H2Y 1920 3.706 2.099 4.460

15POPC C23 1921 3.073 1.778 4.222

15POPC H3R 1922 3.009 1.862 4.253

15POPC H3S 1923 3.006 1.691 4.202

15POPC C24 1924 3.143 1.818 4.090

15POPC H4R 1925 3.186 1.920 4.100

15POPC H4S 1926 3.066 1.827 4.011

15POPC C25 1927 3.258 1.726 4.047

15POPC H5R 1928 3.274 1.646 4.122

15POPC H5S 1929 3.352 1.785 4.040

15POPC C26 1930 3.232 1.655 3.914

15POPC H6R 1931 3.313 1.583 3.895

15POPC H6S 1932 3.230 1.728 3.830

15POPC C27 1933 3.098 1.582 3.922

15POPC H7R 1934 3.015 1.651 3.897

15POPC H7S 1935 3.083 1.548 4.026

15POPC C28 1936 3.092 1.458 3.834

15POPC H8R 1937 2.993 1.412 3.858

15POPC H8S 1938 3.170 1.385 3.864

15POPC C29 1939 3.095 1.491 3.688

15POPC H91 1940 3.012 1.553 3.653

15POPC C210 1941 3.187 1.449 3.601

15POPC H101 1942 3.172 1.481 3.497

15POPC C211 1943 3.311 1.365 3.625

15POPC H11R 1944 3.284 1.257 3.629

15POPC H11S 1945 3.360 1.393 3.720

15POPC C212 1946 3.407 1.389 3.507

15POPC H12R 1947 3.437 1.496 3.510

15POPC H12S 1948 3.353 1.370 3.412

15POPC C213 1949 3.536 1.305 3.502

15POPC H13R 1950 3.509 1.199 3.521

15POPC H13S 1951 3.605 1.338 3.583

15POPC C214 1952 3.606 1.316 3.365

15POPC H14R 1953 3.535 1.280 3.287

15POPC H14S 1954 3.695 1.248 3.364

15POPC C215 1955 3.652 1.458 3.328

15POPC H15R 1956 3.741 1.486 3.387

15POPC H15S 1957 3.569 1.527 3.356

15POPC C216 1958 3.675 1.485 3.178

15POPC H16R 1959 3.584 1.459 3.121

15POPC H16S 1960 3.756 1.420 3.138

15POPC C217 1961 3.718 1.629 3.145

15POPC H17R 1962 3.653 1.700 3.202

15POPC H17S 1963 3.699 1.646 3.037

15POPC C218 1964 3.865 1.658 3.173

15POPC H18R 1965 3.889 1.636 3.280

15POPC H18S 1966 3.890 1.764 3.152

15POPC H18T 1967 3.930 1.594 3.111

15POPC C33 1968 3.622 1.996 4.284

15POPC H3X 1969 3.524 1.951 4.255

15POPC H3Y 1970 3.699 1.918 4.270

15POPC C34 1971 3.656 2.113 4.190

15POPC H4X 1972 3.745 2.167 4.230

15POPC H4Y 1973 3.572 2.185 4.188

15POPC C35 1974 3.687 2.069 4.046

15POPC H5X 1975 3.775 2.001 4.048

15POPC H5Y 1976 3.715 2.158 3.986

15POPC C36 1977 3.571 1.997 3.977

15POPC H6X 1978 3.484 2.065 3.969

15POPC H6Y 1979 3.539 1.909 4.038

15POPC C37 1980 3.607 1.947 3.837

15POPC H7X 1981 3.699 1.885 3.843

15POPC H7Y 1982 3.628 2.034 3.772

15POPC C38 1983 3.495 1.863 3.775

15POPC H8X 1984 3.401 1.922 3.779

15POPC H8Y 1985 3.479 1.771 3.835

15POPC C39 1986 3.521 1.825 3.629

15POPC H9X 1987 3.594 1.741 3.625

15POPC H9Y 1988 3.566 1.911 3.575

15POPC C310 1989 3.392 1.785 3.557

15POPC H10X 1990 3.319 1.869 3.568

15POPC H10Y 1991 3.348 1.696 3.608

15POPC C311 1992 3.408 1.758 3.407

15POPC H11X 1993 3.502 1.701 3.391

15POPC H11Y 1994 3.424 1.855 3.356

15POPC C312 1995 3.286 1.684 3.351

15POPC H12X 1996 3.195 1.728 3.397

15POPC H12Y 1997 3.291 1.578 3.382

15POPC C313 1998 3.267 1.682 3.199

15POPC H13X 1999 3.274 1.784 3.156

15POPC H13Y 2000 3.164 1.644 3.180

15POPC C314 2001 3.363 1.588 3.128

15POPC H14X 2002 3.380 1.497 3.189

15POPC H14Y 2003 3.459 1.642 3.118

15POPC C315 2004 3.316 1.545 2.988

15POPC H15X 2005 3.407 1.521 2.929

15POPC H15Y 2006 3.266 1.630 2.937

15POPC C316 2007 3.227 1.421 2.987

15POPC H16X 2008 3.274 1.338 3.044

15POPC H16Y 2009 3.209 1.387 2.883

15POPC H16Z 2010 3.128 1.443 3.034

16POPC N 2011 4.187 2.093 4.956

16POPC C12 2012 4.322 2.090 4.885

16POPC H12A 2013 4.399 2.114 4.957

16POPC H12B 2014 4.322 2.163 4.806

16POPC C13 2015 4.184 1.993 5.067

16POPC H13A 2016 4.088 1.991 5.117

16POPC H13B 2017 4.199 1.894 5.027

16POPC H13C 2018 4.263 2.008 5.140

16POPC C14 2019 4.156 2.227 5.015

16POPC H14A 2020 4.231 2.256 5.087

16POPC H14B 2021 4.145 2.303 4.938

16POPC H14C 2022 4.062 2.221 5.068

16POPC C15 2023 4.077 2.056 4.860

16POPC H15A 2024 4.072 2.122 4.775

16POPC H15B 2025 3.982 2.056 4.911

16POPC H15C 2026 4.093 1.955 4.825

16POPC C11 2027 4.367 1.956 4.823

16POPC H11A 2028 4.456 1.973 4.758

16POPC H11B 2029 4.287 1.916 4.757

16POPC P 2030 4.358 1.715 4.921

16POPC O13 2031 4.211 1.716 4.943

16POPC O14 2032 4.442 1.636 5.014

16POPC O12 2033 4.406 1.866 4.927

16POPC O11 2034 4.389 1.681 4.770

16POPC C1 2035 4.523 1.651 4.729

16POPC HA 2036 4.559 1.561 4.782

16POPC HB 2037 4.592 1.734 4.754

16POPC C2 2038 4.531 1.618 4.577

16POPC HS 2039 4.635 1.584 4.557

16POPC O21 2040 4.500 1.727 4.488

16POPC C21 2041 4.568 1.838 4.493

16POPC O22 2042 4.661 1.866 4.567

16POPC C22 2043 4.512 1.933 4.387

16POPC H2R 2044 4.463 2.017 4.441

16POPC H2S 2045 4.435 1.881 4.327

16POPC C3 2046 4.437 1.500 4.539

16POPC HX 2047 4.453 1.416 4.610

16POPC HY 2048 4.468 1.467 4.437

16POPC O31 2049 4.299 1.543 4.540

16POPC C31 2050 4.213 1.464 4.480

16POPC O32 2051 4.238 1.357 4.428

16POPC C32 2052 4.074 1.529 4.484

16POPC H2X 2053 4.077 1.613 4.557

16POPC H2Y 2054 4.005 1.451 4.523

16POPC C23 2055 4.623 1.990 4.294

16POPC H3R 2056 4.701 2.040 4.354

16POPC H3S 2057 4.577 2.067 4.229

16POPC C24 2058 4.689 1.884 4.203

16POPC H4R 2059 4.745 1.811 4.266

16POPC H4S 2060 4.763 1.935 4.138

16POPC C25 2061 4.591 1.804 4.114

16POPC H5R 2062 4.529 1.739 4.180

16POPC H5S 2063 4.651 1.737 4.048

16POPC C26 2064 4.497 1.888 4.027

16POPC H6R 2065 4.432 1.951 4.091

16POPC H6S 2066 4.431 1.818 3.970

16POPC C27 2067 4.569 1.977 3.925

16POPC H7R 2068 4.630 1.912 3.859

16POPC H7S 2069 4.638 2.045 3.980

16POPC C28 2070 4.474 2.061 3.838

16POPC H8R 2071 4.388 1.997 3.808

16POPC H8S 2072 4.526 2.085 3.743

16POPC C29 2073 4.432 2.190 3.904

16POPC H91 2074 4.505 2.272 3.896

16POPC C210 2075 4.315 2.213 3.967

16POPC H101 2076 4.296 2.313 4.007

16POPC C211 2077 4.204 2.114 3.984

16POPC H11R 2078 4.166 2.117 4.089

16POPC H11S 2079 4.238 2.009 3.966

16POPC C212 2080 4.087 2.143 3.888

16POPC H12R 2081 4.048 2.245 3.906

16POPC H12S 2082 4.005 2.071 3.910

16POPC C213 2083 4.125 2.128 3.739

16POPC H13R 2084 4.171 2.029 3.724

16POPC H13S 2085 4.200 2.205 3.712

16POPC C214 2086 4.004 2.142 3.646

16POPC H14R 2087 3.967 2.246 3.646

16POPC H14S 2088 3.922 2.077 3.686

16POPC C215 2089 4.032 2.095 3.503

16POPC H15R 2090 3.936 2.088 3.448

16POPC H15S 2091 4.076 1.993 3.506

16POPC C216 2092 4.127 2.185 3.421

16POPC H16R 2093 4.226 2.189 3.471

16POPC H16S 2094 4.086 2.288 3.417

16POPC C217 2095 4.144 2.131 3.278

16POPC H17R 2096 4.044 2.127 3.229

16POPC H17S 2097 4.180 2.026 3.285

16POPC C218 2098 4.240 2.212 3.191

16POPC H18R 2099 4.201 2.315 3.177

16POPC H18S 2100 4.250 2.165 3.091

16POPC H18T 2101 4.340 2.218 3.238

16POPC C33 2102 4.026 1.578 4.345

16POPC H3X 2103 3.918 1.601 4.352

16POPC H3Y 2104 4.037 1.496 4.270

16POPC C34 2105 4.098 1.705 4.296

16POPC H4X 2106 4.204 1.681 4.273

16POPC H4Y 2107 4.098 1.780 4.378

16POPC C35 2108 4.031 1.769 4.173

16POPC H5X 2109 4.080 1.866 4.151

16POPC H5Y 2110 3.925 1.790 4.197

16POPC C36 2111 4.035 1.682 4.046

16POPC H6X 2112 3.965 1.726 3.971

16POPC H6Y 2113 4.000 1.579 4.067

16POPC C37 2114 4.174 1.677 3.982

16POPC H7X 2115 4.249 1.637 4.054

16POPC H7Y 2116 4.205 1.780 3.955

16POPC C38 2117 4.177 1.591 3.855

16POPC H8X 2118 4.094 1.623 3.788

16POPC H8Y 2119 4.161 1.484 3.880

16POPC C39 2120 4.309 1.606 3.777

16POPC H9X 2121 4.388 1.546 3.826

16POPC H9Y 2122 4.340 1.713 3.781

16POPC C310 2123 4.295 1.567 3.630

16POPC H10X 2124 4.232 1.643 3.579

16POPC H10Y 2125 4.244 1.469 3.621

16POPC C311 2126 4.429 1.558 3.555

16POPC H11X 2127 4.410 1.546 3.446

16POPC H11Y 2128 4.476 1.463 3.588

16POPC C312 2129 4.527 1.675 3.579

16POPC H12X 2130 4.627 1.645 3.541

16POPC H12Y 2131 4.539 1.693 3.688

16POPC C313 2132 4.489 1.805 3.509

16POPC H13X 2133 4.556 1.886 3.545

16POPC H13Y 2134 4.385 1.833 3.535

16POPC C314 2135 4.503 1.794 3.357

16POPC H14X 2136 4.428 1.720 3.320

16POPC H14Y 2137 4.604 1.754 3.331

16POPC C315 2138 4.480 1.925 3.283

16POPC H15X 2139 4.568 1.991 3.297

16POPC H15Y 2140 4.392 1.976 3.328

16POPC C316 2141 4.454 1.903 3.134

16POPC H16X 2142 4.358 1.849 3.120

16POPC H16Y 2143 4.535 1.841 3.089

16POPC H16Z 2144 4.449 2.000 3.080

17POPC N 2145 1.395 2.340 4.845

17POPC C12 2146 1.462 2.471 4.806

17POPC H12A 2147 1.394 2.552 4.823

17POPC H12B 2148 1.484 2.469 4.700

17POPC C13 2149 1.278 2.362 4.935

17POPC H13A 2150 1.233 2.269 4.964

17POPC H13B 2151 1.310 2.414 5.024

17POPC H13C 2152 1.202 2.423 4.886

17POPC C14 2153 1.345 2.271 4.721

17POPC H14A 2154 1.264 2.327 4.677

17POPC H14B 2155 1.422 2.263 4.646

17POPC H14C 2156 1.308 2.172 4.742

17POPC C15 2157 1.492 2.257 4.924

17POPC H15A 2158 1.583 2.242 4.868

17POPC H15B 2159 1.451 2.161 4.953

17POPC H15C 2160 1.522 2.312 5.011

17POPC C11 2161 1.594 2.507 4.879

17POPC H11A 2162 1.631 2.606 4.843

17POPC H11B 2163 1.674 2.435 4.850

17POPC P 2164 1.498 2.622 5.092

17POPC O13 2165 1.593 2.718 5.153

17POPC O14 2166 1.400 2.561 5.185

17POPC O12 2167 1.579 2.508 5.021

17POPC O11 2168 1.417 2.690 4.975

17POPC C1 2169 1.275 2.702 4.986

17POPC HA 2170 1.246 2.753 5.081

17POPC HB 2171 1.224 2.604 4.986

17POPC C2 2172 1.213 2.786 4.871

17POPC HS 2173 1.103 2.783 4.885

17POPC O21 2174 1.245 2.736 4.740

17POPC C21 2175 1.178 2.633 4.694

17POPC O22 2176 1.110 2.556 4.761

17POPC C22 2177 1.206 2.622 4.544

17POPC H2R 2178 1.311 2.588 4.535

17POPC H2S 2179 1.201 2.724 4.499

17POPC C3 2180 1.260 2.934 4.884

17POPC HX 2181 1.221 2.970 4.982

17POPC HY 2182 1.212 2.999 4.807

17POPC O31 2183 1.404 2.947 4.882

17POPC C31 2184 1.455 2.930 4.759

17POPC O32 2185 1.395 2.945 4.655

17POPC C32 2186 1.603 2.894 4.770

17POPC H2X 2187 1.614 2.808 4.840

17POPC H2Y 2188 1.653 2.982 4.815

17POPC C23 2189 1.117 2.520 4.468

17POPC H3R 2190 1.099 2.433 4.534

17POPC H3S 2191 1.173 2.485 4.378

17POPC C24 2192 0.979 2.568 4.417

17POPC H4R 2193 0.917 2.593 4.505

17POPC H4S 2194 0.927 2.485 4.364

17POPC C25 2195 0.982 2.690 4.323

17POPC H5R 2196 1.061 2.760 4.357

17POPC H5S 2197 0.883 2.740 4.330

17POPC C26 2198 1.011 2.666 4.176

17POPC H6R 2199 1.074 2.574 4.168

17POPC H6S 2200 1.071 2.751 4.139

17POPC C27 2201 0.883 2.663 4.090

17POPC H7R 2202 0.840 2.766 4.090

17POPC H7S 2203 0.808 2.595 4.136

17POPC C28 2204 0.909 2.622 3.944

17POPC H8R 2205 0.974 2.697 3.895

17POPC H8S 2206 0.813 2.617 3.887

17POPC C29 2207 0.973 2.487 3.953

17POPC H91 2208 0.911 2.416 4.010

17POPC C210 2209 1.098 2.450 3.921

17POPC H101 2210 1.128 2.346 3.940

17POPC C211 2211 1.201 2.530 3.846

17POPC H11R 2212 1.290 2.551 3.909

17POPC H11S 2213 1.160 2.628 3.813

17POPC C212 2214 1.240 2.450 3.722

17POPC H12R 2215 1.281 2.351 3.753

17POPC H12S 2216 1.322 2.503 3.668

17POPC C213 2217 1.122 2.430 3.625

17POPC H13R 2218 1.096 2.529 3.581

17POPC H13S 2219 1.032 2.394 3.679

17POPC C214 2220 1.151 2.330 3.514

17POPC H14R 2221 1.253 2.349 3.473

17POPC H14S 2222 1.078 2.344 3.432

17POPC C215 2223 1.148 2.185 3.560

17POPC H15R 2224 1.048 2.156 3.599

17POPC H15S 2225 1.220 2.176 3.644

17POPC C216 2226 1.188 2.088 3.450

17POPC H16R 2227 1.229 1.997 3.498

17POPC H16S 2228 1.268 2.133 3.388

17POPC C217 2229 1.073 2.039 3.363

17POPC H17R 2230 1.028 2.130 3.319

17POPC H17S 2231 0.996 1.990 3.426

17POPC C218 2232 1.118 1.944 3.252

17POPC H18R 2233 1.157 1.849 3.295

17POPC H18S 2234 1.198 1.991 3.192

17POPC H18T 2235 1.033 1.920 3.184

17POPC C33 2236 1.667 2.864 4.632

17POPC H3X 2237 1.776 2.880 4.641

17POPC H3Y 2238 1.630 2.937 4.556

17POPC C34 2239 1.648 2.723 4.576

17POPC H4X 2240 1.540 2.701 4.560

17POPC H4Y 2241 1.689 2.649 4.649

17POPC C35 2242 1.725 2.703 4.444

17POPC H5X 2243 1.734 2.594 4.422

17POPC H5Y 2244 1.828 2.743 4.458

17POPC C36 2245 1.662 2.772 4.322

17POPC H6X 2246 1.736 2.768 4.240

17POPC H6Y 2247 1.643 2.879 4.342

17POPC C37 2248 1.534 2.704 4.272

17POPC H7X 2249 1.447 2.751 4.323

17POPC H7Y 2250 1.535 2.596 4.300

17POPC C38 2251 1.518 2.713 4.120

17POPC H8X 2252 1.416 2.678 4.093

17POPC H8Y 2253 1.591 2.646 4.071

17POPC C39 2254 1.539 2.856 4.066

17POPC H9X 2255 1.646 2.885 4.073

17POPC H9Y 2256 1.480 2.926 4.129

17POPC C310 2257 1.491 2.872 3.922

17POPC H10X 2258 1.498 2.979 3.891

17POPC H10Y 2259 1.384 2.844 3.920

17POPC C311 2260 1.553 2.782 3.816

17POPC H11X 2261 1.539 2.676 3.843

17POPC H11Y 2262 1.662 2.804 3.808

17POPC C312 2263 1.483 2.808 3.682

17POPC H12X 2264 1.473 2.918 3.670

17POPC H12Y 2265 1.380 2.767 3.683

17POPC C313 2266 1.560 2.756 3.560

17POPC H13X 2267 1.547 2.646 3.551

17POPC H13Y 2268 1.668 2.777 3.573

17POPC C314 2269 1.511 2.828 3.434

17POPC H14X 2270 1.508 2.937 3.451

17POPC H14Y 2271 1.407 2.796 3.414

17POPC C315 2272 1.599 2.803 3.313

17POPC H15X 2273 1.629 2.696 3.310

17POPC H15Y 2274 1.694 2.861 3.322

17POPC C316 2275 1.527 2.840 3.184

17POPC H16X 2276 1.574 2.785 3.099

17POPC H16Y 2277 1.532 2.949 3.165

17POPC H16Z 2278 1.420 2.810 3.188

18POPC N 2279 2.642 1.444 4.986

18POPC C12 2280 2.742 1.413 4.875

18POPC H12A 2281 2.831 1.369 4.919

18POPC H12B 2282 2.775 1.505 4.829

18POPC C13 2283 2.507 1.460 4.925

18POPC H13A 2284 2.431 1.478 4.999

18POPC H13B 2285 2.507 1.538 4.850

18POPC H13C 2286 2.482 1.368 4.874

18POPC C14 2287 2.637 1.328 5.081

18POPC H14A 2288 2.734 1.310 5.125

18POPC H14B 2289 2.563 1.340 5.158

18POPC H14C 2290 2.616 1.238 5.024

18POPC C15 2291 2.682 1.567 5.061

18POPC H15A 2292 2.609 1.591 5.137

18POPC H15B 2293 2.693 1.651 4.994

18POPC H15C 2294 2.777 1.552 5.110

18POPC C11 2295 2.692 1.319 4.762

18POPC H11A 2296 2.775 1.305 4.689

18POPC H11B 2297 2.612 1.369 4.704

18POPC P 2298 2.744 1.078 4.848

18POPC O13 2299 2.668 0.951 4.858

18POPC O14 2300 2.817 1.119 4.970

18POPC O12 2301 2.642 1.194 4.812

18POPC O11 2302 2.841 1.074 4.724

18POPC C1 2303 2.972 1.130 4.725

18POPC HA 2304 3.044 1.056 4.766

18POPC HB 2305 2.978 1.221 4.789

18POPC C2 2306 3.022 1.172 4.583

18POPC HS 2307 3.126 1.207 4.597

18POPC O21 2308 2.938 1.277 4.531

18POPC C21 2309 2.995 1.360 4.446

18POPC O22 2310 3.114 1.364 4.417

18POPC C22 2311 2.888 1.455 4.392

18POPC H2R 2312 2.926 1.559 4.405

18POPC H2S 2313 2.795 1.443 4.451

18POPC C3 2314 3.025 1.052 4.483

18POPC HX 2315 3.095 0.978 4.527

18POPC HY 2316 3.069 1.086 4.387

18POPC O31 2317 2.896 0.990 4.468

18POPC C31 2318 2.825 1.022 4.361

18POPC O32 2319 2.866 1.082 4.263

18POPC C32 2320 2.679 0.980 4.385

18POPC H2X 2321 2.652 1.023 4.484

18POPC H2Y 2322 2.674 0.869 4.393

18POPC C23 2323 2.859 1.429 4.242

18POPC H3R 2324 2.818 1.326 4.231

18POPC H3S 2325 2.954 1.433 4.184

18POPC C24 2326 2.760 1.529 4.182

18POPC H4R 2327 2.793 1.632 4.208

18POPC H4S 2328 2.659 1.514 4.227

18POPC C25 2329 2.753 1.519 4.029

18POPC H5R 2330 2.856 1.532 3.989

18POPC H5S 2331 2.691 1.602 3.987

18POPC C26 2332 2.700 1.385 3.979

18POPC H6R 2333 2.590 1.385 3.992

18POPC H6S 2334 2.741 1.301 4.039

18POPC C27 2335 2.731 1.356 3.831

18POPC H7R 2336 2.780 1.445 3.785

18POPC H7S 2337 2.633 1.345 3.780

18POPC C28 2338 2.816 1.230 3.801

18POPC H8R 2339 2.915 1.236 3.853

18POPC H8S 2340 2.838 1.228 3.692

18POPC C29 2341 2.754 1.102 3.847

18POPC H91 2342 2.763 1.085 3.956

18POPC C210 2343 2.690 1.010 3.773

18POPC H101 2344 2.649 0.922 3.825

18POPC C211 2345 2.670 1.009 3.625

18POPC H11R 2346 2.724 1.092 3.574

18POPC H11S 2347 2.562 1.020 3.600

18POPC C212 2348 2.713 0.876 3.564

18POPC H12R 2349 2.659 0.792 3.614

18POPC H12S 2350 2.822 0.860 3.578

18POPC C213 2351 2.683 0.867 3.416

18POPC H13R 2352 2.577 0.894 3.399

18POPC H13S 2353 2.701 0.763 3.384

18POPC C214 2354 2.774 0.960 3.336

18POPC H14R 2355 2.879 0.938 3.363

18POPC H14S 2356 2.754 1.067 3.359

18POPC C215 2357 2.757 0.929 3.189

18POPC H15R 2358 2.657 0.958 3.149

18POPC H15S 2359 2.764 0.819 3.182

18POPC C216 2360 2.869 0.987 3.104

18POPC H16R 2361 2.875 0.922 3.014

18POPC H16S 2362 2.966 0.979 3.158

18POPC C217 2363 2.848 1.126 3.047

18POPC H17R 2364 2.899 1.202 3.111

18POPC H17S 2365 2.739 1.146 3.048

18POPC C218 2366 2.896 1.133 2.903

18POPC H18R 2367 2.992 1.078 2.893

18POPC H18S 2368 2.911 1.237 2.867

18POPC H18T 2369 2.823 1.083 2.836

18POPC C33 2370 2.579 1.032 4.279

18POPC H3X 2371 2.601 1.138 4.256

18POPC H3Y 2372 2.477 1.027 4.324

18POPC C34 2373 2.573 0.949 4.149

18POPC H4X 2374 2.560 0.842 4.173

18POPC H4Y 2375 2.672 0.959 4.098

18POPC C35 2376 2.461 0.993 4.052

18POPC H5X 2377 2.362 0.963 4.093

18POPC H5Y 2378 2.474 0.937 3.957

18POPC C36 2379 2.460 1.145 4.025

18POPC H6X 2380 2.563 1.171 3.990

18POPC H6Y 2381 2.444 1.198 4.120

18POPC C37 2382 2.360 1.204 3.923

18POPC H7X 2383 2.382 1.312 3.911

18POPC H7Y 2384 2.259 1.199 3.970

18POPC C38 2385 2.352 1.138 3.784

18POPC H8X 2386 2.247 1.113 3.761

18POPC H8Y 2387 2.408 1.042 3.789

18POPC C39 2388 2.410 1.217 3.665

18POPC H9X 2389 2.392 1.160 3.572

18POPC H9Y 2390 2.520 1.216 3.682

18POPC C310 2391 2.366 1.363 3.642

18POPC H10X 2392 2.388 1.424 3.732

18POPC H10Y 2393 2.256 1.366 3.626

18POPC C311 2394 2.436 1.429 3.521

18POPC H11X 2395 2.399 1.533 3.508

18POPC H11Y 2396 2.407 1.373 3.430

18POPC C312 2397 2.590 1.431 3.535

18POPC H12X 2398 2.624 1.328 3.557

18POPC H12Y 2399 2.617 1.496 3.622

18POPC C313 2400 2.672 1.475 3.414

18POPC H13X 2401 2.685 1.585 3.414

18POPC H13Y 2402 2.618 1.447 3.320

18POPC C314 2403 2.810 1.405 3.414

18POPC H14X 2404 2.795 1.295 3.412

18POPC H14Y 2405 2.862 1.430 3.508

18POPC C315 2406 2.898 1.445 3.295

18POPC H15X 2407 2.908 1.556 3.296

18POPC H15Y 2408 2.846 1.416 3.201

18POPC C316 2409 3.036 1.382 3.294

18POPC H16X 2410 3.060 1.341 3.395

18POPC H16Y 2411 3.114 1.456 3.267

18POPC H16Z 2412 3.043 1.299 3.221

19POPC N 2413 2.279 5.223 5.013

19POPC C12 2414 2.187 5.343 5.012

19POPC H12A 2415 2.242 5.429 4.975

19POPC H12B 2416 2.160 5.367 5.114

19POPC C13 2417 2.297 5.176 4.873

19POPC H13A 2418 2.357 5.086 4.871

19POPC H13B 2419 2.201 5.148 4.832

19POPC H13C 2420 2.341 5.251 4.810

19POPC C14 2421 2.413 5.257 5.070

19POPC H14A 2422 2.403 5.291 5.173

19POPC H14B 2423 2.477 5.170 5.070

19POPC H14C 2424 2.461 5.334 5.012

19POPC C15 2425 2.217 5.111 5.091

19POPC H15A 2426 2.199 5.138 5.194

19POPC H15B 2427 2.276 5.021 5.087

19POPC H15C 2428 2.121 5.086 5.047

19POPC C11 2429 2.056 5.333 4.930

19POPC H11A 2430 2.081 5.332 4.821

19POPC H11B 2431 2.002 5.428 4.950

19POPC P 2432 1.928 5.112 4.866

19POPC O13 2433 1.813 5.042 4.925

19POPC O14 2434 2.047 5.031 4.831

19POPC O12 2435 1.973 5.224 4.970

19POPC O11 2436 1.882 5.196 4.741

19POPC C1 2437 1.819 5.132 4.628

19POPC HA 2438 1.760 5.210 4.575

19POPC HB 2439 1.747 5.053 4.659

19POPC C2 2440 1.918 5.071 4.523

19POPC HS 2441 1.950 4.973 4.565

19POPC O21 2442 2.032 5.158 4.503

19POPC C21 2443 2.140 5.092 4.458

19POPC O22 2444 2.150 4.971 4.449

19POPC C22 2445 2.251 5.189 4.419

19POPC H2R 2446 2.346 5.146 4.458

19POPC H2S 2447 2.234 5.288 4.467

19POPC C3 2448 1.843 5.044 4.388

19POPC HX 2449 1.777 5.132 4.369

19POPC HY 2450 1.776 4.957 4.402

19POPC O31 2451 1.933 5.034 4.276

19POPC C31 2452 1.944 4.914 4.220

19POPC O32 2453 1.868 4.820 4.239

19POPC C32 2454 2.062 4.914 4.124

19POPC H2X 2455 2.156 4.914 4.184

19POPC H2Y 2456 2.058 5.009 4.066

19POPC C23 2457 2.270 5.202 4.266

19POPC H3R 2458 2.190 5.268 4.224

19POPC H3S 2459 2.261 5.103 4.218

19POPC C24 2460 2.409 5.256 4.230

19POPC H4R 2461 2.486 5.194 4.280

19POPC H4S 2462 2.420 5.359 4.270

19POPC C25 2463 2.439 5.256 4.078

19POPC H5R 2464 2.449 5.150 4.045

19POPC H5S 2465 2.536 5.306 4.061

19POPC C26 2466 2.331 5.323 3.991

19POPC H6R 2467 2.235 5.269 4.007

19POPC H6S 2468 2.358 5.312 3.884

19POPC C27 2469 2.313 5.472 4.024

19POPC H7R 2470 2.400 5.527 3.984

19POPC H7S 2471 2.315 5.483 4.134

19POPC C28 2472 2.182 5.534 3.971

19POPC H8R 2473 2.163 5.496 3.868

19POPC H8S 2474 2.196 5.644 3.962

19POPC C29 2475 2.068 5.506 4.065

19POPC H91 2476 2.069 5.565 4.158

19POPC C210 2477 1.972 5.414 4.049

19POPC H101 2478 1.895 5.402 4.127

19POPC C211 2479 1.958 5.321 3.932

19POPC H11R 2480 1.917 5.223 3.965

19POPC H11S 2481 2.056 5.300 3.884

19POPC C212 2482 1.865 5.382 3.826

19POPC H12R 2483 1.907 5.479 3.793

19POPC H12S 2484 1.765 5.401 3.870

19POPC C213 2485 1.848 5.293 3.704

19POPC H13R 2486 1.750 5.312 3.656

19POPC H13S 2487 1.849 5.186 3.735

19POPC C214 2488 1.958 5.318 3.599

19POPC H14R 2489 2.057 5.297 3.645

19POPC H14S 2490 1.956 5.425 3.568

19POPC C215 2491 1.937 5.228 3.478

19POPC H15R 2492 1.842 5.254 3.427

19POPC H15S 2493 1.926 5.125 3.518

19POPC C216 2494 2.052 5.229 3.377

19POPC H16R 2495 2.032 5.149 3.303

19POPC H16S 2496 2.148 5.205 3.429

19POPC C217 2497 2.067 5.360 3.298

19POPC H17R 2498 2.096 5.442 3.367

19POPC H17S 2499 1.969 5.385 3.254

19POPC C218 2500 2.171 5.347 3.186

19POPC H18R 2501 2.272 5.330 3.229

19POPC H18S 2502 2.174 5.440 3.125

19POPC H18T 2503 2.146 5.262 3.120

19POPC C33 2504 2.063 4.793 4.028

19POPC H3X 2505 1.962 4.748 4.023

19POPC H3Y 2506 2.131 4.714 4.066

19POPC C34 2507 2.102 4.833 3.885

19POPC H4X 2508 2.211 4.850 3.879

19POPC H4Y 2509 2.050 4.927 3.858

19POPC C35 2510 2.059 4.723 3.788

19POPC H5X 2511 1.948 4.710 3.795

19POPC H5Y 2512 2.106 4.628 3.821

19POPC C36 2513 2.096 4.749 3.642

19POPC H6X 2514 2.050 4.667 3.583

19POPC H6Y 2515 2.206 4.744 3.627

19POPC C37 2516 2.043 4.881 3.585

19POPC H7X 2517 2.120 4.960 3.596

19POPC H7Y 2518 1.954 4.912 3.643

19POPC C38 2519 2.001 4.865 3.438

19POPC H8X 2520 1.961 4.960 3.398

19POPC H8Y 2521 1.917 4.792 3.435

19POPC C39 2522 2.110 4.814 3.344

19POPC H9X 2523 2.062 4.793 3.247

19POPC H9Y 2524 2.153 4.718 3.381

19POPC C310 2525 2.223 4.915 3.325

19POPC H10X 2526 2.272 4.936 3.422

19POPC H10Y 2527 2.181 5.011 3.286

19POPC C311 2528 2.328 4.868 3.224

19POPC H11X 2529 2.380 4.779 3.267

19POPC H11Y 2530 2.403 4.949 3.210

19POPC C312 2531 2.274 4.828 3.088

19POPC H12X 2532 2.199 4.746 3.094

19POPC H12Y 2533 2.363 4.786 3.036

19POPC C313 2534 2.222 4.943 3.000

19POPC H13X 2535 2.281 5.035 3.020

19POPC H13Y 2536 2.116 4.964 3.024

19POPC C314 2537 2.238 4.903 2.853

19POPC H14X 2538 2.178 4.811 2.838

19POPC H14Y 2539 2.345 4.882 2.833

19POPC C315 2540 2.192 5.002 2.748

19POPC H15X 2541 2.086 5.024 2.774

19POPC H15Y 2542 2.194 4.952 2.649

19POPC C316 2543 2.271 5.131 2.738

19POPC H16X 2544 2.378 5.109 2.719

19POPC H16Y 2545 2.262 5.189 2.832

19POPC H16Z 2546 2.233 5.195 2.655

20POPC N 2547 0.908 3.992 5.008

20POPC C12 2548 0.931 4.016 4.859

20POPC H12A 2549 0.844 4.062 4.814

20POPC H12B 2550 0.943 3.921 4.809

20POPC C13 2551 0.775 3.925 5.027

20POPC H13A 2552 0.771 3.832 4.972

20POPC H13B 2553 0.758 3.905 5.132

20POPC H13C 2554 0.694 3.986 4.989

20POPC C14 2555 1.017 3.903 5.060

20POPC H14A 2556 1.011 3.889 5.167

20POPC H14B 2557 1.017 3.807 5.010

20POPC H14C 2558 1.112 3.947 5.035

20POPC C15 2559 0.916 4.120 5.085

20POPC H15A 2560 0.842 4.192 5.054

20POPC H15B 2561 1.014 4.164 5.069

20POPC H15C 2562 0.906 4.104 5.191

20POPC C11 2563 1.056 4.099 4.819

20POPC H11A 2564 1.054 4.199 4.866

20POPC H11B 2565 1.055 4.116 4.709

20POPC P 2566 1.222 3.904 4.774

20POPC O13 2567 1.105 3.852 4.700

20POPC O14 2568 1.282 3.811 4.873

20POPC O12 2569 1.174 4.031 4.858

20POPC O11 2570 1.329 3.964 4.675

20POPC C1 2571 1.385 4.096 4.686

20POPC HA 2572 1.495 4.088 4.697

20POPC HB 2573 1.349 4.150 4.776

20POPC C2 2574 1.354 4.189 4.564

20POPC HS 2575 1.363 4.293 4.602

20POPC O21 2576 1.219 4.166 4.517

20POPC C21 2577 1.172 4.253 4.430

20POPC O22 2578 1.228 4.355 4.392

20POPC C22 2579 1.029 4.215 4.392

20POPC H2R 2580 0.984 4.300 4.336

20POPC H2S 2581 0.972 4.203 4.486

20POPC C3 2582 1.460 4.175 4.450

20POPC HX 2583 1.560 4.195 4.493

20POPC HY 2584 1.444 4.255 4.374

20POPC O31 2585 1.452 4.048 4.382

20POPC C31 2586 1.533 3.954 4.426

20POPC O32 2587 1.618 3.966 4.513

20POPC C32 2588 1.513 3.827 4.346

20POPC H2X 2589 1.409 3.790 4.361

20POPC H2Y 2590 1.583 3.755 4.393

20POPC C23 2591 1.018 4.085 4.309

20POPC H3R 2592 0.912 4.052 4.311

20POPC H3S 2593 1.079 4.003 4.353

20POPC C24 2594 1.057 4.106 4.162

20POPC H4R 2595 1.166 4.122 4.154

20POPC H4S 2596 1.006 4.197 4.124

20POPC C25 2597 1.016 3.989 4.070

20POPC H5R 2598 0.907 3.972 4.079

20POPC H5S 2599 1.068 3.897 4.101

20POPC C26 2600 1.050 4.021 3.924

20POPC H6R 2601 1.160 4.037 3.918

20POPC H6S 2602 1.005 4.118 3.895

20POPC C27 2603 1.011 3.911 3.822

20POPC H7R 2604 1.050 3.813 3.855

20POPC H7S 2605 1.059 3.938 3.726

20POPC C28 2606 0.861 3.894 3.798

20POPC H8R 2607 0.814 3.864 3.894

20POPC H8S 2608 0.845 3.811 3.726

20POPC C29 2609 0.808 4.024 3.747

20POPC H91 2610 0.804 4.102 3.825

20POPC C210 2611 0.772 4.058 3.623

20POPC H101 2612 0.732 4.159 3.610

20POPC C211 2613 0.760 3.971 3.500

20POPC H11R 2614 0.653 3.981 3.472

20POPC H11S 2615 0.781 3.864 3.520

20POPC C212 2616 0.816 4.011 3.363

20POPC H12R 2617 0.827 4.121 3.354

20POPC H12S 2618 0.741 3.981 3.286

20POPC C213 2619 0.947 3.945 3.328

20POPC H13R 2620 0.988 3.897 3.419

20POPC H13S 2621 1.019 4.023 3.294

20POPC C214 2622 0.925 3.846 3.215

20POPC H14R 2623 0.888 3.900 3.124

20POPC H14S 2624 0.846 3.774 3.245

20POPC C215 2625 1.050 3.768 3.182

20POPC H15R 2626 1.133 3.839 3.161

20POPC H15S 2627 1.029 3.711 3.089

20POPC C216 2628 1.088 3.674 3.297

20POPC H16R 2629 1.003 3.606 3.318

20POPC H16S 2630 1.109 3.733 3.389

20POPC C217 2631 1.212 3.592 3.265

20POPC H17R 2632 1.238 3.531 3.354

20POPC H17S 2633 1.297 3.661 3.247

20POPC C218 2634 1.192 3.501 3.145

20POPC H18R 2635 1.209 3.396 3.176

20POPC H18S 2636 1.266 3.522 3.065

20POPC H18T 2637 1.091 3.511 3.101

20POPC C33 2638 1.548 3.840 4.195

20POPC H3X 2639 1.587 3.742 4.162

20POPC H3Y 2640 1.630 3.913 4.178

20POPC C34 2641 1.428 3.871 4.104

20POPC H4X 2642 1.424 3.980 4.084

20POPC H4Y 2643 1.333 3.843 4.154

20POPC C35 2644 1.436 3.792 3.971

20POPC H5X 2645 1.416 3.685 3.994

20POPC H5Y 2646 1.539 3.798 3.930

20POPC C36 2647 1.333 3.834 3.866

20POPC H6X 2648 1.356 3.938 3.834

20POPC H6Y 2649 1.237 3.837 3.922

20POPC C37 2650 1.318 3.737 3.746

20POPC H7X 2651 1.241 3.661 3.769

20POPC H7Y 2652 1.414 3.683 3.733

20POPC C38 2653 1.288 3.798 3.608

20POPC H8X 2654 1.187 3.845 3.607

20POPC H8Y 2655 1.286 3.716 3.534

20POPC C39 2656 1.392 3.901 3.564

20POPC H9X 2657 1.490 3.849 3.552

20POPC H9Y 2658 1.402 3.978 3.643

20POPC C310 2659 1.358 3.976 3.436

20POPC H10X 2660 1.319 3.904 3.360

20POPC H10Y 2661 1.451 4.021 3.396

20POPC C311 2662 1.258 4.090 3.460

20POPC H11X 2663 1.291 4.151 3.546

20POPC H11Y 2664 1.159 4.047 3.487

20POPC C312 2665 1.244 4.180 3.337

20POPC H12X 2666 1.218 4.116 3.250

20POPC H12Y 2667 1.342 4.229 3.316

20POPC C313 2668 1.135 4.287 3.351

20POPC H13X 2669 1.164 4.355 3.433

20POPC H13Y 2670 1.039 4.238 3.380

20POPC C314 2671 1.113 4.369 3.222

20POPC H14X 2672 1.211 4.391 3.175

20POPC H14Y 2673 1.069 4.466 3.254

20POPC C315 2674 1.017 4.306 3.120

20POPC H15X 2675 0.981 4.387 3.053

20POPC H15Y 2676 0.927 4.266 3.172

20POPC C316 2677 1.076 4.196 3.031

20POPC H16X 2678 1.090 4.102 3.088

20POPC H16Y 2679 1.174 4.228 2.989

20POPC H16Z 2680 1.007 4.174 2.946

21POPC N 2681 5.130 2.970 4.789

21POPC C12 2682 5.122 3.068 4.905

21POPC H12A 2683 5.026 3.057 4.952

21POPC H12B 2684 5.127 3.168 4.865

21POPC C13 2685 5.136 2.829 4.840

21POPC H13A 2686 5.046 2.807 4.895

21POPC H13B 2687 5.143 2.759 4.757

21POPC H13C 2688 5.222 2.817 4.903

21POPC C14 2689 5.010 2.983 4.701

21POPC H14A 2690 5.015 2.916 4.616

21POPC H14B 2691 4.919 2.960 4.754

21POPC H14C 2692 4.998 3.082 4.660

21POPC C15 2693 5.255 2.998 4.711

21POPC H15A 2694 5.253 3.096 4.667

21POPC H15B 2695 5.273 2.925 4.634

21POPC H15C 2696 5.339 2.999 4.779

21POPC C11 2697 5.233 3.060 5.013

21POPC H11A 2698 5.243 2.956 5.052

21POPC H11B 2699 5.200 3.124 5.097

21POPC P 2700 5.474 3.148 5.056

21POPC O13 2701 5.530 3.029 5.123

21POPC O14 2702 5.424 3.253 5.149

21POPC O12 2703 5.357 3.104 4.959

21POPC O11 2704 5.582 3.212 4.962

21POPC C1 2705 5.633 3.145 4.847

21POPC HA 2706 5.587 3.045 4.832

21POPC HB 2707 5.742 3.132 4.860

21POPC C2 2708 5.610 3.224 4.716

21POPC HS 2709 5.664 3.321 4.726

21POPC O21 2710 5.468 3.240 4.699

21POPC C21 2711 5.434 3.351 4.635

21POPC O22 2712 5.508 3.438 4.595

21POPC C22 2713 5.283 3.347 4.613

21POPC H2R 2714 5.245 3.451 4.624

21POPC H2S 2715 5.236 3.285 4.693

21POPC C3 2716 5.670 3.146 4.595

21POPC HX 2717 5.633 3.041 4.604

21POPC HY 2718 5.781 3.142 4.604

21POPC O31 2719 5.623 3.195 4.469

21POPC C31 2720 5.705 3.279 4.407

21POPC O32 2721 5.819 3.304 4.438

21POPC C32 2722 5.634 3.331 4.285

21POPC H2X 2723 5.552 3.398 4.318

21POPC H2Y 2724 5.591 3.242 4.234

21POPC C23 2725 5.249 3.293 4.472

21POPC H3R 2726 5.144 3.258 4.470

21POPC H3S 2727 5.315 3.206 4.448

21POPC C24 2728 5.266 3.401 4.365

21POPC H4R 2729 5.353 3.465 4.390

21POPC H4S 2730 5.177 3.467 4.364

21POPC C25 2731 5.290 3.344 4.224

21POPC H5R 2732 5.384 3.285 4.225

21POPC H5S 2733 5.305 3.429 4.154

21POPC C26 2734 5.179 3.253 4.172

21POPC H6R 2735 5.081 3.301 4.195

21POPC H6S 2736 5.181 3.156 4.225

21POPC C27 2737 5.191 3.232 4.020

21POPC H7R 2738 5.185 3.331 3.972

21POPC H7S 2739 5.104 3.173 3.985

21POPC C28 2740 5.322 3.165 3.974

21POPC H8R 2741 5.408 3.222 4.015

21POPC H8S 2742 5.328 3.177 3.864

21POPC C29 2743 5.330 3.021 4.017

21POPC H91 2744 5.337 3.006 4.126

21POPC C210 2745 5.330 2.913 3.937

21POPC H101 2746 5.338 2.813 3.982

21POPC C211 2747 5.322 2.913 3.786

21POPC H11R 2748 5.417 2.875 3.744

21POPC H11S 2749 5.310 3.015 3.744

21POPC C212 2750 5.210 2.823 3.733

21POPC H12R 2751 5.221 2.720 3.774

21POPC H12S 2752 5.221 2.815 3.623

21POPC C213 2753 5.069 2.876 3.764

21POPC H13R 2754 5.052 2.873 3.874

21POPC H13S 2755 4.997 2.807 3.716

21POPC C214 2756 5.041 3.020 3.715

21POPC H14R 2757 5.100 3.091 3.777

21POPC H14S 2758 4.934 3.043 3.731

21POPC C215 2759 5.077 3.043 3.568

21POPC H15R 2760 5.187 3.027 3.555

21POPC H15S 2761 5.058 3.149 3.541

21POPC C216 2762 5.001 2.951 3.471

21POPC H16R 2763 4.898 2.990 3.459

21POPC H16S 2764 4.993 2.848 3.511

21POPC C217 2765 5.070 2.942 3.336

21POPC H17R 2766 5.110 3.042 3.309

21POPC H17S 2767 4.994 2.912 3.260

21POPC C218 2768 5.183 2.841 3.330

21POPC H18R 2769 5.148 2.740 3.362

21POPC H18S 2770 5.267 2.872 3.397

21POPC H18T 2771 5.221 2.834 3.226

21POPC C33 2772 5.726 3.406 4.188

21POPC H3X 2773 5.825 3.356 4.181

21POPC H3Y 2774 5.745 3.509 4.222

21POPC C34 2775 5.662 3.405 4.050

21POPC H4X 2776 5.579 3.479 4.046

21POPC H4Y 2777 5.617 3.305 4.030

21POPC C35 2778 5.763 3.431 3.939

21POPC H5X 2779 5.842 3.354 3.946

21POPC H5Y 2780 5.811 3.530 3.953

21POPC C36 2781 5.695 3.421 3.803

21POPC H6X 2782 5.597 3.474 3.806

21POPC H6Y 2783 5.671 3.314 3.788

21POPC C37 2784 5.781 3.480 3.692

21POPC H7X 2785 5.876 3.421 3.687

21POPC H7Y 2786 5.813 3.582 3.722

21POPC C38 2787 5.714 3.489 3.554

21POPC H8X 2788 5.793 3.488 3.476

21POPC H8Y 2789 5.671 3.591 3.541

21POPC C39 2790 5.598 3.392 3.524

21POPC H9X 2791 5.561 3.417 3.422

21POPC H9Y 2792 5.515 3.417 3.594

21POPC C310 2793 5.622 3.240 3.531

21POPC H10X 2794 5.671 3.210 3.626

21POPC H10Y 2795 5.692 3.209 3.451

21POPC C311 2796 5.492 3.159 3.522

21POPC H11X 2797 5.428 3.182 3.609

21POPC H11Y 2798 5.517 3.051 3.527

21POPC C312 2799 5.414 3.187 3.393

21POPC H12X 2800 5.371 3.289 3.399

21POPC H12Y 2801 5.331 3.114 3.383

21POPC C313 2802 5.506 3.179 3.270

21POPC H13X 2803 5.553 3.078 3.274

21POPC H13Y 2804 5.587 3.254 3.276

21POPC C314 2805 5.439 3.198 3.135

21POPC H14X 2806 5.393 3.299 3.132

21POPC H14Y 2807 5.358 3.122 3.127

21POPC C315 2808 5.542 3.185 3.021

21POPC H15X 2809 5.641 3.161 3.066

21POPC H15Y 2810 5.553 3.285 2.976

21POPC C316 2811 5.515 3.081 2.911

21POPC H16X 2812 5.573 3.106 2.820

21POPC H16Y 2813 5.409 3.078 2.878

21POPC H16Z 2814 5.543 2.978 2.943

22POPC N 2815 2.894 5.520 5.129

22POPC C12 2816 2.990 5.555 5.017

22POPC H12A 2817 3.093 5.551 5.050

22POPC H12B 2818 2.975 5.658 4.986

22POPC C13 2819 2.895 5.371 5.145

22POPC H13A 2820 2.994 5.334 5.166

22POPC H13B 2821 2.826 5.336 5.220

22POPC H13C 2822 2.868 5.327 5.050

22POPC C14 2823 2.931 5.588 5.257

22POPC H14A 2824 2.931 5.695 5.247

22POPC H14B 2825 2.861 5.562 5.336

22POPC H14C 2826 3.030 5.558 5.289

22POPC C15 2827 2.755 5.561 5.088

22POPC H15A 2828 2.736 5.519 4.991

22POPC H15B 2829 2.746 5.668 5.078

22POPC H15C 2830 2.680 5.524 5.157

22POPC C11 2831 2.980 5.468 4.889

22POPC H11A 2832 3.016 5.364 4.909

22POPC H11B 2833 3.051 5.510 4.814

22POPC P 2834 2.810 5.440 4.692

22POPC O13 2835 2.672 5.489 4.670

22POPC O14 2836 2.842 5.301 4.658

22POPC O12 2837 2.845 5.465 4.844

22POPC O11 2838 2.913 5.538 4.623

22POPC C1 2839 2.908 5.679 4.648

22POPC HA 2840 2.980 5.707 4.729

22POPC HB 2841 2.807 5.712 4.678

22POPC C2 2842 2.945 5.758 4.520

22POPC HS 2843 2.897 5.858 4.526

22POPC O21 2844 2.901 5.684 4.406

22POPC C21 2845 2.772 5.692 4.385

22POPC O22 2846 2.700 5.782 4.425

22POPC C22 2847 2.730 5.574 4.298

22POPC H2R 2848 2.637 5.531 4.342

22POPC H2S 2849 2.809 5.496 4.298

22POPC C3 2850 3.096 5.783 4.506

22POPC HX 2851 3.132 5.838 4.596

22POPC HY 2852 3.106 5.853 4.420

22POPC O31 2853 3.175 5.663 4.491

22POPC C31 2854 3.182 5.615 4.367

22POPC O32 2855 3.134 5.665 4.267

22POPC C32 2856 3.270 5.491 4.366

22POPC H2X 2857 3.321 5.481 4.465

22POPC H2Y 2858 3.347 5.504 4.287

22POPC C23 2859 2.703 5.624 4.155

22POPC H3R 2860 2.629 5.707 4.158

22POPC H3S 2861 2.660 5.543 4.094

22POPC C24 2862 2.831 5.676 4.088

22POPC H4R 2863 2.894 5.589 4.061

22POPC H4S 2864 2.891 5.740 4.156

22POPC C25 2865 2.797 5.757 3.964

22POPC H5R 2866 2.757 5.856 3.992

22POPC H5S 2867 2.717 5.701 3.910

22POPC C26 2868 2.920 5.770 3.875

22POPC H6R 2869 2.952 5.666 3.852

22POPC H6S 2870 3.001 5.820 3.932

22POPC C27 2871 2.896 5.848 3.744

22POPC H7R 2872 2.991 5.862 3.689

22POPC H7S 2873 2.862 5.950 3.773

22POPC C28 2874 2.786 5.789 3.654

22POPC H8R 2875 2.751 5.870 3.587

22POPC H8S 2876 2.701 5.770 3.724

22POPC C29 2877 2.798 5.664 3.573

22POPC H91 2878 2.703 5.608 3.567

22POPC C210 2879 2.905 5.611 3.511

22POPC H101 2880 2.896 5.515 3.458

22POPC C211 2881 3.040 5.672 3.502

22POPC H11R 2882 3.026 5.782 3.502

22POPC H11S 2883 3.085 5.647 3.403

22POPC C212 2884 3.137 5.637 3.616

22POPC H12R 2885 3.089 5.652 3.715

22POPC H12S 2886 3.222 5.709 3.610

22POPC C213 2887 3.199 5.498 3.614

22POPC H13R 2888 3.120 5.421 3.599

22POPC H13S 2889 3.250 5.478 3.711

22POPC C214 2890 3.303 5.492 3.502

22POPC H14R 2891 3.383 5.566 3.519

22POPC H14S 2892 3.248 5.523 3.411

22POPC C215 2893 3.363 5.353 3.479

22POPC H15R 2894 3.285 5.277 3.502

22POPC H15S 2895 3.448 5.337 3.549

22POPC C216 2896 3.406 5.333 3.333

22POPC H16R 2897 3.450 5.231 3.325

22POPC H16S 2898 3.485 5.407 3.306

22POPC C217 2899 3.289 5.346 3.234

22POPC H17R 2900 3.270 5.454 3.212

22POPC H17S 2901 3.198 5.309 3.285

22POPC C218 2902 3.305 5.270 3.103

22POPC H18R 2903 3.385 5.315 3.040

22POPC H18S 2904 3.209 5.273 3.048

22POPC H18T 2905 3.331 5.163 3.123

22POPC C33 2906 3.186 5.366 4.337

22POPC H3X 2907 3.095 5.365 4.402

22POPC H3Y 2908 3.245 5.275 4.363

22POPC C34 2909 3.145 5.356 4.190

22POPC H4X 2910 3.235 5.341 4.127

22POPC H4Y 2911 3.094 5.449 4.157

22POPC C35 2912 3.048 5.239 4.170

22POPC H5X 2913 2.954 5.264 4.223

22POPC H5Y 2914 3.091 5.148 4.216

22POPC C36 2915 3.018 5.211 4.023

22POPC H6X 2916 2.962 5.115 4.018

22POPC H6Y 2917 3.112 5.198 3.966

22POPC C37 2918 2.931 5.318 3.957

22POPC H7X 2919 2.993 5.408 3.936

22POPC H7Y 2920 2.850 5.349 4.026

22POPC C38 2921 2.870 5.266 3.828

22POPC H8X 2922 2.803 5.180 3.849

22POPC H8Y 2923 2.952 5.231 3.762

22POPC C39 2924 2.792 5.374 3.754

22POPC H9X 2925 2.861 5.460 3.742

22POPC H9Y 2926 2.704 5.409 3.812

22POPC C310 2927 2.750 5.326 3.615

22POPC H10X 2928 2.833 5.266 3.572

22POPC H10Y 2929 2.735 5.413 3.548

22POPC C311 2930 2.620 5.247 3.610

22POPC H11X 2931 2.536 5.311 3.645

22POPC H11Y 2932 2.628 5.160 3.678

22POPC C312 2933 2.591 5.199 3.467

22POPC H12X 2934 2.493 5.146 3.467

22POPC H12Y 2935 2.671 5.127 3.439

22POPC C313 2936 2.589 5.308 3.359

22POPC H13X 2937 2.562 5.405 3.407

22POPC H13Y 2938 2.509 5.286 3.284

22POPC C314 2939 2.723 5.321 3.284

22POPC H14X 2940 2.733 5.240 3.208

22POPC H14Y 2941 2.807 5.311 3.355

22POPC C315 2942 2.737 5.458 3.217

22POPC H15X 2943 2.723 5.533 3.298

22POPC H15Y 2944 2.655 5.470 3.143

22POPC C316 2945 2.871 5.485 3.149

22POPC H16X 2946 2.872 5.587 3.104

22POPC H16Y 2947 2.890 5.411 3.069

22POPC H16Z 2948 2.955 5.480 3.223

23POPC N 2949 4.861 4.487 4.758

23POPC C12 2950 4.853 4.456 4.906

23POPC H12A 2951 4.911 4.367 4.926

23POPC H12B 2952 4.751 4.429 4.931

23POPC C13 2953 4.756 4.588 4.724

23POPC H13A 2954 4.657 4.550 4.748

23POPC H13B 2955 4.773 4.675 4.786

23POPC H13C 2956 4.759 4.616 4.620

23POPC C14 2957 4.996 4.542 4.723

23POPC H14A 2958 5.013 4.634 4.776

23POPC H14B 2959 5.073 4.472 4.750

23POPC H14C 2960 5.004 4.562 4.617

23POPC C15 2961 4.837 4.362 4.679

23POPC H15A 2962 4.746 4.312 4.708

23POPC H15B 2963 4.916 4.290 4.696

23POPC H15C 2964 4.835 4.380 4.572

23POPC C11 2965 4.900 4.568 5.004

23POPC H11A 2966 5.011 4.571 4.997

23POPC H11B 2967 4.876 4.535 5.108

23POPC P 2968 4.928 4.825 4.992

23POPC O13 2969 5.003 4.813 5.119

23POPC O14 2970 4.843 4.944 4.969

23POPC O12 2971 4.837 4.695 4.979

23POPC O11 2972 5.025 4.806 4.868

23POPC C1 2973 5.099 4.917 4.812

23POPC HA 2974 5.203 4.883 4.792

23POPC HB 2975 5.108 5.002 4.882

23POPC C2 2976 5.040 4.968 4.677

23POPC HS 2977 4.946 5.020 4.706

23POPC O21 2978 5.004 4.855 4.593

23POPC C21 2979 4.916 4.881 4.495

23POPC O22 2980 4.876 4.992 4.468

23POPC C22 2981 4.878 4.750 4.421

23POPC H2R 2982 4.886 4.666 4.494

23POPC H2S 2983 4.953 4.731 4.341

23POPC C3 2984 5.133 5.082 4.614

23POPC HX 2985 5.141 5.160 4.693

23POPC HY 2986 5.076 5.128 4.530

23POPC O31 2987 5.268 5.043 4.571

23POPC C31 2988 5.336 5.146 4.514

23POPC O32 2989 5.290 5.260 4.512

23POPC C32 2990 5.474 5.115 4.445

23POPC H2X 2991 5.553 5.159 4.509

23POPC H2Y 2992 5.470 5.178 4.353

23POPC C23 2993 4.741 4.742 4.348

23POPC H3R 2994 4.660 4.772 4.417

23POPC H3S 2995 4.722 4.636 4.319

23POPC C24 2996 4.734 4.825 4.217

23POPC H4R 2997 4.770 4.928 4.237

23POPC H4S 2998 4.627 4.833 4.187

23POPC C25 2999 4.808 4.763 4.097

23POPC H5R 3000 4.762 4.665 4.073

23POPC H5S 3001 4.915 4.744 4.123

23POPC C26 3002 4.804 4.853 3.972

23POPC H6R 3003 4.702 4.897 3.961

23POPC H6S 3004 4.823 4.792 3.881

23POPC C27 3005 4.910 4.963 3.979

23POPC H7R 3006 5.010 4.916 3.967

23POPC H7S 3007 4.905 5.007 4.081

23POPC C28 3008 4.893 5.080 3.880

23POPC H8R 3009 4.945 5.166 3.926

23POPC H8S 3010 4.785 5.106 3.875

23POPC C29 3011 4.954 5.061 3.744

23POPC H91 3012 5.060 5.034 3.745

23POPC C210 3013 4.894 5.081 3.626

23POPC H101 3014 4.951 5.063 3.533

23POPC C211 3015 4.752 5.127 3.604

23POPC H11R 3016 4.751 5.200 3.520

23POPC H11S 3017 4.713 5.183 3.691

23POPC C212 3018 4.651 5.013 3.574

23POPC H12R 3019 4.556 5.061 3.543

23POPC H12S 3020 4.632 4.959 3.669

23POPC C213 3021 4.691 4.909 3.468

23POPC H13R 3022 4.605 4.843 3.445

23POPC H13S 3023 4.767 4.841 3.512

23POPC C214 3024 4.742 4.964 3.336

23POPC H14R 3025 4.823 5.039 3.352

23POPC H14S 3026 4.657 5.016 3.287

23POPC C215 3027 4.794 4.855 3.243

23POPC H15R 3028 4.739 4.762 3.267

23POPC H15S 3029 4.903 4.839 3.261

23POPC C216 3030 4.772 4.886 3.095

23POPC H16R 3031 4.742 4.993 3.093

23POPC H16S 3032 4.688 4.825 3.054

23POPC C217 3033 4.892 4.881 3.002

23POPC H17R 3034 4.926 4.776 2.985

23POPC H17S 3035 4.976 4.937 3.047

23POPC C218 3036 4.853 4.946 2.869

23POPC H18R 3037 4.940 4.994 2.820

23POPC H18S 3038 4.779 5.028 2.887

23POPC H18T 3039 4.806 4.873 2.800

23POPC C33 3040 5.518 4.971 4.399

23POPC H3X 3041 5.426 4.912 4.379

23POPC H3Y 3042 5.573 4.917 4.479

23POPC C34 3043 5.601 4.963 4.269

23POPC H4X 3044 5.708 4.969 4.297

23POPC H4Y 3045 5.579 5.053 4.207

23POPC C35 3046 5.586 4.838 4.177

23POPC H5X 3047 5.656 4.759 4.213

23POPC H5Y 3048 5.620 4.866 4.075

23POPC C36 3049 5.446 4.775 4.163

23POPC H6X 3050 5.373 4.855 4.139

23POPC H6Y 3051 5.421 4.729 4.261

23POPC C37 3052 5.430 4.665 4.056

23POPC H7X 3053 5.469 4.572 4.103

23POPC H7Y 3054 5.493 4.687 3.967

23POPC C38 3055 5.286 4.631 4.011

23POPC H8X 3056 5.210 4.668 4.083

23POPC H8Y 3057 5.273 4.521 4.004

23POPC C39 3058 5.253 4.690 3.874

23POPC H9X 3059 5.341 4.684 3.807

23POPC H9Y 3060 5.231 4.798 3.893

23POPC C310 3061 5.132 4.634 3.802

23POPC H10X 3062 5.045 4.627 3.872

23POPC H10Y 3063 5.152 4.532 3.762

23POPC C311 3064 5.100 4.732 3.690

23POPC H11X 3065 5.182 4.734 3.615

23POPC H11Y 3066 5.099 4.833 3.737

23POPC C312 3067 4.964 4.715 3.625

23POPC H12X 3068 4.946 4.812 3.574

23POPC H12Y 3069 4.885 4.702 3.702

23POPC C313 3070 4.953 4.605 3.520

23POPC H13X 3071 4.847 4.605 3.486

23POPC H13Y 3072 4.975 4.506 3.565

23POPC C314 3073 5.046 4.629 3.400

23POPC H14X 3074 5.152 4.616 3.433

23POPC H14Y 3075 5.037 4.735 3.368

23POPC C315 3076 5.019 4.536 3.282

23POPC H15X 3077 5.037 4.432 3.317

23POPC H15Y 3078 5.095 4.558 3.203

23POPC C316 3079 4.879 4.544 3.220

23POPC H16X 3080 4.874 4.629 3.149

23POPC H16Y 3081 4.800 4.557 3.297

23POPC H16Z 3082 4.853 4.452 3.164

24POPC N 3083 0.817 3.343 4.930

24POPC C12 3084 0.899 3.297 5.050

24POPC H12A 3085 0.834 3.295 5.136

24POPC H12B 3086 0.978 3.368 5.068

24POPC C13 3087 0.904 3.364 4.810

24POPC H13A 3088 0.847 3.412 4.731

24POPC H13B 3089 0.988 3.428 4.831

24POPC H13C 3090 0.939 3.270 4.771

24POPC C14 3091 0.714 3.240 4.898

24POPC H14A 3092 0.647 3.221 4.981

24POPC H14B 3093 0.657 3.265 4.809

24POPC H14C 3094 0.762 3.146 4.877

24POPC C15 3095 0.748 3.472 4.960

24POPC H15A 3096 0.679 3.460 5.042

24POPC H15B 3097 0.693 3.506 4.873

24POPC H15C 3098 0.819 3.550 4.981

24POPC C11 3099 0.961 3.156 5.041

24POPC H11A 3100 1.039 3.145 5.120

24POPC H11B 3101 1.011 3.147 4.943

24POPC P 3102 0.860 2.918 4.996

24POPC O13 3103 0.723 2.878 4.961

24POPC O14 3104 0.938 2.827 5.083

24POPC O12 3105 0.858 3.060 5.064

24POPC O11 3106 0.945 2.947 4.867

24POPC C1 3107 0.885 2.963 4.739

24POPC HA 3108 0.802 3.037 4.738

24POPC HB 3109 0.841 2.865 4.708

24POPC C2 3110 0.990 3.003 4.633

24POPC HS 3111 1.086 2.954 4.663

24POPC O21 3112 1.009 3.147 4.624

24POPC C21 3113 1.115 3.178 4.547

24POPC O22 3114 1.192 3.097 4.500

24POPC C22 3115 1.132 3.328 4.537

24POPC H2R 3116 1.195 3.359 4.623

24POPC H2S 3117 1.034 3.380 4.545

24POPC C3 3118 0.951 2.937 4.500

24POPC HX 3119 0.964 2.829 4.525

24POPC HY 3120 1.024 2.960 4.419

24POPC O31 3121 0.813 2.956 4.466

24POPC C31 3122 0.792 2.988 4.340

24POPC O32 3123 0.872 3.037 4.263

24POPC C32 3124 0.657 2.931 4.300

24POPC H2X 3125 0.594 2.915 4.390

24POPC H2Y 3126 0.678 2.831 4.255

24POPC C23 3127 1.205 3.369 4.407

24POPC H3R 3128 1.284 3.296 4.380

24POPC H3S 3129 1.254 3.467 4.425

24POPC C24 3130 1.114 3.385 4.284

24POPC H4R 3131 1.152 3.469 4.222

24POPC H4S 3132 1.014 3.414 4.321

24POPC C25 3133 1.104 3.261 4.194

24POPC H5R 3134 1.030 3.281 4.114

24POPC H5S 3135 1.073 3.172 4.253

24POPC C26 3136 1.228 3.232 4.112

24POPC H6R 3137 1.310 3.193 4.175

24POPC H6S 3138 1.255 3.331 4.070

24POPC C27 3139 1.201 3.140 3.992

24POPC H7R 3140 1.300 3.111 3.950

24POPC H7S 3141 1.148 3.198 3.913

24POPC C28 3142 1.116 3.017 4.027

24POPC H8R 3143 1.008 3.043 4.027

24POPC H8S 3144 1.142 2.986 4.131

24POPC C29 3145 1.148 2.905 3.933

24POPC H91 3146 1.201 2.821 3.980

24POPC C210 3147 1.125 2.901 3.801

24POPC H101 3148 1.159 2.813 3.744

24POPC C211 3149 1.040 2.995 3.722

24POPC H11R 3150 1.088 3.025 3.626

24POPC H11S 3151 1.014 3.087 3.778

24POPC C212 3152 0.913 2.915 3.697

24POPC H12R 3153 0.837 2.982 3.651

24POPC H12S 3154 0.870 2.879 3.793

24POPC C213 3155 0.929 2.795 3.604

24POPC H13R 3156 0.932 2.700 3.662

24POPC H13S 3157 1.026 2.804 3.549

24POPC C214 3158 0.817 2.790 3.501

24POPC H14R 3159 0.813 2.888 3.448

24POPC H14S 3160 0.720 2.775 3.553

24POPC C215 3161 0.839 2.680 3.399

24POPC H15R 3162 0.807 2.582 3.441

24POPC H15S 3163 0.949 2.671 3.383

24POPC C216 3164 0.774 2.709 3.264

24POPC H16R 3165 0.812 2.634 3.192

24POPC H16S 3166 0.805 2.810 3.228

24POPC C217 3167 0.622 2.698 3.257

24POPC H17R 3168 0.576 2.784 3.309

24POPC H17S 3169 0.587 2.605 3.307

24POPC C218 3170 0.580 2.693 3.110

24POPC H18R 3171 0.625 2.604 3.060

24POPC H18S 3172 0.615 2.784 3.057

24POPC H18T 3173 0.470 2.685 3.099

24POPC C33 3174 0.587 3.020 4.197

24POPC H3X 3175 0.660 3.074 4.134

24POPC H3Y 3176 0.528 3.098 4.249

24POPC C34 3177 0.497 2.935 4.108

24POPC H4X 3178 0.421 2.998 4.059

24POPC H4Y 3179 0.444 2.859 4.169

24POPC C35 3180 0.571 2.867 3.993

24POPC H5X 3181 0.496 2.816 3.929

24POPC H5Y 3182 0.639 2.787 4.031

24POPC C36 3183 0.648 2.963 3.902

24POPC H6X 3184 0.665 2.907 3.807

24POPC H6Y 3185 0.746 2.986 3.947

24POPC C37 3186 0.579 3.095 3.864

24POPC H7X 3187 0.583 3.167 3.949

24POPC H7Y 3188 0.473 3.079 3.835

24POPC C38 3189 0.658 3.151 3.746

24POPC H8X 3190 0.642 3.082 3.660

24POPC H8Y 3191 0.765 3.146 3.775

24POPC C39 3192 0.632 3.294 3.699

24POPC H9X 3193 0.620 3.361 3.787

24POPC H9Y 3194 0.538 3.298 3.640

24POPC C310 3195 0.750 3.340 3.614

24POPC H10X 3196 0.842 3.343 3.676

24POPC H10Y 3197 0.732 3.442 3.575

24POPC C311 3198 0.773 3.243 3.497

24POPC H11X 3199 0.679 3.236 3.439

24POPC H11Y 3200 0.798 3.140 3.532

24POPC C312 3201 0.887 3.284 3.405

24POPC H12X 3202 0.985 3.266 3.453

24POPC H12Y 3203 0.878 3.393 3.388

24POPC C313 3204 0.879 3.210 3.271

24POPC H13X 3205 0.773 3.209 3.236

24POPC H13Y 3206 0.912 3.105 3.282

24POPC C314 3207 0.965 3.287 3.174

24POPC H14X 3208 1.070 3.267 3.206

24POPC H14Y 3209 0.943 3.394 3.188

24POPC C315 3210 0.952 3.257 3.025

24POPC H15X 3211 0.946 3.354 2.971

24POPC H15Y 3212 0.857 3.202 3.003

24POPC C316 3213 1.072 3.182 2.971

24POPC H16X 3214 1.167 3.227 3.005

24POPC H16Y 3215 1.071 3.181 2.860

24POPC H16Z 3216 1.070 3.077 3.008

25POPC N 3217 4.249 5.361 5.097

25POPC C12 3218 4.187 5.410 4.966

25POPC H12A 3219 4.226 5.508 4.941

25POPC H12B 3220 4.081 5.423 4.979

25POPC C13 3221 4.181 5.236 5.142

25POPC H13A 3222 4.233 5.190 5.225

25POPC H13B 3223 4.079 5.255 5.172

25POPC H13C 3224 4.178 5.167 5.059

25POPC C14 3225 4.394 5.330 5.075

25POPC H14A 3226 4.451 5.419 5.052

25POPC H14B 3227 4.437 5.281 5.161

25POPC H14C 3228 4.405 5.266 4.990

25POPC C15 3229 4.237 5.463 5.205

25POPC H15A 3230 4.133 5.483 5.228

25POPC H15B 3231 4.284 5.556 5.176

25POPC H15C 3232 4.283 5.427 5.296

25POPC C11 3233 4.203 5.318 4.842

25POPC H11A 3234 4.310 5.316 4.812

25POPC H11B 3235 4.146 5.363 4.757

25POPC P 3236 4.191 5.060 4.787

25POPC O13 3237 4.108 5.051 4.665

25POPC O14 3238 4.185 4.946 4.882

25POPC O12 3239 4.151 5.189 4.868

25POPC O11 3240 4.340 5.089 4.747

25POPC C1 3241 4.446 5.045 4.832

25POPC HA 3242 4.433 4.940 4.865

25POPC HB 3243 4.452 5.110 4.922

25POPC C2 3244 4.584 5.052 4.761

25POPC HS 3245 4.663 5.051 4.838

25POPC O21 3246 4.599 5.167 4.678

25POPC C21 3247 4.571 5.285 4.720

25POPC O22 3248 4.566 5.323 4.837

25POPC C22 3249 4.558 5.377 4.598

25POPC H2R 3250 4.648 5.355 4.536

25POPC H2S 3251 4.563 5.482 4.633

25POPC C3 3252 4.607 4.927 4.671

25POPC HX 3253 4.606 4.838 4.738

25POPC HY 3254 4.709 4.935 4.629

25POPC O31 3255 4.507 4.907 4.568

25POPC C31 3256 4.519 4.984 4.459

25POPC O32 3257 4.607 5.065 4.437

25POPC C32 3258 4.412 4.949 4.358

25POPC H2X 3259 4.312 4.956 4.408

25POPC H2Y 3260 4.429 4.844 4.325

25POPC C23 3261 4.432 5.355 4.509

25POPC H3R 3262 4.341 5.386 4.565

25POPC H3S 3263 4.422 5.247 4.484

25POPC C24 3264 4.443 5.434 4.377

25POPC H4R 3265 4.527 5.390 4.318

25POPC H4S 3266 4.470 5.539 4.403

25POPC C25 3267 4.320 5.439 4.286

25POPC H5R 3268 4.230 5.461 4.348

25POPC H5S 3269 4.304 5.341 4.237

25POPC C26 3270 4.330 5.549 4.178

25POPC H6R 3271 4.353 5.644 4.231

25POPC H6S 3272 4.233 5.561 4.126

25POPC C27 3273 4.442 5.530 4.074

25POPC H7R 3274 4.427 5.436 4.017

25POPC H7S 3275 4.538 5.518 4.129

25POPC C28 3276 4.460 5.652 3.981

25POPC H8R 3277 4.566 5.654 3.946

25POPC H8S 3278 4.446 5.746 4.039

25POPC C29 3279 4.368 5.648 3.862

25POPC H91 3280 4.284 5.720 3.864

25POPC C210 3281 4.382 5.564 3.758

25POPC H101 3282 4.310 5.567 3.675

25POPC C211 3283 4.496 5.466 3.740

25POPC H11R 3284 4.465 5.363 3.768

25POPC H11S 3285 4.583 5.493 3.804

25POPC C212 3286 4.545 5.465 3.595

25POPC H12R 3287 4.650 5.430 3.590

25POPC H12S 3288 4.544 5.569 3.554

25POPC C213 3289 4.463 5.372 3.506

25POPC H13R 3290 4.357 5.377 3.537

25POPC H13S 3291 4.497 5.267 3.519

25POPC C214 3292 4.468 5.413 3.359

25POPC H14R 3293 4.428 5.517 3.356

25POPC H14S 3294 4.397 5.348 3.304

25POPC C215 3295 4.606 5.419 3.290

25POPC H15R 3296 4.671 5.486 3.350

25POPC H15S 3297 4.591 5.468 3.191

25POPC C216 3298 4.679 5.286 3.262

25POPC H16R 3299 4.640 5.249 3.165

25POPC H16S 3300 4.651 5.214 3.342

25POPC C217 3301 4.832 5.293 3.249

25POPC H17R 3302 4.880 5.263 3.344

25POPC H17S 3303 4.860 5.399 3.229

25POPC C218 3304 4.895 5.211 3.137

25POPC H18R 3305 4.818 5.182 3.063

25POPC H18S 3306 4.942 5.118 3.177

25POPC H18T 3307 4.974 5.269 3.085

25POPC C33 3308 4.420 5.044 4.237

25POPC H3X 3309 4.524 5.048 4.198

25POPC H3Y 3310 4.394 5.147 4.269

25POPC C34 3311 4.328 5.002 4.122

25POPC H4X 3312 4.223 4.999 4.158

25POPC H4Y 3313 4.357 4.900 4.089

25POPC C35 3314 4.338 5.098 4.003

25POPC H5X 3315 4.440 5.094 3.959

25POPC H5Y 3316 4.321 5.202 4.038

25POPC C36 3317 4.234 5.067 3.894

25POPC H6X 3318 4.244 5.140 3.811

25POPC H6Y 3319 4.132 5.080 3.937

25POPC C37 3320 4.245 4.925 3.836

25POPC H7X 3321 4.159 4.910 3.767

25POPC H7Y 3322 4.235 4.849 3.916

25POPC C38 3323 4.377 4.902 3.761

25POPC H8X 3324 4.458 4.891 3.836

25POPC H8Y 3325 4.400 4.993 3.700

25POPC C39 3326 4.377 4.779 3.669

25POPC H9X 3327 4.307 4.701 3.705

25POPC H9Y 3328 4.479 4.736 3.672

25POPC C310 3329 4.348 4.813 3.522

25POPC H10X 3330 4.375 4.727 3.457

25POPC H10Y 3331 4.416 4.897 3.494

25POPC C311 3332 4.207 4.860 3.486

25POPC H11X 3333 4.164 4.923 3.567

25POPC H11Y 3334 4.140 4.773 3.470

25POPC C312 3335 4.215 4.948 3.361

25POPC H12X 3336 4.266 4.888 3.282

25POPC H12Y 3337 4.280 5.035 3.386

25POPC C313 3338 4.082 5.002 3.309

25POPC H13X 3339 4.058 5.101 3.353

25POPC H13Y 3340 4.006 4.930 3.346

25POPC C314 3341 4.070 5.004 3.155

25POPC H14X 3342 3.971 4.964 3.126

25POPC H14Y 3343 4.147 4.936 3.111

25POPC C315 3344 4.077 5.140 3.086

25POPC H15X 3345 4.164 5.200 3.121

25POPC H15Y 3346 3.987 5.200 3.112

25POPC C316 3347 4.087 5.122 2.935

25POPC H16X 3348 4.131 5.212 2.887

25POPC H16Y 3349 3.986 5.103 2.891

25POPC H16Z 3350 4.152 5.036 2.908

26POPC N 3351 4.912 2.139 4.919

26POPC C12 3352 5.006 2.021 4.898

26POPC H12A 3353 4.962 1.953 4.827

26POPC H12B 3354 5.017 1.968 4.991

26POPC C13 3355 4.892 2.211 4.789

26POPC H13A 3356 4.849 2.145 4.715

26POPC H13B 3357 4.830 2.298 4.801

26POPC H13C 3358 4.988 2.244 4.752

26POPC C14 3359 4.780 2.091 4.971

26POPC H14A 3360 4.733 2.021 4.903

26POPC H14B 3361 4.793 2.040 5.066

26POPC H14C 3362 4.713 2.174 4.989

26POPC C15 3363 4.972 2.235 5.017

26POPC H15A 3364 4.983 2.190 5.115

26POPC H15B 3365 4.914 2.325 5.028

26POPC H15C 3366 5.072 2.261 4.986

26POPC C11 3367 5.150 2.053 4.850

26POPC H11A 3368 5.146 2.121 4.762

26POPC H11B 3369 5.198 1.959 4.818

26POPC P 3370 5.362 2.183 4.933

26POPC O13 3371 5.453 2.091 4.861

26POPC O14 3372 5.405 2.235 5.064

26POPC O12 3373 5.224 2.108 4.958

26POPC O11 3374 5.316 2.300 4.836

26POPC C1 3375 5.357 2.295 4.699

26POPC HA 3376 5.338 2.195 4.653

26POPC HB 3377 5.466 2.315 4.691

26POPC C2 3378 5.284 2.400 4.612

26POPC HS 3379 5.296 2.498 4.665

26POPC O21 3380 5.143 2.368 4.602

26POPC C21 3381 5.072 2.466 4.547

26POPC O22 3382 5.109 2.582 4.534

26POPC C22 3383 4.935 2.413 4.506

26POPC H2R 3384 4.859 2.478 4.554

26POPC H2S 3385 4.924 2.309 4.542

26POPC C3 3386 5.351 2.411 4.472

26POPC HX 3387 5.459 2.428 4.489

26POPC HY 3388 5.312 2.499 4.416

26POPC O31 3389 5.324 2.291 4.397

26POPC C31 3390 5.416 2.256 4.309

26POPC O32 3391 5.523 2.311 4.292

26POPC C32 3392 5.361 2.139 4.227

26POPC H2X 3393 5.253 2.158 4.209

26POPC H2Y 3394 5.371 2.046 4.288

26POPC C23 3395 4.915 2.414 4.353

26POPC H3R 3396 4.941 2.513 4.310

26POPC H3S 3397 4.807 2.395 4.333

26POPC C24 3398 4.997 2.304 4.284

26POPC H4R 3399 4.970 2.204 4.326

26POPC H4S 3400 5.105 2.321 4.305

26POPC C25 3401 4.980 2.300 4.131

26POPC H5R 3402 5.049 2.223 4.091

26POPC H5S 3403 5.010 2.398 4.089

26POPC C26 3404 4.838 2.266 4.086

26POPC H6R 3405 4.768 2.340 4.130

26POPC H6S 3406 4.808 2.165 4.123

26POPC C27 3407 4.820 2.273 3.933

26POPC H7R 3408 4.874 2.361 3.892

26POPC H7S 3409 4.712 2.289 3.917

26POPC C28 3410 4.863 2.146 3.857

26POPC H8R 3411 4.834 2.056 3.916

26POPC H8S 3412 4.973 2.140 3.848

26POPC C29 3413 4.796 2.141 3.722

26POPC H91 3414 4.715 2.213 3.706

26POPC C210 3415 4.826 2.055 3.625

26POPC H101 3416 4.770 2.059 3.530

26POPC C211 3417 4.936 1.952 3.631

26POPC H11R 3418 4.906 1.864 3.692

26POPC H11S 3419 5.028 1.995 3.676

26POPC C212 3420 4.971 1.906 3.489

26POPC H12R 3421 4.887 1.849 3.444

26POPC H12S 3422 5.057 1.836 3.497

26POPC C213 3423 5.007 2.023 3.396

26POPC H13R 3424 5.062 2.101 3.453

26POPC H13S 3425 4.914 2.070 3.358

26POPC C214 3426 5.093 1.979 3.278

26POPC H14R 3427 5.087 2.056 3.197

26POPC H14S 3428 5.051 1.885 3.237

26POPC C215 3429 5.239 1.957 3.319

26POPC H15R 3430 5.288 1.882 3.254

26POPC H15S 3431 5.236 1.912 3.420

26POPC C216 3432 5.327 2.083 3.330

26POPC H16R 3433 5.430 2.056 3.362

26POPC H16S 3434 5.284 2.144 3.412

26POPC C217 3435 5.341 2.174 3.207

26POPC H17R 3436 5.379 2.269 3.251

26POPC H17S 3437 5.243 2.196 3.160

26POPC C218 3438 5.441 2.137 3.099

26POPC H18R 3439 5.531 2.090 3.144

26POPC H18S 3440 5.470 2.229 3.043

26POPC H18T 3441 5.402 2.070 3.020

26POPC C33 3442 5.434 2.123 4.092

26POPC H3X 3443 5.535 2.082 4.113

26POPC H3Y 3444 5.450 2.221 4.044

26POPC C34 3445 5.361 2.029 3.994

26POPC H4X 3446 5.338 1.934 4.045

26POPC H4Y 3447 5.431 2.003 3.912

26POPC C35 3448 5.232 2.085 3.929

26POPC H5X 3449 5.151 2.069 4.003

26POPC H5Y 3450 5.206 2.022 3.841

26POPC C36 3451 5.231 2.233 3.887

26POPC H6X 3452 5.272 2.299 3.967

26POPC H6Y 3453 5.125 2.265 3.877

26POPC C37 3454 5.297 2.264 3.752

26POPC H7X 3455 5.278 2.182 3.680

26POPC H7Y 3456 5.407 2.272 3.768

26POPC C38 3457 5.248 2.396 3.690

26POPC H8X 3458 5.319 2.428 3.611

26POPC H8Y 3459 5.249 2.475 3.769

26POPC C39 3460 5.106 2.388 3.628

26POPC H9X 3461 5.045 2.470 3.671

26POPC H9Y 3462 5.058 2.292 3.657

26POPC C310 3463 5.105 2.403 3.475

26POPC H10X 3464 5.160 2.318 3.429

26POPC H10Y 3465 5.157 2.497 3.448

26POPC C311 3466 4.962 2.408 3.418

26POPC H11X 3467 4.910 2.490 3.473

26POPC H11Y 3468 4.908 2.313 3.440

26POPC C312 3469 4.952 2.443 3.269

26POPC H12X 3470 5.022 2.526 3.246

26POPC H12Y 3471 4.849 2.478 3.245

26POPC C313 3472 4.981 2.323 3.178

26POPC H13X 3473 4.902 2.246 3.188

26POPC H13Y 3474 5.075 2.278 3.215

26POPC C314 3475 4.997 2.363 3.030

26POPC H14X 3476 5.100 2.335 2.999

26POPC H14Y 3477 4.987 2.473 3.020

26POPC C315 3478 4.899 2.293 2.935

26POPC H15X 3479 4.797 2.295 2.980

26POPC H15Y 3480 4.933 2.188 2.929

26POPC C316 3481 4.894 2.349 2.793

26POPC H16X 3482 4.844 2.277 2.724

26POPC H16Y 3483 4.996 2.369 2.754

26POPC H16Z 3484 4.835 2.444 2.792

27POPC N 3485 1.458 4.973 5.014

27POPC C12 3486 1.423 4.832 5.059

27POPC H12A 3487 1.362 4.840 5.148

27POPC H12B 3488 1.514 4.780 5.083

27POPC C13 3489 1.335 5.051 4.981

27POPC H13A 3490 1.272 4.989 4.919

27POPC H13B 3491 1.280 5.076 5.071

27POPC H13C 3492 1.359 5.142 4.929

27POPC C14 3493 1.535 5.046 5.120

27POPC H14A 3494 1.624 4.991 5.147

27POPC H14B 3495 1.569 5.141 5.083

27POPC H14C 3496 1.475 5.063 5.208

27POPC C15 3497 1.544 4.966 4.891

27POPC H15A 3498 1.640 4.924 4.913

27POPC H15B 3499 1.566 5.064 4.852

27POPC H15C 3500 1.498 4.907 4.813

27POPC C11 3501 1.344 4.743 4.959

27POPC H11A 3502 1.336 4.641 5.003

27POPC H11B 3503 1.405 4.733 4.867

27POPC P 3504 1.132 4.738 4.811

27POPC O13 3505 1.065 4.613 4.850

27POPC O14 3506 1.220 4.736 4.692

27POPC O12 3507 1.214 4.795 4.934

27POPC O11 3508 1.022 4.851 4.787

27POPC C1 3509 1.069 4.987 4.794

27POPC HA 3510 1.174 4.994 4.758

27POPC HB 3511 1.066 5.023 4.899

27POPC C2 3512 0.991 5.090 4.706

27POPC HS 3513 0.901 5.120 4.765

27POPC O21 3514 0.949 5.027 4.584

27POPC C21 3515 0.864 5.102 4.514

27POPC O22 3516 0.812 5.205 4.553

27POPC C22 3517 0.852 5.042 4.375

27POPC H2R 3518 0.813 4.938 4.385

27POPC H2S 3519 0.956 5.035 4.337

27POPC C3 3520 1.080 5.216 4.678

27POPC HX 3521 1.093 5.268 4.775

27POPC HY 3522 1.031 5.289 4.610

27POPC O31 3523 1.211 5.177 4.631

27POPC C31 3524 1.219 5.150 4.500

27POPC O32 3525 1.138 5.181 4.416

27POPC C32 3526 1.350 5.072 4.476

27POPC H2X 3527 1.326 4.968 4.507

27POPC H2Y 3528 1.427 5.113 4.545

27POPC C23 3529 0.766 5.123 4.277

27POPC H3R 3530 0.807 5.226 4.270

27POPC H3S 3531 0.662 5.132 4.316

27POPC C24 3532 0.763 5.059 4.138

27POPC H4R 3533 0.705 5.125 4.070

27POPC H4S 3534 0.708 4.963 4.144

27POPC C25 3535 0.902 5.030 4.078

27POPC H5R 3536 0.962 4.968 4.148

27POPC H5S 3537 0.957 5.126 4.066

27POPC C26 3538 0.899 4.951 3.947

27POPC H6R 3539 0.860 4.849 3.968

27POPC H6S 3540 1.004 4.939 3.910

27POPC C27 3541 0.814 5.013 3.836

27POPC H7R 3542 0.878 5.027 3.746

27POPC H7S 3543 0.776 5.112 3.868

27POPC C28 3544 0.691 4.931 3.801

27POPC H8R 3545 0.621 4.934 3.888

27POPC H8S 3546 0.720 4.825 3.788

27POPC C29 3547 0.625 4.988 3.677

27POPC H91 3548 0.547 5.064 3.695

27POPC C210 3549 0.651 4.948 3.552

27POPC H101 3550 0.597 4.994 3.468

27POPC C211 3551 0.756 4.846 3.514

27POPC H11R 3552 0.711 4.761 3.460

27POPC H11S 3553 0.807 4.803 3.603

27POPC C212 3554 0.865 4.913 3.429

27POPC H12R 3555 0.954 4.846 3.426

27POPC H12S 3556 0.898 5.007 3.476

27POPC C213 3557 0.818 4.941 3.286

27POPC H13R 3558 0.893 5.004 3.235

27POPC H13S 3559 0.722 4.997 3.287

27POPC C214 3560 0.801 4.814 3.201

27POPC H14R 3561 0.762 4.846 3.102

27POPC H14S 3562 0.725 4.746 3.245

27POPC C215 3563 0.933 4.737 3.185

27POPC H15R 3564 0.942 4.664 3.268

27POPC H15S 3565 1.019 4.807 3.193

27POPC C216 3566 0.941 4.667 3.050

27POPC H16R 3567 0.849 4.606 3.034

27POPC H16S 3568 1.028 4.598 3.050

27POPC C217 3569 0.955 4.764 2.933

27POPC H17R 3570 0.922 4.866 2.962

27POPC H17S 3571 0.880 4.736 2.855

27POPC C218 3572 1.097 4.765 2.876

27POPC H18R 3573 1.135 4.662 2.862

27POPC H18S 3574 1.168 4.817 2.944

27POPC H18T 3575 1.097 4.814 2.777

27POPC C33 3576 1.409 5.067 4.333

27POPC H3X 3577 1.503 5.008 4.337

27POPC H3Y 3578 1.440 5.166 4.294

27POPC C34 3579 1.316 5.006 4.228

27POPC H4X 3580 1.301 5.077 4.143

27POPC H4Y 3581 1.216 4.985 4.272

27POPC C35 3582 1.375 4.879 4.170

27POPC H5X 3583 1.390 4.808 4.254

27POPC H5Y 3584 1.475 4.899 4.126

27POPC C36 3585 1.280 4.819 4.066

27POPC H6X 3586 1.175 4.839 4.095

27POPC H6Y 3587 1.293 4.709 4.065

27POPC C37 3588 1.308 4.871 3.925

27POPC H7X 3589 1.411 4.913 3.920

27POPC H7Y 3590 1.238 4.953 3.900

27POPC C38 3591 1.300 4.757 3.823

27POPC H8X 3592 1.196 4.716 3.820

27POPC H8Y 3593 1.367 4.675 3.857

27POPC C39 3594 1.344 4.799 3.684

27POPC H9X 3595 1.354 4.709 3.619

27POPC H9Y 3596 1.444 4.846 3.693

27POPC C310 3597 1.245 4.896 3.618

27POPC H10X 3598 1.236 4.988 3.680

27POPC H10Y 3599 1.145 4.849 3.613

27POPC C311 3600 1.289 4.937 3.477

27POPC H11X 3601 1.211 5.006 3.438

27POPC H11Y 3602 1.291 4.847 3.411

27POPC C312 3603 1.428 5.004 3.475

27POPC H12X 3604 1.505 4.933 3.510

27POPC H12Y 3605 1.427 5.090 3.546

27POPC C313 3606 1.472 5.054 3.337

27POPC H13X 3607 1.486 4.966 3.270

27POPC H13Y 3608 1.569 5.107 3.345

27POPC C314 3609 1.369 5.146 3.272

27POPC H14X 3610 1.276 5.086 3.260

27POPC H14Y 3611 1.402 5.174 3.169

27POPC C315 3612 1.331 5.271 3.351

27POPC H15X 3613 1.421 5.336 3.363

27POPC H15Y 3614 1.296 5.243 3.453

27POPC C316 3615 1.220 5.348 3.281

27POPC H16X 3616 1.248 5.367 3.175

27POPC H16Y 3617 1.203 5.446 3.330

27POPC H16Z 3618 1.125 5.291 3.283

28POPC N 3619 4.166 0.831 4.987

28POPC C12 3620 4.201 0.905 4.858

28POPC H12A 3621 4.113 0.912 4.796

28POPC H12B 3622 4.274 0.848 4.803

28POPC C13 3623 4.146 0.685 4.963

28POPC H13A 3624 4.114 0.637 5.054

28POPC H13B 3625 4.072 0.670 4.887

28POPC H13C 3626 4.238 0.638 4.932

28POPC C14 3627 4.271 0.853 5.091

28POPC H14A 3628 4.246 0.805 5.185

28POPC H14B 3629 4.369 0.818 5.061

28POPC H14C 3630 4.281 0.959 5.111

28POPC C15 3631 4.038 0.887 5.042

28POPC H15A 3632 4.010 0.842 5.137

28POPC H15B 3633 3.955 0.876 4.974

28POPC H15C 3634 4.047 0.993 5.057

28POPC C11 3635 4.255 1.051 4.867

28POPC H11A 3636 4.281 1.087 4.765

28POPC H11B 3637 4.350 1.052 4.925

28POPC P 3638 4.029 1.180 4.854

28POPC O13 3639 4.033 1.322 4.816

28POPC O14 3640 3.920 1.139 4.945

28POPC O12 3641 4.162 1.139 4.929

28POPC O11 3642 4.028 1.088 4.727

28POPC C1 3643 3.907 1.056 4.658

28POPC HA 3644 3.909 1.101 4.556

28POPC HB 3645 3.815 1.093 4.709

28POPC C2 3646 3.895 0.902 4.644

28POPC HS 3647 3.903 0.860 4.746

28POPC O21 3648 4.005 0.859 4.560

28POPC C21 3649 4.058 0.742 4.588

28POPC O22 3650 4.025 0.668 4.680

28POPC C22 3651 4.185 0.722 4.505

28POPC H2R 3652 4.227 0.623 4.530

28POPC H2S 3653 4.257 0.801 4.538

28POPC C3 3654 3.757 0.860 4.586

28POPC HX 3655 3.680 0.888 4.660

28POPC HY 3656 3.752 0.749 4.575

28POPC O31 3657 3.727 0.929 4.463

28POPC C31 3658 3.779 0.872 4.355

28POPC O32 3659 3.839 0.766 4.354

28POPC C32 3660 3.751 0.960 4.234

28POPC H2X 3661 3.794 1.060 4.253

28POPC H2Y 3662 3.641 0.969 4.220

28POPC C23 3663 4.171 0.734 4.351

28POPC H3R 3664 4.120 0.830 4.326

28POPC H3S 3665 4.108 0.652 4.311

28POPC C24 3666 4.309 0.732 4.283

28POPC H4R 3667 4.361 0.636 4.306

28POPC H4S 3668 4.371 0.813 4.326

28POPC C25 3669 4.308 0.751 4.131

28POPC H5R 3670 4.258 0.666 4.080

28POPC H5S 3671 4.414 0.753 4.096

28POPC C26 3672 4.241 0.882 4.090

28POPC H6R 3673 4.265 0.959 4.166

28POPC H6S 3674 4.131 0.868 4.089

28POPC C27 3675 4.287 0.937 3.954

28POPC H7R 3676 4.248 0.874 3.871

28POPC H7S 3677 4.398 0.931 3.950

28POPC C28 3678 4.247 1.084 3.935

28POPC H8R 3679 4.301 1.121 3.845

28POPC H8S 3680 4.286 1.142 4.021

28POPC C29 3681 4.098 1.104 3.925

28POPC H91 3682 4.040 1.066 4.010

28POPC C210 3683 4.033 1.162 3.822

28POPC H101 3684 3.924 1.168 3.822

28POPC C211 3685 4.102 1.217 3.700

28POPC H11R 3686 4.194 1.273 3.728

28POPC H11S 3687 4.037 1.288 3.646

28POPC C212 3688 4.142 1.103 3.605

28POPC H12R 3689 4.050 1.063 3.558

28POPC H12S 3690 4.190 1.019 3.658

28POPC C213 3691 4.238 1.150 3.495

28POPC H13R 3692 4.344 1.142 3.530

28POPC H13S 3693 4.219 1.257 3.470

28POPC C214 3694 4.217 1.067 3.370

28POPC H14R 3695 4.111 1.084 3.339

28POPC H14S 3696 4.227 0.959 3.396

28POPC C215 3697 4.306 1.099 3.252

28POPC H15R 3698 4.350 1.200 3.262

28POPC H15S 3699 4.244 1.099 3.160

28POPC C216 3700 4.415 0.992 3.235

28POPC H16R 3701 4.368 0.892 3.226

28POPC H16S 3702 4.481 0.990 3.325

28POPC C217 3703 4.498 1.019 3.110

28POPC H17R 3704 4.433 1.062 3.031

28POPC H17S 3705 4.538 0.923 3.071

28POPC C218 3706 4.613 1.113 3.142

28POPC H18R 3707 4.696 1.057 3.189

28POPC H18S 3708 4.580 1.192 3.213

28POPC H18T 3709 4.650 1.163 3.050

28POPC C33 3710 3.816 0.899 4.108

28POPC H3X 3711 3.922 0.873 4.129

28POPC H3Y 3712 3.815 0.974 4.026

28POPC C34 3713 3.742 0.774 4.060

28POPC H4X 3714 3.656 0.807 3.997

28POPC H4Y 3715 3.703 0.713 4.144

28POPC C35 3716 3.831 0.685 3.973

28POPC H5X 3717 3.771 0.598 3.938

28POPC H5Y 3718 3.916 0.646 4.032

28POPC C36 3719 3.882 0.765 3.854

28POPC H6X 3720 3.960 0.837 3.885

28POPC H6Y 3721 3.797 0.822 3.810

28POPC C37 3722 3.937 0.673 3.747

28POPC H7X 3723 3.862 0.595 3.724

28POPC H7Y 3724 4.029 0.623 3.785

28POPC C38 3725 3.971 0.749 3.620

28POPC H8X 3726 4.060 0.814 3.638

28POPC H8Y 3727 3.886 0.814 3.589

28POPC C39 3728 3.999 0.647 3.513

28POPC H9X 3729 3.902 0.608 3.476

28POPC H9Y 3730 4.056 0.563 3.559

28POPC C310 3731 4.084 0.698 3.398

28POPC H10X 3732 4.105 0.611 3.332

28POPC H10Y 3733 4.182 0.734 3.435

28POPC C311 3734 4.014 0.805 3.317

28POPC H11X 3735 4.024 0.904 3.368

28POPC H11Y 3736 3.904 0.788 3.315

28POPC C312 3737 4.073 0.801 3.177

28POPC H12X 3738 4.064 0.699 3.135

28POPC H12Y 3739 4.181 0.823 3.187

28POPC C313 3740 4.014 0.898 3.077

28POPC H13X 3741 3.999 0.996 3.128

28POPC H13Y 3742 3.913 0.863 3.045

28POPC C314 3743 4.112 0.913 2.961

28POPC H14X 3744 4.171 0.819 2.955

28POPC H14Y 3745 4.186 0.992 2.985

28POPC C315 3746 4.053 0.943 2.823

28POPC H15X 3747 4.132 0.929 2.746

28POPC H15Y 3748 4.026 1.050 2.826

28POPC C316 3749 3.926 0.870 2.780

28POPC H16X 3750 3.906 0.884 2.672

28POPC H16Y 3751 3.841 0.914 2.836

28POPC H16Z 3752 3.932 0.761 2.802

29POPC N 3753 5.485 4.227 4.990

29POPC C12 3754 5.484 4.117 5.095

29POPC H12A 3755 5.383 4.105 5.131

29POPC H12B 3756 5.547 4.147 5.178

29POPC C13 3757 5.376 4.198 4.891

29POPC H13A 3758 5.382 4.262 4.804

29POPC H13B 3759 5.385 4.096 4.858

29POPC H13C 3760 5.277 4.205 4.935

29POPC C14 3761 5.460 4.361 5.051

29POPC H14A 3762 5.460 4.437 4.974

29POPC H14B 3763 5.364 4.363 5.099

29POPC H14C 3764 5.537 4.386 5.123

29POPC C15 3765 5.615 4.223 4.915

29POPC H15A 3766 5.628 4.124 4.874

29POPC H15B 3767 5.700 4.244 4.977

29POPC H15C 3768 5.613 4.288 4.828

29POPC C11 3769 5.532 3.977 5.051

29POPC H11A 3770 5.528 3.907 5.137

29POPC H11B 3771 5.638 3.983 5.019

29POPC P 3772 5.500 3.831 4.836

29POPC O13 3773 5.391 3.828 4.736

29POPC O14 3774 5.540 3.707 4.905

29POPC O12 3775 5.447 3.928 4.949

29POPC O11 3776 5.626 3.914 4.784

29POPC C1 3777 5.695 3.878 4.664

29POPC HA 3778 5.645 3.798 4.605

29POPC HB 3779 5.797 3.841 4.689

29POPC C2 3780 5.710 4.001 4.570

29POPC HS 3781 5.756 4.082 4.630

29POPC O21 3782 5.578 4.035 4.522

29POPC C21 3783 5.522 4.142 4.570

29POPC O22 3784 5.578 4.235 4.626

29POPC C22 3785 5.370 4.131 4.555

29POPC H2R 3786 5.328 4.232 4.576

29POPC H2S 3787 5.333 4.061 4.633

29POPC C3 3788 5.805 3.974 4.451

29POPC HX 3789 5.893 3.917 4.489

29POPC HY 3790 5.844 4.070 4.411

29POPC O31 3791 5.740 3.893 4.351

29POPC C31 3792 5.677 3.963 4.259

29POPC O32 3793 5.695 4.081 4.233

29POPC C32 3794 5.572 3.874 4.196

29POPC H2X 3795 5.504 3.838 4.276

29POPC H2Y 3796 5.627 3.787 4.154

29POPC C23 3797 5.320 4.082 4.417

29POPC H3R 3798 5.331 3.972 4.411

29POPC H3S 3799 5.382 4.127 4.336

29POPC C24 3800 5.174 4.122 4.393

29POPC H4R 3801 5.164 4.233 4.401

29POPC H4S 3802 5.112 4.078 4.475

29POPC C25 3803 5.118 4.076 4.258

29POPC H5R 3804 5.007 4.084 4.261

29POPC H5S 3805 5.144 3.969 4.243

29POPC C26 3806 5.169 4.160 4.140

29POPC H6R 3807 5.280 4.163 4.143

29POPC H6S 3808 5.134 4.265 4.151

29POPC C27 3809 5.125 4.105 4.003

29POPC H7R 3810 5.017 4.129 3.990

29POPC H7S 3811 5.136 3.995 4.001

29POPC C28 3812 5.208 4.167 3.888

29POPC H8R 3813 5.307 4.115 3.884

29POPC H8S 3814 5.228 4.272 3.916

29POPC C29 3815 5.139 4.167 3.755

29POPC H91 3816 5.051 4.232 3.751

29POPC C210 3817 5.168 4.094 3.646

29POPC H101 3818 5.104 4.109 3.557

29POPC C211 3819 5.279 3.996 3.621

29POPC H11R 3820 5.249 3.891 3.640

29POPC H11S 3821 5.366 4.019 3.687

29POPC C212 3822 5.321 4.010 3.474

29POPC H12R 3823 5.424 3.971 3.460

29POPC H12S 3824 5.325 4.119 3.452

29POPC C213 3825 5.229 3.944 3.370

29POPC H13R 3826 5.175 4.025 3.317

29POPC H13S 3827 5.153 3.879 3.419

29POPC C214 3828 5.308 3.862 3.266

29POPC H14R 3829 5.327 3.759 3.304

29POPC H14S 3830 5.407 3.910 3.252

29POPC C215 3831 5.242 3.858 3.127

29POPC H15R 3832 5.202 3.959 3.104

29POPC H15S 3833 5.156 3.787 3.129

29POPC C216 3834 5.341 3.821 3.015

29POPC H16R 3835 5.429 3.889 3.021

29POPC H16S 3836 5.292 3.839 2.917

29POPC C217 3837 5.390 3.676 3.019

29POPC H17R 3838 5.303 3.607 3.009

29POPC H17S 3839 5.436 3.654 3.118

29POPC C218 3840 5.490 3.646 2.908

29POPC H18R 3841 5.585 3.701 2.925

29POPC H18S 3842 5.449 3.677 2.809

29POPC H18T 3843 5.512 3.537 2.904

29POPC C33 3844 5.488 3.947 4.088

29POPC H3X 3845 5.556 3.991 4.012

29POPC H3Y 3846 5.434 4.031 4.136

29POPC C34 3847 5.387 3.855 4.018

29POPC H4X 3848 5.350 3.905 3.925

29POPC H4Y 3849 5.299 3.839 4.084

29POPC C35 3850 5.446 3.719 3.980

29POPC H5X 3851 5.370 3.657 3.928

29POPC H5Y 3852 5.472 3.665 4.073

29POPC C36 3853 5.570 3.735 3.892

29POPC H6X 3854 5.639 3.650 3.909

29POPC H6Y 3855 5.630 3.824 3.919

29POPC C37 3856 5.531 3.743 3.745

29POPC H7X 3857 5.421 3.752 3.736

29POPC H7Y 3858 5.558 3.648 3.694

29POPC C38 3859 5.594 3.862 3.674

29POPC H8X 3860 5.566 3.956 3.728

29POPC H8Y 3861 5.547 3.865 3.573

29POPC C39 3862 5.746 3.859 3.658

29POPC H9X 3863 5.779 3.766 3.608

29POPC H9Y 3864 5.794 3.864 3.758

29POPC C310 3865 5.790 3.978 3.574

29POPC H10X 3866 5.900 3.991 3.585

29POPC H10Y 3867 5.744 4.069 3.617

29POPC C311 3868 5.750 3.966 3.426

29POPC H11X 3869 5.664 3.897 3.413

29POPC H11Y 3870 5.832 3.923 3.364

29POPC C312 3871 5.708 4.103 3.372

29POPC H12X 3872 5.794 4.173 3.381

29POPC H12Y 3873 5.629 4.140 3.440

29POPC C313 3874 5.658 4.100 3.227

29POPC H13X 3875 5.589 4.015 3.212

29POPC H13Y 3876 5.745 4.082 3.160

29POPC C314 3877 5.585 4.230 3.189

29POPC H14X 3878 5.582 4.239 3.079

29POPC H14Y 3879 5.644 4.317 3.226

29POPC C315 3880 5.445 4.232 3.251

29POPC H15X 3881 5.464 4.204 3.357

29POPC H15Y 3882 5.382 4.153 3.206

29POPC C316 3883 5.367 4.363 3.261

29POPC H16X 3884 5.299 4.379 3.174

29POPC H16Y 3885 5.435 4.450 3.270

29POPC H16Z 3886 5.301 4.361 3.351

30POPC N 3887 1.290 1.682 5.160

30POPC C12 3888 1.267 1.534 5.126

30POPC H12A 3889 1.198 1.490 5.197

30POPC H12B 3890 1.360 1.481 5.137

30POPC C13 3891 1.173 1.762 5.112

30POPC H13A 3892 1.160 1.746 5.006

30POPC H13B 3893 1.081 1.730 5.159

30POPC H13C 3894 1.187 1.868 5.128

30POPC C14 3895 1.306 1.698 5.307

30POPC H14A 3896 1.217 1.665 5.359

30POPC H14B 3897 1.390 1.639 5.342

30POPC H14C 3898 1.325 1.801 5.332

30POPC C15 3899 1.414 1.729 5.089

30POPC H15A 3900 1.401 1.718 4.983

30POPC H15B 3901 1.500 1.668 5.112

30POPC H15C 3902 1.437 1.833 5.109

30POPC C11 3903 1.215 1.504 4.985

30POPC H11A 3904 1.107 1.529 4.980

30POPC H11B 3905 1.223 1.395 4.964

30POPC P 3906 1.233 1.654 4.773

30POPC O13 3907 1.323 1.766 4.738

30POPC O14 3908 1.093 1.688 4.806

30POPC O12 3909 1.295 1.575 4.892

30POPC O11 3910 1.244 1.535 4.670

30POPC C1 3911 1.367 1.515 4.602

30POPC HA 3912 1.383 1.407 4.584

30POPC HB 3913 1.456 1.553 4.658

30POPC C2 3914 1.355 1.587 4.467

30POPC HS 3915 1.322 1.690 4.488

30POPC O21 3916 1.261 1.511 4.390

30POPC C21 3917 1.211 1.582 4.291

30POPC O22 3918 1.217 1.703 4.278

30POPC C22 3919 1.169 1.481 4.184

30POPC H2R 3920 1.151 1.383 4.232

30POPC H2S 3921 1.255 1.472 4.114

30POPC C3 3922 1.486 1.591 4.388

30POPC HX 3923 1.501 1.491 4.342

30POPC HY 3924 1.572 1.608 4.457

30POPC O31 3925 1.486 1.685 4.281

30POPC C31 3926 1.493 1.815 4.305

30POPC O32 3927 1.492 1.870 4.413

30POPC C32 3928 1.529 1.881 4.172

30POPC H2X 3929 1.573 1.981 4.192

30POPC H2Y 3930 1.435 1.893 4.112

30POPC C23 3931 1.043 1.525 4.109

30POPC H3R 3932 0.962 1.546 4.182

30POPC H3S 3933 1.011 1.443 4.041

30POPC C24 3934 1.063 1.646 4.019

30POPC H4R 3935 1.132 1.719 4.068

30POPC H4S 3936 0.967 1.699 4.005

30POPC C25 3937 1.118 1.607 3.882

30POPC H5R 3938 1.045 1.541 3.831

30POPC H5S 3939 1.214 1.550 3.892

30POPC C26 3940 1.142 1.731 3.797

30POPC H6R 3941 1.048 1.789 3.793

30POPC H6S 3942 1.168 1.700 3.694

30POPC C27 3943 1.254 1.820 3.853

30POPC H7R 3944 1.349 1.763 3.846

30POPC H7S 3945 1.238 1.848 3.959

30POPC C28 3946 1.259 1.949 3.772

30POPC H8R 3947 1.186 2.022 3.814

30POPC H8S 3948 1.220 1.921 3.672

30POPC C29 3949 1.397 2.009 3.761

30POPC H91 3950 1.420 2.087 3.834

30POPC C210 3951 1.487 1.979 3.666

30POPC H101 3952 1.584 2.031 3.669

30POPC C211 3953 1.475 1.875 3.556

30POPC H11R 3954 1.386 1.811 3.569

30POPC H11S 3955 1.563 1.808 3.563

30POPC C212 3956 1.472 1.932 3.412

30POPC H12R 3957 1.512 2.035 3.407

30POPC H12S 3958 1.367 1.936 3.375

30POPC C213 3959 1.542 1.846 3.307

30POPC H13R 3960 1.640 1.810 3.343

30POPC H13S 3961 1.559 1.911 3.218

30POPC C214 3962 1.455 1.728 3.263

30POPC H14R 3963 1.365 1.766 3.210

30POPC H14S 3964 1.420 1.673 3.353

30POPC C215 3965 1.531 1.632 3.171

30POPC H15R 3966 1.461 1.556 3.130

30POPC H15S 3967 1.606 1.579 3.233

30POPC C216 3968 1.606 1.702 3.057

30POPC H16R 3969 1.679 1.776 3.098

30POPC H16S 3970 1.534 1.758 2.994

30POPC C217 3971 1.685 1.603 2.971

30POPC H17R 3972 1.614 1.524 2.937

30POPC H17S 3973 1.762 1.553 3.034

30POPC C218 3974 1.750 1.669 2.850

30POPC H18R 3975 1.793 1.592 2.782

30POPC H18S 3976 1.833 1.737 2.879

30POPC H18T 3977 1.675 1.728 2.793

30POPC C33 3978 1.628 1.785 4.103

30POPC H3X 3979 1.570 1.695 4.074

30POPC H3Y 3980 1.705 1.754 4.177

30POPC C34 3981 1.703 1.829 3.979

30POPC H4X 3982 1.767 1.917 4.001

30POPC H4Y 3983 1.633 1.856 3.897

30POPC C35 3984 1.791 1.709 3.941

30POPC H5X 3985 1.849 1.676 4.030

30POPC H5Y 3986 1.866 1.741 3.866

30POPC C36 3987 1.713 1.586 3.890

30POPC H6X 3988 1.648 1.544 3.969

30POPC H6Y 3989 1.785 1.504 3.866

30POPC C37 3990 1.627 1.613 3.766

30POPC H7X 3991 1.689 1.656 3.684

30POPC H7Y 3992 1.547 1.686 3.791

30POPC C38 3993 1.563 1.484 3.717

30POPC H8X 3994 1.505 1.440 3.801

30POPC H8Y 3995 1.642 1.411 3.688

30POPC C39 3996 1.468 1.505 3.599

30POPC H9X 3997 1.527 1.514 3.505

30POPC H9Y 3998 1.411 1.600 3.612

30POPC C310 3999 1.369 1.390 3.589

30POPC H10X 4000 1.296 1.399 3.673

30POPC H10Y 4001 1.423 1.294 3.602

30POPC C311 4002 1.294 1.386 3.457

30POPC H11X 4003 1.292 1.282 3.418

30POPC H11Y 4004 1.349 1.446 3.381

30POPC C312 4005 1.152 1.437 3.476

30POPC H12X 4006 1.155 1.539 3.520

30POPC H12Y 4007 1.097 1.372 3.548

30POPC C313 4008 1.075 1.442 3.344

30POPC H13X 4009 0.973 1.478 3.367

30POPC H13Y 4010 1.065 1.340 3.300

30POPC C314 4011 1.139 1.539 3.246

30POPC H14X 4012 1.213 1.485 3.182

30POPC H14Y 4013 1.196 1.616 3.304

30POPC C315 4014 1.035 1.613 3.162

30POPC H15X 4015 1.086 1.694 3.106

30POPC H15Y 4016 0.962 1.661 3.231

30POPC C316 4017 0.961 1.522 3.065

30POPC H16X 4018 0.923 1.431 3.116

30POPC H16Y 4019 1.032 1.485 2.988

30POPC H16Z 4020 0.877 1.574 3.014

31POPC N 4021 3.716 5.727 5.110

31POPC C12 4022 3.614 5.816 5.039

31POPC H12A 4023 3.581 5.894 5.106

31POPC H12B 4024 3.527 5.757 5.013

31POPC C13 4025 3.735 5.602 5.029

31POPC H13A 4026 3.807 5.534 5.073

31POPC H13B 4027 3.641 5.551 5.013

31POPC H13C 4028 3.770 5.627 4.930

31POPC C14 4029 3.847 5.799 5.118

31POPC H14A 4030 3.921 5.740 5.172

31POPC H14B 4031 3.884 5.817 5.019

31POPC H14C 4032 3.836 5.895 5.166

31POPC C15 4033 3.667 5.691 5.247

31POPC H15A 4034 3.739 5.632 5.300

31POPC H15B 4035 3.575 5.634 5.240

31POPC H15C 4036 3.647 5.779 5.305

31POPC C11 4037 3.659 5.885 4.907

31POPC H11A 4038 3.743 5.954 4.927

31POPC H11B 4039 3.577 5.949 4.869

31POPC P 4040 3.588 5.712 4.726

31POPC O13 4041 3.474 5.680 4.816

31POPC O14 4042 3.655 5.600 4.658

31POPC O12 4043 3.700 5.787 4.811

31POPC O11 4044 3.547 5.824 4.624

31POPC C1 4045 3.649 5.900 4.559

31POPC HA 4046 3.697 5.839 4.479

31POPC HB 4047 3.728 5.933 4.630

31POPC C2 4048 3.594 6.030 4.494

31POPC HS 4049 3.533 6.086 4.568

31POPC O21 4050 3.521 5.997 4.376

31POPC C21 4051 3.400 5.951 4.401

31POPC O22 4052 3.335 5.973 4.501

31POPC C22 4053 3.352 5.872 4.281

31POPC H2R 4054 3.240 5.873 4.282

31POPC H2S 4055 3.389 5.768 4.288

31POPC C3 4056 3.714 6.119 4.454

31POPC HX 4057 3.774 6.123 4.548

31POPC HY 4058 3.682 6.223 4.432

31POPC O31 4059 3.792 6.058 4.350

31POPC C31 4060 3.755 6.097 4.229

31POPC O32 4061 3.706 6.205 4.201

31POPC C32 4062 3.780 5.984 4.131

31POPC H2X 4063 3.720 5.897 4.166

31POPC H2Y 4064 3.887 5.957 4.134

31POPC C23 4065 3.389 5.933 4.146

31POPC H3R 4066 3.497 5.921 4.127

31POPC H3S 4067 3.365 6.042 4.152

31POPC C24 4068 3.308 5.872 4.032

31POPC H4R 4069 3.199 5.891 4.046

31POPC H4S 4070 3.321 5.762 4.029

31POPC C25 4071 3.356 5.932 3.902

31POPC H5R 4072 3.451 5.881 3.875

31POPC H5S 4073 3.381 6.039 3.916

31POPC C26 4074 3.259 5.926 3.784

31POPC H6R 4075 3.185 6.009 3.791

31POPC H6S 4076 3.203 5.830 3.785

31POPC C27 4077 3.344 5.939 3.658

31POPC H7R 4078 3.378 5.839 3.624

31POPC H7S 4079 3.438 5.989 3.692

31POPC C28 4080 3.291 6.028 3.547

31POPC H8R 4081 3.376 6.077 3.495

31POPC H8S 4082 3.228 6.108 3.592

31POPC C29 4083 3.212 5.950 3.448

31POPC H91 4084 3.103 5.968 3.455

31POPC C210 4085 3.260 5.861 3.361

31POPC H101 4086 3.188 5.807 3.298

31POPC C211 4087 3.405 5.825 3.340

31POPC H11R 4088 3.453 5.797 3.436

31POPC H11S 4089 3.458 5.913 3.298

31POPC C212 4090 3.428 5.707 3.246

31POPC H12R 4091 3.396 5.613 3.295

31POPC H12S 4092 3.537 5.696 3.229

31POPC C213 4093 3.354 5.722 3.115

31POPC H13R 4094 3.369 5.824 3.074

31POPC H13S 4095 3.245 5.708 3.132

31POPC C214 4096 3.398 5.619 3.013

31POPC H14R 4097 3.429 5.524 3.062

31POPC H14S 4098 3.488 5.659 2.961

31POPC C215 4099 3.287 5.592 2.912

31POPC H15R 4100 3.228 5.684 2.895

31POPC H15S 4101 3.216 5.518 2.956

31POPC C216 4102 3.337 5.546 2.776

31POPC H16R 4103 3.250 5.550 2.707

31POPC H16S 4104 3.377 5.442 2.782

31POPC C217 4105 3.442 5.635 2.713

31POPC H17R 4106 3.453 5.602 2.607

31POPC H17S 4107 3.539 5.619 2.764

31POPC C218 4108 3.405 5.783 2.718

31POPC H18R 4109 3.462 5.841 2.642

31POPC H18S 4110 3.425 5.827 2.818

31POPC H18T 4111 3.296 5.794 2.696

31POPC C33 4112 3.736 6.022 3.988

31POPC H3X 4113 3.780 6.120 3.961

31POPC H3Y 4114 3.625 6.034 3.984

31POPC C34 4115 3.778 5.918 3.884

31POPC H4X 4116 3.709 5.831 3.887

31POPC H4Y 4117 3.881 5.882 3.906

31POPC C35 4118 3.779 5.979 3.744

31POPC H5X 4119 3.882 5.982 3.705

31POPC H5Y 4120 3.744 6.084 3.751

31POPC C36 4121 3.687 5.905 3.647

31POPC H6X 4122 3.599 5.866 3.702

31POPC H6Y 4123 3.738 5.817 3.602

31POPC C37 4124 3.641 6.001 3.538

31POPC H7X 4125 3.605 6.095 3.585

31POPC H7Y 4126 3.557 5.961 3.478

31POPC C38 4127 3.755 6.035 3.445

31POPC H8X 4128 3.851 6.020 3.500

31POPC H8Y 4129 3.748 6.143 3.419

31POPC C39 4130 3.762 5.966 3.311

31POPC H9X 4131 3.675 5.998 3.251

31POPC H9Y 4132 3.758 5.856 3.324

31POPC C310 4133 3.892 6.013 3.245

31POPC H10X 4134 3.934 6.090 3.313

31POPC H10Y 4135 3.874 6.064 3.148

31POPC C311 4136 4.001 5.907 3.230

31POPC H11X 4137 4.095 5.957 3.197

31POPC H11Y 4138 3.973 5.837 3.149

31POPC C312 4139 4.028 5.829 3.360

31POPC H12X 4140 3.964 5.862 3.445

31POPC H12Y 4141 4.133 5.851 3.389

31POPC C313 4142 4.013 5.679 3.342

31POPC H13X 4143 4.086 5.626 3.407

31POPC H13Y 4144 4.036 5.658 3.236

31POPC C314 4145 3.876 5.614 3.360

31POPC H14X 4146 3.804 5.657 3.286

31POPC H14Y 4147 3.835 5.630 3.462

31POPC C315 4148 3.891 5.464 3.330

31POPC H15X 4149 3.954 5.418 3.410

31POPC H15Y 4150 3.947 5.452 3.235

31POPC C316 4151 3.759 5.389 3.319

31POPC H16X 4152 3.698 5.432 3.236

31POPC H16Y 4153 3.702 5.396 3.413

31POPC H16Z 4154 3.779 5.282 3.296

32POPC N 4155 3.403 0.479 4.958

32POPC C12 4156 3.471 0.591 5.036

32POPC H12A 4157 3.578 0.576 5.034

32POPC H12B 4158 3.441 0.583 5.139

32POPC C13 4159 3.414 0.500 4.810

32POPC H13A 4160 3.365 0.592 4.782

32POPC H13B 4161 3.517 0.504 4.777

32POPC H13C 4162 3.367 0.419 4.757

32POPC C14 4163 3.461 0.346 4.992

32POPC H14A 4164 3.567 0.339 4.974

32POPC H14B 4165 3.439 0.316 5.094

32POPC H14C 4166 3.419 0.268 4.930

32POPC C15 4167 3.257 0.478 4.993

32POPC H15A 4168 3.211 0.570 4.961

32POPC H15B 4169 3.242 0.470 5.100

32POPC H15C 4170 3.207 0.394 4.947

32POPC C11 4171 3.440 0.736 4.993

32POPC H11A 4172 3.472 0.751 4.887

32POPC H11B 4173 3.501 0.805 5.055

32POPC P 4174 3.222 0.854 4.914

32POPC O13 4175 3.294 0.981 4.895

32POPC O14 4176 3.083 0.859 4.964

32POPC O12 4177 3.303 0.765 5.015

32POPC O11 4178 3.229 0.767 4.780

32POPC C1 4179 3.103 0.726 4.725

32POPC HA 4180 3.035 0.812 4.708

32POPC HB 4181 3.052 0.656 4.795

32POPC C2 4182 3.116 0.652 4.589

32POPC HS 4183 3.197 0.577 4.597

32POPC O21 4184 3.142 0.748 4.484

32POPC C21 4185 3.214 0.706 4.382

32POPC O22 4186 3.274 0.600 4.377

32POPC C22 4187 3.206 0.809 4.268

32POPC H2R 4188 3.174 0.906 4.313

32POPC H2S 4189 3.126 0.777 4.198

32POPC C3 4190 2.981 0.578 4.563

32POPC HX 4191 2.898 0.647 4.590

32POPC HY 4192 2.976 0.494 4.635

32POPC O31 4193 2.970 0.526 4.430

32POPC C31 4194 2.897 0.597 4.345

32POPC O32 4195 2.843 0.704 4.368

32POPC C32 4196 2.886 0.523 4.214

32POPC H2X 4197 2.794 0.461 4.220

32POPC H2Y 4198 2.976 0.457 4.204

32POPC C23 4199 3.338 0.833 4.192

32POPC H3R 4200 3.421 0.851 4.265

32POPC H3S 4201 3.327 0.926 4.132

32POPC C24 4202 3.379 0.720 4.096

32POPC H4R 4203 3.391 0.624 4.150

32POPC H4S 4204 3.477 0.747 4.053

32POPC C25 4205 3.284 0.702 3.977

32POPC H5R 4206 3.266 0.800 3.929

32POPC H5S 4207 3.186 0.665 4.014

32POPC C26 4208 3.339 0.603 3.871

32POPC H6R 4209 3.262 0.524 3.859

32POPC H6S 4210 3.431 0.555 3.909

32POPC C27 4211 3.369 0.669 3.735

32POPC H7R 4212 3.456 0.737 3.746

32POPC H7S 4213 3.282 0.733 3.708

32POPC C28 4214 3.393 0.574 3.617

32POPC H8R 4215 3.490 0.521 3.631

32POPC H8S 4216 3.405 0.635 3.525

32POPC C29 4217 3.282 0.472 3.601

32POPC H91 4218 3.284 0.391 3.675

32POPC C210 4219 3.184 0.471 3.509

32POPC H101 4220 3.111 0.389 3.514

32POPC C211 4221 3.159 0.567 3.395

32POPC H11R 4222 3.228 0.654 3.397

32POPC H11S 4223 3.057 0.609 3.407

32POPC C212 4224 3.169 0.497 3.258

32POPC H12R 4225 3.164 0.387 3.272

32POPC H12S 4226 3.269 0.517 3.212

32POPC C213 4227 3.056 0.534 3.160

32POPC H13R 4228 3.078 0.629 3.107

32POPC H13S 4229 2.966 0.554 3.222

32POPC C214 4230 3.018 0.420 3.063

32POPC H14R 4231 2.908 0.416 3.057

32POPC H14S 4232 3.047 0.323 3.108

32POPC C215 4233 3.071 0.425 2.917

32POPC H15R 4234 2.986 0.441 2.847

32POPC H15S 4235 3.109 0.324 2.892

32POPC C216 4236 3.180 0.529 2.887

32POPC H16R 4237 3.237 0.554 2.980

32POPC H16S 4238 3.133 0.624 2.853

32POPC C217 4239 3.287 0.489 2.784

32POPC H17R 4240 3.370 0.442 2.841

32POPC H17S 4241 3.327 0.582 2.738

32POPC C218 4242 3.249 0.392 2.673

32POPC H18R 4243 3.141 0.396 2.649

32POPC H18S 4244 3.274 0.288 2.705

32POPC H18T 4245 3.307 0.412 2.581

32POPC C33 4246 2.874 0.610 4.090

32POPC H3X 4247 2.943 0.697 4.096

32POPC H3Y 4248 2.771 0.649 4.083

32POPC C34 4249 2.908 0.526 3.966

32POPC H4X 4250 2.874 0.421 3.979

32POPC H4Y 4251 3.018 0.523 3.952

32POPC C35 4252 2.841 0.583 3.842

32POPC H5X 4253 2.731 0.576 3.856

32POPC H5Y 4254 2.866 0.524 3.752

32POPC C36 4255 2.879 0.729 3.823

32POPC H6X 4256 2.884 0.787 3.918

32POPC H6Y 4257 2.794 0.774 3.767

32POPC C37 4258 3.008 0.749 3.745

32POPC H7X 4259 2.990 0.715 3.640

32POPC H7Y 4260 3.087 0.683 3.787

32POPC C38 4261 3.054 0.895 3.747

32POPC H8X 4262 3.104 0.919 3.844

32POPC H8Y 4263 2.962 0.958 3.743

32POPC C39 4264 3.142 0.933 3.628

32POPC H9X 4265 3.149 0.844 3.562

32POPC H9Y 4266 3.245 0.959 3.661

32POPC C310 4267 3.081 1.049 3.549

32POPC H10X 4268 3.082 1.141 3.611

32POPC H10Y 4269 2.974 1.025 3.530

32POPC C311 4270 3.149 1.076 3.414

32POPC H11X 4271 3.240 1.138 3.428

32POPC H11Y 4272 3.076 1.135 3.354

32POPC C312 4273 3.183 0.953 3.330

32POPC H12X 4274 3.093 0.888 3.324

32POPC H12Y 4275 3.264 0.894 3.378

32POPC C313 4276 3.226 0.990 3.187

32POPC H13X 4277 3.330 1.027 3.188

32POPC H13Y 4278 3.161 1.072 3.149

32POPC C314 4279 3.213 0.869 3.093

32POPC H14X 4280 3.105 0.843 3.079

32POPC H14Y 4281 3.261 0.782 3.141

32POPC C315 4282 3.276 0.889 2.955

32POPC H15X 4283 3.262 0.794 2.898

32POPC H15Y 4284 3.385 0.904 2.971

32POPC C316 4285 3.222 1.000 2.866

32POPC H16X 4286 3.133 0.964 2.811

32POPC H16Y 4287 3.297 1.031 2.790

32POPC H16Z 4288 3.196 1.091 2.926

33POPC N 4289 1.867 0.861 4.719

33POPC C12 4290 1.747 0.930 4.783

33POPC H12A 4291 1.684 0.856 4.830

33POPC H12B 4292 1.688 0.978 4.706

33POPC C13 4293 1.916 0.763 4.822

33POPC H13A 4294 2.007 0.713 4.793

33POPC H13B 4295 1.934 0.817 4.913

33POPC H13C 4296 1.839 0.691 4.847

33POPC C14 4297 1.827 0.793 4.592

33POPC H14A 4298 1.909 0.737 4.550

33POPC H14B 4299 1.743 0.727 4.609

33POPC H14C 4300 1.796 0.866 4.518

33POPC C15 4301 1.976 0.961 4.695

33POPC H15A 4302 1.944 1.037 4.625

33POPC H15B 4303 2.066 0.915 4.658

33POPC H15C 4304 1.998 1.010 4.788

33POPC C11 4305 1.778 1.038 4.889

33POPC H11A 4306 1.681 1.068 4.935

33POPC H11B 4307 1.818 1.126 4.835

33POPC P 4308 1.895 1.091 5.110

33POPC O13 4309 2.012 1.041 5.185

33POPC O14 4310 1.764 1.106 5.177

33POPC O12 4311 1.873 0.995 4.986

33POPC O11 4312 1.928 1.229 5.039

33POPC C1 4313 2.057 1.276 5.001

33POPC HA 4314 2.116 1.196 4.951

33POPC HB 4315 2.112 1.310 5.091

33POPC C2 4316 2.044 1.396 4.902

33POPC HS 4317 1.986 1.477 4.951

33POPC O21 4318 1.981 1.352 4.781

33POPC C21 4319 1.852 1.359 4.776

33POPC O22 4320 1.775 1.395 4.863

33POPC C22 4321 1.814 1.305 4.639

33POPC H2R 4322 1.881 1.221 4.613

33POPC H2S 4323 1.834 1.386 4.565

33POPC C3 4324 2.180 1.455 4.858

33POPC HX 4325 2.229 1.495 4.950

33POPC HY 4326 2.161 1.543 4.792

33POPC O31 4327 2.265 1.356 4.796

33POPC C31 4328 2.251 1.342 4.663

33POPC O32 4329 2.187 1.416 4.591

33POPC C32 4330 2.331 1.220 4.615

33POPC H2X 4331 2.356 1.159 4.704

33POPC H2Y 4332 2.427 1.256 4.571

33POPC C23 4333 1.667 1.260 4.629

33POPC H3R 4334 1.602 1.330 4.686

33POPC H3S 4335 1.653 1.160 4.674

33POPC C24 4336 1.617 1.262 4.485

33POPC H4R 4337 1.606 1.369 4.459

33POPC H4S 4338 1.516 1.215 4.481

33POPC C25 4339 1.709 1.201 4.378

33POPC H5R 4340 1.708 1.090 4.383

33POPC H5S 4341 1.815 1.232 4.396

33POPC C26 4342 1.676 1.250 4.235

33POPC H6R 4343 1.736 1.191 4.162

33POPC H6S 4344 1.712 1.355 4.228

33POPC C27 4345 1.528 1.251 4.193

33POPC H7R 4346 1.503 1.356 4.166

33POPC H7S 4347 1.461 1.226 4.278

33POPC C28 4348 1.494 1.157 4.075

33POPC H8R 4349 1.441 1.070 4.118

33POPC H8S 4350 1.588 1.120 4.029

33POPC C29 4351 1.402 1.216 3.972

33POPC H91 4352 1.344 1.303 4.004

33POPC C210 4353 1.387 1.170 3.847

33POPC H101 4354 1.315 1.221 3.782

33POPC C211 4355 1.458 1.053 3.781

33POPC H11R 4356 1.394 1.015 3.699

33POPC H11S 4357 1.471 0.969 3.853

33POPC C212 4358 1.597 1.091 3.725

33POPC H12R 4359 1.667 1.088 3.811

33POPC H12S 4360 1.597 1.195 3.687

33POPC C213 4361 1.651 1.000 3.612

33POPC H13R 4362 1.650 0.895 3.649

33POPC H13S 4363 1.757 1.026 3.591

33POPC C214 4364 1.576 1.006 3.478

33POPC H14R 4365 1.472 0.975 3.500

33POPC H14S 4366 1.617 0.931 3.407

33POPC C215 4367 1.574 1.144 3.407

33POPC H15R 4368 1.569 1.225 3.483

33POPC H15S 4369 1.482 1.151 3.345

33POPC C216 4370 1.692 1.173 3.314

33POPC H16R 4371 1.683 1.108 3.224

33POPC H16S 4372 1.787 1.146 3.364

33POPC C217 4373 1.700 1.320 3.272

33POPC H17R 4374 1.596 1.357 3.254

33POPC H17S 4375 1.754 1.327 3.175

33POPC C218 4376 1.772 1.410 3.373

33POPC H18R 4377 1.729 1.396 3.475

33POPC H18S 4378 1.763 1.518 3.347

33POPC H18T 4379 1.880 1.386 3.376

33POPC C33 4380 2.256 1.132 4.512

33POPC H3X 4381 2.152 1.112 4.546

33POPC H3Y 4382 2.309 1.035 4.506

33POPC C34 4383 2.253 1.191 4.370

33POPC H4X 4384 2.357 1.216 4.341

33POPC H4Y 4385 2.196 1.287 4.368

33POPC C35 4386 2.195 1.095 4.265

33POPC H5X 4387 2.240 0.994 4.276

33POPC H5Y 4388 2.227 1.135 4.166

33POPC C36 4389 2.042 1.085 4.262

33POPC H6X 4390 2.000 1.188 4.256

33POPC H6Y 4391 2.007 1.042 4.359

33POPC C37 4392 1.986 1.001 4.146

33POPC H7X 4393 1.876 0.997 4.155

33POPC H7Y 4394 2.024 0.896 4.155

33POPC C38 4395 2.020 1.051 4.004

33POPC H8X 4396 1.966 0.987 3.931

33POPC H8Y 4397 2.128 1.037 3.984

33POPC C39 4398 1.981 1.198 3.979

33POPC H9X 4399 2.033 1.263 4.052

33POPC H9Y 4400 1.872 1.208 3.995

33POPC C310 4401 2.012 1.252 3.838

33POPC H10X 4402 2.121 1.268 3.823

33POPC H10Y 4403 1.965 1.353 3.830

33POPC C311 4404 1.958 1.166 3.724

33POPC H11X 4405 1.851 1.151 3.746

33POPC H11Y 4406 2.007 1.066 3.720

33POPC C312 4407 1.966 1.235 3.588

33POPC H12X 4408 1.979 1.344 3.603

33POPC H12Y 4409 1.869 1.221 3.537

33POPC C313 4410 2.077 1.186 3.494

33POPC H13X 4411 2.173 1.177 3.548

33POPC H13Y 4412 2.091 1.265 3.417

33POPC C314 4413 2.048 1.053 3.423

33POPC H14X 4414 1.939 1.030 3.426

33POPC H14Y 4415 2.097 0.972 3.481

33POPC C315 4416 2.094 1.049 3.277

33POPC H15X 4417 2.021 1.105 3.214

33POPC H15Y 4418 2.093 0.944 3.242

33POPC C316 4419 2.232 1.110 3.253

33POPC H16X 4420 2.278 1.071 3.160

33POPC H16Y 4421 2.301 1.090 3.338

33POPC H16Z 4422 2.225 1.221 3.243

34POPC N 4423 4.990 1.271 5.105

34POPC C12 4424 4.943 1.400 5.039

34POPC H12A 4425 4.948 1.480 5.111

34POPC H12B 4426 4.840 1.389 5.010

34POPC C13 4427 5.134 1.279 5.145

34POPC H13A 4428 5.195 1.303 5.060

34POPC H13B 4429 5.153 1.353 5.221

34POPC H13C 4430 5.171 1.184 5.183

34POPC C14 4431 4.905 1.240 5.225

34POPC H14A 4432 4.930 1.144 5.268

34POPC H14B 4433 4.916 1.314 5.302

34POPC H14C 4434 4.800 1.235 5.199

34POPC C15 4435 4.976 1.159 5.007

34POPC H15A 4436 4.872 1.149 4.977

34POPC H15B 4437 5.013 1.065 5.046

34POPC H15C 4438 5.031 1.185 4.918

34POPC C11 4439 5.017 1.445 4.910

34POPC H11A 4440 5.127 1.444 4.926

34POPC H11B 4441 4.990 1.550 4.886

34POPC P 4442 4.843 1.373 4.730

34POPC O13 4443 4.843 1.498 4.650

34POPC O14 4444 4.740 1.356 4.835

34POPC O12 4445 4.985 1.359 4.800

34POPC O11 4446 4.839 1.248 4.634

34POPC C1 4447 4.881 1.121 4.684

34POPC HA 4448 4.801 1.076 4.748

34POPC HB 4449 4.974 1.130 4.744

34POPC C2 4450 4.919 1.022 4.571

34POPC HS 4451 4.993 0.951 4.616

34POPC O21 4452 4.977 1.090 4.458

34POPC C21 4453 5.096 1.143 4.483

34POPC O22 4454 5.164 1.121 4.582

34POPC C22 4455 5.146 1.228 4.367

34POPC H2R 4456 5.233 1.176 4.321

34POPC H2S 4457 5.182 1.323 4.412

34POPC C3 4458 4.801 0.932 4.524

34POPC HX 4459 4.772 0.869 4.610

34POPC HY 4460 4.836 0.864 4.444

34POPC O31 4461 4.685 1.009 4.485

34POPC C31 4462 4.683 1.044 4.357

34POPC O32 4463 4.755 0.999 4.270

34POPC C32 4464 4.570 1.143 4.335

34POPC H2X 4465 4.535 1.184 4.432

34POPC H2Y 4466 4.486 1.086 4.288

34POPC C23 4467 5.041 1.260 4.259

34POPC H3R 4468 4.948 1.295 4.309

34POPC H3S 4469 5.015 1.169 4.200

34POPC C24 4470 5.090 1.372 4.167

34POPC H4R 4471 5.171 1.335 4.101

34POPC H4S 4472 5.133 1.452 4.231

34POPC C25 4473 4.977 1.434 4.083

34POPC H5R 4474 4.891 1.449 4.152

34POPC H5S 4475 4.946 1.363 4.004

34POPC C26 4476 5.013 1.572 4.024

34POPC H6R 4477 5.049 1.636 4.107

34POPC H6S 4478 4.921 1.618 3.982

34POPC C27 4479 5.121 1.568 3.914

34POPC H7R 4480 5.204 1.502 3.947

34POPC H7S 4481 5.163 1.670 3.899

34POPC C28 4482 5.064 1.519 3.780

34POPC H8R 4483 4.997 1.597 3.738

34POPC H8S 4484 4.998 1.432 3.804

34POPC C29 4485 5.166 1.476 3.678

34POPC H91 4486 5.193 1.550 3.601

34POPC C210 4487 5.217 1.352 3.670

34POPC H101 4488 5.288 1.328 3.589

34POPC C211 4489 5.188 1.240 3.767

34POPC H11R 4490 5.139 1.277 3.859

34POPC H11S 4491 5.285 1.196 3.800

34POPC C212 4492 5.102 1.125 3.711

34POPC H12R 4493 5.007 1.165 3.669

34POPC H12S 4494 5.076 1.058 3.795

34POPC C213 4495 5.176 1.043 3.605

34POPC H13R 4496 5.274 1.012 3.647

34POPC H13S 4497 5.196 1.107 3.517

34POPC C214 4498 5.102 0.916 3.560

34POPC H14R 4499 5.004 0.942 3.515

34POPC H14S 4500 5.083 0.854 3.650

34POPC C215 4501 5.184 0.830 3.461

34POPC H15R 4502 5.142 0.727 3.458

34POPC H15S 4503 5.287 0.822 3.502

34POPC C216 4504 5.193 0.883 3.316

34POPC H16R 4505 5.280 0.832 3.267

34POPC H16S 4506 5.211 0.993 3.313

34POPC C217 4507 5.076 0.843 3.227

34POPC H17R 4508 4.985 0.899 3.258

34POPC H17S 4509 5.065 0.735 3.250

34POPC C218 4510 5.093 0.845 3.075

34POPC H18R 4511 5.125 0.944 3.036

34POPC H18S 4512 4.997 0.819 3.027

34POPC H18T 4513 5.169 0.770 3.043

34POPC C33 4514 4.611 1.258 4.241

34POPC H3X 4515 4.671 1.219 4.156

34POPC H3Y 4516 4.673 1.331 4.296

34POPC C34 4517 4.486 1.326 4.185

34POPC H4X 4518 4.417 1.352 4.268

34POPC H4Y 4519 4.432 1.254 4.120

34POPC C35 4520 4.516 1.453 4.105

34POPC H5X 4521 4.559 1.528 4.175

34POPC H5Y 4522 4.421 1.494 4.065

34POPC C36 4523 4.614 1.432 3.990

34POPC H6X 4524 4.715 1.411 4.031

34POPC H6Y 4525 4.620 1.528 3.934

34POPC C37 4526 4.578 1.317 3.894

34POPC H7X 4527 4.481 1.340 3.845

34POPC H7Y 4528 4.565 1.222 3.950

34POPC C38 4529 4.685 1.294 3.787

34POPC H8X 4530 4.644 1.225 3.710

34POPC H8Y 4531 4.775 1.247 3.833

34POPC C39 4532 4.728 1.426 3.723

34POPC H9X 4533 4.789 1.484 3.796

34POPC H9Y 4534 4.636 1.487 3.707

34POPC C310 4535 4.806 1.414 3.593

34POPC H10X 4536 4.903 1.365 3.617

34POPC H10Y 4537 4.829 1.517 3.557

34POPC C311 4538 4.739 1.338 3.478

34POPC H11X 4539 4.632 1.369 3.470

34POPC H11Y 4540 4.741 1.229 3.499

34POPC C312 4541 4.809 1.367 3.345

34POPC H12X 4542 4.799 1.476 3.325

34POPC H12Y 4543 4.757 1.314 3.261

34POPC C313 4544 4.958 1.330 3.344

34POPC H13X 4545 5.003 1.339 3.445

34POPC H13Y 4546 5.013 1.403 3.281

34POPC C314 4547 4.986 1.188 3.293

34POPC H14X 4548 4.895 1.124 3.301

34POPC H14Y 4549 5.062 1.144 3.360

34POPC C315 4550 5.042 1.185 3.151

34POPC H15X 4551 5.128 1.114 3.148

34POPC H15Y 4552 5.086 1.284 3.125

34POPC C316 4553 4.939 1.144 3.046

34POPC H16X 4554 4.894 1.045 3.070

34POPC H16Y 4555 4.985 1.138 2.945

34POPC H16Z 4556 4.857 1.219 3.044

35POPC N 4557 0.634 2.321 5.091

35POPC C12 4558 0.766 2.267 5.037

35POPC H12A 4559 0.789 2.176 5.089

35POPC H12B 4560 0.844 2.339 5.059

35POPC C13 4561 0.531 2.212 5.087

35POPC H13A 4562 0.433 2.248 5.115

35POPC H13B 4563 0.525 2.175 4.986

35POPC H13C 4564 0.559 2.130 5.152

35POPC C14 4565 0.650 2.367 5.233

35POPC H14A 4566 0.687 2.287 5.296

35POPC H14B 4567 0.718 2.450 5.241

35POPC H14C 4568 0.554 2.395 5.275

35POPC C15 4569 0.586 2.432 5.003

35POPC H15A 4570 0.651 2.518 5.002

35POPC H15B 4571 0.484 2.461 5.026

35POPC H15C 4572 0.579 2.395 4.902

35POPC C11 4573 0.773 2.235 4.885

35POPC H11A 4574 0.871 2.189 4.862

35POPC H11B 4575 0.769 2.331 4.829

35POPC P 4576 0.590 2.158 4.712

35POPC O13 4577 0.588 2.300 4.672

35POPC O14 4578 0.460 2.092 4.733

35POPC O12 4579 0.670 2.144 4.848

35POPC O11 4580 0.680 2.070 4.613

35POPC C1 4581 0.676 1.930 4.647

35POPC HA 4582 0.574 1.889 4.632

35POPC HB 4583 0.706 1.918 4.754

35POPC C2 4584 0.773 1.838 4.567

35POPC HS 4585 0.769 1.735 4.611

35POPC O21 4586 0.904 1.886 4.591

35POPC C21 4587 1.002 1.830 4.524

35POPC O22 4588 1.002 1.719 4.475

35POPC C22 4589 1.102 1.943 4.497

35POPC H2R 4590 1.159 1.915 4.405

35POPC H2S 4591 1.171 1.950 4.583

35POPC C3 4592 0.731 1.822 4.417

35POPC HX 4593 0.684 1.722 4.407

35POPC HY 4594 0.820 1.821 4.351

35POPC O31 4595 0.631 1.920 4.380

35POPC C31 4596 0.651 1.983 4.266

35POPC O32 4597 0.742 1.962 4.188

35POPC C32 4598 0.537 2.081 4.241

35POPC H2X 4599 0.581 2.175 4.199

35POPC H2Y 4600 0.489 2.106 4.338

35POPC C23 4601 1.021 2.073 4.474

35POPC H3R 4602 0.965 2.102 4.566

35POPC H3S 4603 0.945 2.054 4.396

35POPC C24 4604 1.102 2.196 4.434

35POPC H4R 4605 1.165 2.226 4.521

35POPC H4S 4606 1.027 2.276 4.415

35POPC C25 4607 1.190 2.181 4.310

35POPC H5R 4608 1.274 2.112 4.333

35POPC H5S 4609 1.233 2.280 4.286

35POPC C26 4610 1.114 2.128 4.187

35POPC H6R 4611 1.092 2.020 4.201

35POPC H6S 4612 1.181 2.137 4.099

35POPC C27 4613 0.982 2.200 4.157

35POPC H7R 4614 1.000 2.309 4.157

35POPC H7S 4615 0.907 2.179 4.237

35POPC C28 4616 0.925 2.150 4.023

35POPC H8R 4617 0.920 2.039 4.030

35POPC H8S 4618 1.000 2.173 3.945

35POPC C29 4619 0.787 2.201 3.986

35POPC H91 4620 0.724 2.238 4.068

35POPC C210 4621 0.743 2.206 3.859

35POPC H101 4622 0.642 2.240 3.836

35POPC C211 4623 0.828 2.159 3.745

35POPC H11R 4624 0.851 2.051 3.757

35POPC H11S 4625 0.925 2.214 3.747

35POPC C212 4626 0.767 2.178 3.605

35POPC H12R 4627 0.665 2.134 3.603

35POPC H12S 4628 0.829 2.121 3.533

35POPC C213 4629 0.758 2.323 3.557

35POPC H13R 4630 0.860 2.362 3.535

35POPC H13S 4631 0.716 2.386 3.638

35POPC C214 4632 0.669 2.337 3.433

35POPC H14R 4633 0.663 2.444 3.403

35POPC H14S 4634 0.566 2.307 3.463

35POPC C215 4635 0.710 2.253 3.312

35POPC H15R 4636 0.627 2.254 3.238

35POPC H15S 4637 0.725 2.148 3.344

35POPC C216 4638 0.838 2.296 3.239

35POPC H16R 4639 0.921 2.302 3.313

35POPC H16S 4640 0.825 2.399 3.199

35POPC C217 4641 0.875 2.195 3.129

35POPC H17R 4642 0.794 2.192 3.053

35POPC H17S 4643 0.875 2.094 3.176

35POPC C218 4644 1.011 2.213 3.059

35POPC H18R 4645 1.039 2.120 3.006

35POPC H18S 4646 1.090 2.236 3.133

35POPC H18T 4647 1.007 2.296 2.984

35POPC C33 4648 0.437 2.019 4.143

35POPC H3X 4649 0.391 1.931 4.193

35POPC H3Y 4650 0.490 1.986 4.051

35POPC C34 4651 0.323 2.113 4.104

35POPC H4X 4652 0.363 2.198 4.046

35POPC H4Y 4653 0.285 2.150 4.202

35POPC C35 4654 0.202 2.051 4.032

35POPC H5X 4655 0.135 2.134 4.002

35POPC H5Y 4656 0.146 1.989 4.105

35POPC C36 4657 0.231 1.964 3.909

35POPC H6X 4658 0.134 1.942 3.859

35POPC H6Y 4659 0.273 1.867 3.942

35POPC C37 4660 0.325 2.025 3.805

35POPC H7X 4661 0.427 2.037 3.847

35POPC H7Y 4662 0.290 2.127 3.777

35POPC C38 4663 0.332 1.936 3.680

35POPC H8X 4664 0.230 1.932 3.635

35POPC H8Y 4665 0.360 1.833 3.710

35POPC C39 4666 0.432 1.986 3.577

35POPC H9X 4667 0.534 1.976 3.619

35POPC H9Y 4668 0.413 2.094 3.558

35POPC C310 4669 0.425 1.910 3.444

35POPC H10X 4670 0.321 1.906 3.408

35POPC H10Y 4671 0.461 1.805 3.458

35POPC C311 4672 0.509 1.980 3.337

35POPC H11X 4673 0.616 1.976 3.369

35POPC H11Y 4674 0.478 2.087 3.334

35POPC C312 4675 0.496 1.922 3.196

35POPC H12X 4676 0.389 1.916 3.167

35POPC H12Y 4677 0.539 1.820 3.194

35POPC C313 4678 0.572 2.012 3.098

35POPC H13X 4679 0.672 2.032 3.143

35POPC H13Y 4680 0.519 2.109 3.088

35POPC C314 4681 0.596 1.953 2.959

35POPC H14X 4682 0.658 1.860 2.968

35POPC H14Y 4683 0.655 2.026 2.900

35POPC C315 4684 0.467 1.921 2.883

35POPC H15X 4685 0.404 2.012 2.881

35POPC H15Y 4686 0.411 1.842 2.937

35POPC C316 4687 0.494 1.876 2.739

35POPC H16X 4688 0.398 1.861 2.686

35POPC H16Y 4689 0.552 1.781 2.738

35POPC H16Z 4690 0.553 1.952 2.685

36POPC N 4691 0.288 3.729 5.178

36POPC C12 4692 0.361 3.598 5.152

36POPC H12A 4693 0.289 3.518 5.158

36POPC H12B 4694 0.432 3.580 5.232

36POPC C13 4695 0.161 3.733 5.096

36POPC H13A 4696 0.112 3.829 5.100

36POPC H13B 4697 0.184 3.715 4.992

36POPC H13C 4698 0.091 3.656 5.126

36POPC C14 4699 0.256 3.743 5.323

36POPC H14A 4700 0.200 3.833 5.342

36POPC H14B 4701 0.197 3.659 5.360

36POPC H14C 4702 0.346 3.748 5.383

36POPC C15 4703 0.369 3.848 5.137

36POPC H15A 4704 0.463 3.853 5.190

36POPC H15B 4705 0.314 3.939 5.150

36POPC H15C 4706 0.390 3.842 5.031

36POPC C11 4707 0.437 3.579 5.018

36POPC H11A 4708 0.480 3.476 5.018

36POPC H11B 4709 0.522 3.651 5.010

36POPC P 4710 0.354 3.701 4.800

36POPC O13 4711 0.426 3.818 4.854

36POPC O14 4712 0.215 3.718 4.755

36POPC O12 4713 0.349 3.587 4.908

36POPC O11 4714 0.444 3.637 4.688

36POPC C1 4715 0.387 3.537 4.602

36POPC HA 4716 0.395 3.573 4.497

36POPC HB 4717 0.280 3.515 4.622

36POPC C2 4718 0.468 3.407 4.609

36POPC HS 4719 0.481 3.379 4.716

36POPC O21 4720 0.596 3.434 4.550

36POPC C21 4721 0.682 3.335 4.549

36POPC O22 4722 0.672 3.232 4.614

36POPC C22 4723 0.788 3.363 4.445

36POPC H2R 4724 0.861 3.279 4.446

36POPC H2S 4725 0.838 3.460 4.467

36POPC C3 4726 0.389 3.291 4.539

36POPC HX 4727 0.335 3.239 4.620

36POPC HY 4728 0.459 3.215 4.497

36POPC O31 4729 0.290 3.333 4.441

36POPC C31 4730 0.335 3.340 4.317

36POPC O32 4731 0.449 3.352 4.284

36POPC C32 4732 0.231 3.313 4.213

36POPC H2X 4733 0.224 3.405 4.151

36POPC H2Y 4734 0.132 3.288 4.257

36POPC C23 4735 0.712 3.367 4.313

36POPC H3R 4736 0.643 3.454 4.315

36POPC H3S 4737 0.657 3.271 4.307

36POPC C24 4738 0.785 3.373 4.181

36POPC H4R 4739 0.716 3.340 4.101

36POPC H4S 4740 0.861 3.293 4.190

36POPC C25 4741 0.838 3.513 4.150

36POPC H5R 4742 0.919 3.504 4.075

36POPC H5S 4743 0.881 3.559 4.242

36POPC C26 4744 0.731 3.608 4.094

36POPC H6R 4745 0.769 3.712 4.098

36POPC H6S 4746 0.640 3.603 4.158

36POPC C27 4747 0.694 3.572 3.950

36POPC H7R 4748 0.680 3.462 3.946

36POPC H7S 4749 0.778 3.597 3.881

36POPC C28 4750 0.563 3.634 3.901

36POPC H8R 4751 0.481 3.595 3.965

36POPC H8S 4752 0.544 3.594 3.799

36POPC C29 4753 0.565 3.785 3.903

36POPC H91 4754 0.623 3.830 3.984

36POPC C210 4755 0.500 3.865 3.817

36POPC H101 4756 0.514 3.974 3.824

36POPC C211 4757 0.414 3.821 3.703

36POPC H11R 4758 0.314 3.868 3.709

36POPC H11S 4759 0.402 3.710 3.706

36POPC C212 4760 0.480 3.857 3.571

36POPC H12R 4761 0.581 3.811 3.571

36POPC H12S 4762 0.486 3.968 3.572

36POPC C213 4763 0.414 3.819 3.437

36POPC H13R 4764 0.437 3.900 3.363

36POPC H13S 4765 0.304 3.818 3.448

36POPC C214 4766 0.467 3.688 3.374

36POPC H14R 4767 0.394 3.659 3.296

36POPC H14S 4768 0.468 3.607 3.450

36POPC C215 4769 0.605 3.697 3.308

36POPC H15R 4770 0.683 3.688 3.387

36POPC H15S 4771 0.615 3.798 3.263

36POPC C216 4772 0.633 3.594 3.196

36POPC H16R 4773 0.742 3.592 3.175

36POPC H16S 4774 0.583 3.630 3.103

36POPC C217 4775 0.586 3.451 3.224

36POPC H17R 4776 0.481 3.449 3.258

36POPC H17S 4777 0.648 3.409 3.307

36POPC C218 4778 0.598 3.361 3.100

36POPC H18R 4779 0.575 3.255 3.127

36POPC H18S 4780 0.701 3.364 3.060

36POPC H18T 4781 0.528 3.393 3.020

36POPC C33 4782 0.286 3.194 4.133

36POPC H3X 4783 0.299 3.106 4.199

36POPC H3Y 4784 0.386 3.218 4.089

36POPC C34 4785 0.197 3.150 4.019

36POPC H4X 4786 0.103 3.110 4.060

36POPC H4Y 4787 0.245 3.070 3.958

36POPC C35 4788 0.165 3.268 3.930

36POPC H5X 4789 0.118 3.349 3.990

36POPC H5Y 4790 0.083 3.225 3.868

36POPC C36 4791 0.278 3.326 3.843

36POPC H6X 4792 0.302 3.254 3.762

36POPC H6Y 4793 0.370 3.340 3.904

36POPC C37 4794 0.238 3.463 3.784

36POPC H7X 4795 0.290 3.541 3.843

36POPC H7Y 4796 0.129 3.478 3.797

36POPC C38 4797 0.272 3.483 3.635

36POPC H8X 4798 0.254 3.390 3.576

36POPC H8Y 4799 0.382 3.500 3.629

36POPC C39 4800 0.202 3.602 3.565

36POPC H9X 4801 0.249 3.612 3.465

36POPC H9Y 4802 0.228 3.693 3.624

36POPC C310 4803 0.049 3.626 3.553

36POPC H10X 4804 0.041 3.734 3.527

36POPC H10Y 4805 0.000 3.615 3.652

36POPC C311 4806 6.367 3.559 3.441

36POPC H11X 4807 6.283 3.629 3.419

36POPC H11Y 4808 6.317 3.466 3.476

36POPC C312 4809 0.045 3.531 3.312

36POPC H12X 4810 0.081 3.426 3.310

36POPC H12Y 4811 0.135 3.597 3.314

36POPC C313 4812 6.369 3.564 3.184

36POPC H13X 4813 6.274 3.612 3.216

36POPC H13Y 4814 6.338 3.471 3.132

36POPC C314 4815 0.038 3.661 3.086

36POPC H14X 4816 6.396 3.763 3.106

36POPC H14Y 4817 0.007 3.634 2.983

36POPC C315 4818 0.192 3.676 3.091

36POPC H15X 4819 0.219 3.737 3.180

36POPC H15Y 4820 0.221 3.733 3.001

36POPC C316 4821 0.279 3.552 3.084

36POPC H16X 4822 0.386 3.581 3.087

36POPC H16Y 4823 0.262 3.503 2.986

36POPC H16Z 4824 0.259 3.480 3.166

37POPC N 4825 0.666 4.484 4.929

37POPC C12 4826 0.590 4.573 4.829

37POPC H12A 4827 0.493 4.532 4.809

37POPC H12B 4828 0.643 4.573 4.735

37POPC C13 4829 0.611 4.502 5.067

37POPC H13A 4830 0.670 4.453 5.143

37POPC H13B 4831 0.606 4.608 5.088

37POPC H13C 4832 0.508 4.469 5.073

37POPC C14 4833 0.657 4.340 4.890

37POPC H14A 4834 0.704 4.323 4.795

37POPC H14B 4835 0.705 4.277 4.965

37POPC H14C 4836 0.555 4.307 4.883

37POPC C15 4837 0.809 4.526 4.930

37POPC H15A 4838 0.817 4.632 4.949

37POPC H15B 4839 0.858 4.513 4.835

37POPC H15C 4840 0.868 4.474 5.003

37POPC C11 4841 0.565 4.722 4.867

37POPC H11A 4842 0.517 4.773 4.781

37POPC H11B 4843 0.664 4.772 4.880

37POPC P 4844 0.333 4.711 4.993

37POPC O13 4845 0.256 4.837 4.975

37POPC O14 4846 0.304 4.636 5.118

37POPC O12 4847 0.488 4.738 4.987

37POPC O11 4848 0.308 4.613 4.872

37POPC C1 4849 0.294 4.473 4.898

37POPC HA 4850 0.210 4.455 4.968

37POPC HB 4851 0.385 4.428 4.941

37POPC C2 4852 0.259 4.396 4.768

37POPC HS 4853 0.224 4.296 4.803

37POPC O21 4854 0.370 4.386 4.676

37POPC C21 4855 0.429 4.268 4.674

37POPC O22 4856 0.424 4.187 4.765

37POPC C22 4857 0.519 4.253 4.551

37POPC H2R 4858 0.608 4.196 4.585

37POPC H2S 4859 0.552 4.353 4.518

37POPC C3 4860 0.137 4.465 4.701

37POPC HX 4861 0.104 4.550 4.766

37POPC HY 4862 0.055 4.390 4.699

37POPC O31 4863 0.162 4.510 4.567

37POPC C31 4864 0.056 4.572 4.517

37POPC O32 4865 6.348 4.581 4.573

37POPC C32 4866 0.098 4.646 4.392

37POPC H2X 4867 0.036 4.611 4.307

37POPC H2Y 4868 0.204 4.623 4.371

37POPC C23 4869 0.455 4.174 4.434

37POPC H3R 4870 0.400 4.086 4.473

37POPC H3S 4871 0.538 4.133 4.371

37POPC C24 4872 0.366 4.257 4.341

37POPC H4R 4873 0.428 4.332 4.288

37POPC H4S 4874 0.290 4.312 4.400

37POPC C25 4875 0.295 4.169 4.237

37POPC H5R 4876 0.233 4.095 4.293

37POPC H5S 4877 0.369 4.109 4.181

37POPC C26 4878 0.201 4.244 4.141

37POPC H6R 4879 0.137 4.312 4.201

37POPC H6S 4880 0.134 4.169 4.092

37POPC C27 4881 0.265 4.329 4.030

37POPC H7R 4882 0.333 4.405 4.074

37POPC H7S 4883 0.182 4.385 3.981

37POPC C28 4884 0.336 4.249 3.919

37POPC H8R 4885 0.344 4.313 3.829

37POPC H8S 4886 0.271 4.163 3.890

37POPC C29 4887 0.472 4.200 3.960

37POPC H91 4888 0.478 4.094 3.989

37POPC C210 4889 0.581 4.278 3.974

37POPC H101 4890 0.676 4.233 4.008

37POPC C211 4891 0.586 4.426 3.946

37POPC H11R 4892 0.581 4.483 4.041

37POPC H11S 4893 0.498 4.459 3.885

37POPC C212 4894 0.713 4.467 3.870

37POPC H12R 4895 0.803 4.447 3.932

37POPC H12S 4896 0.710 4.577 3.855

37POPC C213 4897 0.728 4.399 3.734

37POPC H13R 4898 0.628 4.375 3.692

37POPC H13S 4899 0.783 4.304 3.751

37POPC C214 4900 0.805 4.485 3.634

37POPC H14R 4901 0.896 4.524 3.685

37POPC H14S 4902 0.743 4.573 3.605

37POPC C215 4903 0.851 4.409 3.508

37POPC H15R 4904 0.914 4.324 3.542

37POPC H15S 4905 0.915 4.476 3.447

37POPC C216 4906 0.740 4.353 3.418

37POPC H16R 4907 0.678 4.277 3.471

37POPC H16S 4908 0.792 4.303 3.334

37POPC C217 4909 0.645 4.450 3.351

37POPC H17R 4910 0.650 4.549 3.402

37POPC H17S 4911 0.542 4.410 3.361

37POPC C218 4912 0.677 4.464 3.202

37POPC H18R 4913 0.611 4.539 3.153

37POPC H18S 4914 0.665 4.366 3.150

37POPC H18T 4915 0.783 4.495 3.192

37POPC C33 4916 0.081 4.797 4.415

37POPC H3X 4917 0.095 4.823 4.523

37POPC H3Y 4918 6.377 4.827 4.388

37POPC C34 4919 0.181 4.882 4.338

37POPC H4X 4920 0.283 4.867 4.381

37POPC H4Y 4921 0.155 4.989 4.348

37POPC C35 4922 0.186 4.845 4.190

37POPC H5X 4923 0.085 4.856 4.146

37POPC H5Y 4924 0.217 4.739 4.178

37POPC C36 4925 0.286 4.933 4.115

37POPC H6X 4926 0.384 4.932 4.168

37POPC H6Y 4927 0.249 5.038 4.117

37POPC C37 4928 0.307 4.887 3.972

37POPC H7X 4929 0.362 4.967 3.919

37POPC H7Y 4930 0.211 4.871 3.919

37POPC C38 4931 0.388 4.757 3.963

37POPC H8X 4932 0.330 4.669 3.998

37POPC H8Y 4933 0.477 4.768 4.030

37POPC C39 4934 0.438 4.737 3.821

37POPC H9X 4935 0.537 4.687 3.821

37POPC H9Y 4936 0.449 4.838 3.775

37POPC C310 4937 0.344 4.661 3.730

37POPC H10X 4938 0.241 4.701 3.743

37POPC H10Y 4939 0.345 4.554 3.758

37POPC C311 4940 0.386 4.679 3.585

37POPC H11X 4941 0.495 4.657 3.575

37POPC H11Y 4942 0.370 4.785 3.556

37POPC C312 4943 0.308 4.588 3.492

37POPC H12X 4944 0.200 4.608 3.508

37POPC H12Y 4945 0.327 4.481 3.515

37POPC C313 4946 0.344 4.620 3.348

37POPC H13X 4947 0.452 4.601 3.328

37POPC H13Y 4948 0.329 4.730 3.340

37POPC C314 4949 0.257 4.552 3.242

37POPC H14X 4950 0.244 4.446 3.269

37POPC H14Y 4951 0.309 4.555 3.144

37POPC C315 4952 0.125 4.628 3.232

37POPC H15X 4953 0.147 4.727 3.187

37POPC H15Y 4954 0.086 4.643 3.334

37POPC C316 4955 0.014 4.565 3.152

37POPC H16X 4956 6.386 4.465 3.191

37POPC H16Y 4957 0.039 4.557 3.044

37POPC H16Z 4958 6.325 4.632 3.159

38POPC N 4959 2.371 6.225 4.941

38POPC C12 4960 2.310 6.135 5.047

38POPC H12A 4961 2.330 6.176 5.145

38POPC H12B 4962 2.359 6.038 5.044

38POPC C13 4963 2.318 6.365 4.952

38POPC H13A 4964 2.329 0.003 5.052

38POPC H13B 4965 2.375 0.031 4.889

38POPC H13C 4966 2.213 6.368 4.924

38POPC C14 4967 2.518 6.232 4.964

38POPC H14A 4968 2.540 6.251 5.068

38POPC H14B 4969 2.567 6.140 4.934

38POPC H14C 4970 2.559 6.315 4.909

38POPC C15 4971 2.344 6.172 4.804

38POPC H15A 4972 2.381 6.071 4.793

38POPC H15B 4973 2.387 6.234 4.727

38POPC H15C 4974 2.238 6.163 4.788

38POPC C11 4975 2.159 6.107 5.033

38POPC H11A 4976 2.107 6.203 5.013

38POPC H11B 4977 2.120 6.071 5.131

38POPC P 4978 2.183 5.865 4.942

38POPC O13 4979 2.080 5.773 4.888

38POPC O14 4980 2.225 5.843 5.082

38POPC O12 4981 2.132 6.014 4.929

38POPC O11 4982 2.312 5.870 4.848

38POPC C1 4983 2.295 5.834 4.710

38POPC HA 4984 2.234 5.909 4.655

38POPC HB 4985 2.243 5.736 4.702

38POPC C2 4986 2.430 5.816 4.635

38POPC HS 4987 2.498 5.756 4.699

38POPC O21 4988 2.485 5.946 4.599

38POPC C21 4989 2.607 5.974 4.641

38POPC O22 4990 2.669 5.915 4.728

38POPC C22 4991 2.658 6.105 4.579

38POPC H2R 4992 2.762 6.116 4.616

38POPC H2S 4993 2.600 6.189 4.621

38POPC C3 4994 2.404 5.732 4.506

38POPC HX 4995 2.340 5.645 4.532

38POPC HY 4996 2.502 5.689 4.476

38POPC O31 4997 2.341 5.811 4.401

38POPC C31 4998 2.364 5.759 4.280

38POPC O32 4999 2.413 5.649 4.260

38POPC C32 5000 2.315 5.853 4.170

38POPC H2X 5001 2.309 5.956 4.212

38POPC H2Y 5002 2.214 5.821 4.139

38POPC C23 5003 2.663 6.111 4.424

38POPC H3R 5004 2.718 6.023 4.383

38POPC H3S 5005 2.722 6.201 4.396

38POPC C24 5006 2.527 6.121 4.354

38POPC H4R 5007 2.476 6.023 4.361

38POPC H4S 5008 2.544 6.142 4.246

38POPC C25 5009 2.436 6.229 4.414

38POPC H5R 5010 2.489 6.327 4.413

38POPC H5S 5011 2.413 6.201 4.519

38POPC C26 5012 2.303 6.242 4.341

38POPC H6R 5013 2.232 6.296 4.407

38POPC H6S 5014 2.261 6.141 4.322

38POPC C27 5015 2.316 6.322 4.211

38POPC H7R 5016 2.389 6.274 4.142

38POPC H7S 5017 2.357 0.022 4.238

38POPC C28 5018 2.181 6.343 4.142

38POPC H8R 5019 2.188 0.028 4.071

38POPC H8S 5020 2.110 6.376 4.221

38POPC C29 5021 2.132 6.216 4.075

38POPC H91 5022 2.138 6.126 4.138

38POPC C210 5023 2.085 6.204 3.950

38POPC H101 5024 2.054 6.104 3.916

38POPC C211 5025 2.068 6.314 3.849

38POPC H11R 5026 2.095 0.014 3.889

38POPC H11S 5027 1.960 6.326 3.825

38POPC C212 5028 2.144 6.293 3.716

38POPC H12R 5029 2.249 6.325 3.731

38POPC H12S 5030 2.099 6.360 3.640

38POPC C213 5031 2.153 6.150 3.662

38POPC H13R 5032 2.059 6.094 3.684

38POPC H13S 5033 2.234 6.095 3.715

38POPC C214 5034 2.174 6.140 3.510

38POPC H14R 5035 2.282 6.136 3.487

38POPC H14S 5036 2.135 6.232 3.460

38POPC C215 5037 2.100 6.017 3.453

38POPC H15R 5038 1.995 6.017 3.491

38POPC H15S 5039 2.146 5.924 3.491

38POPC C216 5040 2.093 6.008 3.301

38POPC H16R 5041 2.021 6.083 3.264

38POPC H16S 5042 2.052 5.908 3.272

38POPC C217 5043 2.228 6.027 3.232

38POPC H17R 5044 2.298 5.948 3.265

38POPC H17S 5045 2.272 6.125 3.260

38POPC C218 5046 2.214 6.021 3.080

38POPC H18R 5047 2.313 6.035 3.032

38POPC H18S 5048 2.148 6.103 3.046

38POPC H18T 5049 2.169 5.925 3.047

38POPC C33 5050 2.409 5.855 4.046

38POPC H3X 5051 2.414 5.754 4.001

38POPC H3Y 5052 2.512 5.881 4.079

38POPC C34 5053 2.365 5.956 3.940

38POPC H4X 5054 2.357 6.056 3.989

38POPC H4Y 5055 2.265 5.930 3.901

38POPC C35 5056 2.465 5.970 3.824

38POPC H5X 5057 2.567 5.980 3.866

38POPC H5Y 5058 2.444 6.064 3.768

38POPC C36 5059 2.465 5.854 3.723

38POPC H6X 5060 2.487 5.758 3.774

38POPC H6Y 5061 2.546 5.877 3.651

38POPC C37 5062 2.340 5.841 3.637

38POPC H7X 5063 2.325 5.941 3.591

38POPC H7Y 5064 2.252 5.816 3.699

38POPC C38 5065 2.353 5.739 3.521

38POPC H8X 5066 2.255 5.733 3.470

38POPC H8Y 5067 2.375 5.638 3.562

38POPC C39 5068 2.459 5.777 3.416

38POPC H9X 5069 2.560 5.755 3.457

38POPC H9Y 5070 2.454 5.886 3.396

38POPC C310 5071 2.440 5.699 3.285

38POPC H10X 5072 2.343 5.726 3.238

38POPC H10Y 5073 2.437 5.591 3.309

38POPC C311 5074 2.553 5.722 3.184

38POPC H11X 5075 2.543 5.648 3.102

38POPC H11Y 5076 2.651 5.707 3.235

38POPC C312 5077 2.559 5.860 3.121

38POPC H12X 5078 2.583 5.935 3.199

38POPC H12Y 5079 2.459 5.886 3.079

38POPC C313 5080 2.664 5.864 3.010

38POPC H13X 5081 2.624 5.814 2.919

38POPC H13Y 5082 2.754 5.806 3.042

38POPC C314 5083 2.709 6.006 2.978

38POPC H14X 5084 2.776 6.006 2.889

38POPC H14Y 5085 2.763 6.051 3.064

38POPC C315 5086 2.598 6.104 2.950

38POPC H15X 5087 2.646 6.198 2.915

38POPC H15Y 5088 2.544 6.124 3.045

38POPC C316 5089 2.507 6.051 2.842

38POPC H16X 5090 2.573 5.997 2.771

38POPC H16Y 5091 2.453 6.133 2.789

38POPC H16Z 5092 2.434 5.977 2.881

39POPC N 5093 2.407 0.628 4.935

39POPC C12 5094 2.394 0.539 4.812

39POPC H12A 5095 2.491 0.534 4.764

39POPC H12B 5096 2.328 0.586 4.741

39POPC C13 5097 2.544 0.610 4.994

39POPC H13A 5098 2.621 0.648 4.929

39POPC H13B 5099 2.554 0.660 5.089

39POPC H13C 5100 2.565 0.505 5.009

39POPC C14 5101 2.394 0.773 4.899

39POPC H14A 5102 2.405 0.835 4.987

39POPC H14B 5103 2.475 0.804 4.833

39POPC H14C 5104 2.299 0.796 4.853

39POPC C15 5105 2.306 0.590 5.040

39POPC H15A 5106 2.204 0.601 5.007

39POPC H15B 5107 2.318 0.648 5.130

39POPC H15C 5108 2.321 0.487 5.067

39POPC C11 5109 2.345 0.392 4.834

39POPC H11A 5110 2.337 0.341 4.736

39POPC H11B 5111 2.241 0.394 4.874

39POPC P 5112 2.566 0.259 4.879

39POPC O13 5113 2.571 0.116 4.914

39POPC O14 5114 2.675 0.343 4.929

39POPC O12 5115 2.427 0.320 4.924

39POPC O11 5116 2.561 0.268 4.722

39POPC C1 5117 2.679 0.308 4.651

39POPC HA 5118 2.767 0.246 4.679

39POPC HB 5119 2.704 0.414 4.676

39POPC C2 5120 2.658 0.299 4.497

39POPC HS 5121 2.732 0.368 4.451

39POPC O21 5122 2.526 0.335 4.454

39POPC C21 5123 2.481 0.451 4.492

39POPC O22 5124 2.541 0.539 4.552

39POPC C22 5125 2.334 0.464 4.455

39POPC H2R 5126 2.317 0.572 4.434

39POPC H2S 5127 2.273 0.435 4.543

39POPC C3 5128 2.688 0.152 4.447

39POPC HX 5129 2.606 0.089 4.488

39POPC HY 5130 2.781 0.117 4.496

39POPC O31 5131 2.695 0.133 4.303

39POPC C31 5132 2.803 0.191 4.249

39POPC O32 5133 2.877 0.265 4.311

39POPC C32 5134 2.826 0.161 4.102

39POPC H2X 5135 2.856 0.054 4.093

39POPC H2Y 5136 2.913 0.224 4.072

39POPC C23 5137 2.292 0.379 4.333

39POPC H3R 5138 2.294 0.271 4.362

39POPC H3S 5139 2.366 0.391 4.251

39POPC C24 5140 2.152 0.415 4.282

39POPC H4R 5141 2.082 0.417 4.369

39POPC H4S 5142 2.117 0.336 4.212

39POPC C25 5143 2.145 0.550 4.208

39POPC H5R 5144 2.195 0.630 4.267

39POPC H5S 5145 2.039 0.580 4.194

39POPC C26 5146 2.212 0.542 4.071

39POPC H6R 5147 2.160 0.466 4.008

39POPC H6S 5148 2.317 0.509 4.085

39POPC C27 5149 2.217 0.675 3.996

39POPC H7R 5150 2.286 0.743 4.051

39POPC H7S 5151 2.116 0.721 3.997

39POPC C28 5152 2.265 0.659 3.851

39POPC H8R 5153 2.195 0.592 3.796

39POPC H8S 5154 2.365 0.611 3.849

39POPC C29 5155 2.270 0.795 3.787

39POPC H91 5156 2.247 0.878 3.855

39POPC C210 5157 2.303 0.825 3.661

39POPC H101 5158 2.302 0.930 3.630

39POPC C211 5159 2.340 0.731 3.549

39POPC H11R 5160 2.294 0.631 3.560

39POPC H11S 5161 2.450 0.718 3.545

39POPC C212 5162 2.294 0.796 3.417

39POPC H12R 5163 2.321 0.904 3.416

39POPC H12S 5164 2.184 0.787 3.408

39POPC C213 5165 2.360 0.734 3.295

39POPC H13R 5166 2.378 0.627 3.320

39POPC H13S 5167 2.459 0.782 3.277

39POPC C214 5168 2.280 0.741 3.166

39POPC H14R 5169 2.262 0.846 3.134

39POPC H14S 5170 2.181 0.693 3.182

39POPC C215 5171 2.357 0.667 3.056

39POPC H15R 5172 2.420 0.591 3.106

39POPC H15S 5173 2.426 0.736 3.002

39POPC C216 5174 2.270 0.583 2.965

39POPC H16R 5175 2.224 0.660 2.899

39POPC H16S 5176 2.190 0.531 3.022

39POPC C217 5177 2.340 0.487 2.869

39POPC H17R 5178 2.442 0.525 2.850

39POPC H17S 5179 2.285 0.490 2.773

39POPC C218 5180 2.348 0.341 2.909

39POPC H18R 5181 2.296 0.279 2.833

39POPC H18S 5182 2.299 0.325 3.007

39POPC H18T 5183 2.453 0.306 2.912

39POPC C33 5184 2.708 0.188 4.010

39POPC H3X 5185 2.649 0.093 4.009

39POPC H3Y 5186 2.744 0.203 3.907

39POPC C34 5187 2.619 0.306 4.050

39POPC H4X 5188 2.672 0.403 4.035

39POPC H4Y 5189 2.588 0.301 4.157

39POPC C35 5190 2.492 0.305 3.966

39POPC H5X 5191 2.526 0.304 3.860

39POPC H5Y 5192 2.435 0.399 3.977

39POPC C36 5193 2.406 0.184 4.004

39POPC H6X 5194 2.317 0.218 4.061

39POPC H6Y 5195 2.462 0.120 4.075

39POPC C37 5196 2.362 0.093 3.888

39POPC H7X 5197 2.263 0.129 3.853

39POPC H7Y 5198 2.348 6.390 3.928

39POPC C38 5199 2.456 0.084 3.767

39POPC H8X 5200 2.450 6.382 3.723

39POPC H8Y 5201 2.562 0.097 3.798

39POPC C39 5202 2.415 0.187 3.661

39POPC H9X 5203 2.503 0.249 3.633

39POPC H9Y 5204 2.340 0.256 3.705

39POPC C310 5205 2.356 0.123 3.536

39POPC H10X 5206 2.299 0.030 3.560

39POPC H10Y 5207 2.440 0.089 3.473

39POPC C311 5208 2.262 0.218 3.462

39POPC H11X 5209 2.292 0.323 3.482

39POPC H11Y 5210 2.160 0.205 3.503

39POPC C312 5211 2.263 0.192 3.311

39POPC H12X 5212 2.184 0.250 3.259

39POPC H12Y 5213 2.244 0.084 3.295

39POPC C313 5214 2.396 0.230 3.245

39POPC H13X 5215 2.394 0.202 3.138

39POPC H13Y 5216 2.477 0.170 3.292

39POPC C314 5217 2.429 0.378 3.256

39POPC H14X 5218 2.438 0.408 3.362

39POPC H14Y 5219 2.344 0.435 3.213

39POPC C315 5220 2.561 0.413 3.184

39POPC H15X 5221 2.540 0.422 3.075

39POPC H15Y 5222 2.631 0.328 3.193

39POPC C316 5223 2.629 0.540 3.237

39POPC H16X 5224 2.622 0.545 3.348

39POPC H16Y 5225 2.588 0.634 3.195

39POPC H16Z 5226 2.737 0.540 3.209

40POPC N 5227 5.888 2.328 4.783

40POPC C12 5228 5.980 2.446 4.818

40POPC H12A 5229 5.923 2.537 4.821

40POPC H12B 5230 6.054 2.458 4.740

40POPC C13 5231 5.759 2.342 4.859

40POPC H13A 5232 5.690 2.261 4.842

40POPC H13B 5233 5.779 2.347 4.965

40POPC H13C 5234 5.710 2.436 4.838

40POPC C14 5235 5.859 2.323 4.636

40POPC H14A 5236 5.949 2.306 4.578

40POPC H14B 5237 5.790 2.243 4.612

40POPC H14C 5238 5.815 2.415 4.601

40POPC C15 5239 5.953 2.201 4.828

40POPC H15A 5240 5.977 2.211 4.933

40POPC H15B 5241 6.045 2.184 4.774

40POPC H15C 5242 5.887 2.117 4.817

40POPC C11 5243 6.059 2.439 4.953

40POPC H11A 5244 6.125 2.528 4.960

40POPC H11B 5245 6.128 2.352 4.952

40POPC P 5246 5.887 2.554 5.112

40POPC O13 5247 5.919 2.592 5.252

40POPC O14 5248 5.744 2.527 5.085

40POPC O12 5249 5.973 2.429 5.067

40POPC O11 5250 5.937 2.668 5.015

40POPC C1 5251 5.880 2.799 5.020

40POPC HA 5252 5.962 2.874 5.011

40POPC HB 5253 5.829 2.820 5.117

40POPC C2 5254 5.777 2.828 4.906

40POPC HS 5255 5.709 2.906 4.947

40POPC O21 5256 5.708 2.710 4.859

40POPC C21 5257 5.579 2.726 4.838

40POPC O22 5258 5.506 2.804 4.896

40POPC C22 5259 5.532 2.636 4.724

40POPC H2R 5260 5.457 2.569 4.770

40POPC H2S 5261 5.617 2.575 4.686

40POPC C3 5262 5.848 2.886 4.782

40POPC HX 5263 5.909 2.975 4.809

40POPC HY 5264 5.766 2.922 4.716

40POPC O31 5265 5.929 2.784 4.721

40POPC C31 5266 5.924 2.780 4.588

40POPC O32 5267 5.866 2.861 4.518

40POPC C32 5268 5.993 2.654 4.541

40POPC H2X 5269 5.943 2.568 4.591

40POPC H2Y 5270 6.099 2.660 4.574

40POPC C23 5271 5.470 2.719 4.608

40POPC H3R 5272 5.441 2.820 4.643

40POPC H3S 5273 5.377 2.671 4.570

40POPC C24 5274 5.565 2.735 4.489

40POPC H4R 5275 5.593 2.636 4.447

40POPC H4S 5276 5.658 2.781 4.531

40POPC C25 5277 5.514 2.828 4.378

40POPC H5R 5278 5.489 2.927 4.423

40POPC H5S 5279 5.423 2.787 4.331

40POPC C26 5280 5.620 2.854 4.270

40POPC H6R 5281 5.715 2.886 4.318

40POPC H6S 5282 5.586 2.939 4.206

40POPC C27 5283 5.648 2.732 4.180

40POPC H7R 5284 5.555 2.701 4.129

40POPC H7S 5285 5.681 2.647 4.243

40POPC C28 5286 5.758 2.757 4.075

40POPC H8R 5287 5.780 2.662 4.022

40POPC H8S 5288 5.852 2.786 4.128

40POPC C29 5289 5.716 2.867 3.981

40POPC H91 5290 5.729 2.969 4.018

40POPC C210 5291 5.660 2.851 3.860

40POPC H101 5292 5.635 2.941 3.802

40POPC C211 5293 5.626 2.720 3.795

40POPC H11R 5294 5.523 2.689 3.824

40POPC H11S 5295 5.695 2.639 3.825

40POPC C212 5296 5.630 2.735 3.642

40POPC H12R 5297 5.568 2.821 3.609

40POPC H12S 5298 5.588 2.644 3.595

40POPC C213 5299 5.773 2.755 3.593

40POPC H13R 5300 5.834 2.667 3.625

40POPC H13S 5301 5.817 2.845 3.640

40POPC C214 5302 5.781 2.767 3.441

40POPC H14R 5303 5.730 2.678 3.398

40POPC H14S 5304 5.888 2.766 3.413

40POPC C215 5305 5.718 2.893 3.380

40POPC H15R 5306 5.770 2.982 3.421

40POPC H15S 5307 5.612 2.900 3.410

40POPC C216 5308 5.729 2.895 3.226

40POPC H16R 5309 5.833 2.866 3.199

40POPC H16S 5310 5.717 2.998 3.188

40POPC C217 5311 5.627 2.804 3.155

40POPC H17R 5312 5.645 2.699 3.186

40POPC H17S 5313 5.644 2.808 3.045

40POPC C218 5314 5.481 2.838 3.178

40POPC H18R 5315 5.456 2.838 3.286

40POPC H18S 5316 5.415 2.763 3.127

40POPC H18T 5317 5.455 2.937 3.136

40POPC C33 5318 5.986 2.634 4.389

40POPC H3X 5319 6.010 2.729 4.336

40POPC H3Y 5320 5.884 2.604 4.357

40POPC C34 5321 6.089 2.530 4.346

40POPC H4X 5322 6.083 2.439 4.409

40POPC H4Y 5323 6.190 2.574 4.360

40POPC C35 5324 6.070 2.491 4.200

40POPC H5X 5325 6.062 2.584 4.139

40POPC H5Y 5326 5.974 2.436 4.188

40POPC C36 5327 6.187 2.408 4.147

40POPC H6X 5328 6.179 2.305 4.187

40POPC H6Y 5329 6.282 2.450 4.188

40POPC C37 5330 6.194 2.409 3.995

40POPC H7X 5331 6.272 2.337 3.962

40POPC H7Y 5332 6.226 2.510 3.959

40POPC C38 5333 6.059 2.376 3.930

40POPC H8X 5334 5.999 2.470 3.925

40POPC H8Y 5335 6.002 2.305 3.993

40POPC C39 5336 6.076 2.318 3.790

40POPC H9X 5337 6.047 2.211 3.787

40POPC H9Y 5338 6.184 2.323 3.763

40POPC C310 5339 6.000 2.396 3.683

40POPC H10X 5340 6.016 2.505 3.702

40POPC H10Y 5341 5.890 2.378 3.690

40POPC C311 5342 6.053 2.363 3.544

40POPC H11X 5343 6.023 2.260 3.514

40POPC H11Y 5344 6.164 2.365 3.549

40POPC C312 5345 6.008 2.465 3.440

40POPC H12X 5346 6.000 2.565 3.489

40POPC H12Y 5347 5.907 2.439 3.403

40POPC C313 5348 6.109 2.478 3.326

40POPC H13X 5349 6.101 2.388 3.261

40POPC H13Y 5350 6.212 2.479 3.366

40POPC C314 5351 6.085 2.606 3.243

40POPC H14X 5352 6.162 2.612 3.163

40POPC H14Y 5353 6.094 2.698 3.305

40POPC C315 5354 5.945 2.599 3.185

40POPC H15X 5355 5.868 2.632 3.258

40POPC H15Y 5356 5.927 2.490 3.175

40POPC C316 5357 5.920 2.665 3.051

40POPC H16X 5358 5.924 2.585 2.974

40POPC H16Y 5359 5.994 2.745 3.029

40POPC H16Z 5360 5.817 2.706 3.041

41POPC N 5361 1.712 5.649 4.825

41POPC C12 5362 1.713 5.718 4.961

41POPC H12A 5363 1.796 5.787 4.963

41POPC H12B 5364 1.730 5.644 5.037

41POPC C13 5365 1.587 5.570 4.810

41POPC H13A 5366 1.569 5.508 4.896

41POPC H13B 5367 1.503 5.637 4.804

41POPC H13C 5368 1.587 5.511 4.719

41POPC C14 5369 1.719 5.750 4.714

41POPC H14A 5370 1.638 5.820 4.720

41POPC H14B 5371 1.810 5.808 4.718

41POPC H14C 5372 1.714 5.703 4.616

41POPC C15 5373 1.832 5.560 4.812

41POPC H15A 5374 1.829 5.481 4.885

41POPC H15B 5375 1.923 5.617 4.825

41POPC H15C 5376 1.835 5.513 4.715

41POPC C11 5377 1.583 5.792 5.003

41POPC H11A 5378 1.553 5.857 4.919

41POPC H11B 5379 1.605 5.858 5.090

41POPC P 5380 1.328 5.732 5.010

41POPC O13 5381 1.283 5.842 5.097

41POPC O14 5382 1.248 5.608 5.016

41POPC O12 5383 1.480 5.699 5.040

41POPC O11 5384 1.332 5.785 4.861

41POPC C1 5385 1.225 5.761 4.769

41POPC HA 5386 1.155 5.847 4.767

41POPC HB 5387 1.166 5.670 4.795

41POPC C2 5388 1.280 5.734 4.627

41POPC HS 5389 1.193 5.719 4.560

41POPC O21 5390 1.369 5.623 4.634

41POPC C21 5391 1.332 5.527 4.555

41POPC O22 5392 1.218 5.487 4.539

41POPC C22 5393 1.457 5.486 4.480

41POPC H2R 5394 1.521 5.428 4.550

41POPC H2S 5395 1.514 5.576 4.448

41POPC C3 5396 1.363 5.852 4.569

41POPC HX 5397 1.423 5.889 4.655

41POPC HY 5398 1.291 5.930 4.539

41POPC O31 5399 1.456 5.822 4.462

41POPC C31 5400 1.395 5.797 4.347

41POPC O32 5401 1.276 5.776 4.337

41POPC C32 5402 1.494 5.802 4.230

41POPC H2X 5403 1.564 5.716 4.241

41POPC H2Y 5404 1.552 5.896 4.231

41POPC C23 5405 1.422 5.399 4.360

41POPC H3R 5406 1.350 5.322 4.396

41POPC H3S 5407 1.514 5.348 4.324

41POPC C24 5408 1.354 5.466 4.241

41POPC H4R 5409 1.433 5.504 4.172

41POPC H4S 5410 1.293 5.552 4.276

41POPC C25 5411 1.256 5.372 4.167

41POPC H5R 5412 1.155 5.398 4.205

41POPC H5S 5413 1.271 5.267 4.199

41POPC C26 5414 1.258 5.382 4.013

41POPC H6R 5415 1.172 5.325 3.972

41POPC H6S 5416 1.350 5.334 3.974

41POPC C27 5417 1.254 5.530 3.972

41POPC H7R 5418 1.360 5.561 3.958

41POPC H7S 5419 1.209 5.584 4.058

41POPC C28 5420 1.173 5.581 3.854

41POPC H8R 5421 1.186 5.692 3.853

41POPC H8S 5422 1.064 5.565 3.870

41POPC C29 5423 1.218 5.525 3.724

41POPC H91 5424 1.285 5.438 3.728

41POPC C210 5425 1.182 5.577 3.607

41POPC H101 5426 1.216 5.524 3.517

41POPC C211 5427 1.088 5.694 3.585

41POPC H11R 5428 1.068 5.752 3.678

41POPC H11S 5429 0.989 5.655 3.552

41POPC C212 5430 1.129 5.790 3.472

41POPC H12R 5431 1.213 5.854 3.507

41POPC H12S 5432 1.043 5.856 3.447

41POPC C213 5433 1.171 5.708 3.351

41POPC H13R 5434 1.097 5.626 3.338

41POPC H13S 5435 1.269 5.663 3.378

41POPC C214 5436 1.188 5.775 3.216

41POPC H14R 5437 1.256 5.862 3.224

41POPC H14S 5438 1.089 5.810 3.180

41POPC C215 5439 1.246 5.669 3.120

41POPC H15R 5440 1.244 5.707 3.015

41POPC H15S 5441 1.182 5.579 3.121

41POPC C216 5442 1.391 5.631 3.153

41POPC H16R 5443 1.394 5.571 3.246

41POPC H16S 5444 1.449 5.724 3.171

41POPC C217 5445 1.462 5.551 3.043

41POPC H17R 5446 1.519 5.621 2.978

41POPC H17S 5447 1.385 5.502 2.980

41POPC C218 5448 1.556 5.444 3.100

41POPC H18R 5449 1.632 5.489 3.167

41POPC H18S 5450 1.607 5.388 3.019

41POPC H18T 5451 1.497 5.370 3.160

41POPC C33 5452 1.416 5.787 4.098

41POPC H3X 5453 1.332 5.859 4.086

41POPC H3Y 5454 1.364 5.689 4.110

41POPC C34 5455 1.492 5.791 3.966

41POPC H4X 5456 1.530 5.893 3.945

41POPC H4Y 5457 1.417 5.769 3.887

41POPC C35 5458 1.606 5.690 3.958

41POPC H5X 5459 1.571 5.591 3.994

41POPC H5Y 5460 1.688 5.724 4.025

41POPC C36 5461 1.659 5.677 3.815

41POPC H6X 5462 1.748 5.611 3.816

41POPC H6Y 5463 1.694 5.777 3.782

41POPC C37 5464 1.557 5.621 3.715

41POPC H7X 5465 1.476 5.695 3.696

41POPC H7Y 5466 1.509 5.530 3.759

41POPC C38 5467 1.619 5.583 3.580

41POPC H8X 5468 1.540 5.542 3.514

41POPC H8Y 5469 1.694 5.503 3.595

41POPC C39 5470 1.688 5.701 3.511

41POPC H9X 5471 1.794 5.677 3.489

41POPC H9Y 5472 1.687 5.789 3.578

41POPC C310 5473 1.616 5.742 3.384

41POPC H10X 5474 1.507 5.750 3.406

41POPC H10Y 5475 1.628 5.665 3.305

41POPC C311 5476 1.666 5.877 3.335

41POPC H11X 5477 1.763 5.867 3.283

41POPC H11Y 5478 1.681 5.943 3.423

41POPC C312 5479 1.564 5.945 3.244

41POPC H12X 5480 1.461 5.928 3.281

41POPC H12Y 5481 1.573 5.897 3.144

41POPC C313 5482 1.588 6.095 3.237

41POPC H13X 5483 1.694 6.111 3.208

41POPC H13Y 5484 1.573 6.138 3.338

41POPC C314 5485 1.497 6.168 3.138

41POPC H14X 5486 1.529 6.274 3.129

41POPC H14Y 5487 1.392 6.166 3.174

41POPC C315 5488 1.498 6.103 3.001

41POPC H15X 5489 1.427 6.156 2.935

41POPC H15Y 5490 1.460 5.999 3.012

41POPC C316 5491 1.637 6.098 2.942

41POPC H16X 5492 1.634 6.055 2.839

41POPC H16Y 5493 1.706 6.035 3.002

41POPC H16Z 5494 1.678 6.201 2.936

42POPC N 5495 6.047 3.487 5.142

42POPC C12 5496 6.098 3.590 5.044

42POPC H12A 5497 6.205 3.585 5.038

42POPC H12B 5498 6.074 3.690 5.080

42POPC C13 5499 6.103 3.353 5.111

42POPC H13A 5500 6.068 3.278 5.180

42POPC H13B 5501 6.070 3.327 5.011

42POPC H13C 5502 6.211 3.355 5.108

42POPC C14 5503 6.084 3.526 5.281

42POPC H14A 5504 6.052 3.627 5.302

42POPC H14B 5505 6.035 3.461 5.352

42POPC H14C 5506 6.191 3.521 5.296

42POPC C15 5507 5.898 3.475 5.129

42POPC H15A 5508 5.873 3.438 5.030

42POPC H15B 5509 5.848 3.570 5.142

42POPC H15C 5510 5.857 3.405 5.201

42POPC C11 5511 6.048 3.583 4.899

42POPC H11A 5512 6.088 3.668 4.840

42POPC H11B 5513 5.938 3.592 4.895

42POPC P 5514 6.006 3.376 4.755

42POPC O13 5515 5.970 3.459 4.638

42POPC O14 5516 5.902 3.320 4.844

42POPC O12 5517 6.100 3.463 4.845

42POPC O11 5518 6.110 3.264 4.723

42POPC C1 5519 6.164 3.189 4.832

42POPC HA 5520 6.084 3.139 4.890

42POPC HB 5521 6.222 3.255 4.900

42POPC C2 5522 6.259 3.078 4.779

42POPC HS 5523 6.306 3.029 4.867

42POPC O21 5524 6.361 3.128 4.693

42POPC C21 5525 0.019 3.239 4.733

42POPC O22 5526 0.054 3.263 4.847

42POPC C22 5527 0.040 3.325 4.609

42POPC H2R 5528 0.031 3.261 4.518

42POPC H2S 5529 0.144 3.364 4.616

42POPC C3 5530 6.181 2.974 4.698

42POPC HX 5531 6.114 2.921 4.768

42POPC HY 5532 6.254 2.900 4.659

42POPC O31 5533 6.104 3.043 4.596

42POPC C31 5534 6.118 2.998 4.473

42POPC O32 5535 6.162 2.887 4.447

42POPC C32 5536 6.073 3.097 4.365

42POPC H2X 5537 6.165 3.146 4.326

42POPC H2Y 5538 6.008 3.174 4.412

42POPC C23 5539 6.342 3.442 4.594

42POPC H3R 5540 6.349 3.506 4.685

42POPC H3S 5541 6.237 3.406 4.584

42POPC C24 5542 6.375 3.529 4.472

42POPC H4R 5543 0.081 3.558 4.485

42POPC H4S 5544 6.312 3.620 4.478

42POPC C25 5545 6.359 3.467 4.332

42POPC H5R 5546 6.346 3.357 4.340

42POPC H5S 5547 0.053 3.489 4.276

42POPC C26 5548 6.245 3.526 4.247

42POPC H6R 5549 6.260 3.636 4.235

42POPC H6S 5550 6.152 3.511 4.305

42POPC C27 5551 6.225 3.460 4.107

42POPC H7R 5552 6.115 3.458 4.090

42POPC H7S 5553 6.263 3.356 4.112

42POPC C28 5554 6.289 3.531 3.985

42POPC H8R 5555 6.395 3.497 3.978

42POPC H8S 5556 6.295 3.638 4.015

42POPC C29 5557 6.222 3.529 3.848

42POPC H91 5558 6.166 3.621 3.833

42POPC C210 5559 6.226 3.443 3.742

42POPC H101 5560 6.168 3.475 3.654

42POPC C211 5561 6.288 3.306 3.720

42POPC H11R 5562 6.213 3.239 3.673

42POPC H11S 5563 6.306 3.262 3.821

42POPC C212 5564 0.015 3.285 3.632

42POPC H12R 5565 0.093 3.225 3.683

42POPC H12S 5566 0.055 3.387 3.614

42POPC C213 5567 0.008 3.215 3.497

42POPC H13R 5568 0.111 3.210 3.457

42POPC H13S 5569 6.347 3.275 3.426

42POPC C214 5570 6.355 3.073 3.506

42POPC H14R 5571 6.247 3.079 3.530

42POPC H14S 5572 0.006 3.018 3.588

42POPC C215 5573 6.373 2.996 3.375

42POPC H15R 5574 6.319 2.899 3.380

42POPC H15S 5575 0.081 2.972 3.365

42POPC C216 5576 6.327 3.074 3.251

42POPC H16R 5577 0.016 3.111 3.196

42POPC H16S 5578 6.267 3.163 3.279

42POPC C217 5579 6.238 2.990 3.158

42POPC H17R 5580 6.201 3.057 3.077

42POPC H17S 5581 6.150 2.954 3.215

42POPC C218 5582 6.313 2.872 3.097

42POPC H18R 5583 0.011 2.905 3.056

42POPC H18S 5584 6.251 2.830 3.015

42POPC H18T 5585 6.331 2.793 3.173

42POPC C33 5586 6.000 3.017 4.255

42POPC H3X 5587 5.909 2.972 4.300

42POPC H3Y 5588 6.064 2.931 4.226

42POPC C34 5589 5.956 3.087 4.124

42POPC H4X 5590 5.873 3.158 4.145

42POPC H4Y 5591 5.917 3.005 4.060

42POPC C35 5592 6.065 3.154 4.042

42POPC H5X 5593 6.157 3.091 4.044

42POPC H5Y 5594 6.092 3.248 4.096

42POPC C36 5595 6.040 3.185 3.892

42POPC H6X 5596 6.138 3.158 3.847

42POPC H6Y 5597 6.026 3.294 3.876

42POPC C37 5598 5.940 3.104 3.806

42POPC H7X 5599 5.839 3.149 3.813

42POPC H7Y 5600 5.935 3.002 3.849

42POPC C38 5601 5.983 3.088 3.658

42POPC H8X 5602 5.923 3.008 3.610

42POPC H8Y 5603 6.089 3.055 3.655

42POPC C39 5604 5.971 3.212 3.568

42POPC H9X 5605 6.034 3.295 3.605

42POPC H9Y 5606 5.866 3.247 3.579

42POPC C310 5607 5.998 3.193 3.417

42POPC H10X 5608 5.937 3.108 3.378

42POPC H10Y 5609 6.105 3.170 3.400

42POPC C311 5610 5.957 3.323 3.347

42POPC H11X 5611 6.017 3.408 3.385

42POPC H11Y 5612 5.853 3.334 3.382

42POPC C312 5613 5.942 3.332 3.195

42POPC H12X 5614 5.913 3.233 3.154

42POPC H12Y 5615 6.037 3.364 3.148

42POPC C313 5616 5.828 3.434 3.168

42POPC H13X 5617 5.847 3.525 3.228

42POPC H13Y 5618 5.735 3.389 3.208

42POPC C314 5619 5.799 3.475 3.024

42POPC H14X 5620 5.856 3.567 2.997

42POPC H14Y 5621 5.692 3.502 3.019

42POPC C315 5622 5.828 3.367 2.921

42POPC H15X 5623 5.796 3.271 2.967

42POPC H15Y 5624 5.938 3.360 2.904

42POPC C316 5625 5.755 3.387 2.788

42POPC H16X 5626 5.648 3.361 2.798

42POPC H16Y 5627 5.800 3.324 2.709

42POPC H16Z 5628 5.762 3.494 2.756

43POPC N 5629 4.939 5.372 4.951

43POPC C12 5630 5.090 5.372 4.972

43POPC H12A 5631 5.141 5.372 4.877

43POPC H12B 5632 5.122 5.281 5.021

43POPC C13 5633 4.903 5.474 4.847

43POPC H13A 5634 4.950 5.452 4.752

43POPC H13B 5635 4.796 5.483 4.834

43POPC H13C 5636 4.942 5.571 4.877

43POPC C14 5637 4.889 5.238 4.907

43POPC H14A 5638 4.933 5.209 4.813

43POPC H14B 5639 4.910 5.160 4.979

43POPC H14C 5640 4.782 5.239 4.894

43POPC C15 5641 4.871 5.410 5.079

43POPC H15A 5642 4.896 5.341 5.158

43POPC H15B 5643 4.764 5.414 5.066

43POPC H15C 5644 4.907 5.508 5.108

43POPC C11 5645 5.147 5.492 5.052

43POPC H11A 5646 5.257 5.479 5.058

43POPC H11B 5647 5.112 5.488 5.157

43POPC P 5648 5.206 5.688 4.893

43POPC O13 5649 5.297 5.590 4.831

43POPC O14 5650 5.267 5.799 4.969

43POPC O12 5651 5.108 5.616 4.994

43POPC O11 5652 5.108 5.748 4.785

43POPC C1 5653 5.066 5.884 4.800

43POPC HA 5654 5.150 5.951 4.828

43POPC HB 5655 4.988 5.892 4.879

43POPC C2 5656 5.011 5.940 4.666

43POPC HS 5657 4.964 6.039 4.684

43POPC O21 5658 4.914 5.858 4.603

43POPC C21 5659 4.819 5.795 4.665

43POPC O22 5660 4.796 5.790 4.785

43POPC C22 5661 4.731 5.728 4.559

43POPC H2R 5662 4.625 5.747 4.583

43POPC H2S 5663 4.751 5.619 4.568

43POPC C3 5664 5.127 5.962 4.564

43POPC HX 5665 5.189 5.870 4.563

43POPC HY 5666 5.192 6.044 4.601

43POPC O31 5667 5.076 5.999 4.434

43POPC C31 5668 5.092 5.909 4.337

43POPC O32 5669 5.166 5.813 4.339

43POPC C32 5670 4.999 5.947 4.222

43POPC H2X 5671 5.041 6.039 4.176

43POPC H2Y 5672 4.898 5.971 4.263

43POPC C23 5673 4.764 5.777 4.415

43POPC H3R 5674 4.874 5.772 4.399

43POPC H3S 5675 4.732 5.883 4.403

43POPC C24 5676 4.707 5.692 4.303

43POPC H4R 5677 4.743 5.730 4.205

43POPC H4S 5678 4.596 5.700 4.302

43POPC C25 5679 4.747 5.544 4.316

43POPC H5R 5680 4.708 5.493 4.226

43POPC H5S 5681 4.692 5.502 4.403

43POPC C26 5682 4.894 5.507 4.334

43POPC H6R 5683 4.931 5.549 4.430

43POPC H6S 5684 4.958 5.550 4.254

43POPC C27 5685 4.912 5.353 4.339

43POPC H7R 5686 4.833 5.307 4.401

43POPC H7S 5687 5.009 5.333 4.389

43POPC C28 5688 4.918 5.282 4.201

43POPC H8R 5689 4.945 5.176 4.223

43POPC H8S 5690 5.004 5.324 4.145

43POPC C29 5691 4.793 5.281 4.116

43POPC H91 5692 4.721 5.201 4.139

43POPC C210 5693 4.769 5.363 4.011

43POPC H101 5694 4.676 5.351 3.954

43POPC C211 5695 4.857 5.479 3.971

43POPC H11R 5696 4.920 5.511 4.056

43POPC H11S 5697 4.793 5.567 3.947

43POPC C212 5698 4.954 5.455 3.854

43POPC H12R 5699 5.002 5.355 3.861

43POPC H12S 5700 5.036 5.529 3.863

43POPC C213 5701 4.886 5.472 3.718

43POPC H13R 5702 4.831 5.569 3.718

43POPC H13S 5703 4.813 5.390 3.706

43POPC C214 5704 4.983 5.471 3.599

43POPC H14R 5705 5.026 5.369 3.590

43POPC H14S 5706 5.070 5.538 3.619

43POPC C215 5707 4.918 5.510 3.465

43POPC H15R 5708 4.841 5.431 3.451

43POPC H15S 5709 4.993 5.502 3.384

43POPC C216 5710 4.842 5.644 3.457

43POPC H16R 5711 4.883 5.714 3.533

43POPC H16S 5712 4.737 5.626 3.490

43POPC C217 5713 4.837 5.723 3.323

43POPC H17R 5714 4.921 5.796 3.324

43POPC H17S 5715 4.745 5.785 3.326

43POPC C218 5716 4.839 5.652 3.186

43POPC H18R 5717 4.928 5.587 3.175

43POPC H18S 5718 4.749 5.590 3.169

43POPC H18T 5719 4.842 5.727 3.104

43POPC C33 5720 4.989 5.843 4.110

43POPC H3X 5721 4.951 5.745 4.147

43POPC H3Y 5722 5.091 5.823 4.070

43POPC C34 5723 4.897 5.896 3.998

43POPC H4X 5724 4.910 6.006 3.991

43POPC H4Y 5725 4.791 5.876 4.026

43POPC C35 5726 4.928 5.836 3.861

43POPC H5X 5727 4.847 5.858 3.789

43POPC H5Y 5728 4.932 5.726 3.874

43POPC C36 5729 5.061 5.885 3.803

43POPC H6X 5730 5.137 5.898 3.883

43POPC H6Y 5731 5.048 5.984 3.754

43POPC C37 5732 5.116 5.781 3.705

43POPC H7X 5733 5.045 5.772 3.620

43POPC H7Y 5734 5.122 5.684 3.758

43POPC C38 5735 5.253 5.812 3.649

43POPC H8X 5736 5.324 5.823 3.734

43POPC H8Y 5737 5.246 5.907 3.592

43POPC C39 5738 5.309 5.708 3.553

43POPC H9X 5739 5.319 5.610 3.605

43POPC H9Y 5740 5.409 5.739 3.517

43POPC C310 5741 5.226 5.694 3.427

43POPC H10X 5742 5.125 5.656 3.452

43POPC H10Y 5743 5.276 5.616 3.366

43POPC C311 5744 5.208 5.821 3.343

43POPC H11X 5745 5.303 5.880 3.338

43POPC H11Y 5746 5.128 5.886 3.385

43POPC C312 5747 5.171 5.768 3.206

43POPC H12X 5748 5.088 5.696 3.222

43POPC H12Y 5749 5.259 5.707 3.175

43POPC C313 5750 5.134 5.861 3.091

43POPC H13X 5751 5.095 5.958 3.128

43POPC H13Y 5752 5.051 5.811 3.036

43POPC C314 5753 5.253 5.878 2.996

43POPC H14X 5754 5.323 5.792 3.004

43POPC H14Y 5755 5.312 5.963 3.038

43POPC C315 5756 5.226 5.897 2.847

43POPC H15X 5757 5.252 5.803 2.794

43POPC H15Y 5758 5.297 5.975 2.810

43POPC C316 5759 5.084 5.935 2.807

43POPC H16X 5760 5.046 6.020 2.868

43POPC H16Y 5761 5.015 5.849 2.821

43POPC H16Z 5762 5.083 5.964 2.700

44POPC N 5763 5.844 4.842 5.090

44POPC C12 5764 5.880 4.863 4.943

44POPC H12A 5765 5.877 4.768 4.892

44POPC H12B 5766 5.980 4.902 4.933

44POPC C13 5767 5.717 4.763 5.095

44POPC H13A 5768 5.677 4.756 5.195

44POPC H13B 5769 5.643 4.814 5.036

44POPC H13C 5770 5.728 4.664 5.053

44POPC C14 5771 5.945 4.765 5.168

44POPC H14A 5772 6.038 4.819 5.177

44POPC H14B 5773 5.909 4.748 5.268

44POPC H14C 5774 5.960 4.668 5.123

44POPC C15 5775 5.817 4.974 5.155

44POPC H15A 5776 5.904 5.038 5.159

44POPC H15B 5777 5.778 4.962 5.255

44POPC H15C 5778 5.741 5.027 5.099

44POPC C11 5779 5.786 4.957 4.863

44POPC H11A 5780 5.816 4.955 4.756

44POPC H11B 5781 5.799 5.062 4.898

44POPC P 5782 5.534 4.979 4.795

44POPC O13 5783 5.582 5.106 4.737

44POPC O14 5784 5.416 4.990 4.883

44POPC O12 5785 5.651 4.916 4.879

44POPC O11 5786 5.508 4.869 4.685

44POPC C1 5787 5.479 4.733 4.720

44POPC HA 5788 5.409 4.729 4.806

44POPC HB 5789 5.572 4.680 4.748

44POPC C2 5790 5.412 4.648 4.605

44POPC HS 5791 5.390 4.554 4.660

44POPC O21 5792 5.505 4.616 4.499

44POPC C21 5793 5.472 4.504 4.433

44POPC O22 5794 5.373 4.437 4.458

44POPC C22 5795 5.578 4.468 4.329

44POPC H2R 5796 5.669 4.430 4.381

44POPC H2S 5797 5.605 4.560 4.273

44POPC C3 5798 5.268 4.696 4.557

44POPC HX 5799 5.215 4.721 4.651

44POPC HY 5800 5.216 4.608 4.515

44POPC O31 5801 5.259 4.813 4.471

44POPC C31 5802 5.199 4.798 4.352

44POPC O32 5803 5.172 4.690 4.304

44POPC C32 5804 5.144 4.934 4.294

44POPC H2X 5805 5.104 4.988 4.383

44POPC H2Y 5806 5.058 4.908 4.229

44POPC C23 5807 5.527 4.360 4.230

44POPC H3R 5808 5.419 4.378 4.211

44POPC H3S 5809 5.533 4.259 4.277

44POPC C24 5810 5.596 4.355 4.092

44POPC H4R 5811 5.685 4.289 4.095

44POPC H4S 5812 5.631 4.456 4.063

44POPC C25 5813 5.497 4.305 3.986

44POPC H5R 5814 5.404 4.365 3.995

44POPC H5S 5815 5.469 4.200 4.012

44POPC C26 5816 5.541 4.309 3.839

44POPC H6R 5817 5.527 4.209 3.795

44POPC H6S 5818 5.648 4.336 3.832

44POPC C27 5819 5.456 4.409 3.758

44POPC H7R 5820 5.483 4.511 3.795

44POPC H7S 5821 5.348 4.394 3.779

44POPC C28 5822 5.477 4.398 3.607

44POPC H8R 5823 5.410 4.319 3.567

44POPC H8S 5824 5.580 4.361 3.586

44POPC C29 5825 5.450 4.530 3.540

44POPC H91 5826 5.347 4.541 3.501

44POPC C210 5827 5.534 4.634 3.526

44POPC H101 5828 5.497 4.721 3.470

44POPC C211 5829 5.679 4.648 3.567

44POPC H11R 5830 5.685 4.669 3.676

44POPC H11S 5831 5.722 4.737 3.515

44POPC C212 5832 5.776 4.532 3.534

44POPC H12R 5833 5.758 4.449 3.606

44POPC H12S 5834 5.881 4.565 3.551

44POPC C213 5835 5.761 4.480 3.391

44POPC H13R 5836 5.655 4.482 3.358

44POPC H13S 5837 5.791 4.373 3.390

44POPC C214 5838 5.848 4.553 3.289

44POPC H14R 5839 5.872 4.655 3.325

44POPC H14S 5840 5.792 4.563 3.193

44POPC C215 5841 5.975 4.471 3.265

44POPC H15R 5842 5.949 4.383 3.203

44POPC H15S 5843 6.010 4.433 3.363

44POPC C216 5844 6.091 4.546 3.202

44POPC H16R 5845 6.175 4.474 3.193

44POPC H16S 5846 6.121 4.628 3.271

44POPC C217 5847 6.063 4.607 3.065

44POPC H17R 5848 6.107 4.709 3.064

44POPC H17S 5849 5.953 4.623 3.056

44POPC C218 5850 6.120 4.529 2.945

44POPC H18R 5851 6.066 4.433 2.929

44POPC H18S 5852 6.227 4.506 2.959

44POPC H18T 5853 6.109 4.588 2.851

44POPC C33 5854 5.228 5.045 4.224

44POPC H3X 5855 5.336 5.036 4.249

44POPC H3Y 5856 5.194 5.145 4.261

44POPC C34 5857 5.219 5.049 4.072

44POPC H4X 5858 5.131 5.109 4.038

44POPC H4Y 5859 5.202 4.944 4.041

44POPC C35 5860 5.347 5.099 4.003

44POPC H5X 5861 5.434 5.094 4.071

44POPC H5Y 5862 5.335 5.205 3.971

44POPC C36 5863 5.374 5.008 3.884

44POPC H6X 5864 5.278 5.000 3.830

44POPC H6Y 5865 5.402 4.907 3.922

44POPC C37 5866 5.482 5.049 3.785

44POPC H7X 5867 5.581 5.011 3.817

44POPC H7Y 5868 5.487 5.159 3.773

44POPC C38 5869 5.439 4.985 3.654

44POPC H8X 5870 5.342 5.030 3.625

44POPC H8Y 5871 5.421 4.876 3.669

44POPC C39 5872 5.534 5.002 3.537

44POPC H9X 5873 5.620 4.931 3.548

44POPC H9Y 5874 5.574 5.105 3.533

44POPC C310 5875 5.454 4.972 3.412

44POPC H10X 5876 5.392 5.062 3.388

44POPC H10Y 5877 5.384 4.889 3.430

44POPC C311 5878 5.540 4.936 3.292

44POPC H11X 5879 5.601 4.846 3.310

44POPC H11Y 5880 5.609 5.021 3.272

44POPC C312 5881 5.447 4.914 3.174

44POPC H12X 5882 5.368 4.993 3.177

44POPC H12Y 5883 5.395 4.816 3.179

44POPC C313 5884 5.522 4.930 3.044

44POPC H13X 5885 5.578 4.837 3.020

44POPC H13Y 5886 5.596 5.012 3.058

44POPC C314 5887 5.430 4.970 2.930

44POPC H14X 5888 5.353 5.042 2.965

44POPC H14Y 5889 5.376 4.879 2.897

44POPC C315 5890 5.508 5.038 2.818

44POPC H15X 5891 5.521 5.142 2.853

44POPC H15Y 5892 5.448 5.041 2.724

44POPC C316 5893 5.647 4.979 2.790

44POPC H16X 5894 5.707 5.042 2.721

44POPC H16Y 5895 5.637 4.879 2.743

44POPC H16Z 5896 5.708 4.969 2.882

45POPC N 5897 0.147 2.898 5.104

45POPC C12 5898 0.011 2.860 5.048

45POPC H12A 5899 6.347 2.947 5.049

45POPC H12B 5900 6.366 2.786 5.114

45POPC C13 5901 0.222 2.983 5.004

45POPC H13A 5902 0.171 3.076 4.982

45POPC H13B 5903 0.322 3.004 5.036

45POPC H13C 5904 0.230 2.930 4.910

45POPC C14 5905 0.134 2.973 5.233

45POPC H14A 5906 0.072 2.919 5.304

45POPC H14B 5907 0.230 2.987 5.279

45POPC H14C 5908 0.090 3.071 5.217

45POPC C15 5909 0.224 2.772 5.127

45POPC H15A 5910 0.176 2.709 5.200

45POPC H15B 5911 0.325 2.791 5.159

45POPC H15C 5912 0.225 2.715 5.036

45POPC C11 5913 0.007 2.799 4.906

45POPC H11A 5914 0.049 2.872 4.833

45POPC H11B 5915 6.300 2.784 4.881

45POPC P 5916 0.081 2.610 4.751

45POPC O13 5917 6.351 2.636 4.686

45POPC O14 5918 0.121 2.468 4.766

45POPC O12 5919 0.077 2.674 4.897

45POPC O11 5920 0.197 2.692 4.683

45POPC C1 5921 0.323 2.709 4.751

45POPC HA 5922 0.347 2.625 4.820

45POPC HB 5923 0.318 2.801 4.813

45POPC C2 5924 0.445 2.724 4.654

45POPC HS 5925 0.529 2.770 4.710

45POPC O21 5926 0.414 2.804 4.540

45POPC C21 5927 0.369 2.923 4.565

45POPC O22 5928 0.374 2.985 4.670

45POPC C22 5929 0.314 2.978 4.434

45POPC H2R 5930 0.302 2.895 4.361

45POPC H2S 5931 0.390 3.048 4.394

45POPC C3 5932 0.494 2.588 4.599

45POPC HX 5933 0.537 2.532 4.685

45POPC HY 5934 0.576 2.604 4.526

45POPC O31 5935 0.384 2.513 4.545

45POPC C31 5936 0.361 2.528 4.416

45POPC O32 5937 0.433 2.584 4.335

45POPC C32 5938 0.226 2.462 4.383

45POPC H2X 5939 0.146 2.539 4.389

45POPC H2Y 5940 0.206 2.388 4.463

45POPC C23 5941 0.182 3.050 4.458

45POPC H3R 5942 0.172 3.136 4.388

45POPC H3S 5943 0.175 3.091 4.561

45POPC C24 5944 0.061 2.964 4.429

45POPC H4R 5945 6.371 3.022 4.459

45POPC H4S 5946 0.064 2.872 4.492

45POPC C25 5947 0.048 2.928 4.281

45POPC H5R 5948 0.145 2.935 4.227

45POPC H5S 5949 6.382 3.005 4.236

45POPC C26 5950 6.396 2.785 4.267

45POPC H6R 5951 6.314 2.769 4.341

45POPC H6S 5952 0.076 2.715 4.297

45POPC C27 5953 6.338 2.744 4.132

45POPC H7R 5954 6.271 2.658 4.152

45POPC H7S 5955 0.020 2.704 4.067

45POPC C28 5956 6.264 2.853 4.055

45POPC H8R 5957 6.334 2.902 3.983

45POPC H8S 5958 6.236 2.932 4.128

45POPC C29 5959 6.138 2.802 3.991

45POPC H91 5960 6.059 2.772 4.062

45POPC C210 5961 6.115 2.795 3.860

45POPC H101 5962 6.018 2.759 3.823

45POPC C211 5963 6.214 2.841 3.758

45POPC H11R 5964 6.317 2.806 3.783

45POPC H11S 5965 6.222 2.952 3.761

45POPC C212 5966 6.173 2.796 3.618

45POPC H12R 5967 6.197 2.877 3.546

45POPC H12S 5968 6.064 2.782 3.609

45POPC C213 5969 6.246 2.670 3.571

45POPC H13R 5970 6.222 2.662 3.463

45POPC H13S 5971 6.207 2.581 3.625

45POPC C214 5972 6.399 2.672 3.583

45POPC H14R 5973 0.026 2.653 3.689

45POPC H14S 5974 0.036 2.772 3.554

45POPC C215 5975 0.066 2.566 3.496

45POPC H15R 5976 0.015 2.468 3.509

45POPC H15S 5977 0.171 2.554 3.529

45POPC C216 5978 0.069 2.606 3.348

45POPC H16R 5979 0.121 2.704 3.340

45POPC H16S 5980 6.366 2.622 3.308

45POPC C217 5981 0.140 2.503 3.260

45POPC H17R 5982 0.066 2.438 3.210

45POPC H17S 5983 0.202 2.437 3.325

45POPC C218 5984 0.232 2.570 3.159

45POPC H18R 5985 0.312 2.623 3.214

45POPC H18S 5986 0.177 2.644 3.097

45POPC H18T 5987 0.279 2.495 3.091

45POPC C33 5988 0.224 2.389 4.248

45POPC H3X 5989 0.158 2.301 4.262

45POPC H3Y 5990 0.326 2.352 4.223

45POPC C34 5991 0.166 2.464 4.128

45POPC H4X 5992 0.070 2.511 4.158

45POPC H4Y 5993 0.145 2.391 4.047

45POPC C35 5994 0.257 2.573 4.071

45POPC H5X 5995 0.363 2.540 4.068

45POPC H5Y 5996 0.254 2.658 4.143

45POPC C36 5997 0.212 2.621 3.933

45POPC H6X 5998 0.105 2.595 3.914

45POPC H6Y 5999 0.270 2.568 3.855

45POPC C37 6000 0.226 2.773 3.921

45POPC H7X 6001 0.309 2.803 3.990

45POPC H7Y 6002 0.137 2.827 3.960

45POPC C38 6003 0.256 2.826 3.782

45POPC H8X 6004 0.290 2.932 3.792

45POPC H8Y 6005 0.162 2.829 3.723

45POPC C39 6006 0.363 2.747 3.706

45POPC H9X 6007 0.318 2.658 3.658

45POPC H9Y 6008 0.442 2.709 3.774

45POPC C310 6009 0.428 2.835 3.601

45POPC H10X 6010 0.519 2.786 3.560

45POPC H10Y 6011 0.458 2.932 3.645

45POPC C311 6012 0.330 2.857 3.487

45POPC H11X 6013 0.228 2.882 3.524

45POPC H11Y 6014 0.323 2.762 3.430

45POPC C312 6015 0.374 2.970 3.396

45POPC H12X 6016 0.485 2.980 3.397

45POPC H12Y 6017 0.332 3.066 3.432

45POPC C313 6018 0.330 2.939 3.255

45POPC H13X 6019 0.225 2.904 3.250

45POPC H13Y 6020 0.391 2.854 3.219

45POPC C314 6021 0.348 3.060 3.166

45POPC H14X 6022 0.397 3.143 3.221

45POPC H14Y 6023 0.247 3.095 3.135

45POPC C315 6024 0.432 3.022 3.045

45POPC H15X 6025 0.530 2.981 3.078

45POPC H15Y 6026 0.454 3.115 2.987

45POPC C316 6027 0.359 2.922 2.958

45POPC H16X 6028 0.255 2.957 2.942

45POPC H16Y 6029 0.351 2.822 3.005

45POPC H16Z 6030 0.407 2.910 2.858

46POPC N 6031 4.503 5.988 5.115

46POPC C12 6032 4.541 5.900 4.997

46POPC H12A 6033 4.469 5.820 4.989

46POPC H12B 6034 4.637 5.855 5.017

46POPC C13 6035 4.592 6.108 5.125

46POPC H13A 6036 4.696 6.079 5.134

46POPC H13B 6037 4.581 6.169 5.037

46POPC H13C 6038 4.564 6.169 5.209

46POPC C14 6039 4.363 6.039 5.099

46POPC H14A 6040 4.332 6.097 5.184

46POPC H14B 6041 4.360 6.103 5.012

46POPC H14C 6042 4.293 5.958 5.080

46POPC C15 6043 4.513 5.912 5.243

46POPC H15A 6044 4.488 5.977 5.325

46POPC H15B 6045 4.614 5.877 5.258

46POPC H15C 6046 4.446 5.828 5.245

46POPC C11 6047 4.550 5.963 4.855

46POPC H11A 6048 4.571 5.881 4.782

46POPC H11B 6049 4.639 6.029 4.853

46POPC P 6050 4.436 6.180 4.778

46POPC O13 6051 4.517 6.193 4.656

46POPC O14 6052 4.478 6.254 4.899

46POPC O12 6053 4.427 6.026 4.815

46POPC O11 6054 4.285 6.220 4.754

46POPC C1 6055 4.199 6.138 4.676

46POPC HA 6056 4.096 6.153 4.714

46POPC HB 6057 4.219 6.029 4.692

46POPC C2 6058 4.202 6.178 4.526

46POPC HS 6059 4.099 6.165 4.487

46POPC O21 6060 4.290 6.096 4.448

46POPC C21 6061 4.253 5.972 4.431

46POPC O22 6062 4.163 5.914 4.490

46POPC C22 6063 4.338 5.910 4.323

46POPC H2R 6064 4.288 5.813 4.299

46POPC H2S 6065 4.441 5.892 4.362

46POPC C3 6066 4.237 6.329 4.504

46POPC HX 6067 4.212 6.386 4.596

46POPC HY 6068 4.172 6.368 4.424

46POPC O31 6069 4.376 6.353 4.478

46POPC C31 6070 4.409 6.342 4.351

46POPC O32 6071 4.332 6.327 4.258

46POPC C32 6072 4.559 6.363 4.336

46POPC H2X 6073 4.589 6.302 4.248

46POPC H2Y 6074 4.610 6.322 4.426

46POPC C23 6075 4.342 5.997 4.196

46POPC H3R 6076 4.404 5.949 4.118

46POPC H3S 6077 4.390 6.095 4.218

46POPC C24 6078 4.201 6.020 4.141

46POPC H4R 6079 4.133 6.060 4.219

46POPC H4S 6080 4.160 5.923 4.106

46POPC C25 6081 4.199 6.119 4.026

46POPC H5R 6082 4.277 6.089 3.952

46POPC H5S 6083 4.223 6.221 4.062

46POPC C26 6084 4.061 6.118 3.960

46POPC H6R 6085 3.985 6.135 4.039

46POPC H6S 6086 4.046 6.017 3.916

46POPC C27 6087 4.039 6.221 3.851

46POPC H7R 6088 4.007 6.319 3.893

46POPC H7S 6089 3.949 6.191 3.794

46POPC C28 6090 4.161 6.246 3.763

46POPC H8R 6091 4.257 6.248 3.820

46POPC H8S 6092 4.155 6.347 3.717

46POPC C29 6093 4.160 6.139 3.659

46POPC H91 6094 4.221 6.049 3.677

46POPC C210 6095 4.075 6.145 3.557

46POPC H101 6096 4.075 6.068 3.479

46POPC C211 6097 3.982 6.262 3.538

46POPC H11R 6098 3.969 6.327 3.627

46POPC H11S 6099 3.877 6.230 3.521

46POPC C212 6100 4.029 6.354 3.429

46POPC H12R 6101 4.097 0.024 3.481

46POPC H12S 6102 3.941 0.011 3.391

46POPC C213 6103 4.100 6.295 3.307

46POPC H13R 6104 4.026 6.244 3.241

46POPC H13S 6105 4.181 6.224 3.333

46POPC C214 6106 4.156 0.008 3.223

46POPC H14R 6107 4.071 0.076 3.200

46POPC H14S 6108 4.199 6.365 3.131

46POPC C215 6109 4.266 0.088 3.288

46POPC H15R 6110 4.351 0.015 3.288

46POPC H15S 6111 4.246 0.114 3.394

46POPC C216 6112 4.296 0.219 3.215

46POPC H16R 6113 4.361 0.280 3.282

46POPC H16S 6114 4.201 0.275 3.204

46POPC C217 6115 4.363 0.209 3.077

46POPC H17R 6116 4.306 0.147 3.004

46POPC H17S 6117 4.459 0.153 3.082

46POPC C218 6118 4.387 0.350 3.022

46POPC H18R 6119 4.424 0.417 3.102

46POPC H18S 6120 4.292 0.392 2.983

46POPC H18T 6121 4.464 0.348 2.941

46POPC C33 6122 4.588 0.114 4.321

46POPC H3X 6123 4.627 0.153 4.418

46POPC H3Y 6124 4.492 0.168 4.308

46POPC C34 6125 4.678 0.164 4.208

46POPC H4X 6126 4.748 0.237 4.255

46POPC H4Y 6127 4.621 0.223 4.133

46POPC C35 6128 4.763 0.060 4.139

46POPC H5X 6129 4.792 6.384 4.215

46POPC H5Y 6130 4.854 0.108 4.098

46POPC C36 6131 4.697 6.390 4.023

46POPC H6X 6132 4.692 0.061 3.937

46POPC H6Y 6133 4.594 6.360 4.049

46POPC C37 6134 4.782 6.268 3.986

46POPC H7X 6135 4.796 6.205 4.077

46POPC H7Y 6136 4.882 6.304 3.953

46POPC C38 6137 4.720 6.181 3.878

46POPC H8X 6138 4.798 6.113 3.837

46POPC H8Y 6139 4.685 6.247 3.796

46POPC C39 6140 4.602 6.099 3.930

46POPC H9X 6141 4.516 6.166 3.951

46POPC H9Y 6142 4.631 6.051 4.026

46POPC C310 6143 4.560 5.989 3.832

46POPC H10X 6144 4.482 5.927 3.881

46POPC H10Y 6145 4.647 5.924 3.811

46POPC C311 6146 4.504 6.040 3.699

46POPC H11X 6147 4.580 6.101 3.644

46POPC H11Y 6148 4.420 6.109 3.722

46POPC C312 6149 4.456 5.923 3.611

46POPC H12X 6150 4.349 5.935 3.584

46POPC H12Y 6151 4.463 5.830 3.671

46POPC C313 6152 4.536 5.905 3.482

46POPC H13X 6153 4.552 5.796 3.464

46POPC H13Y 6154 4.635 5.953 3.490

46POPC C314 6155 4.459 5.963 3.363

46POPC H14X 6156 4.423 6.063 3.392

46POPC H14Y 6157 4.366 5.904 3.347

46POPC C315 6158 4.542 5.966 3.233

46POPC H15X 6159 4.573 5.862 3.209

46POPC H15Y 6160 4.636 6.023 3.252

46POPC C316 6161 4.471 6.026 3.112

46POPC H16X 6162 4.374 5.975 3.093

46POPC H16Y 6163 4.534 6.014 3.021

46POPC H16Z 6164 4.450 6.134 3.126

47POPC N 6165 2.868 6.371 5.006

47POPC C12 6166 2.997 6.313 5.059

47POPC H12A 6167 2.975 6.227 5.121

47POPC H12B 6168 3.044 6.386 5.123

47POPC C13 6169 2.817 6.288 4.893

47POPC H13A 6170 2.802 6.185 4.922

47POPC H13B 6171 2.727 6.331 4.852

47POPC H13C 6172 2.890 6.288 4.813

47POPC C14 6173 2.767 6.389 5.115

47POPC H14A 6174 2.805 0.057 5.189

47POPC H14B 6175 2.678 0.034 5.074

47POPC H14C 6176 2.742 6.296 5.164

47POPC C15 6177 2.889 0.107 4.947

47POPC H15A 6178 2.959 0.099 4.865

47POPC H15B 6179 2.936 0.174 5.018

47POPC H15C 6180 2.797 0.152 4.914

47POPC C11 6181 3.102 6.269 4.954

47POPC H11A 6182 3.061 6.187 4.890

47POPC H11B 6183 3.192 6.227 5.004

47POPC P 6184 3.234 0.089 4.919

47POPC O13 6185 3.335 0.030 5.008

47POPC O14 6186 3.156 0.201 4.977

47POPC O12 6187 3.134 6.377 4.869

47POPC O11 6188 3.296 0.139 4.783

47POPC C1 6189 3.217 0.199 4.679

47POPC HA 6190 3.285 0.237 4.600

47POPC HB 6191 3.164 0.289 4.718

47POPC C2 6192 3.112 0.109 4.605

47POPC HS 6193 3.022 0.121 4.668

47POPC O21 6194 3.143 6.368 4.587

47POPC C21 6195 3.032 6.294 4.569

47POPC O22 6196 2.919 6.332 4.596

47POPC C22 6197 3.061 6.155 4.513

47POPC H2R 6198 2.972 6.091 4.534

47POPC H2S 6199 3.147 6.115 4.572

47POPC C3 6200 3.080 0.167 4.465

47POPC HX 6201 3.056 0.275 4.476

47POPC HY 6202 2.987 0.114 4.436

47POPC O31 6203 3.186 0.151 4.368

47POPC C31 6204 3.157 0.080 4.258

47POPC O32 6205 3.049 0.036 4.229

47POPC C32 6206 3.287 0.054 4.182

47POPC H2X 6207 3.337 6.376 4.242

47POPC H2Y 6208 3.344 0.150 4.184

47POPC C23 6209 3.095 6.148 4.362

47POPC H3R 6210 3.152 6.053 4.348

47POPC H3S 6211 3.166 6.229 4.333

47POPC C24 6212 2.978 6.137 4.262

47POPC H4R 6213 2.919 6.231 4.258

47POPC H4S 6214 2.910 6.056 4.296

47POPC C25 6215 3.026 6.097 4.120

47POPC H5R 6216 2.992 5.994 4.095

47POPC H5S 6217 3.137 6.093 4.125

47POPC C26 6218 2.993 6.193 4.004

47POPC H6R 6219 3.070 6.175 3.927

47POPC H6S 6220 3.002 6.299 4.036

47POPC C27 6221 2.857 6.176 3.933

47POPC H7R 6222 2.780 6.234 3.989

47POPC H7S 6223 2.828 6.069 3.937

47POPC C28 6224 2.866 6.224 3.786

47POPC H8R 6225 2.947 6.167 3.737

47POPC H8S 6226 2.897 6.330 3.786

47POPC C29 6227 2.736 6.209 3.710

47POPC H91 6228 2.647 6.245 3.764

47POPC C210 6229 2.721 6.158 3.586

47POPC H101 6230 2.620 6.154 3.542

47POPC C211 6231 2.831 6.106 3.498

47POPC H11R 6232 2.898 6.037 3.553

47POPC H11S 6233 2.895 6.191 3.467

47POPC C212 6234 2.782 6.034 3.370

47POPC H12R 6235 2.713 6.101 3.314

47POPC H12S 6236 2.725 5.942 3.396

47POPC C213 6237 2.897 5.995 3.275

47POPC H13R 6238 2.857 5.936 3.190

47POPC H13S 6239 2.965 5.923 3.326

47POPC C214 6240 2.973 6.119 3.225

47POPC H14R 6241 3.058 6.137 3.295

47POPC H14S 6242 2.904 6.206 3.230

47POPC C215 6243 3.030 6.117 3.083

47POPC H15R 6244 3.074 6.217 3.062

47POPC H15S 6245 2.947 6.105 3.009

47POPC C216 6246 3.137 6.011 3.063

47POPC H16R 6247 3.140 5.949 3.155

47POPC H16S 6248 3.236 6.061 3.058

47POPC C217 6249 3.118 5.913 2.947

47POPC H17R 6250 3.068 5.821 2.984

47POPC H17S 6251 3.221 5.885 2.917

47POPC C218 6252 3.045 5.960 2.820

47POPC H18R 6253 3.049 6.071 2.809

47POPC H18S 6254 2.939 5.926 2.821

47POPC H18T 6255 3.092 5.915 2.731

47POPC C33 6256 3.292 6.395 4.039

47POPC H3X 6257 3.299 0.075 3.963

47POPC H3Y 6258 3.199 6.338 4.018

47POPC C34 6259 3.408 6.293 4.027

47POPC H4X 6260 3.358 6.194 4.036

47POPC H4Y 6261 3.481 6.295 4.111

47POPC C35 6262 3.486 6.294 3.896

47POPC H5X 6263 3.563 6.214 3.899

47POPC H5Y 6264 3.544 6.387 3.880

47POPC C36 6265 3.392 6.264 3.780

47POPC H6X 6266 3.292 6.234 3.819

47POPC H6Y 6267 3.435 6.178 3.724

47POPC C37 6268 3.377 6.375 3.678

47POPC H7X 6269 3.453 6.356 3.600

47POPC H7Y 6270 3.397 0.075 3.722

47POPC C38 6271 3.241 6.378 3.611

47POPC H8X 6272 3.193 0.074 3.640

47POPC H8Y 6273 3.174 6.298 3.650

47POPC C39 6274 3.259 6.362 3.460

47POPC H9X 6275 3.297 6.260 3.439

47POPC H9Y 6276 3.331 0.037 3.421

47POPC C310 6277 3.133 6.377 3.381

47POPC H10X 6278 3.066 6.296 3.415

47POPC H10Y 6279 3.149 6.363 3.272

47POPC C311 6280 3.071 0.111 3.412

47POPC H11X 6281 3.062 0.172 3.320

47POPC H11Y 6282 3.135 0.173 3.479

47POPC C312 6283 2.937 0.082 3.477

47POPC H12X 6284 2.913 0.167 3.544

47POPC H12Y 6285 2.940 6.394 3.544

47POPC C313 6286 2.839 0.059 3.363

47POPC H13X 6287 2.814 6.352 3.352

47POPC H13Y 6288 2.885 0.089 3.266

47POPC C314 6289 2.711 0.139 3.375

47POPC H14X 6290 2.639 0.079 3.435

47POPC H14Y 6291 2.672 0.146 3.271

47POPC C315 6292 2.725 0.279 3.438

47POPC H15X 6293 2.738 0.270 3.548

47POPC H15Y 6294 2.628 0.332 3.425

47POPC C316 6295 2.838 0.365 3.380

47POPC H16X 6296 2.829 0.471 3.413

47POPC H16Y 6297 2.836 0.361 3.269

47POPC H16Z 6298 2.939 0.331 3.411

48POPC N 6299 5.668 1.416 4.810

48POPC C12 6300 5.784 1.501 4.761

48POPC H12A 6301 5.752 1.604 4.754

48POPC H12B 6302 5.811 1.471 4.661

48POPC C13 6303 5.612 1.472 4.937

48POPC H13A 6304 5.566 1.568 4.921

48POPC H13B 6305 5.538 1.406 4.982

48POPC H13C 6306 5.691 1.486 5.008

48POPC C14 6307 5.557 1.417 4.708

48POPC H14A 6308 5.592 1.380 4.613

48POPC H14B 6309 5.475 1.356 4.741

48POPC H14C 6310 5.519 1.517 4.694

48POPC C15 6311 5.715 1.277 4.839

48POPC H15A 6312 5.789 1.279 4.917

48POPC H15B 6313 5.759 1.230 4.752

48POPC H15C 6314 5.634 1.214 4.875

48POPC C11 6315 5.913 1.500 4.848

48POPC H11A 6316 5.991 1.560 4.796

48POPC H11B 6317 5.953 1.397 4.854

48POPC P 6318 5.878 1.706 5.000

48POPC O13 6319 6.015 1.753 5.024

48POPC O14 6320 5.778 1.733 5.107

48POPC O12 6321 5.887 1.549 4.980

48POPC O11 6322 5.828 1.757 4.859

48POPC C1 6323 5.729 1.859 4.844

48POPC HA 6324 5.765 1.954 4.886

48POPC HB 6325 5.635 1.831 4.897

48POPC C2 6326 5.691 1.884 4.694

48POPC HS 6327 5.611 1.961 4.699

48POPC O21 6328 5.642 1.763 4.630

48POPC C21 6329 5.575 1.791 4.518

48POPC O22 6330 5.531 1.900 4.488

48POPC C22 6331 5.565 1.666 4.430

48POPC H2R 6332 5.461 1.629 4.438

48POPC H2S 6333 5.635 1.588 4.468

48POPC C3 6334 5.807 1.944 4.608

48POPC HX 6335 5.836 2.040 4.658

48POPC HY 6336 5.769 1.971 4.507

48POPC O31 6337 5.921 1.855 4.597

48POPC C31 6338 6.023 1.911 4.532

48POPC O32 6339 6.021 2.023 4.484

48POPC C32 6340 6.143 1.816 4.531

48POPC H2X 6341 6.127 1.736 4.606

48POPC H2Y 6342 6.231 1.876 4.563

48POPC C23 6343 5.597 1.699 4.282

48POPC H3R 6344 5.542 1.790 4.250

48POPC H3S 6345 5.562 1.615 4.218

48POPC C24 6346 5.748 1.718 4.257

48POPC H4R 6347 5.800 1.623 4.280

48POPC H4S 6348 5.787 1.796 4.325

48POPC C25 6349 5.783 1.759 4.113

48POPC H5R 6350 5.743 1.685 4.040

48POPC H5S 6351 5.894 1.759 4.104

48POPC C26 6352 5.734 1.900 4.074

48POPC H6R 6353 5.745 1.968 4.161

48POPC H6S 6354 5.626 1.895 4.047

48POPC C27 6355 5.815 1.959 3.957

48POPC H7R 6356 5.811 1.891 3.870

48POPC H7S 6357 5.921 1.962 3.990

48POPC C28 6358 5.776 2.104 3.918

48POPC H8R 6359 5.864 2.147 3.864

48POPC H8S 6360 5.769 2.161 4.013

48POPC C29 6361 5.654 2.134 3.833

48POPC H91 6362 5.600 2.223 3.868

48POPC C210 6363 5.604 2.068 3.726

48POPC H101 6364 5.511 2.106 3.682

48POPC C211 6365 5.658 1.945 3.659

48POPC H11R 6366 5.768 1.939 3.673

48POPC H11S 6367 5.643 1.953 3.549

48POPC C212 6368 5.593 1.815 3.710

48POPC H12R 6369 5.594 1.813 3.821

48POPC H12S 6370 5.654 1.730 3.675

48POPC C213 6371 5.451 1.784 3.659

48POPC H13R 6372 5.380 1.858 3.700

48POPC H13S 6373 5.422 1.684 3.698

48POPC C214 6374 5.440 1.781 3.506

48POPC H14R 6375 5.445 1.886 3.472

48POPC H14S 6376 5.341 1.739 3.479

48POPC C215 6377 5.548 1.703 3.430

48POPC H15R 6378 5.548 1.599 3.468

48POPC H15S 6379 5.648 1.748 3.450

48POPC C216 6380 5.527 1.700 3.277

48POPC H16R 6381 5.419 1.685 3.257

48POPC H16S 6382 5.581 1.613 3.234

48POPC C217 6383 5.576 1.826 3.203

48POPC H17R 6384 5.556 1.915 3.268

48POPC H17S 6385 5.515 1.838 3.111

48POPC C218 6386 5.723 1.826 3.162

48POPC H18R 6387 5.790 1.816 3.251

48POPC H18S 6388 5.750 1.920 3.109

48POPC H18T 6389 5.744 1.742 3.093

48POPC C33 6390 6.172 1.755 4.392

48POPC H3X 6391 6.189 1.835 4.317

48POPC H3Y 6392 6.083 1.696 4.360

48POPC C34 6393 6.295 1.663 4.395

48POPC H4X 6394 6.283 1.588 4.477

48POPC H4Y 6395 6.385 1.725 4.420

48POPC C35 6396 6.323 1.588 4.264

48POPC H5X 6397 0.013 1.525 4.277

48POPC H5Y 6398 6.345 1.662 4.184

48POPC C36 6399 6.206 1.498 4.220

48POPC H6X 6400 6.121 1.562 4.189

48POPC H6Y 6401 6.172 1.437 4.306

48POPC C37 6402 6.241 1.403 4.104

48POPC H7X 6403 6.149 1.349 4.073

48POPC H7Y 6404 6.313 1.327 4.142

48POPC C38 6405 6.304 1.471 3.981

48POPC H8X 6406 6.316 1.395 3.901

48POPC H8Y 6407 0.006 1.507 4.008

48POPC C39 6408 6.223 1.589 3.925

48POPC H9X 6409 6.279 1.629 3.837

48POPC H9Y 6410 6.215 1.671 3.999

48POPC C310 6411 6.083 1.549 3.878

48POPC H10X 6412 6.019 1.525 3.965

48POPC H10Y 6413 6.094 1.458 3.815

48POPC C311 6414 6.019 1.661 3.795

48POPC H11X 6415 6.088 1.686 3.712

48POPC H11Y 6416 6.009 1.752 3.859

48POPC C312 6417 5.882 1.627 3.735

48POPC H12X 6418 5.855 1.712 3.669

48POPC H12Y 6419 5.808 1.622 3.817

48POPC C313 6420 5.874 1.498 3.653

48POPC H13X 6421 5.767 1.473 3.636

48POPC H13Y 6422 5.917 1.413 3.711

48POPC C314 6423 5.943 1.505 3.516

48POPC H14X 6424 5.965 1.402 3.483

48POPC H14Y 6425 6.040 1.560 3.522

48POPC C315 6426 5.853 1.569 3.409

48POPC H15X 6427 5.846 1.678 3.427

48POPC H15Y 6428 5.751 1.525 3.420

48POPC C316 6429 5.897 1.543 3.265

48POPC H16X 6430 5.812 1.561 3.195

48POPC H16Y 6431 5.930 1.438 3.251

48POPC H16Z 6432 5.979 1.612 3.236

49POPC N 6433 0.809 5.603 4.984

49POPC C12 6434 0.820 5.452 4.975

49POPC H12A 6435 0.902 5.419 5.038

49POPC H12B 6436 0.845 5.424 4.874

49POPC C13 6437 0.708 5.649 4.884

49POPC H13A 6438 0.678 5.752 4.902

49POPC H13B 6439 0.747 5.639 4.784

49POPC H13C 6440 0.622 5.585 4.892

49POPC C14 6441 0.762 5.643 5.121

49POPC H14A 6442 0.834 5.614 5.196

49POPC H14B 6443 0.749 5.749 5.129

49POPC H14C 6444 0.668 5.595 5.143

49POPC C15 6445 0.942 5.666 4.958

49POPC H15A 6446 0.938 5.774 4.968

49POPC H15B 6447 0.980 5.642 4.860

49POPC H15C 6448 1.017 5.633 5.028

49POPC C11 6449 0.695 5.369 5.013

49POPC H11A 6450 0.658 5.394 5.114

49POPC H11B 6451 0.728 5.263 5.017

49POPC P 6452 0.497 5.268 4.877

49POPC O13 6453 0.382 5.262 4.970

49POPC O14 6454 0.582 5.147 4.867

49POPC O12 6455 0.590 5.390 4.918

49POPC O11 6456 0.452 5.314 4.732

49POPC C1 6457 0.549 5.362 4.637

49POPC HA 6458 0.558 5.290 4.553

49POPC HB 6459 0.651 5.371 4.680

49POPC C2 6460 0.507 5.500 4.580

49POPC HS 6461 0.453 5.553 4.661

49POPC O21 6462 0.419 5.475 4.470

49POPC C21 6463 0.377 5.575 4.397

49POPC O22 6464 0.385 5.694 4.424

49POPC C22 6465 0.312 5.515 4.272

49POPC H2R 6466 0.211 5.560 4.262

49POPC H2S 6467 0.304 5.406 4.288

49POPC C3 6468 0.629 5.588 4.539

49POPC HX 6469 0.708 5.578 4.617

49POPC HY 6470 0.596 5.694 4.538

49POPC O31 6471 0.683 5.545 4.414

49POPC C31 6472 0.721 5.639 4.329

49POPC O32 6473 0.732 5.758 4.354

49POPC C32 6474 0.745 5.573 4.194

49POPC H2X 6475 0.694 5.633 4.116

49POPC H2Y 6476 0.699 5.472 4.196

49POPC C23 6477 0.389 5.532 4.141

49POPC H3R 6478 0.497 5.546 4.161

49POPC H3S 6479 0.354 5.623 4.088

49POPC C24 6480 0.374 5.410 4.049

49POPC H4R 6481 0.275 5.361 4.061

49POPC H4S 6482 0.450 5.334 4.077

49POPC C25 6483 0.392 5.449 3.902

49POPC H5R 6484 0.302 5.503 3.867

49POPC H5S 6485 0.403 5.356 3.841

49POPC C26 6486 0.514 5.538 3.884

49POPC H6R 6487 0.597 5.493 3.942

49POPC H6S 6488 0.495 5.639 3.926

49POPC C27 6489 0.557 5.551 3.738

49POPC H7R 6490 0.567 5.449 3.696

49POPC H7S 6491 0.657 5.600 3.735

49POPC C28 6492 0.459 5.630 3.652

49POPC H8R 6493 0.361 5.579 3.658

49POPC H8S 6494 0.492 5.622 3.546

49POPC C29 6495 0.448 5.772 3.697

49POPC H91 6496 0.368 5.794 3.770

49POPC C210 6497 0.529 5.870 3.655

49POPC H101 6498 0.515 5.973 3.689

49POPC C211 6499 0.640 5.849 3.556

49POPC H11R 6500 0.668 5.742 3.545

49POPC H11S 6501 0.730 5.903 3.593

49POPC C212 6502 0.602 5.902 3.419

49POPC H12R 6503 0.691 5.909 3.352

49POPC H12S 6504 0.568 6.006 3.438

49POPC C213 6505 0.491 5.824 3.348

49POPC H13R 6506 0.441 5.894 3.278

49POPC H13S 6507 0.413 5.791 3.419

49POPC C214 6508 0.544 5.704 3.267

49POPC H14R 6509 0.487 5.612 3.295

49POPC H14S 6510 0.649 5.685 3.295

49POPC C215 6511 0.534 5.724 3.115

49POPC H15R 6512 0.575 5.635 3.063

49POPC H15S 6513 0.602 5.809 3.089

49POPC C216 6514 0.393 5.753 3.061

49POPC H16R 6515 0.404 5.785 2.955

49POPC H16S 6516 0.343 5.838 3.112

49POPC C217 6517 0.299 5.631 3.063

49POPC H17R 6518 0.358 5.539 3.046

49POPC H17S 6519 0.229 5.641 2.977

49POPC C218 6520 0.218 5.615 3.192

49POPC H18R 6521 0.170 5.711 3.219

49POPC H18S 6522 0.282 5.583 3.276

49POPC H18T 6523 0.138 5.539 3.177

49POPC C33 6524 0.894 5.560 4.161

49POPC H3X 6525 0.955 5.554 4.253

49POPC H3Y 6526 0.927 5.650 4.104

49POPC C34 6527 0.922 5.433 4.081

49POPC H4X 6528 0.923 5.343 4.145

49POPC H4Y 6529 1.026 5.448 4.043

49POPC C35 6530 0.837 5.409 3.957

49POPC H5X 6531 0.809 5.508 3.915

49POPC H5Y 6532 0.744 5.355 3.986

49POPC C36 6533 0.910 5.329 3.849

49POPC H6X 6534 0.936 5.228 3.888

49POPC H6Y 6535 1.009 5.374 3.827

49POPC C37 6536 0.832 5.323 3.718

49POPC H7X 6537 0.723 5.315 3.738

49POPC H7Y 6538 0.862 5.235 3.657

49POPC C38 6539 0.868 5.449 3.641

49POPC H8X 6540 0.975 5.437 3.612

49POPC H8Y 6541 0.863 5.537 3.709

49POPC C39 6542 0.788 5.482 3.516

49POPC H9X 6543 0.779 5.592 3.509

49POPC H9Y 6544 0.685 5.440 3.521

49POPC C310 6545 0.858 5.440 3.389

49POPC H10X 6546 0.959 5.397 3.410

49POPC H10Y 6547 0.872 5.527 3.322

49POPC C311 6548 0.772 5.337 3.320

49POPC H11X 6549 0.674 5.385 3.298

49POPC H11Y 6550 0.752 5.251 3.388

49POPC C312 6551 0.837 5.288 3.193

49POPC H12X 6552 0.912 5.211 3.220

49POPC H12Y 6553 0.891 5.371 3.142

49POPC C313 6554 0.728 5.235 3.103

49POPC H13X 6555 0.659 5.187 3.175

49POPC H13Y 6556 0.762 5.154 3.034

49POPC C314 6557 0.666 5.352 3.025

49POPC H14X 6558 0.738 5.385 2.948

49POPC H14Y 6559 0.653 5.437 3.095

49POPC C315 6560 0.529 5.324 2.963

49POPC H15X 6561 0.536 5.274 2.865

49POPC H15Y 6562 0.480 5.421 2.940

49POPC C316 6563 0.442 5.237 3.053

49POPC H16X 6564 0.358 5.192 2.996

49POPC H16Y 6565 0.405 5.292 3.142

49POPC H16Z 6566 0.498 5.146 3.083

50POPC N 6567 0.925 0.924 4.820

50POPC C12 6568 1.071 0.944 4.858

50POPC H12A 6569 1.108 1.035 4.815

50POPC H12B 6570 1.129 0.863 4.815

50POPC C13 6571 0.840 1.006 4.910

50POPC H13A 6572 0.737 1.005 4.879

50POPC H13B 6573 0.848 0.970 5.011

50POPC H13C 6574 0.872 1.109 4.911

50POPC C14 6575 0.902 0.961 4.676

50POPC H14A 6576 0.925 1.064 4.656

50POPC H14B 6577 0.961 0.902 4.607

50POPC H14C 6578 0.799 0.947 4.648

50POPC C15 6579 0.880 0.782 4.841

50POPC H15A 6580 0.941 0.711 4.787

50POPC H15B 6581 0.777 0.768 4.814

50POPC H15C 6582 0.891 0.755 4.945

50POPC C11 6583 1.106 0.946 5.010

50POPC H11A 6584 1.061 1.036 5.058

50POPC H11B 6585 1.216 0.956 5.022

50POPC P 6586 1.132 0.692 5.056

50POPC O13 6587 1.023 0.593 5.051

50POPC O14 6588 1.238 0.679 5.157

50POPC O12 6589 1.060 0.830 5.078

50POPC O11 6590 1.196 0.713 4.911

50POPC C1 6591 1.207 0.602 4.824

50POPC HA 6592 1.122 0.531 4.835

50POPC HB 6593 1.300 0.549 4.852

50POPC C2 6594 1.219 0.636 4.673

50POPC HS 6595 1.281 0.557 4.625

50POPC O21 6596 1.276 0.765 4.655

50POPC C21 6597 1.405 0.774 4.651

50POPC O22 6598 1.487 0.684 4.662

50POPC C22 6599 1.440 0.918 4.619

50POPC H2R 6600 1.548 0.926 4.598

50POPC H2S 6601 1.416 0.976 4.711

50POPC C3 6602 1.083 0.636 4.598

50POPC HX 6603 1.026 0.726 4.627

50POPC HY 6604 1.024 0.549 4.633

50POPC O31 6605 1.103 0.623 4.455

50POPC C31 6606 1.078 0.732 4.382

50POPC O32 6607 1.037 0.838 4.425

50POPC C32 6608 1.114 0.705 4.237

50POPC H2X 6609 1.207 0.644 4.235

50POPC H2Y 6610 1.134 0.802 4.188

50POPC C23 6611 1.357 0.974 4.501

50POPC H3R 6612 1.343 1.083 4.519

50POPC H3S 6613 1.254 0.933 4.497

50POPC C24 6614 1.425 0.958 4.365

50POPC H4R 6615 1.519 1.019 4.367

50POPC H4S 6616 1.357 1.003 4.290

50POPC C25 6617 1.463 0.817 4.319

50POPC H5R 6618 1.370 0.764 4.291

50POPC H5S 6619 1.512 0.759 4.400

50POPC C26 6620 1.559 0.821 4.198

50POPC H6R 6621 1.658 0.860 4.232

50POPC H6S 6622 1.520 0.889 4.120

50POPC C27 6623 1.577 0.685 4.129

50POPC H7R 6624 1.590 0.608 4.208

50POPC H7S 6625 1.670 0.687 4.068

50POPC C28 6626 1.459 0.653 4.035

50POPC H8R 6627 1.474 0.708 3.940

50POPC H8S 6628 1.365 0.693 4.079

50POPC C29 6629 1.445 0.505 4.015

50POPC H91 6630 1.451 0.446 4.108

50POPC C210 6631 1.430 0.440 3.899

50POPC H101 6632 1.424 0.330 3.902

50POPC C211 6633 1.417 0.498 3.760

50POPC H11R 6634 1.509 0.478 3.702

50POPC H11S 6635 1.400 0.607 3.759

50POPC C212 6636 1.298 0.427 3.692

50POPC H12R 6637 1.204 0.471 3.730

50POPC H12S 6638 1.299 0.320 3.718

50POPC C213 6639 1.297 0.433 3.538

50POPC H13R 6640 1.386 0.380 3.496

50POPC H13S 6641 1.304 0.540 3.509

50POPC C214 6642 1.170 0.369 3.481

50POPC H14R 6643 1.086 0.441 3.494

50POPC H14S 6644 1.147 0.282 3.546

50POPC C215 6645 1.171 0.304 3.341

50POPC H15R 6646 1.202 0.198 3.353

50POPC H15S 6647 1.247 0.347 3.271

50POPC C216 6648 1.032 0.307 3.276

50POPC H16R 6649 0.966 0.359 3.349

50POPC H16S 6650 0.994 0.202 3.269

50POPC C217 6651 1.008 0.381 3.143

50POPC H17R 6652 1.003 0.490 3.163

50POPC H17S 6653 0.907 0.354 3.106

50POPC C218 6654 1.103 0.355 3.026

50POPC H18R 6655 1.179 0.279 3.052

50POPC H18S 6656 1.155 0.450 2.997

50POPC H18T 6657 1.046 0.321 2.937

50POPC C33 6658 1.005 0.629 4.161

50POPC H3X 6659 0.909 0.685 4.162

50POPC H3Y 6660 0.986 0.531 4.211

50POPC C34 6661 1.047 0.601 4.017

50POPC H4X 6662 1.154 0.571 4.014

50POPC H4Y 6663 1.038 0.693 3.955

50POPC C35 6664 0.965 0.487 3.956

50POPC H5X 6665 0.974 0.400 4.024

50POPC H5Y 6666 1.010 0.458 3.859

50POPC C36 6667 0.817 0.521 3.938

50POPC H6X 6668 0.772 0.551 4.034

50POPC H6Y 6669 0.761 0.430 3.905

50POPC C37 6670 0.794 0.630 3.833

50POPC H7X 6671 0.832 0.726 3.874

50POPC H7Y 6672 0.686 0.644 3.814

50POPC C38 6673 0.861 0.600 3.698

50POPC H8X 6674 0.836 0.496 3.669

50POPC H8Y 6675 0.971 0.608 3.707

50POPC C39 6676 0.812 0.694 3.588

50POPC H9X 6677 0.702 0.683 3.580

50POPC H9Y 6678 0.857 0.667 3.490

50POPC C310 6679 0.841 0.841 3.620

50POPC H10X 6680 0.811 0.868 3.723

50POPC H10Y 6681 0.780 0.902 3.551

50POPC C311 6682 0.987 0.882 3.602

50POPC H11X 6683 1.035 0.814 3.528

50POPC H11Y 6684 1.043 0.872 3.697

50POPC C312 6685 0.990 1.026 3.551

50POPC H12X 6686 0.936 1.093 3.622

50POPC H12Y 6687 0.935 1.030 3.455

50POPC C313 6688 1.129 1.082 3.530

50POPC H13X 6689 1.181 1.101 3.627

50POPC H13Y 6690 1.116 1.178 3.477

50POPC C314 6691 1.218 0.996 3.442

50POPC H14X 6692 1.252 0.904 3.494

50POPC H14Y 6693 1.307 1.057 3.416

50POPC C315 6694 1.152 0.959 3.311

50POPC H15X 6695 1.082 1.039 3.278

50POPC H15Y 6696 1.086 0.870 3.323

50POPC C316 6697 1.260 0.936 3.207

50POPC H16X 6698 1.230 0.861 3.131

50POPC H16Y 6699 1.354 0.901 3.256

50POPC H16Z 6700 1.285 1.029 3.152

51POPC N 6701 4.029 0.274 4.867

51POPC C12 6702 3.997 0.284 5.014

51POPC H12A 6703 3.987 0.387 5.044

51POPC H12B 6704 4.080 0.245 5.071

51POPC C13 6705 3.907 0.300 4.784

51POPC H13A 6706 3.870 0.402 4.793

51POPC H13B 6707 3.926 0.282 4.679

51POPC H13C 6708 3.826 0.234 4.810

51POPC C14 6709 4.135 0.369 4.820

51POPC H14A 6710 4.097 0.470 4.818

51POPC H14B 6711 4.224 0.360 4.880

51POPC H14C 6712 4.159 0.344 4.717

51POPC C15 6713 4.079 0.136 4.841

51POPC H15A 6714 4.174 0.116 4.889

51POPC H15B 6715 4.089 0.119 4.735

51POPC H15C 6716 4.008 0.066 4.881

51POPC C11 6717 3.869 0.209 5.063

51POPC H11A 6718 3.783 0.265 5.020

51POPC H11B 6719 3.863 0.220 5.173

51POPC P 6720 3.726 6.399 5.029

51POPC O13 6721 3.636 0.087 4.953

51POPC O14 6722 3.695 6.370 5.171

51POPC O12 6723 3.869 0.070 5.027

51POPC O11 6724 3.753 6.262 4.953

51POPC C1 6725 3.841 6.246 4.842

51POPC HA 6726 3.825 6.145 4.798

51POPC HB 6727 3.947 6.251 4.877

51POPC C2 6728 3.820 6.350 4.728

51POPC HS 6729 3.838 0.048 4.776

51POPC O21 6730 3.687 6.339 4.676

51POPC C21 6731 3.643 0.057 4.636

51POPC O22 6732 3.705 0.162 4.640

51POPC C22 6733 3.504 0.046 4.583

51POPC H2R 6734 3.455 0.139 4.620

51POPC H2S 6735 3.452 6.359 4.627

51POPC C3 6736 3.924 6.337 4.613

51POPC HX 6737 3.930 6.229 4.587

51POPC HY 6738 4.023 6.363 4.656

51POPC O31 6739 3.894 0.009 4.491

51POPC C31 6740 3.962 0.122 4.468

51POPC O32 6741 4.039 0.175 4.546

51POPC C32 6742 3.945 0.167 4.323

51POPC H2X 6743 3.988 0.268 4.312

51POPC H2Y 6744 3.836 0.173 4.303

51POPC C23 6745 3.494 0.046 4.430

51POPC H3R 6746 3.384 0.040 4.416

51POPC H3S 6747 3.536 6.353 4.386

51POPC C24 6748 3.549 0.169 4.353

51POPC H4R 6749 3.630 0.214 4.414

51POPC H4S 6750 3.468 0.245 4.344

51POPC C25 6751 3.606 0.130 4.214

51POPC H5R 6752 3.529 0.073 4.158

51POPC H5S 6753 3.688 0.058 4.230

51POPC C26 6754 3.650 0.238 4.111

51POPC H6R 6755 3.702 0.183 4.030

51POPC H6S 6756 3.724 0.307 4.156

51POPC C27 6757 3.538 0.319 4.043

51POPC H7R 6758 3.566 0.426 4.044

51POPC H7S 6759 3.445 0.310 4.103

51POPC C28 6760 3.505 0.279 3.895

51POPC H8R 6761 3.405 0.324 3.875

51POPC H8S 6762 3.488 0.169 3.890

51POPC C29 6763 3.598 0.329 3.787

51POPC H91 6764 3.620 0.436 3.801

51POPC C210 6765 3.654 0.273 3.676

51POPC H101 6766 3.713 0.343 3.614

51POPC C211 6767 3.657 0.134 3.615

51POPC H11R 6768 3.608 0.059 3.681

51POPC H11S 6769 3.762 0.098 3.612

51POPC C212 6770 3.608 0.122 3.466

51POPC H12R 6771 3.644 0.210 3.408

51POPC H12S 6772 3.497 0.128 3.465

51POPC C213 6773 3.660 6.395 3.394

51POPC H13R 6774 3.699 6.332 3.477

51POPC H13S 6775 3.745 0.021 3.328

51POPC C214 6776 3.565 6.302 3.315

51POPC H14R 6777 3.478 6.274 3.377

51POPC H14S 6778 3.616 6.204 3.298

51POPC C215 6779 3.524 6.340 3.172

51POPC H15R 6780 3.563 0.041 3.149

51POPC H15S 6781 3.413 6.342 3.170

51POPC C216 6782 3.568 6.238 3.066

51POPC H16R 6783 3.495 6.154 3.061

51POPC H16S 6784 3.665 6.195 3.100

51POPC C217 6785 3.592 6.293 2.925

51POPC H17R 6786 3.521 6.375 2.901

51POPC H17S 6787 3.584 6.214 2.847

51POPC C218 6788 3.736 6.336 2.911

51POPC H18R 6789 3.757 0.000 2.999

51POPC H18S 6790 3.756 6.389 2.816

51POPC H18T 6791 3.803 6.248 2.917

51POPC C33 6792 4.014 0.068 4.228

51POPC H3X 6793 3.984 6.364 4.252

51POPC H3Y 6794 4.124 0.073 4.243

51POPC C34 6795 3.988 0.085 4.078

51POPC H4X 6796 3.893 0.037 4.050

51POPC H4Y 6797 4.072 0.027 4.035

51POPC C35 6798 3.997 0.224 4.012

51POPC H5X 6799 4.084 0.281 4.053

51POPC H5Y 6800 3.906 0.284 4.034

51POPC C36 6801 4.014 0.208 3.860

51POPC H6X 6802 3.915 0.178 3.817

51POPC H6Y 6803 4.084 0.124 3.840

51POPC C37 6804 4.070 0.332 3.788

51POPC H7X 6805 4.165 0.362 3.837

51POPC H7Y 6806 3.997 0.415 3.801

51POPC C38 6807 4.097 0.310 3.638

51POPC H8X 6808 4.148 0.213 3.622

51POPC H8Y 6809 4.163 0.389 3.598

51POPC C39 6810 3.964 0.309 3.563

51POPC H9X 6811 3.908 0.405 3.575

51POPC H9Y 6812 3.907 0.230 3.616

51POPC C310 6813 3.966 0.265 3.417

51POPC H10X 6814 3.877 0.199 3.407

51POPC H10Y 6815 4.058 0.207 3.394

51POPC C311 6816 3.948 0.372 3.310

51POPC H11X 6817 4.047 0.416 3.283

51POPC H11Y 6818 3.883 0.451 3.353

51POPC C312 6819 3.878 0.312 3.187

51POPC H12X 6820 3.771 0.342 3.185

51POPC H12Y 6821 3.878 0.202 3.203

51POPC C313 6822 3.944 0.340 3.052

51POPC H13X 6823 4.052 0.363 3.066

51POPC H13Y 6824 3.901 0.430 3.003

51POPC C314 6825 3.927 0.219 2.960

51POPC H14X 6826 3.878 0.242 2.863

51POPC H14Y 6827 3.861 0.150 3.016

51POPC C315 6828 4.057 0.144 2.941

51POPC H15X 6829 4.112 0.184 2.853

51POPC H15Y 6830 4.118 0.171 3.030

51POPC C316 6831 4.045 6.391 2.938

51POPC H16X 6832 3.978 6.358 2.856

51POPC H16Y 6833 4.144 6.344 2.923

51POPC H16Z 6834 4.001 6.352 3.032

52POPC N 6835 4.648 0.605 4.812

52POPC C12 6836 4.742 0.485 4.808

52POPC H12A 6837 4.813 0.498 4.728

52POPC H12B 6838 4.685 0.397 4.783

52POPC C13 6839 4.582 0.623 4.677

52POPC H13A 6840 4.655 0.641 4.599

52POPC H13B 6841 4.528 0.535 4.647

52POPC H13C 6842 4.513 0.705 4.678

52POPC C14 6843 4.544 0.583 4.918

52POPC H14A 6844 4.475 0.665 4.928

52POPC H14B 6845 4.492 0.489 4.904

52POPC H14C 6846 4.595 0.569 5.012

52POPC C15 6847 4.725 0.727 4.850

52POPC H15A 6848 4.807 0.746 4.781

52POPC H15B 6849 4.768 0.713 4.948

52POPC H15C 6850 4.662 0.814 4.856

52POPC C11 6851 4.822 0.447 4.936

52POPC H11A 6852 4.890 0.530 4.963

52POPC H11B 6853 4.887 0.360 4.913

52POPC P 6854 4.649 0.285 5.049

52POPC O13 6855 4.511 0.323 5.085

52POPC O14 6856 4.719 0.181 5.127

52POPC O12 6857 4.737 0.417 5.047

52POPC O11 6858 4.646 0.247 4.896

52POPC C1 6859 4.564 0.141 4.848

52POPC HA 6860 4.465 0.135 4.898

52POPC HB 6861 4.616 0.045 4.868

52POPC C2 6862 4.542 0.150 4.693

52POPC HS 6863 4.516 0.050 4.654

52POPC O21 6864 4.662 0.199 4.631

52POPC C21 6865 4.767 0.120 4.637

52POPC O22 6866 4.779 0.013 4.693

52POPC C22 6867 4.875 0.183 4.552

52POPC H2R 6868 4.845 0.171 4.446

52POPC H2S 6869 4.966 0.121 4.570

52POPC C3 6870 4.425 0.247 4.657

52POPC HX 6871 4.457 0.350 4.685

52POPC HY 6872 4.341 0.218 4.724

52POPC O31 6873 4.377 0.237 4.520

52POPC C31 6874 4.373 0.347 4.444

52POPC O32 6875 4.418 0.456 4.478

52POPC C32 6876 4.289 0.320 4.315

52POPC H2X 6877 4.199 0.385 4.322

52POPC H2Y 6878 4.255 0.214 4.320

52POPC C23 6879 4.903 0.330 4.587

52POPC H3R 6880 4.946 0.335 4.689

52POPC H3S 6881 4.806 0.385 4.584

52POPC C24 6882 4.996 0.400 4.489

52POPC H4R 6883 5.095 0.349 4.488

52POPC H4S 6884 5.015 0.503 4.526

52POPC C25 6885 4.939 0.409 4.347

52POPC H5R 6886 4.931 0.307 4.302

52POPC H5S 6887 5.015 0.460 4.284

52POPC C26 6888 4.805 0.485 4.337

52POPC H6R 6889 4.799 0.554 4.424

52POPC H6S 6890 4.721 0.414 4.351

52POPC C27 6891 4.780 0.570 4.211

52POPC H7R 6892 4.801 0.676 4.236

52POPC H7S 6893 4.672 0.566 4.185

52POPC C28 6894 4.863 0.538 4.084

52POPC H8R 6895 4.972 0.541 4.105

52POPC H8S 6896 4.847 0.623 4.014

52POPC C29 6897 4.826 0.408 4.020

52POPC H91 6898 4.872 0.318 4.063

52POPC C210 6899 4.745 0.396 3.913

52POPC H101 6900 4.727 0.296 3.871

52POPC C211 6901 4.678 0.511 3.844

52POPC H11R 6902 4.659 0.596 3.913

52POPC H11S 6903 4.578 0.480 3.805

52POPC C212 6904 4.766 0.560 3.729

52POPC H12R 6905 4.778 0.477 3.656

52POPC H12S 6906 4.867 0.586 3.766

52POPC C213 6907 4.706 0.685 3.662

52POPC H13R 6908 4.716 0.770 3.732

52POPC H13S 6909 4.598 0.670 3.642

52POPC C214 6910 4.776 0.719 3.531

52POPC H14R 6911 4.885 0.724 3.550

52POPC H14S 6912 4.744 0.819 3.495

52POPC C215 6913 4.751 0.617 3.419

52POPC H15R 6914 4.646 0.619 3.383

52POPC H15S 6915 4.776 0.516 3.458

52POPC C216 6916 4.850 0.647 3.308

52POPC H16R 6917 4.948 0.629 3.357

52POPC H16S 6918 4.839 0.754 3.281

52POPC C217 6919 4.850 0.566 3.179

52POPC H17R 6920 4.853 0.457 3.202

52POPC H17S 6921 4.948 0.588 3.131

52POPC C218 6922 4.742 0.604 3.077

52POPC H18R 6923 4.744 0.533 2.991

52POPC H18S 6924 4.760 0.706 3.038

52POPC H18T 6925 4.641 0.600 3.123

52POPC C33 6926 4.355 0.343 4.174

52POPC H3X 6927 4.458 0.384 4.184

52POPC H3Y 6928 4.295 0.420 4.121

52POPC C34 6929 4.356 0.217 4.083

52POPC H4X 6930 4.253 0.178 4.077

52POPC H4Y 6931 4.415 0.133 4.125

52POPC C35 6932 4.409 0.239 3.939

52POPC H5X 6933 4.520 0.243 3.938

52POPC H5Y 6934 4.372 0.337 3.902

52POPC C36 6935 4.359 0.130 3.841

52POPC H6X 6936 4.304 0.179 3.759

52POPC H6Y 6937 4.287 0.065 3.896

52POPC C37 6938 4.462 0.036 3.775

52POPC H7X 6939 4.402 6.367 3.713

52POPC H7Y 6940 4.511 6.372 3.852

52POPC C38 6941 4.568 0.102 3.685

52POPC H8X 6942 4.649 0.147 3.746

52POPC H8Y 6943 4.519 0.186 3.631

52POPC C39 6944 4.629 0.008 3.579

52POPC H9X 6945 4.680 6.319 3.622

52POPC H9Y 6946 4.710 0.061 3.524

52POPC C310 6947 4.522 6.358 3.483

52POPC H10X 6948 4.459 0.046 3.458

52POPC H10Y 6949 4.456 6.285 3.535

52POPC C311 6950 4.572 6.289 3.355

52POPC H11X 6951 4.486 6.227 3.321

52POPC H11Y 6952 4.659 6.223 3.372

52POPC C312 6953 4.588 6.382 3.235

52POPC H12X 6954 4.529 0.073 3.260

52POPC H12Y 6955 4.546 6.337 3.143

52POPC C313 6956 4.729 0.029 3.208

52POPC H13X 6957 4.779 6.349 3.150

52POPC H13Y 6958 4.780 0.039 3.306

52POPC C314 6959 4.728 0.163 3.133

52POPC H14X 6960 4.673 0.150 3.037

52POPC H14Y 6961 4.831 0.194 3.105

52POPC C315 6962 4.663 0.279 3.210

52POPC H15X 6963 4.552 0.267 3.210

52POPC H15Y 6964 4.678 0.373 3.153

52POPC C316 6965 4.693 0.293 3.358

52POPC H16X 6966 4.651 0.206 3.412

52POPC H16Y 6967 4.637 0.381 3.398

52POPC H16Z 6968 4.801 0.303 3.378

53POPC N 6969 0.311 1.477 5.065

53POPC C12 6970 0.292 1.480 4.914

53POPC H12A 6971 0.368 1.545 4.872

53POPC H12B 6972 0.197 1.525 4.891

53POPC C13 6973 0.453 1.438 5.097

53POPC H13A 6974 0.525 1.510 5.063

53POPC H13B 6975 0.469 1.424 5.203

53POPC H13C 6976 0.478 1.346 5.046

53POPC C14 6977 0.283 1.610 5.127

53POPC H14A 6978 0.296 1.604 5.234

53POPC H14B 6979 0.350 1.686 5.090

53POPC H14C 6980 0.181 1.641 5.110

53POPC C15 6981 0.220 1.376 5.129

53POPC H15A 6982 0.235 1.375 5.236

53POPC H15B 6983 0.243 1.278 5.090

53POPC H15C 6984 0.117 1.400 5.111

53POPC C11 6985 0.305 1.347 4.833

53POPC H11A 6986 0.297 1.372 4.725

53POPC H11B 6987 0.219 1.280 4.858

53POPC P 6988 0.517 1.207 4.757

53POPC O13 6989 0.445 1.184 4.630

53POPC O14 6990 0.573 1.087 4.823

53POPC O12 6991 0.426 1.279 4.863

53POPC O11 6992 0.632 1.314 4.733

53POPC C1 6993 0.764 1.283 4.782

53POPC HA 6994 0.806 1.197 4.727

53POPC HB 6995 0.758 1.259 4.890

53POPC C2 6996 0.867 1.400 4.769

53POPC HS 6997 0.943 1.386 4.850

53POPC O21 6998 0.805 1.527 4.783

53POPC C21 6999 0.748 1.551 4.897

53POPC O22 7000 0.747 1.480 4.997

53POPC C22 7001 0.693 1.692 4.893

53POPC H2R 7002 0.763 1.749 4.827

53POPC H2S 7003 0.706 1.735 4.995

53POPC C3 7004 0.948 1.400 4.637

53POPC HX 7005 1.007 1.306 4.627

53POPC HY 7006 1.021 1.483 4.644

53POPC O31 7007 0.865 1.426 4.523

53POPC C31 7008 0.820 1.324 4.456

53POPC O32 7009 0.843 1.206 4.479

53POPC C32 7010 0.733 1.378 4.344

53POPC H2X 7011 0.782 1.468 4.301

53POPC H2Y 7012 0.637 1.408 4.391

53POPC C23 7013 0.545 1.704 4.849

53POPC H3R 7014 0.521 1.813 4.843

53POPC H3S 7015 0.480 1.663 4.930

53POPC C24 7016 0.508 1.640 4.714

53POPC H4R 7017 0.504 1.529 4.722

53POPC H4S 7018 0.588 1.661 4.640

53POPC C25 7019 0.376 1.693 4.653

53POPC H5R 7020 0.362 1.799 4.685

53POPC H5S 7021 0.290 1.635 4.693

53POPC C26 7022 0.367 1.693 4.498

53POPC H6R 7023 0.423 1.781 4.460

53POPC H6S 7024 0.261 1.706 4.469

53POPC C27 7025 0.425 1.567 4.431

53POPC H7R 7026 0.400 1.477 4.492

53POPC H7S 7027 0.535 1.578 4.432

53POPC C28 7028 0.380 1.539 4.285

53POPC H8R 7029 0.277 1.496 4.290

53POPC H8S 7030 0.445 1.460 4.244

53POPC C29 7031 0.375 1.658 4.193

53POPC H91 7032 0.288 1.723 4.211

53POPC C210 7033 0.460 1.694 4.095

53POPC H101 7034 0.435 1.784 4.037

53POPC C211 7035 0.588 1.632 4.045

53POPC H11R 7036 0.613 1.537 4.097

53POPC H11S 7037 0.672 1.703 4.062

53POPC C212 7038 0.573 1.610 3.894

53POPC H12R 7039 0.532 1.700 3.844

53POPC H12S 7040 0.500 1.527 3.878

53POPC C213 7041 0.703 1.576 3.821

53POPC H13R 7042 0.758 1.499 3.879

53POPC H13S 7043 0.769 1.666 3.815

53POPC C214 7044 0.672 1.523 3.680

53POPC H14R 7045 0.628 1.605 3.619

53POPC H14S 7046 0.595 1.444 3.690

53POPC C215 7047 0.792 1.463 3.607

53POPC H15R 7048 0.843 1.391 3.675

53POPC H15S 7049 0.866 1.542 3.581

53POPC C216 7050 0.749 1.387 3.481

53POPC H16R 7051 0.666 1.317 3.507

53POPC H16S 7052 0.834 1.325 3.446

53POPC C217 7053 0.704 1.478 3.366

53POPC H17R 7054 0.779 1.559 3.353

53POPC H17S 7055 0.608 1.527 3.392

53POPC C218 7056 0.690 1.402 3.234

53POPC H18R 7057 0.780 1.339 3.216

53POPC H18S 7058 0.678 1.473 3.149

53POPC H18T 7059 0.604 1.332 3.234

53POPC C33 7060 0.704 1.274 4.235

53POPC H3X 7061 0.633 1.320 4.162

53POPC H3Y 7062 0.654 1.184 4.277

53POPC C34 7063 0.832 1.233 4.161

53POPC H4X 7064 0.901 1.181 4.230

53POPC H4Y 7065 0.882 1.327 4.126

53POPC C35 7066 0.805 1.146 4.040

53POPC H5X 7067 0.731 1.068 4.069

53POPC H5Y 7068 0.899 1.093 4.010

53POPC C36 7069 0.756 1.228 3.921

53POPC H6X 7070 0.840 1.293 3.886

53POPC H6Y 7071 0.673 1.295 3.950

53POPC C37 7072 0.709 1.141 3.805

53POPC H7X 7073 0.789 1.068 3.781

53POPC H7Y 7074 0.695 1.207 3.716

53POPC C38 7075 0.578 1.069 3.836

53POPC H8X 7076 0.498 1.146 3.848

53POPC H8Y 7077 0.585 1.014 3.932

53POPC C39 7078 0.537 0.969 3.727

53POPC H9X 7079 0.437 0.928 3.751

53POPC H9Y 7080 0.608 0.884 3.732

53POPC C310 7081 0.537 1.028 3.586

53POPC H10X 7082 0.528 0.944 3.513

53POPC H10Y 7083 0.634 1.077 3.562

53POPC C311 7084 0.422 1.127 3.567

53POPC H11X 7085 0.435 1.209 3.640

53POPC H11Y 7086 0.325 1.076 3.588

53POPC C312 7087 0.414 1.187 3.427

53POPC H12X 7088 0.376 1.111 3.356

53POPC H12Y 7089 0.517 1.215 3.393

53POPC C313 7090 0.325 1.312 3.426

53POPC H13X 7091 0.364 1.383 3.503

53POPC H13Y 7092 0.222 1.284 3.455

53POPC C314 7093 0.324 1.385 3.292

53POPC H14X 7094 0.297 1.315 3.210

53POPC H14Y 7095 0.427 1.421 3.271

53POPC C315 7096 0.234 1.509 3.288

53POPC H15X 7097 0.242 1.561 3.385

53POPC H15Y 7098 0.128 1.477 3.275

53POPC C316 7099 0.274 1.606 3.177

53POPC H16X 7100 0.379 1.639 3.193

53POPC H16Y 7101 0.210 1.696 3.178

53POPC H16Z 7102 0.268 1.558 3.077

54POPC N 7103 6.192 4.435 5.067

54POPC C12 7104 6.234 4.472 4.926

54POPC H12A 7105 6.335 4.510 4.927

54POPC H12B 7106 6.168 4.547 4.886

54POPC C13 7107 6.292 4.344 5.133

54POPC H13A 7108 6.309 4.257 5.072

54POPC H13B 7109 6.389 4.391 5.146

54POPC H13C 7110 6.258 4.310 5.230

54POPC C14 7111 6.185 4.555 5.154

54POPC H14A 7112 6.284 4.591 5.175

54POPC H14B 7113 6.129 4.634 5.105

54POPC H14C 7114 6.140 4.529 5.249

54POPC C15 7115 6.062 4.360 5.058

54POPC H15A 7116 6.079 4.270 5.002

54POPC H15B 7117 5.988 4.416 5.003

54POPC H15C 7118 6.024 4.328 5.154

54POPC C11 7119 6.233 4.357 4.824

54POPC H11A 7120 6.256 4.399 4.723

54POPC H11B 7121 6.132 4.312 4.812

54POPC P 7122 6.305 4.112 4.877

54POPC O13 7123 6.163 4.097 4.916

54POPC O14 7124 0.004 4.058 4.972

54POPC O12 7125 6.334 4.266 4.863

54POPC O11 7126 6.330 4.059 4.729

54POPC C1 7127 0.008 4.149 4.648

54POPC HA 7128 0.092 4.191 4.706

54POPC HB 7129 6.344 4.231 4.609

54POPC C2 7130 0.089 4.082 4.536

54POPC HS 7131 0.166 4.154 4.501

54POPC O21 7132 0.004 4.047 4.425

54POPC C21 7133 6.355 4.150 4.358

54POPC O22 7134 6.381 4.268 4.381

54POPC C22 7135 6.262 4.102 4.247

54POPC H2R 7136 6.169 4.067 4.296

54POPC H2S 7137 6.310 4.016 4.195

54POPC C3 7138 0.166 3.964 4.600

54POPC HX 7139 0.094 3.896 4.650

54POPC HY 7140 0.229 4.004 4.682

54POPC O31 7141 0.254 3.895 4.508

54POPC C31 7142 0.184 3.807 4.439

54POPC O32 7143 0.064 3.791 4.445

54POPC C32 7144 0.271 3.740 4.336

54POPC H2X 7145 0.263 3.630 4.348

54POPC H2Y 7146 0.377 3.773 4.344

54POPC C23 7147 6.230 4.215 4.147

54POPC H3R 7148 6.320 4.240 4.087

54POPC H3S 7149 6.202 4.307 4.203

54POPC C24 7150 6.113 4.182 4.053

54POPC H4R 7151 6.029 4.143 4.114

54POPC H4S 7152 6.143 4.101 3.983

54POPC C25 7153 6.063 4.305 3.975

54POPC H5R 7154 6.042 4.386 4.047

54POPC H5S 7155 5.968 4.279 3.924

54POPC C26 7156 6.162 4.356 3.869

54POPC H6R 7157 6.179 4.276 3.794

54POPC H6S 7158 6.260 4.377 3.918

54POPC C27 7159 6.114 4.484 3.798

54POPC H7R 7160 6.113 4.567 3.872

54POPC H7S 7161 6.010 4.467 3.763

54POPC C28 7162 6.199 4.523 3.677

54POPC H8R 7163 6.153 4.610 3.625

54POPC H8S 7164 6.199 4.438 3.605

54POPC C29 7165 6.339 4.559 3.721

54POPC H91 7166 6.349 4.656 3.773

54POPC C210 7167 0.049 4.484 3.705

54POPC H101 7168 0.144 4.518 3.748

54POPC C211 7169 0.057 4.350 3.638

54POPC H11R 7170 6.377 4.285 3.678

54POPC H11S 7171 0.154 4.301 3.663

54POPC C212 7172 0.043 4.354 3.484

54POPC H12R 7173 0.068 4.454 3.444

54POPC H12S 7174 6.336 4.336 3.460

54POPC C213 7175 0.131 4.252 3.410

54POPC H13R 7176 0.138 4.154 3.462

54POPC H13S 7177 0.236 4.288 3.403

54POPC C214 7178 0.074 4.225 3.271

54POPC H14R 7179 0.112 4.305 3.203

54POPC H14S 7180 6.363 4.233 3.269

54POPC C215 7181 0.115 4.090 3.214

54POPC H15R 7182 0.034 4.015 3.227

54POPC H15S 7183 0.205 4.053 3.268

54POPC C216 7184 0.147 4.112 3.067

54POPC H16R 7185 0.224 4.192 3.062

54POPC H16S 7186 0.054 4.142 3.015

54POPC C217 7187 0.211 3.995 2.998

54POPC H17R 7188 0.209 4.012 2.888

54POPC H17S 7189 0.143 3.909 3.018

54POPC C218 7190 0.356 3.975 3.045

54POPC H18R 7191 0.419 4.057 3.007

54POPC H18S 7192 0.398 3.878 3.009

54POPC H18T 7193 0.362 3.974 3.156

54POPC C33 7194 0.207 3.781 4.205

54POPC H3X 7195 0.208 3.892 4.199

54POPC H3Y 7196 0.101 3.747 4.203

54POPC C34 7197 0.275 3.726 4.083

54POPC H4X 7198 0.297 3.618 4.094

54POPC H4Y 7199 0.371 3.781 4.070

54POPC C35 7200 0.183 3.747 3.963

54POPC H5X 7201 0.105 3.669 3.965

54POPC H5Y 7202 0.240 3.734 3.868

54POPC C36 7203 0.114 3.883 3.960

54POPC H6X 7204 0.192 3.960 3.947

54POPC H6Y 7205 0.057 3.908 4.052

54POPC C37 7206 0.013 3.885 3.847

54POPC H7X 7207 6.313 3.851 3.880

54POPC H7Y 7208 0.048 3.813 3.770

54POPC C38 7209 0.007 4.023 3.784

54POPC H8X 7210 0.107 4.070 3.792

54POPC H8Y 7211 6.334 4.089 3.836

54POPC C39 7212 6.378 4.010 3.637

54POPC H9X 7213 0.037 3.929 3.589

54POPC H9Y 7214 0.013 4.105 3.592

54POPC C310 7215 6.231 4.001 3.601

54POPC H10X 7216 6.176 4.079 3.658

54POPC H10Y 7217 6.188 3.902 3.626

54POPC C311 7218 6.220 4.033 3.452

54POPC H11X 7219 6.262 3.949 3.392

54POPC H11Y 7220 6.280 4.124 3.431

54POPC C312 7221 6.077 4.064 3.412

54POPC H12X 7222 6.031 4.128 3.490

54POPC H12Y 7223 6.023 3.967 3.408

54POPC C313 7224 6.067 4.137 3.279

54POPC H13X 7225 6.114 4.238 3.288

54POPC H13Y 7226 5.959 4.152 3.260

54POPC C314 7227 6.131 4.062 3.163

54POPC H14X 7228 6.164 3.961 3.195

54POPC H14Y 7229 6.222 4.117 3.130

54POPC C315 7230 6.035 4.046 3.045

54POPC H15X 7231 5.958 3.970 3.069

54POPC H15Y 7232 6.096 4.010 2.959

54POPC C316 7233 5.966 4.175 3.003

54POPC H16X 7234 5.884 4.203 3.072

54POPC H16Y 7235 5.924 4.166 2.901

54POPC H16Z 7236 6.041 4.257 3.006

55POPC N 7237 1.802 0.280 4.724

55POPC C12 7238 1.718 0.313 4.847

55POPC H12A 7239 1.629 0.365 4.816

55POPC H12B 7240 1.772 0.386 4.906

55POPC C13 7241 1.727 0.207 4.617

55POPC H13A 7242 1.632 0.254 4.593

55POPC H13B 7243 1.784 0.200 4.526

55POPC H13C 7244 1.706 0.105 4.647

55POPC C14 7245 1.849 0.409 4.664

55POPC H14A 7246 1.904 0.466 4.738

55POPC H14B 7247 1.911 0.391 4.578

55POPC H14C 7248 1.763 0.468 4.633

55POPC C15 7249 1.918 0.195 4.765

55POPC H15A 7250 1.981 0.246 4.837

55POPC H15B 7251 1.976 0.160 4.682

55POPC H15C 7252 1.880 0.109 4.819

55POPC C11 7253 1.674 0.206 4.951

55POPC H11A 7254 1.602 0.134 4.905

55POPC H11B 7255 1.616 0.263 5.026

55POPC P 7256 1.726 0.039 5.135

55POPC O13 7257 1.580 0.055 5.152

55POPC O14 7258 1.807 0.066 5.256

55POPC O12 7259 1.778 0.130 5.012

55POPC O11 7260 1.751 6.291 5.094

55POPC C1 7261 1.839 6.246 4.994

55POPC HA 7262 1.887 6.154 5.034

55POPC HB 7263 1.921 6.318 4.973

55POPC C2 7264 1.769 6.205 4.860

55POPC HS 7265 1.781 6.293 4.794

55POPC O21 7266 1.626 6.189 4.881

55POPC C21 7267 1.552 6.098 4.819

55POPC O22 7268 1.595 6.008 4.749

55POPC C22 7269 1.400 6.118 4.847

55POPC H2R 7270 1.385 6.211 4.906

55POPC H2S 7271 1.370 6.033 4.912

55POPC C3 7272 1.857 6.091 4.797

55POPC HX 7273 1.838 5.996 4.853

55POPC HY 7274 1.963 6.118 4.817

55POPC O31 7275 1.842 6.068 4.656

55POPC C31 7276 1.894 6.159 4.577

55POPC O32 7277 1.953 6.260 4.614

55POPC C32 7278 1.871 6.117 4.432

55POPC H2X 7279 1.764 6.091 4.419

55POPC H2Y 7280 1.930 6.025 4.415

55POPC C23 7281 1.309 6.117 4.717

55POPC H3R 7282 1.249 6.024 4.724

55POPC H3S 7283 1.375 6.111 4.628

55POPC C24 7284 1.204 6.228 4.692

55POPC H4R 7285 1.251 6.329 4.696

55POPC H4S 7286 1.134 6.225 4.778

55POPC C25 7287 1.125 6.229 4.557

55POPC H5R 7288 1.016 6.219 4.579

55POPC H5S 7289 1.149 6.137 4.499

55POPC C26 7290 1.140 6.359 4.469

55POPC H6R 7291 1.238 0.005 4.496

55POPC H6S 7292 1.061 0.029 4.500

55POPC C27 7293 1.138 6.343 4.313

55POPC H7R 7294 1.035 6.324 4.276

55POPC H7S 7295 1.189 6.244 4.308

55POPC C28 7296 1.215 0.047 4.219

55POPC H8R 7297 1.125 0.103 4.188

55POPC H8S 7298 1.270 0.115 4.288

55POPC C29 7299 1.284 0.050 4.081

55POPC H91 7300 1.230 0.126 4.021

55POPC C210 7301 1.389 6.400 4.009

55POPC H101 7302 1.398 0.039 3.907

55POPC C211 7303 1.502 6.301 4.030

55POPC H11R 7304 1.501 6.263 4.135

55POPC H11S 7305 1.603 6.345 4.016

55POPC C212 7306 1.495 6.169 3.952

55POPC H12R 7307 1.388 6.139 3.942

55POPC H12S 7308 1.544 6.090 4.012

55POPC C213 7309 1.555 6.167 3.814

55POPC H13R 7310 1.572 6.272 3.779

55POPC H13S 7311 1.484 6.119 3.743

55POPC C214 7312 1.684 6.086 3.814

55POPC H14R 7313 1.666 5.983 3.774

55POPC H14S 7314 1.723 6.076 3.917

55POPC C215 7315 1.789 6.159 3.734

55POPC H15R 7316 1.888 6.116 3.762

55POPC H15S 7317 1.784 6.264 3.768

55POPC C216 7318 1.767 6.155 3.584

55POPC H16R 7319 1.658 6.162 3.563

55POPC H16S 7320 1.801 6.058 3.543

55POPC C217 7321 1.837 6.272 3.516

55POPC H17R 7322 1.931 6.236 3.468

55POPC H17S 7323 1.867 6.347 3.592

55POPC C218 7324 1.752 6.342 3.412

55POPC H18R 7325 1.734 6.277 3.324

55POPC H18S 7326 1.808 0.031 3.376

55POPC H18T 7327 1.654 6.373 3.454

55POPC C33 7328 1.913 6.226 4.331

55POPC H3X 7329 1.904 6.182 4.229

55POPC H3Y 7330 2.020 6.250 4.349

55POPC C34 7331 1.828 6.353 4.337

55POPC H4X 7332 1.838 0.002 4.436

55POPC H4Y 7333 1.723 6.318 4.327

55POPC C35 7334 1.851 0.054 4.224

55POPC H5X 7335 1.847 6.399 4.128

55POPC H5Y 7336 1.953 0.099 4.233

55POPC C36 7337 1.750 0.170 4.223

55POPC H6X 7338 1.765 0.232 4.314

55POPC H6Y 7339 1.647 0.130 4.225

55POPC C37 7340 1.765 0.260 4.098

55POPC H7X 7341 1.873 0.278 4.078

55POPC H7Y 7342 1.716 0.357 4.115

55POPC C38 7343 1.701 0.193 3.976

55POPC H8X 7344 1.592 0.186 3.994

55POPC H8Y 7345 1.740 0.090 3.967

55POPC C39 7346 1.723 0.259 3.840

55POPC H9X 7347 1.832 0.271 3.819

55POPC H9Y 7348 1.678 0.361 3.838

55POPC C310 7349 1.661 0.171 3.732

55POPC H10X 7350 1.567 0.127 3.772

55POPC H10Y 7351 1.729 0.085 3.712

55POPC C311 7352 1.623 0.238 3.599

55POPC H11X 7353 1.523 0.286 3.612

55POPC H11Y 7354 1.609 0.157 3.525

55POPC C312 7355 1.721 0.340 3.540

55POPC H12X 7356 1.824 0.298 3.544

55POPC H12Y 7357 1.721 0.432 3.602

55POPC C313 7358 1.686 0.377 3.394

55POPC H13X 7359 1.752 0.460 3.362

55POPC H13Y 7360 1.582 0.414 3.388

55POPC C314 7361 1.703 0.257 3.298

55POPC H14X 7362 1.629 0.179 3.324

55POPC H14Y 7363 1.805 0.216 3.316

55POPC C315 7364 1.690 0.281 3.147

55POPC H15X 7365 1.586 0.310 3.124

55POPC H15Y 7366 1.705 0.184 3.096

55POPC C316 7367 1.790 0.382 3.091

55POPC H16X 7368 1.780 0.481 3.140

55POPC H16Y 7369 1.773 0.395 2.982

55POPC H16Z 7370 1.895 0.348 3.106

56POPC N 7371 5.573 0.538 4.855

56POPC C12 7372 5.588 0.564 5.004

56POPC H12A 7373 5.674 0.511 5.042

56POPC H12B 7374 5.503 0.523 5.055

56POPC C13 7375 5.444 0.596 4.807

56POPC H13A 7376 5.432 0.586 4.700

56POPC H13B 7377 5.360 0.550 4.856

56POPC H13C 7378 5.438 0.701 4.835

56POPC C14 7379 5.688 0.594 4.776

56POPC H14A 7380 5.781 0.547 4.806

56POPC H14B 7381 5.673 0.578 4.671

56POPC H14C 7382 5.696 0.700 4.795

56POPC C15 7383 5.564 0.392 4.834

56POPC H15A 7384 5.549 0.370 4.729

56POPC H15B 7385 5.479 0.351 4.887

56POPC H15C 7386 5.651 0.339 4.870

56POPC C11 7387 5.598 0.712 5.047

56POPC H11A 7388 5.688 0.758 5.002

56POPC H11B 7389 5.611 0.712 5.158

56POPC P 7390 5.466 0.936 5.042

56POPC O13 7391 5.596 0.986 5.092

56POPC O14 7392 5.348 0.955 5.131

56POPC O12 7393 5.480 0.782 5.012

56POPC O11 7394 5.444 0.997 4.898

56POPC C1 7395 5.532 0.967 4.789

56POPC HA 7396 5.558 0.859 4.785

56POPC HB 7397 5.627 1.023 4.801

56POPC C2 7398 5.466 1.007 4.654

56POPC HS 7399 5.378 1.069 4.679

56POPC O21 7400 5.426 0.894 4.576

56POPC C21 7401 5.323 0.826 4.617

56POPC O22 7402 5.268 0.832 4.725

56POPC C22 7403 5.276 0.738 4.503

56POPC H2R 7404 5.287 0.631 4.532

56POPC H2S 7405 5.336 0.758 4.412

56POPC C3 7406 5.554 1.094 4.559

56POPC HX 7407 5.603 1.176 4.617

56POPC HY 7408 5.485 1.142 4.487

56POPC O31 7409 5.648 1.016 4.483

56POPC C31 7410 5.775 1.017 4.520

56POPC O32 7411 5.822 1.086 4.609

56POPC C32 7412 5.851 0.911 4.441

56POPC H2X 7413 5.886 0.834 4.514

56POPC H2Y 7414 5.941 0.958 4.396

56POPC C23 7415 5.130 0.775 4.480

56POPC H3R 7416 5.122 0.885 4.466

56POPC H3S 7417 5.070 0.746 4.569

56POPC C24 7418 5.068 0.711 4.357

56POPC H4R 7419 4.960 0.697 4.377

56POPC H4S 7420 5.117 0.612 4.344

56POPC C25 7421 5.081 0.791 4.227

56POPC H5R 7422 5.099 0.898 4.250

56POPC H5S 7423 4.986 0.787 4.171

56POPC C26 7424 5.191 0.731 4.139

56POPC H6R 7425 5.162 0.626 4.118

56POPC H6S 7426 5.287 0.728 4.195

56POPC C27 7427 5.212 0.803 4.005

56POPC H7R 7428 5.123 0.785 3.941

56POPC H7S 7429 5.299 0.757 3.954

56POPC C28 7430 5.234 0.955 4.019

56POPC H8R 7431 5.137 1.003 4.043

56POPC H8S 7432 5.264 0.992 3.918

56POPC C29 7433 5.336 0.989 4.125

56POPC H91 7434 5.306 0.963 4.227

56POPC C210 7435 5.457 1.044 4.107

56POPC H101 7436 5.522 1.061 4.194

56POPC C211 7437 5.516 1.085 3.974

56POPC H11R 7438 5.584 1.171 3.987

56POPC H11S 7439 5.437 1.116 3.903

56POPC C212 7440 5.593 0.968 3.911

56POPC H12R 7441 5.525 0.883 3.890

56POPC H12S 7442 5.669 0.935 3.984

56POPC C213 7443 5.665 1.008 3.782

56POPC H13R 7444 5.731 0.925 3.750

56POPC H13S 7445 5.730 1.096 3.802

56POPC C214 7446 5.572 1.039 3.664

56POPC H14R 7447 5.509 1.128 3.688

56POPC H14S 7448 5.504 0.953 3.646

56POPC C215 7449 5.655 1.067 3.539

56POPC H15R 7450 5.694 0.970 3.502

56POPC H15S 7451 5.743 1.129 3.568

56POPC C216 7452 5.581 1.141 3.427

56POPC H16R 7453 5.653 1.163 3.345

56POPC H16S 7454 5.543 1.238 3.466

56POPC C217 7455 5.466 1.061 3.369

56POPC H17R 7456 5.410 1.123 3.296

56POPC H17S 7457 5.395 1.035 3.451

56POPC C218 7458 5.512 0.932 3.301

56POPC H18R 7459 5.470 0.844 3.355

56POPC H18S 7460 5.623 0.923 3.303

56POPC H18T 7461 5.478 0.929 3.196

56POPC C33 7462 5.768 0.841 4.330

56POPC H3X 7463 5.741 0.913 4.250

56POPC H3Y 7464 5.674 0.801 4.373

56POPC C34 7465 5.846 0.725 4.268

56POPC H4X 7466 5.887 0.663 4.351

56POPC H4Y 7467 5.930 0.765 4.208

56POPC C35 7468 5.763 0.632 4.179

56POPC H5X 7469 5.657 0.631 4.211

56POPC H5Y 7470 5.806 0.530 4.193

56POPC C36 7471 5.778 0.663 4.031

56POPC H6X 7472 5.874 0.621 3.994

56POPC H6Y 7473 5.781 0.773 4.019

56POPC C37 7474 5.663 0.609 3.946

56POPC H7X 7475 5.571 0.667 3.968

56POPC H7Y 7476 5.641 0.503 3.972

56POPC C38 7477 5.696 0.618 3.797

56POPC H8X 7478 5.782 0.552 3.772

56POPC H8Y 7479 5.725 0.722 3.773

56POPC C39 7480 5.576 0.581 3.711

56POPC H9X 7481 5.589 0.625 3.610

56POPC H9Y 7482 5.486 0.629 3.754

56POPC C310 7483 5.553 0.429 3.706

56POPC H10X 7484 5.443 0.414 3.710

56POPC H10Y 7485 5.596 0.379 3.795

56POPC C311 7486 5.598 0.358 3.578

56POPC H11X 7487 5.551 0.257 3.577

56POPC H11Y 7488 5.708 0.344 3.579

56POPC C312 7489 5.556 0.433 3.452

56POPC H12X 7490 5.477 0.507 3.474

56POPC H12Y 7491 5.513 0.364 3.376

56POPC C313 7492 5.676 0.499 3.387

56POPC H13X 7493 5.730 0.421 3.329

56POPC H13Y 7494 5.743 0.539 3.465

56POPC C314 7495 5.635 0.611 3.294

56POPC H14X 7496 5.618 0.701 3.357

56POPC H14Y 7497 5.540 0.584 3.244

56POPC C315 7498 5.740 0.640 3.187

56POPC H15X 7499 5.764 0.545 3.134

56POPC H15Y 7500 5.833 0.677 3.234

56POPC C316 7501 5.686 0.741 3.087

56POPC H16X 7502 5.735 0.730 2.988

56POPC H16Y 7503 5.702 0.844 3.123

56POPC H16Z 7504 5.577 0.727 3.074

57POPC N 7505 5.644 5.750 4.890

57POPC C12 7506 5.747 5.643 4.923

57POPC H12A 7507 5.702 5.546 4.909

57POPC H12B 7508 5.773 5.654 5.027

57POPC C13 7509 5.546 5.754 5.001

57POPC H13A 7510 5.498 5.658 5.016

57POPC H13B 7511 5.592 5.784 5.094

57POPC H13C 7512 5.466 5.823 4.980

57POPC C14 7513 5.708 5.885 4.884

57POPC H14A 7514 5.747 5.913 4.980

57POPC H14B 7515 5.794 5.883 4.820

57POPC H14C 7516 5.640 5.963 4.852

57POPC C15 7517 5.573 5.721 4.761

57POPC H15A 7518 5.643 5.712 4.679

57POPC H15B 7519 5.503 5.800 4.738

57POPC H15C 7520 5.514 5.631 4.769

57POPC C11 7521 5.883 5.647 4.849

57POPC H11A 7522 5.866 5.657 4.740

57POPC H11B 7523 5.940 5.553 4.865

57POPC P 7524 6.014 5.771 5.041

57POPC O13 7525 6.118 5.671 5.068

57POPC O14 7526 5.900 5.773 5.137

57POPC O12 7527 5.959 5.757 4.894

57POPC O11 7528 6.076 5.915 5.044

57POPC C1 7529 6.020 6.029 4.979

57POPC HA 7530 6.067 6.120 5.024

57POPC HB 7531 5.911 6.039 4.997

57POPC C2 7532 6.048 6.041 4.825

57POPC HS 7533 6.076 6.146 4.808

57POPC O21 7534 5.928 6.007 4.751

57POPC C21 7535 5.918 6.064 4.633

57POPC O22 7536 5.979 6.162 4.594

57POPC C22 7537 5.826 5.981 4.542

57POPC H2R 7538 5.828 5.875 4.577

57POPC H2S 7539 5.872 5.985 4.441

57POPC C3 7540 6.168 5.953 4.780

57POPC HX 7541 6.217 5.906 4.868

57POPC HY 7542 6.246 6.021 4.740

57POPC O31 7543 6.128 5.850 4.686

57POPC C31 7544 6.227 5.792 4.621

57POPC O32 7545 6.340 5.836 4.612

57POPC C32 7546 6.180 5.662 4.559

57POPC H2X 7547 6.085 5.629 4.606

57POPC H2Y 7548 6.258 5.587 4.583

57POPC C23 7549 5.682 6.033 4.532

57POPC H3R 7550 5.683 6.142 4.511

57POPC H3S 7551 5.632 6.020 4.631

57POPC C24 7552 5.598 5.962 4.424

57POPC H4R 7553 5.492 5.993 4.435

57POPC H4S 7554 5.601 5.852 4.440

57POPC C25 7555 5.639 5.992 4.279

57POPC H5R 7556 5.562 5.947 4.212

57POPC H5S 7557 5.735 5.940 4.257

57POPC C26 7558 5.654 6.141 4.246

57POPC H6R 7559 5.755 6.175 4.278

57POPC H6S 7560 5.579 6.198 4.306

57POPC C27 7561 5.632 6.177 4.098

57POPC H7R 7562 5.627 6.287 4.087

57POPC H7S 7563 5.533 6.136 4.069

57POPC C28 7564 5.742 6.123 4.006

57POPC H8R 7565 5.763 6.019 4.039

57POPC H8S 7566 5.836 6.181 4.020

57POPC C29 7567 5.702 6.116 3.861

57POPC H91 7568 5.743 6.194 3.795

57POPC C210 7569 5.629 6.016 3.809

57POPC H101 7570 5.606 6.019 3.701

57POPC C211 7571 5.565 5.902 3.886

57POPC H11R 7572 5.455 5.903 3.867

57POPC H11S 7573 5.575 5.914 3.996

57POPC C212 7574 5.609 5.759 3.851

57POPC H12R 7575 5.529 5.690 3.884

57POPC H12S 7576 5.699 5.732 3.910

57POPC C213 7577 5.636 5.736 3.702

57POPC H13R 7578 5.723 5.797 3.670

57POPC H13S 7579 5.548 5.774 3.644

57POPC C214 7580 5.663 5.589 3.673

57POPC H14R 7581 5.579 5.527 3.712

57POPC H14S 7582 5.753 5.566 3.733

57POPC C215 7583 5.688 5.552 3.526

57POPC H15R 7584 5.605 5.591 3.463

57POPC H15S 7585 5.689 5.441 3.514

57POPC C216 7586 5.822 5.607 3.475

57POPC H16R 7587 5.906 5.565 3.535

57POPC H16S 7588 5.818 5.716 3.495

57POPC C217 7589 5.851 5.582 3.327

57POPC H17R 7590 5.768 5.624 3.267

57POPC H17S 7591 5.854 5.473 3.308

57POPC C218 7592 5.983 5.645 3.281

57POPC H18R 7593 5.966 5.731 3.211

57POPC H18S 7594 6.045 5.570 3.228

57POPC H18T 7595 6.043 5.681 3.367

57POPC C33 7596 6.168 5.674 4.406

57POPC H3X 7597 6.165 5.570 4.366

57POPC H3Y 7598 6.259 5.722 4.364

57POPC C34 7599 6.040 5.742 4.358

57POPC H4X 7600 5.986 5.786 4.444

57POPC H4Y 7601 5.977 5.661 4.315

57POPC C35 7602 6.061 5.848 4.250

57POPC H5X 7603 6.165 5.840 4.210

57POPC H5Y 7604 6.052 5.950 4.293

57POPC C36 7605 5.967 5.831 4.130

57POPC H6X 7606 5.897 5.917 4.134

57POPC H6Y 7607 5.908 5.738 4.137

57POPC C37 7608 6.039 5.836 3.994

57POPC H7X 7609 6.123 5.763 3.992

57POPC H7Y 7610 6.080 5.939 3.988

57POPC C38 7611 5.943 5.808 3.878

57POPC H8X 7612 5.854 5.874 3.892

57POPC H8Y 7613 5.909 5.703 3.886

57POPC C39 7614 5.994 5.830 3.734

57POPC H9X 7615 5.947 5.753 3.669

57POPC H9Y 7616 6.103 5.811 3.729

57POPC C310 7617 5.955 5.968 3.680

57POPC H10X 7618 5.989 6.045 3.752

57POPC H10Y 7619 5.844 5.976 3.674

57POPC C311 7620 6.012 6.003 3.542

57POPC H11X 7621 6.114 5.959 3.533

57POPC H11Y 7622 6.020 6.114 3.535

57POPC C312 7623 5.934 5.954 3.421

57POPC H12X 7624 5.940 5.843 3.425

57POPC H12Y 7625 5.990 5.984 3.330

57POPC C313 7626 5.787 5.994 3.407

57POPC H13X 7627 5.774 6.104 3.412

57POPC H13Y 7628 5.729 5.950 3.492

57POPC C314 7629 5.729 5.942 3.275

57POPC H14X 7630 5.804 5.874 3.228

57POPC H14Y 7631 5.715 6.026 3.203

57POPC C315 7632 5.600 5.862 3.292

57POPC H15X 7633 5.519 5.931 3.323

57POPC H15Y 7634 5.614 5.789 3.374

57POPC C316 7635 5.559 5.786 3.165

57POPC H16X 7636 5.549 5.855 3.078

57POPC H16Y 7637 5.462 5.734 3.181

57POPC H16Z 7638 5.634 5.709 3.137

58POPC N 7639 6.301 4.998 4.941

58POPC C12 7640 0.036 5.065 4.971

58POPC H12A 7641 0.097 5.061 4.883

58POPC H12B 7642 0.091 5.008 5.045

58POPC C13 7643 6.331 4.870 4.868

58POPC H13A 7644 0.004 4.811 4.921

58POPC H13B 7645 6.242 4.813 4.845

58POPC H13C 7646 6.375 4.893 4.772

58POPC C14 7647 6.227 4.979 5.069

58POPC H14A 7648 6.129 4.937 5.051

58POPC H14B 7649 6.284 4.919 5.138

58POPC H14C 7650 6.214 5.075 5.116

58POPC C15 7651 6.221 5.087 4.851

58POPC H15A 7652 6.280 5.110 4.763

58POPC H15B 7653 6.204 5.184 4.895

58POPC H15C 7654 6.127 5.044 4.818

58POPC C11 7655 0.032 5.214 5.009

58POPC H11A 7656 0.128 5.243 5.055

58POPC H11B 7657 6.350 5.235 5.081

58POPC P 7658 6.346 5.421 4.870

58POPC O13 7659 6.393 5.509 4.978

58POPC O14 7660 6.201 5.393 4.855

58POPC O12 7661 0.017 5.283 4.887

58POPC O11 7662 0.009 5.471 4.734

58POPC C1 7663 0.142 5.433 4.697

58POPC HA 7664 0.200 5.384 4.779

58POPC HB 7665 0.199 5.524 4.670

58POPC C2 7666 0.145 5.340 4.573

58POPC HS 7667 0.252 5.318 4.553

58POPC O21 7668 0.091 5.416 4.464

58POPC C21 7669 0.066 5.353 4.352

58POPC O22 7670 0.113 5.247 4.315

58POPC C22 7671 6.370 5.439 4.272

58POPC H2R 7672 6.384 5.546 4.301

58POPC H2S 7673 6.269 5.406 4.303

58POPC C3 7674 0.073 5.202 4.593

58POPC HX 7675 0.083 5.170 4.698

58POPC HY 7676 0.128 5.129 4.531

58POPC O31 7677 6.334 5.210 4.552

58POPC C31 7678 6.275 5.094 4.532

58POPC O32 7679 6.312 4.988 4.579

58POPC C32 7680 6.147 5.115 4.452

58POPC H2X 7681 6.133 5.223 4.429

58POPC H2Y 7682 6.063 5.083 4.518

58POPC C23 7683 6.389 5.424 4.120

58POPC H3R 7684 6.373 5.317 4.093

58POPC H3S 7685 0.092 5.452 4.092

58POPC C24 7686 6.292 5.508 4.037

58POPC H4R 7687 6.307 5.482 3.930

58POPC H4S 7688 6.316 5.616 4.045

58POPC C25 7689 6.146 5.487 4.075

58POPC H5R 7690 6.106 5.576 4.129

58POPC H5S 7691 6.135 5.399 4.142

58POPC C26 7692 6.064 5.457 3.951

58POPC H6R 7693 6.110 5.369 3.902

58POPC H6S 7694 6.071 5.542 3.879

58POPC C27 7695 5.917 5.424 3.981

58POPC H7R 7696 5.858 5.519 3.983

58POPC H7S 7697 5.910 5.379 4.082

58POPC C28 7698 5.858 5.325 3.880

58POPC H8R 7699 5.748 5.320 3.893

58POPC H8S 7700 5.901 5.224 3.903

58POPC C29 7701 5.894 5.362 3.739

58POPC H91 7702 5.931 5.465 3.726

58POPC C210 7703 5.887 5.283 3.631

58POPC H101 7704 5.918 5.324 3.534

58POPC C211 7705 5.844 5.139 3.630

58POPC H11R 7706 5.797 5.116 3.531

58POPC H11S 7707 5.768 5.118 3.708

58POPC C212 7708 5.965 5.047 3.649

58POPC H12R 7709 5.934 4.942 3.632

58POPC H12S 7710 6.002 5.055 3.753

58POPC C213 7711 6.080 5.082 3.554

58POPC H13R 7712 6.117 5.184 3.580

58POPC H13S 7713 6.042 5.087 3.449

58POPC C214 7714 6.200 4.989 3.560

58POPC H14R 7715 6.182 4.899 3.498

58POPC H14S 7716 6.219 4.956 3.665

58POPC C215 7717 6.321 5.067 3.509

58POPC H15R 7718 6.339 5.152 3.579

58POPC H15S 7719 6.293 5.111 3.411

58POPC C216 7720 0.049 4.985 3.494

58POPC H16R 7721 0.061 4.917 3.581

58POPC H16S 7722 0.136 5.054 3.493

58POPC C217 7723 0.051 4.906 3.364

58POPC H17R 7724 6.368 4.832 3.364

58POPC H17S 7725 0.147 4.850 3.364

58POPC C218 7726 0.042 4.991 3.237

58POPC H18R 7727 0.067 4.929 3.147

58POPC H18S 7728 0.113 5.076 3.241

58POPC H18T 7729 6.340 5.032 3.223

58POPC C33 7730 6.143 5.032 4.322

58POPC H3X 7731 6.037 5.029 4.287

58POPC H3Y 7732 6.174 4.927 4.339

58POPC C34 7733 6.224 5.094 4.207

58POPC H4X 7734 6.332 5.093 4.233

58POPC H4Y 7735 6.193 5.200 4.195

58POPC C35 7736 6.203 5.022 4.073

58POPC H5X 7737 6.093 5.016 4.054

58POPC H5Y 7738 6.241 4.918 4.081

58POPC C36 7739 6.268 5.093 3.954

58POPC H6X 7740 6.236 5.200 3.953

58POPC H6Y 7741 6.231 5.046 3.860

58POPC C37 7742 0.021 5.083 3.956

58POPC H7X 7743 0.045 4.975 3.968

58POPC H7Y 7744 0.061 5.135 4.046

58POPC C38 7745 0.093 5.137 3.831

58POPC H8X 7746 0.068 5.071 3.745

58POPC H8Y 7747 0.202 5.131 3.848

58POPC C39 7748 0.056 5.281 3.799

58POPC H9X 7749 0.079 5.342 3.888

58POPC H9Y 7750 6.346 5.289 3.779

58POPC C310 7751 0.132 5.343 3.681

58POPC H10X 7752 0.106 5.292 3.585

58POPC H10Y 7753 0.241 5.329 3.697

58POPC C311 7754 0.101 5.493 3.671

58POPC H11X 7755 0.179 5.544 3.610

58POPC H11Y 7756 0.102 5.535 3.774

58POPC C312 7757 6.369 5.524 3.601

58POPC H12X 7758 6.327 5.619 3.639

58POPC H12Y 7759 6.296 5.443 3.623

58POPC C313 7760 6.392 5.537 3.451

58POPC H13X 7761 0.070 5.464 3.418

58POPC H13Y 7762 0.032 5.638 3.428

58POPC C314 7763 6.265 5.510 3.372

58POPC H14X 7764 6.254 5.589 3.294

58POPC H14Y 7765 6.178 5.518 3.441

58POPC C315 7766 6.267 5.371 3.307

58POPC H15X 7767 6.319 5.305 3.379

58POPC H15Y 7768 6.329 5.374 3.215

58POPC C316 7769 6.129 5.311 3.276

58POPC H16X 7770 6.139 5.206 3.243

58POPC H16Y 7771 6.077 5.367 3.196

58POPC H16Z 7772 6.065 5.314 3.367

59POPC N 7773 0.702 6.201 4.930

59POPC C12 7774 0.746 6.325 4.852

59POPC H12A 7775 0.717 0.013 4.907

59POPC H12B 7776 0.692 6.329 4.759

59POPC C13 7777 0.553 6.193 4.931

59POPC H13A 7778 0.518 6.109 4.989

59POPC H13B 7779 0.513 6.282 4.977

59POPC H13C 7780 0.513 6.189 4.830

59POPC C14 7781 0.762 6.077 4.871

59POPC H14A 7782 0.730 5.990 4.926

59POPC H14B 7783 0.735 6.068 4.767

59POPC H14C 7784 0.869 6.086 4.877

59POPC C15 7785 0.751 6.217 5.071

59POPC H15A 7786 0.857 6.237 5.070

59POPC H15B 7787 0.732 6.131 5.134

59POPC H15C 7788 0.704 6.301 5.119

59POPC C11 7789 0.898 6.336 4.823

59POPC H11A 7790 0.914 0.019 4.751

59POPC H11B 7791 0.930 6.244 4.769

59POPC P 7792 1.061 0.079 4.970

59POPC O13 7793 1.096 0.081 5.114

59POPC O14 7794 0.984 0.193 4.915

59POPC O12 7795 0.975 6.348 4.943

59POPC O11 7796 1.188 0.050 4.879

59POPC C1 7797 1.321 0.098 4.898

59POPC HA 7798 1.380 0.024 4.956

59POPC HB 7799 1.320 0.191 4.960

59POPC C2 7800 1.396 0.133 4.763

59POPC HS 7801 1.461 0.221 4.782

59POPC O21 7802 1.307 0.163 4.656

59POPC C21 7803 1.238 0.269 4.678

59POPC O22 7804 1.247 0.341 4.775

59POPC C22 7805 1.163 0.301 4.551

59POPC H2R 7806 1.198 0.231 4.472

59POPC H2S 7807 1.189 0.403 4.517

59POPC C3 7808 1.487 0.020 4.705

59POPC HX 7809 1.445 6.328 4.752

59POPC HY 7810 1.592 0.025 4.742

59POPC O31 7811 1.481 0.010 4.560

59POPC C31 7812 1.519 6.291 4.513

59POPC O32 7813 1.603 6.222 4.566

59POPC C32 7814 1.455 6.261 4.377

59POPC H2X 7815 1.540 6.283 4.309

59POPC H2Y 7816 1.380 6.342 4.359

59POPC C23 7817 1.014 0.286 4.577

59POPC H3R 7818 0.979 0.351 4.660

59POPC H3S 7819 0.993 0.183 4.613

59POPC C24 7820 0.932 0.312 4.452

59POPC H4R 7821 0.896 0.416 4.443

59POPC H4S 7822 0.849 0.239 4.466

59POPC C25 7823 0.994 0.269 4.321

59POPC H5R 7824 1.048 0.176 4.349

59POPC H5S 7825 1.068 0.343 4.282

59POPC C26 7826 0.892 0.227 4.215

59POPC H6R 7827 0.950 0.198 4.124

59POPC H6S 7828 0.827 0.313 4.186

59POPC C27 7829 0.808 0.105 4.254

59POPC H7R 7830 0.719 0.137 4.313

59POPC H7S 7831 0.867 0.038 4.321

59POPC C28 7832 0.763 0.022 4.134

59POPC H8R 7833 0.700 6.336 4.166

59POPC H8S 7834 0.853 6.382 4.083

59POPC C29 7835 0.684 0.116 4.047

59POPC H91 7836 0.608 0.177 4.098

59POPC C210 7837 0.709 0.146 3.920

59POPC H101 7838 0.644 0.219 3.869

59POPC C211 7839 0.818 0.087 3.837

59POPC H11R 7840 0.781 0.061 3.735

59POPC H11S 7841 0.862 6.394 3.878

59POPC C212 7842 0.919 0.199 3.828

59POPC H12R 7843 0.969 0.212 3.927

59POPC H12S 7844 0.870 0.294 3.798

59POPC C213 7845 1.026 0.178 3.725

59POPC H13R 7846 1.098 0.262 3.739

59POPC H13S 7847 0.980 0.185 3.625

59POPC C214 7848 1.101 0.046 3.736

59POPC H14R 7849 1.028 6.365 3.717

59POPC H14S 7850 1.145 0.032 3.837

59POPC C215 7851 1.207 0.042 3.628

59POPC H15R 7852 1.274 0.130 3.639

59POPC H15S 7853 1.156 0.051 3.530

59POPC C216 7854 1.294 6.318 3.629

59POPC H16R 7855 1.235 6.226 3.609

59POPC H16S 7856 1.339 6.307 3.730

59POPC C217 7857 1.404 6.335 3.525

59POPC H17R 7858 1.482 6.257 3.538

59POPC H17S 7859 1.452 0.034 3.544

59POPC C218 7860 1.354 6.333 3.381

59POPC H18R 7861 1.313 6.233 3.356

59POPC H18S 7862 1.437 6.358 3.311

59POPC H18T 7863 1.273 0.007 3.363

59POPC C33 7864 1.396 6.119 4.344

59POPC H3X 7865 1.322 6.093 4.423

59POPC H3Y 7866 1.481 6.048 4.355

59POPC C34 7867 1.323 6.094 4.206

59POPC H4X 7868 1.393 6.039 4.140

59POPC H4Y 7869 1.309 6.194 4.159

59POPC C35 7870 1.179 6.032 4.196

59POPC H5X 7871 1.119 6.058 4.286

59POPC H5Y 7872 1.182 5.921 4.195

59POPC C36 7873 1.091 6.068 4.072

59POPC H6X 7874 1.048 6.170 4.084

59POPC H6Y 7875 1.005 5.997 4.069

59POPC C37 7876 1.160 6.062 3.934

59POPC H7X 7877 1.247 5.992 3.941

59POPC H7Y 7878 1.197 6.164 3.911

59POPC C38 7879 1.079 6.016 3.812

59POPC H8X 7880 1.051 5.910 3.823

59POPC H8Y 7881 1.146 6.023 3.724

59POPC C39 7882 0.954 6.099 3.784

59POPC H9X 7883 0.972 6.206 3.811

59POPC H9Y 7884 0.873 6.063 3.852

59POPC C310 7885 0.904 6.087 3.638

59POPC H10X 7886 0.800 6.126 3.633

59POPC H10Y 7887 0.905 5.978 3.615

59POPC C311 7888 0.986 6.152 3.527

59POPC H11X 7889 1.084 6.101 3.518

59POPC H11Y 7890 1.005 6.260 3.549

59POPC C312 7891 0.915 6.146 3.391

59POPC H12X 7892 0.822 6.206 3.397

59POPC H12Y 7893 0.887 6.041 3.367

59POPC C313 7894 1.003 6.202 3.279

59POPC H13X 7895 1.098 6.144 3.272

59POPC H13Y 7896 1.029 6.306 3.307

59POPC C314 7897 0.933 6.200 3.143

59POPC H14X 7898 0.836 6.252 3.154

59POPC H14Y 7899 0.912 6.095 3.113

59POPC C315 7900 1.013 6.270 3.034

59POPC H15X 7901 0.952 6.269 2.941

59POPC H15Y 7902 1.107 6.215 3.011

59POPC C316 7903 1.046 0.014 3.072

59POPC H16X 7904 1.044 0.081 2.984

59POPC H16Y 7905 1.144 0.023 3.124

59POPC H16Z 7906 0.966 0.052 3.140

60POPC N 7907 5.148 6.272 4.915

60POPC C12 7908 5.209 6.225 5.046

60POPC H12A 7909 5.204 6.117 5.049

60POPC H12B 7910 5.149 6.263 5.127

60POPC C13 7911 5.244 6.236 4.806

60POPC H13A 7912 5.276 6.133 4.813

60POPC H13B 7913 5.202 6.253 4.708

60POPC H13C 7914 5.333 6.297 4.814

60POPC C14 7915 5.013 6.211 4.890

60POPC H14A 7916 5.019 6.103 4.884

60POPC H14B 7917 4.944 6.238 4.968

60POPC H14C 7918 4.970 6.248 4.798

60POPC C15 7919 5.126 0.021 4.918

60POPC H15A 7920 5.054 0.050 4.994

60POPC H15B 7921 5.092 0.060 4.823

60POPC H15C 7922 5.217 0.074 4.942

60POPC C11 7923 5.357 6.263 5.075

60POPC H11A 7924 5.425 6.225 4.995

60POPC H11B 7925 5.389 6.209 5.167

60POPC P 7926 5.459 0.101 5.010

60POPC O13 7927 5.582 0.132 5.086

60POPC O14 7928 5.373 0.215 4.973

60POPC O12 7929 5.371 0.003 5.099

60POPC O11 7930 5.487 0.018 4.878

60POPC C1 7931 5.547 0.075 4.759

60POPC HA 7932 5.597 6.394 4.703

60POPC HB 7933 5.626 0.148 4.786

60POPC C2 7934 5.442 0.140 4.665

60POPC HS 7935 5.387 0.215 4.726

60POPC O21 7936 5.361 0.034 4.616

60POPC C21 7937 5.237 0.069 4.603

60POPC O22 7938 5.181 0.163 4.656

60POPC C22 7939 5.173 6.374 4.506

60POPC H2R 7940 5.080 6.335 4.552

60POPC H2S 7941 5.240 6.289 4.480

60POPC C3 7942 5.499 0.217 4.541

60POPC HX 7943 5.550 0.142 4.476

60POPC HY 7944 5.579 0.284 4.580

60POPC O31 7945 5.403 0.302 4.472

60POPC C31 7946 5.392 0.282 4.339

60POPC O32 7947 5.429 0.182 4.281

60POPC C32 7948 5.338 0.408 4.270

60POPC H2X 7949 5.414 0.488 4.286

60POPC H2Y 7950 5.243 0.439 4.320

60POPC C23 7951 5.144 0.057 4.382

60POPC H3R 7952 5.233 0.057 4.316

60POPC H3S 7953 5.122 0.164 4.405

60POPC C24 7954 5.022 0.006 4.310

60POPC H4R 7955 4.987 0.090 4.248

60POPC H4S 7956 4.942 6.377 4.382

60POPC C25 7957 5.056 6.288 4.222

60POPC H5R 7958 4.963 6.238 4.190

60POPC H5S 7959 5.117 6.215 4.278

60POPC C26 7960 5.132 6.336 4.099

60POPC H6R 7961 5.229 6.380 4.130

60POPC H6S 7962 5.076 0.014 4.044

60POPC C27 7963 5.165 6.221 4.006

60POPC H7R 7964 5.074 6.183 3.955

60POPC H7S 7965 5.205 6.139 4.069

60POPC C28 7966 5.268 6.269 3.906

60POPC H8R 7967 5.348 6.328 3.956

60POPC H8S 7968 5.220 6.336 3.831

60POPC C29 7969 5.328 6.146 3.849

60POPC H91 7970 5.374 6.081 3.924

60POPC C210 7971 5.324 6.108 3.722

60POPC H101 7972 5.383 6.020 3.693

60POPC C211 7973 5.267 6.185 3.607

60POPC H11R 7974 5.193 6.261 3.637

60POPC H11S 7975 5.219 6.118 3.532

60POPC C212 7976 5.392 6.250 3.550

60POPC H12R 7977 5.463 6.173 3.514

60POPC H12S 7978 5.444 6.310 3.628

60POPC C213 7979 5.360 6.343 3.435

60POPC H13R 7980 5.290 0.022 3.467

60POPC H13S 7981 5.313 6.284 3.354

60POPC C214 7982 5.492 0.002 3.390

60POPC H14R 7983 5.556 6.319 3.352

60POPC H14S 7984 5.541 0.045 3.480

60POPC C215 7985 5.479 0.110 3.286

60POPC H15R 7986 5.423 0.195 3.331

60POPC H15S 7987 5.416 0.071 3.203

60POPC C216 7988 5.616 0.155 3.237

60POPC H16R 7989 5.675 0.065 3.211

60POPC H16S 7990 5.669 0.204 3.323

60POPC C217 7991 5.615 0.251 3.118

60POPC H17R 7992 5.719 0.260 3.079

60POPC H17S 7993 5.584 0.351 3.154

60POPC C218 7994 5.522 0.210 3.005

60POPC H18R 7995 5.543 0.107 2.968

60POPC H18S 7996 5.535 0.278 2.918

60POPC H18T 7997 5.416 0.213 3.038

60POPC C33 7998 5.316 0.394 4.116

60POPC H3X 7999 5.402 0.345 4.066

60POPC H3Y 8000 5.310 0.495 4.071

60POPC C34 8001 5.190 0.316 4.077

60POPC H4X 8002 5.102 0.359 4.130

60POPC H4Y 8003 5.201 0.211 4.109

60POPC C35 8004 5.160 0.322 3.927

60POPC H5X 8005 5.152 0.428 3.894

60POPC H5Y 8006 5.062 0.273 3.907

60POPC C36 8007 5.267 0.251 3.844

60POPC H6X 8008 5.280 0.147 3.882

60POPC H6Y 8009 5.365 0.302 3.858

60POPC C37 8010 5.234 0.243 3.694

60POPC H7X 8011 5.150 0.171 3.682

60POPC H7Y 8012 5.322 0.200 3.642

60POPC C38 8013 5.195 0.376 3.627

60POPC H8X 8014 5.258 0.460 3.663

60POPC H8Y 8015 5.090 0.401 3.652

60POPC C39 8016 5.210 0.368 3.474

60POPC H9X 8017 5.313 0.334 3.451

60POPC H9Y 8018 5.195 0.467 3.426

60POPC C310 8019 5.111 0.267 3.417

60POPC H10X 8020 5.008 0.305 3.430

60POPC H10Y 8021 5.119 0.173 3.475

60POPC C311 8022 5.134 0.227 3.271

60POPC H11X 8023 5.239 0.200 3.251

60POPC H11Y 8024 5.106 0.311 3.203

60POPC C312 8025 5.046 0.105 3.248

60POPC H12X 8026 4.944 0.144 3.267

60POPC H12Y 8027 5.065 0.028 3.325

60POPC C313 8028 5.057 0.041 3.110

60POPC H13X 8029 5.059 0.118 3.030

60POPC H13Y 8030 4.965 6.380 3.094

60POPC C314 8031 5.173 6.343 3.103

60POPC H14X 8032 5.182 6.296 3.203

60POPC H14Y 8033 5.268 6.396 3.080

60POPC C315 8034 5.150 6.228 3.005

60POPC H15X 8035 5.043 6.201 3.001

60POPC H15Y 8036 5.199 6.138 3.047

60POPC C316 8037 5.208 6.257 2.867

60POPC H16X 8038 5.316 6.283 2.874

60POPC H16Y 8039 5.154 6.343 2.822

60POPC H16Z 8040 5.198 6.171 2.798

61POPC N 8041 6.273 1.176 4.891

61POPC C12 8042 6.221 1.065 4.801

61POPC H12A 8043 6.302 1.037 4.734

61POPC H12B 8044 6.145 1.104 4.736

61POPC C13 8045 6.162 1.230 4.975

61POPC H13A 8046 6.123 1.155 5.044

61POPC H13B 8047 6.194 1.314 5.034

61POPC H13C 8048 6.080 1.264 4.914

61POPC C14 8049 6.381 1.124 4.981

61POPC H14A 8050 6.399 1.192 5.063

61POPC H14B 8051 6.352 1.029 5.021

61POPC H14C 8052 0.073 1.109 4.925

61POPC C15 8053 6.327 1.289 4.809

61POPC H15A 8054 6.256 1.322 4.735

61POPC H15B 8055 0.017 1.261 4.757

61POPC H15C 8056 6.348 1.375 4.871

61POPC C11 8057 6.165 0.934 4.866

61POPC H11A 8058 6.121 0.875 4.783

61POPC H11B 8059 6.083 0.960 4.936

61POPC P 8060 6.258 0.698 4.944

61POPC O13 8061 6.122 0.650 4.914

61POPC O14 8062 6.316 0.659 5.074

61POPC O12 8063 6.266 0.858 4.935

61POPC O11 8064 6.357 0.646 4.833

61POPC C1 8065 0.071 0.721 4.792

61POPC HA 8066 0.147 0.649 4.754

61POPC HB 8067 0.118 0.776 4.876

61POPC C2 8068 0.038 0.818 4.676

61POPC HS 8069 6.359 0.887 4.714

61POPC O21 8070 6.391 0.740 4.565

61POPC C21 8071 6.275 0.776 4.514

61POPC O22 8072 6.217 0.881 4.536

61POPC C22 8073 6.217 0.659 4.434

61POPC H2R 8074 6.130 0.623 4.492

61POPC H2S 8075 6.289 0.575 4.427

61POPC C3 8076 0.162 0.907 4.646

61POPC HX 8077 0.250 0.840 4.643

61POPC HY 8078 0.180 0.974 4.733

61POPC O31 8079 0.161 0.976 4.519

61POPC C31 8080 0.167 1.108 4.527

61POPC O32 8081 0.124 1.175 4.620

61POPC C32 8082 0.236 1.163 4.403

61POPC H2X 8083 0.339 1.120 4.400

61POPC H2Y 8084 0.246 1.273 4.415

61POPC C23 8085 6.167 0.701 4.294

61POPC H3R 8086 6.110 0.796 4.303

61POPC H3S 8087 6.099 0.623 4.255

61POPC C24 8088 6.278 0.720 4.189

61POPC H4R 8089 6.320 0.620 4.163

61POPC H4S 8090 6.359 0.781 4.234

61POPC C25 8091 6.230 0.790 4.061

61POPC H5R 8092 6.137 0.741 4.025

61POPC H5S 8093 6.307 0.777 3.982

61POPC C26 8094 6.207 0.941 4.081

61POPC H6R 8095 6.299 0.980 4.129

61POPC H6S 8096 6.122 0.958 4.151

61POPC C27 8097 6.186 1.020 3.951

61POPC H7R 8098 6.255 0.982 3.872

61POPC H7S 8099 6.217 1.126 3.971

61POPC C28 8100 6.042 1.026 3.896

61POPC H8R 8101 6.040 1.102 3.816

61POPC H8S 8102 5.973 1.062 3.975

61POPC C29 8103 5.993 0.894 3.843

61POPC H91 8104 5.943 0.830 3.918

61POPC C210 8105 6.004 0.848 3.718

61POPC H101 8106 5.959 0.751 3.693

61POPC C211 8107 6.075 0.917 3.604

61POPC H11R 8108 6.162 0.855 3.574

61POPC H11S 8109 6.115 1.016 3.634

61POPC C212 8110 5.990 0.936 3.479

61POPC H12R 8111 5.898 0.994 3.502

61POPC H12S 8112 5.960 0.837 3.440

61POPC C213 8113 6.067 1.010 3.369

61POPC H13R 8114 6.026 0.977 3.271

61POPC H13S 8115 6.172 0.975 3.374

61POPC C214 8116 6.060 1.164 3.373

61POPC H14R 8117 6.095 1.197 3.473

61POPC H14S 8118 5.954 1.195 3.362

61POPC C215 8119 6.144 1.235 3.265

61POPC H15R 8120 6.250 1.204 3.281

61POPC H15S 8121 6.140 1.345 3.281

61POPC C216 8122 6.101 1.205 3.120

61POPC H16R 8123 6.006 1.259 3.100

61POPC H16S 8124 6.080 1.097 3.111

61POPC C217 8125 6.201 1.237 3.007

61POPC H17R 8126 6.148 1.227 2.910

61POPC H17S 8127 6.280 1.159 3.006

61POPC C218 8128 6.271 1.372 3.012

61POPC H18R 8129 6.196 1.454 3.009

61POPC H18S 8130 6.340 1.383 2.926

61POPC H18T 8131 6.331 1.381 3.105

61POPC C33 8132 0.161 1.127 4.274

61POPC H3X 8133 0.055 1.159 4.282

61POPC H3Y 8134 0.162 1.016 4.261

61POPC C34 8135 0.220 1.191 4.148

61POPC H4X 8136 0.326 1.159 4.137

61POPC H4Y 8137 0.218 1.301 4.157

61POPC C35 8138 0.140 1.146 4.026

61POPC H5X 8139 0.033 1.174 4.040

61POPC H5Y 8140 0.145 1.035 4.024

61POPC C36 8141 0.187 1.204 3.893

61POPC H6X 8142 0.294 1.176 3.877

61POPC H6Y 8143 0.182 1.315 3.896

61POPC C37 8144 0.104 1.152 3.774

61POPC H7X 8145 0.141 1.199 3.680

61POPC H7Y 8146 6.398 1.181 3.787

61POPC C38 8147 0.114 0.999 3.761

61POPC H8X 8148 0.076 0.949 3.852

61POPC H8Y 8149 0.221 0.972 3.751

61POPC C39 8150 0.037 0.940 3.641

61POPC H9X 8151 0.062 1.000 3.551

61POPC H9Y 8152 6.328 0.948 3.661

61POPC C310 8153 0.082 0.795 3.623

61POPC H10X 8154 0.078 0.744 3.721

61POPC H10Y 8155 0.188 0.805 3.591

61POPC C311 8156 0.015 0.704 3.518

61POPC H11X 8157 0.028 0.746 3.416

61POPC H11Y 8158 6.305 0.704 3.534

61POPC C312 8159 0.074 0.562 3.531

61POPC H12X 8160 6.397 0.501 3.584

61POPC H12Y 8161 0.163 0.567 3.597

61POPC C313 8162 0.117 0.480 3.407

61POPC H13X 8163 0.159 0.384 3.443

61POPC H13Y 8164 0.199 0.532 3.353

61POPC C314 8165 0.006 0.449 3.308

61POPC H14X 8166 6.360 0.543 3.271

61POPC H14Y 8167 6.329 0.390 3.364

61POPC C315 8168 0.047 0.359 3.192

61POPC H15X 8169 0.156 0.369 3.171

61POPC H15Y 8170 6.393 0.391 3.100

61POPC C316 8171 0.011 0.214 3.221

61POPC H16X 8172 6.301 0.203 3.233

61POPC H16Y 8173 0.059 0.179 3.315

61POPC H16Z 8174 0.043 0.146 3.139

62POPC N 8175 0.271 0.457 5.030

62POPC C12 8176 0.298 0.443 4.881

62POPC H12A 8177 0.404 0.445 4.864

62POPC H12B 8178 0.256 0.528 4.829

62POPC C13 8179 0.125 0.437 5.058

62POPC H13A 8180 0.097 0.337 5.030

62POPC H13B 8181 0.104 0.452 5.163

62POPC H13C 8182 0.064 0.507 5.004

62POPC C14 8183 0.350 0.354 5.105

62POPC H14A 8184 0.328 0.255 5.067

62POPC H14B 8185 0.456 0.367 5.090

62POPC H14C 8186 0.331 0.355 5.212

62POPC C15 8187 0.311 0.593 5.076

62POPC H15A 8188 0.251 0.669 5.028

62POPC H15B 8189 0.415 0.613 5.055

62POPC H15C 8190 0.298 0.603 5.183

62POPC C11 8191 0.246 0.316 4.809

62POPC H11A 8192 0.263 0.326 4.700

62POPC H11B 8193 0.136 0.311 4.822

62POPC P 8194 0.458 0.166 4.826

62POPC O13 8195 0.474 0.062 4.722

62POPC O14 8196 0.525 0.138 4.955

62POPC O12 8197 0.305 0.195 4.858

62POPC O11 8198 0.504 0.307 4.772

62POPC C1 8199 0.637 0.351 4.761

62POPC HA 8200 0.710 0.269 4.779

62POPC HB 8201 0.659 0.428 4.839

62POPC C2 8202 0.665 0.405 4.618

62POPC HS 8203 0.775 0.419 4.615

62POPC O21 8204 0.607 0.528 4.577

62POPC C21 8205 0.574 0.622 4.657

62POPC O22 8206 0.620 0.648 4.767

62POPC C22 8207 0.453 0.693 4.595

62POPC H2R 8208 0.483 0.795 4.563

62POPC H2S 8209 0.381 0.699 4.680

62POPC C3 8210 0.624 0.303 4.508

62POPC HX 8211 0.657 0.202 4.541

62POPC HY 8212 0.684 0.331 4.419

62POPC O31 8213 0.484 0.297 4.476

62POPC C31 8214 0.453 0.333 4.352

62POPC O32 8215 0.523 0.398 4.276

62POPC C32 8216 0.329 0.259 4.311

62POPC H2X 8217 0.285 0.314 4.225

62POPC H2Y 8218 0.256 0.254 4.395

62POPC C23 8219 0.388 0.614 4.478

62POPC H3R 8220 0.379 0.509 4.511

62POPC H3S 8221 0.453 0.619 4.388

62POPC C24 8222 0.246 0.656 4.444

62POPC H4R 8223 0.242 0.765 4.422

62POPC H4S 8224 0.188 0.636 4.537

62POPC C25 8225 0.176 0.578 4.332

62POPC H5R 8226 0.070 0.611 4.331

62POPC H5S 8227 0.174 0.470 4.358

62POPC C26 8228 0.232 0.598 4.191

62POPC H6R 8229 0.166 0.546 4.118

62POPC H6S 8230 0.331 0.548 4.187

62POPC C27 8231 0.248 0.745 4.149

62POPC H7R 8232 0.318 0.796 4.218

62POPC H7S 8233 0.150 0.797 4.157

62POPC C28 8234 0.299 0.763 4.005

62POPC H8R 8235 0.311 0.871 3.982

62POPC H8S 8236 0.223 0.723 3.934

62POPC C29 8237 0.431 0.691 3.995

62POPC H91 8238 0.509 0.730 4.062

62POPC C210 8239 0.458 0.582 3.922

62POPC H101 8240 0.557 0.535 3.934

62POPC C211 8241 0.368 0.509 3.826

62POPC H11R 8242 0.264 0.549 3.826

62POPC H11S 8243 0.362 0.403 3.859

62POPC C212 8244 0.423 0.513 3.681

62POPC H12R 8245 0.357 0.448 3.620

62POPC H12S 8246 0.525 0.470 3.676

62POPC C213 8247 0.422 0.655 3.621

62POPC H13R 8248 0.506 0.712 3.666

62POPC H13S 8249 0.329 0.703 3.659

62POPC C214 8250 0.431 0.658 3.467

62POPC H14R 8251 0.383 0.566 3.427

62POPC H14S 8252 0.540 0.653 3.446

62POPC C215 8253 0.385 0.781 3.388

62POPC H15R 8254 0.474 0.829 3.343

62POPC H15S 8255 0.340 0.854 3.458

62POPC C216 8256 0.285 0.760 3.272

62POPC H16R 8257 0.273 0.860 3.223

62POPC H16S 8258 0.186 0.732 3.314

62POPC C217 8259 0.313 0.660 3.159

62POPC H17R 8260 0.329 0.559 3.201

62POPC H17S 8261 0.408 0.691 3.109

62POPC C218 8262 0.201 0.653 3.055

62POPC H18R 8263 0.221 0.573 2.981

62POPC H18S 8264 0.191 0.750 3.001

62POPC H18T 8265 0.104 0.631 3.105

62POPC C33 8266 0.378 0.119 4.273

62POPC H3X 8267 0.396 0.065 4.368

62POPC H3Y 8268 0.477 0.124 4.223

62POPC C34 8269 0.290 0.040 4.178

62POPC H4X 8270 0.196 0.006 4.227

62POPC H4Y 8271 0.348 6.351 4.148

62POPC C35 8272 0.247 0.109 4.051

62POPC H5X 8273 0.200 0.207 4.073

62POPC H5Y 8274 0.171 0.044 4.004

62POPC C36 8275 0.361 0.125 3.953

62POPC H6X 8276 0.423 0.032 3.954

62POPC H6Y 8277 0.426 0.210 3.982

62POPC C37 8278 0.305 0.146 3.814

62POPC H7X 8279 0.246 0.240 3.811

62POPC H7Y 8280 0.235 0.063 3.789

62POPC C38 8281 0.413 0.152 3.708

62POPC H8X 8282 0.480 0.237 3.732

62POPC H8Y 8283 0.362 0.174 3.612

62POPC C39 8284 0.491 0.021 3.697

62POPC H9X 8285 0.422 6.341 3.732

62POPC H9Y 8286 0.577 0.011 3.767

62POPC C310 8287 0.534 6.387 3.554

62POPC H10X 8288 0.450 0.018 3.489

62POPC H10Y 8289 0.549 6.278 3.543

62POPC C311 8290 0.658 0.060 3.505

62POPC H11X 8291 0.664 0.051 3.395

62POPC H11Y 8292 0.750 0.012 3.544

62POPC C312 8293 0.654 0.209 3.539

62POPC H12X 8294 0.675 0.225 3.647

62POPC H12Y 8295 0.553 0.250 3.518

62POPC C313 8296 0.753 0.282 3.455

62POPC H13X 8297 0.843 0.217 3.445

62POPC H13Y 8298 0.785 0.377 3.504

62POPC C314 8299 0.692 0.313 3.319

62POPC H14X 8300 0.642 0.224 3.274

62POPC H14Y 8301 0.773 0.346 3.251

62POPC C315 8302 0.594 0.428 3.327

62POPC H15X 8303 0.630 0.487 3.414

62POPC H15Y 8304 0.492 0.392 3.349

62POPC C316 8305 0.595 0.517 3.203

62POPC H16X 8306 0.567 0.621 3.229

62POPC H16Y 8307 0.520 0.480 3.131

62POPC H16Z 8308 0.695 0.521 3.155

63POPC N 8309 0.187 5.861 4.858

63POPC C12 8310 0.172 5.891 5.006

63POPC H12A 8311 0.236 5.973 5.034

63POPC H12B 8312 0.204 5.804 5.062

63POPC C13 8313 0.095 5.747 4.827

63POPC H13A 8314 6.393 5.773 4.854

63POPC H13B 8315 0.092 5.725 4.721

63POPC H13C 8316 0.117 5.656 4.881

63POPC C14 8317 0.144 5.979 4.777

63POPC H14A 8318 0.053 6.019 4.819

63POPC H14B 8319 0.216 6.059 4.778

63POPC H14C 8320 0.120 5.950 4.676

63POPC C15 8321 0.325 5.819 4.820

63POPC H15A 8322 0.349 5.725 4.868

63POPC H15B 8323 0.397 5.894 4.848

63POPC H15C 8324 0.329 5.804 4.713

63POPC C11 8325 0.028 5.927 5.056

63POPC H11A 8326 0.024 5.918 5.166

63POPC H11B 8327 6.355 5.851 5.020

63POPC P 8328 6.386 6.179 5.108

63POPC O13 8329 0.121 6.192 5.168

63POPC O14 8330 6.274 6.170 5.204

63POPC O12 8331 6.388 6.056 5.010

63POPC O11 8332 6.365 6.301 5.011

63POPC C1 8333 0.059 6.318 4.904

63POPC HA 8334 0.128 6.233 4.887

63POPC HB 8335 0.122 0.006 4.928

63POPC C2 8336 6.387 6.347 4.770

63POPC HS 8337 6.298 0.008 4.796

63POPC O21 8338 6.351 6.227 4.697

63POPC C21 8339 6.267 6.260 4.598

63POPC O22 8340 6.217 6.370 4.587

63POPC C22 8341 6.252 6.148 4.494

63POPC H2R 8342 6.163 6.170 4.431

63POPC H2S 8343 6.227 6.053 4.547

63POPC C3 8344 0.085 0.031 4.684

63POPC HX 8345 0.113 0.119 4.745

63POPC HY 8346 0.033 0.074 4.596

63POPC O31 8347 0.204 6.351 4.658

63POPC C31 8348 0.219 6.322 4.529

63POPC O32 8349 0.152 6.371 4.441

63POPC C32 8350 0.320 6.207 4.508

63POPC H2X 8351 0.274 6.115 4.548

63POPC H2Y 8352 0.413 6.231 4.564

63POPC C23 8353 6.375 6.125 4.405

63POPC H3R 8354 0.067 6.138 4.467

63POPC H3S 8355 6.377 6.198 4.322

63POPC C24 8356 6.384 5.981 4.354

63POPC H4R 8357 6.282 5.938 4.356

63POPC H4S 8358 0.047 5.923 4.425

63POPC C25 8359 0.045 5.965 4.216

63POPC H5R 8360 0.150 6.002 4.220

63POPC H5S 8361 6.389 6.027 4.143

63POPC C26 8362 0.044 5.817 4.173

63POPC H6R 8363 6.341 5.792 4.138

63POPC H6S 8364 0.065 5.755 4.263

63POPC C27 8365 0.145 5.777 4.065

63POPC H7R 8366 0.137 5.669 4.041

63POPC H7S 8367 0.244 5.795 4.112

63POPC C28 8368 0.140 5.862 3.937

63POPC H8R 8369 0.245 5.870 3.903

63POPC H8S 8370 0.111 5.964 3.970

63POPC C29 8371 0.060 5.822 3.817

63POPC H91 8372 0.104 5.744 3.753

63POPC C210 8373 6.354 5.891 3.771

63POPC H101 8374 6.306 5.859 3.678

63POPC C211 8375 6.284 6.001 3.846

63POPC H11R 8376 6.297 5.992 3.956

63POPC H11S 8377 6.176 5.988 3.826

63POPC C212 8378 6.307 6.147 3.807

63POPC H12R 8379 0.002 6.188 3.847

63POPC H12S 8380 6.221 6.202 3.852

63POPC C213 8381 6.298 6.169 3.657

63POPC H13R 8382 6.216 6.103 3.620

63POPC H13S 8383 6.391 6.132 3.609

63POPC C214 8384 6.262 6.312 3.617

63POPC H14R 8385 6.346 6.381 3.638

63POPC H14S 8386 6.176 6.346 3.680

63POPC C215 8387 6.217 6.326 3.471

63POPC H15R 8388 6.240 0.028 3.435

63POPC H15S 8389 6.107 6.314 3.470

63POPC C216 8390 6.270 6.221 3.373

63POPC H16R 8391 6.194 6.140 3.364

63POPC H16S 8392 6.359 6.167 3.414

63POPC C217 8393 6.314 6.281 3.239

63POPC H17R 8394 6.383 6.365 3.261

63POPC H17S 8395 6.228 6.325 3.184

63POPC C218 8396 6.385 6.183 3.147

63POPC H18R 8397 6.315 6.110 3.101

63POPC H18S 8398 0.064 6.127 3.201

63POPC H18T 8399 0.033 6.242 3.065

63POPC C33 8400 0.349 6.190 4.356

63POPC H3X 8401 0.419 6.272 4.329

63POPC H3Y 8402 0.254 6.202 4.299

63POPC C34 8403 0.418 6.066 4.299

63POPC H4X 8404 0.351 5.977 4.306

63POPC H4Y 8405 0.509 6.044 4.359

63POPC C35 8406 0.461 6.091 4.151

63POPC H5X 8407 0.527 6.006 4.121

63POPC H5Y 8408 0.522 6.184 4.146

63POPC C36 8409 0.345 6.101 4.049

63POPC H6X 8410 0.274 6.183 4.075

63POPC H6Y 8411 0.289 6.007 4.063

63POPC C37 8412 0.384 6.115 3.899

63POPC H7X 8413 0.427 6.018 3.867

63POPC H7Y 8414 0.463 6.193 3.889

63POPC C38 8415 0.266 6.152 3.805

63POPC H8X 8416 0.232 6.255 3.833

63POPC H8Y 8417 0.179 6.085 3.824

63POPC C39 8418 0.289 6.146 3.652

63POPC H9X 8419 0.247 6.051 3.614

63POPC H9Y 8420 0.398 6.145 3.631

63POPC C310 8421 0.224 6.264 3.575

63POPC H10X 8422 0.276 6.361 3.587

63POPC H10Y 8423 0.127 6.281 3.625

63POPC C311 8424 0.194 6.245 3.425

63POPC H11X 8425 0.114 6.320 3.405

63POPC H11Y 8426 0.156 6.143 3.407

63POPC C312 8427 0.302 6.275 3.319

63POPC H12X 8428 0.344 6.376 3.333

63POPC H12Y 8429 0.254 6.278 3.218

63POPC C313 8430 0.408 6.165 3.317

63POPC H13X 8431 0.370 6.080 3.256

63POPC H13Y 8432 0.416 6.129 3.422

63POPC C314 8433 0.548 6.208 3.270

63POPC H14X 8434 0.631 6.149 3.315

63POPC H14Y 8435 0.560 6.314 3.301

63POPC C315 8436 0.562 6.205 3.120

63POPC H15X 8437 0.626 6.289 3.086

63POPC H15Y 8438 0.457 6.224 3.091

63POPC C316 8439 0.607 6.075 3.054

63POPC H16X 8440 0.629 6.092 2.946

63POPC H16Y 8441 0.524 6.001 3.060

63POPC H16Z 8442 0.697 6.033 3.102

64POPC N 8443 6.145 0.257 5.049

64POPC C12 8444 6.110 0.116 5.098

64POPC H12A 8445 6.172 0.088 5.182

64POPC H12B 8446 6.138 0.046 5.019

64POPC C13 8447 6.025 0.329 4.996

64POPC H13A 8448 5.978 0.267 4.921

64POPC H13B 8449 5.952 0.341 5.073

64POPC H13C 8450 6.051 0.426 4.955

64POPC C14 8451 6.203 0.340 5.159

64POPC H14A 8452 6.291 0.294 5.201

64POPC H14B 8453 6.227 0.439 5.125

64POPC H14C 8454 6.131 0.351 5.239

64POPC C15 8455 6.242 0.240 4.936

64POPC H15A 8456 6.330 0.185 4.968

64POPC H15B 8457 6.269 0.335 4.893

64POPC H15C 8458 6.196 0.180 4.859

64POPC C11 8459 5.962 0.080 5.128

64POPC H11A 8460 5.926 0.146 5.210

64POPC H11B 8461 5.958 6.376 5.166

64POPC P 8462 5.894 6.396 4.891

64POPC O13 8463 5.800 6.283 4.908

64POPC O14 8464 6.038 6.364 4.878

64POPC O12 8465 5.877 0.095 5.014

64POPC O11 8466 5.851 0.091 4.771

64POPC C1 8467 5.946 0.152 4.683

64POPC HA 8468 6.049 0.110 4.694

64POPC HB 8469 5.953 0.261 4.704

64POPC C2 8470 5.907 0.132 4.534

64POPC HS 8471 5.830 0.052 4.530

64POPC O21 8472 6.024 0.099 4.457

64POPC C21 8473 6.005 0.011 4.360

64POPC O22 8474 5.900 6.359 4.329

64POPC C22 8475 6.137 6.385 4.289

64POPC H2R 8476 6.132 6.283 4.245

64POPC H2S 8477 6.221 6.389 4.362

64POPC C3 8478 5.851 0.262 4.474

64POPC HX 8479 5.932 0.338 4.473

64POPC HY 8480 5.771 0.298 4.542

64POPC O31 8481 5.806 0.239 4.340

64POPC C31 8482 5.714 0.319 4.295

64POPC O32 8483 5.660 0.408 4.360

64POPC C32 8484 5.686 0.291 4.148

64POPC H2X 8485 5.744 0.363 4.085

64POPC H2Y 8486 5.578 0.310 4.131

64POPC C23 8487 6.173 0.086 4.181

64POPC H3R 8488 6.169 0.191 4.218

64POPC H3S 8489 6.098 0.075 4.099

64POPC C24 8490 6.310 0.056 4.120

64POPC H4R 8491 6.352 6.365 4.168

64POPC H4S 8492 6.380 0.139 4.141

64POPC C25 8493 6.294 0.030 3.971

64POPC H5R 8494 6.207 6.361 3.960

64POPC H5S 8495 6.384 6.381 3.929

64POPC C26 8496 6.263 0.157 3.892

64POPC H6R 8497 6.199 0.224 3.954

64POPC H6S 8498 6.207 0.132 3.799

64POPC C27 8499 6.392 0.228 3.854

64POPC H7R 8500 0.047 0.160 3.786

64POPC H7S 8501 0.052 0.244 3.946

64POPC C28 8502 6.374 0.362 3.786

64POPC H8R 8503 6.328 0.341 3.687

64POPC H8S 8504 0.075 0.405 3.766

64POPC C29 8505 6.292 0.458 3.868

64POPC H91 8506 6.345 0.505 3.953

64POPC C210 8507 6.162 0.485 3.847

64POPC H101 8508 6.110 0.559 3.910

64POPC C211 8509 6.082 0.428 3.732

64POPC H11R 8510 5.973 0.423 3.755

64POPC H11S 8511 6.114 0.324 3.710

64POPC C212 8512 6.105 0.513 3.606

64POPC H12R 8513 6.205 0.561 3.616

64POPC H12S 8514 6.030 0.595 3.597

64POPC C213 8515 6.112 0.437 3.474

64POPC H13R 8516 6.164 0.339 3.487

64POPC H13S 8517 6.172 0.501 3.407

64POPC C214 8518 5.980 0.421 3.403

64POPC H14R 8519 5.937 0.522 3.391

64POPC H14S 8520 5.912 0.359 3.465

64POPC C215 8521 5.994 0.358 3.263

64POPC H15R 8522 5.893 0.333 3.225

64POPC H15S 8523 6.053 0.264 3.271

64POPC C216 8524 6.062 0.448 3.160

64POPC H16R 8525 6.172 0.447 3.178

64POPC H16S 8526 6.027 0.553 3.171

64POPC C217 8527 6.035 0.401 3.017

64POPC H17R 8528 6.107 0.453 2.951

64POPC H17S 8529 5.933 0.434 2.988

64POPC C218 8530 6.044 0.250 2.995

64POPC H18R 8531 5.958 0.199 3.043

64POPC H18S 8532 6.139 0.210 3.038

64POPC H18T 8533 6.041 0.226 2.887

64POPC C33 8534 5.718 0.148 4.102

64POPC H3X 8535 5.650 0.119 4.019

64POPC H3Y 8536 5.699 0.075 4.184

64POPC C34 8537 5.863 0.138 4.057

64POPC H4X 8538 5.891 0.030 4.054

64POPC H4Y 8539 5.927 0.187 4.134

64POPC C35 8540 5.893 0.199 3.921

64POPC H5X 8541 6.000 0.182 3.896

64POPC H5Y 8542 5.877 0.309 3.921

64POPC C36 8543 5.807 0.134 3.813

64POPC H6X 8544 5.716 0.195 3.796

64POPC H6Y 8545 5.774 0.035 3.851

64POPC C37 8546 5.880 0.108 3.682

64POPC H7X 8547 5.965 0.039 3.699

64POPC H7Y 8548 5.919 0.202 3.638

64POPC C38 8549 5.778 0.044 3.588

64POPC H8X 8550 5.712 0.124 3.550

64POPC H8Y 8551 5.716 6.371 3.644

64POPC C39 8552 5.839 6.371 3.469

64POPC H9X 8553 5.756 6.326 3.410

64POPC H9Y 8554 5.901 6.288 3.510

64POPC C310 8555 5.921 0.062 3.378

64POPC H10X 8556 6.011 0.098 3.433

64POPC H10Y 8557 5.862 0.154 3.357

64POPC C311 8558 5.964 6.399 3.243

64POPC H11X 8559 6.068 0.030 3.223

64POPC H11Y 8560 5.902 0.044 3.163

64POPC C312 8561 5.944 6.247 3.239

64POPC H12X 8562 5.836 6.228 3.222

64POPC H12Y 8563 5.970 6.205 3.339

64POPC C313 8564 6.024 6.156 3.144

64POPC H13X 8565 5.965 6.062 3.140

64POPC H13Y 8566 6.121 6.122 3.187

64POPC C314 8567 6.052 6.201 3.002

64POPC H14X 8568 6.161 6.207 2.981

64POPC H14Y 8569 6.013 6.303 2.983

64POPC C315 8570 5.989 6.093 2.913

64POPC H15X 8571 5.891 6.060 2.954

64POPC H15Y 8572 6.054 6.003 2.920

64POPC C316 8573 5.963 6.137 2.770

64POPC H16X 8574 5.967 6.049 2.703

64POPC H16Y 8575 6.035 6.214 2.736

64POPC H16Z 8576 5.859 6.176 2.763

65POPC N 8577 3.492 3.185 1.029

65POPC C12 8578 3.527 3.037 1.034

65POPC H12A 8579 3.628 3.024 0.997

65POPC H12B 8580 3.459 2.986 0.968

65POPC C13 8581 3.349 3.203 1.072

65POPC H13A 8582 3.334 3.159 1.169

65POPC H13B 8583 3.317 3.307 1.074

65POPC H13C 8584 3.283 3.146 1.007

65POPC C14 8585 3.580 3.261 1.123

65POPC H14A 8586 3.564 3.225 1.223

65POPC H14B 8587 3.685 3.246 1.101

65POPC H14C 8588 3.557 3.367 1.122

65POPC C15 8589 3.504 3.238 0.889

65POPC H15A 8590 3.467 3.339 0.885

65POPC H15B 8591 3.443 3.179 0.823

65POPC H15C 8592 3.607 3.235 0.856

65POPC C11 8593 3.515 2.964 1.170

65POPC H11A 8594 3.573 3.018 1.248

65POPC H11B 8595 3.561 2.863 1.162

65POPC P 8596 3.281 2.862 1.129

65POPC O13 8597 3.331 2.723 1.142

65POPC O14 8598 3.270 2.919 0.992

65POPC O12 8599 3.380 2.954 1.211

65POPC O11 8600 3.147 2.876 1.209

65POPC C1 8601 3.072 2.998 1.216

65POPC HA 8602 2.964 2.974 1.205

65POPC HB 8603 3.095 3.067 1.132

65POPC C2 8604 3.089 3.077 1.350

65POPC HS 8605 3.019 3.163 1.340

65POPC O21 8606 3.224 3.126 1.364

65POPC C21 8607 3.239 3.229 1.445

65POPC O22 8608 3.151 3.301 1.489

65POPC C22 8609 3.387 3.248 1.476

65POPC H2R 8610 3.405 3.357 1.478

65POPC H2S 8611 3.448 3.202 1.395

65POPC C3 8612 3.042 2.998 1.477

65POPC HX 8613 2.932 2.988 1.461

65POPC HY 8614 3.055 3.067 1.563

65POPC O31 8615 3.104 2.867 1.504

65POPC C31 8616 3.042 2.808 1.609

65POPC O32 8617 2.954 2.861 1.673

65POPC C32 8618 3.096 2.666 1.641

65POPC H2X 8619 3.203 2.677 1.668

65POPC H2Y 8620 3.090 2.608 1.547

65POPC C23 8621 3.424 3.187 1.614

65POPC H3R 8622 3.352 3.221 1.691

65POPC H3S 8623 3.525 3.224 1.644

65POPC C24 8624 3.429 3.033 1.612

65POPC H4R 8625 3.495 2.998 1.530

65POPC H4S 8626 3.327 2.997 1.588

65POPC C25 8627 3.470 2.973 1.748

65POPC H5R 8628 3.411 2.882 1.769

65POPC H5S 8629 3.442 3.051 1.822

65POPC C26 8630 3.620 2.944 1.772

65POPC H6R 8631 3.676 3.014 1.706

65POPC H6S 8632 3.644 2.840 1.741

65POPC C27 8633 3.667 2.970 1.917

65POPC H7R 8634 3.638 3.074 1.941

65POPC H7S 8635 3.778 2.963 1.925

65POPC C28 8636 3.606 2.882 2.029

65POPC H8R 8637 3.500 2.870 1.996

65POPC H8S 8638 3.607 2.945 2.120

65POPC C29 8639 3.656 2.745 2.064

65POPC H91 8640 3.656 2.674 1.980

65POPC C210 8641 3.688 2.697 2.187

65POPC H101 8642 3.720 2.592 2.193

65POPC C211 8643 3.699 2.770 2.321

65POPC H11R 8644 3.743 2.870 2.305

65POPC H11S 8645 3.774 2.713 2.380

65POPC C212 8646 3.578 2.790 2.417

65POPC H12R 8647 3.534 2.890 2.397

65POPC H12S 8648 3.615 2.800 2.521

65POPC C213 8649 3.469 2.681 2.415

65POPC H13R 8650 3.518 2.586 2.447

65POPC H13S 8651 3.440 2.661 2.310

65POPC C214 8652 3.341 2.700 2.501

65POPC H14R 8653 3.348 2.631 2.588

65POPC H14S 8654 3.253 2.670 2.440

65POPC C215 8655 3.303 2.830 2.572

65POPC H15R 8656 3.280 2.906 2.495

65POPC H15S 8657 3.389 2.864 2.633

65POPC C216 8658 3.179 2.811 2.663

65POPC H16R 8659 3.162 2.701 2.671

65POPC H16S 8660 3.088 2.853 2.617

65POPC C217 8661 3.192 2.860 2.809

65POPC H17R 8662 3.294 2.837 2.847

65POPC H17S 8663 3.122 2.798 2.869

65POPC C218 8664 3.154 3.005 2.840

65POPC H18R 8665 3.234 3.076 2.811

65POPC H18S 8666 3.139 3.018 2.950

65POPC H18T 8667 3.060 3.034 2.788

65POPC C33 8668 3.021 2.586 1.756

65POPC H3X 8669 3.058 2.482 1.753

65POPC H3Y 8670 2.912 2.580 1.736

65POPC C34 8671 3.047 2.643 1.897

65POPC H4X 8672 3.059 2.752 1.880

65POPC H4Y 8673 3.143 2.598 1.929

65POPC C35 8674 2.947 2.633 2.016

65POPC H5X 8675 2.955 2.528 2.053

65POPC H5Y 8676 2.841 2.642 1.982

65POPC C36 8677 2.971 2.727 2.141

65POPC H6X 8678 3.075 2.712 2.175

65POPC H6Y 8679 2.907 2.689 2.223

65POPC C37 8680 2.940 2.881 2.135

65POPC H7X 8681 2.832 2.899 2.152

65POPC H7Y 8682 2.964 2.920 2.034

65POPC C38 8683 3.009 2.971 2.243

65POPC H8X 8684 3.107 2.927 2.271

65POPC H8Y 8685 2.948 2.967 2.336

65POPC C39 8686 3.028 3.120 2.201

65POPC H9X 8687 2.927 3.164 2.189

65POPC H9Y 8688 3.067 3.123 2.097

65POPC C310 8689 3.117 3.215 2.289

65POPC H10X 8690 3.064 3.308 2.315

65POPC H10Y 8691 3.200 3.254 2.225

65POPC C311 8692 3.176 3.152 2.416

65POPC H11X 8693 3.245 3.073 2.379

65POPC H11Y 8694 3.098 3.097 2.475

65POPC C312 8695 3.264 3.238 2.509

65POPC H12X 8696 3.201 3.286 2.586

65POPC H12Y 8697 3.315 3.317 2.450

65POPC C313 8698 3.370 3.146 2.575

65POPC H13X 8699 3.428 3.096 2.495

65POPC H13Y 8700 3.312 3.069 2.630

65POPC C314 8701 3.471 3.205 2.673

65POPC H14X 8702 3.419 3.265 2.752

65POPC H14Y 8703 3.537 3.271 2.613

65POPC C315 8704 3.564 3.104 2.742

65POPC H15X 8705 3.666 3.147 2.740

65POPC H15Y 8706 3.568 3.010 2.682

65POPC C316 8707 3.529 3.074 2.888

65POPC H16X 8708 3.516 3.168 2.946

65POPC H16Y 8709 3.610 3.015 2.936

65POPC H16Z 8710 3.435 3.015 2.895

66POPC N 8711 2.444 3.768 1.069

66POPC C12 8712 2.453 3.622 1.027

66POPC H12A 8713 2.534 3.612 0.956

66POPC H12B 8714 2.362 3.591 0.978

66POPC C13 8715 2.580 3.816 1.111

66POPC H13A 8716 2.580 3.919 1.144

66POPC H13B 8717 2.616 3.752 1.191

66POPC H13C 8718 2.653 3.803 1.033

66POPC C14 8719 2.397 3.853 0.955

66POPC H14A 8720 2.300 3.823 0.920

66POPC H14B 8721 2.390 3.956 0.987

66POPC H14C 8722 2.467 3.849 0.873

66POPC C15 8723 2.353 3.782 1.186

66POPC H15A 8724 2.254 3.744 1.163

66POPC H15B 8725 2.343 3.885 1.217

66POPC H15C 8726 2.392 3.726 1.269

66POPC C11 8727 2.484 3.516 1.135

66POPC H11A 8728 2.478 3.413 1.092

66POPC H11B 8729 2.409 3.520 1.217

66POPC P 8730 2.744 3.503 1.108

66POPC O13 8731 2.773 3.616 1.016

66POPC O14 8732 2.729 3.373 1.041

66POPC O12 8733 2.613 3.539 1.189

66POPC O11 8734 2.849 3.502 1.226

66POPC C1 8735 2.850 3.608 1.322

66POPC HA 8736 2.777 3.689 1.300

66POPC HB 8737 2.951 3.656 1.319

66POPC C2 8738 2.825 3.563 1.470

66POPC HS 8739 2.903 3.488 1.495

66POPC O21 8740 2.689 3.512 1.486

66POPC C21 8741 2.680 3.377 1.504

66POPC O22 8742 2.775 3.302 1.511

66POPC C22 8743 2.532 3.333 1.528

66POPC H2R 8744 2.493 3.299 1.430

66POPC H2S 8745 2.473 3.422 1.558

66POPC C3 8746 2.853 3.688 1.562

66POPC HX 8747 2.783 3.769 1.531

66POPC HY 8748 2.956 3.725 1.538

66POPC O31 8749 2.846 3.659 1.704

66POPC C31 8750 2.825 3.762 1.787

66POPC O32 8751 2.813 3.878 1.753

66POPC C32 8752 2.807 3.706 1.929

66POPC H2X 8753 2.906 3.670 1.965

66POPC H2Y 8754 2.741 3.617 1.923

66POPC C23 8755 2.516 3.219 1.636

66POPC H3R 8756 2.607 3.217 1.698

66POPC H3S 8757 2.513 3.121 1.584

66POPC C24 8758 2.394 3.227 1.733

66POPC H4R 8759 2.316 3.156 1.697

66POPC H4S 8760 2.343 3.325 1.724

66POPC C25 8761 2.424 3.187 1.881

66POPC H5R 8762 2.533 3.180 1.896

66POPC H5S 8763 2.386 3.083 1.890

66POPC C26 8764 2.363 3.267 2.001

66POPC H6R 8765 2.340 3.192 2.080

66POPC H6S 8766 2.265 3.314 1.975

66POPC C27 8767 2.461 3.365 2.069

66POPC H7R 8768 2.482 3.455 2.007

66POPC H7S 8769 2.558 3.310 2.068

66POPC C28 8770 2.434 3.426 2.210

66POPC H8R 8771 2.380 3.521 2.192

66POPC H8S 8772 2.531 3.457 2.254

66POPC C29 8773 2.354 3.348 2.310

66POPC H91 8774 2.266 3.300 2.265

66POPC C210 8775 2.371 3.337 2.443

66POPC H101 8776 2.296 3.275 2.493

66POPC C211 8777 2.477 3.388 2.539

66POPC H11R 8778 2.451 3.489 2.579

66POPC H11S 8779 2.576 3.395 2.489

66POPC C212 8780 2.484 3.288 2.657

66POPC H12R 8781 2.519 3.192 2.615

66POPC H12S 8782 2.382 3.276 2.700

66POPC C213 8783 2.574 3.316 2.778

66POPC H13R 8784 2.572 3.226 2.843

66POPC H13S 8785 2.538 3.403 2.837

66POPC C214 8786 2.717 3.339 2.735

66POPC H14R 8787 2.737 3.448 2.736

66POPC H14S 8788 2.719 3.311 2.628

66POPC C215 8789 2.822 3.254 2.810

66POPC H15R 8790 2.920 3.267 2.759

66POPC H15S 8791 2.796 3.147 2.805

66POPC C216 8792 2.838 3.292 2.957

66POPC H16R 8793 2.932 3.247 2.996

66POPC H16S 8794 2.754 3.250 3.016

66POPC C217 8795 2.842 3.443 2.976

66POPC H17R 8796 2.863 3.462 3.083

66POPC H17S 8797 2.742 3.487 2.956

66POPC C218 8798 2.946 3.512 2.886

66POPC H18R 8799 3.045 3.463 2.899

66POPC H18S 8800 2.953 3.620 2.910

66POPC H18T 8801 2.922 3.505 2.778

66POPC C33 8802 2.741 3.799 2.035

66POPC H3X 8803 2.676 3.735 2.097

66POPC H3Y 8804 2.675 3.876 1.989

66POPC C34 8805 2.844 3.864 2.128

66POPC H4X 8806 2.861 3.969 2.097

66POPC H4Y 8807 2.942 3.812 2.118

66POPC C35 8808 2.805 3.859 2.278

66POPC H5X 8809 2.708 3.910 2.293

66POPC H5Y 8810 2.882 3.917 2.333

66POPC C36 8811 2.806 3.719 2.340

66POPC H6X 8812 2.869 3.719 2.431

66POPC H6Y 8813 2.855 3.649 2.268

66POPC C37 8814 2.668 3.664 2.380

66POPC H7X 8815 2.682 3.560 2.416

66POPC H7Y 8816 2.603 3.661 2.290

66POPC C38 8817 2.594 3.742 2.490

66POPC H8X 8818 2.492 3.701 2.498

66POPC H8Y 8819 2.585 3.849 2.460

66POPC C39 8820 2.661 3.734 2.629

66POPC H9X 8821 2.769 3.757 2.618

66POPC H9Y 8822 2.653 3.630 2.668

66POPC C310 8823 2.602 3.833 2.730

66POPC H10X 8824 2.605 3.934 2.683

66POPC H10Y 8825 2.665 3.837 2.821

66POPC C311 8826 2.455 3.812 2.766

66POPC H11X 8827 2.394 3.790 2.676

66POPC H11Y 8828 2.417 3.907 2.809

66POPC C312 8829 2.437 3.702 2.870

66POPC H12X 8830 2.504 3.724 2.956

66POPC H12Y 8831 2.467 3.604 2.827

66POPC C313 8832 2.293 3.697 2.920

66POPC H13X 8833 2.226 3.654 2.842

66POPC H13Y 8834 2.261 3.802 2.938

66POPC C314 8835 2.279 3.621 3.052

66POPC H14X 8836 2.178 3.637 3.094

66POPC H14Y 8837 2.352 3.665 3.124

66POPC C315 8838 2.307 3.472 3.042

66POPC H15X 8839 2.393 3.454 2.974

66POPC H15Y 8840 2.219 3.423 2.995

66POPC C316 8841 2.339 3.410 3.178

66POPC H16X 8842 2.449 3.392 3.187

66POPC H16Y 8843 2.286 3.313 3.192

66POPC H16Z 8844 2.308 3.478 3.260

67POPC N 8845 3.495 3.989 0.925

67POPC C12 8846 3.390 3.883 0.892

67POPC H12A 8847 3.420 3.829 0.804

67POPC H12B 8848 3.297 3.932 0.870

67POPC C13 8849 3.537 4.065 0.803

67POPC H13A 8850 3.584 3.999 0.731

67POPC H13B 8851 3.452 4.112 0.756

67POPC H13C 8852 3.607 4.141 0.831

67POPC C14 8853 3.437 4.086 1.023

67POPC H14A 8854 3.398 4.031 1.107

67POPC H14B 8855 3.512 4.155 1.060

67POPC H14C 8856 3.354 4.142 0.982

67POPC C15 8857 3.614 3.928 0.991

67POPC H15A 8858 3.683 4.005 1.024

67POPC H15B 8859 3.666 3.858 0.927

67POPC H15C 8860 3.581 3.874 1.079

67POPC C11 8861 3.360 3.777 1.002

67POPC H11A 8862 3.450 3.714 1.017

67POPC H11B 8863 3.279 3.710 0.963

67POPC P 8864 3.313 3.747 1.254

67POPC O13 8865 3.286 3.607 1.215

67POPC O14 8866 3.227 3.810 1.357

67POPC O12 8867 3.316 3.838 1.123

67POPC O11 8868 3.465 3.757 1.295

67POPC C1 8869 3.523 3.880 1.339

67POPC HA 8870 3.476 3.915 1.434

67POPC HB 8871 3.511 3.960 1.263

67POPC C2 8872 3.673 3.854 1.364

67POPC HS 8873 3.709 3.797 1.276

67POPC O21 8874 3.682 3.777 1.484

67POPC C21 8875 3.776 3.686 1.489

67POPC O22 8876 3.861 3.666 1.404

67POPC C22 8877 3.759 3.603 1.617

67POPC H2R 8878 3.847 3.622 1.681

67POPC H2S 8879 3.758 3.496 1.587

67POPC C3 8880 3.761 3.982 1.375

67POPC HX 8881 3.760 4.036 1.278

67POPC HY 8882 3.866 3.949 1.392

67POPC O31 8883 3.721 4.064 1.487

67POPC C31 8884 3.649 4.172 1.460

67POPC O32 8885 3.612 4.208 1.349

67POPC C32 8886 3.606 4.234 1.592

67POPC H2X 8887 3.577 4.339 1.575

67POPC H2Y 8888 3.695 4.234 1.659

67POPC C23 8889 3.629 3.635 1.695

67POPC H3R 8890 3.542 3.637 1.625

67POPC H3S 8891 3.637 3.736 1.740

67POPC C24 8892 3.596 3.534 1.805

67POPC H4R 8893 3.575 3.434 1.759

67POPC H4S 8894 3.502 3.567 1.853

67POPC C25 8895 3.706 3.519 1.910

67POPC H5R 8896 3.738 3.620 1.944

67POPC H5S 8897 3.794 3.469 1.864

67POPC C26 8898 3.661 3.440 2.033

67POPC H6R 8899 3.751 3.418 2.095

67POPC H6S 8900 3.618 3.343 2.000

67POPC C27 8901 3.558 3.516 2.117

67POPC H7R 8902 3.466 3.532 2.057

67POPC H7S 8903 3.598 3.617 2.144

67POPC C28 8904 3.517 3.441 2.244

67POPC H8R 8905 3.501 3.335 2.213

67POPC H8S 8906 3.420 3.479 2.281

67POPC C29 8907 3.624 3.438 2.349

67POPC H91 8908 3.713 3.378 2.323

67POPC C210 8909 3.618 3.498 2.468

67POPC H101 8910 3.703 3.484 2.536

67POPC C211 8911 3.505 3.580 2.526

67POPC H11R 8912 3.419 3.593 2.457

67POPC H11S 8913 3.542 3.683 2.549

67POPC C212 8914 3.456 3.510 2.654

67POPC H12R 8915 3.541 3.459 2.704

67POPC H12S 8916 3.383 3.434 2.619

67POPC C213 8917 3.382 3.586 2.766

67POPC H13R 8918 3.303 3.518 2.805

67POPC H13S 8919 3.328 3.674 2.725

67POPC C214 8920 3.471 3.623 2.885

67POPC H14R 8921 3.415 3.692 2.951

67POPC H14S 8922 3.558 3.681 2.847

67POPC C215 8923 3.520 3.502 2.967

67POPC H15R 8924 3.580 3.436 2.900

67POPC H15S 8925 3.437 3.440 3.006

67POPC C216 8926 3.613 3.542 3.082

67POPC H16R 8927 3.671 3.626 3.040

67POPC H16S 8928 3.683 3.459 3.106

67POPC C217 8929 3.548 3.591 3.212

67POPC H17R 8930 3.456 3.649 3.189

67POPC H17S 8931 3.616 3.665 3.260

67POPC C218 8932 3.520 3.481 3.314

67POPC H18R 8933 3.614 3.455 3.367

67POPC H18S 8934 3.482 3.390 3.263

67POPC H18T 8935 3.446 3.514 3.389

67POPC C33 8936 3.491 4.152 1.655

67POPC H3X 8937 3.510 4.043 1.644

67POPC H3Y 8938 3.396 4.175 1.602

67POPC C34 8939 3.471 4.177 1.805

67POPC H4X 8940 3.381 4.121 1.838

67POPC H4Y 8941 3.452 4.285 1.824

67POPC C35 8942 3.590 4.130 1.890

67POPC H5X 8943 3.673 4.204 1.884

67POPC H5Y 8944 3.627 4.033 1.850

67POPC C36 8945 3.551 4.109 2.037

67POPC H6X 8946 3.639 4.072 2.094

67POPC H6Y 8947 3.473 4.030 2.043

67POPC C37 8948 3.497 4.235 2.104

67POPC H7X 8949 3.407 4.271 2.049

67POPC H7Y 8950 3.572 4.316 2.101

67POPC C38 8951 3.455 4.210 2.249

67POPC H8X 8952 3.385 4.123 2.254

67POPC H8Y 8953 3.397 4.299 2.283

67POPC C39 8954 3.573 4.188 2.343

67POPC H9X 8955 3.655 4.257 2.314

67POPC H9Y 8956 3.612 4.084 2.334

67POPC C310 8957 3.537 4.220 2.489

67POPC H10X 8958 3.499 4.325 2.486

67POPC H10Y 8959 3.627 4.217 2.554

67POPC C311 8960 3.425 4.135 2.550

67POPC H11X 8961 3.334 4.148 2.487

67POPC H11Y 8962 3.400 4.172 2.652

67POPC C312 8963 3.463 3.987 2.559

67POPC H12X 8964 3.549 3.974 2.627

67POPC H12Y 8965 3.494 3.952 2.458

67POPC C313 8966 3.348 3.896 2.604

67POPC H13X 8967 3.339 3.894 2.715

67POPC H13Y 8968 3.373 3.794 2.569

67POPC C314 8969 3.211 3.929 2.544

67POPC H14X 8970 3.228 3.953 2.437

67POPC H14Y 8971 3.168 4.019 2.594

67POPC C315 8972 3.114 3.812 2.552

67POPC H15X 8973 3.165 3.719 2.517

67POPC H15Y 8974 3.032 3.833 2.479

67POPC C316 8975 3.052 3.788 2.690

67POPC H16X 8976 2.980 3.703 2.685

67POPC H16Y 8977 2.997 3.879 2.724

67POPC H16Z 8978 3.130 3.764 2.765

68POPC N 8979 2.388 2.524 1.055

68POPC C12 8980 2.532 2.529 1.106

68POPC H12A 8981 2.599 2.505 1.024

68POPC H12B 8982 2.547 2.455 1.183

68POPC C13 8983 2.300 2.603 1.148

68POPC H13A 8984 2.198 2.610 1.113

68POPC H13B 8985 2.301 2.564 1.248

68POPC H13C 8986 2.341 2.703 1.155

68POPC C14 8987 2.384 2.591 0.921

68POPC H14A 8988 2.423 2.691 0.930

68POPC H14B 8989 2.448 2.542 0.848

68POPC H14C 8990 2.284 2.597 0.880

68POPC C15 8991 2.336 2.385 1.038

68POPC H15A 8992 2.239 2.386 0.990

68POPC H15B 8993 2.321 2.335 1.132

68POPC H15C 8994 2.403 2.325 0.979

68POPC C11 8995 2.578 2.666 1.162

68POPC H11A 8996 2.689 2.672 1.170

68POPC H11B 8997 2.537 2.676 1.265

68POPC P 8998 2.593 2.874 0.995

68POPC O13 8999 2.520 2.866 0.866

68POPC O14 9000 2.738 2.848 0.993

68POPC O12 9001 2.522 2.771 1.087

68POPC O11 9002 2.553 3.005 1.071

68POPC C1 9003 2.424 3.018 1.133

68POPC HA 9004 2.353 2.938 1.103

68POPC HB 9005 2.380 3.115 1.101

68POPC C2 9006 2.426 3.023 1.289

68POPC HS 9007 2.462 3.125 1.315

68POPC O21 9008 2.508 2.921 1.348

68POPC C21 9009 2.632 2.961 1.372

68POPC O22 9010 2.685 3.062 1.329

68POPC C22 9011 2.693 2.875 1.482

68POPC H2R 9012 2.707 2.947 1.565

68POPC H2S 9013 2.794 2.842 1.447

68POPC C3 9014 2.281 3.012 1.348

68POPC HX 9015 2.209 3.038 1.268

68POPC HY 9016 2.270 3.090 1.425

68POPC O31 9017 2.249 2.879 1.398

68POPC C31 9018 2.275 2.864 1.528

68POPC O32 9019 2.322 2.948 1.601

68POPC C32 9020 2.239 2.723 1.574

68POPC H2X 9021 2.315 2.655 1.530

68POPC H2Y 9022 2.140 2.699 1.529

68POPC C23 9023 2.614 2.751 1.530

68POPC H3R 9024 2.607 2.677 1.447

68POPC H3S 9025 2.513 2.778 1.564

68POPC C24 9026 2.686 2.681 1.645

68POPC H4R 9027 2.787 2.658 1.604

68POPC H4S 9028 2.638 2.584 1.671

68POPC C25 9029 2.702 2.769 1.770

68POPC H5R 9030 2.718 2.876 1.746

68POPC H5S 9031 2.796 2.738 1.820

68POPC C26 9032 2.588 2.761 1.871

68POPC H6R 9033 2.572 2.655 1.898

68POPC H6S 9034 2.495 2.802 1.825

68POPC C27 9035 2.624 2.839 1.997

68POPC H7R 9036 2.640 2.947 1.974

68POPC H7S 9037 2.723 2.797 2.027

68POPC C28 9038 2.530 2.820 2.116

68POPC H8R 9039 2.581 2.859 2.207

68POPC H8S 9040 2.514 2.711 2.132

68POPC C29 9041 2.401 2.892 2.090

68POPC H91 9042 2.370 2.895 1.985

68POPC C210 9043 2.325 2.954 2.181

68POPC H101 9044 2.234 3.007 2.148

68POPC C211 9045 2.350 2.959 2.329

68POPC H11R 9046 2.404 3.052 2.356

68POPC H11S 9047 2.412 2.873 2.362

68POPC C212 9048 2.216 2.955 2.406

68POPC H12R 9049 2.149 2.879 2.362

68POPC H12S 9050 2.167 3.054 2.395

68POPC C213 9051 2.233 2.921 2.555

68POPC H13R 9052 2.268 2.816 2.561

68POPC H13S 9053 2.136 2.931 2.608

68POPC C214 9054 2.341 3.005 2.623

68POPC H14R 9055 2.316 3.110 2.596

68POPC H14S 9056 2.439 2.981 2.578

68POPC C215 9057 2.354 2.993 2.776

68POPC H15R 9058 2.453 3.035 2.801

68POPC H15S 9059 2.350 2.888 2.811

68POPC C216 9060 2.252 3.075 2.852

68POPC H16R 9061 2.161 3.015 2.873

68POPC H16S 9062 2.224 3.158 2.783

68POPC C217 9063 2.305 3.144 2.978

68POPC H17R 9064 2.219 3.196 3.026

68POPC H17S 9065 2.377 3.222 2.946

68POPC C218 9066 2.375 3.055 3.079

68POPC H18R 9067 2.408 3.112 3.168

68POPC H18S 9068 2.464 3.005 3.034

68POPC H18T 9069 2.305 2.975 3.113

68POPC C33 9070 2.232 2.701 1.728

68POPC H3X 9071 2.126 2.679 1.754

68POPC H3Y 9072 2.261 2.793 1.783

68POPC C34 9073 2.319 2.585 1.780

68POPC H4X 9074 2.424 2.600 1.746

68POPC H4Y 9075 2.287 2.488 1.737

68POPC C35 9076 2.316 2.565 1.933

68POPC H5X 9077 2.362 2.653 1.981

68POPC H5Y 9078 2.378 2.476 1.959

68POPC C36 9079 2.174 2.548 1.989

68POPC H6X 9080 2.119 2.476 1.925

68POPC H6Y 9081 2.123 2.646 1.983

68POPC C37 9082 2.169 2.499 2.135

68POPC H7X 9083 2.189 2.390 2.138

68POPC H7Y 9084 2.065 2.514 2.172

68POPC C38 9085 2.266 2.570 2.231

68POPC H8X 9086 2.268 2.679 2.210

68POPC H8Y 9087 2.369 2.530 2.214

68POPC C39 9088 2.226 2.550 2.378

68POPC H9X 9089 2.187 2.447 2.392

68POPC H9Y 9090 2.144 2.621 2.401

68POPC C310 9091 2.341 2.572 2.477

68POPC H10X 9092 2.391 2.669 2.457

68POPC H10Y 9093 2.417 2.492 2.463

68POPC C311 9094 2.290 2.569 2.621

68POPC H11X 9095 2.237 2.472 2.637

68POPC H11Y 9096 2.212 2.647 2.638

68POPC C312 9097 2.399 2.579 2.728

68POPC H12X 9098 2.457 2.485 2.718

68POPC H12Y 9099 2.349 2.584 2.827

68POPC C313 9100 2.508 2.687 2.720

68POPC H13X 9101 2.467 2.784 2.684

68POPC H13Y 9102 2.581 2.654 2.643

68POPC C314 9103 2.579 2.700 2.856

68POPC H14X 9104 2.650 2.616 2.869

68POPC H14Y 9105 2.499 2.688 2.932

68POPC C315 9106 2.645 2.835 2.892

68POPC H15X 9107 2.604 2.866 2.990

68POPC H15Y 9108 2.612 2.912 2.819

68POPC C316 9109 2.798 2.842 2.906

68POPC H16X 9110 2.849 2.802 2.816

68POPC H16Y 9111 2.831 2.784 2.995

68POPC H16Z 9112 2.830 2.947 2.920

69POPC N 9113 4.324 3.497 1.013

69POPC C12 9114 4.246 3.374 0.967

69POPC H12A 9115 4.264 3.292 1.035

69POPC H12B 9116 4.284 3.343 0.871

69POPC C13 9117 4.295 3.614 0.923

69POPC H13A 9118 4.354 3.700 0.950

69POPC H13B 9119 4.316 3.589 0.820

69POPC H13C 9120 4.190 3.640 0.931

69POPC C14 9121 4.287 3.535 1.153

69POPC H14A 9122 4.180 3.547 1.160

69POPC H14B 9123 4.315 3.457 1.222

69POPC H14C 9124 4.334 3.627 1.184

69POPC C15 9125 4.471 3.468 1.005

69POPC H15A 9126 4.499 3.441 0.904

69POPC H15B 9127 4.498 3.386 1.070

69POPC H15C 9128 4.529 3.554 1.031

69POPC C11 9129 4.092 3.386 0.956

69POPC H11A 9130 4.055 3.305 0.890

69POPC H11B 9131 4.064 3.481 0.906

69POPC P 9132 4.010 3.238 1.151

69POPC O13 9133 4.137 3.162 1.136

69POPC O14 9134 3.887 3.179 1.095

69POPC O12 9135 4.032 3.381 1.085

69POPC O11 9136 3.991 3.279 1.301

69POPC C1 9137 4.103 3.317 1.382

69POPC HA 9138 4.073 3.395 1.454

69POPC HB 9139 4.187 3.358 1.322

69POPC C2 9140 4.158 3.197 1.463

69POPC HS 9141 4.164 3.111 1.392

69POPC O21 9142 4.059 3.165 1.562

69POPC C21 9143 4.088 3.105 1.675

69POPC O22 9144 4.196 3.067 1.713

69POPC C22 9145 3.957 3.083 1.751

69POPC H2R 9146 3.955 2.977 1.784

69POPC H2S 9147 3.873 3.098 1.680

69POPC C3 9148 4.304 3.221 1.512

69POPC HX 9149 4.364 3.260 1.427

69POPC HY 9150 4.346 3.121 1.539

69POPC O31 9151 4.311 3.315 1.621

69POPC C31 9152 4.417 3.295 1.699

69POPC O32 9153 4.512 3.223 1.672

69POPC C32 9154 4.404 3.381 1.823

69POPC H2X 9155 4.318 3.343 1.882

69POPC H2Y 9156 4.381 3.485 1.790

69POPC C23 9157 3.939 3.178 1.871

69POPC H3R 9158 3.830 3.188 1.889

69POPC H3S 9159 3.976 3.281 1.849

69POPC C24 9160 4.001 3.124 2.000

69POPC H4R 9161 4.000 3.013 2.000

69POPC H4S 9162 3.940 3.157 2.087

69POPC C25 9163 4.144 3.175 2.023

69POPC H5R 9164 4.140 3.284 2.042

69POPC H5S 9165 4.207 3.157 1.934

69POPC C26 9166 4.209 3.104 2.141

69POPC H6R 9167 4.241 3.002 2.111

69POPC H6S 9168 4.136 3.094 2.224

69POPC C27 9169 4.331 3.181 2.192

69POPC H7R 9170 4.297 3.283 2.217

69POPC H7S 9171 4.408 3.188 2.113

69POPC C28 9172 4.388 3.112 2.316

69POPC H8R 9173 4.445 3.022 2.285

69POPC H8S 9174 4.302 3.076 2.376

69POPC C29 9175 4.474 3.208 2.392

69POPC H91 9176 4.535 3.277 2.332

69POPC C210 9177 4.477 3.215 2.526

69POPC H101 9178 4.540 3.291 2.573

69POPC C211 9179 4.401 3.125 2.619

69POPC H11R 9180 4.457 3.030 2.635

69POPC H11S 9181 4.301 3.097 2.579

69POPC C212 9182 4.380 3.190 2.756

69POPC H12R 9183 4.312 3.128 2.818

69POPC H12S 9184 4.332 3.290 2.743

69POPC C213 9185 4.512 3.205 2.830

69POPC H13R 9186 4.593 3.200 2.754

69POPC H13S 9187 4.527 3.118 2.897

69POPC C214 9188 4.525 3.334 2.911

69POPC H14R 9189 4.617 3.328 2.974

69POPC H14S 9190 4.438 3.333 2.980

69POPC C215 9191 4.523 3.470 2.840

69POPC H15R 9192 4.444 3.473 2.762

69POPC H15S 9193 4.620 3.489 2.789

69POPC C216 9194 4.499 3.585 2.939

69POPC H16R 9195 4.391 3.582 2.965

69POPC H16S 9196 4.518 3.683 2.890

69POPC C217 9197 4.578 3.575 3.071

69POPC H17R 9198 4.685 3.598 3.051

69POPC H17S 9199 4.573 3.472 3.111

69POPC C218 9200 4.524 3.662 3.184

69POPC H18R 9201 4.533 3.770 3.162

69POPC H18S 9202 4.576 3.641 3.280

69POPC H18T 9203 4.416 3.640 3.198

69POPC C33 9204 4.531 3.380 1.910

69POPC H3X 9205 4.616 3.420 1.851

69POPC H3Y 9206 4.557 3.275 1.939

69POPC C34 9207 4.515 3.464 2.037

69POPC H4X 9208 4.611 3.465 2.093

69POPC H4Y 9209 4.440 3.417 2.104

69POPC C35 9210 4.471 3.609 2.008

69POPC H5X 9211 4.363 3.610 1.982

69POPC H5Y 9212 4.527 3.645 1.919

69POPC C36 9213 4.497 3.706 2.124

69POPC H6X 9214 4.475 3.810 2.092

69POPC H6Y 9215 4.606 3.700 2.146

69POPC C37 9216 4.416 3.674 2.250

69POPC H7X 9217 4.468 3.713 2.340

69POPC H7Y 9218 4.409 3.564 2.263

69POPC C38 9219 4.276 3.736 2.247

69POPC H8X 9220 4.234 3.730 2.144

69POPC H8Y 9221 4.284 3.843 2.274

69POPC C39 9222 4.178 3.665 2.342

69POPC H9X 9223 4.094 3.733 2.366

69POPC H9Y 9224 4.230 3.641 2.437

69POPC C310 9225 4.120 3.538 2.277

69POPC H10X 9226 4.204 3.472 2.245

69POPC H10Y 9227 4.063 3.567 2.187

69POPC C311 9228 4.028 3.458 2.369

69POPC H11X 9229 3.982 3.376 2.310

69POPC H11Y 9230 3.946 3.523 2.406

69POPC C312 9231 4.101 3.396 2.489

69POPC H12X 9232 4.129 3.477 2.559

69POPC H12Y 9233 4.194 3.345 2.457

69POPC C313 9234 4.010 3.296 2.560

69POPC H13X 9235 4.004 3.201 2.503

69POPC H13Y 9236 3.908 3.340 2.564

69POPC C314 9237 4.057 3.269 2.702

69POPC H14X 9238 4.099 3.362 2.746

69POPC H14Y 9239 4.137 3.192 2.700

69POPC C315 9240 3.944 3.220 2.793

69POPC H15X 9241 3.846 3.255 2.755

69POPC H15Y 9242 3.958 3.270 2.891

69POPC C316 9243 3.946 3.069 2.808

69POPC H16X 9244 4.043 3.034 2.850

69POPC H16Y 9245 3.932 3.021 2.709

69POPC H16Z 9246 3.865 3.036 2.877

70POPC N 9247 3.309 2.266 0.923

70POPC C12 9248 3.395 2.142 0.943

70POPC H12A 9249 3.474 2.168 1.013

70POPC H12B 9250 3.444 2.115 0.850

70POPC C13 9251 3.239 2.302 1.051

70POPC H13A 9252 3.181 2.219 1.088

70POPC H13B 9253 3.310 2.324 1.130

70POPC H13C 9254 3.175 2.388 1.042

70POPC C14 9255 3.398 2.380 0.886

70POPC H14A 9256 3.341 2.471 0.870

70POPC H14B 9257 3.466 2.401 0.967

70POPC H14C 9258 3.457 2.360 0.797

70POPC C15 9259 3.205 2.244 0.817

70POPC H15A 9260 3.251 2.216 0.724

70POPC H15B 9261 3.148 2.334 0.803

70POPC H15C 9262 3.137 2.166 0.849

70POPC C11 9263 3.330 2.014 1.002

70POPC H11A 9264 3.399 1.928 0.992

70POPC H11B 9265 3.238 1.989 0.944

70POPC P 9266 3.409 2.050 1.248

70POPC O13 9267 3.509 2.152 1.206

70POPC O14 9268 3.461 1.916 1.282

70POPC O12 9269 3.295 2.034 1.138

70POPC O11 9270 3.330 2.110 1.368

70POPC C1 9271 3.197 2.064 1.392

70POPC HA 9272 3.199 1.956 1.419

70POPC HB 9273 3.131 2.080 1.304

70POPC C2 9274 3.134 2.138 1.510

70POPC HS 9275 3.122 2.245 1.484

70POPC O21 9276 3.213 2.113 1.627

70POPC C21 9277 3.270 2.218 1.680

70POPC O22 9278 3.257 2.334 1.645

70POPC C22 9279 3.348 2.171 1.802

70POPC H2R 9280 3.403 2.079 1.774

70POPC H2S 9281 3.275 2.146 1.882

70POPC C3 9282 2.994 2.081 1.537

70POPC HX 9283 3.012 1.972 1.548

70POPC HY 9284 2.928 2.097 1.449

70POPC O31 9285 2.941 2.147 1.653

70POPC C31 9286 2.896 2.062 1.742

70POPC O32 9287 2.877 1.943 1.722

70POPC C32 9288 2.868 2.133 1.873

70POPC H2X 9289 2.806 2.066 1.936

70POPC H2Y 9290 2.809 2.225 1.852

70POPC C23 9291 3.448 2.277 1.851

70POPC H3R 9292 3.393 2.365 1.893

70POPC H3S 9293 3.509 2.313 1.766

70POPC C24 9294 3.542 2.219 1.957

70POPC H4R 9295 3.615 2.297 1.987

70POPC H4S 9296 3.600 2.137 1.910

70POPC C25 9297 3.470 2.167 2.082

70POPC H5R 9298 3.360 2.185 2.076

70POPC H5S 9299 3.505 2.228 2.168

70POPC C26 9300 3.499 2.019 2.114

70POPC H6R 9301 3.523 2.020 2.223

70POPC H6S 9302 3.592 1.986 2.064

70POPC C27 9303 3.386 1.915 2.088

70POPC H7R 9304 3.361 1.922 1.980

70POPC H7S 9305 3.294 1.945 2.142

70POPC C28 9306 3.428 1.767 2.123

70POPC H8R 9307 3.523 1.772 2.181

70POPC H8S 9308 3.469 1.732 2.026

70POPC C29 9309 3.334 1.656 2.171

70POPC H91 9310 3.309 1.594 2.083

70POPC C210 9311 3.278 1.606 2.286

70POPC H101 9312 3.210 1.521 2.269

70POPC C211 9313 3.278 1.635 2.435

70POPC H11R 9314 3.365 1.696 2.469

70POPC H11S 9315 3.289 1.539 2.490

70POPC C212 9316 3.152 1.703 2.493

70POPC H12R 9317 3.077 1.623 2.510

70POPC H12S 9318 3.108 1.772 2.418

70POPC C213 9319 3.168 1.784 2.624

70POPC H13R 9320 3.246 1.862 2.615

70POPC H13S 9321 3.197 1.716 2.708

70POPC C214 9322 3.035 1.855 2.658

70POPC H14R 9323 2.957 1.776 2.655

70POPC H14S 9324 3.010 1.926 2.577

70POPC C215 9325 3.023 1.925 2.794

70POPC H15R 9326 3.097 1.885 2.867

70POPC H15S 9327 2.921 1.906 2.834

70POPC C216 9328 3.033 2.075 2.779

70POPC H16R 9329 2.974 2.106 2.690

70POPC H16S 9330 3.138 2.100 2.754

70POPC C217 9331 2.987 2.158 2.899

70POPC H17R 9332 2.932 2.096 2.973

70POPC H17S 9333 2.916 2.237 2.865

70POPC C218 9334 3.106 2.227 2.961

70POPC H18R 9335 3.190 2.155 2.968

70POPC H18S 9336 3.085 2.267 3.063

70POPC H18T 9337 3.137 2.312 2.896

70POPC C33 9338 3.000 2.167 1.942

70POPC H3X 9339 3.056 2.240 1.879

70POPC H3Y 9340 3.063 2.076 1.953

70POPC C34 9341 2.979 2.229 2.080

70POPC H4X 9342 2.929 2.156 2.147

70POPC H4Y 9343 2.912 2.316 2.069

70POPC C35 9344 3.112 2.274 2.143

70POPC H5X 9345 3.170 2.334 2.070

70POPC H5Y 9346 3.171 2.185 2.173

70POPC C36 9347 3.080 2.359 2.266

70POPC H6X 9348 3.020 2.294 2.333

70POPC H6Y 9349 3.016 2.443 2.233

70POPC C37 9350 3.196 2.422 2.345

70POPC H7X 9351 3.150 2.503 2.405

70POPC H7Y 9352 3.271 2.467 2.277

70POPC C38 9353 3.262 2.325 2.445

70POPC H8X 9354 3.348 2.280 2.390

70POPC H8Y 9355 3.190 2.244 2.468

70POPC C39 9356 3.309 2.385 2.580

70POPC H9X 9357 3.230 2.450 2.624

70POPC H9Y 9358 3.398 2.447 2.555

70POPC C310 9359 3.356 2.280 2.682

70POPC H10X 9360 3.451 2.248 2.634

70POPC H10Y 9361 3.287 2.193 2.683

70POPC C311 9362 3.409 2.312 2.821

70POPC H11X 9363 3.340 2.278 2.902

70POPC H11Y 9364 3.419 2.422 2.835

70POPC C312 9365 3.544 2.243 2.848

70POPC H12X 9366 3.586 2.288 2.939

70POPC H12Y 9367 3.614 2.261 2.764

70POPC C313 9368 3.536 2.091 2.872

70POPC H13X 9369 3.640 2.053 2.868

70POPC H13Y 9370 3.479 2.040 2.791

70POPC C314 9371 3.475 2.060 3.009

70POPC H14X 9372 3.367 2.038 2.996

70POPC H14Y 9373 3.483 2.151 3.072

70POPC C315 9374 3.543 1.945 3.085

70POPC H15X 9375 3.623 1.900 3.022

70POPC H15Y 9376 3.468 1.866 3.103

70POPC C316 9377 3.606 1.989 3.217

70POPC H16X 9378 3.687 2.063 3.199

70POPC H16Y 9379 3.650 1.901 3.269

70POPC H16Z 9380 3.529 2.033 3.284

71POPC N 9381 3.958 2.689 1.065

71POPC C12 9382 4.049 2.652 1.181

71POPC H12A 9383 3.987 2.616 1.261

71POPC H12B 9384 4.102 2.739 1.216

71POPC C13 9385 3.859 2.577 1.053

71POPC H13A 9386 3.913 2.484 1.044

71POPC H13B 9387 3.799 2.567 1.142

71POPC H13C 9388 3.793 2.587 0.968

71POPC C14 9389 3.886 2.817 1.092

71POPC H14A 9390 3.816 2.839 1.012

71POPC H14B 9391 3.829 2.811 1.183

71POPC H14C 9392 3.954 2.901 1.100

71POPC C15 9393 4.040 2.695 0.940

71POPC H15A 9394 4.092 2.602 0.929

71POPC H15B 9395 4.115 2.773 0.946

71POPC H15C 9396 3.978 2.710 0.852

71POPC C11 9397 4.155 2.542 1.154

71POPC H11A 9398 4.201 2.519 1.253

71POPC H11B 9399 4.236 2.583 1.090

71POPC P 9400 4.179 2.291 1.107

71POPC O13 9401 4.318 2.320 1.067

71POPC O14 9402 4.110 2.184 1.030

71POPC O12 9403 4.098 2.426 1.092

71POPC O11 9404 4.164 2.260 1.262

71POPC C1 9405 4.031 2.226 1.307

71POPC HA 9406 4.007 2.121 1.277

71POPC HB 9407 3.953 2.290 1.261

71POPC C2 9408 4.011 2.232 1.463

71POPC HS 9409 3.907 2.200 1.485

71POPC O21 9410 4.033 2.361 1.520

71POPC C21 9411 3.979 2.468 1.472

71POPC O22 9412 3.891 2.478 1.388

71POPC C22 9413 4.051 2.588 1.534

71POPC H2R 9414 4.003 2.612 1.632

71POPC H2S 9415 4.038 2.674 1.466

71POPC C3 9416 4.106 2.133 1.537

71POPC HX 9417 4.211 2.167 1.522

71POPC HY 9418 4.096 2.034 1.489

71POPC O31 9419 4.070 2.121 1.677

71POPC C31 9420 4.133 2.204 1.759

71POPC O32 9421 4.223 2.280 1.731

71POPC C32 9422 4.077 2.187 1.901

71POPC H2X 9423 4.143 2.113 1.951

71POPC H2Y 9424 3.974 2.145 1.893

71POPC C23 9425 4.202 2.557 1.554

71POPC H3R 9426 4.241 2.497 1.470

71POPC H3S 9427 4.216 2.497 1.646

71POPC C24 9428 4.287 2.683 1.563

71POPC H4R 9429 4.272 2.738 1.467

71POPC H4S 9430 4.394 2.653 1.570

71POPC C25 9431 4.253 2.775 1.680

71POPC H5R 9432 4.153 2.821 1.664

71POPC H5S 9433 4.328 2.857 1.683

71POPC C26 9434 4.254 2.702 1.814

71POPC H6R 9435 4.351 2.648 1.823

71POPC H6S 9436 4.173 2.627 1.817

71POPC C27 9437 4.235 2.798 1.933

71POPC H7R 9438 4.144 2.859 1.915

71POPC H7S 9439 4.320 2.869 1.937

71POPC C28 9440 4.218 2.723 2.067

71POPC H8R 9441 4.119 2.672 2.064

71POPC H8S 9442 4.213 2.797 2.149

71POPC C29 9443 4.326 2.622 2.093

71POPC H91 9444 4.407 2.615 2.018

71POPC C210 9445 4.328 2.539 2.198

71POPC H101 9446 4.410 2.466 2.208

71POPC C211 9447 4.228 2.541 2.310

71POPC H11R 9448 4.165 2.450 2.310

71POPC H11S 9449 4.158 2.628 2.303

71POPC C212 9450 4.300 2.551 2.445

71POPC H12R 9451 4.364 2.462 2.463

71POPC H12S 9452 4.222 2.550 2.524

71POPC C213 9453 4.384 2.679 2.459

71POPC H13R 9454 4.326 2.766 2.423

71POPC H13S 9455 4.475 2.671 2.395

71POPC C214 9456 4.425 2.703 2.604

71POPC H14R 9457 4.510 2.773 2.610

71POPC H14S 9458 4.457 2.607 2.649

71POPC C215 9459 4.309 2.763 2.684

71POPC H15R 9460 4.219 2.700 2.667

71POPC H15S 9461 4.285 2.865 2.648

71POPC C216 9462 4.337 2.765 2.834

71POPC H16R 9463 4.392 2.858 2.860

71POPC H16S 9464 4.401 2.679 2.861

71POPC C217 9465 4.206 2.754 2.911

71POPC H17R 9466 4.124 2.803 2.855

71POPC H17S 9467 4.216 2.810 3.007

71POPC C218 9468 4.169 2.608 2.941

71POPC H18R 9469 4.137 2.557 2.848

71POPC H18S 9470 4.087 2.604 3.015

71POPC H18T 9471 4.256 2.553 2.982

71POPC C33 9472 4.073 2.319 1.981

71POPC H3X 9473 4.021 2.397 1.921

71POPC H3Y 9474 4.176 2.356 2.001

71POPC C34 9475 3.999 2.304 2.115

71POPC H4X 9476 3.896 2.268 2.093

71POPC H4Y 9477 3.989 2.403 2.164

71POPC C35 9478 4.066 2.205 2.211

71POPC H5X 9479 4.160 2.248 2.253

71POPC H5Y 9480 4.095 2.114 2.155

71POPC C36 9481 3.973 2.161 2.324

71POPC H6X 9482 3.877 2.130 2.277

71POPC H6Y 9483 3.951 2.245 2.394

71POPC C37 9484 4.029 2.041 2.401

71POPC H7X 9485 4.098 1.989 2.329

71POPC H7Y 9486 3.954 1.962 2.422

71POPC C38 9487 4.101 2.077 2.532

71POPC H8X 9488 4.179 2.154 2.510

71POPC H8Y 9489 4.153 1.985 2.566

71POPC C39 9490 4.019 2.124 2.652

71POPC H9X 9491 4.028 2.046 2.731

71POPC H9Y 9492 3.911 2.137 2.630

71POPC C310 9493 4.073 2.256 2.709

71POPC H10X 9494 4.045 2.341 2.643

71POPC H10Y 9495 4.184 2.251 2.710

71POPC C311 9496 4.025 2.281 2.852

71POPC H11X 9497 4.104 2.336 2.907

71POPC H11Y 9498 4.011 2.183 2.904

71POPC C312 9499 3.895 2.362 2.857

71POPC H12X 9500 3.825 2.320 2.782

71POPC H12Y 9501 3.917 2.467 2.828

71POPC C313 9502 3.827 2.360 2.995

71POPC H13X 9503 3.802 2.254 3.020

71POPC H13Y 9504 3.733 2.418 2.986

71POPC C314 9505 3.909 2.421 3.109

71POPC H14X 9506 3.910 2.531 3.094

71POPC H14Y 9507 4.014 2.385 3.104

71POPC C315 9508 3.852 2.389 3.247

71POPC H15X 9509 3.906 2.452 3.321

71POPC H15Y 9510 3.872 2.283 3.272

71POPC C316 9511 3.701 2.416 3.257

71POPC H16X 9512 3.643 2.332 3.214

71POPC H16Y 9513 3.676 2.507 3.200

71POPC H16Z 9514 3.671 2.431 3.363

72POPC N 9515 2.367 4.533 1.027

72POPC C12 9516 2.479 4.625 0.978

72POPC H12A 9517 2.442 4.725 0.968

72POPC H12B 9518 2.508 4.590 0.880

72POPC C13 9519 2.409 4.390 1.009

72POPC H13A 9520 2.417 4.363 0.904

72POPC H13B 9521 2.506 4.377 1.053

72POPC H13C 9522 2.342 4.323 1.061

72POPC C14 9523 2.344 4.554 1.173

72POPC H14A 9524 2.276 4.480 1.212

72POPC H14B 9525 2.438 4.538 1.224

72POPC H14C 9526 2.311 4.654 1.196

72POPC C15 9527 2.242 4.555 0.948

72POPC H15A 9528 2.263 4.537 0.844

72POPC H15B 9529 2.207 4.656 0.961

72POPC H15C 9530 2.166 4.486 0.982

72POPC C11 9531 2.607 4.631 1.066

72POPC H11A 9532 2.586 4.686 1.160

72POPC H11B 9533 2.685 4.686 1.011

72POPC P 9534 2.791 4.456 1.138

72POPC O13 9535 2.887 4.565 1.110

72POPC O14 9536 2.815 4.325 1.073

72POPC O12 9537 2.648 4.499 1.096

72POPC O11 9538 2.772 4.436 1.293

72POPC C1 9539 2.707 4.318 1.342

72POPC HA 9540 2.766 4.227 1.315

72POPC HB 9541 2.606 4.306 1.299

72POPC C2 9542 2.700 4.322 1.497

72POPC HS 9543 2.662 4.225 1.535

72POPC O21 9544 2.617 4.430 1.542

72POPC C21 9545 2.489 4.419 1.518

72POPC O22 9546 2.431 4.321 1.477

72POPC C22 9547 2.424 4.554 1.548

72POPC H2R 9548 2.457 4.626 1.469

72POPC H2S 9549 2.465 4.590 1.645

72POPC C3 9550 2.843 4.343 1.553

72POPC HX 9551 2.883 4.440 1.516

72POPC HY 9552 2.908 4.263 1.511

72POPC O31 9553 2.845 4.333 1.696

72POPC C31 9554 2.833 4.445 1.764

72POPC O32 9555 2.822 4.558 1.719

72POPC C32 9556 2.838 4.410 1.911

72POPC H2X 9557 2.923 4.339 1.924

72POPC H2Y 9558 2.744 4.357 1.939

72POPC C23 9559 2.270 4.547 1.553

72POPC H3R 9560 2.235 4.469 1.623

72POPC H3S 9561 2.232 4.520 1.452

72POPC C24 9562 2.207 4.682 1.594

72POPC H4R 9563 2.110 4.694 1.541

72POPC H4S 9564 2.273 4.765 1.560

72POPC C25 9565 2.180 4.695 1.744

72POPC H5R 9566 2.099 4.626 1.774

72POPC H5S 9567 2.145 4.799 1.763

72POPC C26 9568 2.302 4.671 1.835

72POPC H6R 9569 2.384 4.738 1.803

72POPC H6S 9570 2.336 4.565 1.824

72POPC C27 9571 2.270 4.696 1.983

72POPC H7R 9572 2.183 4.633 2.012

72POPC H7S 9573 2.241 4.802 1.997

72POPC C28 9574 2.386 4.662 2.078

72POPC H8R 9575 2.411 4.554 2.068

72POPC H8S 9576 2.351 4.676 2.182

72POPC C29 9577 2.508 4.746 2.049

72POPC H91 9578 2.566 4.715 1.961

72POPC C210 9579 2.549 4.854 2.118

72POPC H101 9580 2.643 4.902 2.088

72POPC C211 9581 2.483 4.913 2.241

72POPC H11R 9582 2.379 4.876 2.255

72POPC H11S 9583 2.476 5.023 2.231

72POPC C212 9584 2.563 4.880 2.367

72POPC H12R 9585 2.515 4.929 2.455

72POPC H12S 9586 2.666 4.922 2.358

72POPC C213 9587 2.574 4.729 2.394

72POPC H13R 9588 2.610 4.676 2.303

72POPC H13S 9589 2.473 4.688 2.417

72POPC C214 9590 2.674 4.697 2.506

72POPC H14R 9591 2.773 4.741 2.481

72POPC H14S 9592 2.687 4.587 2.508

72POPC C215 9593 2.629 4.746 2.644

72POPC H15R 9594 2.626 4.857 2.648

72POPC H15S 9595 2.705 4.713 2.718

72POPC C216 9596 2.492 4.689 2.684

72POPC H16R 9597 2.474 4.595 2.627

72POPC H16S 9598 2.412 4.760 2.655

72POPC C217 9599 2.482 4.654 2.833

72POPC H17R 9600 2.493 4.748 2.891

72POPC H17S 9601 2.569 4.591 2.863

72POPC C218 9602 2.353 4.579 2.866

72POPC H18R 9603 2.351 4.480 2.815

72POPC H18S 9604 2.263 4.634 2.832

72POPC H18T 9605 2.345 4.562 2.976

72POPC C33 9606 2.861 4.531 2.004

72POPC H3X 9607 2.764 4.580 2.027

72POPC H3Y 9608 2.925 4.607 1.954

72POPC C34 9609 2.929 4.486 2.133

72POPC H4X 9610 2.942 4.572 2.202

72POPC H4Y 9611 3.030 4.445 2.108

72POPC C35 9612 2.855 4.373 2.201

72POPC H5X 9613 2.852 4.284 2.135

72POPC H5Y 9614 2.750 4.404 2.222

72POPC C36 9615 2.923 4.330 2.330

72POPC H6X 9616 2.910 4.410 2.407

72POPC H6Y 9617 3.031 4.313 2.315

72POPC C37 9618 2.857 4.200 2.375

72POPC H7X 9619 2.891 4.117 2.309

72POPC H7Y 9620 2.748 4.211 2.354

72POPC C38 9621 2.880 4.170 2.522

72POPC H8X 9622 2.893 4.266 2.576

72POPC H8Y 9623 2.973 4.112 2.536

72POPC C39 9624 2.761 4.096 2.584

72POPC H9X 9625 2.781 4.078 2.692

72POPC H9Y 9626 2.753 3.996 2.536

72POPC C310 9627 2.625 4.167 2.570

72POPC H10X 9628 2.554 4.133 2.648

72POPC H10Y 9629 2.581 4.137 2.473

72POPC C311 9630 2.630 4.321 2.572

72POPC H11X 9631 2.532 4.356 2.534

72POPC H11Y 9632 2.706 4.362 2.502

72POPC C312 9633 2.648 4.386 2.709

72POPC H12X 9634 2.546 4.374 2.752

72POPC H12Y 9635 2.663 4.495 2.692

72POPC C313 9636 2.758 4.344 2.809

72POPC H13X 9637 2.824 4.264 2.771

72POPC H13Y 9638 2.709 4.303 2.901

72POPC C314 9639 2.847 4.461 2.856

72POPC H14X 9640 2.786 4.554 2.860

72POPC H14Y 9641 2.927 4.476 2.780

72POPC C315 9642 2.909 4.442 2.995

72POPC H15X 9643 2.829 4.454 3.072

72POPC H15Y 9644 2.983 4.523 3.012

72POPC C316 9645 2.978 4.307 3.018

72POPC H16X 9646 2.903 4.226 3.027

72POPC H16Y 9647 3.038 4.309 3.111

72POPC H16Z 9648 3.046 4.283 2.933

73POPC N 9649 1.589 3.118 1.098

73POPC C12 9650 1.580 3.259 1.157

73POPC H12A 9651 1.639 3.261 1.248

73POPC H12B 9652 1.477 3.278 1.182

73POPC C13 9653 1.531 3.119 0.961

73POPC H13A 9654 1.586 3.188 0.898

73POPC H13B 9655 1.535 3.021 0.914

73POPC H13C 9656 1.428 3.150 0.964

73POPC C14 9657 1.732 3.075 1.094

73POPC H14A 9658 1.791 3.149 1.043

73POPC H14B 9659 1.774 3.070 1.194

73POPC H14C 9660 1.746 2.979 1.045

73POPC C15 9661 1.510 3.024 1.184

73POPC H15A 9662 1.407 3.056 1.190

73POPC H15B 9663 1.552 3.021 1.284

73POPC H15C 9664 1.510 2.925 1.143

73POPC C11 9665 1.630 3.379 1.070

73POPC H11A 9666 1.619 3.472 1.129

73POPC H11B 9667 1.562 3.389 0.983

73POPC P 9668 1.890 3.390 1.112

73POPC O13 9669 1.961 3.508 1.058

73POPC O14 9670 1.967 3.264 1.122

73POPC O12 9671 1.763 3.361 1.023

73POPC O11 9672 1.832 3.423 1.256

73POPC C1 9673 1.926 3.429 1.366

73POPC HA 9674 1.892 3.507 1.437

73POPC HB 9675 2.028 3.456 1.334

73POPC C2 9676 1.932 3.296 1.444

73POPC HS 9677 1.945 3.214 1.371

73POPC O21 9678 1.809 3.288 1.519

73POPC C21 9679 1.721 3.201 1.480

73POPC O22 9680 1.740 3.105 1.406

73POPC C22 9681 1.584 3.241 1.534

73POPC H2R 9682 1.512 3.234 1.450

73POPC H2S 9683 1.587 3.347 1.568

73POPC C3 9684 2.056 3.292 1.540

73POPC HX 9685 2.145 3.319 1.480

73POPC HY 9686 2.073 3.188 1.576

73POPC O31 9687 2.044 3.386 1.648

73POPC C31 9688 1.975 3.339 1.753

73POPC O32 9689 1.940 3.224 1.769

73POPC C32 9690 1.940 3.455 1.846

73POPC H2X 9691 1.973 3.551 1.799

73POPC H2Y 9692 1.994 3.441 1.942

73POPC C23 9693 1.537 3.149 1.648

73POPC H3R 9694 1.612 3.070 1.669

73POPC H3S 9695 1.444 3.096 1.617

73POPC C24 9696 1.509 3.222 1.781

73POPC H4R 9697 1.425 3.294 1.768

73POPC H4S 9698 1.600 3.278 1.811

73POPC C25 9699 1.476 3.118 1.887

73POPC H5R 9700 1.564 3.051 1.893

73POPC H5S 9701 1.388 3.061 1.850

73POPC C26 9702 1.443 3.160 2.031

73POPC H6R 9703 1.410 3.071 2.088

73POPC H6S 9704 1.358 3.231 2.029

73POPC C27 9705 1.560 3.224 2.106

73POPC H7R 9706 1.525 3.260 2.205

73POPC H7S 9707 1.587 3.313 2.045

73POPC C28 9708 1.690 3.141 2.126

73POPC H8R 9709 1.764 3.207 2.175

73POPC H8S 9710 1.732 3.117 2.026

73POPC C29 9711 1.680 3.016 2.209

73POPC H91 9712 1.684 2.923 2.150

73POPC C210 9713 1.680 3.006 2.343

73POPC H101 9714 1.674 2.906 2.388

73POPC C211 9715 1.687 3.118 2.443

73POPC H11R 9716 1.741 3.206 2.401

73POPC H11S 9717 1.749 3.085 2.530

73POPC C212 9718 1.550 3.165 2.496

73POPC H12R 9719 1.498 3.225 2.418

73POPC H12S 9720 1.569 3.232 2.583

73POPC C213 9721 1.456 3.055 2.546

73POPC H13R 9722 1.419 2.996 2.460

73POPC H13S 9723 1.367 3.102 2.595

73POPC C214 9724 1.524 2.961 2.645

73POPC H14R 9725 1.608 2.910 2.593

73POPC H14S 9726 1.454 2.880 2.678

73POPC C215 9727 1.583 3.029 2.768

73POPC H15R 9728 1.505 3.082 2.827

73POPC H15S 9729 1.658 3.104 2.737

73POPC C216 9730 1.652 2.923 2.853

73POPC H16R 9731 1.694 2.845 2.786

73POPC H16S 9732 1.576 2.873 2.917

73POPC C217 9733 1.768 2.979 2.933

73POPC H17R 9734 1.733 3.058 3.004

73POPC H17S 9735 1.840 3.027 2.863

73POPC C218 9736 1.841 2.869 3.008

73POPC H18R 9737 1.785 2.838 3.099

73POPC H18S 9738 1.940 2.908 3.041

73POPC H18T 9739 1.857 2.780 2.945

73POPC C33 9740 1.788 3.462 1.867

73POPC H3X 9741 1.756 3.372 1.925

73POPC H3Y 9742 1.737 3.459 1.769

73POPC C34 9743 1.744 3.590 1.938

73POPC H4X 9744 1.633 3.589 1.943

73POPC H4Y 9745 1.775 3.678 1.878

73POPC C35 9746 1.792 3.606 2.083

73POPC H5X 9747 1.754 3.702 2.122

73POPC H5Y 9748 1.903 3.609 2.088

73POPC C36 9749 1.739 3.491 2.167

73POPC H6X 9750 1.795 3.400 2.137

73POPC H6Y 9751 1.631 3.479 2.144

73POPC C37 9752 1.748 3.507 2.318

73POPC H7X 9753 1.851 3.535 2.346

73POPC H7Y 9754 1.725 3.410 2.366

73POPC C38 9755 1.648 3.611 2.369

73POPC H8X 9756 1.544 3.585 2.339

73POPC H8Y 9757 1.676 3.706 2.319

73POPC C39 9758 1.658 3.623 2.521

73POPC H9X 9759 1.633 3.723 2.560

73POPC H9Y 9760 1.766 3.608 2.544

73POPC C310 9761 1.567 3.521 2.590

73POPC H10X 9762 1.578 3.426 2.534

73POPC H10Y 9763 1.461 3.553 2.582

73POPC C311 9764 1.606 3.498 2.736

73POPC H11X 9765 1.543 3.417 2.779

73POPC H11Y 9766 1.591 3.592 2.792

73POPC C312 9767 1.753 3.458 2.745

73POPC H12X 9768 1.818 3.546 2.728

73POPC H12Y 9769 1.771 3.386 2.662

73POPC C313 9770 1.796 3.397 2.878

73POPC H13X 9771 1.742 3.302 2.898

73POPC H13Y 9772 1.773 3.468 2.961

73POPC C314 9773 1.947 3.373 2.876

73POPC H14X 9774 1.981 3.327 2.972

73POPC H14Y 9775 1.999 3.470 2.867

73POPC C315 9776 1.991 3.281 2.762

73POPC H15X 9777 1.906 3.247 2.700

73POPC H15Y 9778 2.029 3.187 2.808

73POPC C316 9779 2.096 3.348 2.674

73POPC H16X 9780 2.055 3.436 2.621

73POPC H16Y 9781 2.133 3.274 2.600

73POPC H16Z 9782 2.183 3.382 2.735

74POPC N 9783 4.307 4.259 0.933

74POPC C12 9784 4.320 4.194 1.071

74POPC H12A 9785 4.327 4.272 1.146

74POPC H12B 9786 4.413 4.139 1.078

74POPC C13 9787 4.255 4.160 0.834

74POPC H13A 9788 4.246 4.204 0.736

74POPC H13B 9789 4.322 4.075 0.826

74POPC H13C 9790 4.158 4.125 0.868

74POPC C14 9791 4.210 4.373 0.941

74POPC H14A 9792 4.241 4.448 1.013

74POPC H14B 9793 4.193 4.420 0.845

74POPC H14C 9794 4.116 4.336 0.978

74POPC C15 9795 4.441 4.307 0.886

74POPC H15A 9796 4.488 4.371 0.959

74POPC H15B 9797 4.432 4.359 0.792

74POPC H15C 9798 4.506 4.224 0.866

74POPC C11 9799 4.203 4.103 1.116

74POPC H11A 9800 4.225 4.063 1.218

74POPC H11B 9801 4.196 4.014 1.050

74POPC P 9802 4.037 4.263 1.235

74POPC O13 9803 3.990 4.176 1.344

74POPC O14 9804 3.946 4.368 1.182

74POPC O12 9805 4.079 4.173 1.113

74POPC O11 9806 4.175 4.330 1.278

74POPC C1 9807 4.170 4.424 1.386

74POPC HA 9808 4.088 4.406 1.459

74POPC HB 9809 4.156 4.526 1.344

74POPC C2 9810 4.301 4.425 1.470

74POPC HS 9811 4.386 4.417 1.398

74POPC O21 9812 4.301 4.314 1.564

74POPC C21 9813 4.422 4.284 1.612

74POPC O22 9814 4.525 4.338 1.576

74POPC C22 9815 4.414 4.172 1.720

74POPC H2R 9816 4.517 4.159 1.759

74POPC H2S 9817 4.384 4.079 1.667

74POPC C3 9818 4.312 4.562 1.542

74POPC HX 9819 4.333 4.638 1.463

74POPC HY 9820 4.398 4.562 1.612

74POPC O31 9821 4.188 4.593 1.610

74POPC C31 9822 4.179 4.721 1.644

74POPC O32 9823 4.270 4.801 1.636

74POPC C32 9824 4.035 4.757 1.682

74POPC H2X 9825 3.967 4.711 1.607

74POPC H2Y 9826 4.030 4.867 1.672

74POPC C23 9827 4.313 4.198 1.836

74POPC H3R 9828 4.215 4.166 1.797

74POPC H3S 9829 4.301 4.308 1.846

74POPC C24 9830 4.329 4.126 1.975

74POPC H4R 9831 4.334 4.018 1.952

74POPC H4S 9832 4.233 4.143 2.027

74POPC C25 9833 4.441 4.155 2.083

74POPC H5R 9834 4.541 4.146 2.037

74POPC H5S 9835 4.431 4.075 2.159

74POPC C26 9836 4.432 4.288 2.163

74POPC H6R 9837 4.327 4.317 2.181

74POPC H6S 9838 4.484 4.363 2.098

74POPC C27 9839 4.508 4.310 2.296

74POPC H7R 9840 4.598 4.245 2.298

74POPC H7S 9841 4.442 4.285 2.382

74POPC C28 9842 4.559 4.458 2.312

74POPC H8R 9843 4.641 4.471 2.239

74POPC H8S 9844 4.605 4.465 2.413

74POPC C29 9845 4.464 4.575 2.295

74POPC H91 9846 4.476 4.629 2.199

74POPC C210 9847 4.377 4.626 2.384

74POPC H101 9848 4.318 4.714 2.355

74POPC C211 9849 4.347 4.573 2.521

74POPC H11R 9850 4.358 4.653 2.597

74POPC H11S 9851 4.419 4.493 2.550

74POPC C212 9852 4.205 4.515 2.527

74POPC H12R 9853 4.193 4.464 2.625

74POPC H12S 9854 4.194 4.437 2.449

74POPC C213 9855 4.091 4.618 2.511

74POPC H13R 9856 3.994 4.562 2.511

74POPC H13S 9857 4.099 4.668 2.413

74POPC C214 9858 4.084 4.724 2.622

74POPC H14R 9859 3.998 4.791 2.602

74POPC H14S 9860 4.176 4.786 2.622

74POPC C215 9861 4.064 4.661 2.761

74POPC H15R 9862 4.153 4.600 2.787

74POPC H15S 9863 3.977 4.593 2.756

74POPC C216 9864 4.040 4.763 2.871

74POPC H16R 9865 3.964 4.837 2.838

74POPC H16S 9866 4.134 4.818 2.893

74POPC C217 9867 3.990 4.695 2.998

74POPC H17R 9868 3.897 4.639 2.973

74POPC H17S 9869 3.962 4.772 3.074

74POPC C218 9870 4.091 4.600 3.063

74POPC H18R 9871 4.188 4.651 3.078

74POPC H18S 9872 4.107 4.511 2.999

74POPC H18T 9873 4.054 4.566 3.162

74POPC C33 9874 3.990 4.721 1.826

74POPC H3X 9875 3.892 4.771 1.844

74POPC H3Y 9876 4.058 4.765 1.901

74POPC C34 9877 3.971 4.571 1.850

74POPC H4X 9878 4.064 4.517 1.824

74POPC H4Y 9879 3.890 4.533 1.784

74POPC C35 9880 3.933 4.534 1.995

74POPC H5X 9881 3.845 4.592 2.030

74POPC H5Y 9882 4.015 4.564 2.064

74POPC C36 9883 3.908 4.383 2.010

74POPC H6X 9884 3.956 4.332 1.924

74POPC H6Y 9885 3.799 4.363 2.005

74POPC C37 9886 3.967 4.322 2.139

74POPC H7X 9887 3.920 4.368 2.228

74POPC H7Y 9888 4.076 4.345 2.142

74POPC C38 9889 3.947 4.170 2.141

74POPC H8X 9890 3.969 4.131 2.039

74POPC H8Y 9891 3.840 4.149 2.163

74POPC C39 9892 4.037 4.095 2.240

74POPC H9X 9893 4.144 4.117 2.219

74POPC H9Y 9894 4.023 3.986 2.225

74POPC C310 9895 4.008 4.129 2.387

74POPC H10X 9896 3.899 4.139 2.402

74POPC H10Y 9897 4.055 4.227 2.410

74POPC C311 9898 4.061 4.020 2.481

74POPC H11X 9899 4.169 4.002 2.461

74POPC H11Y 9900 4.007 3.925 2.460

74POPC C312 9901 4.043 4.055 2.630

74POPC H12X 9902 4.070 3.965 2.690

74POPC H12Y 9903 3.937 4.079 2.650

74POPC C313 9904 4.134 4.170 2.672

74POPC H13X 9905 4.110 4.261 2.613

74POPC H13Y 9906 4.238 4.142 2.647

74POPC C314 9907 4.124 4.207 2.821

74POPC H14X 9908 4.103 4.116 2.881

74POPC H14Y 9909 4.040 4.278 2.834

74POPC C315 9910 4.255 4.269 2.871

74POPC H15X 9911 4.235 4.326 2.964

74POPC H15Y 9912 4.292 4.342 2.795

74POPC C316 9913 4.363 4.166 2.900

74POPC H16X 9914 4.339 4.115 2.995

74POPC H16Y 9915 4.463 4.213 2.912

74POPC H16Z 9916 4.370 4.090 2.819

75POPC N 9917 3.229 4.773 1.100

75POPC C12 9918 3.355 4.765 1.187

75POPC H12A 9919 3.334 4.707 1.276

75POPC H12B 9920 3.379 4.865 1.221

75POPC C13 9921 3.233 4.896 1.016

75POPC H13A 9922 3.325 4.896 0.959

75POPC H13B 9923 3.150 4.899 0.947

75POPC H13C 9924 3.235 4.985 1.076

75POPC C14 9925 3.227 4.657 1.006

75POPC H14A 9926 3.137 4.657 0.945

75POPC H14B 9927 3.314 4.663 0.942

75POPC H14C 9928 3.232 4.564 1.060

75POPC C15 9929 3.105 4.774 1.185

75POPC H15A 9930 3.021 4.816 1.132

75POPC H15B 9931 3.120 4.828 1.277

75POPC H15C 9932 3.074 4.674 1.211

75POPC C11 9933 3.485 4.708 1.123

75POPC H11A 9934 3.472 4.599 1.108

75POPC H11B 9935 3.567 4.717 1.197

75POPC P 9936 3.596 4.908 1.000

75POPC O13 9937 3.597 4.961 0.862

75POPC O14 9938 3.728 4.884 1.062

75POPC O12 9939 3.518 4.769 0.997

75POPC O11 9940 3.498 4.991 1.092

75POPC C1 9941 3.475 5.131 1.093

75POPC HA 9942 3.380 5.156 1.041

75POPC HB 9943 3.555 5.187 1.038

75POPC C2 9944 3.465 5.186 1.239

75POPC HS 9945 3.467 5.297 1.237

75POPC O21 9946 3.570 5.140 1.323

75POPC C21 9947 3.696 5.139 1.283

75POPC O22 9948 3.740 5.176 1.176

75POPC C22 9949 3.778 5.079 1.397

75POPC H2R 9950 3.842 5.161 1.437

75POPC H2S 9951 3.845 5.001 1.354

75POPC C3 9952 3.330 5.141 1.306

75POPC HX 9953 3.317 5.033 1.282

75POPC HY 9954 3.249 5.196 1.254

75POPC O31 9955 3.318 5.167 1.449

75POPC C31 9956 3.317 5.061 1.531

75POPC O32 9957 3.339 4.946 1.497

75POPC C32 9958 3.268 5.105 1.671

75POPC H2X 9959 3.158 5.118 1.666

75POPC H2Y 9960 3.310 5.206 1.692

75POPC C23 9961 3.690 5.023 1.511

75POPC H3R 9962 3.623 4.943 1.472

75POPC H3S 9963 3.626 5.105 1.550

75POPC C24 9964 3.774 4.971 1.626

75POPC H4R 9965 3.850 5.048 1.650

75POPC H4S 9966 3.827 4.879 1.593

75POPC C25 9967 3.690 4.943 1.750

75POPC H5R 9968 3.607 5.017 1.759

75POPC H5S 9969 3.756 4.959 1.838

75POPC C26 9970 3.640 4.800 1.766

75POPC H6R 9971 3.714 4.730 1.722

75POPC H6S 9972 3.543 4.788 1.714

75POPC C27 9973 3.627 4.765 1.915

75POPC H7R 9974 3.552 4.833 1.960

75POPC H7S 9975 3.722 4.788 1.968

75POPC C28 9976 3.593 4.618 1.941

75POPC H8R 9977 3.580 4.602 2.050

75POPC H8S 9978 3.681 4.557 1.909

75POPC C29 9979 3.475 4.565 1.865

75POPC H91 9980 3.499 4.488 1.791

75POPC C210 9981 3.348 4.606 1.877

75POPC H101 9982 3.271 4.559 1.815

75POPC C211 9983 3.297 4.714 1.969

75POPC H11R 9984 3.237 4.785 1.908

75POPC H11S 9985 3.377 4.774 2.019

75POPC C212 9986 3.203 4.665 2.079

75POPC H12R 9987 3.124 4.608 2.026

75POPC H12S 9988 3.155 4.752 2.128

75POPC C213 9989 3.268 4.577 2.186

75POPC H13R 9990 3.323 4.492 2.138

75POPC H13S 9991 3.186 4.532 2.246

75POPC C214 9992 3.361 4.650 2.284

75POPC H14R 9993 3.325 4.755 2.299

75POPC H14S 9994 3.463 4.655 2.241

75POPC C215 9995 3.361 4.578 2.419

75POPC H15R 9996 3.363 4.468 2.400

75POPC H15S 9997 3.265 4.601 2.469

75POPC C216 9998 3.479 4.613 2.511

75POPC H16R 9999 3.473 4.721 2.535

75POPC H16S10000 3.574 4.596 2.457

75POPC C21710001 3.482 4.531 2.642

75POPC H17R10002 3.530 4.593 2.721

75POPC H17S10003 3.552 4.445 2.632

75POPC C21810004 3.347 4.479 2.692

75POPC H18R10005 3.305 4.403 2.622

75POPC H18S10006 3.273 4.561 2.700

75POPC H18T10007 3.357 4.431 2.792

75POPC C3310008 3.303 5.014 1.792

75POPC H3X10009 3.396 4.955 1.774

75POPC H3Y10010 3.218 4.942 1.795

75POPC C3410011 3.313 5.084 1.930

75POPC H4X10012 3.276 5.013 2.007

75POPC H4Y10013 3.244 5.171 1.932

75POPC C3510014 3.455 5.127 1.975

75POPC H5X10015 3.502 5.191 1.897

75POPC H5Y10016 3.519 5.037 1.986

75POPC C3610017 3.457 5.203 2.109

75POPC H6X10018 3.420 5.134 2.188

75POPC H6Y10019 3.388 5.290 2.104

75POPC C3710020 3.596 5.255 2.149

75POPC H7X10021 3.630 5.326 2.071

75POPC H7Y10022 3.668 5.169 2.152

75POPC C3810023 3.604 5.329 2.284

75POPC H8X10024 3.537 5.418 2.277

75POPC H8Y10025 3.707 5.365 2.301

75POPC C3910026 3.560 5.246 2.405

75POPC H9X10027 3.457 5.209 2.388

75POPC H9Y10028 3.556 5.314 2.493

75POPC C31010029 3.651 5.127 2.440

75POPC H10X10030 3.744 5.166 2.487

75POPC H10Y10031 3.681 5.073 2.348

75POPC C31110032 3.582 5.027 2.534

75POPC H11X10033 3.657 4.956 2.575

75POPC H11Y10034 3.509 4.968 2.475

75POPC C31210035 3.506 5.095 2.649

75POPC H12X10036 3.445 5.019 2.703

75POPC H12Y10037 3.432 5.167 2.609

75POPC C31310038 3.597 5.167 2.749

75POPC H13X10039 3.535 5.240 2.806

75POPC H13Y10040 3.674 5.225 2.695

75POPC C31410041 3.663 5.070 2.848

75POPC H14X10042 3.770 5.098 2.862

75POPC H14Y10043 3.662 4.967 2.807

75POPC C31510044 3.595 5.074 2.985

75POPC H15X10045 3.621 4.982 3.041

75POPC H15Y10046 3.484 5.073 2.971

75POPC C31610047 3.636 5.197 3.066

75POPC H16X10048 3.746 5.196 3.083

75POPC H16Y10049 3.586 5.197 3.165

75POPC H16Z10050 3.610 5.291 3.013

76POPC N10051 1.650 4.208 0.929

76POPC C1210052 1.640 4.151 1.070

76POPC H12A10053 1.674 4.049 1.070

76POPC H12B10054 1.537 4.152 1.103

76POPC C1310055 1.638 4.358 0.936

76POPC H13A10056 1.713 4.397 1.003

76POPC H13B10057 1.649 4.405 0.840

76POPC H13C10058 1.543 4.389 0.979

76POPC C1410059 1.786 4.175 0.874

76POPC H14A10060 1.861 4.208 0.945

76POPC H14B10061 1.801 4.068 0.864

76POPC H14C10062 1.804 4.222 0.779

76POPC C1510063 1.548 4.153 0.836

76POPC H15A10064 1.560 4.197 0.738

76POPC H15B10065 1.448 4.178 0.870

76POPC H15C10066 1.558 4.046 0.826

76POPC C1110067 1.723 4.224 1.180

76POPC H11A10068 1.708 4.172 1.278

76POPC H11B10069 1.683 4.326 1.196

76POPC P10070 1.966 4.123 1.185

76POPC O1310071 2.047 4.177 1.297

76POPC O1410072 2.035 4.083 1.060

76POPC O1210073 1.860 4.233 1.144

76POPC O1110074 1.872 4.007 1.236

76POPC C110075 1.870 3.877 1.178

76POPC HA10076 1.952 3.860 1.105

76POPC HB10077 1.774 3.863 1.123

76POPC C210078 1.882 3.764 1.285

76POPC HS10079 1.865 3.667 1.233

76POPC O2110080 1.788 3.777 1.393

76POPC C2110081 1.661 3.769 1.363

76POPC O2210082 1.612 3.746 1.253

76POPC C2210083 1.577 3.804 1.486

76POPC H2R10084 1.471 3.791 1.458

76POPC H2S10085 1.595 3.912 1.508

76POPC C310086 2.027 3.758 1.344

76POPC HX10087 2.094 3.748 1.256

76POPC HY10088 2.041 3.666 1.404

76POPC O3110089 2.062 3.878 1.417

76POPC C3110090 2.022 3.875 1.544

76POPC O3210091 1.981 3.776 1.602

76POPC C3210092 2.041 4.014 1.607

76POPC H2X10093 1.959 4.079 1.568

76POPC H2Y10094 2.137 4.054 1.568

76POPC C2310095 1.606 3.720 1.613

76POPC H3R10096 1.711 3.733 1.645

76POPC H3S10097 1.592 3.612 1.590

76POPC C2410098 1.511 3.759 1.727

76POPC H4R10099 1.518 3.683 1.808

76POPC H4S10100 1.406 3.757 1.691

76POPC C2510101 1.543 3.898 1.787

76POPC H5R10102 1.464 3.968 1.753

76POPC H5S10103 1.641 3.933 1.748

76POPC C2610104 1.550 3.899 1.940

76POPC H6R10105 1.628 3.829 1.975

76POPC H6S10106 1.453 3.863 1.980

76POPC C2710107 1.583 4.038 1.997

76POPC H7R10108 1.507 4.112 1.964

76POPC H7S10109 1.681 4.072 1.957

76POPC C2810110 1.595 4.041 2.150

76POPC H8R10111 1.623 4.144 2.183

76POPC H8S10112 1.676 3.973 2.182

76POPC C2910113 1.463 4.002 2.211

76POPC H9110114 1.376 4.012 2.146

76POPC C21010115 1.447 3.951 2.334

76POPC H10110116 1.346 3.921 2.364

76POPC C21110117 1.553 3.933 2.439

76POPC H11R10118 1.639 3.879 2.392

76POPC H11S10119 1.509 3.868 2.517

76POPC C21210120 1.622 4.051 2.512

76POPC H12R10121 1.635 4.138 2.444

76POPC H12S10122 1.723 4.016 2.541

76POPC C21310123 1.551 4.091 2.642

76POPC H13R10124 1.524 3.998 2.697

76POPC H13S10125 1.458 4.145 2.616

76POPC C21410126 1.632 4.182 2.736

76POPC H14R10127 1.651 4.278 2.684

76POPC H14S10128 1.731 4.136 2.757

76POPC C21510129 1.557 4.205 2.868

76POPC H15R10130 1.447 4.205 2.849

76POPC H15S10131 1.584 4.304 2.911

76POPC C21610132 1.592 4.097 2.969

76POPC H16R10133 1.695 4.116 3.006

76POPC H16S10134 1.593 3.999 2.917

76POPC C21710135 1.499 4.085 3.091

76POPC H17R10136 1.464 4.186 3.122

76POPC H17S10137 1.558 4.043 3.175

76POPC C21810138 1.381 3.992 3.064

76POPC H18R10139 1.326 3.968 3.158

76POPC H18S10140 1.417 3.896 3.021

76POPC H18T10141 1.310 4.040 2.993

76POPC C3310142 2.045 4.020 1.763

76POPC H3X10143 2.059 4.126 1.794

76POPC H3Y10144 2.133 3.967 1.804

76POPC C3410145 1.921 3.965 1.834

76POPC H4X10146 1.880 3.876 1.782

76POPC H4Y10147 1.841 4.042 1.833

76POPC C3510148 1.951 3.918 1.977

76POPC H5X10149 2.029 3.839 1.975

76POPC H5Y10150 1.859 3.871 2.019

76POPC C3610151 1.995 4.029 2.072

76POPC H6X10152 1.915 4.107 2.075

76POPC H6Y10153 2.088 4.077 2.036

76POPC C3710154 2.017 3.975 2.213

76POPC H7X10155 2.107 3.910 2.212

76POPC H7Y10156 1.930 3.911 2.241

76POPC C3810157 2.036 4.082 2.320

76POPC H8X10158 1.951 4.154 2.316

76POPC H8Y10159 2.130 4.137 2.303

76POPC C3910160 2.039 4.013 2.457

76POPC H9X10161 2.118 3.936 2.457

76POPC H9Y10162 1.941 3.961 2.471

76POPC C31010163 2.062 4.107 2.575

76POPC H10X10164 1.997 4.196 2.567

76POPC H10Y10165 2.168 4.141 2.575

76POPC C31110166 2.030 4.032 2.705

76POPC H11X10167 2.072 3.930 2.701

76POPC H11Y10168 1.919 4.022 2.712

76POPC C31210169 2.082 4.102 2.831

76POPC H12X10170 2.087 4.211 2.812

76POPC H12Y10171 2.186 4.067 2.852

76POPC C31310172 1.992 4.075 2.952

76POPC H13X10173 1.890 4.113 2.930

76POPC H13Y10174 2.031 4.132 3.040

76POPC C31410175 1.982 3.926 2.987

76POPC H14X10176 2.085 3.888 3.001

76POPC H14Y10177 1.937 3.870 2.903

76POPC C31510178 1.903 3.899 3.116

76POPC H15X10179 1.879 3.994 3.168

76POPC H15Y10180 1.971 3.841 3.183

76POPC C31610181 1.777 3.817 3.095

76POPC H16X10182 1.736 3.782 3.193

76POPC H16Y10183 1.799 3.728 3.033

76POPC H16Z10184 1.697 3.876 3.045

77POPC N10185 2.017 1.848 0.870

77POPC C1210186 2.131 1.945 0.847

77POPC H12A10187 2.109 2.008 0.763

77POPC H12B10188 2.220 1.887 0.825

77POPC C1310189 2.067 1.733 0.953

77POPC H13A10190 2.101 1.773 1.048

77POPC H13B10191 1.989 1.661 0.971

77POPC H13C10192 2.152 1.684 0.908

77POPC C1410193 1.905 1.913 0.948

77POPC H14A10194 1.944 1.952 1.041

77POPC H14B10195 1.860 1.996 0.896

77POPC H14C10196 1.828 1.842 0.975

77POPC C1510197 1.965 1.797 0.740

77POPC H15A10198 2.044 1.751 0.682

77POPC H15B10199 1.918 1.875 0.683

77POPC H15C10200 1.891 1.721 0.759

77POPC C1110201 2.166 2.036 0.966

77POPC H11A10202 2.091 2.118 0.966

77POPC H11B10203 2.266 2.081 0.949

77POPC P10204 2.131 2.042 1.217

77POPC O1310205 2.156 1.958 1.337

77POPC O1410206 1.996 2.099 1.200

77POPC O1210207 2.167 1.960 1.088

77POPC O1110208 2.241 2.153 1.191

77POPC C110209 2.378 2.130 1.224

77POPC HA10210 2.445 2.177 1.149

77POPC HB10211 2.406 2.022 1.224

77POPC C210212 2.404 2.192 1.361

77POPC HS10213 2.512 2.213 1.373

77POPC O2110214 2.359 2.100 1.458

77POPC C2110215 2.437 2.003 1.493

77POPC O2210216 2.542 1.971 1.439

77POPC C2210217 2.385 1.957 1.635

77POPC H2R10218 2.438 1.859 1.638

77POPC H2S10219 2.283 1.922 1.610

77POPC C310220 2.323 2.323 1.390

77POPC HX10221 2.346 2.397 1.311

77POPC HY10222 2.366 2.362 1.485

77POPC O3110223 2.179 2.298 1.398

77POPC C3110224 2.116 2.367 1.491

77POPC O3210225 2.165 2.461 1.549

77POPC C3210226 1.977 2.315 1.524

77POPC H2X10227 1.975 2.204 1.518

77POPC H2Y10228 1.911 2.354 1.444

77POPC C2310229 2.396 2.099 1.911

77POPC H3R10230 2.408 2.208 1.896

77POPC H3S10231 2.493 2.077 1.960

77POPC C2410232 2.294 2.108 2.025

77POPC H4R10233 2.256 2.010 2.060

77POPC H4S10234 2.209 2.172 1.995

77POPC C2510235 2.363 2.178 2.143

77POPC H5R10236 2.288 2.210 2.219

77POPC H5S10237 2.413 2.270 2.107

77POPC C2610238 2.465 2.086 2.210

77POPC H6R10239 2.530 2.031 2.140

77POPC H6S10240 2.412 2.008 2.268

77POPC C2710241 2.558 2.164 2.301

77POPC H7R10242 2.499 2.224 2.374

77POPC H7S10243 2.618 2.234 2.239

77POPC C2810244 2.648 2.064 2.372

77POPC H8R10245 2.707 2.009 2.295

77POPC H8S10246 2.586 1.990 2.426

77POPC C2910247 2.739 2.140 2.461

77POPC H9110248 2.809 2.206 2.408

77POPC C21010249 2.745 2.132 2.594

77POPC H10110250 2.808 2.204 2.648

77POPC C21110251 2.667 2.043 2.685

77POPC H11R10252 2.729 1.956 2.715

77POPC H11S10253 2.574 2.004 2.640

77POPC C21210254 2.634 2.126 2.809

77POPC H12R10255 2.726 2.163 2.860

77POPC H12S10256 2.586 2.056 2.881

77POPC C21310257 2.544 2.244 2.776

77POPC H13R10258 2.458 2.205 2.718

77POPC H13S10259 2.596 2.318 2.712

77POPC C21410260 2.490 2.315 2.900

77POPC H14R10261 2.401 2.373 2.870

77POPC H14S10262 2.565 2.384 2.944

77POPC C21510263 2.439 2.216 3.003

77POPC H15R10264 2.522 2.175 3.064

77POPC H15S10265 2.396 2.131 2.946

77POPC C21610266 2.329 2.277 3.087

77POPC H16R10267 2.290 2.372 3.043

77POPC H16S10268 2.371 2.300 3.188

77POPC C21710269 2.215 2.178 3.096

77POPC H17R10270 2.254 2.092 3.155

77POPC H17S10271 2.194 2.141 2.993

77POPC C21810272 2.087 2.229 3.157

77POPC H18R10273 2.005 2.156 3.141

77POPC H18S10274 2.058 2.326 3.111

77POPC H18T10275 2.100 2.242 3.266

77POPC C3310276 1.924 2.367 1.662

77POPC H3X10277 1.815 2.383 1.651

77POPC H3Y10278 1.966 2.467 1.689

77POPC C3410279 1.937 2.270 1.780

77POPC H4X10280 2.044 2.256 1.806

77POPC H4Y10281 1.894 2.174 1.745

77POPC C3510282 1.859 2.306 1.908

77POPC H5X10283 1.763 2.352 1.878

77POPC H5Y10284 1.915 2.382 1.967

77POPC C3610285 1.826 2.183 1.996

77POPC H6X10286 1.772 2.108 1.934

77POPC H6Y10287 1.756 2.216 2.076

77POPC C3710288 1.946 2.115 2.063

77POPC H7X10289 2.000 2.190 2.125

77POPC H7Y10290 2.016 2.080 1.984

77POPC C3810291 1.906 1.994 2.149

77POPC H8X10292 1.857 1.919 2.083

77POPC H8Y10293 1.831 2.026 2.224

77POPC C3910294 2.023 1.926 2.222

77POPC H9X10295 2.101 1.897 2.149

77POPC H9Y10296 1.986 1.831 2.269

77POPC C31010297 2.082 2.013 2.333

77POPC H10X10298 2.002 2.042 2.404

77POPC H10Y10299 2.123 2.106 2.289

77POPC C31110300 2.194 1.943 2.412

77POPC H11X10301 2.261 1.890 2.341

77POPC H11Y10302 2.149 1.865 2.476

77POPC C31210303 2.279 2.040 2.496

77POPC H12X10304 2.319 2.120 2.430

77POPC H12Y10305 2.365 1.989 2.542

77POPC C31310306 2.198 2.112 2.604

77POPC H13X10307 2.112 2.162 2.555

77POPC H13Y10308 2.260 2.192 2.650

77POPC C31410309 2.147 2.018 2.714

77POPC H14X10310 2.223 2.012 2.795

77POPC H14Y10311 2.132 1.914 2.675

77POPC C31510312 2.013 2.067 2.766

77POPC H15X10313 1.981 2.003 2.852

77POPC H15Y10314 1.937 2.055 2.686

77POPC C31610315 2.019 2.213 2.809

77POPC H16X10316 1.975 2.222 2.910

77POPC H16Y10317 1.963 2.278 2.738

77POPC H16Z10318 2.123 2.251 2.815

78POPC N10319 1.590 2.539 0.895

78POPC C1210320 1.466 2.452 0.888

78POPC H12A10321 1.497 2.349 0.882

78POPC H12B10322 1.410 2.475 0.798

78POPC C1310323 1.676 2.491 1.008

78POPC H13A10324 1.711 2.391 0.989

78POPC H13B10325 1.760 2.556 1.027

78POPC H13C10326 1.615 2.483 1.096

78POPC C1410327 1.669 2.530 0.768

78POPC H14A10328 1.697 2.429 0.746

78POPC H14B10329 1.615 2.573 0.685

78POPC H14C10330 1.760 2.588 0.776

78POPC C1510331 1.550 2.682 0.918

78POPC H15A10332 1.483 2.717 0.840

78POPC H15B10333 1.637 2.746 0.920

78POPC H15C10334 1.497 2.689 1.012

78POPC C1110335 1.368 2.460 1.007

78POPC H11A10336 1.279 2.398 0.983

78POPC H11B10337 1.333 2.564 1.024

78POPC P10338 1.351 2.407 1.260

78POPC O1310339 1.416 2.307 1.347

78POPC O1410340 1.208 2.390 1.228

78POPC O1210341 1.432 2.409 1.124

78POPC O1110342 1.379 2.555 1.310

78POPC C110343 1.511 2.611 1.311

78POPC HA10344 1.513 2.701 1.245

78POPC HB10345 1.587 2.541 1.272

78POPC C210346 1.557 2.653 1.454

78POPC HS10347 1.666 2.669 1.453

78POPC O2110348 1.524 2.554 1.553

78POPC C2110349 1.587 2.440 1.543

78POPC O2210350 1.672 2.412 1.459

78POPC C2210351 1.556 2.356 1.667

78POPC H2R10352 1.599 2.411 1.753

78POPC H2S10353 1.609 2.259 1.656

78POPC C310354 1.493 2.785 1.501

78POPC HX10355 1.383 2.772 1.491

78POPC HY10356 1.525 2.868 1.434

78POPC O3110357 1.538 2.811 1.635

78POPC C3110358 1.443 2.824 1.727

78POPC O3210359 1.328 2.858 1.706

78POPC C3210360 1.505 2.806 1.865

78POPC H2X10361 1.448 2.876 1.931

78POPC H2Y10362 1.611 2.837 1.863

78POPC C2310363 1.405 2.333 1.691

78POPC H3R10364 1.362 2.278 1.605

78POPC H3S10365 1.352 2.430 1.697

78POPC C2410366 1.378 2.254 1.821

78POPC H4R10367 1.433 2.157 1.820

78POPC H4S10368 1.269 2.231 1.823

78POPC C2510369 1.413 2.333 1.947

78POPC H5R10370 1.380 2.437 1.927

78POPC H5S10371 1.523 2.335 1.964

78POPC C2610372 1.339 2.290 2.074

78POPC H6R10373 1.231 2.280 2.051

78POPC H6S10374 1.349 2.370 2.150

78POPC C2710375 1.390 2.159 2.135

78POPC H7R10376 1.495 2.170 2.167

78POPC H7S10377 1.387 2.080 2.056

78POPC C2810378 1.303 2.111 2.253

78POPC H8R10379 1.335 2.009 2.280

78POPC H8S10380 1.199 2.101 2.215

78POPC C2910381 1.307 2.207 2.370

78POPC H9110382 1.259 2.304 2.351

78POPC C21010383 1.357 2.183 2.492

78POPC H10110384 1.350 2.262 2.568

78POPC C21110385 1.425 2.056 2.539

78POPC H11R10386 1.445 1.987 2.455

78POPC H11S10387 1.360 2.002 2.611

78POPC C21210388 1.559 2.084 2.609

78POPC H12R10389 1.619 2.155 2.549

78POPC H12S10390 1.617 1.989 2.615

78POPC C21310391 1.541 2.138 2.752

78POPC H13R10392 1.498 2.058 2.816

78POPC H13S10393 1.469 2.222 2.749

78POPC C21410394 1.673 2.188 2.812

78POPC H14R10395 1.708 2.273 2.749

78POPC H14S10396 1.749 2.107 2.807

78POPC C21510397 1.659 2.236 2.958

78POPC H15R10398 1.655 2.148 3.026

78POPC H15S10399 1.560 2.286 2.966

78POPC C21610400 1.770 2.333 3.001

78POPC H16R10401 1.824 2.370 2.911

78POPC H16S10402 1.841 2.274 3.063

78POPC C21710403 1.730 2.458 3.080

78POPC H17R10404 1.656 2.431 3.158

78POPC H17S10405 1.684 2.533 3.012

78POPC C21810406 1.851 2.523 3.148

78POPC H18R10407 1.824 2.621 3.193

78POPC H18S10408 1.933 2.538 3.075

78POPC H18T10409 1.887 2.456 3.229

78POPC C3310410 1.488 2.670 1.934

78POPC H3X10411 1.558 2.596 1.891

78POPC H3Y10412 1.384 2.636 1.918

78POPC C3410413 1.511 2.689 2.085

78POPC H4X10414 1.413 2.698 2.138

78POPC H4Y10415 1.562 2.786 2.098

78POPC C3510416 1.599 2.585 2.155

78POPC H5X10417 1.692 2.567 2.096

78POPC H5Y10418 1.544 2.488 2.161

78POPC C3610419 1.637 2.632 2.296

78POPC H6X10420 1.547 2.677 2.343

78POPC H6Y10421 1.715 2.711 2.289

78POPC C3710422 1.687 2.519 2.387

78POPC H7X10423 1.779 2.476 2.342

78POPC H7Y10424 1.611 2.438 2.390

78POPC C3810425 1.718 2.564 2.531

78POPC H8X10426 1.796 2.643 2.527

78POPC H8Y10427 1.762 2.478 2.586

78POPC C3910428 1.598 2.618 2.611

78POPC H9X10429 1.559 2.709 2.561

78POPC H9Y10430 1.635 2.647 2.712

78POPC C31010431 1.482 2.519 2.629

78POPC H10X10432 1.521 2.417 2.644

78POPC H10Y10433 1.421 2.519 2.536

78POPC C31110434 1.392 2.557 2.750

78POPC H11X10435 1.286 2.533 2.726

78POPC H11Y10436 1.400 2.666 2.768

78POPC C31210437 1.431 2.479 2.877

78POPC H12X10438 1.542 2.475 2.879

78POPC H12Y10439 1.393 2.375 2.865

78POPC C31310440 1.379 2.534 3.010

78POPC H13X10441 1.274 2.563 2.991

78POPC H13Y10442 1.435 2.626 3.037

78POPC C31410443 1.375 2.435 3.129

78POPC H14X10444 1.457 2.360 3.114

78POPC H14Y10445 1.280 2.377 3.128

78POPC C31510446 1.401 2.494 3.269

78POPC H15X10447 1.489 2.562 3.261

78POPC H15Y10448 1.433 2.410 3.335

78POPC C31610449 1.287 2.567 3.339

78POPC H16X10450 1.191 2.513 3.325

78POPC H16Y10451 1.275 2.671 3.303

78POPC H16Z10452 1.307 2.574 3.448

79POPC N10453 2.684 1.315 1.025

79POPC C1210454 2.689 1.418 1.135

79POPC H12A10455 2.788 1.461 1.134

79POPC H12B10456 2.678 1.368 1.230

79POPC C1310457 2.702 1.383 0.893

79POPC H13A10458 2.690 1.317 0.809

79POPC H13B10459 2.629 1.462 0.884

79POPC H13C10460 2.797 1.434 0.887

79POPC C1410461 2.794 1.214 1.046

79POPC H14A10462 2.795 1.141 0.967

79POPC H14B10463 2.891 1.262 1.050

79POPC H14C10464 2.779 1.162 1.140

79POPC C1510465 2.553 1.244 1.029

79POPC H15A10466 2.543 1.192 1.123

79POPC H15B10467 2.545 1.172 0.949

79POPC H15C10468 2.475 1.317 1.017

79POPC C1110469 2.593 1.541 1.133

79POPC H11A10470 2.601 1.598 1.228

79POPC H11B10471 2.488 1.505 1.129

79POPC P10472 2.740 1.728 1.001

79POPC O1310473 2.680 1.856 0.959

79POPC O1410474 2.827 1.665 0.897

79POPC O1210475 2.618 1.627 1.023

79POPC O1110476 2.819 1.746 1.132

79POPC C110477 3.014 1.769 1.304

79POPC HA10478 2.998 1.834 1.392

79POPC HB10479 3.108 1.817 1.270

79POPC C210480 3.092 1.655 1.399

79POPC HS10481 3.112 1.567 1.334

79POPC O2110482 3.008 1.621 1.512

79POPC C2110483 2.988 1.492 1.535

79POPC O2210484 3.051 1.401 1.486

79POPC C2210485 2.857 1.476 1.612

79POPC H2R10486 2.775 1.504 1.541

79POPC H2S10487 2.851 1.549 1.696

79POPC C310488 3.236 1.692 1.447

79POPC HX10489 3.283 1.759 1.372

79POPC HY10490 3.298 1.600 1.446

79POPC O3110491 3.237 1.757 1.576

79POPC C3110492 3.249 1.674 1.679

79POPC O3210493 3.257 1.552 1.673

79POPC C3210494 3.251 1.754 1.808

79POPC H2X10495 3.225 1.860 1.786

79POPC H2Y10496 3.356 1.750 1.845

79POPC C2310497 2.830 1.333 1.663

79POPC H3R10498 2.888 1.315 1.756

79POPC H3S10499 2.864 1.258 1.588

79POPC C2410500 2.681 1.309 1.688

79POPC H4R10501 2.667 1.206 1.727

79POPC H4S10502 2.626 1.317 1.592

79POPC C2510503 2.618 1.408 1.788

79POPC H5R10504 2.508 1.388 1.791

79POPC H5S10505 2.631 1.513 1.754

79POPC C2610506 2.674 1.390 1.929

79POPC H6R10507 2.781 1.420 1.933

79POPC H6S10508 2.668 1.283 1.955

79POPC C2710509 2.595 1.470 2.032

79POPC H7R10510 2.486 1.455 2.012

79POPC H7S10511 2.616 1.578 2.021

79POPC C2810512 2.627 1.422 2.173

79POPC H8R10513 2.733 1.444 2.196

79POPC H8S10514 2.615 1.312 2.177

79POPC C2910515 2.536 1.492 2.269

79POPC H9110516 2.528 1.601 2.256

79POPC C21010517 2.467 1.435 2.368

79POPC H10110518 2.406 1.500 2.433

79POPC C21110519 2.465 1.289 2.402

79POPC H11R10520 2.472 1.226 2.310

79POPC H11S10521 2.368 1.267 2.451

79POPC C21210522 2.574 1.245 2.501

79POPC H12R10523 2.672 1.287 2.473

79POPC H12S10524 2.582 1.134 2.498

79POPC C21310525 2.533 1.287 2.642

79POPC H13R10526 2.431 1.249 2.662

79POPC H13S10527 2.531 1.398 2.646

79POPC C21410528 2.625 1.233 2.751

79POPC H14R10529 2.730 1.253 2.720

79POPC H14S10530 2.614 1.123 2.761

79POPC C21510531 2.598 1.302 2.885

79POPC H15R10532 2.567 1.408 2.867

79POPC H15S10533 2.694 1.309 2.941

79POPC C21610534 2.498 1.229 2.975

79POPC H16R10535 2.526 1.122 2.982

79POPC H16S10536 2.397 1.235 2.929

79POPC C21710537 2.495 1.290 3.116

79POPC H17R10538 2.390 1.319 3.139

79POPC H17S10539 2.556 1.382 3.116

79POPC C21810540 2.548 1.200 3.227

79POPC H18R10541 2.516 1.235 3.327

79POPC H18S10542 2.659 1.199 3.227

79POPC H18T10543 2.511 1.096 3.215

79POPC C3310544 3.154 1.697 1.914

79POPC H3X10545 3.167 1.754 2.008

79POPC H3Y10546 3.172 1.589 1.933

79POPC C3410547 3.009 1.719 1.875

79POPC H4X10548 2.987 1.662 1.782

79POPC H4Y10549 3.008 1.827 1.849

79POPC C3510550 2.904 1.694 1.984

79POPC H5X10551 2.937 1.613 2.053

79POPC H5Y10552 2.812 1.658 1.933

79POPC C3610553 2.868 1.822 2.061

79POPC H6X10554 2.762 1.850 2.040

79POPC H6Y10555 2.932 1.904 2.023

79POPC C3710556 2.891 1.815 2.212

79POPC H7X10557 2.903 1.919 2.250

79POPC H7Y10558 2.986 1.762 2.233

79POPC C3810559 2.775 1.750 2.288

79POPC H8X10560 2.771 1.643 2.258

79POPC H8Y10561 2.678 1.796 2.259

79POPC C3910562 2.793 1.759 2.440

79POPC H9X10563 2.798 1.865 2.474

79POPC H9Y10564 2.894 1.715 2.459

79POPC C31010565 2.688 1.683 2.521

79POPC H10X10566 2.661 1.591 2.465

79POPC H10Y10567 2.597 1.745 2.534

79POPC C31110568 2.738 1.632 2.657

79POPC H11X10569 2.652 1.590 2.714

79POPC H11Y10570 2.777 1.717 2.717

79POPC C31210571 2.843 1.519 2.642

79POPC H12X10572 2.899 1.531 2.547

79POPC H12Y10573 2.788 1.424 2.629

79POPC C31310574 2.944 1.507 2.757

79POPC H13X10575 3.017 1.590 2.753

79POPC H13Y10576 3.002 1.413 2.742

79POPC C31410577 2.878 1.506 2.895

79POPC H14X10578 2.913 1.420 2.956

79POPC H14Y10579 2.768 1.495 2.880

79POPC C31510580 2.901 1.633 2.977

79POPC H15X10581 2.912 1.718 2.907

79POPC H15Y10582 2.996 1.623 3.033

79POPC C31610583 2.785 1.664 3.072

79POPC H16X10584 2.705 1.721 3.018

79POPC H16Y10585 2.819 1.725 3.158

79POPC H16Z10586 2.740 1.570 3.111

80POPC N10587 5.134 2.865 1.056

80POPC C1210588 5.033 2.763 1.101

80POPC H12A10589 5.084 2.676 1.139

80POPC H12B10590 4.972 2.733 1.017

80POPC C1310591 5.198 2.922 1.179

80POPC H13A10592 5.269 2.999 1.154

80POPC H13B10593 5.122 2.964 1.243

80POPC H13C10594 5.246 2.844 1.236

80POPC C1410595 5.234 2.799 0.968

80POPC H14A10596 5.302 2.872 0.928

80POPC H14B10597 5.291 2.725 1.022

80POPC H14C10598 5.185 2.752 0.884

80POPC C1510599 5.068 2.975 0.977

80POPC H15A10600 5.019 2.935 0.890

80POPC H15B10601 5.139 3.051 0.946

80POPC H15C10602 4.991 3.020 1.037

80POPC C1110603 4.936 2.808 1.212

80POPC H11A10604 4.989 2.832 1.307

80POPC H11B10605 4.877 2.716 1.232

80POPC P10606 4.751 2.905 1.054

80POPC O1310607 4.777 2.783 0.974

80POPC O1410608 4.760 3.032 0.980

80POPC O1210609 4.851 2.916 1.175

80POPC O1110610 4.607 2.894 1.115

80POPC C110611 4.520 3.006 1.133

80POPC HA10612 4.427 2.987 1.075

80POPC HB10613 4.562 3.102 1.097

80POPC C210614 4.476 3.024 1.282

80POPC HS10615 4.396 3.101 1.283

80POPC O2110616 4.583 3.062 1.368

80POPC C2110617 4.641 3.176 1.358

80POPC O2210618 4.610 3.269 1.285

80POPC C2210619 4.760 3.171 1.454

80POPC H2R10620 4.847 3.132 1.397

80POPC H2S10621 4.737 3.098 1.534

80POPC C310622 4.418 2.896 1.344

80POPC HX10623 4.337 2.857 1.278

80POPC HY10624 4.372 2.925 1.441

80POPC O3110625 4.523 2.801 1.372

80POPC C3110626 4.517 2.686 1.309

80POPC O3210627 4.421 2.644 1.247

80POPC C3210628 4.652 2.614 1.316

80POPC H2X10629 4.699 2.635 1.217

80POPC H2Y10630 4.633 2.505 1.316

80POPC C2310631 4.794 3.306 1.519

80POPC H3R10632 4.714 3.335 1.590

80POPC H3S10633 4.800 3.386 1.442

80POPC C2410634 4.927 3.299 1.595

80POPC H4R10635 4.950 3.399 1.639

80POPC H4S10636 5.010 3.275 1.525

80POPC C2510637 4.926 3.193 1.707

80POPC H5R10638 4.923 3.093 1.659

80POPC H5S10639 4.835 3.202 1.770

80POPC C2610640 5.051 3.200 1.796

80POPC H6R10641 5.039 3.276 1.876

80POPC H6S10642 5.136 3.231 1.732

80POPC C2710643 5.087 3.067 1.861

80POPC H7R10644 5.197 3.064 1.877

80POPC H7S10645 5.062 2.984 1.792

80POPC C2810646 5.018 3.048 1.997

80POPC H8R10647 4.908 3.052 1.984

80POPC H8S10648 5.046 3.132 2.064

80POPC C2910649 5.061 2.915 2.051

80POPC H9110650 5.022 2.828 1.995

80POPC C21010651 5.141 2.891 2.156

80POPC H10110652 5.159 2.786 2.183

80POPC C21110653 5.209 2.992 2.247

80POPC H11R10654 5.198 3.096 2.210

80POPC H11S10655 5.318 2.971 2.254

80POPC C21210656 5.147 2.979 2.386

80POPC H12R10657 5.207 3.032 2.464

80POPC H12S10658 5.149 2.873 2.416

80POPC C21310659 5.002 3.028 2.390

80POPC H13R10660 4.952 2.964 2.466

80POPC H13S10661 4.951 3.008 2.293

80POPC C21410662 4.975 3.174 2.425

80POPC H14R10663 4.865 3.190 2.420

80POPC H14S10664 5.020 3.243 2.350

80POPC C21510665 5.020 3.212 2.566

80POPC H15R10666 5.130 3.228 2.567

80POPC H15S10667 5.003 3.126 2.635

80POPC C21610668 4.952 3.340 2.613

80POPC H16R10669 4.939 3.409 2.527

80POPC H16S10670 5.015 3.392 2.689

80POPC C21710671 4.819 3.318 2.685

80POPC H17R10672 4.743 3.283 2.612

80POPC H17S10673 4.789 3.416 2.726

80POPC C21810674 4.834 3.218 2.798

80POPC H18R10675 4.931 3.235 2.849

80POPC H18S10676 4.832 3.113 2.762

80POPC H18T10677 4.758 3.228 2.879

80POPC C3310678 4.748 2.658 1.430

80POPC H3X10679 4.772 2.766 1.419

80POPC H3Y10680 4.843 2.602 1.419

80POPC C3410681 4.700 2.639 1.574

80POPC H4X10682 4.667 2.534 1.590

80POPC H4Y10683 4.612 2.705 1.589

80POPC C3510684 4.811 2.675 1.675

80POPC H5X10685 4.903 2.705 1.620

80POPC H5Y10686 4.839 2.585 1.734

80POPC C3610687 4.773 2.786 1.774

80POPC H6X10688 4.764 2.882 1.720

80POPC H6Y10689 4.855 2.796 1.848

80POPC C3710690 4.641 2.756 1.847

80POPC H7X10691 4.640 2.650 1.879

80POPC H7Y10692 4.556 2.771 1.777

80POPC C3810693 4.618 2.846 1.969

80POPC H8X10694 4.512 2.834 2.000

80POPC H8Y10695 4.633 2.953 1.941

80POPC C3910696 4.704 2.808 2.089

80POPC H9X10697 4.810 2.839 2.071

80POPC H9Y10698 4.703 2.698 2.102

80POPC C31010699 4.652 2.876 2.216

80POPC H10X10700 4.544 2.850 2.226

80POPC H10Y10701 4.660 2.986 2.203

80POPC C31110702 4.723 2.834 2.344

80POPC H11X10703 4.833 2.838 2.328

80POPC H11Y10704 4.697 2.728 2.367

80POPC C31210705 4.680 2.923 2.461

80POPC H12X10706 4.570 2.916 2.470

80POPC H12Y10707 4.702 3.029 2.435

80POPC C31310708 4.745 2.893 2.598

80POPC H13X10709 4.670 2.909 2.679

80POPC H13Y10710 4.824 2.969 2.616

80POPC C31410711 4.808 2.754 2.612

80POPC H14X10712 4.880 2.736 2.530

80POPC H14Y10713 4.727 2.677 2.605

80POPC C31510714 4.884 2.736 2.744

80POPC H15X10715 4.909 2.628 2.756

80POPC H15Y10716 4.818 2.765 2.829

80POPC C31610717 5.016 2.813 2.751

80POPC H16X10718 5.066 2.797 2.849

80POPC H16Y10719 4.999 2.922 2.737

80POPC H16Z10720 5.085 2.779 2.671

81POPC N10721 4.863 3.555 1.132

81POPC C1210722 4.979 3.570 1.034

81POPC H12A10723 4.941 3.617 0.944

81POPC H12B10724 5.013 3.471 1.006

81POPC C1310725 4.775 3.676 1.118

81POPC H13A10726 4.691 3.674 1.185

81POPC H13B10727 4.834 3.764 1.138

81POPC H13C10728 4.742 3.689 1.015

81POPC C1410729 4.786 3.431 1.100

81POPC H14A10730 4.848 3.343 1.107

81POPC H14B10731 4.704 3.416 1.169

81POPC H14C10732 4.749 3.433 0.999

81POPC C1510733 4.914 3.551 1.273

81POPC H15A10734 4.978 3.637 1.290

81POPC H15B10735 4.973 3.462 1.290

81POPC H15C10736 4.833 3.553 1.344

81POPC C1110737 5.101 3.653 1.079

81POPC H11A10738 5.175 3.661 0.996

81POPC H11B10739 5.154 3.599 1.161

81POPC P10740 5.032 3.903 1.031

81POPC O1310741 4.923 3.864 0.939

81POPC O1410742 5.155 3.956 0.971

81POPC O1210743 5.063 3.782 1.127

81POPC O1110744 4.978 4.011 1.133

81POPC C110745 4.961 3.991 1.273

81POPC HA10746 4.917 3.892 1.297

81POPC HB10747 4.889 4.067 1.309

81POPC C210748 5.093 4.009 1.355

81POPC HS10749 5.163 4.062 1.288

81POPC O2110750 5.147 3.880 1.387

81POPC C2110751 5.271 3.881 1.435

81POPC O2210752 5.339 3.980 1.454

81POPC C2210753 5.318 3.737 1.450

81POPC H2R10754 5.428 3.736 1.461

81POPC H2S10755 5.291 3.683 1.357

81POPC C310756 5.074 4.099 1.482

81POPC HX10757 5.017 4.191 1.454

81POPC HY10758 5.173 4.136 1.516

81POPC O3110759 5.015 4.026 1.591

81POPC C3110760 4.884 4.037 1.606

81POPC O3210761 4.812 4.112 1.543

81POPC C3210762 4.838 3.932 1.705

81POPC H2X10763 4.757 3.975 1.767

81POPC H2Y10764 4.924 3.910 1.772

81POPC C2310765 5.252 3.667 1.570

81POPC H3R10766 5.260 3.557 1.558

81POPC H3S10767 5.144 3.693 1.574

81POPC C2410768 5.320 3.706 1.701

81POPC H4R10769 5.348 3.813 1.701

81POPC H4S10770 5.414 3.647 1.712

81POPC C2510771 5.227 3.680 1.819

81POPC H5R10772 5.199 3.573 1.820

81POPC H5S10773 5.136 3.742 1.803

81POPC C2610774 5.284 3.717 1.956

81POPC H6R10775 5.392 3.695 1.961

81POPC H6S10776 5.236 3.652 2.033

81POPC C2710777 5.259 3.863 1.995

81POPC H7R10778 5.150 3.883 2.005

81POPC H7S10779 5.297 3.927 1.911

81POPC C2810780 5.333 3.902 2.124

81POPC H8R10781 5.323 4.011 2.140

81POPC H8S10782 5.441 3.880 2.109

81POPC C2910783 5.286 3.826 2.245

81POPC H9110784 5.230 3.733 2.225

81POPC C21010785 5.306 3.865 2.371

81POPC H10110786 5.268 3.803 2.455

81POPC C21110787 5.379 3.990 2.408

81POPC H11R10788 5.454 4.017 2.331

81POPC H11S10789 5.437 3.974 2.502

81POPC C21210790 5.282 4.108 2.424

81POPC H12R10791 5.210 4.112 2.340

81POPC H12S10792 5.340 4.202 2.421

81POPC C21310793 5.208 4.102 2.558

81POPC H13R10794 5.284 4.093 2.638

81POPC H13S10795 5.146 4.011 2.563

81POPC C21410796 5.123 4.227 2.584

81POPC H14R10797 5.043 4.235 2.507

81POPC H14S10798 5.186 4.318 2.576

81POPC C21510799 5.059 4.222 2.723

81POPC H15R10800 5.136 4.192 2.798

81POPC H15S10801 4.982 4.142 2.719

81POPC C21610802 4.999 4.356 2.766

81POPC H16R10803 4.914 4.376 2.698

81POPC H16S10804 5.078 4.432 2.748

81POPC C21710805 4.946 4.373 2.910

81POPC H17R10806 4.844 4.329 2.914

81POPC H17S10807 4.931 4.482 2.928

81POPC C21810808 5.028 4.318 3.027

81POPC H18R10809 4.963 4.290 3.113

81POPC H18S10810 5.099 4.395 3.062

81POPC H18T10811 5.085 4.226 3.000

81POPC C3310812 4.790 3.805 1.630

81POPC H3X10813 4.864 3.776 1.554

81POPC H3Y10814 4.694 3.829 1.578

81POPC C3410815 4.768 3.685 1.723

81POPC H4X10816 4.721 3.602 1.666

81POPC H4Y10817 4.699 3.713 1.805

81POPC C3510818 4.901 3.638 1.782

81POPC H5X10819 4.941 3.721 1.844

81POPC H5Y10820 4.971 3.620 1.698

81POPC C3610821 4.895 3.512 1.869

81POPC H6X10822 4.997 3.469 1.868

81POPC H6Y10823 4.825 3.438 1.825

81POPC C3710824 4.862 3.540 2.015

81POPC H7X10825 4.832 3.446 2.066

81POPC H7Y10826 4.775 3.610 2.019

81POPC C3810827 4.981 3.601 2.089

81POPC H8X10828 5.032 3.672 2.021

81POPC H8Y10829 5.054 3.522 2.118

81POPC C3910830 4.938 3.684 2.209

81POPC H9X10831 4.862 3.758 2.174

81POPC H9Y10832 5.024 3.742 2.248

81POPC C31010833 4.879 3.600 2.322

81POPC H10X10834 4.963 3.569 2.388

81POPC H10Y10835 4.833 3.507 2.283

81POPC C31110836 4.771 3.677 2.400

81POPC H11X10837 4.740 3.617 2.488

81POPC H11Y10838 4.682 3.687 2.334

81POPC C31210839 4.814 3.818 2.443

81POPC H12X10840 4.733 3.862 2.505

81POPC H12Y10841 4.826 3.884 2.354

81POPC C31310842 4.944 3.819 2.523

81POPC H13X10843 4.957 3.921 2.565

81POPC H13Y10844 5.031 3.799 2.457

81POPC C31410845 4.943 3.716 2.635

81POPC H14X10846 4.937 3.612 2.595

81POPC H14Y10847 4.850 3.730 2.695

81POPC C31510848 5.065 3.726 2.724

81POPC H15X10849 5.103 3.831 2.728

81POPC H15Y10850 5.147 3.663 2.682

81POPC C31610851 5.030 3.681 2.864

81POPC H16X10852 4.958 3.750 2.912

81POPC H16Y10853 5.121 3.677 2.928

81POPC H16Z10854 4.982 3.581 2.861

82POPC N10855 4.199 1.770 1.044

82POPC C1210856 4.171 1.656 1.141

82POPC H12A10857 4.067 1.665 1.172

82POPC H12B10858 4.231 1.669 1.230

82POPC C1310859 4.333 1.756 0.978

82POPC H13A10860 4.335 1.667 0.917

82POPC H13B10861 4.354 1.842 0.916

82POPC H13C10862 4.413 1.750 1.050

82POPC C1410863 4.095 1.772 0.937

82POPC H14A10864 4.101 1.680 0.880

82POPC H14B10865 3.996 1.772 0.981

82POPC H14C10866 4.104 1.859 0.873

82POPC C1510867 4.187 1.902 1.116

82POPC H15A10868 4.257 1.911 1.197

82POPC H15B10869 4.087 1.918 1.154

82POPC H15C10870 4.206 1.986 1.050

82POPC C1110871 4.187 1.510 1.091

82POPC H11A10872 4.177 1.439 1.176

82POPC H11B10873 4.290 1.495 1.051

82POPC P10874 3.940 1.470 1.024

82POPC O1310875 3.866 1.406 0.913

82POPC O1410876 3.893 1.605 1.062

82POPC O1210877 4.093 1.479 0.988

82POPC O1110878 3.933 1.379 1.155

82POPC C110879 3.815 1.299 1.165

82POPC HA10880 3.813 1.224 1.082

82POPC HB10881 3.725 1.364 1.160

82POPC C210882 3.799 1.217 1.296

82POPC HS10883 3.696 1.177 1.293

82POPC O2110884 3.804 1.309 1.408

82POPC C2110885 3.819 1.265 1.532

82POPC O2210886 3.850 1.153 1.566

82POPC C2210887 3.788 1.378 1.630

82POPC H2R10888 3.718 1.451 1.583

82POPC H2S10889 3.883 1.432 1.652

82POPC C310890 3.890 1.090 1.295

82POPC HX10891 3.874 1.037 1.198

82POPC HY10892 3.856 1.020 1.374

82POPC O3110893 4.028 1.128 1.305

82POPC C3110894 4.101 1.048 1.380

82POPC O3210895 4.071 0.936 1.418

82POPC C3210896 4.223 1.126 1.423

82POPC H2X10897 4.246 1.204 1.348

82POPC H2Y10898 4.308 1.054 1.424

82POPC C2310899 3.728 1.324 1.762

82POPC H3R10900 3.779 1.230 1.794

82POPC H3S10901 3.621 1.299 1.744

82POPC C2410902 3.733 1.423 1.880

82POPC H4R10903 3.675 1.380 1.964

82POPC H4S10904 3.684 1.517 1.849

82POPC C2510905 3.875 1.454 1.932

82POPC H5R10906 3.863 1.524 2.018

82POPC H5S10907 3.934 1.506 1.854

82POPC C2610908 3.954 1.332 1.982

82POPC H6R10909 3.993 1.273 1.897

82POPC H6S10910 3.883 1.268 2.039

82POPC C2710911 4.070 1.378 2.073

82POPC H7R10912 4.026 1.443 2.151

82POPC H7S10913 4.137 1.439 2.009

82POPC C2810914 4.156 1.270 2.141

82POPC H8R10915 4.239 1.320 2.197

82POPC H8S10916 4.201 1.209 2.059

82POPC C2910917 4.076 1.183 2.232

82POPC H9110918 3.970 1.195 2.206

82POPC C21010919 4.098 1.105 2.339

82POPC H10110920 4.011 1.051 2.378

82POPC C21110921 4.216 1.074 2.428

82POPC H11R10922 4.315 1.079 2.377

82POPC H11S10923 4.208 0.973 2.473

82POPC C21210924 4.206 1.175 2.542

82POPC H12R10925 4.238 1.271 2.496

82POPC H12S10926 4.277 1.148 2.623

82POPC C21310927 4.064 1.194 2.600

82POPC H13R10928 4.018 1.094 2.614

82POPC H13S10929 3.999 1.247 2.527

82POPC C21410930 4.059 1.273 2.730

82POPC H14R10931 4.098 1.376 2.719

82POPC H14S10932 4.124 1.224 2.805

82POPC C21510933 3.916 1.290 2.781

82POPC H15R10934 3.862 1.193 2.772

82POPC H15S10935 3.864 1.363 2.715

82POPC C21610936 3.910 1.337 2.926

82POPC H16R10937 3.805 1.364 2.949

82POPC H16S10938 3.971 1.430 2.939

82POPC C21710939 3.958 1.228 3.022

82POPC H17R10940 4.068 1.217 3.014

82POPC H17S10941 3.912 1.133 2.987

82POPC C21810942 3.919 1.245 3.169

82POPC H18R10943 3.810 1.231 3.180

82POPC H18S10944 3.948 1.346 3.206

82POPC H18T10945 3.968 1.169 3.234

82POPC C3310946 4.203 1.186 1.564

82POPC H3X10947 4.137 1.124 1.628

82POPC H3Y10948 4.303 1.185 1.611

82POPC C3410949 4.162 1.334 1.569

82POPC H4X10950 4.054 1.340 1.595

82POPC H4Y10951 4.175 1.383 1.471

82POPC C3510952 4.241 1.409 1.677

82POPC H5X10953 4.349 1.402 1.653

82POPC H5Y10954 4.227 1.360 1.776

82POPC C3610955 4.207 1.558 1.690

82POPC H6X10956 4.115 1.572 1.750

82POPC H6Y10957 4.187 1.599 1.588

82POPC C3710958 4.324 1.639 1.750

82POPC H7X10959 4.303 1.748 1.743

82POPC H7Y10960 4.413 1.618 1.687

82POPC C3810961 4.362 1.603 1.895

82POPC H8X10962 4.466 1.638 1.913

82POPC H8Y10963 4.361 1.493 1.908

82POPC C3910964 4.271 1.671 1.998

82POPC H9X10965 4.165 1.646 1.975

82POPC H9Y10966 4.282 1.781 1.991

82POPC C31010967 4.303 1.626 2.142

82POPC H10X10968 4.403 1.664 2.171

82POPC H10Y10969 4.309 1.515 2.145

82POPC C31110970 4.195 1.674 2.240

82POPC H11X10971 4.102 1.617 2.223

82POPC H11Y10972 4.171 1.780 2.218

82POPC C31210973 4.233 1.664 2.388

82POPC H12X10974 4.140 1.683 2.446

82POPC H12Y10975 4.307 1.742 2.415

82POPC C31310976 4.296 1.533 2.436

82POPC H13X10977 4.406 1.535 2.422

82POPC H13Y10978 4.256 1.450 2.373

82POPC C31410979 4.259 1.505 2.582

82POPC H14X10980 4.304 1.408 2.614

82POPC H14Y10981 4.148 1.499 2.582

82POPC C31510982 4.301 1.603 2.691

82POPC H15X10983 4.246 1.698 2.677

82POPC H15Y10984 4.410 1.623 2.682

82POPC C31610985 4.271 1.549 2.832

82POPC H16X10986 4.311 1.616 2.911

82POPC H16Y10987 4.315 1.448 2.845

82POPC H16Z10988 4.161 1.541 2.847

83POPC N10989 2.770 5.444 0.925

83POPC C1210990 2.718 5.304 0.955

83POPC H12A10991 2.777 5.263 1.036

83POPC H12B10992 2.733 5.240 0.869

83POPC C1310993 2.817 5.504 1.055

83POPC H13A10994 2.903 5.452 1.094

83POPC H13B10995 2.842 5.609 1.045

83POPC H13C10996 2.739 5.495 1.129

83POPC C1410997 2.884 5.440 0.828

83POPC H14A10998 2.855 5.390 0.737

83POPC H14B10999 2.917 5.540 0.802

83POPC H14C11000 2.968 5.387 0.871

83POPC C1511001 2.658 5.527 0.871

83POPC H15A11002 2.577 5.523 0.943

83POPC H15B11003 2.622 5.486 0.778

83POPC H15C11004 2.687 5.630 0.857

83POPC C1111005 2.570 5.291 0.998

83POPC H11A11006 2.549 5.186 1.028

83POPC H11B11007 2.504 5.310 0.910

83POPC P11008 2.544 5.344 1.254

83POPC O1311009 2.451 5.230 1.272

83POPC O1411010 2.524 5.467 1.333

83POPC O1211011 2.537 5.385 1.101

83POPC O1111012 2.696 5.299 1.266

83POPC C111013 2.737 5.162 1.281

83POPC HA11014 2.837 5.150 1.236

83POPC HB11015 2.668 5.094 1.227

83POPC C211016 2.745 5.112 1.429

83POPC HS11017 2.763 5.003 1.421

83POPC O2111018 2.620 5.137 1.494

83POPC C2111019 2.600 5.070 1.606

83POPC O2211020 2.666 4.976 1.645

83POPC C2211021 2.479 5.131 1.678

83POPC H2R11022 2.389 5.072 1.648

83POPC H2S11023 2.466 5.236 1.644

83POPC C311024 2.865 5.171 1.510

83POPC HX11025 2.958 5.161 1.450

83POPC HY11026 2.880 5.107 1.600

83POPC O3111027 2.841 5.306 1.556

83POPC C3111028 2.907 5.404 1.497

83POPC O3211029 2.995 5.392 1.414

83POPC C3211030 2.857 5.539 1.551

83POPC H2X11031 2.814 5.594 1.465

83POPC H2Y11032 2.945 5.596 1.588

83POPC C2311033 2.497 5.126 1.831

83POPC H3R11034 2.511 5.021 1.865

83POPC H3S11035 2.404 5.164 1.879

83POPC C2411036 2.615 5.212 1.878

83POPC H4R11037 2.602 5.316 1.842

83POPC H4S11038 2.709 5.173 1.835

83POPC C2511039 2.628 5.211 2.031

83POPC H5R11040 2.624 5.105 2.064

83POPC H5S11041 2.543 5.264 2.077

83POPC C2611042 2.761 5.269 2.082

83POPC H6R11043 2.762 5.379 2.068

83POPC H6S11044 2.845 5.226 2.024

83POPC C2711045 2.782 5.236 2.231

83POPC H7R11046 2.800 5.127 2.241

83POPC H7S11047 2.688 5.257 2.286

83POPC C2811048 2.895 5.318 2.295

83POPC H8R11049 2.895 5.299 2.404

83POPC H8S11050 2.872 5.426 2.282

83POPC C2911051 3.031 5.291 2.235

83POPC H9111052 3.071 5.372 2.173

83POPC C21011053 3.104 5.180 2.253

83POPC H10111054 3.203 5.174 2.205

83POPC C21111055 3.067 5.058 2.334

83POPC H11R11056 3.099 4.966 2.280

83POPC H11S11057 2.957 5.050 2.349

83POPC C21211058 3.130 5.057 2.475

83POPC H12R11059 3.240 5.072 2.467

83POPC H12S11060 3.113 4.958 2.522

83POPC C21311061 3.069 5.165 2.565

83POPC H13R11062 2.959 5.154 2.572

83POPC H13S11063 3.089 5.263 2.519

83POPC C21411064 3.128 5.164 2.706

83POPC H14R11065 3.231 5.124 2.699

83POPC H14S11066 3.071 5.099 2.775

83POPC C21511067 3.141 5.306 2.760

83POPC H15R11068 3.186 5.365 2.677

83POPC H15S11069 3.214 5.305 2.843

83POPC C21611070 3.012 5.375 2.808

83POPC H16R11071 3.036 5.426 2.904

83POPC H16S11072 2.934 5.300 2.831

83POPC C21711073 2.959 5.484 2.713

83POPC H17R11074 2.887 5.437 2.643

83POPC H17S11075 3.042 5.524 2.652

83POPC C21811076 2.894 5.602 2.786

83POPC H18R11077 2.970 5.661 2.842

83POPC H18S11078 2.818 5.567 2.860

83POPC H18T11079 2.844 5.670 2.714

83POPC C3311080 2.750 5.529 1.663

83POPC H3X11081 2.797 5.487 1.754

83POPC H3Y11082 2.669 5.460 1.632

83POPC C3411083 2.687 5.665 1.692

83POPC H4X11084 2.634 5.700 1.601

83POPC H4Y11085 2.768 5.738 1.716

83POPC C3511086 2.588 5.665 1.809

83POPC H5X11087 2.498 5.606 1.783

83POPC H5Y11088 2.555 5.770 1.825

83POPC C3611089 2.651 5.611 1.938

83POPC H6X11090 2.754 5.652 1.944

83POPC H6Y11091 2.657 5.500 1.933

83POPC C3711092 2.574 5.653 2.064

83POPC H7X11093 2.481 5.594 2.074

83POPC H7Y11094 2.545 5.758 2.047

83POPC C3811095 2.661 5.646 2.190

83POPC H8X11096 2.664 5.745 2.240

83POPC H8Y11097 2.766 5.624 2.160

83POPC C3911098 2.617 5.540 2.291

83POPC H9X11099 2.696 5.533 2.369

83POPC H9Y11100 2.612 5.443 2.237

83POPC C31011101 2.481 5.560 2.359

83POPC H10X11102 2.401 5.556 2.282

83POPC H10Y11103 2.476 5.660 2.407

83POPC C31111104 2.455 5.451 2.464

83POPC H11X11105 2.483 5.353 2.420

83POPC H11Y11106 2.347 5.446 2.488

83POPC C31211107 2.534 5.474 2.594

83POPC H12X11108 2.499 5.569 2.641

83POPC H12Y11109 2.641 5.487 2.569

83POPC C31311110 2.521 5.359 2.695

83POPC H13X11111 2.549 5.264 2.645

83POPC H13Y11112 2.416 5.352 2.728

83POPC C31411113 2.613 5.382 2.815

83POPC H14X11114 2.582 5.474 2.868

83POPC H14Y11115 2.715 5.399 2.774

83POPC C31511116 2.626 5.266 2.915

83POPC H15X11117 2.700 5.297 2.992

83POPC H15Y11118 2.668 5.177 2.864

83POPC C31611119 2.496 5.227 2.986

83POPC H16X11120 2.447 5.144 2.930

83POPC H16Y11121 2.426 5.313 2.991

83POPC H16Z11122 2.517 5.190 3.089

84POPC N11123 1.893 4.983 0.890

84POPC C1211124 1.767 5.029 0.964

84POPC H12A11125 1.743 4.956 1.041

84POPC H12B11126 1.683 5.033 0.897

84POPC C1311127 1.996 4.943 0.991

84POPC H13A11128 1.957 4.868 1.058

84POPC H13B11129 2.090 4.911 0.949

84POPC H13C11130 2.014 5.027 1.055

84POPC C1411131 1.858 4.868 0.800

84POPC H14A11132 1.777 4.895 0.734

84POPC H14B11133 1.943 4.838 0.740

84POPC H14C11134 1.824 4.784 0.859

84POPC C1511135 1.946 5.098 0.811

84POPC H15A11136 1.970 5.177 0.880

84POPC H15B11137 1.872 5.136 0.743

84POPC H15C11138 2.036 5.072 0.756

84POPC C1111139 1.776 5.165 1.038

84POPC H11A11140 1.678 5.186 1.084

84POPC H11B11141 1.793 5.248 0.966

84POPC P11142 1.856 5.090 1.272

84POPC O1311143 1.979 5.104 1.354

84POPC O1411144 1.810 4.952 1.243

84POPC O1211145 1.882 5.163 1.134

84POPC O1111146 1.737 5.177 1.329

84POPC C111147 1.737 5.321 1.319

84POPC HA11148 1.651 5.353 1.256

84POPC HB11149 1.830 5.361 1.274

84POPC C211150 1.724 5.385 1.459

84POPC HS11151 1.736 5.495 1.447

84POPC O2111152 1.828 5.327 1.539

84POPC C2111153 1.904 5.406 1.610

84POPC O2211154 1.888 5.526 1.626

84POPC C2211155 2.020 5.320 1.662

84POPC H2R11156 2.061 5.269 1.572

84POPC H2S11157 1.978 5.242 1.728

84POPC C311158 1.584 5.359 1.522

84POPC HX11159 1.509 5.407 1.457

84POPC HY11160 1.579 5.409 1.621

84POPC O3111161 1.553 5.218 1.528

84POPC C3111162 1.613 5.156 1.630

84POPC O3211163 1.657 5.212 1.729

84POPC C3211164 1.608 5.004 1.608

84POPC H2X11165 1.665 4.983 1.515

84POPC H2Y11166 1.502 4.975 1.592

84POPC C2311167 2.134 5.398 1.730

84POPC H3R11168 2.163 5.485 1.667

84POPC H3S11169 2.222 5.331 1.736

84POPC C2411170 2.104 5.447 1.872

84POPC H4R11171 2.028 5.527 1.870

84POPC H4S11172 2.198 5.491 1.912

84POPC C2511173 2.064 5.339 1.974

84POPC H5R11174 2.104 5.370 2.072

84POPC H5S11175 2.109 5.240 1.949

84POPC C2611176 1.913 5.328 1.990

84POPC H6R11177 1.867 5.267 1.909

84POPC H6S11178 1.871 5.431 1.979

84POPC C2711179 1.872 5.273 2.126

84POPC H7R11180 1.926 5.325 2.208

84POPC H7S11181 1.899 5.165 2.132

84POPC C2811182 1.721 5.289 2.147

84POPC H8R11183 1.689 5.232 2.237

84POPC H8S11184 1.676 5.241 2.058

84POPC C2911185 1.680 5.434 2.155

84POPC H9111186 1.650 5.481 2.060

84POPC C21011187 1.679 5.507 2.267

84POPC H10111188 1.645 5.612 2.264

84POPC C21111189 1.720 5.456 2.401

84POPC H11R11190 1.776 5.535 2.455

84POPC H11S11191 1.788 5.368 2.395

84POPC C21211192 1.596 5.415 2.481

84POPC H12R11193 1.550 5.324 2.437

84POPC H12S11194 1.521 5.497 2.478

84POPC C21311195 1.633 5.389 2.626

84POPC H13R11196 1.716 5.458 2.650

84POPC H13S11197 1.674 5.287 2.636

84POPC C21411198 1.521 5.416 2.725

84POPC H14R11199 1.459 5.503 2.694

84POPC H14S11200 1.570 5.445 2.820

84POPC C21511201 1.431 5.296 2.757

84POPC H15R11202 1.354 5.284 2.678

84POPC H15S11203 1.382 5.326 2.853

84POPC C21611204 1.505 5.164 2.778

84POPC H16R11205 1.585 5.180 2.854

84POPC H16S11206 1.550 5.134 2.681

84POPC C21711207 1.420 5.044 2.822

84POPC H17R11208 1.349 5.021 2.739

84POPC H17S11209 1.362 5.072 2.913

84POPC C21811210 1.501 4.919 2.852

84POPC H18R11211 1.572 4.941 2.935

84POPC H18S11212 1.560 4.888 2.763

84POPC H18T11213 1.435 4.835 2.882

84POPC C3311214 1.668 4.924 1.726

84POPC H3X11215 1.669 4.817 1.698

84POPC H3Y11216 1.604 4.935 1.817

84POPC C3411217 1.811 4.967 1.760

84POPC H4X11218 1.809 5.061 1.819

84POPC H4Y11219 1.865 4.990 1.665

84POPC C3511220 1.892 4.866 1.840

84POPC H5X11221 1.993 4.911 1.856

84POPC H5Y11222 1.905 4.773 1.781

84POPC C3611223 1.835 4.832 1.978

84POPC H6X11224 1.740 4.777 1.966

84POPC H6Y11225 1.815 4.926 2.034

84POPC C3711226 1.936 4.746 2.056

84POPC H7X11227 2.032 4.801 2.060

84POPC H7Y11228 1.953 4.652 1.998

84POPC C3811229 1.896 4.710 2.199

84POPC H8X11230 1.962 4.626 2.231

84POPC H8Y11231 1.791 4.673 2.203

84POPC C3911232 1.917 4.825 2.298

84POPC H9X11233 1.840 4.904 2.282

84POPC H9Y11234 2.016 4.871 2.279

84POPC C31011235 1.914 4.776 2.443

84POPC H10X11236 1.995 4.700 2.455

84POPC H10Y11237 1.818 4.726 2.466

84POPC C31111238 1.940 4.888 2.545

84POPC H11X11239 2.026 4.950 2.512

84POPC H11Y11240 1.969 4.839 2.641

84POPC C31211241 1.819 4.979 2.572

84POPC H12X11242 1.730 4.914 2.586

84POPC H12Y11243 1.800 5.045 2.485

84POPC C31311244 1.837 5.062 2.701

84POPC H13X11245 1.881 4.996 2.779

84POPC H13Y11246 1.738 5.091 2.740

84POPC C31411247 1.921 5.190 2.685

84POPC H14X11248 1.872 5.264 2.617

84POPC H14Y11249 2.015 5.163 2.631

84POPC C31511250 1.956 5.255 2.820

84POPC H15X11251 2.063 5.285 2.817

84POPC H15Y11252 1.946 5.179 2.901

84POPC C31611253 1.876 5.379 2.859

84POPC H16X11254 1.768 5.355 2.866

84POPC H16Y11255 1.890 5.459 2.782

84POPC H16Z11256 1.910 5.420 2.956

85POPC N11257 4.842 2.153 0.936

85POPC C1211258 4.964 2.093 1.005

85POPC H12A11259 5.054 2.128 0.957

85POPC H12B11260 4.958 1.986 0.992

85POPC C1311261 4.791 2.271 1.014

85POPC H13A11262 4.697 2.306 0.974

85POPC H13B11263 4.773 2.238 1.116

85POPC H13C11264 4.863 2.351 1.018

85POPC C1411265 4.872 2.193 0.795

85POPC H14A11266 4.902 2.107 0.737

85POPC H14B11267 4.783 2.235 0.750

85POPC H14C11268 4.948 2.269 0.791

85POPC C1511269 4.732 2.051 0.937

85POPC H15A11270 4.708 2.027 1.040

85POPC H15B11271 4.761 1.958 0.892

85POPC H15C11272 4.642 2.088 0.891

85POPC C1111273 4.979 2.116 1.157

85POPC H11A11274 4.986 2.224 1.182

85POPC H11B11275 5.074 2.072 1.194

85POPC P11276 4.862 1.904 1.241

85POPC O1311277 4.963 1.862 1.338

85POPC O1411278 4.870 1.842 1.106

85POPC O1211279 4.866 2.061 1.223

85POPC O1111280 4.717 1.882 1.299

85POPC C111281 4.617 1.817 1.222

85POPC HA11282 4.644 1.711 1.203

85POPC HB11283 4.607 1.865 1.122

85POPC C211284 4.480 1.817 1.296

85POPC HS11285 4.403 1.784 1.224

85POPC O2111286 4.442 1.946 1.346

85POPC C2111287 4.455 2.047 1.261

85POPC O2211288 4.452 2.038 1.139

85POPC C2211289 4.476 2.174 1.340

85POPC H2R11290 4.385 2.195 1.400

85POPC H2S11291 4.491 2.258 1.268

85POPC C311292 4.474 1.713 1.411

85POPC HX11293 4.461 1.614 1.362

85POPC HY11294 4.383 1.730 1.473

85POPC O3111295 4.594 1.705 1.490

85POPC C3111296 4.600 1.786 1.595

85POPC O3211297 4.511 1.861 1.632

85POPC C3211298 4.731 1.764 1.671

85POPC H2X11299 4.784 1.677 1.627

85POPC H2Y11300 4.705 1.741 1.776

85POPC C2311301 4.598 2.155 1.431

85POPC H3R11302 4.685 2.129 1.367

85POPC H3S11303 4.584 2.071 1.502

85POPC C2411304 4.635 2.280 1.510

85POPC H4R11305 4.565 2.294 1.596

85POPC H4S11306 4.621 2.367 1.443

85POPC C2511307 4.780 2.276 1.558

85POPC H5R11308 4.827 2.377 1.552

85POPC H5S11309 4.839 2.210 1.491

85POPC C2611310 4.788 2.225 1.701

85POPC H6R11311 4.882 2.167 1.716

85POPC H6S11312 4.702 2.156 1.716

85POPC C2711313 4.780 2.336 1.805

85POPC H7R11314 4.721 2.420 1.762

85POPC H7S11315 4.883 2.375 1.823

85POPC C2811316 4.717 2.290 1.937

85POPC H8R11317 4.765 2.344 2.021

85POPC H8S11318 4.741 2.182 1.950

85POPC C2911319 4.569 2.320 1.936

85POPC H9111320 4.545 2.427 1.942

85POPC C21011321 4.467 2.234 1.920

85POPC H10111322 4.365 2.275 1.922

85POPC C21111323 4.472 2.084 1.906

85POPC H11R11324 4.528 2.055 1.815

85POPC H11S11325 4.369 2.045 1.892

85POPC C21211326 4.533 2.011 2.028

85POPC H12R11327 4.643 2.022 2.025

85POPC H12S11328 4.513 1.902 2.017

85POPC C21311329 4.482 2.061 2.164

85POPC H13R11330 4.428 2.158 2.157

85POPC H13S11331 4.570 2.079 2.230

85POPC C21411332 4.390 1.960 2.231

85POPC H14R11333 4.438 1.859 2.227

85POPC H14S11334 4.296 1.955 2.172

85POPC C21511335 4.359 1.995 2.376

85POPC H15R11336 4.269 1.935 2.403

85POPC H15S11337 4.336 2.104 2.387

85POPC C21611338 4.466 1.950 2.475

85POPC H16R11339 4.546 2.027 2.478

85POPC H16S11340 4.505 1.856 2.431

85POPC C21711341 4.422 1.915 2.617

85POPC H17R11342 4.484 1.829 2.651

85POPC H17S11343 4.317 1.878 2.616

85POPC C21811344 4.438 2.027 2.719

85POPC H18R11345 4.410 1.992 2.820

85POPC H18S11346 4.374 2.114 2.693

85POPC H18T11347 4.544 2.062 2.722

85POPC C3311348 4.819 1.889 1.663

85POPC H3X11349 4.773 1.968 1.726

85POPC H3Y11350 4.824 1.926 1.558

85POPC C3411351 4.963 1.867 1.709

85POPC H4X11352 5.024 1.956 1.681

85POPC H4Y11353 5.006 1.780 1.655

85POPC C3511354 4.975 1.844 1.860

85POPC H5X11355 4.921 1.751 1.886

85POPC H5Y11356 4.926 1.928 1.915

85POPC C3611357 5.121 1.831 1.905

85POPC H6X11358 5.168 1.931 1.912

85POPC H6Y11359 5.176 1.775 1.827

85POPC C3711360 5.135 1.755 2.037

85POPC H7X11361 5.241 1.726 2.052

85POPC H7Y11362 5.076 1.660 2.030

85POPC C3811363 5.088 1.836 2.159

85POPC H8X11364 4.983 1.869 2.142

85POPC H8Y11365 5.149 1.928 2.168

85POPC C3911366 5.097 1.754 2.288

85POPC H9X11367 5.192 1.696 2.289

85POPC H9Y11368 5.013 1.682 2.290

85POPC C31011369 5.093 1.843 2.414

85POPC H10X11370 5.017 1.922 2.399

85POPC H10Y11371 5.190 1.896 2.423

85POPC C31111372 5.062 1.763 2.541

85POPC H11X11373 5.147 1.697 2.566
[truncated: 505,302 more chars]
