# Supplementary material for: Benzaldehyde, A New Absorption Promoter, Accelerating Absorption on Low Bioavailability Drugs Through Membrane Permeability
Source: Front Pharmacol. 2021 May 28;12:663743. doi: 10.3389/fphar.2021.663743 (PMC8194254; doi:10.3389/fphar.2021.663743)
Supplement: Supplementary file 1 [file DataSheet1.zip › Supplementary file 7.DOCX]

integrator = md

dt = 0.002

nsteps = 5000000

nstxout = 50000

nstvout = 50000

nstfout = 50000

nstcalcenergy = 100

nstenergy = 1000

nstlog = 1000

;

cutoff-scheme = Verlet

nstlist = 20

rlist = 1.2

vdwtype = Cut-off

vdw-modifier = Force-switch

rvdw_switch = 1.0

rvdw = 1.2

coulombtype = pme

rcoulomb = 1.2

;

tcoupl = Nose-Hoover

tc_grps = POPC SOL benza

tau_t = 1.0 1.0 1.0

ref_t = 313.15 313.15 313.15

;

pcoupl = Parrinello-Rahman

pcoupltype = semiisotropic

tau_p = 5.0

compressibility = 4.5e-5 4.5e-5 4.5e-5

ref_p = 1.0 1.0 1.0

;

constraints = h-bonds

constraint_algorithm = LINCS

continuation = yes

;

nstcomm = 100

comm_mode = linear

comm_grps = POPC SOL benza

;

refcoord_scaling = com
