# Supplementary material for: Benzaldehyde, A New Absorption Promoter, Accelerating Absorption on Low Bioavailability Drugs Through Membrane Permeability
Source: Front Pharmacol. 2021 May 28;12:663743. doi: 10.3389/fphar.2021.663743 (PMC8194254; doi:10.3389/fphar.2021.663743)
Supplement: Supplementary file 1 [file DataSheet1.zip › Supplementary file 8.DOCX]

integrator = steep

emtol = 1000.0

nsteps = 5000

nstlist = 10

cutoff-scheme = Verlet

rlist = 1.2

vdwtype = Cut-off

vdw-modifier = Force-switch

rvdw_switch = 1.0

rvdw = 1.2

coulombtype = pme

rcoulomb = 1.2

;

constraints = h-bonds

constraint_algorithm = LINCS
